# Supplementary material for: Quadruple C-H activation coupled to hydrofunctionalization and C-H silylation/borylation enabled by weakly coordinated palladium catalyst
Source: Nat Commun. 2020 Nov 9;11:5662. doi: 10.1038/s41467-020-19508-z (PMC7652853; doi:10.1038/s41467-020-19508-z)
Supplement: Supplementary file 1 — Supplementary Information [file 41467_2020_19508_MOESM1_ESM.pdf]

Supplementary Information

Quadruple C-H Activation Coupled to Hydrofunctionalization and C-H Silylation/Borylation Enabled by Weakly Coordinated Palladium Catalyst

Bo-Cheng Tang[a], Wen-Xuan Lin[b], Xiang-Long Chen[a], Cai He[a], Jin-Tian Ma[a], Yan-Dong Wu[a], Yu Lan*[bc] and An-Xin Wu*[a]

[a]Key Laboratory of Pesticide & Chemical Biology, Ministry of Education, College of Chemistry, Central China Normal University, Wuhan 430079, P. R. China.

[b]College of Chemistry and Institute of Green Catalysis, Zhengzhou University, 100 Science Avenue, Zhengzhou, Henan, 450001, P. R. China

[c]School of Chemistry and Chemical Engineering, and Chongqing Key Laboratory of Theoretical and Computational Chemistry, Chongqing University, Chongqing 400030, P.R.China.

E-mail: chwuax@mail.ccnu.edu.cn. lanyu@cqu.edu.cn.

Table of Contents **page**

1. Supplementary Notes……………………………………....................................2
2. Supplementary Methods………………………………….………….……..….…2
3. Supplementary Tables…………………………………….....…………….........3
4. Supplementary Figures………………………..…………………….............7
5. Supplementary References………..……………………..............………....…236

1. Supplementary Notes

$Pd(OAc)_2$ was purchased from Energy Chemical, alkenes were synthesized by previous reports.[1] Besides, all substrates and reagents were commercially available and used without further purification. TLC analysis was performed using pre-coated glass plates. Column chromatography was performed using silica gel (200–300 mesh). ^{1}H spectra were recorded in $CDCl_3$, CD_2Cl_2 and DMSO-d_6 on 600/400 MHz NMR spectrometers and resonances (δ) are given in parts per million relative to tetramethylsilane. Data are reported as follows: chemical shift, multiplicity (s = singlet, d = doublet, t = triplet, m = multiplet), coupling constants (Hz) and integration. ^{13}C spectra were recorded in $CDCl_3$, CD_2Cl_2 and DMSO-d_6 on 150/100 MHz NMR spectrometers and resonances (δ) are given in ppm. HRMS were obtained on a Bruker 7-tesla FT-ICR MS equipped with an electrospray source. The X-ray crystal-structure determinations were obtained on a Bruker SMART APEX CCD system.

2. Supplementary Methods

2.1 General procedure for synthesis of 4-54 (4 as an example)

A 25Ml Schlenk-type tube (with a Teflon screw cap and a side arm) equipped with a magnetic stir bar was charged with the mixture of alkene (0.1 mmol), iodobenzene (0.4 mmol), $Pd(OAc)_2$ (5 mol%), Na_2CO_3 (0.2mmol), H_2O (40μL) in DMF (2 mL). The reaction was frozen with the liquid nitrogen and then the tube was evacuated and backfilled with nitrogen (5 times). The mixture was first stirred at room temperature for 10 minutes and then stirred at 110°C for 12 hours. After cooling to room temperature, the mixture was quenched with water (25 mL), extracted with EtOAc (3 × 50 mL), the combined organic layers were washed with brine, dried over anhydrous Na_2SO_4 and concentrated under reduced pressure. The crude product was purified by column chromatography on silica gel (eluent: petroleum ether/EtOAc) to afford the products **4**.

2.2 General procedure for synthesis of 55-102 (69 as an example)

A 25Ml Schlenk-type tube (with a Teflon screw cap and a side arm) equipped with a magnetic stir bar was charged with the mixture of alkene (0.1 mmol), iodobenzene (0.15 mmol), TMS-TMS (0.2mmol), $Pd(OAc)_2$ (5 mol%), K_2CO_3 (0.15mmol), Me_4NOAc (0.1mmol) in DMF (2 mL). The reaction was frozen with the liquid nitrogen and then the tube was evacuated and backfilled with nitrogen (5 times). The mixture was first stirred at room temperature for 10 minutes and then stirred at 70°C for 12 hours. After cooling to room temperature, the mixture was quenched with water (25 mL), extracted with EtOAc (3 × 50 mL), the combined organic layers were washed with brine, dried over anhydrous Na_2SO_4 and concentrated under reduced pressure. The crude product was purified by column chromatography on silica gel (eluent: petroleum ether/EtOAc) to afford the products **69**.

2.3 General procedure for synthesis of 103-135 (103 as an example)

A 25Ml Schlenk-type tube (with a Teflon screw cap and a side arm) equipped with a magnetic stir bar was charged with the mixture of alkene (0.1 mmol), iodobenzene (0.15 mmol), B_2Pin_2 (0.2mmol), $Pd(OAc)_2$ (5 mol%), PivOK (0.2mmol) in DMF (2 mL). The reaction was frozen with the liquid nitrogen and then the tube was evacuated and backfilled with nitrogen (5 times). The mixture was first stirred at room temperature for 10 minutes and then stirred at 100°C for 12 hours. After cooling to room temperature, the mixture was quenched with water (25 mL), extracted with EtOAc (3 × 50 mL), the combined organic layers were washed with brine, dried over anhydrous Na_2SO_4 and concentrated under reduced pressure. The crude product was purified by column chromatography on silica gel (eluent: petroleum ether/EtOAc) to afford the products **103**.

3. Supplementary Tables

3.1 Optimization of the Reaction Conditions for synthesis of 4

We first tested several bases and palladium sources; Na_2CO_3 and $Pd(OAc)_2$ were optimal for formation of **4** (Supplementary Table 2). Compound **3** probably forms via a migration insertion, β-H elimination, reinsertion, and nucleophilic cyclization sequence. Reduced temperature promotes formation of **3** whereas higher temperature favors **4** (Supplementary Table 1, entries 1-6). Distinct from the typical domino-type C-H activation reaction, we observed no monoarylation, diarylation, or simple β-H elimination product in this reaction. Next, we investigated the dosage of PhI. Reducing the amounts of PhI to less than 3eq, or increasing it to greater than 4eq, sharply decreased the yield of **4**. Therefore, we chose 4eq of PhI as the best dosage, although some homo-coupling product of PhI was observed (Supplementary Table 1, entries 7-11). Not surprisingly, only dipolar solvents were compatible with this reaction (Supplementary Table 1, entries 12-18). However, although DMSO functioned well with model substrates, it was suboptimal in additional tests of substrate scope. Thus, we selected DMF as the optimal solvent.

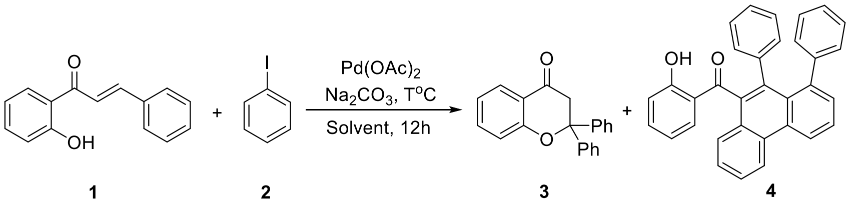

Entry	Temp.(°C)	Equiv. (PhI)	Solvent	**3** [%][a]	**4** [%][a]
1	25	4	DMF	56	15
2	50	4	DMF	52	18
3	70	4	DMF	42	44
4	90	4	DMF	16	73
5	110	4	DMF	<5	85
6	130	4	DMF	<5	81
7	110	1.2	DMF	32	30

8	110	**2**	DMF	26	51
9	110	3	DMF	14	75
10	110	5	DMF	<5	71
11	110	8	DMF	-	trace
12	110	4	DMSO	7	86
13	110	4	DMA	21	74
14	110	4	DMF/H_2O	<5	88
15	110	4	THF	-	-
16	110	4	DCE	-	45
17	110	4	EtOH	-	-
18	110	4	MeCN	-	-
19[b]	110	4	DMF/H_2O	15	79
20[c]	110	4	DMF/H_2O	29	64

Supplementary Table 1. Screening the solvent and temperature for synthesis of 4.

Reaction conditions: **1** (0.1 mmol), **2** (x mmol), $Pd(OAc)_2$ (5 mol%), Na_2CO_3 (0.2 mmol), solvent (2 ml), H_2O (40 μl), T °C, 12 h. [a]Yields of isolated products based on **1**. [b]Reaction performed in 2 mmol scale. [c]air atmosphere.

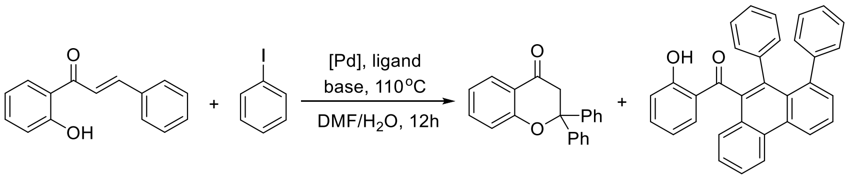

Entry	Base (2eq)	[Pd] (5 mol%)	ligand	**3** [%][a]	**4** [%][a]
1	-	$Pd(OAc)_2$	-	21	34
2	Li_2CO_3	$Pd(OAc)_2$	-	52	35
3	K_2CO_3	$Pd(OAc)_2$	-	18	68
4	Cs_2CO_3	$Pd(OAc)_2$	-	10	73
5	K_3PO_4	$Pd(OAc)_2$	-	26	45
6	NaOAc	$Pd(OAc)_2$	-	46	trace
7	NaOH	$Pd(OAc)_2$	-	33	48
8	t-BuOK	$Pd(OAc)_2$	-	23	56
9	CsF	$Pd(OAc)_2$	-	55	trace
10	Et_3N	$Pd(OAc)_2$	-	41	n.d.
11	DBU	$Pd(OAc)_2$	-	trace	trace
12	DABCO	$Pd(OAc)_2$	-	27	n.d.
13	Na_2CO_3	$Pd(TFA)_2$	-	21	66
14	Na_2CO_3	$Pd(acac)_2$	-	trace	87
15	Na_2CO_3	K_2PdCl_6	-	trace	88
16	Na_2CO_3	$PdCl_2$	-	15	70
17	Na_2CO_3	$Pd_2(dba)_3$	-	24	57
18	Na_2CO_3	$Pd(OAc)_2$	1,10-Phen	n.d.	n.d.

19	Na_2CO_3	$Pd(OAc)_2$	2,2'-Biquinoline	n.d.	Trace
20	Na_2CO_3	$Pd(OAc)_2$	PPh_3	trace	54
21	Na_2CO_3	$Pd(OAc)_2$	$P(o\text{-}tol)_3$	n.d.	20
22	Na_2CO_3	$Pd(OAc)_2$	dppp	n.d.	56
23	Na_2CO_3	$Pd(OAc)_2$	dppf	n.d.	16
24	Na_2CO_3	$Pd(OAc)_2$	DPEPhos	trace	85
25	Na_2CO_3	$Pd(OAc)_2$	t-BuXPhos	trace	83
26	Na_2CO_3	$Pd(OAc)_2$	XantPhos	trace	79

Supplementary Table 2. Screening the bases and palladium sources for synthesis of 4. Reaction conditions: **1** (0.1 mmol), **2** (0.4 mmol), [Pd] (5 mol%), ligand (22 mol%), base (0.2 mmol), DMF (2 ml), H_2O (40 μl), 110 °C, 12 h. [a]Yields of isolated products based on **1**.

3.2 Optimization of the Reaction Conditions for synthesis of 69

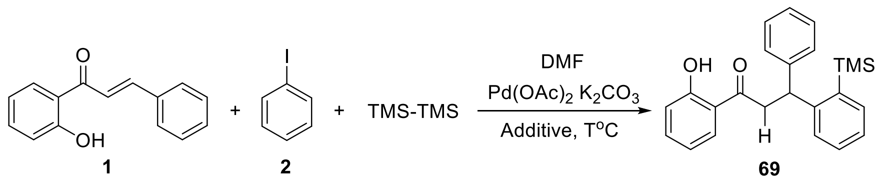

Entry	K_2CO_3 (x eq)	Temp. (°C)	Additives (1 eq)	**69** [%][a]	**1** [%][a]
1	-	110	-	16	75
2	0.5	110	-	38	51
3	1	110	-	58	23
4	1.5	110	-	78	16
5	2	110	-	77	15
6	3	110	-	75	18
7	4	110	-	73	16
8	1.5	r.t.	-	47	42
9	1.5	50	-	61	35
10	1.5	70	-	81	15
11	1.5	90	-	80	14
12	1.5	130	-	75	16
13	1.5	70	Bu_4NBr	82	13
14	1.5	70	Bu_4NOAc	83	14
15	1.5	70	Me_4NOAc	92	-

Supplementary Table 3. Optimization of the Reaction Conditions for synthesis of 69. Reaction conditions: **1** (0.1 mmol), **2** (0.15 mmol), hexamethyldisilane (0.2 mmol), additives (0.1 mmol), $Pd(OAc)_2$ (5 mol%), K_2CO_3 (x mmol), DMF (2 mL), T °C, 12 h. [a]Products were obtained in isolated yields based on **1.**

3.3 Optimization of the Reaction Conditions for synthesis of 103

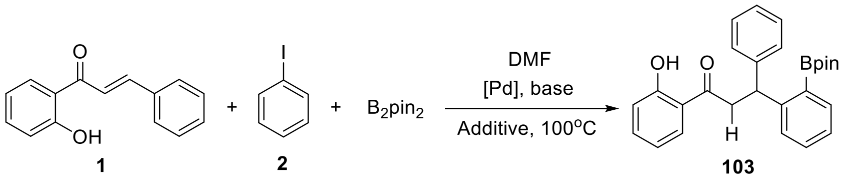

Entry	base (2 eq)	[Pd]	Additives (1 eq)	Equiv. of B_2pin_2	**103** [%][a]
1	$LiCO_3$	$Pd(OAc)_2$	-	2	Complex
2	Na_2CO_3	$Pd(OAc)_2$	-	2	41
3	K_2CO_3	$Pd(OAc)_2$	-	2	77
4	$CsCO_3$	$Pd(OAc)_2$	-	2	59
5	K_3PO_4	$Pd(OAc)_2$	-	2	52
6	KOAc	$Pd(OAc)_2$	-	2	75
7	tBuOK	$Pd(OAc)_2$	-	2	70
8	PivOK	$Pd(OAc)_2$	-	2	84
9	DABCO	$Pd(OAc)_2$	-	2	-
10	Et_3N	$Pd(OAc)_2$	-	2	-
11	PivOK	$Pd(PPh_3)_4$	-	2	-
12	PivOK	$Pd_2(dba)_3$	-	2	49
13	PivOK	$Pd(tBu_3P)_2$	-	2	-
14	PivOK	$PdCl_2$	-	2	-
15	PivOK	$Pd(OAc)_2$	Bu_4NBr	2	78
16	PivOK	$Pd(OAc)_2$	Bu_4NF	2	42
17	PivOK	$Pd(OAc)_2$	Bu_4NI	2	38
18	PivOK	$Pd(OAc)_2$	Bu_4NOAc	2	75
19	PivOK	$Pd(OAc)_2$	Me_4NOAc	2	76
20	PivOK	$Pd(OAc)_2$	-	1	52
21	PivOK	$Pd(OAc)_2$	-	1.5	64
22	PivOK	$Pd(OAc)_2$	-	2.5	84
23	PivOK	$Pd(OAc)_2$	-	3	82
24	PivOK	$Pd(OAc)_2$	-	4	73
25	PivOK	$Pd(OAc)_2$	-	5	54

Supplementary Table 4. Optimization of the Reaction Conditions for synthesis of 103. Reaction conditions: **1** (0.1 mmol), **2** (0.15 mmol), bis(pinacolato)diboron (x mmol), [Pd] (5 mol%), base (0.2 mmol), additives (0.1 mmol), DMF (2 ml), 100 °C, 12 h. [a]Yields of isolated products based on **1**.

4. Supplementary Figures

4.1 Monitoring of hydrogen gas

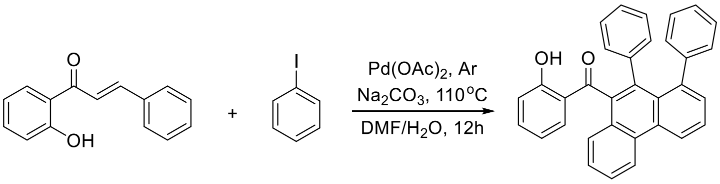

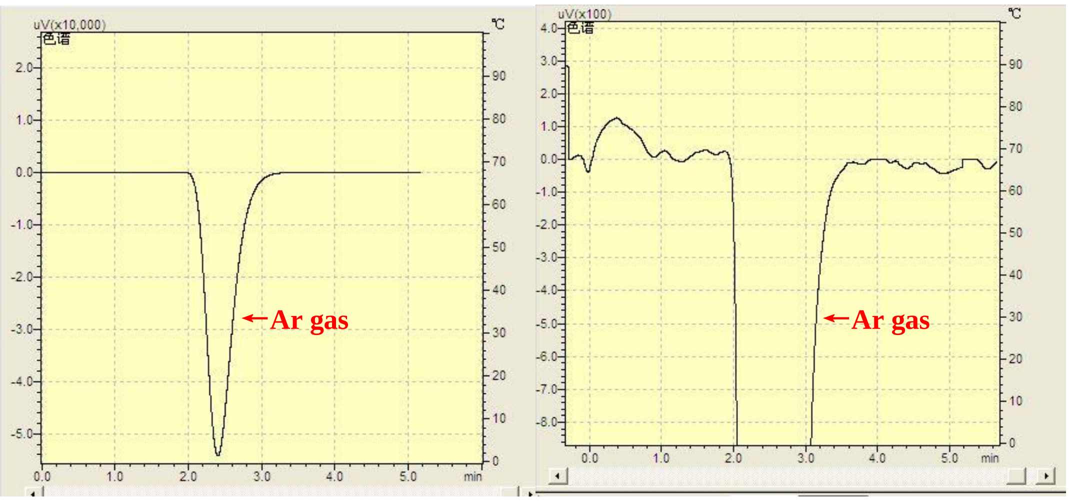

Supplementary Figure 1. Further control experiments. Monitoring of hydrogen gas in the formation of **4**.

4.2 Detection for the generation of PhH from 142 to 4.

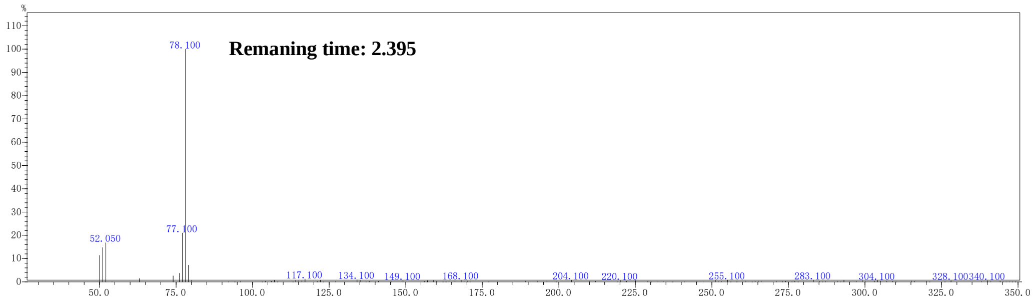

Supplementary Figure 2. Further control experiments. Detection for the generation of PhH from **142** to **4**.

Note: The detection of benzene that possibly to be a reduction product of PhI. Nevertheless, the amount of benzene that detected by GC-MS is less than the amount of corresponding oxidation product, the possibility for other alternative oxidative pathways cannot be excluded.

4.3 Isotopic labeling experiments

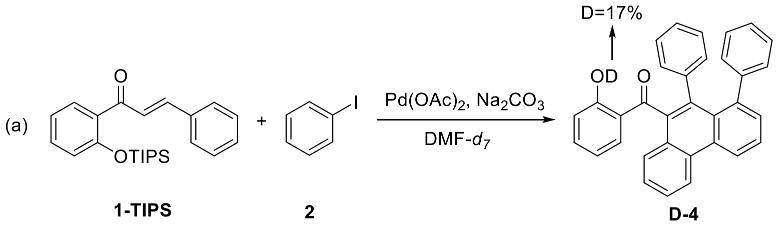

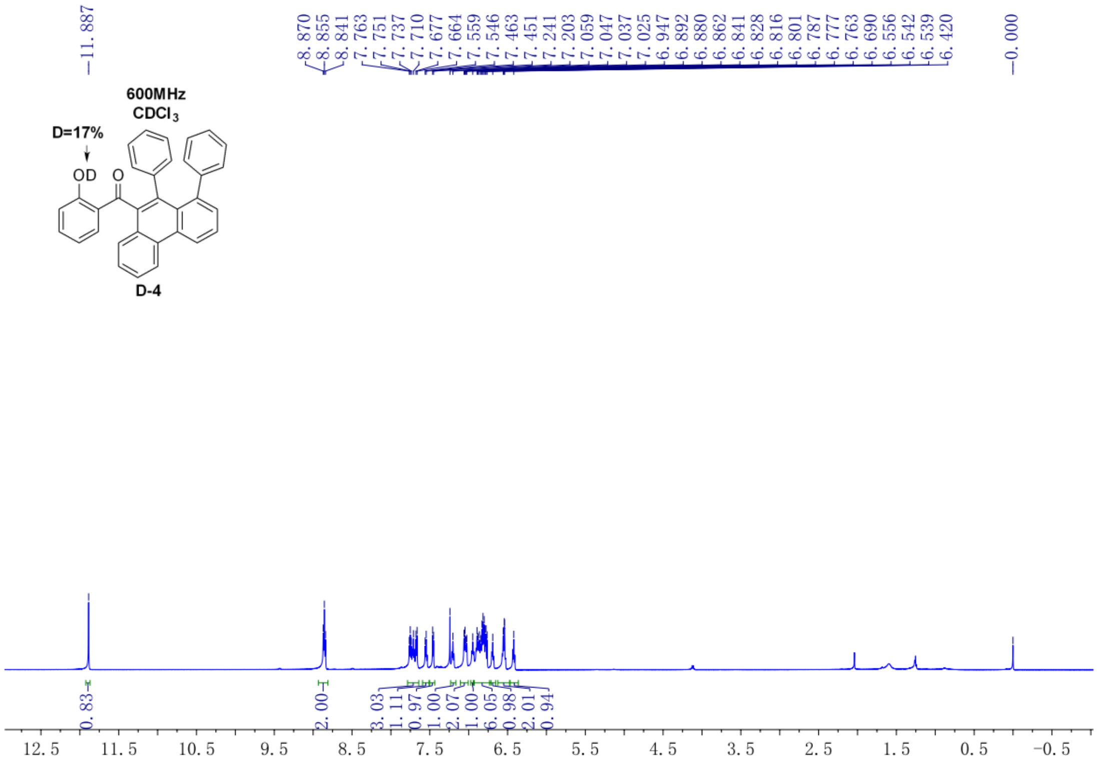

Supplementary Figure 3. Isotopic labeling experiments. Investigation on the source of hydrogen at hydroxyl group using DMF-d_7.

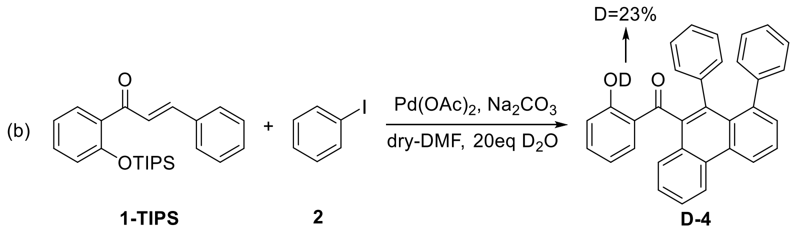

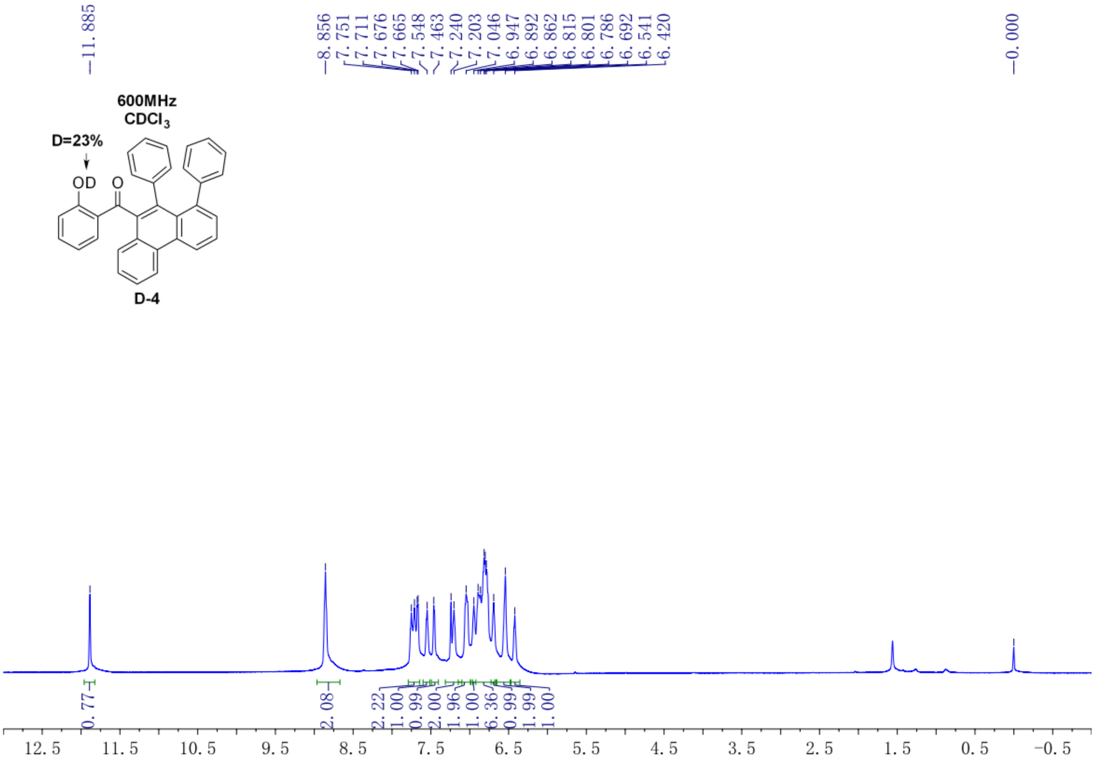

Supplementary Figure 4. Isotopic labeling experiments. Investigation on the source of hydrogen at hydroxyl group using D_2O.

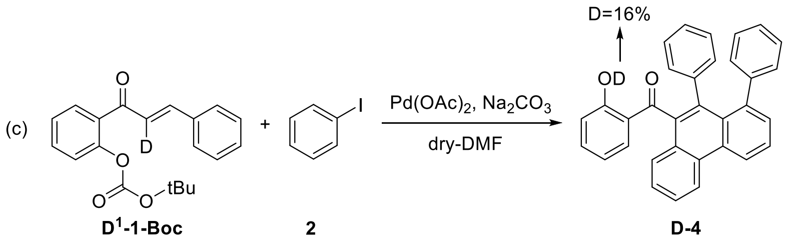
(c)
Pd(OAc)2, Na2CO3
dry-DMF
D=16%
D1-1-Boc
2
D-4

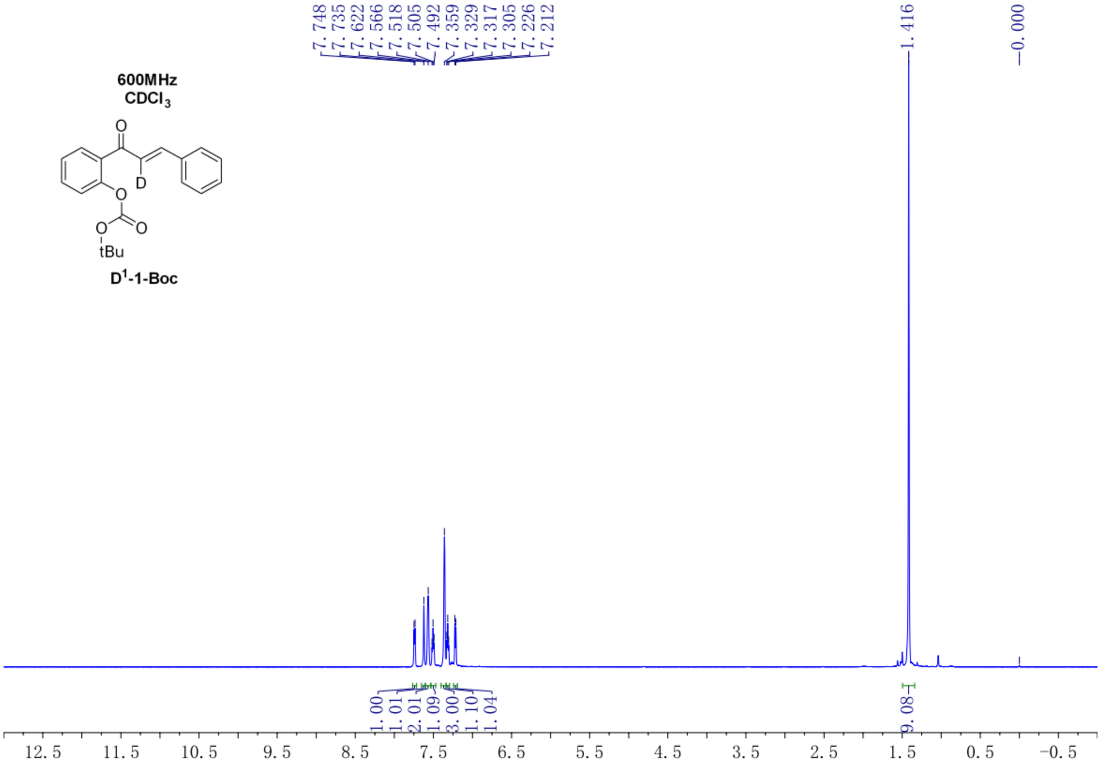
600MHz
CDCl3
D1-1-Boc
7.748
7.735
7.622
7.566
7.518
7.505
7.492
7.359
7.329
7.317
7.305
7.226
7.212
1.416
-0.000
1.00
1.01
2.01
1.09
3.00
1.10
1.04
9.08
12.5
11.5
10.5
9.5
8.5
7.5
6.5
5.5
4.5
3.5
2.5
1.5
0.5
-0.5

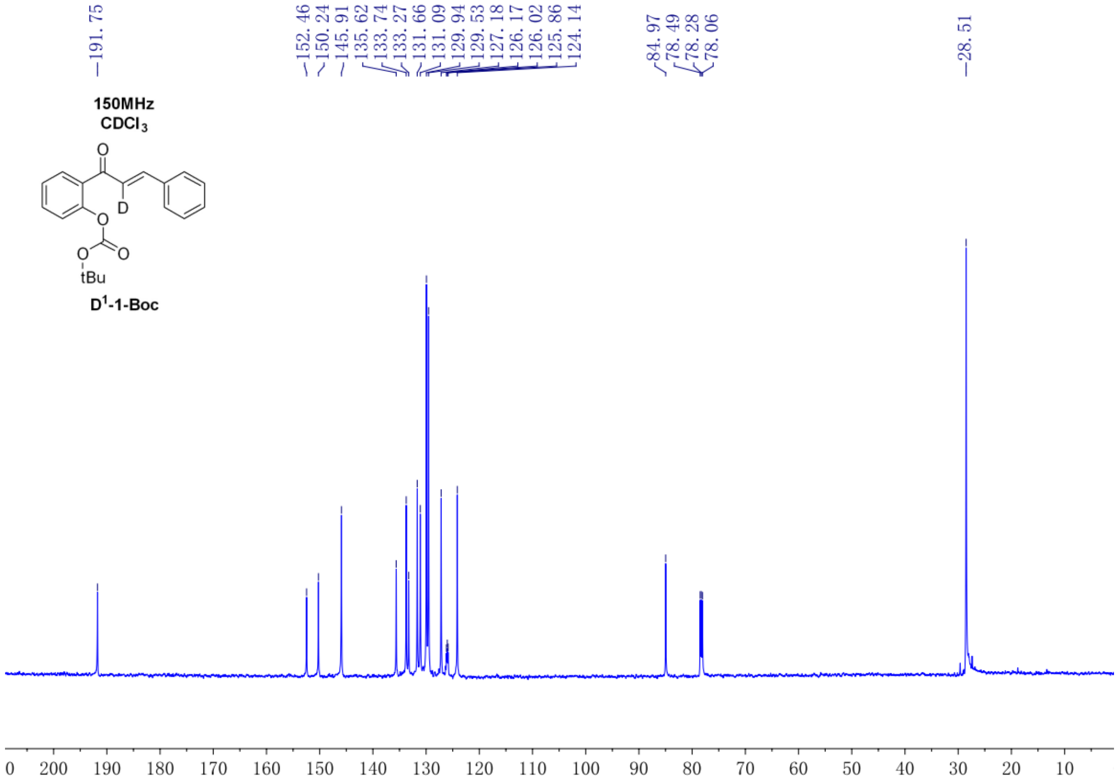
150MHz
CDCl3
D1-1-Boc
191.75
152.46
150.24
145.91
135.62
133.74
133.27
131.66
131.09
129.94
129.53
127.18
126.17
126.02
125.86
124.14
84.97
78.49
78.28
78.06
28.51
0
200
190
180
170
160
150
140
130
120
110
100
90
80
70
60
50
40
30
20
10

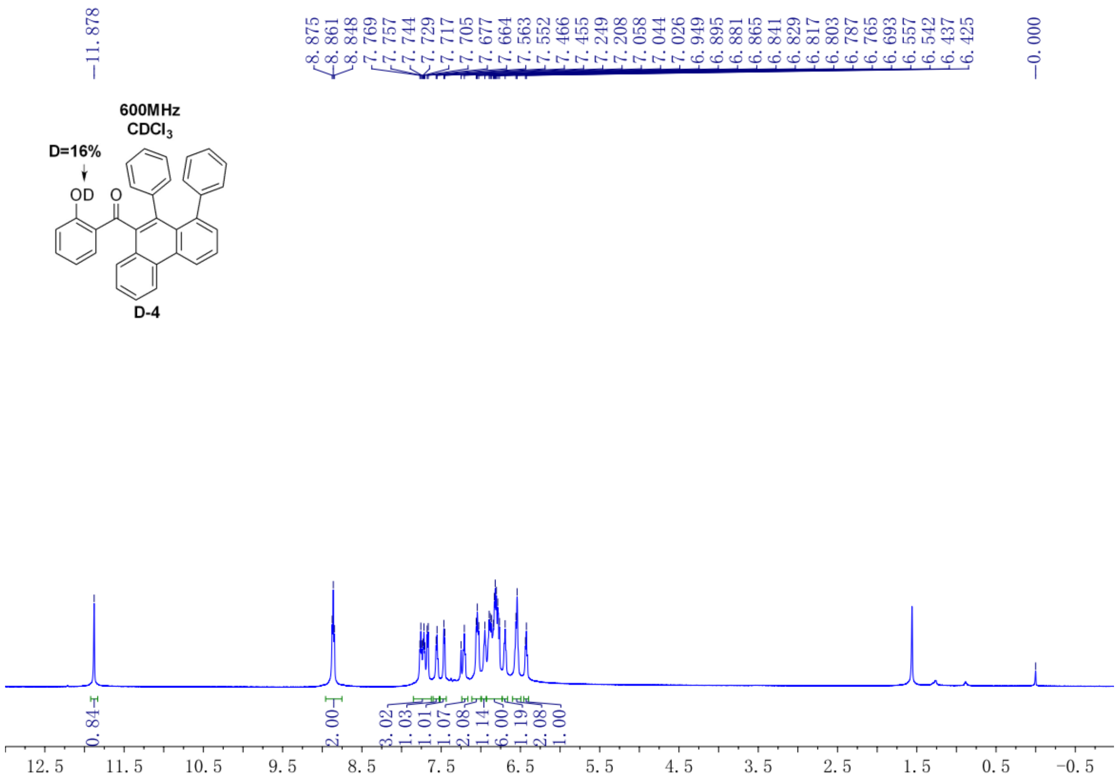

Supplementary Figure 5. Isotopic labeling experiments. Investigation on the source of hydrogen at hydroxyl group using [α-D]-2’-OBoc-Chalcone.

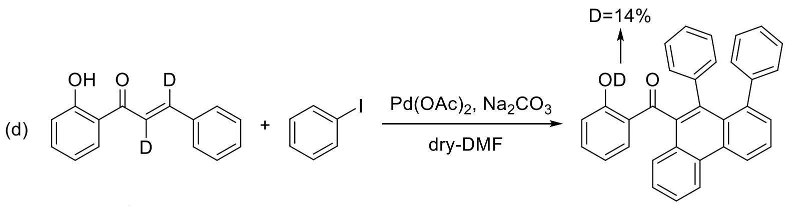
(d)
OH
O
D
D
+
I
Pd(OAc)2, Na2CO3
dry-DMF
D=14%
OD
O

D²-1 **2** **D-4**

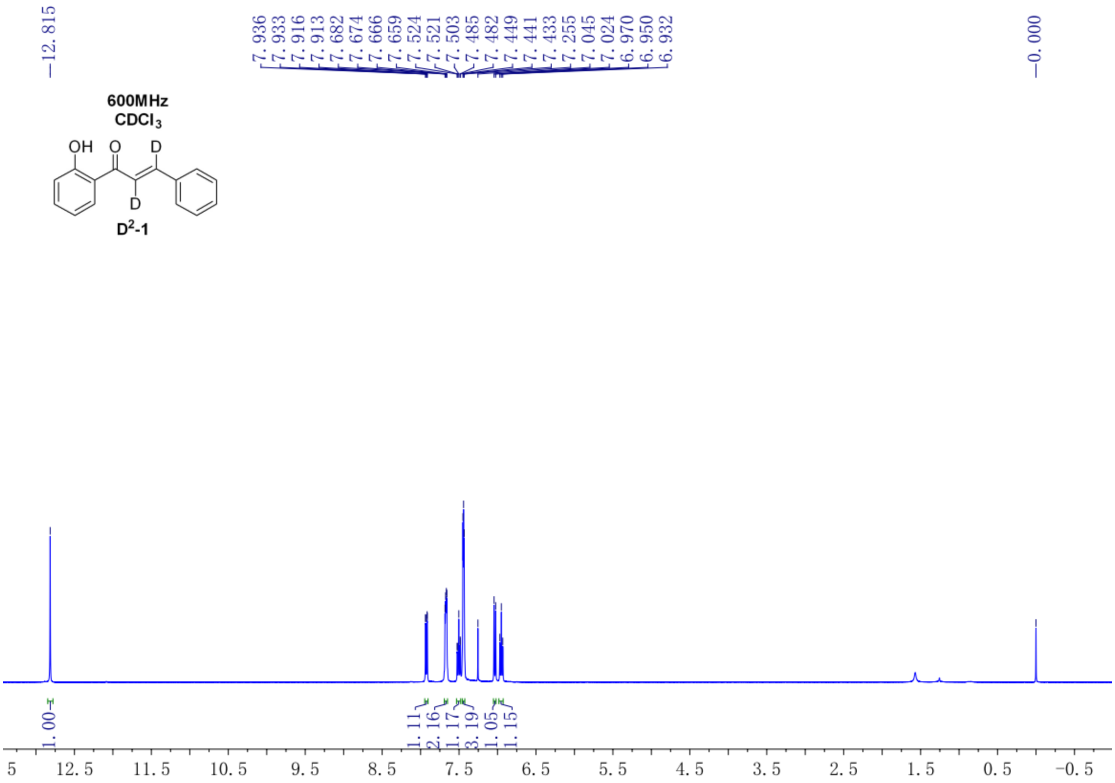
12.815
7.936
7.933
7.916
7.913
7.682
7.674
7.666
7.659
7.524
7.521
7.503
7.485
7.482
7.449
7.441
7.433
7.255
7.045
7.024
6.970
6.950
6.932
0.000
600MHz
CDCl3
OH
O
D
D
D2-1
1.00
1.11
2.16
1.17
3.19
1.05
1.15
5
12.5
11.5
10.5
9.5
8.5
7.5
6.5
5.5
4.5
3.5
2.5
1.5
0.5
-0.5

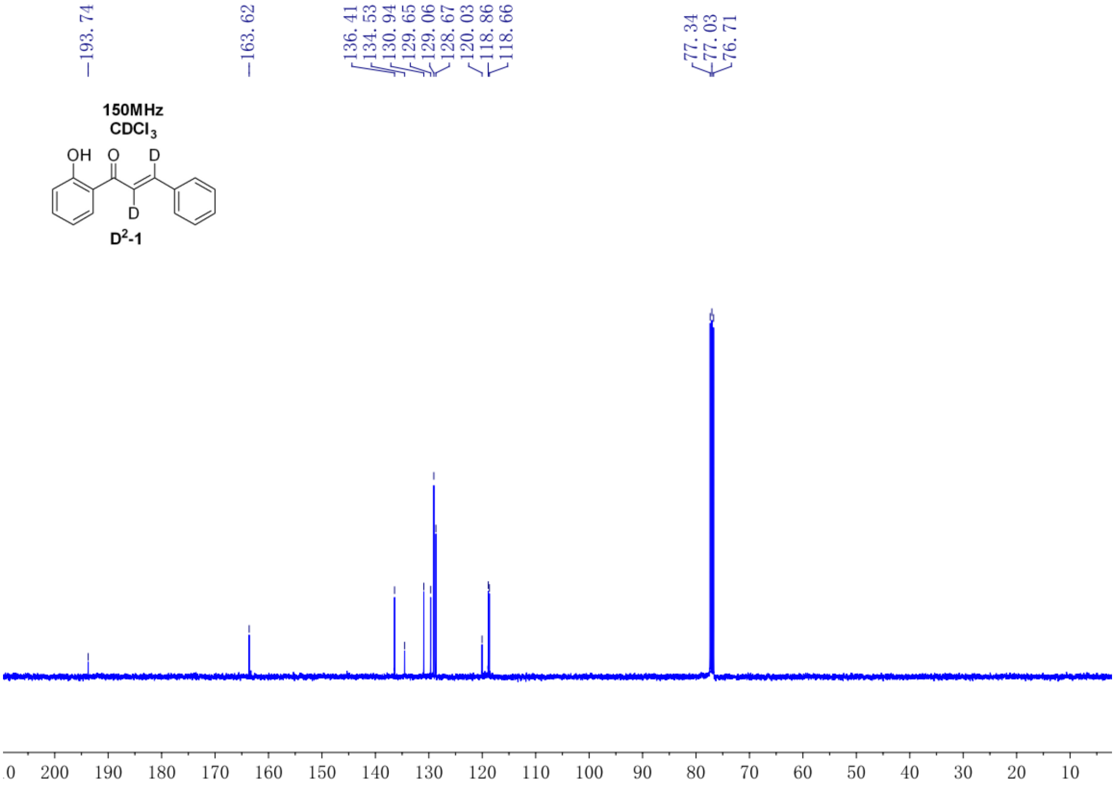
193.74
163.62
136.41
134.53
130.94
129.65
129.06
128.67
120.03
118.86
118.66
77.34
77.03
76.71
150MHz
CDCl3
OH
O
D
D
D2-1
0
200
190
180
170
160
150
140
130
120
110
100
90
80
70
60
50
40
30
20
10

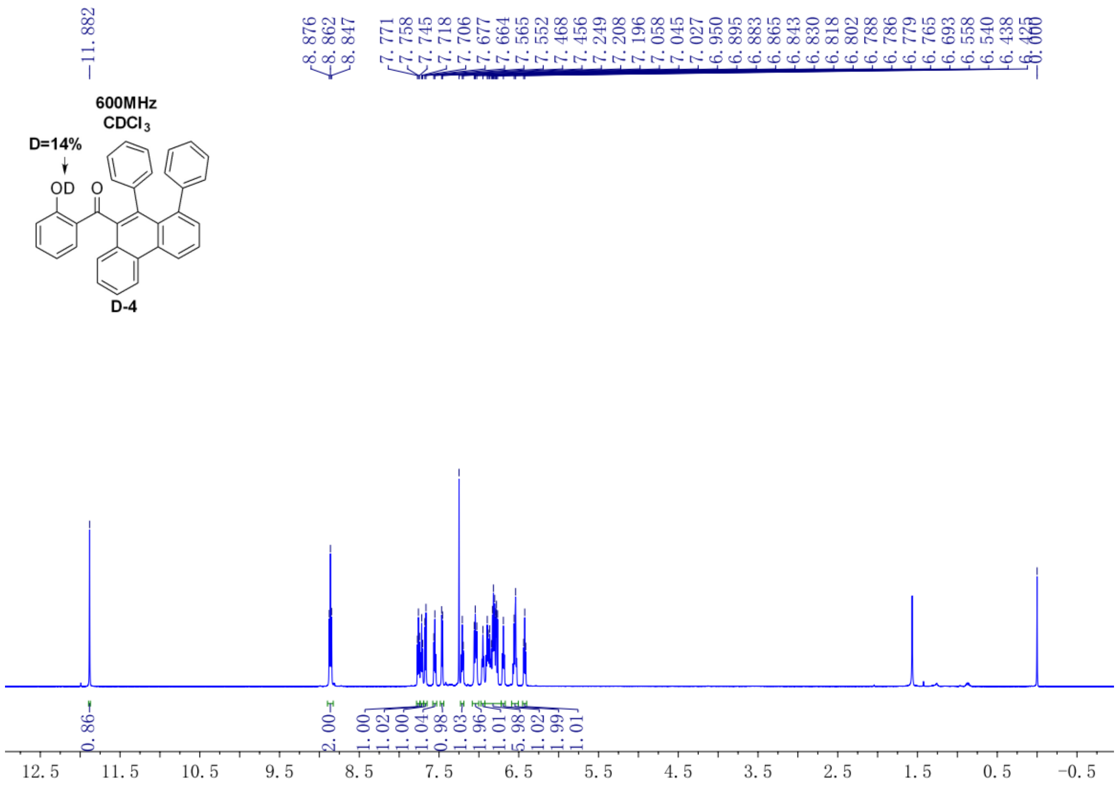

Supplementary Figure 6. Isotopic labeling experiments. Investigation on the source of hydrogen at hydroxyl group using [α-β-D_2]-2’-OH-Chalcone.

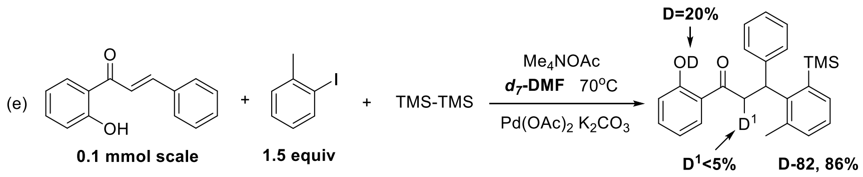

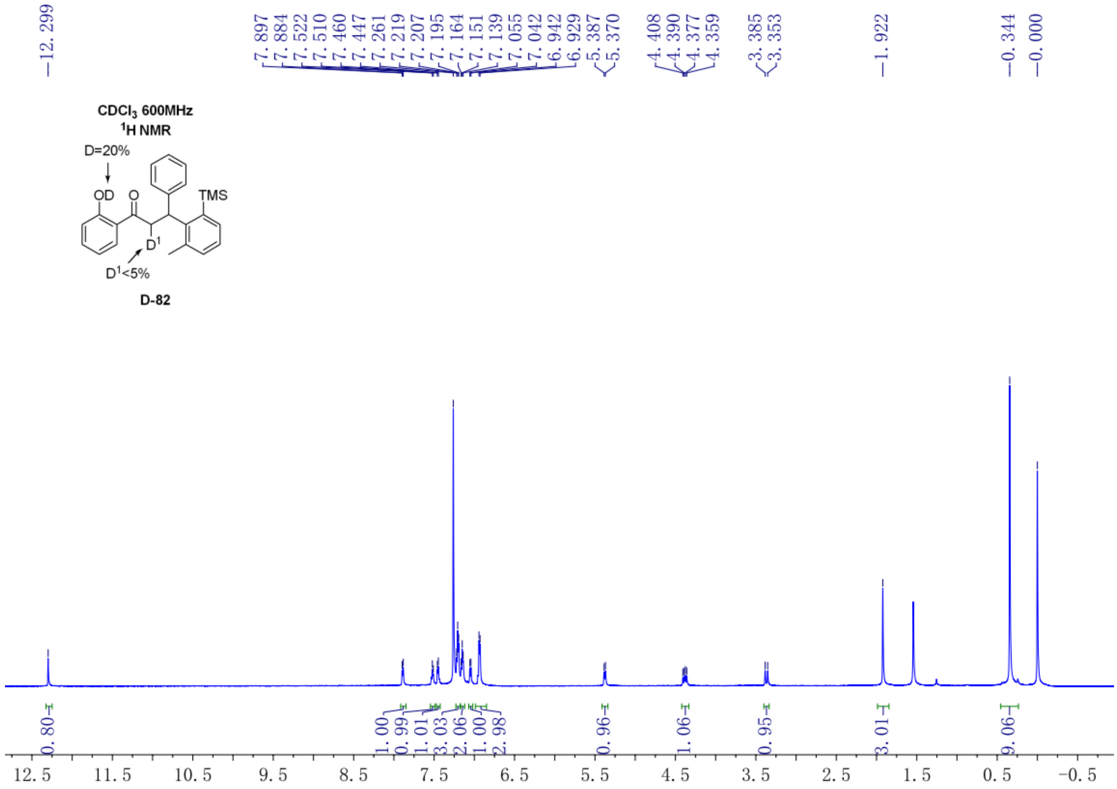

Supplementary Figure 7. Isotopic labeling experiments. Investigation on the source of D and D[1] using DMF-d_7.

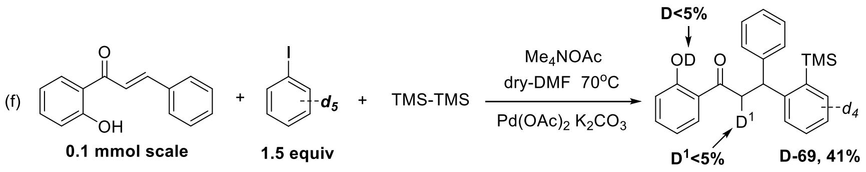

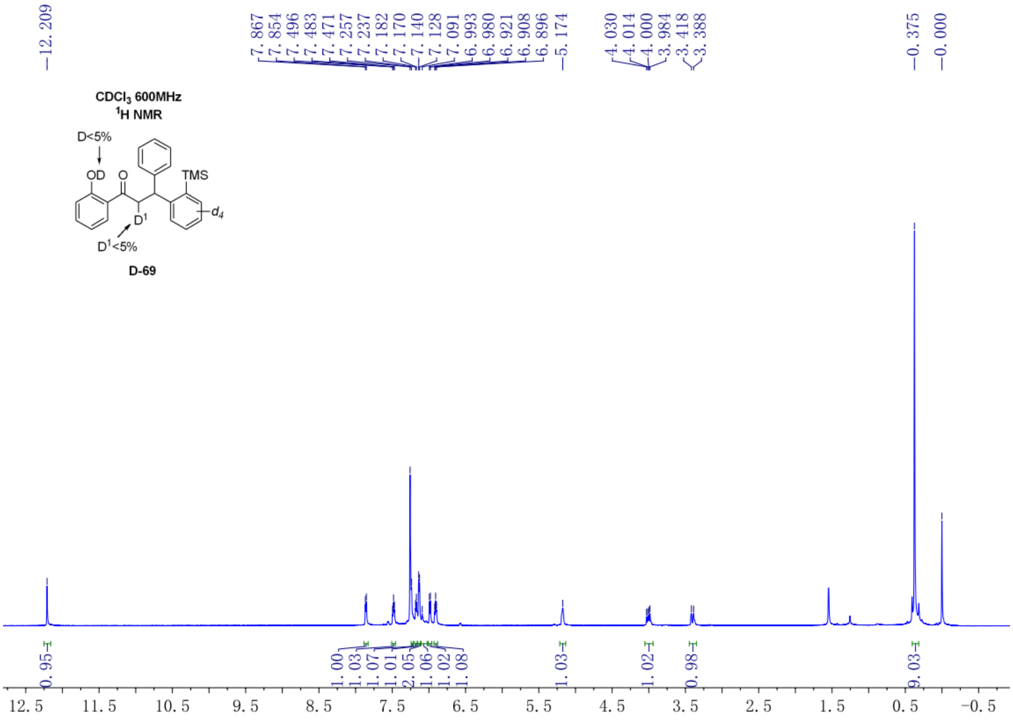

Supplementary Figure 8. Isotopic labeling experiments. Investigation on the source of D and D^1 using PhI-d_5.

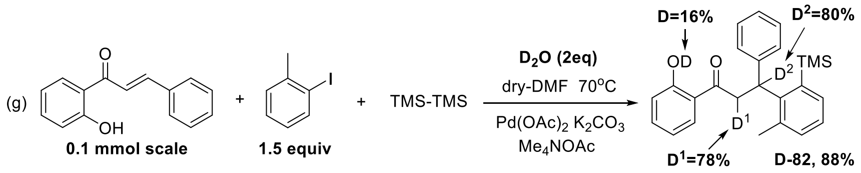

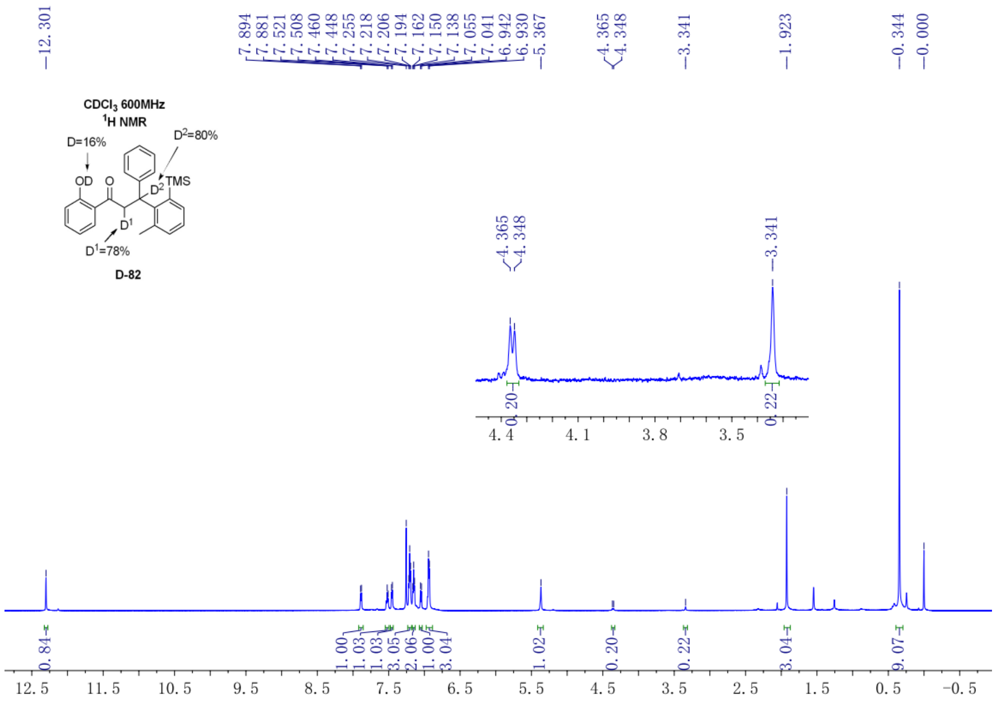

Supplementary Figure 9. Isotopic labeling experiments. Investigation on the source of D and D[1] using D_2O.

4.4 Necessity of hydroxyl group for silylation and borylation

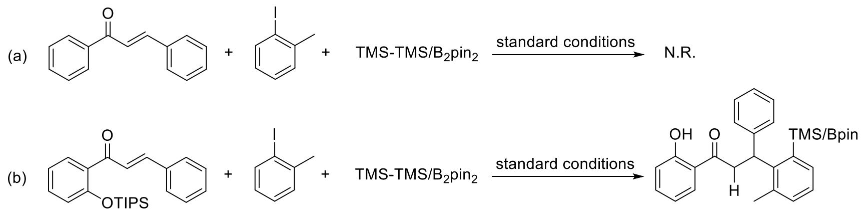

Supplementary Figure 10. Further control experiments. Necessity of hydroxyl group for silylation and borylation.

4.5 Proposed mechanism

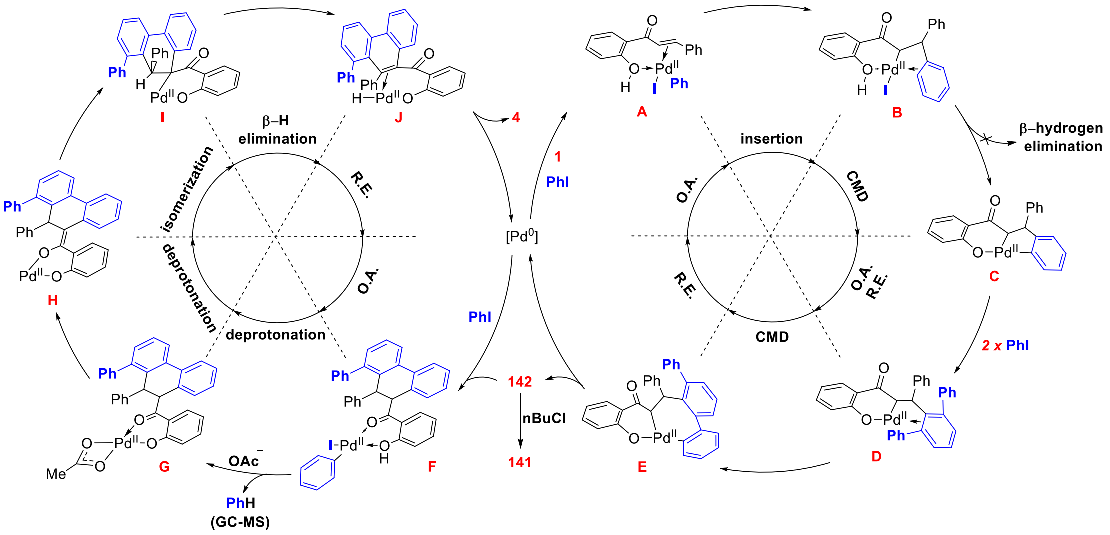

Supplementary Figure 11. Mechanism proposal. Possible mechanism of quadruple C-H activation cascade (**4** as example).

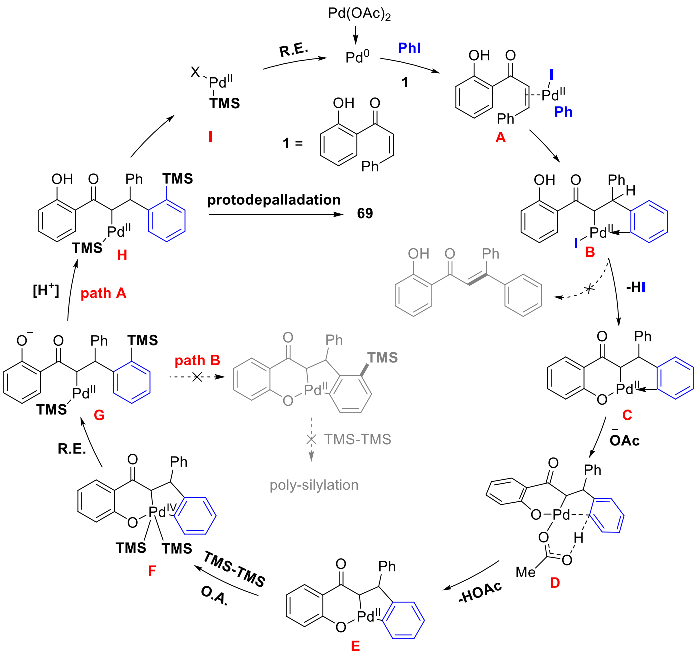

Supplementary Figure 12. Mechanism proposal. Possible mechanism for silylation (**69** as example).

4.6 Characterization data for compounds[a]

[a]**note**: For compounds **4-54**, asymmetrical substitutions on benzaldehyde moiety (the R^2 groups) restrain the free rotation of the C^9-C^{10} bond due to the steric effects, resulting in unpredictable and unequal peak split of ^{13}C and ^{1}H NMR spectra for all the corresponding compounds (**20-25**, **33-41**, **44**, **46**, **131**). The disorder of single crystal structure of **44** between bromide and methyl group is consistent with this explanation.

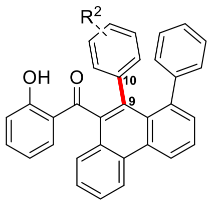

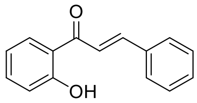

2’-Hydroxychalcone (1):

yellow solid; TLC (PET:EtOAc, 100:1 v/v): R_f = 0.3; ^{1}H NMR (600 MHz, $CDCl_3$) δ=12.89 (s, 1H), 7.91–7.73 (m, 2H), 7.63–7.46 (m, 3H), 7.41 (t, *J* = 7.8 Hz, 1H), 7.35 (s, 3H), 6.98 (d, *J* = 8.4 Hz, 1H), 6.87 (t, *J* = 7.8 Hz, 1H). ^{13}C NMR (150 MHz, $CDCl_3$) δ=194.7, 164.7, 146.5, 137.4, 135.5, 132.0, 130.8, 130.1, 129.7, 121.0, 121.1, 119.9, 119.6.

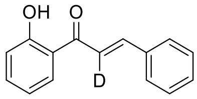

[α-D]-2’-Hydroxychalcone (D^1-1):

yellow solid; TLC (PET:EtOAc, 100:1 v/v): R_f = 0.3; ^{1}H NMR (600 MHz, $CDCl_3$) δ=12.85 (s, 1H), 7.89 (d, *J* = 6.0 Hz, 2H), 7.63 (s, 2H), 7.51–7.45 (m, 1H), 7.41 (s, 3H), 7.01 (d, *J* = 8.4 Hz, 1H), 6.93 (d, *J* = 7.2 Hz, 1H). ^{13}C NMR (150 MHz, $CDCl_3$) δ=194.7, 164.6, 146.4, 137.4, 135.6, 132.0, 130.7, 130.1, 129.7, 121.0, 119.9, 119.6.

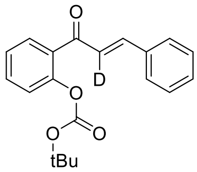

[α-D]-2’-OBoc-chalcone (D^1-1-Boc):

yellow solid; TLC (PET:EtOAc, 50:1 v/v): R_f = 0.3; ^{1}H NMR (600 MHz, $CDCl_3$) δ=7.74 (d, *J* = 7.8 Hz, 1H), 7.62 (s, 1H), 7.57 (s, 2H), 7.51 (t, *J* = 7.8 Hz, 1H), 7.36 (s, 3H), 7.32 (t, *J* = 7.2 Hz, 1H), 7.22 (d, *J* = 7.8 Hz, 1H), 1.42 (s, 9H). ^{13}C NMR (150 MHz, $CDCl_3$) δ=191.8, 152.5, 150.2, 145.9, 135.6, 133.7, 133.3, 131.7, 131.1, 129.9, 129.5, 127.2, 126.2, 126.0, 125.9, 124.1, 85.0, 28.5. HRMS (ESI) m/z calcd for $C_{20}H_{19}DO_4Na+$ $(M+Na)^+$ 348.13166, found 348.13146.

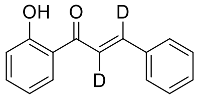

[α-D-β-D]-2’-Hydroxychalcone (D^2-1):

yellow solid; TLC (PET:EtOAc, 100:1 v/v): R_f = 0.3; ^{1}H NMR (400 MHz, $CDCl_3$) δ=12.81 (s, 1H), 7.92 (dd, *J* = 8.0, 1.2 Hz, 1H), 7.67 (dd, *J* = 6.4, 2.8 Hz, 2H), 7.53–7.48 (m, 1H), 7.46–7.43 (m, 3H), 7.03 (d, *J* = 8.4 Hz, 1H), 6.95 (t, *J* = 7.6 Hz,

1H). ^{13}C NMR (100 MHz, $CDCl_3$) δ=193.7, 163.6, 136.4, 134.5, 130.9, 129.7, 129.1, 128.7, 120.0, 118.9, 118.7. HRMS (ESI) m/z calcd for $C_{15}H_{11}D_2O_2+$ $(M+H)^+$ 227.10356, found 227.10363.

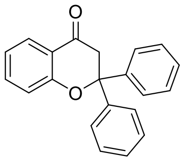

2,2-diphenylchroman-4-one (3):

Yield 56%; 16.8 mg; white solid; mp 137–140°C; TLC (PET:EtOAc, 50:1 v/v): R_f = 0.3; ^1H NMR (600 MHz, $CDCl_3$) δ=7.74 (d, *J* = 7.8 Hz, 1H), 7.40 (t, *J* = 8.4 Hz, 4H), 7.39–7.35 (m, 1H), 7.24 (t, *J* = 7.8 Hz, 4H), 7.17 (t, *J* = 7.2 Hz, 2H), 7.09 (d, *J* = 8.4 Hz, 1H), 6.84 (t, *J* = 7.2 Hz, 1H), 3.48 (s, 2H). ^{13}C NMR (150 MHz, $CDCl_3$) δ=191.1, 159.6, 142.7, 136.1, 128.4, 127.8, 126.5, 126.2, 121.3, 121.3, 118.5, 85.8, 48.5. HRMS (ESI) m/z calcd for $C_{21}H_{17}O_2+$ $(M+H)^+$ 301.12231, found 301.12237.

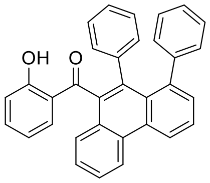

(1,10-diphenylphenanthren-9-yl)(2-hydroxyphenyl)methanone (4):

Yield 88%; 39.7 mg; white solid; mp 236–239°C; TLC (PET:EtOAc, 100:1 v/v): R_f = 0.3; ^1H NMR (600 MHz, $CDCl_3$) δ=12.00 (s, 1H), 8.90 (t, *J* = 9.0 Hz, 2H), 7.84–7.72 (m, 3H), 7.59 (t, *J* = 7.2 Hz, 1H), 7.51 (d, *J* = 7.2 Hz, 1H), 7.25 (t, *J* = 7.2 Hz, 1H), 7.12 (dd, *J* = 22.2, 7.8 Hz, 2H), 7.04–7.00 (m, 1H), 6.99–6.81 (m, 6H), 6.76 (d, *J* = 6.6 Hz, 1H), 6.68–6.56 (m, 2H), 6.47 (d, *J* = 7.8 Hz, 1H). ^{13}C NMR (150 MHz, $CDCl_3$) δ=205.2, 162.1, 143.3, 142.8, 139.0, 136.4, 136.3, 135.9, 133.0, 132.4, 132.0, 130.9, 130.7, 129.8, 129.1, 129.0, 128.8, 128.2, 127.4, 126.9, 126.8, 126.7, 126.5, 125.8, 123.3, 122.0, 120.5, 118.5, 117.6. HRMS (ESI) m/z calcd for $C_{33}H_{23}O_2+$ $(M+H)^+$ 451.16926, found 451.16943.

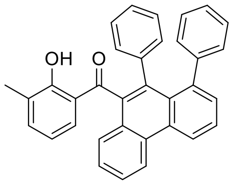

(1,10-diphenylphenanthren-9-yl)(2-hydroxy-3-methylphenyl)methanone (5):

Yield 84%; 39.2 mg; yellow solid; mp 214–218°C; TLC (PET:EtOAc, 100:1 v/v): R_f = 0.3; ^1H NMR (600 MHz, $CDCl_3$) δ=12.21 (s, 1H), 8.85 (t, *J* = 9.6 Hz, 2H), 7.75 (t, *J* = 7.8 Hz, 1H), 7.70 (t, *J* = 7.8 Hz, 1H), 7.65 (d, *J* = 8.4 Hz, 1H), 7.53 (t, *J* = 7.8 Hz, 1H), 7.45 (d, *J* = 7.2 Hz, 1H), 7.10–7.00 (m, 3H), 6.95 (t, *J* = 7.2 Hz, 1H), 6.86 (ddd, *J* = 21.0, 13.8, 6.0 Hz, 4H), 6.69 (dd, *J* = 19.2, 7.2 Hz, 2H), 6.56

(d, J = 6.0 Hz, 2H), 6.34 (t, J = 7.8Hz, 1H), 2.14 (s, 3H). ^{13}C NMR (150 MHz, $CDCl_3$) δ=205.3, 160.6, 143.3, 142.7, 139.0, 137.1, 136.2, 135.6, 132.3, 131.9, 130.7, 130.7, 130.6, 129.7, 129.0, 128.8, 128.3, 127.4, 127.3, 126.9, 126.7, 126.5, 126.3, 126.0, 125.7, 123.3, 122.0, 119.8, 117.8, 15.2. HRMS (ESI) m/z calcd for $C_{34}H_{24}O_2Na+$ $(M+Na)^+$ 487.16685, found 487.16692.

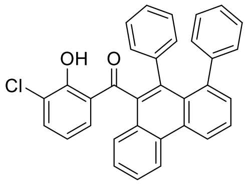

(3-chloro-2-hydroxyphenyl)(1,10-diphenylphenanthren-9-yl)methanone (6):
Yield 88%; 42.6 mg; white solid; mp 206–209°C; TLC (PET:EtOAc, 100:1 v/v): R_f = 0.3; ^{1}H NMR (600 MHz, $CDCl_3$) δ=12.57 (d, J = 2.4 Hz, 1H), 8.89 (t, J = 7.8 Hz, 2H), 7.77 (dt, J = 25.8, 7.8 Hz, 2H), 7.69 (d, J = 7.8 Hz, 1H), 7.59 (t, J = 7.2 Hz, 1H), 7.52 (d, J = 6.6 Hz, 1H), 7.32 (d, J = 7.8 Hz, 1H), 7.11 (dd, J = 12.6, 7.8 Hz, 2H), 7.02 (t, J = 7.2 Hz, 1H), 6.95 (t, J = 7.2 Hz, 1H), 6.93–6.87 (m, 2H), 6.84 (d, J = 7.2 Hz, 1H), 6.76 (dd, J = 5.4, 2.4 Hz, 2H), 6.66–6.57 (m, 2H), 6.40 (t, J = 7.8 Hz, 1H). ^{13}C NMR (150 MHz, $CDCl_3$) δ=204.8, 157.4, 142.8, 142.5, 138.5, 135.9, 135.7, 135.1, 132.1, 131.7, 131.2, 130.6, 130.3, 129.4, 128.6, 128.3, 127.7, 127.3, 127.2, 126.8, 126.6, 126.4, 125.6, 125.4, 123.1, 121.7, 121.5, 121.0, 118.3. HRMS (ESI) m/z calcd for $C_{33}H_{22}ClO_2+$ $(M+H)^+$ 485.13028, found 485.13019.

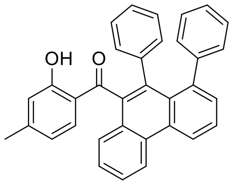

(1,10-diphenylphenanthren-9-yl)(2-hydroxy-4-methylphenyl)methanone (7):
Yield 87%; 40.4 mg; yellow solid; mp 192–195°C; TLC (PET:EtOAc, 100:1 v/v): R_f = 0.3; ^{1}H NMR (600 MHz, $CDCl_3$) δ=12.19 (s, 1H), 8.97–8.84 (m, 2H), 7.77 (ddd, J = 25.2, 18.6, 7.8 Hz, 3H), 7.59 (t, J =7.2 Hz, 1H), 7.54 (d, J = 7.2 Hz, 1H), 7.23 (d, J = 7.2 Hz, 1H), 7.15 (d, J = 7.2 Hz, 1H), 7.06 (t, J = 7.2 Hz, 1H), 7.03–6.89 (m, 4H), 6.81 (d, J = 7.8 Hz, 2H), 6.71 (d, J = 19.2 Hz, 3H), 6.30 (d, J = 7.8 Hz, 1H), 2.21 (s, 3H). ^{13}C NMR (150 MHz, $CDCl_3$) δ=204.2, 162.2, 147.9, 143.2, 142.6, 138.9, 136.0, 135.5, 132.8, 132.2, 131.8, 130.6, 130.4, 129.6, 128.8, 128.2, 127.4, 127.2, 126.8, 126.7, 126.5, 126.3, 125.8, 125.6, 123.2, 121.8, 119.7, 118.4, 117.6, 21.8. HRMS (ESI) m/z calcd for $C_{34}H_{25}O_2+$ $(M+H)^+$ 465.18491, found 465.18536.

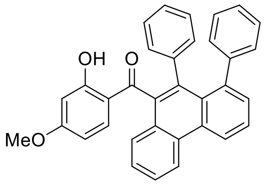

(1,10-diphenylphenanthren-9-yl)(2-hydroxy-4-methoxyphenyl)methanone (8):
Yield 80%; 38.4 mg; yellow solid; mp 177–180°C; TLC (PET:EtOAc, 50:1 v/v): R_f = 0.3; ^{1}H NMR (600 MHz, $CDCl_3$) δ=12.48 (s, 1H), 8.86–8.72 (m, 2H), 7.66

(ddd, J = 31.2, 16.2, 7.8 Hz, 3H), 7.49 (t, J = 7.2 Hz, 1H), 7.41 (d, J = 6.6 Hz, 1H), 7.09 (d, J = 7.2 Hz, 1H), 7.01 (s, 1H), 6.93 (s, 1H), 6.89–6.75 (m, 4H), 6.72–6.63 (m, 2H), 6.57 (dd, J = 16.2, 6.6 Hz, 2H), 6.21 (s, 1H), 5.93 (d, J = 9.0 Hz, 1H), 3.59 (s, 3H). ^{13}C NMR (150 MHz, $CDCl_3$) δ=202.7, 165.9, 165.1, 143.2, 142.7, 139.1, 136.0, 135.5, 134.6, 132.2, 131.8, 130.7, 130.5, 129.7, 129.2, 128.9, 128.3, 127.7, 127.4, 127.2, 126.8, 126.4, 125.9, 125.6, 123.2, 121.8, 114.8, 107.3, 100.0, 55.2. HRMS (ESI) m/z calcd for $C_{34}H_{25}O_3$+ $(M+H)^+$ 481.17982, found 481.18077.

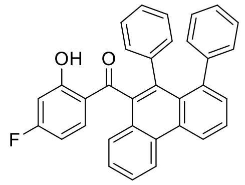

(1,10-diphenylphenanthren-9-yl)(4-fluoro-2-hydroxyphenyl)methanone (9):
Yield 86%; 40.3 mg; yellow solid; mp 195–199°C; TLC (PET:EtOAc, 100:1 v/v): R_f = 0.3; ^{1}H NMR (600 MHz, $CDCl_3$) δ=12.33 (s, 1H), 9.02–8.84 (m, 2H), 7.78 (ddd, J = 20.4, 13.2, 7.8 Hz, 3H), 7.67–7.59 (m, 1H), 7.53 (d, J = 6.6 Hz, 1H), 7.13 (dd, J = 17.4, 6.6 Hz, 2H), 7.03 (d, J = 7.2 Hz, 1H), 6.94 (dt, J = 13.8, 6.6 Hz, 3H), 6.82 (ddd, J = 26.4, 13.2, 7.2 Hz, 3H), 6.67 (t, J = 7.2 Hz, 1H), 6.60 (d, J = 7.2 Hz, 1H), 6.52 (d, J = 10.2 Hz, 1H), 6.17 (t, J = 7.2 Hz, 1H). (m, 4H), 7.60 (t, J = 7.4 Hz, 2H), 7.55 (d, J = 8.6 Hz, 2H), 2.16–2.10 (m, 2H), 1.84 (s, 3H), 1.15 (dt, J = 20.5, 7.2 Hz, 4H), 1.06 (dd, J = 14.7, 7.7 Hz, 2H), 0.96–0.91 (m, 2H), 0.79 (t, J = 7.2 Hz, 3H); ^{13}C NMR (150 MHz, $CDCl_3$) δ=204.0, 168.0, 166.3, 164.7, 164.6, 143.2, 142.9, 139.0, 135.9, 135.7, 135.5, 135.4, 132.5, 132.1, 132.0, 131.0, 130.6, 129.8, 129.0, 128.7, 128.1, 127.7, 127.5, 127.0, 126.9, 126.8, 126.7, 126.6, 125.9, 125.8, 123.4, 122.0, 117.7, 106.9, 106.8, 104.4, 104.2. ^{19}F NMR (375 MHz, $CDCl_3$) δ=98.56. HRMS (ESI) m/z calcd for $C_{33}H_{22}FO_2$+ $(M+H)^+$ 469.15984, found 469.15990.

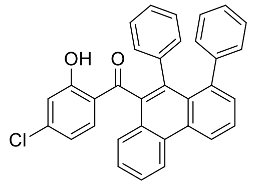

(4-chloro-2-hydroxyphenyl)(1,10-diphenylphenanthren-9-yl)methanone (10):
Yield 85%; 41.2 mg; white solid; mp 226–229°C; TLC (PET:EtOAc, 100:1 v/v): R_f = 0.3; ^{1}H NMR (600 MHz, $CDCl_3$) δ=12.10 (d, J = 13.2 Hz, 1H), 8.88 (t, J = 7.2 Hz, 2H), 7.92–7.64 (m, 3H), 7.64–7.47 (m, 2H), 7.11 (dd, J = 16.2, 7.2 Hz, 2H), 7.01 (t, J = 6.6 Hz, 1H), 6.95 (t, J = 6.6 Hz, 1H), 6.94–6.78 (m, 4H), 6.81–6.69 (m, 2H), 6.65 (t, J = 7.2 Hz, 1H), 6.58 (d, J = 7.2 Hz, 1H), 6.42 (d, J = 8.4 Hz, 1H). ^{13}C NMR (150 MHz, $CDCl_3$) δ=204.3, 162.6, 143.1, 142.8, 141.9, 138.8, 135.9, 135.5, 133.8, 132.4, 131.9, 130.8, 130.5, 129.7, 128.9, 128.6, 128.0, 127.5, 127.4, 126.9, 126.8, 126.7, 126.5, 125.8, 125.6, 123.3, 121.9, 119.1, 117.7. HRMS (ESI) m/z calcd for $C_{33}H_{22}ClO_2$+ $(M+H)^+$ 485.13028, found 485.13049.

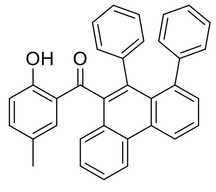

(1,10-diphenylphenanthren-9-yl)(2-hydroxy-5-methylphenyl)methanone (11): Yield 86%; 39.9 mg; yellow solid; mp 249–251°C; TLC (PET:EtOAc, 100:1 v/v): R_f = 0.3; ^{1}H NMR (600 MHz, $CDCl_3$) δ=11.84 (s, 1H), 8.93–8.86 (m, 2H), 7.82–7.70 (m, 3H), 7.59 (t, *J* = 7.2 Hz, 1H), 7.50 (d,*J* = 7.2 Hz, 1H), 7.09 (d, *J* = 7.2 Hz, 2H), 7.04 (d, *J* = 8.4 Hz, 1H), 7.00 (t, *J* = 7.2 Hz, 1H), 6.90 (ddd, *J* = 30.6, 15.0, 7.8 Hz, 4H), 6.72 (d, *J* = 7.8 Hz, 2H), 6.64–6.57 (m, 3H), 1.92 (s, 3H). ^{13}C NMR (150 MHz, $CDCl_3$) δ=205.1, 160.1, 143.3, 142.8, 139.1, 137.4, 136.1, 135.6, 132.5, 132.4, 131.9, 131.9, 130.9, 130.6, 129.8, 129.0, 128.7, 128.3, 127.5, 127.3, 126.7, 126.4, 126.4, 126.0, 125.8, 123.3, 122.0, 120.1, 117.3, 20.0. HRMS (ESI) m/z calcd for $C_{34}H_{25}O_2$+ $(M+H)^+$ 465.18491, found 465.18497.

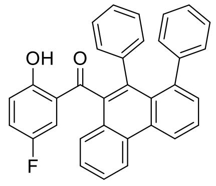

(1,10-diphenylphenanthren-9-yl)(2-hydroxy-5-methylphenyl)methanone (12): Yield 84%; 39.3 mg; yellow solid; mp 236–239°C; TLC (PET:EtOAc, 100:1 v/v): R_f = 0.3; ^{1}H NMR (600 MHz, $CDCl_3$) δ=11.67 (s, 1H), 8.82 (t, *J* = 7.2 Hz, 2H), 7.76–7.62 (m, 3H), 7.53 (t, *J* = 7.2 Hz, 1H), 7.44 (d, *J* = 7.2 Hz, 1H), 7.06 (t, *J* = 9.0 Hz, 2H), 6.95 (t, *J* = 7.2 Hz, 1H), 6.93–6.79 (m, 4H), 6.77 (d, *J* = 6.6 Hz, 1H), 6.69 (dd, *J* = 13.8, 8.4 Hz, 2H), 6.62–6.53 (m, 2H), 6.45–6.37 (m, 1H). ^{13}C NMR (150 MHz, $CDCl_3$) δ=204.4, 158.3, 155.0, 153.4, 143.2, 142.8, 138.9, 136.0, 135.3, 132.5, 132.1, 132.0, 130.9, 130.7, 129.8, 129.0, 128.6, 128.0, 127.6, 127.5, 127.0, 126.9, 126.6, 125.8, 125.6, 124.0, 123.8, 123.5, 122.0, 120.1, 120.0, 119.0, 118.9, 117.3, 117.2. ^{19}F NMR (375 MHz, $CDCl_3$) δ=124.61. HRMS (ESI) m/z calcd for $C_{33}H_{21}FO_2$+ $(M)^+$ 468.15202, found 468.15210.

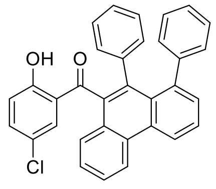

(5-chloro-2-hydroxyphenyl)(1,10-diphenylphenanthren-9-yl)methanone (13): Yield 85%; 41.2 mg; yellow solid; mp 225–228°C; TLC (PET:EtOAc, 100:1 v/v): R_f = 0.3; ^{1}H NMR (600 MHz, $CDCl_3$) δ=11.93 (s, 1H), 8.91 (t, *J* = 8.4 Hz, 2H), 7.78 (ddd, *J* = 20.4, 13.8, 7.8 Hz, 3H), 7.62 (t, *J*= 7.2 Hz, 1H), 7.53 (d, *J* = 7.2 Hz, 1H), 7.18–7.08 (m, 3H), 7.03 (t, *J* = 7.2 Hz, 1H), 6.95 (t, *J* = 7.2 Hz, 1H), 6.90 (dd, *J* = 14.4, 7.2 Hz, 2H), 6.84 (d, *J* = 7.2 Hz, 1H), 6.76 (d, *J* = 9.0 Hz, 3H), 6.69

(t, J = 7.2 Hz, 1H), 6.65 (d, J =7.2 Hz, 1H). ^{13}C NMR (150 MHz, $CDCl_3$) δ=204.4, 160.6, 143.1, 142.8, 138.9, 136.1, 136.0, 135.2, 132.5, 132.1, 131.9, 131.6, 131.0, 130.8, 129.9, 128.9, 128.4, 127.9, 127.7, 127.5, 126.8, 126.5, 125.8, 125.5, 123.5, 122.9, 122.1, 120.9, 119.2. HRMS (ESI) m/z calcd for $C_{33}H_{21}ClO_2$+ $(M)^+$ 484.12246, found 484.12259.

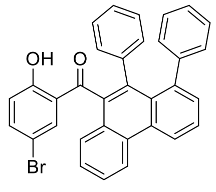

(5-bromo-2-hydroxyphenyl)(1,10-diphenylphenanthren-9-yl)methanone (14): Yield 89%; 47.0 mg; yellow solid; mp 216–220°C; TLC (PET:EtOAc, 100:1 v/v): R_f = 0.3; ^{1}H NMR (600 MHz, $CDCl_3$) δ=8.75 (t, J = 8.4 Hz, 2H), 7.63 (dt, J = 24.0, 7.8 Hz, 2H), 7.57 (d, J = 7.8 Hz, 1H), 7.46 (t, J = 7.2 Hz, 1H), 7.36 (d, J = 6.0 Hz, 1H), 7.11 (d, J = 8.4 Hz, 1H), 6.97 (s, 1H), 6.93 (s, 1H), 6.87 (s, 1H), 6.78 (s, 1H), 6.71 (s, 3H), 6.67 (d, J = 5.4 Hz, 1H), 6.58 (s, 1H), 6.54 (d, J = 9.0 Hz, 2H), 6.47 (d, J = 5.4 Hz, 1H). ^{13}C NMR (150 MHz, $CDCl_3$) δ=204.4, 161.0, 143.1, 142.8, 139.0, 138.9, 136.0, 135.1, 134.7, 132.6, 132.1, 131.8, 131.0, 130.8, 129.9, 129.0, 128.4, 127.8, 127.5, 127.0, 126.9, 126.6, 125.7, 125.5, 123.5, 122.1, 121.5, 119.6, 109.8. HRMS (ESI) m/z calcd for $C_{33}H_{22}BrO_2$+ $(M+H)^+$ 529.07977, found 529.07984.

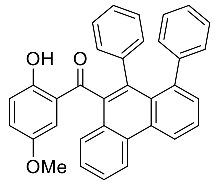

(1,10-diphenylphenanthren-9-yl)(2-hydroxy-5-methoxyphenyl)methanone (15): Yield 64%; 30.7 mg; yellow solid; mp 244–247°C; TLC (PET:EtOAc, 50:1 v/v): R_f = 0.3; ^{1}H NMR (600 MHz, $CDCl_3$) δ=11.58 (s, 1H), 8.91–8.81 (m, 2H), 7.77 (d, J = 7.8 Hz, 1H), 7.72 (d, J = 7.8 Hz, 1H), 7.67 (d, J = 7.8 Hz, 1H), 7.57 (d, J = 7.2 Hz, 1H), 7.47 (d, J = 7.2 Hz, 1H), 7.10–7.02 (m, 2H), 6.97 (s, 1H), 6.93–6.81 (m, 5H), 6.72 (dd, J = 16.2, 8.4 Hz, 2H), 6.60 (d, J = 10.2 Hz, 2H), 6.25 (d, J = 2.4 Hz, 1H), 3.37 (s, 3H). ^{13}C NMR (150 MHz, $CDCl_3$) δ=204.8, 156.7, 151.1, 143.3, 142.8, 139.1, 135.9, 135.7, 132.5, 132.0, 130.9, 130.7, 129.8, 129.0, 127.6, 127.4, 127.1, 127.0, 126.8, 126.7, 126.5, 125.9, 125.8, 124.3, 123.4, 122.0, 120.1, 118.5, 115.6, 55.7. HRMS (ESI) m/z calcd for $C_{34}H_{25}O_3$+ $(M+H)^+$ 481.17982, found 481.17985.

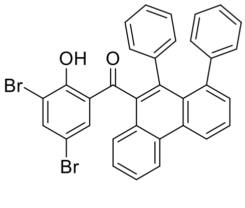

(3,5-dibromo-2-hydroxyphenyl)(1,10-diphenylphenanthren-9-yl)methanone (16): Yield 91%; 55.1 mg; yellow solid; mp 201–204°C; TLC (PET:EtOAc, 100:1 v/v): R_f = 0.3; ^{1}H NMR (600 MHz, $CDCl_3$) δ=12.57 (s, 1H), 8.90 (t, *J* = 7.8 Hz, 2H), 7.83–7.79 (m, 1H), 7.78–7.74 (m, 1H), 7.65 (d,*J* = 7.8 Hz, 1H), 7.63–7.59 (m, 1H), 7.55 (s, 1H), 7.52 (d, *J* = 6.6 Hz, 1H), 7.12 (d, *J* = 7.2 Hz, 1H), 7.05 (d, *J* = 7.2Hz, 1H), 7.03–6.99 (m, 1H), 6.93 (t, *J* = 7.2 Hz, 1H), 6.87 (s, 2H), 6.79 (d, *J* = 8.4 Hz, 2H), 6.74 (t, *J* = 7.2 Hz, 1H), 6.70–6.65 (m, 1H), 6.59 (d, *J* = 7.2 Hz, 1H). ^{13}C NMR (150 MHz, $CDCl_3$) δ=204.2, 157.7, 143.0, 142.9, 141.1, 138.8, 136.2, 134.6, 134.0, 132.6, 132.2, 131.8, 131.0, 130.8, 129.9, 128.9, 128.3, 127.8, 127.7, 127.6, 127.2, 127.1, 125.9, 125.3, 123.6, 122.1, 121.7, 112.1, 109.8. HRMS (ESI) m/z calcd for $C_{33}H_{20}Br_2O_2Na+$ $(M+Na)^+$ 630.97047, found 630.97017.

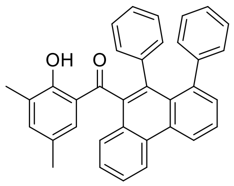

(1,10-diphenylphenanthren-9-yl)(2-hydroxy-3,5-dimethylphenyl)methanone (17): Yield 90%; 43.0 mg; yellow solid; mp 147–150°C; TLC (PET:EtOAc, 100:1 v/v): R_f = 0.3; ^{1}H NMR (600 MHz, $CDCl_3$) δ=12.18 (s, 1H), 8.90 (dd, *J* = 13.8, 8.4 Hz, 2H), 7.83–7.70 (m, 3H), 7.58 (t, *J* = 7.8 Hz, 1H), 7.51 (d, *J* = 7.2 Hz, 1H), 7.14–7.06 (m, 2H), 7.01 (t, *J* = 7.2 Hz, 1H), 6.97–6.89 (m, 3H), 6.87 (t, *J* = 7.2 Hz, 2H), 6.73 (t, *J* = 6.6 Hz, 1H), 6.67–6.61 (m, 2H), 6.50 (s, 1H), 2.16 (s, 3H), 1.90 (s, 3H). ^{13}C NMR (150 MHz, $CDCl_3$) δ=205.1, 158.7, 143.3, 142.8, 139.2, 138.5, 136.4, 135.5, 132.4, 131.9, 130.8, 130.6, 130.1, 129.8, 129.0, 128.8, 128.4, 127.5, 127.3, 126.9, 126.7, 126.6, 126.5, 126.4, 126.1, 125.7, 123.2, 121.9, 119.5, 20.1, 15.2. HRMS (ESI) m/z calcd for $C_{35}H_{27}O_2+$ $(M+H)^+$ 479.20056, found 479.20058.

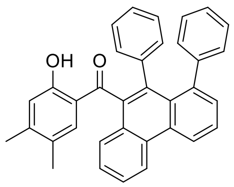

(1,10-diphenylphenanthren-9-yl)(2-hydroxy-4,5-dimethylphenyl)methanone (18): Yield 88%; 42.1 mg; yellow solid; mp 272–275°C; TLC (PET:EtOAc, 100:1 v/v): R_f = 0.3; ^{1}H NMR (600 MHz, $CDCl_3$) δ=11.87 (s, 1H), 8.89 (dd, *J* = 14.4, 8.4 Hz, 2H), 7.82–7.69 (m, 3H), 7.57 (t, *J* = 7.8 Hz, 1H), 7.49 (d, *J* = 7.2 Hz, 1H), 7.08 (d, *J* = 6.6 Hz, 2H), 6.99 (t, *J* = 7.2 Hz, 1H), 6.95–6.88 (m, 2H), 6.87 (d, *J* = 19.8Hz, 2H), 6.72 (t, *J* = 7.2 Hz, 1H), 6.61 (q, *J* = 9.0 Hz, 3H), 6.55 (s, 1H), 2.10 (s, 3H), 1.82 (s, 3H). ^{13}C NMR (150 MHz, $CDCl_3$) δ=203.9, 160.2, 146.7, 143.0, 142.5, 138.8, 135.9, 135.1, 132.6, 132.0, 131.6, 131.5, 130.5, 130.3, 129.5, 128.7, 128.5, 128.0, 127.5, 127.3, 126.8, 126.6, 126.2, 125.7, 125.4, 122.9, 121.6, 118.2, 117.8, 20.1, 18.1. HRMS (ESI) m/z calcd for $C_{35}H_{27}O_2+$ $(M+H)^+$ 479.20056, found 479.20016.

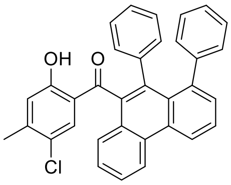

(5-chloro-2-hydroxy-4-methylphenyl)(1,10-diphenylphenanthren-9-yl)methanone (19):

Yield 87%; 43.3 mg; white solid; mp 219–222℃; TLC (PET:EtOAc, 100:1 v/v): R_f = 0.3; ^{1}H NMR (600 MHz, $CDCl_3$) δ=11.84 (s, 1H), 8.89 (t, *J* = 9.6 Hz, 2H), 7.79 (t, *J* = 7.8 Hz, 1H), 7.75 (t, *J* = 7.8 Hz, 1H), 7.68 (d, *J* = 7.8 Hz, 1H), 7.58 (t, *J* = 7.2 Hz, 1H), 7.49 (d, *J* = 7.2 Hz, 1H), 7.08 (dd, *J* = 18.0, 7.2 Hz, 2H), 6.99 (t, *J*= 7.2 Hz, 1H), 6.94–6.90 (m, 1H), 6.90–6.78 (m, 3H), 6.78–6.62 (m, 4H), 6.61 (d, *J* = 7.2 Hz, 1H), 2.19 (s, 3H). ^{13}C NMR (150 MHz, $CDCl_3$) δ=203.9, 160.5, 145.6, 143.2, 142.8, 139.0, 135.9, 135.4, 132.5, 132.1, 132.0, 131.9, 130.9, 130.7, 129.9, 129.0, 128.6, 128.0, 127.6, 127.5, 127.0, 126.9, 126.9, 126.6, 125.7, 123.6, 122.1, 119.7, 119.4, 20.7. HRMS (ESI) m/z calcd for $C_{34}H_{24}ClO_2$+ $(M+H)^+$ 499.14593, found 499.14609.

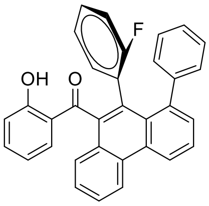

(10-(2-fluorophenyl)-1-phenylphenanthren-9-yl)(2-hydroxyphenyl)methanone (20):

Yield 82%; 38.4 mg; white solid; mp 197–199℃; TLC (PET:EtOAc, 100:1 v/v): R_f = 0.3; ^{1}H NMR (600 MHz, $CDCl_3$) δ=11.83 (s, 1H), 11.75 (s, 1H), 8.86 (dt, *J* = 16.8, 8.4 Hz, 2H), 7.79–7.60 (m, 3H), 7.54 (dd, *J* = 12.6, 5.4 Hz, 1H), 7.41 (dd, *J* = 18.0, 7.2 Hz, 1H), 7.31–7.17 (m, 2H), 7.13–7.05 (m, 1H), 7.04–6.97 (m, 1H), 6.97–6.86 (m, 2H), 6.86–6.79 (m, 1H), 6.78–6.65 (m, 3H), 6.46 (ddd, *J* = 46.8, 28.2, 19.8 Hz, 2H), 6.13 (d, *J* =7.8 Hz, 1H). ^{13}C NMR (150 MHz, $CDCl_3$) δ=204.9, 203.9, 162.1， 162.0, 159.3, 157.7, 142.7, 142.4, 142.4, 136.9, 136.6, 136.4, 133.9, 133.0, 132.7, 132.2, 132.0, 131.9, 131.7, 131.3, 130.4, 130.2, 129.8, 129.4, 129.3, 128.6, 128.2, 128.1, 128.1, 127.7, 127.5, 127.5, 127.2, 126.9, 126.8, 126.7, 126.6, 126.2, 126.0, 123.5, 122.8, 122.6, 122.4, 120.8, 120.4, 118.7, 118.6, 117.9, 117.4, 115.2, 114.6, 114.5. ^{19}F NMR (375 MHz, $CDCl_3$) δ=108.43, 109.52. HRMS (ESI) m/z calcd for $C_{33}H_{22}FO_2$+ $(M+H)^+$ 469.15984, found 469.15996.

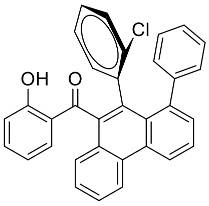

(10-(2-chlorophenyl)-1-phenylphenanthren-9-yl)(2-hydroxyphenyl)methanone (21):

Yield 60%; 29.0 mg; white solid; mp 214-217℃; TLC (PET:EtOAc, 100:1 v/v): R_f = 0.3; ^{1}H NMR (600 MHz, $CDCl_3$) δ=11.86 (s, 1H), 8.96–8.88 (m, 2H), 7.78 (dd, J = 17.4, 8.4 Hz, 2H), 7.73 (d, J = 7.8 Hz, 1H), 7.62–7.58 (m, 1H), 7.47 (d, J = 6.6 Hz, 1H), 7.42 (d, J = 9.0 Hz, 1H), 7.25 (dd, J = 14.4, 7.2 Hz, 2H), 7.05–7.01 (m, 1H), 6.99–6.92 (m, 3H), 6.87–6.83 (m, 1H), 6.79 (d, J = 6.0 Hz, 2H), 6.71–6.66 (m, 1H), 6.59 (d, J = 7.8 Hz, 1H), 6.50 (d, J = 7.2 Hz, 1H). ^{13}C NMR (150 MHz, $CDCl_3$) δ=205.1, 161.8, 142.4, 136.9, 136.8, 136.6, 136.5, 134.9, 133.6, 133.2, 133.2, 132.6, 132.5, 132.0, 131.9, 131.7, 131.5, 130.3, 128.9, 128.7, 128.6, 128.3, 128.2, 128.1, 127.8, 127.6, 127.5, 127.3, 126.7, 126.4, 126.3, 126.2, 126.1, 125.4, 125.2, 123.5, 122.4, 120.4, 118.9, 118.5, 118.0, 117.2. HRMS (ESI) m/z calcd for $C_{33}H_{21}ClO_2Na+$ $(M+Na)^+$ 507.11223, found 507.11229.

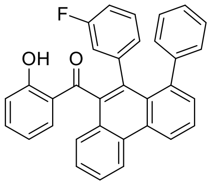

(10-(3-fluorophenyl)-1-phenylphenanthren-9-yl)(2-hydroxyphenyl)methanone (22):

Yield 76%; 35.5 mg; white solid; mp 195–198℃; TLC (PET:EtOAc, 100:1 v/v): R_f = 0.3; ^{1}H NMR (600 MHz, $CDCl_3$) δ=11.89 (d, J = 2.4 Hz, 1H), 8.88–8.74 (m, 2H), 7.76–7.61 (m, 3H), 7.51 (dd, J = 15.0, 7.8 Hz, 1H), 7.47–7.40 (m, 1H), 7.23–7.15 (m, 1H), 7.12–7.02 (m, 1H), 7.01–6.86 (m, 3H), 6.86–6.71 (m, 4H), 6.55–6.49 (m, 1H), 6.38 (ddd, J = 28.2, 13.2, 7.8 Hz, 2H), 6.22 (d, J = 9.6 Hz, 1H). ^{13}C NMR (150 MHz, $CDCl_3$) δ=204.9, 204.8, 162.4, 162.2, 162.1, 161.9, 160.5, 160.3, 143.2, 143.1, 142.7, 142.6, 141.2, 141.2, 141.1, 136.7, 136.1, 136.2, 136.2, 134.4, 134.3, 133.1, 132.8, 132.5, 132.4, 132.2, 132.0, 130.8, 130.8, 130.0, 130.0, 129.2, 129.1, 128.8, 128.8, 128.7, 128.5, 128.4, 128.3, 128.2, 128.2, 128.0, 127.8, 127.7, 127.6, 127.5, 127.2, 126.9, 126.9, 126.6, 126.2, 126.2, 126.1, 123.5, 122.2, 122.1, 120.7, 120.4, 119.4, 119.2, 118.7, 118.7, 118.0, 117.9, 117.8, 117.8, 113.6, 113.6, 113.5, 113.4. ^{19}F NMR (375 MHz, $CDCl_3$) δ=114.81, 115.53. HRMS (ESI) m/z calcd for $C_{33}H_{22}FO_2+$ $(M+H)^+$ 469.15984, found 469.15991.

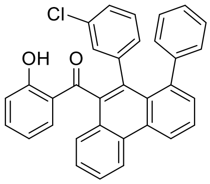

(10-(3-chlorophenyl)-1-phenylphenanthren-9-yl)(2-hydroxyphenyl)methanone (23):

Yield 81%; 39.2 mg; white solid; mp 187–190℃; TLC (PET:EtOAc, 100:1 v/v): R_f = 0.3; ^{1}H NMR (600 MHz, $CDCl_3$) δ=11.86 (d, J = 2.4 Hz, 1H), 8.89–8.73 (m, 2H), 7.77–7.60 (m, 3H), 7.51 (dd, J = 17.4, 7.8 Hz, 1H), 7.44 (dd, J = 10.8, 7.2 Hz, 1H),

7.20 (dt, J = 6.6, 6.0 Hz, 1H), 7.13–7.07 (m, 1H), 6.96 (ddt, J = 27.0, 14.4, 7.2 Hz, 4H), 6.78 (ddt, J = 25.2, 21.0, 8.4 Hz, 4H), 6.64 (dd, J = 24.6, 7.8 Hz, 1H), 6.56–6.34 (m, 3H). ^{13}C NMR (150 MHz, $CDCl_3$) δ=204.9, 204.7, 162.3, 162.2, 143.1, 143.0, 142.6, 142.6, 140.7, 136.7, 136.6, 136.3, 134.3, 134.1, 133.0, 132.9, 132.7, 132.6, 132.4, 132.3, 132.2, 132.0, 131.2, 130.8, 130.6, 130.0, 130.0, 129.2, 129.1, 128.6, 128.6, 128.4, 128.3, 128.2, 128.1, 128.1, 127.8, 127.7, 127.5, 127.5, 127.2, 126.9, 126.9, 126.9, 126.7, 126.6, 126.3, 126.2, 126.1, 123.5, 122.2, 122.2, 120.8, 120.4, 118.7, 117.9, 117.8. HRMS (ESI) m/z calcd for $C_{33}H_{22}ClO_2$+ $(M+H)^+$ 485.13028, found 485.13159.

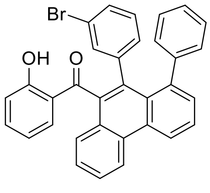

(10-(3-bromophenyl)-1-phenylphenanthren-9-yl)(2-hydroxyphenyl)methanone (24):

Yield 87%; 45.9 mg; yellow solid; mp 185–188°C; TLC (PET:EtOAc, 100:1 v/v): R_f = 0.3; ^{1}H NMR (600 MHz, $CDCl_3$) δ=11.95 (s, 1H), 8.98–8.86 (m, 2H), 7.86–7.71 (m, 3H), 7.67–7.59 (m, 1H), 7.54 (dd, J =11.4, 7.2 Hz, 1H), 7.37–7.17 (m, 3H), 7.12 (d, J = 6.6 Hz, 1H), 7.03 (dd, J = 26.4, 6.6 Hz, 2H), 6.97–6.83 (m, 4H), 6.79–6.74 (m, 1H), 6.62–6.45 (m, 2H). ^{13}C NMR (150 MHz, $CDCl_3$) δ=204.9, 204.7, 162.3, 162.2, 143.1, 142.9, 142.6, 142.5, 141.0, 136.8, 136.6, 136.3, 135.2, 134.2, 134.1, 133.0, 132.7, 132.4, 132.3, 132.2, 132.0, 130.9, 130.5, 130.4, 130.2, 129.5, 129.5, 129.2, 129.1, 129.0, 128.5, 128.3, 128.2, 128.2, 128.1, 127.8, 127.5, 127.5, 127.2, 126.9, 126.9, 126.3, 126.2, 126.1, 123.5, 122.3, 122.2, 121.4, 121.1, 120.8, 120.3, 118.7, 118.0, 117.8. HRMS (ESI) m/z calcd for $C_{33}H_{22}BrO_2$+ $(M+H)^+$ 529.07977, found 529.07984.

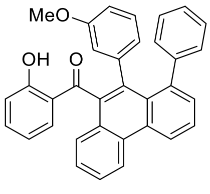

(2-hydroxyphenyl)(10-(3-methoxyphenyl)-1-phenylphenanthren-9-yl)methanone (25):

Yield 85%; 40.8 mg; white solid; mp 153–156°C; TLC (PET:EtOAc, 50:1 v/v): R_f = 0.3; ^{1}H NMR (600 MHz, $CDCl_3$) δ=11.99 (s, 0.5H), 11.91 (s, 0.5H), 8.88–8.71 (m, 2H), 7.81–7.55 (m, 3H), 7.55–7.35 (m, 2H), 7.17 (dd, J = 8.4, 4.8 Hz, 1H), 7.07 (d, J = 7.2 Hz, 1H), 7.00 (d, J = 6.6 Hz, 1H), 6.99–6.62 (m, 7H), 6.60 (s, 1H), 6.51–6.35 (m, 2H), 6.19 (dd, J = 44.4, 7.8 Hz, 2H), 6.04 (s, 1H), 3.61 (s, 1H), 3.30 (s, 2H). ^{13}C NMR (150 MHz, $CDCl_3$) δ=205.4, 205.1, 162.3, 162.2, 158.0, 157.6, 143.3, 143.3, 143.0, 142.8, 140.4, 140.2, 136.5, 136.4, 135.9, 135.8, 135.7, 135.6, 133.2, 133.0, 132.5, 132.2, 131.9, 130.6, 129.2, 129.2, 128.6, 128.3, 128.3, 128.1, 127.7,

127.6, 127.5, 127.2, 127.1, 126.8, 126.0, 126.0, 125.9, 125.1, 123.6, 123.4, 122.2, 122.0, 120.9, 120.7, 118.6, 118.5, 117.8, 117.7, 117.4, 116.1, 113.4, 113.3, 55.2, 54.8. HRMS (ESI) m/z calcd for $C_{34}H_{25}O_3$+ $(M+H)^+$ 481.17982, found 481.17986.

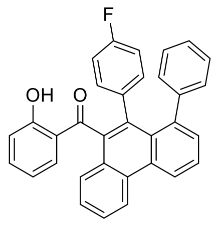

(10-(4-fluorophenyl)-1-phenylphenanthren-9-yl)(2-hydroxyphenyl)methanone (26):

Yield 77%; 36.0 mg; yellow solid; mp 269–272℃; TLC (PET:EtOAc, 100:1 v/v): R_f = 0.3; ^{1}H NMR (600 MHz, $CDCl_3$) δ=11.89 (s, 1H), 8.85 (t, *J* = 9.6 Hz, 2H), 7.73 (dt, *J* = 15.6, 7.2 Hz, 2H), 7.66 (d, *J* = 7.8 Hz, 1H), 7.55 (t, *J* = 7.2 Hz, 1H), 7.46 (d, *J* = 7.2 Hz, 1H), 7.23 (d, *J* = 8.4 Hz, 1H), 6.99 (d, *J* = 28.2 Hz, 4H), 6.92–6.87 (m, 1H), 6.79 (dd, *J* = 19.2, 8.4 Hz, 3H), 6.51 (dd, *J* = 15.0, 6.6 Hz, 2H), 6.44 (t, *J* = 7.2 Hz, 1H), 6.25 (t, *J* = 7.2 Hz, 1H). ^{13}C NMR (150 MHz, $CDCl_3$) δ=205.1, 162.3, 161.9, 160.2, 143.3, 142.6, 136.6, 136.2, 134.9, 134.7, 133.7, 132.9, 132.4, 132.0, 130.8, 129.8, 128.9, 128.2, 127.6, 127.0, 126.7, 125.9, 123.4, 122.1, 120.5, 118.6, 117.8, 113.8. ^{19}F NMR (375 MHz, $CDCl_3$) δ=115.65. HRMS (ESI) m/z calcd for $C_{33}H_{21}FO_2Na$+ $(M+Na)^+$ 491.14178, found 491.14185.

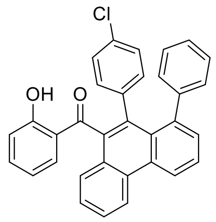

(10-(4-chlorophenyl)-1-phenylphenanthren-9-yl)(2-hydroxyphenyl)methanone (27):

Yield 80%; 38.7 mg; yellow soild; mp 237–239℃; TLC (PET:EtOAc, 100:1 v/v): R_f = 0.3; ^{1}H NMR (600 MHz, $CDCl_3$) δ=11.90 (s, 1H), 8.88–8.80 (m, 2H), 7.77–7.68 (m, 2H), 7.65 (d, *J* = 7.8 Hz, 1H), 7.56–7.52 (m, 1H), 7.45 (d, *J* = 7.2 Hz, 1H), 7.26–7.21 (m, 1H), 7.03–6.94 (m, 4H), 6.89 (s, 1H), 6.80 (dd, *J* = 18.0, 9.0Hz, 3H), 6.75 (d, *J* = 7.2 Hz, 1H), 6.52 (d, *J* = 7.8 Hz, 1H), 6.44 (dd, *J* = 12.6, 7.2 Hz, 2H). ^{13}C NMR (150 MHz, $CDCl_3$) δ=204.9, 162.3, 143.2, 142.5, 137.4, 136.6, 136.1, 134.4, 133.2, 132.8, 132.4, 132.3, 131.9, 130.7, 129.8, 129.1, 128.8, 128.4, 128.1, 127.9, 126.8, 126.7, 126.0, 125.9, 123.3, 122.1, 120.5, 118.6, 117.9. HRMS (ESI) m/z calcd for $C_{33}H_{21}ClO_2Na$+ $(M+Na)^+$ 507.11223, found 507.11228.

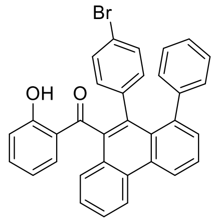

(10-(4-bromophenyl)-1-phenylphenanthren-9-yl)(2-hydroxyphenyl)methanone (28):

Yield 84%; 44.3 mg; white solid; mp 225–228°C; TLC (PET:EtOAc, 100:1 v/v): R_f = 0.3; ^{1}H NMR (600 MHz, $CDCl_3$) δ=12.04 (s, 1H), 8.99–8.83 (m, 2H), 7.78 (dd, *J* = 24.0, 7.2 Hz, 3H), 7.64–7.57 (m, 1H), 7.52 (d, *J* = 6.6 Hz, 1H), 7.34–7.28 (m, 1H), 7.06 (dd, *J* = 26.4, 13.2 Hz, 4H), 7.02–6.94 (m, 2H), 6.91 (d, *J* = 7.8Hz, 2H), 6.82 (d, *J* = 7.2 Hz, 1H), 6.77 (d, *J* = 7.8 Hz, 1H), 6.50 (dd, *J* = 12.6, 7.2 Hz, 2H). ^{13}C NMR (150 MHz, $CDCl_3$) δ=204.8, 162.2, 143.0, 142.4, 137.8, 136.6, 135.9, 134.3, 133.4, 132.7, 132.2, 132.1, 131.9, 130.6, 129.9, 129.8, 129.0, 128.6, 128.0, 127.4, 126.7, 125.9, 125.7, 123.3, 122.0, 120.8, 120.4, 118.6, 117.8. HRMS (ESI) m/z calcd for $C_{33}H_{22}BrO_2$+ $(M+H)^+$ 529.07977, found 529.07986.

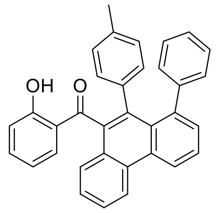

(2-hydroxyphenyl)(1-phenyl-10-(p-tolyl)phenanthren-9-yl)methanone (29):

Yield 69%; 32.0 mg; yellow solid; mp 195–197°C; TLC (PET:EtOAc, 100:1 v/v): R_f = 0.3; ^{1}H NMR (600 MHz, $CDCl_3$) δ=11.95 (s, 1H), 8.86 (t, *J* = 8.4 Hz, 2H), 7.73 (d, *J* = 22.8 Hz, 2H), 7.65 (d, *J* = 8.4 Hz, 1H), 7.55 (d, *J* = 7.2 Hz, 1H), 7.45 (d, *J* = 7.2 Hz, 1H), 7.23 (d, *J* = 8.4 Hz, 1H), 6.99 (s, 1H), 6.96–6.89 (m, 3H), 6.85 (d, *J* = 7.8 Hz, 2H), 6.82–6.77 (m, 2H), 6.61 (d, *J* = 7.2 Hz, 1H), 6.46 (d, *J* = 7.2 Hz, 1H), 6.40 (d, *J* = 7.8 Hz, 1H), 6.34 (d, *J* = 7.8 Hz, 1H), 2.00 (s, 3H). ^{13}C NMR (150 MHz, $CDCl_3$) δ=205.3, 162.2, 143.4, 142.9, 136.3, 136.0, 135.9, 133.1, 132.4, 132.0, 131.9, 130.7, 130.6, 129.8, 129.6, 129.2, 129.1, 128.3, 128.0, 127.1, 127.0, 127.1, 125.9, 125.3, 123.3, 122.0, 120.7, 118.5, 117.6, 20.8. HRMS (ESI) m/z calcd for $C_{34}H_{24}$ O_2Na+ $(M+Na)^+$ 487.16685, found 487.16694.

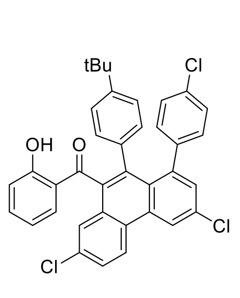

(10-(4-(tert-butyl)phenyl)-3,7-dichloro-1-(4-chlorophenyl)phenanthren-9-yl)(2-hydroxyphenyl)methanone (30):

Yield 65%; 39.5 mg; yellow solid; mp 258–260°C; TLC (PET:EtOAc, 100:1 v/v): R_f = 0.3; ^{1}H NMR (600 MHz, $CDCl_3$) δ=11.66 (s, 1H), 8.77 (s, 1H), 8.69 (d, *J* = 9.0 Hz, 1H), 7.68 (d, *J* = 8.4 Hz, 2H), 7.41 (d, *J*= 1.8 Hz, 1H), 7.23 (t, *J* = 7.2 Hz, 1H), 6.96–6.87 (m, 4H), 6.82–6.74 (m, 3H), 6.66 (d, *J* = 7.8 Hz, 1H), 6.57 (d, *J* =7.8 Hz, 1H), 6.46 (t, *J* = 7.8 Hz, 1H), 6.36 (d, *J* = 7.8 Hz, 1H), 1.11 (s, 9H). ^{13}C NMR (150 MHz, $CDCl_3$) δ=203.7, 162.0, 149.8, 143.3, 140.1, 136.6, 136.4, 135.2, 134.8, 134.2, 133.1, 133.0, 132.5, 132.0, 131.8, 131.7, 130.4, 130.1, 129.5, 128.2, 128.0, 127.0, 126.9, 124.9, 123.8, 121.8, 120.3, 118.3, 117.5, 34.0, 30.8. HRMS (ESI) m/z calcd for $C_{37}H_{28}Cl_3O_2$+ $(M+H)^+$ 609.11494, found 609.11503.

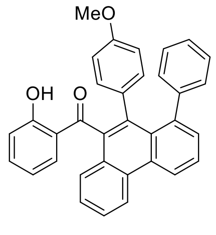

(2-hydroxyphenyl)(10-(4-methoxyphenyl)-1-phenylphenanthren-9-yl)methanone (31):

Yield 78%; 37.4 mg; yellow solid; mp 180–183°C; TLC (PET:EtOAc, 50:1 v/v): R_f = 0.3; ^{1}H NMR (600 MHz, $CDCl_3$) δ=12.19 (s, 1H), 9.00–8.84 (m, 2H), 7.85–7.70 (m, 3H), 7.65–7.51 (m, 2H), 7.30–7.25 (m, 1H), 7.19–7.09 (m, 3H), 7.06–7.01 (m, 1H), 6.93 (ddd, *J* = 29.4, 20.4, 6.6 Hz, 4H), 6.57 (d, *J* = 8.4 Hz, 1H), 6.49 (s, 2H), 6.23 (d, *J* = 8.4 Hz, 1H), 3.63 (s, 3H). ^{13}C NMR (150 MHz, $CDCl_3$) δ=205.4, 162.4, 157.9, 143.6, 142.9, 136.5, 136.1, 135.6, 133.2, 132.5, 132.1, 131.5, 130.8, 129.9, 129.7, 129.2, 128.4, 127.7, 127.4, 127.0, 126.7, 125.8, 123.5, 122.2, 120.7, 118.7, 117.8, 113.1, 112.3, 55.1. HRMS (ESI) m/z calcd for $C_{34}H_{24}O_3$+ $(M+)^+$ 480.17199, found 480.17277.

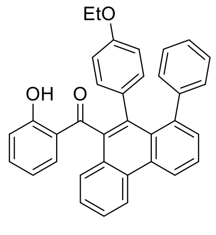

(10-(4-ethoxyphenyl)-1-phenylphenanthren-9-yl)(2-hydroxyphenyl)methanone (32):

Yield 81%; 40.0 mg; white solid; mp 192–195°C; TLC (PET:EtOAc, 50:1 v/v): R_f = 0.3; ^{1}H NMR (600 MHz, $CDCl_3$) δ=12.00 (s, 1H), 8.78 (t, *J* = 9.0 Hz, 2H), 7.71–7.56 (m, 3H), 7.52–7.36 (m, 2H), 7.14 (t, *J*= 7.8 Hz, 1H), 7.02 (d, *J* = 7.2 Hz, 1H), 6.96 (dd, *J* = 11.4, 4.8 Hz, 2H), 6.89 (t, *J* = 7.2 Hz, 1H), 6.84 (t, *J* = 7.2 Hz, 1H), 6.82–6.69 (m, 3H), 6.36 (ddd, *J* = 17.4, 8.4, 4.8 Hz, 3H), 6.07 (dd, *J* = 8.4, 2.4 Hz, 1H), 3.73 (dd, *J* = 9.6, 4.2Hz, 2H), 1.22 (t, *J* = 7.2 Hz, 3H). ^{13}C NMR (150 MHz, $CDCl_3$) δ=205.4, 162.4, 157.1, 143.6, 142.9, 136.4, 136.0, 135.7, 133.3, 133.1, 132.5, 132.1, 131.4, 130.8, 129.8, 129.3, 129.0, 128.4, 127.6, 127.4, 127.0, 126.7, 125.9, 125.7,

123.4, 122.1, 120.7, 118.6, 117.8, 113.8, 113.0, 63.2, 14.7. HRMS (ESI) m/z calcd for $C_{35}H_{26}O_3$+ $(M)^+$ 494.18765, found 494.18829.

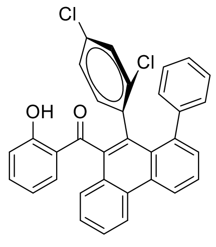

(10-(2,4-dichlorophenyl)-1-phenylphenanthren-9-yl)(2-hydroxyphenyl)methanone (33):

Yield 73%; 37.8 mg; white solid; mp 210–213°C; TLC (PET:EtOAc, 100:1 v/v): R_f = 0.3; ^{1}H NMR (600 MHz, $CDCl_3$) δ=11.90 (s, 1H), 11.77 (s, 1H), 9.01–8.85 (m, 2H), 7.79 (dd, J = 15.0, 7.2 Hz, 2H), 7.68 (dd, J = 39.0, 7.8 Hz, 1H), 7.63–7.56 (m, 1H), 7.44 (ddd, J = 22.2, 16.2, 6.6 Hz, 2H), 7.34–7.17 (m, 2H), 7.15–7.05 (m, 2H), 7.02–6.79 (m, 4H), 6.74 (d, J = 7.2 Hz, 1H), 6.66–6.55 (m, 1H), 6.51 (t, J = 7.8 Hz, 1H). ^{13}C NMR (150 MHz, $CDCl_3$) δ=204.7, 203.6, 162.2, 162.0, 142.5, 142.4, 142.1, 142.0, 137.0, 136.8, 136.1, 135.6, 135.6, 134.4, 133.8, 133.7, 133.2, 133.0, 132.0, 131.9, 131.6, 131.3, 130.3, 128.8, 128.4, 128.2, 128.1, 128.0, 127.9, 127.7, 127.6, 127.5, 126.8, 126.7, 126.5, 126.4, 126.3, 126.2, 125.8, 125.5, 123.5, 122.7, 122.5, 120.3, 119.1, 118.6, 118.3, 117.6. HRMS (ESI) m/z calcd for $C_{33}H_{21}Cl_2O_2$+ $(M+H)^+$ 519.09131, found 519.09136.

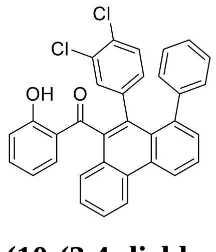

(10-(3,4-dichlorophenyl)-1-phenylphenanthren-9-yl)(2-hydroxyphenyl)methanone (34):

Yield 75%; 38.8 mg; white solid; mp 236–240°C; TLC (PET:EtOAc, 100:1 v/v): R_f = 0.3; ^{1}H NMR (600 MHz, $CDCl_3$) δ=11.84 (d, J = 5.4 Hz, 1H), 8.93–8.76 (m, 2H), 7.75 (dt, J = 11.4, 7.2 Hz, 2H), 7.65 (dd, J = 28.8, 7.8 Hz, 1H), 7.56 (dd, J = 16.2, 8.4 Hz, 1H), 7.47 (dd, J = 12.0, 7.2 Hz, 1H), 7.27 (dd, J = 14.4, 7.2 Hz, 1H), 7.13 (dd, J = 25.2, 17.4 Hz, 1H), 7.01 (dd, J = 21.6, 14.4 Hz, 2H), 6.97–6.72 (m, 6H), 6.65–6.56 (m, 1H), 6.53–6.45 (m, 1H), 6.39 (d, J = 8.4 Hz, 1H). ^{13}C NMR (150 MHz, $CDCl_3$) δ=204.6, 204.4, 162.4, 162.3, 143.0, 142.9, 142.4, 142.3, 138.9, 137.0, 136.8, 136.3, 136.3, 134.2, 133.0, 132.8, 132.6, 132.3, 132.3, 132.1, 132.0, 131.2, 130.7, 130.4, 129.7, 129.1, 129.1, 129.0, 128.7, 127.9, 127.9, 127.8, 127.8, 127.8, 127.5, 127.3, 127.3, 127.0, 126.9, 126.2, 126.1, 126.1, 126.0, 123.4, 122.3, 122.2, 120.7, 120.3, 118.8, 118.1, 118.0. HRMS (ESI) m/z calcd for $C_{33}H_{21}Cl_2O_2$+ $(M+H)^+$ 519.09131, found 519.09176.

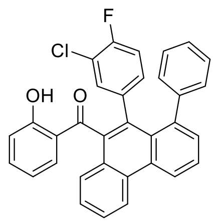

(10-(3-chloro-4-fluorophenyl)-1-phenylphenanthren-9-yl)(2-hydroxyphenyl)methanone (35):

Yield 77%; 38.6 mg; yellow solid; mp 233–237℃; TLC (PET:EtOAc, 100:1 v/v): R_f = 0.3; ^{1}H NMR (600 MHz, $CDCl_3$) δ=11.87 (d, *J* = 6.0 Hz, 1H), 8.90 (dd, *J* = 15.6, 7.8 Hz, 2H), 7.79 (dd, *J* = 15.6, 7.8 Hz, 2H), 7.69 (dd, *J* = 30.6, 7.8 Hz, 1H), 7.59 (dd, *J* = 15.6, 8.4 Hz, 1H), 7.50 (dd, *J* = 11.4, 7.2 Hz, 1H), 7.31 (dd, *J* =13.2, 7.2 Hz, 1H), 7.18 (t, *J* = 7.2 Hz, 1H), 7.15–7.03 (m, 2H), 7.00 (d, *J* = 7.8 Hz, 2H), 6.94–6.83 (m, 3H), 6.78 (t, *J*= 8.4 Hz, 1H), 6.64–6.55 (m, 1H), 6.51 (dt, *J* = 11.4, 7.8 Hz, 1H), 6.38 (dd, *J* = 35.4, 27.0 Hz, 1H). ^{13}C NMR (150 MHz, $CDCl_3$) δ=204.7, 204.6, 162.4, 162.3, 157.0, 155.3, 143.1, 143.0, 142.4, 142.3, 136.9, 136.8, 136.5, 136.0, 134.5, 133.3, 133.1, 132.8, 132.6, 132.4, 132.3, 132.2, 132.0, 131.7, 131.7, 130.7, 130.4, 130.3, 130.0, 130.0, 129.0, 128.7, 128.3, 128.0, 127.8, 127.8, 127.7, 127.5, 127.4, 127.2, 126.9, 126.9, 126.8, 126.2, 126.1, 126.0, 123.4, 122.3, 122.2, 120.6, 120.2, 118.8, 118.1, 118.0, 115.1, 115.0, 114.8. ^{19}F NMR (375 MHz, $CDCl_3$) δ=117.92, 117.98. HRMS (ESI) m/z calcd for $C_{33}H_{21}ClFO_2$+ $(M+H)^+$ 503.12086, found 503.12104.

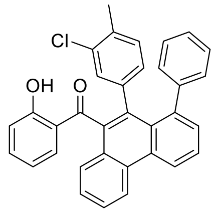

(10-(3-chloro-4-methylphenyl)-1-phenylphenanthren-9-yl)(2-hydroxyphenyl)methanone (36):

Yield 82%; 40.8 mg; white solid; mp 201–204℃; TLC (PET:EtOAc, 100:1 v/v): R_f = 0.3; ^{1}H NMR (600 MHz, $CDCl_3$) δ=11.89 (d, *J* = 6.0 Hz, 1H), 8.83–8.68 (m, 2H), 7.68–7.55 (m, 3H), 7.50–7.42 (m, 1H), 7.38 (dd, *J* = 12.6, 7.2 Hz, 1H), 7.20–7.13 (m, 1H), 7.03 (s, 1H), 6.98–6.78 (m, 4H), 6.80–6.67 (m, 3H), 6.59 (d, *J* =7.8 Hz, 1H), 6.45–6.28 (m, 3H), 1.94 (d, *J* = 14.4 Hz, 3H). ^{13}C NMR (150 MHz, $CDCl_3$) δ=205.0, 204.8, 162.4, 162.3, 143.3, 143.1, 142.7, 142.6, 137.9, 136.7, 136.6, 136.1, 134.4, 134.2, 134.0, 134.1, 133.1, 133.0, 132.9, 132.8, 132.8, 132.4, 132.3, 132.2, 132.0, 131.7, 130.8, 130.4, 130.2, 130.0, 129.9, 129.6, 129.4, 129.2, 129.1, 129.0, 128.7, 128.6, 128.2, 128.2, 127.7, 127.7, 127.6, 127.6, 127.2, 127.1, 126.8, 126.8, 126.6, 126.1, 126.0, 125.7, 125.6, 123.5, 122.3, 122.2, 120.9, 120.5, 118.7, 117.9, 117.8, 19.5, 19.4. HRMS (ESI) m/z calcd for $C_{34}H_{24}ClO_2$+ $(M+H)^+$ 499.14593, found 499.14608.

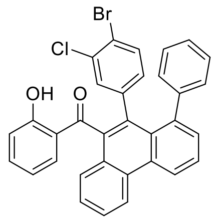

(10-(4-bromo-3-chlorophenyl)-1-phenylphenanthren-9-yl)(2-hydroxyphenyl)methanone (37):

Yield 85%; 47.7 mg; white solid; mp 241–243℃; TLC (PET:EtOAc, 100:1 v/v): R_f = 0.3; ^{1}H NMR (600 MHz, $CDCl_3$) δ=11.85 (d, *J* = 4.8 Hz, 1H), 8.93–8.76 (m, 2H), 7.70 (ddd, *J* = 36.6, 22.2, 7.8 Hz, 3H), 7.54 (dd, *J* = 16.2, 8.4 Hz, 1H), 7.46 (dd, *J* = 11.4, 7.2 Hz, 1H), 7.31–7.24 (m, 1H), 7.17–6.91 (m, 5H), 6.92–6.69 (m, 5H), 6.58 (s, 1H), 6.52–6.43 (m, 1H), 6.31 (d, *J* = 7.8 Hz, 1H). ^{13}C NMR (150 MHz, $CDCl_3$) δ=204.6, 204.4, 162.4, 162.3, 143.0, 142.9, 142.4, 142.3, 139.6, 137.0, 136.9, 136.2, 136.2, 134.0, 132.8, 132.8, 132.6, 132.4, 132.3, 132.2, 132.0, 132.1, 131.3, 130.7, 130.4, 130.1, 130.0, 129.8, 129.2, 129.1, 128.6, 128.2, 128.0, 128.0, 127.9, 127.9, 127.8, 127.8, 127.5, 127.4, 127.3, 127.0, 126.9, 126.8, 126.2, 126.1, 126.0, 123.5, 122.3, 122.2, 120.8, 120.7, 120.3, 118.8, 118.2, 118.0. HRMS (ESI) m/z calcd for $C_{33}H_{21}BrClO_2$+ $(M+H)^+$ 563.04080, found 563.04084.

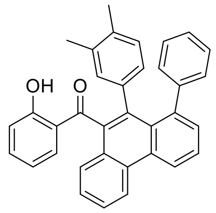

(10-(3,4-dimethylphenyl)-1-phenylphenanthren-9-yl)(2-hydroxyphenyl)methanone (38):

Yield 89%; 42.5 mg; white solid; mp 175–178℃; TLC (PET:EtOAc, 100:1 v/v): R_f = 0.3; ^{1}H NMR (600 MHz, $CDCl_3$) δ=12.13 (d, *J* = 2.4 Hz, 1H), 8.95–8.82 (m, 2H), 7.82–7.68 (m, 3H), 7.55 (ddd, *J* = 22.2, 15.6, 7.8 Hz, 2H), 7.26 (dd, *J* = 18.0, 7.8 Hz, 1H), 7.12 (dd, *J* = 15.6, 7.2 Hz, 1H), 7.07–6.92 (m, 4H), 6.93–6.77 (m, 3H), 6.68 (d, *J* = 7.8 Hz, 1H), 6.50 (dt, *J* = 24.0, 7.8 Hz, 1H), 6.41 (d, *J* = 6.0 Hz, 1H), 6.34 (s, 1H), 2.04 (s, 1.5H), 1.97 (d, *J* = 9.6 Hz, 3H), 1.78 (s, 1.5H). ^{13}C NMR (150 MHz, $CDCl_3$) δ=205.5, 205.4, 162.3, 143.6, 143.5, 143.1, 143.0, 136.5, 136.4, 136.3, 136.2, 136.2, 136.1, 136.0, 135.7, 135.6, 134.6, 134.5, 134.5, 134.3, 133.8, 133.3, 133.2, 132.7, 132.5, 132.4, 132.2, 131.9, 130.7, 130.4, 129.9, 129.8, 129.6, 129.5, 129.1, 128.5, 128.4, 128.3, 128.1, 127.6, 127.5, 127.3, 127.2, 127.1, 127.0, 126.6, 126.6, 126.4, 126.1, 126.0, 125.9, 125.2, 125.1, 123.5, 122.2, 122.1, 121.0, 120.9, 118.7, 118.4, 117.7, 117.6, 19.3, 19.1, 19.1. HRMS (ESI) m/z calcd for : $C_{35}H_{27}O_2$+ $(M+H)^+$ 479.20056, found 479.20060.

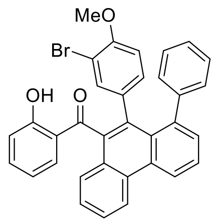

(10-(3-bromo-4-methoxyphenyl)-1-phenylphenanthren-9-yl)(2-hydroxyphenyl)methanone (39):

Yield 91%; 50.8 mg; white solid; mp 214–217℃; TLC (PET:EtOAc, 50:1 v/v): R_f = 0.3; ^{1}H NMR (600 MHz, $CDCl_3$) δ=11.93 (d, *J* = 9.6 Hz, 1H), 8.88–8.73 (m, 2H), 7.66 (ddd, *J* = 25.8, 16.2, 7.2 Hz, 3H), 7.51 (dd, *J* = 13.8, 7.2 Hz, 1H), 7.43 (dd, *J* = 12.0, 7.2 Hz, 1H), 7.23–7.10 (m, 3H), 7.01 (dd, *J* = 11.4, 4.8 Hz, 1H), 6.92 (dd, *J* = 36.0, 6.0 Hz, 2H), 6.86–6.74 (m, 2H), 6.70 (dd, *J* = 32.4, 4.8 Hz, 2H), 6.40 (ddd, *J* = 60.0, 29.4, 5.4Hz, 2H), 6.07 (d, *J* = 8.4 Hz, 1H), 3.60 (d, *J* = 28.2 Hz, 3H). ^{13}C NMR (150 MHz, $CDCl_3$) δ=205.1, 204.8, 162.4, 162.3, 154.0, 154.0, 143.3, 143.2, 142.6, 142.6, 137.0, 136.8, 136.6, 136.3, 136.3, 136.0, 133.9, 133.0, 132.7, 132.4, 132.3, 132.2, 132.1, 132.1, 130.7, 130.3, 130.0, 129.9, 129.1, 128.9, 128.6, 128.1, 127.7, 127.7, 127.6, 127.6, 127.3, 127.0, 126.8, 126.8, 126.8, 126.1, 125.9, 125.8, 123.5, 123.4, 122.3, 122.2, 120.9, 120.3, 118.8, 118.7, 118.0, 117.8, 110.6, 110.6, 110.5, 110.5, 56.1, 56.1. HRMS (ESI) m/z calcd for $C_{34}H_{23}BrO_3Na+$ $(M+Na)^+$ 581.072279, found 581.072283.

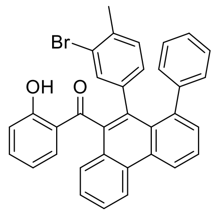

(10-(3-bromo-4-methylphenyl)-1-phenylphenanthren-9-yl)(2-hydroxyphenyl)methanone (40):

Yield 90%; 48.8 mg; white solid; mp 207–210℃; TLC (PET:EtOAc, 100:1 v/v): R_f = 0.3; ^{1}H NMR (600 MHz, $CDCl_3$) δ=11.93 (s, 1H), 8.85–8.69 (m, 2H), 7.73–7.56 (m, 3H), 7.52–7.44 (m, 1H), 7.40 (dd, *J* =13.8, 7.2 Hz, 1H), 7.21–6.98 (m, 3H), 6.98–6.83 (m, 3H), 6.83–6.73 (m, 3H), 6.72–6.64 (m, 1H), 6.62 (d, *J* = 7.8 Hz, 1H), 6.52–6.27 (m, 2H), 1.98 (d, *J* = 14.4 Hz, 3H). ^{13}C NMR (150 MHz, $CDCl_3$) δ=205.0, 204.8, 162.4, 162.3, 143.3, 143.1, 142.7, 142.6, 138.1, 138.1, 136.7, 136.6, 136.2, 136.1, 136.0, 135.8, 135.8, 134.9, 134.2, 134.1, 133.1, 132.8, 132.4, 132.3, 132.2, 132.1, 130.8, 130.4, 130.0, 130.0, 129.3, 129.3, 129.1, 129.1, 128.6, 128.2, 127.7, 127.7, 127.6, 127.3, 127.2, 127.1, 126.9, 126.7, 126.2, 126.0, 125.7, 125.6, 123.9, 123.7, 123.5, 122.3, 122.2, 121.0, 120.5, 118.8, 118.0, 117.8, 22.3, 22.3. HRMS (ESI) m/z calcd for $C_{34}H_{24}BrO_2+$ $(M+H)^+$ 543.09542, found 543.09564.

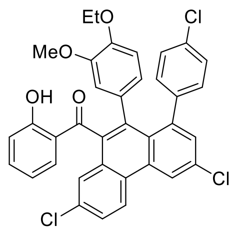

(3,7-dichloro-1-(4-chlorophenyl)-10-(4-ethoxy-3-methoxyphenyl)phenanthren-9-yl)(2-hydroxyphenyl)methanone (41):

Yield 71%; 44.4 mg; white solid; mp 226–229°C; TLC (PET:EtOAc, 20:1 v/v): R_f = 0.3; ^{1}H NMR (600 MHz, $CDCl_3$) δ=11.84 (s, 1H), 11.77 (s, 1H), 8.81–8.54 (m, 2H), 7.74–7.49 (m, 2H), 7.38 (d, *J* = 4.8Hz, 1H), 7.21 (dd, *J* = 16.2, 7.8 Hz, 1H), 7.04–6.82 (m, 3H), 6.83–6.68 (m, 3H), 6.69–6.60 (m, 1H), 6.53 (d, *J* = 8.4 Hz, 1H), 6.49–6.39 (m, 1H), 6.36 (d, *J* = 8.4 Hz, 1H), 6.10 (d, *J* = 8.4 Hz, 1H), 5.96 (d, *J* = 39.0 Hz, 1H), 3.97–3.65 (m, 3H), 3.35 (s, 2H), 1.41–1.22 (m, 3H). ^{13}C NMR (150 MHz, $CDCl_3$) δ=204.0, 203.5, 162.4, 162.3, 147.9, 147.2, 147.1, 147.0, 143.2, 143.1, 140.2, 140.0, 136.7, 136.1, 135.9, 135.0, 134.3, 133.2, 133.0, 132.6, 132.4, 132.1, 132.0, 131.6, 131.3, 130.2, 130.0, 129.6, 129.5, 129.4, 128.1, 127.5, 127.4, 127.1, 127.0, 126.7, 125.2, 125.0, 124.8, 123.8, 121.9, 121.8, 120.1, 118.5, 118.2, 117.9, 115.5, 114.2, 112.3, 111.5, 64.4, 64.1, 55.7, 55.2, 14.4. HRMS (ESI) m/z calcd for $C_{36}H_{25}Cl_3O_4$+ $(M)^+$ 626.08129, found 626.08127.

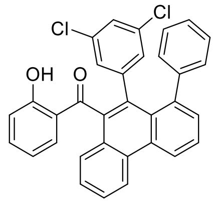

(10-(3,5-dichlorophenyl)-1-phenylphenanthren-9-yl)(2-hydroxyphenyl)methanone (42):

Yield 84%; 43.5 mg; white solid; mp 182–185°C; TLC (PET:EtOAc, 100:1 v/v): R_f = 0.3; ^{1}H NMR (600 MHz, $CDCl_3$) δ=11.99 (s, 1H), 8.92 (dd, *J* = 12.0, 9.0 Hz, 2H), 7.80 (dt, *J* = 13.8, 7.2 Hz, 3H), 7.66–7.48 (m, 2H), 7.33 (t, *J* = 7.2 Hz, 1H), 7.19 (s, 2H), 7.09 (s, 3H), 6.92 (dd, *J* = 33.6, 7.8 Hz, 3H), 6.77 (s, 1H), 6.54 (d, *J* = 9.6 Hz, 2H). ^{13}C NMR (150 MHz, $CDCl_3$) δ=204.3, 162.3, 142.6, 142.2, 141.7, 136.8, 136.4, 133.6, 133.2, 132.6, 132.5, 132.2, 132.0, 130.5, 130.4, 130.0, 129.2, 129.1, 128.0, 127.9, 127.4, 126.9, 126.5, 126.2, 123.4, 122.2, 120.4, 118.7, 118.0. HRMS (ESI) m/z calcd for $C_{33}H_{21}Cl_2O_2$+ $(M+H)^+$ 519.09131, found 519.09156.

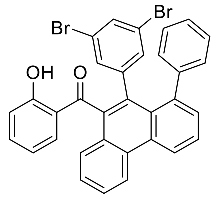

(10-(3,5-dibromophenyl)-1-phenylphenanthren-9-yl)(2-hydroxyphenyl)methanone (43):

Yield 90%; 54.5 mg; white solid; mp 190–193°C; TLC (PET:EtOAc, 100:1 v/v): R_f = 0.3; ^{1}H NMR (600 MHz, $CDCl_3$) δ=11.98 (s, 1H), 8.98–8.79 (m, 2H), 7.80 (dd, *J* = 18.6, 7.8 Hz, 3H), 7.66–7.50 (m, 2H), 7.32 (d, *J* = 7.2 Hz, 1H), 7.27 (s, 1H), 7.20 (d, *J* = 16.8 Hz, 2H), 7.09 (d, *J* = 21.6 Hz, 3H), 6.95 (d, *J* = 8.4 Hz, 1H), 6.88 (s, 2H), 6.74 (s, 1H), 6.53 (d, *J* = 6.6 Hz, 1H). ^{13}C NMR (150 MHz, $CDCl_3$) δ=204.4, 162.5, 142.7, 142.3, 137.0, 136.7, 133.7, 132.6, 132.6, 132.4, 132.2, 132.1, 130.7, 130.2, 129.3, 128.2, 128.0, 127.6, 127.4, 127.1, 126.7, 126.3, 123.6, 122.4, 121.8, 121.5, 120.5, 118.9, 118.2. HRMS (ESI) m/z calcd for$C_{33}H_{21}Br_2O_2$+ $(M+H)^+$ 608.98852, found 608.98829.

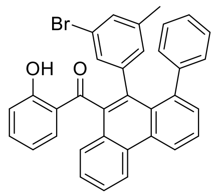

(10-(3-bromo-5-methylphenyl)-1-phenylphenanthren-9-yl)(2-hydroxyphenyl)methanone (44):

Yield 92%; 49.8 mg; white solid; mp 182–185°C; TLC (PET:EtOAc, 100:1 v/v): R_f = 0.3; ^{1}H NMR (600 MHz, $CDCl_3$) δ=12.12 (s, 1H), 8.91 (t, *J* = 8.4 Hz, 2H), 7.87 (dd, *J* = 13.2, 8.4 Hz, 1H), 7.77 (dd, *J* =16.8, 8.4 Hz, 2H), 7.61 (dd, *J* = 12.6, 6.6 Hz, 1H), 7.56 (d, *J* = 7.2 Hz, 1H), 7.30 (dd, *J* = 16.8, 8.4 Hz, 1H), 7.26–7.00 (m, 5H), 6.98–6.87 (m, 3H), 6.75 (d, *J* = 12.0 Hz, 1H), 6.66 (s, 1H), 6.57–6.48 (m, 1H), 6.40 (s, 1H), 2.14 (s, 1.7H), 1.87 (s, 1.3H). ^{13}C NMR (150 MHz, $CDCl_3$) δ=205.0, 204.9, 204.8, 162.5, 162.4, 143.1, 143.1, 142.7, 142.6, 140.8, 140.6, 138.5, 138.0, 136.8, 136.6, 136.3, 136.3, 134.3, 133.0, 132.9, 132.5, 132.4, 132.3, 132.2, 132.1, 131.7, 131.0, 130.8, 130.7, 130.6, 130.2, 130.1, 130.1, 129.3, 129.1, 128.5, 128.5, 128.3, 127.9, 127.5, 127.0, 127.0, 126.5, 126.3, 126.2, 126.2, 123.6, 122.4, 122.3, 121.4, 121.1, 121.0, 120.6, 118.8, 118.6, 118.0, 117.9, 20.8, 20.6. HRMS (ESI) m/z calcd for $C_{34}H_{24}BrO_2$+ $(M+H)^+$ 543.09542, found 543.09544.

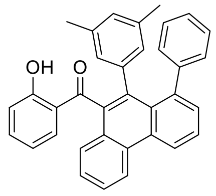

(10-(3,5-dimethylphenyl)-1-phenylphenanthren-9-yl)(2-hydroxyphenyl)methanone (45):

Yield 89%; 42.5 mg; white solid; mp 184–188°C; TLC (PET:EtOAc, 100:1 v/v): R_f = 0.3; ^{1}H NMR (600 MHz, $CDCl_3$) δ=12.15 (s, 1H), 8.98–8.88 (m, 2H), 7.87 (d, *J* = 8.4 Hz, 1H), 7.77 (dt, *J* = 15.0, 7.8 Hz, 2H), 7.63–7.55 (m, 2H), 7.28 (t, *J* = 7.8 Hz, 1H), 7.21–7.09 (m, 2H), 7.06–6.97 (m, 3H), 6.97–6.90 (m, 2H), 6.84 (s, 1H), 6.51 (t, *J* = 7.8 Hz, 1H), 6.39 (s, 1H), 6.28 (s, 1H), 2.17 (s, 3H), 1.90 (s, 3H). ^{13}C NMR (150 MHz, $CDCl_3$) δ=205.3, 162.1, 143.2, 142.9, 138.5, 136.1, 135.7, 133.0, 132.4, 131.9, 130.6, 130.2, 129.8, 128.9, 128.8, 128.3, 127.9, 127.5, 127.2, 126.7,

126.6, 126.3, 125.9, 125.6, 123.4, 122.0, 120.8, 118.2, 117.5, 20.9, 20.6. HRMS (ESI) m/z calcd for $C_{35}H_{27}O_2$+ $(M+H)^+$ 479.20056, found 479.20082.

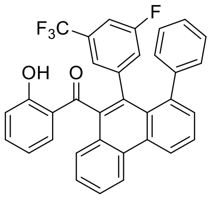

(10-(3-fluoro-5-(trifluoromethyl)phenyl)-1-phenylphenanthren-9-yl)(2-hydroxyphenyl)methanone (46):

Yield 74%; 39.6 mg; white solid; mp 194–197°C; TLC (PET:EtOAc, 100:1 v/v): R_f = 0.3; ^{1}H NMR (600 MHz, $CDCl_3$) δ=11.73 (d, *J* = 8.4 Hz, 1H), 8.83 (dd, *J* = 15.6, 8.4 Hz, 2H), 7.83–7.59 (m, 3H), 7.53 (t, *J*= 6.6 Hz, 1H), 7.45 (t, *J* = 7.8 Hz, 1H), 7.20 (dd, *J* = 15.6, 7.8 Hz, 2H), 7.05 (dd, *J* = 18.6, 7.2 Hz, 1H), 7.00–6.85 (m, 4H), 6.86–6.68 (m, 3H), 6.67–6.58 (m, 2H), 6.41 (dd, *J* = 18.6, 10.2 Hz, 2H). ^{13}C NMR (150 MHz, $CDCl_3$) δ=204.4, 204.4, 162.4, 162.3, 161.8, 161.5, 160.1, 159.8, 142.8, 142.5, 142.4, 142.2, 142.1, 137.0, 137.0, 136.8, 136.7, 132.8, 132.6, 132.5, 132.4, 132.4, 132.2, 132.2, 130.8, 130.7, 130.2, 129.4, 129.0, 128.1, 128.0, 128.0, 127.9, 127.7, 127.1, 127.0, 126.6, 126.6, 126.2, 125.0, 123.8, 123.5, 122.5, 122.4, 121.1, 120.9, 120.4, 118.8, 118.8, 118.1, 118.0, 110.8. HRMS (ESI) m/z calcd for $C_{34}H_{21}F_4O_2$+ $(M+H)^+$ 537.14722, found 537.14720.

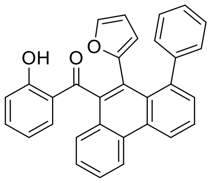

(10-(furan-2-yl)-1-phenylphenanthren-9-yl)(2-hydroxyphenyl)methanone (47):

Yield 91%; 40.0 mg; yellow solid; mp 195–199°C; TLC (PET:EtOAc, 100:1 v/v): R_f = 0.3; ^{1}H NMR (600 MHz, $CDCl_3$) δ=12.11 (s, 1H), 8.88 (d, *J* = 7.2 Hz, 2H), 7.89–7.72 (m, 3H), 7.61 (dd, *J* = 18.0, 7.2 Hz, 2H), 7.43 (s, 1H), 7.35–7.28 (m, 1H), 7.22 (s, 1H), 7.14–6.99 (m, 3H), 6.93 (d, *J* = 8.4 Hz, 1H), 6.79 (d, *J* = 11.4 Hz, 2H), 6.49 (t, *J* = 7.2 Hz, 1H), 5.95 (s, 1H), 5.72 (s, 1H). ^{13}C NMR (150 MHz, $CDCl_3$) δ=204.4, 162.4, 149.3, 142.4, 142.2, 142.0, 136.8, 136.5, 132.5, 132.4, 132.0, 130.6, 130.3, 128.1, 128.0, 127.9, 127.9, 127.7, 127.5, 127.1, 126.7, 124.9, 123.5, 122.0, 120.3, 118.6, 117.7, 113.7, 110.8. HRMS (ESI) m/z calcd for $C_{31}H_{21}O_3$+ $(M+H)^+$ 441.14852, found 441.14867.

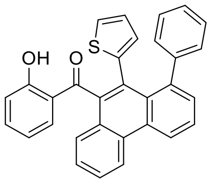

(2-hydroxyphenyl)(1-phenyl-10-(thiophen-2-yl)phenanthren-9-yl)methanone (48):

Yield 74%; 33.7 mg; yellow solid; mp 223–226℃; TLC (PET:EtOAc, 100:1 v/v): R_f = 0.3; ^{1}H NMR (600 MHz, $CDCl_3$) δ=12.00 (s, 1H), 8.86 (d, *J* = 7.8 Hz, 2H), 7.76 (ddd, *J* = 23.4, 15.6, 7.8 Hz, 3H), 7.61–7.50 (m, 2H), 7.42 (dd, *J* = 23.4, 7.8 Hz, 1H), 7.35–7.24 (m, 2H), 7.11 (s, 2H), 7.07–6.95 (m, 3H), 6.83 (d, *J* = 57.0Hz, 3H), 6.51 (dd, *J* = 24.6, 17.4 Hz, 1H). ^{13}C NMR (150 MHz, $CDCl_3$) δ=204.5, 162.1, 142.6, 141.8, 141.2, 137.2, 136.4, 135.8, 133.0, 132.2, 132.0, 131.6, 131.0, 130.0, 129.5, 129.0, 128.0, 127.8, 127.7, 127.5, 127.1, 126.8, 126.5, 126.0, 125.7, 123.3, 121.6, 118.4, 117.5. HRMS (ESI) m/z calcd for $C_{31}H_{21}O_2S+$ $(M+H)^+$ 457.12568, found 457.12701.

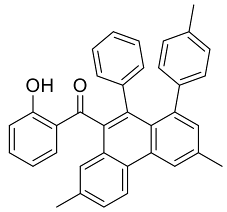

(3,7-dimethyl-10-phenyl-1-(p-tolyl)phenanthren-9-yl)(2-hydroxyphenyl)methanone (49):

Yield 75%; 36.9 mg; yellow solid; mp 270–273℃; TLC (PET:EtOAc, 100:1 v/v): R_f = 0.3; ^{1}H NMR (600 MHz, $CDCl_3$) δ=11.92 (s, 1H), 8.71 (d, *J* = 8.4 Hz, 1H), 8.60 (s, 1H), 7.50 (d, *J* = 8.4 Hz, 1H), 7.41 (s, 1H), 7.18 (d, *J* = 7.2 Hz, 1H), 7.03 (d, *J* = 7.2 Hz, 1H), 6.90 (d, *J* = 7.2 Hz, 1H), 6.77 (ddd, *J* = 38.4, 15.6, 7.2 Hz, 5H), 6.68 (s, 1H), 6.63 (d, *J* = 10.2 Hz, 2H), 6.49 (s, 2H), 6.41 (t, *J* = 7.2 Hz, 1H), 2.64 (s, 3H), 2.44 (s, 3H), 2.13 (s, 3H). ^{13}C NMR (150 MHz, $CDCl_3$) δ=205.7, 162.0, 142.6, 140.4, 139.2, 137.3, 136.3, 136.2, 135.9, 135.1, 134.8, 133.2, 133.1, 132.6, 132.0, 130.8, 128.9, 128.7, 127.9, 127.5, 126.7, 126.5, 126.0, 125.2, 123.2, 121.5, 120.7, 118.4, 117.5, 21.7, 21.6, 20.9. HRMS (ESI) m/z calcd for $C_{36}H_{29}O_2^+$ $(M+H)^+$ 493.21621, found 493.21649.

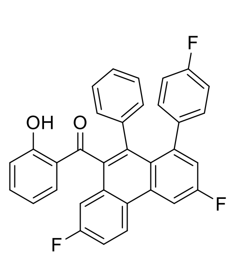

(3,7-difluoro-1-(4-fluorophenyl)-10-phenylphenanthren-9-yl)(2-hydroxyphenyl) methanone (50):

Yield 93%; 46.9 mg; white solid; mp 213–216℃; TLC (PET:EtOAc, 100:1 v/v): R_f = 0.3; ^{1}H NMR (400 MHz, $CDCl_3$) δ=11.77 (s, 1H), 8.67 (dd, *J* = 9.2, 5.2 Hz, 1H), 8.39 (dd, *J* = 10.4, 2.0 Hz, 1H), 7.43 (dd,*J* = 12.0, 5.2 Hz, 1H), 7.31 (dd, *J* = 10.0, 2.0 Hz, 1H), 7.24–7.17 (m, 2H), 7.03 (d, *J* = 7.6 Hz, 1H), 6.99–6.93 (m, 1H), 6.89 (t, *J* = 7.6 Hz, 1H), 6.80–6.74 (m, 3H), 6.71 (d, *J* = 5.6 Hz, 1H), 6.68–6.58 (m, 2H),

6.53 (dd, J = 15.6, 4.8Hz, 2H), 6.45 (t, J = 7.6 Hz, 1H). ^{13}C NMR (100 MHz, $CDCl_3$) δ=204.1, 163.3, 162.5, 162.3, 162.1, 160.8, 160.0, 159.6, 144.7, 144.7, 138.4, 138.0, 136.7, 134.9, 134.2, 134.1, 132.7, 132.1, 132.0, 131.7, 130.6, 130.5, 130.4, 130.0, 129.9, 127.2, 127.0, 126.9, 126.1, 126.0, 125.9, 125.4, 120.5, 120.2, 118.5, 117.8, 116.7, 116.5, 114.5, 114.3, 114.1, 113.9, 110.7, 110.5, 107.3, 107.1. ^{19}F NMR (375 MHz, $CDCl_3$) δ=111.16, 112.64, 116.04. HRMS (ESI) m/z calcd for $C_{33}H_{20}F_3O_2$+ $(M+H)^+$ 505.14099, found 505.14107.

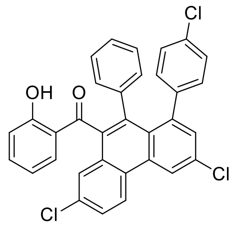

(3,7-dichloro-1-(4-chlorophenyl)-10-phenylphenanthren-9-yl)(2-hydroxyphenyl) methanone (51):

Yield 94%; 51.8 mg; white solid; mp 275–278°C; TLC (PET:EtOAc, 100:1 v/v): R_f = 0.3; ^{1}H NMR (400 MHz, CD_2Cl_2) δ=11.71 (s, 1H), 8.82 (s, 1H), 8.73 (d, J = 8.9 Hz, 1H), 7.72 (d, J = 9.0 Hz, 1H), 7.64 (s, 1H), 7.44 (s, 1H), 7.27 (t, J = 7.8 Hz, 1H), 7.02 (d, J = 7.1 Hz, 1H), 6.98–6.90 (m, 3H), 6.83 (t, J = 7.5 Hz, 2H), 6.78 (d, J = 8.2 Hz, 2H), 6.71 (d, J = 8.1 Hz, 1H), 6.62 (t, J = 7.5 Hz, 1H), 6.50 (dd, J = 14.6, 7.4 Hz, 2H). ^{13}C NMR (100 MHz, CD_2Cl_2) δ=204.2, 162.8, 143.9, 140.7, 138.6, 137.2, 137.0, 135.8, 134.7, 133.8, 133.5, 133.2, 132.7, 132.4, 132.4, 131.3, 130.8, 130.1, 128.7, 128.1, 127.9, 127.7, 127.3, 125.7, 125.3, 122.3, 120.7, 119.1, 118.1. HRMS (ESI) m/z calcd for $C_{33}H_{20}Cl_3O_2$+ $(M+H)^+$ 553.05234, found 553.05238.

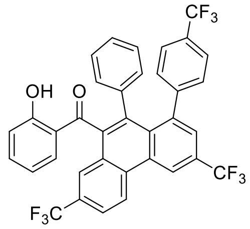

(2-hydroxyphenyl)(10-phenyl-3,7-bis(trifluoromethyl)-1-(4-(trifluoromethyl)phenyl)phenanthren-9-yl)methanone (52):

Yield 91%; 59.5 mg; yellow solid; mp 245–249°C; TLC (PET:EtOAc, 100:1 v/v): R_f = 0.3; ^{1}H NMR (600 MHz, $CDCl_3$) δ=11.63 (d, J = 1.8 Hz, 1H), 9.19 (s, 1H), 9.02 (d, J = 8.4 Hz, 1H), 8.04–7.96 (m, 2H), 7.71 (s, 1H), 7.26 (d, J = 5.4 Hz, 2H), 7.19–7.12 (m, 2H), 7.01 (d, J = 7.2 Hz, 1H), 6.88 (d, J = 6.6 Hz, 2H), 6.79 (dd, J = 13.8, 7.2 Hz, 2H), 6.71 (d, J = 7.8 Hz, 1H), 6.60 (t, J = 7.2 Hz, 1H), 6.48 (dd, J = 15.6, 7.2 Hz, 2H). ^{13}C NMR (150 MHz, $CDCl_3$) δ=203.0, 162.3, 145.2, 142.7, 138.1, 137.3, 137.1, 136.4, 132.4, 131.9, 131.6, 131.6, 131.5, 130.9, 130.7, 130.5, 130.3, 129.3, 129.0, 128.8, 128.7, 128.5, 128.2, 128.1, 127.5, 127.3, 124.6, 124.2, 124.1, 123.4, 120.4, 120.1, 118.7, 118.1. ^{19}F NMR (375 MHz, $CDCl_3$) δ=62.18, 62.30, 63.03. HRMS (ESI) m/z calcd for $C_{36}H_{20}F_9O_2$+ $(M+H)^+$ 655.13141, found 655.13131.

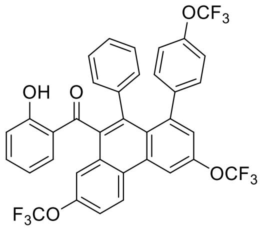

(2-hydroxyphenyl)(10-phenyl-3,7-bis(trifluoromethoxy)-1-(4-(trifluoromethoxy)phenyl)phenanthren-9-yl)methanone (53):

Yield 91%; 63.8 mg; white solid; mp 163–166℃; TLC (PET:EtOAc, 100:1 v/v): R_f = 0.3; ^{1}H NMR (600 MHz, $CDCl_3$) δ=11.78 (s, 1H), 8.85 (d, *J* = 9.0 Hz, 1H), 8.71 (s, 1H), 7.68 (d, *J* = 9.0 Hz, 1H), 7.58 (s, 1H), 7.42 (s, 1H), 7.29 (t, *J* = 7.8 Hz, 1H), 7.11 (t, *J* = 7.2 Hz, 2H), 6.95 (t, *J* = 7.2 Hz, 1H), 6.90 (s, 1H), 6.85 (d, *J* =4.2 Hz, 4H), 6.79 (d, *J* = 7.8 Hz, 1H), 6.68 (t, *J* = 7.2 Hz, 1H), 6.60 (d, *J* = 7.2 Hz, 1H), 6.53 (t, *J* = 7.2 Hz, 1H). ^{13}C NMR (150 MHz, $CDCl_3$) δ=203.5, 162.4, 148.8, 147.6, 147.6, 144.0, 140.5, 137.9, 136.9, 136.6, 136.1, 133.3, 132.6, 131.9, 131.8, 130.6, 130.3, 129.6, 127.7, 127.5, 127.4, 127.2, 127.2, 125.7, 124.9, 121.2, 120.8, 120.4, 120.1, 119.9, 119.8, 119.5, 118.7, 117.9, 117.2, 113.8. ^{19}F NMR (375 MHz, $CDCl_3$) δ=57.33, 57.62, 57.73. HRMS (ESI) m/z calcd for $C_{36}H_{20}F_9O_5$+ $(M+H)^+$ 703.11615, found 703.11598.

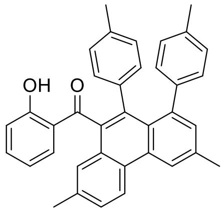

(10-(4-ethoxyphe (54):

Yield 66%; 33.4 mg; white solid; mp 248–251℃; TLC (PET:EtOAc, 100:1 v/v): R_f = 0.3; ^{1}H NMR (400 MHz, $CDCl_3$) δ=11.98 (s, 1H), 8.72 (d, *J* = 8.4 Hz, 1H), 8.61 (s, 1H), 7.51 (d, *J* = 8.8 Hz, 1H), 7.39 (s, 1H), 7.25 (d, *J* = 11.6 Hz, 2H), 6.88 (dd, *J* = 12.8, 8.0 Hz, 3H), 6.81–6.73 (m, 2H), 6.62 (s, 3H), 6.45 (s, 1H), 6.35 (s, 1H), 6.31 (s, 1H), 2.65 (s, 3H), 2.45 (s, 3H), 2.18 (s, 3H), 2.02 (s, 3H). ^{13}C NMR (100 MHz, $CDCl_3$) δ=205.8, 162.1, 142.7, 140.6, 137.3, 136.2, 136.1, 136.0, 135.7, 134.9, 134.6, 133.3, 133.2, 132.6, 132.0, 130.8, 130.6, 128.9, 128.8, 128.6, 127.6, 127.4, 126.7, 125.2, 123.2, 121.6, 120.9, 118.4, 117.5, 21.8, 21.6, 20.9. HRMS (ESI) m/z calcd for $C_{37}H_{30}O_2$+ $(M)^+$ 506.22401, found 506.22454.

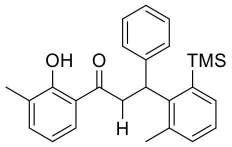

1-(2-hydroxy-3-methylphenyl)-3-(2-methyl-6-(trimethylsilyl)phenyl)-3-phenylpropan-1-one (55):

Yield 94%; 37.8 mg; white solid; mp 159–161°C; TLC (PET:EtOAc, 100:1 v/v): R_f = 0.3; ^{1}H NMR (600 MHz, $CDCl_3$) δ=12.64 (s, 1H), 7.72 (d, *J* = 7.8 Hz, 1H), 7.46 (d, *J* = 7.2 Hz, 1H), 7.31 (d, *J* = 7.2 Hz, 1H), 7.17 (dd, *J* = 17.4, 7.8 Hz, 3H), 7.10 (dd, *J* = 12.6, 6.6 Hz, 2H), 6.96 (d, *J* = 7.8 Hz, 2H), 6.78 (t, *J* = 7.8 Hz, 1H), 5.42 (d, *J* = 10.2 Hz, 1H), 4.40 (dd, *J* = 18.6, 10.2 Hz, 1H), 3.37 (d, *J* = 18.6 Hz, 1H), 2.26 (s, 3H), 1.92 (s, 3H), 0.36 (s, 9H). ^{13}C NMR (150 MHz, $CDCl_3$) δ=204.2, 161.3, 148.8, 143.0, 140.6, 137.4, 137.1, 134.0, 132.9, 128.3, 128.0, 127.3, 127.2, 126.6, 125.9, 118.7, 118.5, 43.4, 42.0, 21.6, 15.8, 0.8. HRMS (ESI) m/z calcd for $C_{26}H_{30}O_2SiNa^+$ $(M+Na)^+$ 425.19073, found 425.19081.

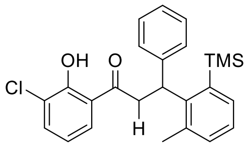

1-(3-chloro-2-hydroxyphenyl)-3-(2-methyl-6-(trimethylsilyl)phenyl)-3-phenylpropan-1-one (56):

Yield 66%; 27.8 mg; yellow solid; mp 195–198°C; TLC (PET:EtOAc, 100:1 v/v): R_f = 0.3; ^{1}H NMR (600 MHz, $CDCl_3$) δ=12.90 (s, 1H), 7.86 (d, *J* = 7.8 Hz, 1H), 7.63 (d, *J* = 7.8 Hz, 1H), 7.49 (d, *J* = 7.2 Hz, 1H), 7.24 (t, *J* = 7.2 Hz, 3H), 7.18 (t, *J* = 8.4 Hz, 2H), 6.99–6.89 (m, 3H), 5.41 (d, *J* = 10.2 Hz, 1H), 4.41 (dd, *J* =19.2, 10.2 Hz, 1H), 3.44 (d, *J* = 19.2 Hz, 1H), 1.95 (s, 3H), 0.38 (s, 9H). ^{13}C NMR (150 MHz, $CDCl_3$) δ=203.9, 158.3, 148.3, 142.5, 140.6, 136.9, 136.5, 133.9, 132.8, 128.3, 128.0, 127.1, 126.5, 125.8, 123.3, 120.2, 119.2, 43.2, 42.0, 21.4, 0.6. HRMS (ESI) m/z calcd for $C_{25}H_{27}ClO_2SiNa^+$ $(M+Na)^+$ 445.13611, found 445.13620.

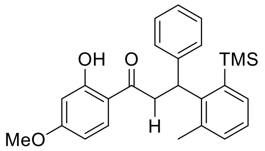

1-(2-hydroxy-4-methoxyphenyl)-3-(2-methyl-6-(trimethylsilyl)phenyl)-3-phenylpropan-1-one (57):

Yield 96%; 40.1 mg; white solid; mp 206–209°C; TLC (PET:EtOAc, 50:1 v/v): R_f = 0.3; ^{1}H NMR (600 MHz, $CDCl_3$) δ=12.81 (s, 1H), 7.77 (d, *J* = 9.6 Hz, 1H), 7.45 (d, *J* = 7.2 Hz, 1H), 7.18 (td, *J* = 7.8, 3.6Hz, 3H), 7.14–7.09 (m, 2H), 6.96 (d, *J* = 7.8 Hz, 2H), 6.48–6.42 (m, 2H), 5.39 (d, *J* = 10.2 Hz, 1H), 4.30 (dd, *J* =18.6, 10.2 Hz, 1H), 3.78 (s, 3H), 3.29 (d, *J* = 18.6 Hz, 1H), 1.92 (s, 3H), 0.35 (s, 9H). ^{13}C NMR (150 MHz, $CDCl_3$) δ=201.9, 166.2, 165.6, 148.8, 143.1, 140.6, 137.0, 133.9, 132.8, 131.2, 128.2, 127.2, 126.5, 125.7, 113.5, 107.9, 101.2, 55.6, 43.3, 41.4, 21.5, 0.7. HRMS (ESI) m/z calcd for $C_{26}H_{31}O_3Si^+$ $(M+H)^+$ 419.20370, found 419.20380.

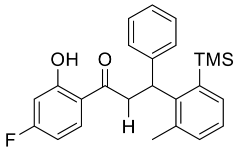

1-(4-fluoro-2-hydroxyphenyl)-3-(2-methyl-6-(trimethylsilyl)phenyl)-3-phenylpropan-1-one (58):

Yield 87%; 35.3 mg; yellow solid; mp 137–140℃; TLC (PET:EtOAc, 100:1 v/v): R_f = 0.3; ^{1}H NMR (600 MHz, $CDCl_3$) δ=12.66 (s, 1H), 7.88–7.82 (m, 1H), 7.47 (d, *J* = 7.2 Hz, 1H), 7.17 (dt, *J* = 15.0, 7.8 Hz, 3H), 7.13–7.07 (m, 2H), 6.97 (d, *J* = 7.8 Hz, 2H), 6.63 (d, *J* = 10.2 Hz, 1H), 6.56 (t, *J* = 7.2 Hz, 1H), 5.43 (d, *J* =10.2 Hz, 1H), 4.33 (dd, *J* = 18.6, 10.2 Hz, 1H), 3.36 (d, *J* = 18.6 Hz, 1H), 1.94 (s, 3H), 0.36 (s, 9H). ^{13}C NMR (150 MHz, $CDCl_3$) δ=203.0, 168.5, 166.8, 165.5, 165.4, 148.6, 143.0, 140.7, 137.1, 134.1, 133.0, 132.2, 132.1, 128.5, 127.3, 126.8, 126.0, 116.7, 107.6, 107.5, 105.5, 105.4, 43.4, 42.1, 21.7, 0.9. ^{19}F NMR (375 MHz, $CDCl_3$) δ=98.92. HRMS (ESI) m/z calcd for $C_{25}H_{27}FO_2SiNa$+ $(M+Na)^+$ 429.165656, found 429.165763.

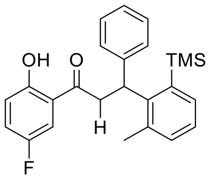

1-(5-fluoro-2-hydroxyphenyl)-3-(2-methyl-6-(trimethylsilyl)phenyl)-3-phenylpropan-1-one (59):

Yield 65%; 26.4 mg; yellow solid; mp 149–152℃; TLC (PET:EtOAc, 100:1 v/v): R_f = 0.3; ^{1}H NMR (600 MHz, $CDCl_3$) δ=12.05 (s, 1H), 7.55 (d, *J* = 8.4 Hz, 1H), 7.48 (d, *J* = 7.2 Hz, 1H), 7.16 (ddd, *J* = 22.8, 13.8, 6.6 Hz, 6H), 6.96 (d, *J* = 7.2 Hz, 3H), 5.41 (d, *J* = 10.2 Hz, 1H), 4.33 (dd, *J* = 18.6, 10.2 Hz, 1H), 3.36 (d, *J* =18.6 Hz, 1H), 1.94 (s, 3H), 0.37 (s, 9H). ^{13}C NMR (150 MHz, $CDCl_3$) δ=203.3, 159.0, 155.7, 154.1, 148.4, 142.7, 140.6, 137.0, 134.0, 132.9, 128.4, 127.2, 126.7, 125.9, 124.4, 124.2, 120.3, 118.8, 114.7, 114.5, 43.3, 42.1, 21.6, 0.8. ^{19}F NMR (375 MHz, $CDCl_3$) δ=123.47. HRMS (ESI) m/z calcd for $C_{25}H_{27}FO_2SiNa$+ $(M+Na)^+$ 429.165656, found 429.165755.

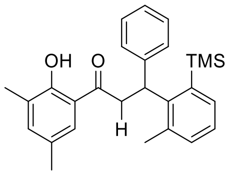

1-(2-hydroxy-3,5-dimethylphenyl)-3-(2-methyl-6-(trimethylsilyl)phenyl)-3-phenylpropan-1-one (60):

Yield 89%; 37.0 mg; yellow oil; TLC (PET:EtOAc, 100:1 v/v): R_f = 0.3; ^{1}H NMR (600 MHz, $CDCl_3$) δ=12.34 (s, 1H), 7.38 (s, 1H), 7.31 (d, *J* = 7.2 Hz, 1H), 7.01 (t, *J* = 7.8 Hz, 4H), 6.98–6.94 (m, 2H), 6.82 (d, *J* = 7.8 Hz, 2H), 5.26 (d, *J* = 10.2 Hz, 1H), 4.25 (dd, *J* = 18.6, 10.2 Hz, 1H), 3.24 (d, *J* = 18.6 Hz, 1H), 2.10 (d, *J* = 11.4 Hz, 6H), 1.78 (s, 3H), 0.21 (s, 9H). ^{13}C NMR (150 MHz, $CDCl_3$) δ=204.0, 159.3, 148.9, 143.1, 140.6, 138.7, 137.1, 134.0, 132.9, 128.3, 127.7, 127.4, 127.4, 126.9, 126.6,

125.8, 118.4, 43.5, 42.0, 21.7, 20.8, 15.8, 0.8. HRMS (ESI) m/z calcd for $C_{27}H_{33}O_2Si+$ $(M+H)^+$ 417.22443, found 417.22454.

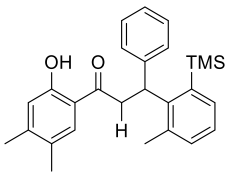

1-(2-hydroxy-4,5-dimethylphenyl)-3-(2-methyl-6-(trimethylsilyl)phenyl)-3-phenyl propan-1-one (61):

Yield 95%; 39.5 mg; yellow solid; mp 230–233°C; TLC (PET:EtOAc, 100:1 v/v): R_f = 0.3; ^{1}H NMR (600 MHz, $CDCl_3$) δ=12.27 (d, *J* = 1.2 Hz, 1H), 7.70 (s, 1H), 7.56 (d, *J* = 7.2 Hz, 1H), 7.29 (dd, *J* = 13.8, 7.2Hz, 3H), 7.25–7.22 (m, 2H), 7.07 (d, *J* = 7.2 Hz, 2H), 6.92 (s, 1H), 5.50 (d, *J* = 10.2 Hz, 1H), 4.46 (dd, *J* = 18.6, 10.8Hz, 1H), 3.47 (d, *J* = 18.6 Hz, 1H), 2.35 (s, 3H), 2.31 (s, 3H), 2.04 (s, 3H), 0.46 (s, 9H). ^{13}C NMR (150 MHz, $CDCl_3$) δ=203.2, 161.0, 148.9, 147.2, 143.0, 140.6, 137.0, 133.9, 132.8, 129.7, 128.2, 127.4, 127.3, 126.5, 125.8, 119.3, 117.4, 43.4, 41.7, 21.6, 20.6, 19.1, 0.7. HRMS (ESI) m/z calcd for $C_{27}H_{33}O_2Si+$ $(M+H)^+$ 417.22443, found 417.22454.

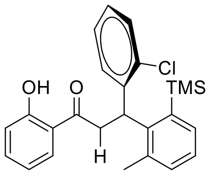

1-(2-hydroxyphenyl)-3-(2-methyl-6-(trimethylsilyl)phenyl)-3-(o-tolyl)propan-1-one (62):

Yield 92%; 38.8 mg; yellow oil; TLC (PET:EtOAc, 100:1 v/v): R_f = 0.3; ^{1}H NMR (600 MHz, $CDCl_3$) δ=12.20 (s, 1H), 7.79 (d, *J* = 7.8 Hz, 1H), 7.47 (d, *J* = 7.2 Hz, 1H), 7.38 (t, *J* = 7.8 Hz, 1H), 7.31 (d, *J* = 7.8 Hz, 1H), 7.15 (t, *J* = 7.2 Hz, 1H), 7.07 (dd, *J* = 16.8, 7.2 Hz, 3H), 6.99 (d, *J* = 7.2 Hz, 1H), 6.92 (d, *J* = 8.4 Hz, 1H), 6.82 (t, *J* = 7.2 Hz, 1H), 5.43 (t, *J* = 6.6 Hz, 1H), 4.23 (dd, *J* = 18.6, 7.2 Hz, 1H), 3.70 (dd, *J* =18.6, 5.4 Hz, 1H), 2.10 (s, 3H), 0.30 (s, 9H). ^{13}C NMR (150 MHz, $CDCl_3$) δ=203.5, 162.6, 146.0, 141.4, 140.0, 136.5, 136.0, 134.1, 134.0, 133.7, 130.6, 130.0, 129.5, 127.9, 126.7, 126.4, 119.3, 119.1, 118.8, 43.8, 42.1, 23.2, 1.4. HRMS (ESI) m/z calcd for $C_{25}H_{28}ClO_2Si+$ $(M+H)^+$ 423.15416, found 423.15448.

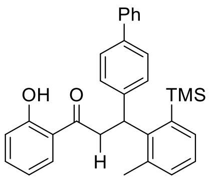

3-([1,1'-biphenyl]-4-yl)-1-(2-hydroxyphenyl)-3-(2-methyl-6-(trimethylsilyl)phenyl)propan-1-one (63):

Yield 88%; 40.8 mg; yellow solid; mp 123–124°C; TLC (PET:EtOAc, 100:1 v/v): R_f = 0.3; ^{1}H NMR (600 MHz, $CDCl_3$) δ=12.30 (s, 1H), 7.90 (d, *J* = 7.8 Hz, 1H), 7.53 (dd, *J* = 16.2, 7.8 Hz, 3H), 7.46 (dd, *J* =15.6, 7.8 Hz, 3H), 7.39 (t, *J* = 7.8 Hz, 2H), 7.30 (d, *J* = 7.2 Hz, 1H), 7.21 (d, *J* = 7.2 Hz, 1H), 7.16 (d, *J* = 7.2 Hz, 1H), 7.05 (d, *J* = 8.4 Hz, 1H), 7.01 (d, *J* = 7.8 Hz, 2H), 6.94 (s, 1H), 5.41 (d, *J* = 10.2 Hz, 1H), 4.41 (dd, *J* = 18.6, 10.8 Hz, 1H), 3.40 (d, *J* = 18.6 Hz, 1H), 1.98 (s, 3H), 0.36 (s, 9H). ^{13}C NMR (150 MHz, $CDCl_3$) δ=204.9, 163.6, 149.5, 142.9, 141.7, 141.6, 139.5, 138.0, 137.6, 134.9, 133.8, 130.6, 129.7, 128.6, 128.1, 127.9, 127.9, 127.5, 120.3, 120.1, 119.8, 44.0, 42.8, 22.6, 1.6. HRMS (ESI) m/z calcd for $C_{31}H_{32}O_2SiNa+$ $(M+Na)^+$ 487.20638, found 487.20597.

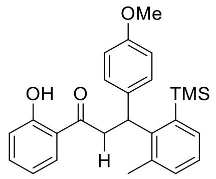

1-(2-hydroxyphenyl)-3-(4-methoxyphenyl)-3-(2-methyl-6-(trimethylsilyl)phenyl)propan-1-one (64):

Yield 90%; 37.6 mg; yellow solid; mp 155–158°C; TLC (PET:EtOAc, 50:1 v/v): R_f = 0.3; ^{1}H NMR (600 MHz, $CDCl_3$) δ=12.35 (s, 1H), 7.86 (d, *J* = 7.8 Hz, 1H), 7.47–7.38 (m, 2H), 7.19–7.09 (m, 2H), 6.97 (d,*J* = 8.4 Hz, 1H), 6.85 (t, *J* = 7.8 Hz, 3H), 6.72 (d, *J* = 8.4 Hz, 2H), 5.35 (d, *J* = 10.2 Hz, 1H), 4.36 (dd, *J* = 18.6, 10.2Hz, 1H), 3.64 (s, 3H), 3.36 (d, *J* = 18.6 Hz, 1H), 1.96 (s, 3H), 0.36 (s, 9H). ^{13}C NMR (150 MHz, $CDCl_3$) δ=204.2, 162.8, 157.7, 148.9, 140.5, 137.1, 136.7, 134.9, 134.1, 132.9, 129.7, 128.2, 126.6, 119.5, 119.2, 118.9, 113.8, 55.2, 42.7, 42.1, 21.6, 0.8. HRMS (ESI) m/z calcd for $C_{26}H_{30}O_3SiNa+$ $(M+Na)^+$ 441.18564, found 441.18574.

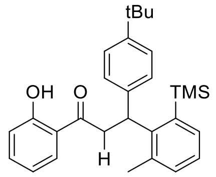

3-(4-(tert-butyl)phenyl)-1-(2-hydroxyphenyl)-3-(2-methyl-6-(trimethylsilyl)phenyl)propan-1-one (65):

Yield 96%; 42.6 mg; white solid; mp 160–163°C; TLC (PET:EtOAc, 100:1 v/v): R_f = 0.3; ^{1}H NMR (600 MHz, $CDCl_3$) δ=12.37 (s, 1H), 7.86 (d, *J* = 6.6 Hz, 1H), 7.46 (d, *J* = 6.0 Hz, 1H), 7.37 (d, *J* = 6.6 Hz, 1H), 7.17 (dd, *J* = 23.4, 7.2 Hz, 3H), 7.10 (d, *J* = 6.0 Hz, 1H), 6.97 (d, *J* = 7.8 Hz, 1H), 6.86 (dd, *J* = 34.2, 6.6 Hz, 3H), 5.40 (d, *J* = 9.6 Hz, 1H), 4.42 (dd, *J* = 18.0, 10.8 Hz, 1H), 3.33 (d, *J* = 18.6 Hz, 1H), 1.93 (s, 3H), 1.24 (s, 9H), 0.38 (s, 9H). ^{13}C NMR (150 MHz, $CDCl_3$) δ=204.2, 162.9, 149.0, 148.5, 140.5, 139.8, 137.1, 136.7, 134.1, 132.9, 129.7, 127.0, 126.6, 125.3, 119.5, 119.2, 119.0, 43.1, 41.9, 34.4, 31.6, 21.7, 0.9. HRMS (ESI) m/z calcd for $C_{29}H_{36}O_2SiNa+$ $(M+Na)^+$ 467.23768, found 467.23775.

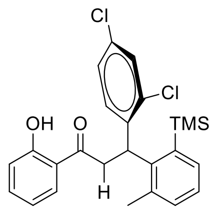

3-(2,4-dichlorophenyl)-1-(2-hydroxyphenyl)-3-(2-methyl-6-(trimethylsilyl)phenyl)propan-1-one (66):

Yield 62%; 28.3 mg; yellow oil; TLC (PET:EtOAc, 100:1 v/v): R_f = 0.3; ^{1}H NMR (600 MHz, $CDCl_3$) δ=12.31 (s, 1H), 8.00 (d, *J* = 7.2 Hz, 1H), 7.67 (s, 2H), 7.57 (s, 1H), 7.44 (s, 1H), 7.40–7.37 (m, 1H), 7.30 (d, *J* = 6.6 Hz, 2H), 7.18 (d, *J* = 7.8 Hz, 1H), 7.10 (d, *J* = 8.4 Hz, 1H), 5.55 (s, 1H), 4.38 (dd, *J* =18.6, 7.2 Hz, 1H), 3.86 (d, *J* = 19.2 Hz, 1H), 2.28 (s, 3H), 0.50 (s, 9H). ^{13}C NMR (150 MHz, $CDCl_3$) δ=203.0, 162.5, 145.5, 141.3, 138.7, 136.5, 135.7, 134.6, 133.9, 133.6, 132.7, 130.7, 130.2, 129.3, 126.7, 126.4, 119.0, 118.7, 43.2, 41.9, 23.0, 1.2. HRMS (ESI) m/z calcd for $C_{25}H_{27}Cl_2O_2Si+$ $(M+H)^+$ 457.11519, found 457.11529.

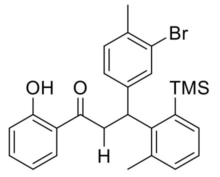

3-(3-bromo-4-methylphenyl)-1-(2-hydroxyphenyl)-3-(2-methyl-6-(trimethylsilyl)phenyl)propan-1-one (67):

Yield 93%; 44.6 mg; yellow oil; TLC (PET:EtOAc, 100:1 v/v): R_f = 0.3; ^{1}H NMR (600 MHz, $CDCl_3$) δ=12.43 (s, 1H), 8.00 (d, *J* = 7.8 Hz, 1H), 7.60 (dd, *J* = 18.6, 7.8 Hz, 2H), 7.34 (d, *J* = 7.2Hz, 1H), 7.33–7.28 (m, 2H), 7.19 (d, *J* = 7.8 Hz, 1H), 7.15 (d, *J* = 8.4 Hz, 1H), 7.03 (t, *J* = 7.2 Hz, 1H), 6.96 (d, *J* =7.8 Hz, 1H), 5.50 (d, *J* = 10.2 Hz, 1H), 4.48 (dd, *J* = 18.6, 10.2 Hz, 1H), 3.57 (d, *J* = 18.6 Hz, 1H), 2.45 (s, 3H), 2.13 (s, 3H), 0.51 (s, 9H). ^{13}C NMR (150 MHz, $CDCl_3$) δ=203.8, 162.7, 148.0, 142.6, 140.5, 136.8, 135.3, 134.1, 133.0, 131.2, 130.6, 129.7, 126.8, 126.2, 125.2, 119.3, 119.2, 118.9, 42.7, 42.0, 22.5, 21.7, 0.8. HRMS (ESI) m/z calcd for $C_{26}H_{29}BrO_2SiNa+$ $(M+Na)^+$ 503.10124, found 503.10131.

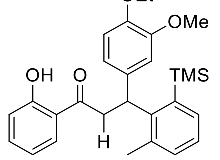

3-(4-ethoxy-3-methoxyphenyl)-1-(2-hydroxyphenyl)-3-(2-methyl-6-(trimethylsilyl)phenyl)propan-1-one (68):

Yield 87%; 40.2 mg; yellow oil; TLC (PET:EtOAc, 25:1 v/v): R_f = 0.3; ^{1}H NMR (600 MHz, $CDCl_3$) δ=12.31 (s, 1H), 7.90 (d, *J* = 7.8 Hz, 1H), 7.56–7.49 (m, 1H), 7.45 (d, *J* = 7.2 Hz, 1H), 7.23–7.17 (m, 1H), 7.15 (d, *J* = 7.2 Hz, 1H), 7.04 (d, *J* = 8.4 Hz, 1H), 6.97–6.91 (m, 1H), 6.72 (d, *J* = 8.4 Hz, 1H), 6.45 (s, 2H), 5.32 (d, *J* = 10.2 Hz,

1H), 4.37 (dd, J = 18.6, 10.8 Hz, 1H), 4.07–3.99 (m, 2H), 3.61–3.53 (m, 3H), 3.32 (d, J = 18.6 Hz, 1H), 1.99 (s, 3H), 1.42 (d, J = 7.2 Hz, 3H), 0.40–0.32 (m, 9H). ^{13}C NMR (150 MHz, $CDCl_3$) δ=203.9, 162.4, 148.8, 148.3, 146.2, 140.1, 136.9, 136.4, 135.0, 133.7, 132.5, 129.3, 126.2, 119.2, 118.9, 118.8, 118.6, 112.0, 111.5, 64.0, 55.6, 42.9, 41.5, 21.2, 14.7, 0.5. HRMS (ESI) m/z calcd for: $C_{28}H_{34}O_4SiNa+$ $(M+Na)^+$ 485.21186, found 485.21194.

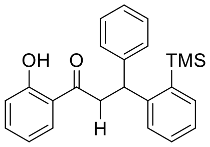

1-(2-hydroxyphenyl)-3-phenyl-3-(2-(trimethylsilyl)phenyl)propan-1-one (69):
Yield 92%; 34.4 mg; yellow solid; mp 120–123°C; TLC (PET:EtOAc, 100:1 v/v): R_f = 0.3; ^{1}H NMR (600 MHz, $CDCl_3$) δ=12.54 (s, 1H), 8.05 (d, J = 7.8 Hz, 1H), 7.83 (d, J = 7.2 Hz, 1H), 7.62 (t, J = 7.8 Hz, 1H), 7.53–7.50 (m, 1H), 7.44 (dt, J = 22.2, 7.2 Hz, 5H), 7.37 (t, J = 7.2 Hz, 2H), 7.19 (d, J = 8.4 Hz, 1H), 7.07 (t, J =7.8 Hz, 1H), 5.49 (dd, J = 9.6, 3.6 Hz, 1H), 4.25 (dd, J = 18.0, 9.6 Hz, 1H), 3.67 (dd, J = 18.0, 4.2 Hz, 1H), 0.66 (s, 9H). ^{13}C NMR (150 MHz, $CDCl_3$) δ=203.5, 162.4, 150.0, 143.4, 138.2, 136.3, 135.0, 129.5, 129.4, 128.3, 128.1, 127.6, 126.1, 125.8, 119.2, 118.8, 118.5, 44.9, 43.8, 0.5. HRMS (ESI) m/z calcd for $C_{24}H_{26}O_2SiNa+$ $(M+Na)^+$ 397.15943, found 397.15951.

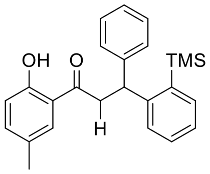

1-(2-hydroxy-5-methylphenyl)-3-phenyl-3-(2-(trimethylsilyl)phenyl)propan-1-on e (70):
Yield 51%; 19.8 mg; white solid; mp 179–182°C; TLC (PET:EtOAc, 100:1 v/v): R_f = 0.3; ^{1}H NMR (600 MHz, $CDCl_3$) δ=12.21 (s, 1H), 7.78 (s, 1H), 7.71 (d, J = 7.2 Hz, 1H), 7.51–7.33 (m, 5H), 7.30 (d, J =6.0 Hz, 3H), 7.26 (d, J = 7.8 Hz, 1H), 7.02 (d, J = 8.4 Hz, 1H), 5.35 (dd, J = 9.6, 3.6 Hz, 1H), 4.16 (dd, J = 18.0, 10.2 Hz, 1H), 3.56 (dd, J = 18.0, 4.2 Hz, 1H), 2.43 (s, 3H), 0.54 (s, 9H). ^{13}C NMR (150 MHz, $CDCl_3$) δ=203.7, 160.8, 150.6, 143.8, 138.6, 137.8, 135.4, 129.9, 129.7, 128.7, 128.6, 128.4, 128.1, 126.5, 126.2, 119.3, 118.7, 45.4, 44.2, 20.9, 0.9. HRMS (ESI) m/z calcd for $C_{25}H_{29}O_2Si+$ $(M+H)^+$ 389.19313, found 389.19322.

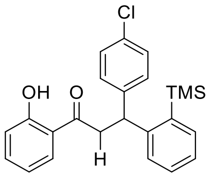

3-(4-chlorophenyl)-1-(2-hydroxyphenyl)-3-(2-(trimethylsilyl)phenyl)propan-1-one (71):

Yield 91%; 37.1 mg; yellow oil; TLC (PET:EtOAc, 100:1 v/v): R_f = 0.3; ^{1}H NMR (600 MHz, $CDCl_3$) δ=12.29 (s, 1H), 7.93 (d, *J* = 7.8 Hz, 1H), 7.67 (d, *J* = 7.2 Hz, 1H), 7.55 (t, *J* = 7.8 Hz, 1H), 7.42–7.38 (m, 1H), 7.32 (dd, *J* = 15.6, 7.8 Hz, 3H), 7.17 (t, *J* = 7.8 Hz, 3H), 7.07 (d, *J* = 8.4 Hz, 1H), 6.99 (d, *J*= 7.2 Hz, 1H), 5.31–5.24 (m, 1H), 4.07 (dd, *J* = 18.0, 10.2 Hz, 1H), 3.53 (dd, *J* = 18.0, 3.6 Hz, 1H), 0.49 (s, 9H). ^{13}C NMR (150 MHz, $CDCl_3$) δ=203.3, 162.5, 149.6, 142.1, 138.3, 136.6, 135.1, 132.0, 129.6, 129.5, 129.1, 128.5, 128.0, 126.0, 119.2, 119.0, 118.6, 44.9, 43.2, 0.5. HRMS (ESI) m/z calcd for $C_{24}H_{25}ClO_2SiNa+$ $(M+Na)^+$ 431.12046, found 431.12058.

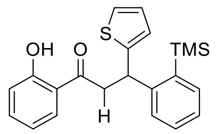

1-(2-hydroxyphenyl)-3-(thiophen-2-yl)-3-(2-(trimethylsilyl)phenyl)propan-1-one (72):

Yield 85%; 32.3 mg; yellow solid; mp 130–132°C; TLC (PET:EtOAc, 100:1 v/v): R_f = 0.3; ^{1}H NMR (600 MHz, $CDCl_3$) δ=12.26 (s, 1H), 7.79 (d, *J* = 7.8 Hz, 1H), 7.55 (d, *J* = 7.2 Hz, 1H), 7.41 (t, *J* = 7.8 Hz, 1H), 7.33 (s, 2H), 7.23 (s, 1H), 7.06 (d, *J* = 4.8 Hz, 1H), 6.95 (d, *J* = 8.4 Hz, 1H), 6.89–6.78 (m, 2H), 6.70 (d, *J* = 2.4Hz, 1H), 5.39–5.31 (m, 1H), 4.04 (dd, *J* = 18.0, 10.2 Hz, 1H), 3.39 (dd, *J* = 18.0, 3.0 Hz, 1H), 0.39 (s, 9H). ^{13}C NMR (150 MHz, $CDCl_3$) δ=203.2, 162.6, 149.5, 148.3, 138.1, 136.7, 135.0, 135.0, 129.7, 127.7, 126.6, 126.4, 124.4, 124.1, 119.4, 119.1, 118.7, 46.5, 40.2, 0.5. HRMS (ESI) m/z calcd for $C_{22}H_{24}O_2SSiNa+$ $(M+Na)^+$ 403.11585, found 403.11595.

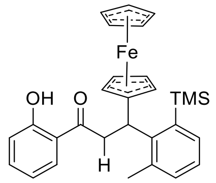

3-(ferroceneyl)-1-(2-hydroxyphenyl)-3-(2-methyl-6-(trimethylsilyl)phenyl)propan-1-one (73):

Yield 94%; 46.6 mg; brown solid; mp 195–198°C; TLC (PET:EtOAc, 100:1 v/v): R_f = 0.3; ^{1}H NMR (600 MHz, $CDCl_3$) δ=12.72 (s, 1H), 7.85 (d, *J* = 5.4 Hz, 1H), 7.48 (s, 1H), 7.36 (s, 1H), 7.05 (dd, *J* = 24.6, 18.0 Hz, 3H), 6.91 (s, 1H), 5.32 (d, *J* = 8.4 Hz, 1H), 4.21 (d, *J* = 12.0 Hz, 1H), 4.13 (s, 5H), 4.00 (s, 1H), 3.95 (s, 1H), 3.88 (s, 1H), 3.79 (s, 1H), 2.87 (d, *J* = 16.8 Hz, 1H), 1.91 (s, 3H), 0.53 (s, 9H). ^{13}C NMR (150 MHz, $CDCl_3$) δ=203.9, 163.1, 149.5, 139.0, 136.7, 134.0, 132.8, 129.8, 126.2, 119.7, 119.3, 119.0, 92.1, 69.5, 69.1, 67.9, 67.5, 65.6, 40.9, 40.7, 21.8, 1.1. HRMS (ESI) m/z calcd for $C_{29}H_{32}FeO_2Si+$ $(M)^+$ 496.15160, found 496.15278.

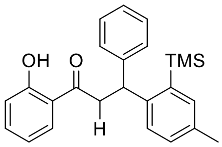

1-(2-hydroxyphenyl)-3-(4-methyl-2-(trimethylsilyl)phenyl)-3-phenylpropan-1-one (74):

Yield 97%; 37.6 mg; yellow solid; mp 121–124°C; TLC (PET:EtOAc, 100:1 v/v): R_f = 0.3; ^{1}H NMR (600 MHz, $CDCl_3$) δ=12.36 (s, 1H), 7.96 (d, *J* = 7.8 Hz, 1H), 7.55 (t, *J* = 7.8 Hz, 1H), 7.49 (s, 1H), 7.37–7.33 (m, 2H), 7.25 (dd, *J* = 17.4, 7.8 Hz, 4H), 7.13 (d, *J* = 7.8 Hz, 1H), 7.08 (d, *J* = 8.4 Hz, 1H), 6.99 (t, *J* = 7.2 Hz, 1H), 5.29 (d, *J* = 6.0 Hz, 1H), 4.11 (dd, *J* = 18.0, 9.6 Hz, 1H), 3.56–3.49 (m, 1H), 2.44 (s, 3H), 0.50 (s, 9H). ^{13}C NMR (150 MHz, $CDCl_3$) δ=203.6, 162.5, 147.1, 143.6, 138.1, 136.4, 135.7, 135.0, 130.3, 129.6, 128.4, 128.1, 127.7, 126.1, 119.3, 118.9, 118.6, 45.1, 43.4, 21.1, 0.6. HRMS (ESI) m/z calcd for $C_{25}H_{28}O_2SiNa+$ $(M+Na)^+$ 411.17508, found 411.17517.

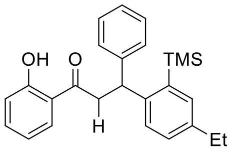

3-(4-ethyl-2-(trimethylsilyl)phenyl)-1-(2-hydroxyphenyl)-3-phenylpropan-1-one (75):

Yield 95%; 38.2 mg; yellow solid; mp 145–148°C; TLC (PET:EtOAc, 100:1 v/v): R_f = 0.3; ^{1}H NMR (600 MHz, $CDCl_3$) δ=12.39 (s, 1H), 7.97 (d, *J* = 7.8 Hz, 1H), 7.59–7.46 (m, 2H), 7.40–7.31 (m, 2H), 7.32–7.20 (m, 4H), 7.16 (d, *J* = 7.8 Hz, 1H), 7.09 (d, *J* = 8.4 Hz, 1H), 6.99 (d, *J* = 7.2 Hz, 1H), 5.31 (dd, *J* = 9.6, 3.6 Hz, 1H), 4.13 (dd, *J* = 18.0, 10.2 Hz, 1H), 3.53 (dd, *J* = 18.0, 4.2 Hz, 1H), 2.76 (dd, *J* = 15.0, 7.2 Hz, 2H), 1.37 (s, 3H), 0.53 (s, 9H). ^{13}C NMR (150 MHz, $CDCl_3$) δ=203.4, 162.2, 147.2, 143.4, 141.0, 137.7, 136.2, 134.3, 129.4, 128.8, 128.1, 127.9, 127.4, 125.9, 119.1, 118.7, 118.3, 44.8, 43.2, 28.3, 15.2, 0.4. HRMS (ESI) m/z calcd for $C_{26}H_{30}O_2SiNa+$ $(M+Na)^+$ 425.19073, found 425.19082.

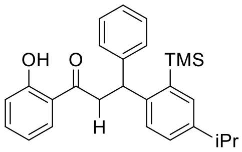

1-(2-hydroxyphenyl)-3-(4-isopropyl-2-(trimethylsilyl)phenyl)-3-phenylpropan-1-one (76):

Yield 91%; 37.8 mg; yellow solid; mp 138–141°C; TLC (PET:EtOAc, 100:1 v/v): R_f = 0.3; ^{1}H NMR (600 MHz, $CDCl_3$) δ=12.43 (s, 1H), 7.99 (d, *J* = 7.8 Hz, 1H), 7.62–7.54 (m, 2H), 7.39 (t, *J* = 7.2 Hz, 2H), 7.36–7.28 (m, 4H), 7.22 (d, *J* = 7.8 Hz, 1H), 7.12 (d, *J* = 8.4 Hz, 1H), 7.01 (t, *J* = 7.8 Hz, 1H), 5.35 (dd, *J* = 9.6, 3.0 Hz, 1H), 4.17 (dd, *J* = 18.0, 10.2 Hz, 1H), 3.57 (dd, *J* = 18.0, 3.6 Hz, 1H), 3.11–3.02 (m, 1H), 1.42 (dd, *J* = 6.6, 2.4 Hz, 6H), 0.59 (d, *J* = 16.2 Hz, 9H). ^{13}C NMR (150 MHz, $CDCl_3$)

δ=203.8, 162.6, 147.6, 145.9, 143.8, 138.0, 136.5, 133.4, 129.8, 128.5, 128.2, 127.9, 127.6, 126.2, 119.5, 119.0, 118.7, 45.3, 43.6, 33.9, 24.2, 24.1, 0.8. HRMS (ESI) m/z calcd for $C_{27}H_{33}O_2Si+$ $(M+H)^+$ 417.22443, found 417.22456.

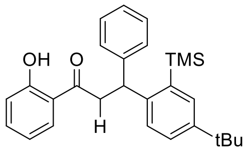

3-(4-(tert-butyl)-2-(trimethylsilyl)phenyl)-1-(2-hydroxyphenyl)-3-phenylpropan-1-one (77):

Yield 93%; 40.0 mg; yellow solid; mp 165–168°C; TLC (PET:EtOAc, 100:1 v/v): R_f = 0.3; ^{1}H NMR (600 MHz, $CDCl_3$) δ=12.16 (s, 1H), 7.73 (d, *J* = 7.8 Hz, 1H), 7.48 (s, 1H), 7.32 (t, *J* = 7.8 Hz, 1H), 7.21 (d, *J*= 7.8 Hz, 1H), 7.13 (t, *J* = 7.2 Hz, 2H), 7.09–7.03 (m, 3H), 6.94 (d, *J* = 8.4 Hz, 1H), 6.86 (d, *J* = 8.4 Hz, 1H), 6.76 (t,*J* = 7.8 Hz, 1H), 5.07 (dd, *J* = 9.6, 3.0 Hz, 1H), 3.91 (dd, *J* = 18.0, 10.2 Hz, 1H), 3.29 (dd, *J* = 18.0, 3.6 Hz, 1H), 1.22 (s, 9H), 0.30 (s, 9H). ^{13}C NMR (150 MHz, $CDCl_3$) δ=203.8, 162.6, 148.0, 147.2, 143.7, 137.5, 136.5, 131.8, 129.7, 128.5, 127.9, 127.8, 126.7, 126.2, 119.5, 119.0, 118.7, 45.3, 43.5, 34.6, 31.5, 0.8. HRMS (ESI) m/z calcd for $C_{28}H_{34}O_2SiNa+$ $(M+Na)^+$ 453.22203, found 453.22213.

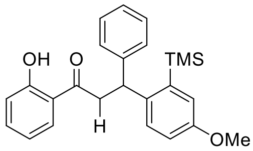

1-(2-hydroxyphenyl)-3-(4-methoxy-2-(trimethylsilyl)phenyl)-3-phenylpropan-1-one (78):

Yield 98%; 39.6 mg; yellow oil; TLC (PET:EtOAc, 50:1 v/v): R_f = 0.3; ^{1}H NMR (600 MHz, $CDCl_3$) δ=12.44 (s, 1H), 7.99 (d, *J* = 7.8 Hz, 1H), 7.60–7.53 (m, 1H), 7.41–7.34 (m, 2H), 7.36–7.25 (m, 4H), 7.22 (d, *J* = 8.4 Hz, 1H), 7.10 (d, *J* = 8.4 Hz, 1H), 7.00 (dd, *J* = 15.6, 7.8 Hz, 2H), 5.40–5.26 (m, 1H), 4.13 (dd, *J* = 18.0, 9.6 Hz, 1H), 3.90 (s, 3H), 3.57 (dd, *J* = 18.0, 4.2 Hz, 1H), 0.55 (s, 9H). ^{13}C NMR (150 MHz, $CDCl_3$) δ=203.8, 162.6, 157.3, 143.9, 142.0, 139.9, 136.5, 129.7, 129.3, 128.4, 127.7, 126.2, 120.8, 119.3, 119.0, 118.6, 114.2, 55.0, 45.3, 43.1, 0.5. HRMS (ESI) m/z calcd for $C_{25}H_{28}O_3SiNa+$ $(M+Na)^+$ 427.16999, found 427.17008.

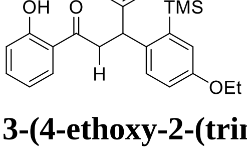

3-(4-ethoxy-2-(trimethylsilyl)phenyl)-1-(2-hydroxyphenyl)-3-phenylpropan-1-one (79):

Yield 97%; 40.6 mg; yellow solid; mp 155–156°C; TLC (PET:EtOAc, 100:1 v/v): R_f = 0.3; ^{1}H NMR (600 MHz, $CDCl_3$) δ=12.34 (s, 1H), 7.81 (d, *J* = 7.2 Hz, 1H), 7.40–7.33 (m, 1H), 7.17 (ddd, *J* = 24.6, 20.4, 6.6 Hz, 6H), 7.07 (d, *J* = 8.4 Hz, 1H),

6.93 (d, *J* = 7.8 Hz, 1H), 6.87–6.76 (m, 2H), 5.25–5.13 (m, 1H), 4.04–3.87 (m, 3H), 3.49–3.37 (m, 1H), 1.43–1.29 (m, 3H), 0.41 (s, 9H). ^{13}C NMR (150 MHz, $CDCl_3$) δ=204.1, 162.8, 157.0, 144.2, 142.0, 140.0, 136.7, 129.9, 129.5, 128.6, 127.9, 126.4, 121.7, 119.5, 119.2, 118.8, 114.9, 63.3, 45.5, 43.4, 15.2, 0.8. HRMS (ESI) m/z calcd for $C_{26}H_{30}O_3SNa+$ $(M+Na)^+$ 441.18564, found 441.18572.

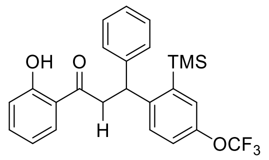

1-(2-hydroxyphenyl)-3-phenyl-3-(4-(trifluoromethoxy)-2-(trimethylsilyl)phenyl)propan-1-one (80):

Yield 94%; 43.0 mg; yellow oil; TLC (PET:EtOAc, 100:1 v/v): R_f = 0.3; ^{1}H NMR (600 MHz, $CDCl_3$) δ=12.19 (s, 1H), 7.88 (d, *J* = 7.8 Hz, 1H), 7.51 (t, *J* = 7.8 Hz, 1H), 7.37 (s, 1H), 7.28 (dd, *J* = 13.8, 6.6 Hz, 2H), 7.23–7.19 (m, 1H), 7.13 (t, *J* = 8.4 Hz, 4H), 7.01 (d, *J* = 8.4 Hz, 1H), 6.94 (t, *J* = 7.8 Hz, 1H), 5.21 (dd, *J* = 9.6, 4.2 Hz, 1H), 4.02 (dd, *J* = 18.0, 9.6 Hz, 1H), 3.39 (dd, *J* = 18.0, 4.2 Hz, 1H), 0.42 (s, 9H). ^{13}C NMR (150 MHz, $CDCl_3$) δ=203.1, 162.5, 148.7, 147.3, 142.9, 141.2, 136.6, 129.6, 128.6, 127.6, 127.1, 126.5, 121.7, 119.2, 119.0, 118.7, 44.9, 43.2, 0.3. ^{19}F NMR (375 MHz, $CDCl_3$) δ=57.63. HRMS (ESI) m/z calcd for $C_{25}H_{25}F_3O_3SiNa+$ $(M+Na)^+$ 481.14173, found 481.14181.

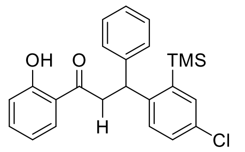

3-(4-chloro-2-(trimethylsilyl)phenyl)-1-(2-hydroxyphenyl)-3-phenylpropan-1-one (81):

Yield 93%; 37.9 mg; yellow solid; mp 190–193℃; TLC (PET:EtOAc, 100:1 v/v): R_f = 0.3; ^{1}H NMR (600 MHz, $CDCl_3$) δ=12.16 (s, 1H), 7.85 (d, *J* = 7.8 Hz, 1H), 7.48 (d, *J* = 6.6 Hz, 2H), 7.25 (d, *J* = 7.8 Hz, 3H), 7.19 (d, *J* = 7.2 Hz, 1H), 7.09 (d, *J* = 7.8 Hz, 2H), 7.00 (dd, *J* = 18.6, 8.4 Hz, 2H), 6.92 (t, *J* = 7.2 Hz, 1H), 5.15 (dd, *J* = 9.6, 4.2 Hz, 1H), 3.96 (dd, *J* = 18.0, 9.6 Hz, 1H), 3.37 (dd, *J* = 18.0, 4.2 Hz, 1H), 0.37 (s, 9H). ^{13}C NMR (150 MHz, $CDCl_3$) δ=203.0, 162.4, 148.2, 142.8, 141.0, 136.4, 134.5, 132.0, 129.5, 129.4, 129.3, 128.4, 127.5, 126.3, 119.1, 118.9, 118.5, 44.7, 43.1, 0.2. HRMS (ESI) m/z calcd for $C_{24}H_{25}ClO_2Si+$ $(M+Na)^+$ 431.12046, found 431.12056.

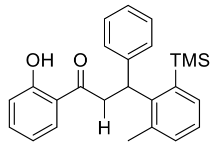

1-(2-hydroxyphenyl)-3-(2-methyl-6-(trimethylsilyl)phenyl)-3-phenylpropan-1-one (82):

Yield 96%; 37.3 mg; white solid; mp 173–176°C; TLC (PET:EtOAc, 100:1 v/v): R_f = 0.3; ^{1}H NMR (600 MHz, cdcl$_3$) δ=12.35 (s, 1H), 7.87 (d, *J* = 7.3 Hz, 1H), 7.49 – 7.41 (m, 2H), 7.18 (dt, *J* = 11.8, 7.6 Hz, 3H), 7.11 (dd, *J* = 12.5, 6.7 Hz, 2H), 7.00 (d, *J* = 8.3 Hz, 1H), 6.95 (d, *J* = 7.7 Hz, 2H), 6.88 (t, *J* = 7.3 Hz, 1H), 5.41 (d, *J* = 10.2 Hz, 1H), 4.40 (dd, *J* = 18.8, 10.5 Hz, 1H), 3.37 (d, *J* = 18.7 Hz, 1H), 1.92 (s, 3H), 0.36 (s, 9H). ^{13}C NMR (150 MHz, CDCl$_3$) δ=203.6, 162.4, 148.3, 142.5, 140.2, 136.7, 136.4, 133.7, 132.5, 129.3, 128.0, 126.9, 126.2, 125.5, 119.0, 118.9, 118.6, 42.9, 41.5, 21.2, 0.4. HRMS (ESI) m/z calcd for $C_{25}H_{29}O_2Si+$ $(M+H)^+$ 389.19313, found 389.19323.

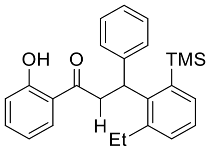

3-(2-ethyl-6-(trimethylsilyl)phenyl)-1-(2-hydroxyphenyl)-3-phenylpropan-1-one (83):

Yield 98%; 39.4 mg; white solid; mp 193–197 °C; TLC (PET:EtOAc, 100:1 v/v): R_f = 0.3; ^{1}H NMR (600 MHz, CDCl$_3$) δ=12.32 (s, 1H), 7.86 (d, *J* = 7.2 Hz, 1H), 7.44 (d, *J* = 19.2 Hz, 2H), 7.24 (s, 2H), 7.16 (s, 2H), 7.10 (s, 1H), 6.99 (d, *J* = 10.2 Hz, 3H), 6.88 (s, 1H), 5.40 (d, *J* = 9.6 Hz, 1H), 4.37 (dd, *J* = 18.0, 10.8 Hz, 1H), 3.41 (d, *J* = 18.6 Hz, 1H), 2.40–2.25 (m, 2H), 0.83 (s, 3H), 0.36 (s, 9H). ^{13}C NMR (150 MHz, CDCl$_3$) δ=204.0, 162.8, 148.2, 143.6, 143.2, 140.6, 136.7, 132.8, 132.3, 129.6, 128.3, 127.4, 126.9, 125.9, 119.4, 119.2, 119.0, 43.4, 43.1, 26.4, 15.3, 0.8. HRMS (ESI) m/z calcd for $C_{26}H_{30}O_2SiNa+$ $(M+Na)^+$ 425.19073, found 425.19080.

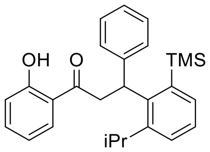

1-(2-hydroxyphenyl)-3-(2-isopropyl-6-(trimethylsilyl)phenyl)-3-phenylpropan-1-one (84):

Yield 97%; 40.4 mg; yellow solid; mp 125–128°C; TLC (PET:EtOAc, 100:1 v/v): R_f = 0.3; ^{1}H NMR (600 MHz, CDCl$_3$) δ=12.61 (s, 1H), 8.12 (d, *J* = 7.8 Hz, 1H), 7.77–7.63 (m, 2H), 7.61–7.51 (m, 2H), 7.41 (t, *J* = 7.2 Hz, 2H), 7.37–7.32 (m, 1H), 7.18 (dt, *J* = 15.0, 9.0 Hz, 4H), 5.66 (d, *J* = 10.2 Hz, 1H), 4.61 (dd, *J* = 18.6, 10.8 Hz, 1H), 3.71 (d, *J* = 18.6 Hz, 1H), 3.10–2.99 (m, 1H), 1.52 (d, *J* = 6.6 Hz, 3H), 1.13 (t, *J* = 6.6 Hz, 1H), 0.82 (d, *J* = 6.6 Hz, 2H), 0.63 (s, 9H). ^{13}C NMR (150 MHz, CDCl$_3$) δ=203.9, 162.9, 148.1, 147.6, 143.7, 140.5, 136.7, 132.8, 129.5, 128.2, 127.3, 127.1, 125.8, 119.4, 119.3, 119.0, 43.3, 30.1, 25.0, 23.1, 0.8. HRMS (ESI) m/z calcd for $C_{27}H_{32}O_2SiNa+$ $(M+Na)^+$ 439.20638, found 439.20647.

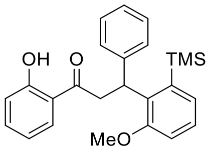

1-(2-hydroxyphenyl)-3-(2-methoxy-6-(trimethylsilyl)phenyl)-3-phenylpropan-1-one (85):

Yield 99%; 40.1 mg; white solid; mp 158–161 °C; TLC (PET:EtOAc, 50:1 v/v): R_f = 0.3; ^{1}H NMR (600 MHz, $CDCl_3$) δ=12.39 (s, 1H), 7.90 (d, *J* = 7.2 Hz, 1H), 7.42 (s, 1H), 7.24 (t, *J* = 6.6 Hz, 1H), 7.16 (dd, *J* = 27.0, 6.0 Hz, 3H), 7.07 (d, *J* = 6.0 Hz, 1H), 7.01 (d, *J* = 6.6 Hz, 2H), 6.97 (d, *J* = 7.8 Hz, 1H), 6.87 (s, 2H), 5.17 (d, *J* = 9.0 Hz, 1H), 4.56 (dd, *J* = 18.0, 9.6 Hz, 1H), 3.37 (d, *J* = 13.2 Hz, 4H), 0.37 (s, 9H). ^{13}C NMR (150 MHz, $CDCl_3$) δ=205.1, 162.7, 158.4, 143.7, 141.0, 138.9, 136.4, 129.9, 127.8, 127.3, 127.1, 125.4, 119.6, 119.0, 118.7, 114.4, 55.5, 42.0, 41.8, 0.7. HRMS (ESI) m/z calcd for $C_{25}H_{28}O_3SiNa+$ $(M+Na)^+$ 427.16999, found 427.17007.

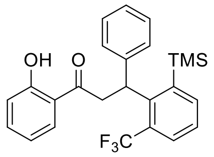

1-(2-hydroxyphenyl)-3-phenyl-3-(2-(trifluoromethyl)-6-(trimethylsilyl)phenyl)propan-1-one (86):

Yield 97%; 42.9 mg; yellow solid; mp 155–158°C; TLC (PET:EtOAc, 100:1 v/v): R_f = 0.3; ^{1}H NMR (600 MHz, $CDCl_3$) δ=12.28 (s, 1H), 7.87 (dd, *J* = 29.4, 6.0 Hz, 2H), 7.73 (d, *J* = 7.2 Hz, 1H), 7.41 (dd, *J* = 18.6, 6.6 Hz, 2H), 7.20–7.08 (m, 3H), 6.99 (d, *J* = 7.8 Hz, 1H), 6.87 (s, 3H), 5.65 (d, *J* = 9.0 Hz, 1H), 4.45 (dd, *J* = 18.0, 9.6 Hz, 1H), 3.52 (d, *J* = 19.2 Hz, 1H), 0.37 (s, 9H). ^{13}C NMR (150 MHz, $CDCl_3$) δ=203.9, 162.8, 147.8, 144.7, 142.7, 139.0, 136.7, 129.9, 129.7, 128.0, 127.1, 126.9, 125.9, 125.8, 124.0, 119.3, 118.8, 43.6, 42.9, 0.8. ^{19}F NMR (375 MHz, $CDCl_3$) δ=55.30. HRMS (ESI) m/z calcd for: $C_{25}H_{26}F_3O_2Si+$ $(M+H)^+$ 443.16487, found 443.16497.

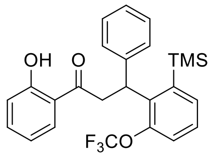

1-(2-hydroxyphenyl)-3-phenyl-3-(2-(trifluoromethoxy)-6-(trimethylsilyl)phenyl)propan-1-one (87):

Yield 97%; 44.4 mg; yellow oil; TLC (PET:EtOAc, 100:1 v/v): R_f = 0.3; ^{1}H NMR (600 MHz, $CDCl_3$) δ=12.23 (s, 1H), 7.86 (d, *J* = 7.2 Hz, 1H), 7.42 (d, *J* = 6.0 Hz, 1H), 7.37 (d, *J* = 7.2 Hz, 1H), 7.19 (dd, *J* = 15.0, 7.8 Hz, 2H), 7.10 (d, *J* = 6.6 Hz, 2H), 7.05 (d, *J* = 6.0 Hz, 1H), 6.98–6.90 (m, 3H), 6.83 (d, *J* = 7.2 Hz, 1H), 5.30 (d, *J* = 9.6 Hz, 1H), 4.48 (dd, *J* = 18.6, 10.2 Hz, 1H), 3.26 (d, *J* = 18.6 Hz, 1H), 0.36 (s, 9H). ^{13}C NMR (150 MHz, $CDCl_3$) δ=203.8, 162.8, 149.2, 143.1, 141.9, 141.1, 136.7, 132.6, 129.7, 128.2, 128.1, 127.0, 126.0, 120.0, 119.4, 119.3, 118.9, 41.4, 0.5. ^{19}F NMR (375 MHz, $CDCl_3$) δ=55.20. HRMS (ESI) m/z calcd for $C_{25}H_{25}F_3O_3SiNa+$ $(M+Na)^+$ 481.14173, found 481.14180.

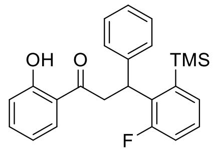

3-(2-fluoro-6-(trimethylsilyl)phenyl)-1-(2-hydroxyphenyl)-3-phenylpropan-1-one (88):

Yield 84%; 32.9 mg; yellow solid; mp 123–125°C; TLC (PET:EtOAc, 100:1 v/v): R_f = 0.3; ^{1}H NMR (600 MHz, $CDCl_3$) δ=12.29 (s, 1H), 7.88 (d, *J* = 7.2 Hz, 1H), 7.43 (s, 1H), 7.34 (d, *J* = 6.0 Hz, 1H), 7.21 (dd, *J* = 13.2, 6.6 Hz, 3H), 7.14 (d, *J* = 6.6 Hz, 1H), 7.07 (d, *J* = 6.0 Hz, 2H), 6.99 (t, *J* = 9.0 Hz, 2H), 6.88 (d, *J* = 6.6 Hz, 1H), 5.25 (d, *J* = 7.2 Hz, 1H), 4.36 (dd, *J* = 18.0, 9.6 Hz, 1H), 3.45 (d, *J* = 18.6 Hz, 1H), 0.38 (s, 9H). ^{13}C NMR (150 MHz, $CDCl_3$) δ=204.1, 162.9, 162.7, 161.3, 142.4, 136.6, 136.4, 136.4, 130.6, 129.9, 128.3, 128.3, 127.1, 126.2, 119.4, 119.1, 118.7, 117.6, 117.4, 42.5, 41.1, 0.6. ^{19}F NMR (375 MHz, $CDCl_3$) δ=111.04. HRMS (ESI) m/z calcd for $C_{24}H_{26}FO_2Si$+ $(M+H)^+$ 393.16806, found 393.16817.

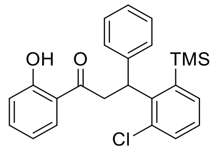

3-(2-chloro-6-(trimethylsilyl)phenyl)-1-(2-hydroxyphenyl)-3-phenylpropan-1-one (89):

Yield 95%; 38.7 mg; yellow solid; mp 140–143°C; TLC (PET:EtOAc, 100:1 v/v): R_f = 0.3; ^{1}H NMR (600 MHz, $CDCl_3$) δ=12.32 (s, 1H), 7.88 (d, *J* = 7.2 Hz, 1H), 7.48 (d, *J* = 6.6 Hz, 1H), 7.36 (s, 1H), 7.28 (d, *J* = 7.2 Hz, 1H), 7.19–7.04 (m, 4H), 6.94 (d, *J* = 6.0 Hz, 3H), 6.82 (d, *J* = 6.0 Hz, 1H), 5.44 (d, *J* = 9.0 Hz, 1H), 4.77 (dd, *J* = 18.6, 10.2 Hz, 1H), 3.36 (d, *J* = 18.6 Hz, 1H), 0.37 (s, 9H). ^{13}C NMR (150 MHz, $CDCl_3$) δ=204.2, 162.9, 147.3, 143.6, 142.0, 136.8, 135.6, 133.6, 132.9, 130.0, 128.3, 128.1, 127.4, 126.0, 119.5, 119.3, 118.9, 43.2, 41.6, 0.7. HRMS (ESI) m/z calcd for $C_{24}H_{26}ClO_2Si$+ $(M+H)^+$ 409.13851, found 409.13861.

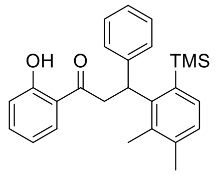

3-(2,3-dimethyl-6-(trimethylsilyl)phenyl)-1-(2-hydroxyphenyl)-3-phenylpropan-1-one (90):

Yield 97%; 39.0 mg; white solid; mp 190–193°C; TLC (PET:EtOAc, 100:1 v/v): R_f = 0.3; ^{1}H NMR (600 MHz, $CDCl_3$) δ=12.33 (s, 1H), 7.86 (d, *J* = 6.6 Hz, 1H), 7.44 (s, 1H), 7.37 (d, *J* = 5.4 Hz, 1H), 7.18 (s, 2H), 7.11 (d, *J* = 4.2 Hz, 2H), 6.98 (dd, *J* = 23.4, 6.6 Hz, 3H), 6.88 (s, 1H), 5.41 (d, *J* = 9.0 Hz, 1H), 4.36 (dd, *J* = 18.0, 10.2 Hz, 1H), 3.43 (d, *J* = 18.6 Hz, 1H), 2.21 (s, 3H), 1.87 (s, 3H), 0.33 (s, 9H). ^{13}C NMR (150 MHz, $CDCl_3$) δ=204.2, 162.7, 148.7, 143.6, 140.3, 138.2, 136.6, 135.8, 132.6, 129.7,

128.5, 128.3, 127.1, 125.7, 119.4, 119.1, 118.9, 43.5, 42.9, 21.0, 17.5, 0.8. HRMS (ESI) m/z calcd for $C_{26}H_{30}O_2SiNa+$ $(M+Na)^+$ 425.19073, found 425.19083.

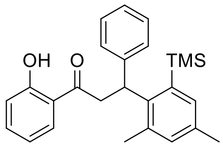

3-(2,4-dimethyl-6-(trimethylsilyl)phenyl)-1-(2-hydroxyphenyl)-3-phenylpropan-1-one (91):

Yield 94%; 37.8 mg; white solid; mp 201–204°C; TLC (PET:EtOAc, 100:1 v/v): R_f = 0.3; ^{1}H NMR (600 MHz, $CDCl_3$) δ=12.34 (d, *J* = 1.2 Hz, 1H), 7.86 (d, *J* = 7.8 Hz, 1H), 7.41 (t, *J* = 7.8 Hz, 1H), 7.28 (s, 1H), 7.15 (t, *J* = 6.6 Hz, 2H), 7.12–7.07 (m, 1H), 7.01–6.93 (m, 4H), 6.86 (t, *J* = 7.2 Hz, 1H), 5.40 (d, *J* = 10.2 Hz, 1H), 4.38 (dd, *J* = 18.6, 10.2 Hz, 1H), 3.36 (d, *J* = 18.6 Hz, 1H), 2.31 (s, 3H), 1.90 (s, 3H), 0.36 (s, 9H). ^{13}C NMR (150 MHz, $CDCl_3$) δ=204.2, 162.8, 145.8, 143.2, 140.5, 136.9, 136.7, 135.7, 134.9, 133.6, 129.7, 128.3, 127.3, 125.8, 119.5, 119.2, 42.9, 42.2, 21.5, 21.2, 0.9. HRMS (ESI) m/z calcd for $C_{26}H_{30}O_2SiNa+$ $(M+Na)^+$ 425.19073, found 425.19080.

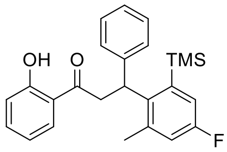

3-(4-fluoro-2-methyl-6-(trimethylsilyl)phenyl)-1-(2-hydroxyphenyl)-3-phenylpropan-1-one (92):

Yield 95%; 38.6 mg; yellow solid; mp 159–162°C; TLC (PET:EtOAc, 100:1 v/v): R_f = 0.3; ^{1}H NMR (600 MHz, $CDCl_3$) δ=12.32 (s, 1H), 7.91 (d, *J* = 7.2 Hz, 1H), 7.44 (d, *J* = 6.6 Hz, 1H), 7.22–7.11 (m, 4H), 6.98 (dd, *J* = 21.6, 7.2 Hz, 3H), 6.90 (s, 1H), 6.82 (d, *J* = 8.4 Hz, 1H), 5.43 (d, *J* = 9.6 Hz, 1H), 4.41 (dd, *J* = 18.0, 10.2 Hz, 1H), 3.36 (d, *J* = 18.6 Hz, 1H), 1.94 (s, 3H), 0.37 (s, 9H). ^{13}C NMR (150 MHz, $CDCl_3$) δ=203.9, 162.8, 162.2, 160.5, 144.4, 143.6, 142.7, 139.8, 139.7, 136.8, 129.7, 128.4, 127.2, 126.0, 120.1, 120.0, 119.4, 119.2, 118.9, 118.8, 118.7, 42.5, 42.0, 21.5, 0.5. HRMS (ESI) m/z calcd for $C_{25}H_{27}FO_2SiNa+$ $(M+Na)^+$ 429.16566, found 429.16576.

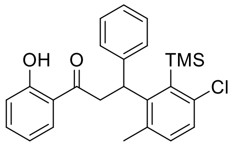

3-(3-chloro-6-methyl-2-(trimethylsilyl)phenyl)-1-(2-hydroxyphenyl)-3-phenylpropan-1-one (93):

Yield 78%; 32.9 mg; yellow solid; mp 133–136°C; TLC (PET:EtOAc, 100:1 v/v): R_f = 0.3; ^{1}H NMR (600 MHz, $CDCl_3$) δ=12.26 (s, 1H), 7.78 (d, *J* = 6.0 Hz, 1H), 7.47 (s, 1H), 7.23 (d, *J* = 17.4 Hz, 2H), 7.17 (d, *J* = 5.4 Hz, 2H), 7.01 (s, 4H), 6.89 (s, 1H), 5.62 (s, 1H), 4.14 (dd, *J* = 16.8, 6.6 Hz, 1H), 3.50 (d, *J* = 15.6 Hz, 1H), 1.97 (s, 3H), 0.48 (s, 9H). ^{13}C NMR (150 MHz, $CDCl_3$) δ=204.2, 162.6, 150.0, 142.9, 139.6, 139.4,

136.6, 135.6, 134.5, 129.6, 128.8, 128.4, 126.9, 125.9, 119.2, 119.0, 118.8, 42.6, 42.5, 21.4, 3.7. HRMS (ESI) m/z calcd for $C_{25}H_{27}ClO_2SiNa+$ $(M+Na)^+$ 445.13611, found 445.13620.

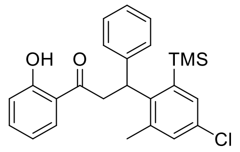

3-(4-chloro-2-methyl-6-(trimethylsilyl)phenyl)-1-(2-hydroxyphenyl)-3-phenylpropan-1-one (94):

Yield 90%; 38.0 mg; yellow solid; mp 201–204°C; TLC (PET:EtOAc, 100:1 v/v): R_f = 0.3; ^{1}H NMR (600 MHz, $CDCl_3$) δ=12.29 (s, 1H), 7.91 (d, *J* = 7.2 Hz, 1H), 7.47 (d, *J* = 6.6 Hz, 1H), 7.42 (s, 1H), 7.19 (d, *J* = 6.0 Hz, 2H), 7.14 (d, *J* = 13.2 Hz, 2H), 7.02 (d, *J* = 7.8 Hz, 1H), 6.98–6.91 (m, 3H), 5.41 (d, *J* = 9.6 Hz, 1H), 4.39 (dd, *J* = 18.6, 10.2 Hz, 1H), 3.35 (d, *J* = 18.6 Hz, 1H), 1.92 (s, 3H), 0.38 (s, 9H). ^{13}C NMR (150 MHz, $CDCl_3$) δ=203.6, 162.7, 147.0, 143.3, 142.3, 139.2, 136.8, 133.3, 132.5, 132.3, 129.6, 128.4, 127.1, 126.0, 119.3, 119.2, 118.9, 42.6, 41.8, 21.3, 0.5. HRMS (ESI) m/z calcd for $C_{25}H_{27}ClO_2SiNa+$ $(M+Na)^+$ 445.13611, found 445.13581.

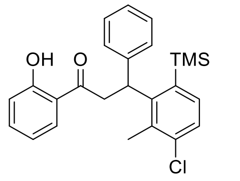

3-(3-chloro-2-methyl-6-(trimethylsilyl)phenyl)-1-(2-hydroxyphenyl)-3-phenylpropan-1-one (95):

Yield 97%; 40.9 mg; yellow solid; mp 190–193°C; TLC (PET:EtOAc, 100:1 v/v): R_f = 0.3; ^{1}H NMR (600 MHz, $CDCl_3$) δ=12.03 (s, 1H), 7.61 (d, *J* = 7.8 Hz, 1H), 7.20 (t, *J* = 7.8 Hz, 1H), 7.12 (d, *J* = 7.8 Hz, 1H), 7.05 (d, *J* = 7.8 Hz, 1H), 6.94 (t, *J* = 7.2 Hz, 2H), 6.91–6.86 (m, 1H), 6.76 (d, *J* = 8.4 Hz, 1H), 6.68 (d, *J* = 7.8 Hz, 2H), 6.64 (t, *J* = 7.8 Hz, 1H), 5.19 (d, *J* = 9.6 Hz, 1H), 4.09 (dd, *J* = 18.6, 10.2 Hz, 1H), 3.14 (d, *J* = 18.6 Hz, 1H), 1.76 (s, 3H), 0.08 (s, 9H).^{13}C NMR (150 MHz, $CDCl_3$) δ=203.6, 162.7, 150.7, 142.6, 139.6, 138.5, 136.7, 135.0, 133.5, 129.6, 128.4, 127.5, 126.9, 126.0, 119.2, 119.2, 118.9, 43.6, 42.4, 18.2, 0.5. HRMS (ESI) m/z calcd for $C_{25}H_{27}ClO_2SiNa+$ $(M+Na)^+$ 445.13611, found 445.13581.

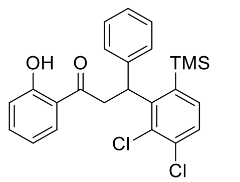

3-(2,3-dichloro-6-(trimethylsilyl)phenyl)-1-(2-hydroxyphenyl)-3-phenylpropan-1-one (96):

Yield 41%; 18.2 mg; yellow solid; mp 147–150℃; TLC (PET:EtOAc, 100:1 v/v): R_f = 0.3; ^{1}H NMR (600 MHz, $CDCl_3$) δ=12.27 (s, 1H), 7.91 (d, *J* = 7.8 Hz, 1H), 7.46–7.38 (m, 2H), 7.36 (d, *J* = 7.8 Hz, 1H), 7.17 (t, *J* = 7.2 Hz, 2H), 7.14–7.09 (m, 1H), 6.98 (d, *J* = 8.4 Hz, 1H), 6.94 (d, *J* = 7.8 Hz, 2H), 6.87 (t, *J* = 7.2 Hz, 1H), 5.48 (d, *J* = 9.6 Hz, 1H), 4.80 (dd, *J* = 19.2, 10.2 Hz, 1H), 3.35 (d, *J* = 18.6 Hz, 1H), 0.37 (s, 9H). ^{13}C NMR (150 MHz, $CDCl_3$) δ=203.8, 162.7, 149.6, 141.8, 141.3, 136.8, 136.1, 133.8, 133.5, 129.8, 128.7, 128.3, 127.1, 126.0, 119.3, 118.8, 43.7, 41.1, 0.4. HRMS (ESI) m/z calcd for $C_{24}H_{24}Cl_2O_2SiNa+$ $(M+Na)^+$ 465.08148, found 465.08157.

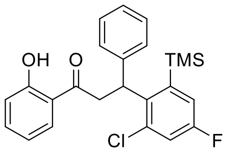

3-(2-chloro-4-fluoro-6-(trimethylsilyl)phenyl)-1-(2-hydroxyphenyl)-3-phenylpropan-1-one (97):

Yield 88%; 37.5 mg; yellow solid; mp 147–150℃; TLC (PET:EtOAc, 100:1 v/v): R_f = 0.3; ^{1}H NMR (600 MHz, $CDCl_3$) δ=12.26 (s, 1H), 7.94 (d, *J* = 7.2 Hz, 1H), 7.47 (s, 1H), 7.25–7.17 (m, 3H), 7.14 (d, *J* = 5.4 Hz, 1H), 7.07 (d, *J* = 7.2 Hz, 1H), 7.01 (d, *J* = 7.2 Hz, 1H), 6.92 (s, 3H), 5.41 (d, *J* = 9.0 Hz, 1H), 4.75 (dd, *J* = 18.6, 10.2 Hz, 1H), 3.35 (d, *J* = 18.6 Hz, 1H), 0.38 (s, 9H). ^{13}C NMR (150 MHz, $CDCl_3$) δ=203.8, 162.7, 161.8, 160.1, 145.6, 143.0, 141.5, 136.7, 135.9, 135.8, 129.8, 128.2, 127.1, 125.9, 120.0, 120.1, 119.6, 119.4, 119.3, 119.2, 118.8, 42.2, 41.4, 0.2. HRMS (ESI) m/z calcd for $C_{24}H_{25}ClFO_2Si+$ $(M+H)^+$ 427.12909, found 427.12919.

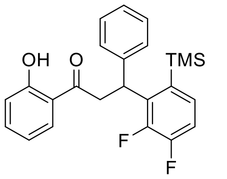

3-(2,3-difluoro-6-(trimethylsilyl)phenyl)-1-(2-hydroxyphenyl)-3-phenylpropan-1-one (98):

Yield 75%; 30.7 mg; yellow solid; mp 131–134℃; TLC (PET:EtOAc, 100:1 v/v): R_f = 0.3; ^{1}H NMR (600 MHz, $CDCl_3$) δ=12.23 (s, 1H), 7.90 (d, *J* = 7.8 Hz, 1H), 7.48 (t, *J* = 7.8 Hz, 1H), 7.29–7.25 (m, 1H), 7.25–7.21 (m, 2H), 7.19–7.15 (m, 1H), 7.07 (d, *J* = 7.8 Hz, 3H), 7.00 (d, *J* = 8.4 Hz, 1H), 6.92 (t, *J* = 7.8 Hz, 1H), 5.27 (d, *J* = 8.4 Hz, 1H), 4.37 (dd, *J* = 18.0, 9.6 Hz, 1H), 3.44 (d, *J* = 18.6 Hz, 1H), 0.37 (s, 9H). ^{13}C NMR (150 MHz, $CDCl_3$) δ=203.6, 162.6, 152.8, 151.0, 149.3, 141.5, 138.9, 136.6, 136.5, 130.5, 129.7, 128.3, 127.0, 126.3, 119.2, 119.1, 118.6, 115.5, 115.4, 42.1, 41.0, 0.4. ^{19}F NMR (375 MHz, $CDCl_3$) δ=136.43, 136.48, 137.51, 137.56. HRMS (ESI) m/z calcd for $C_{24}H_{25}F_2O_2Si+$ $(M+H)^+$ 411.15864, found 411.15874.

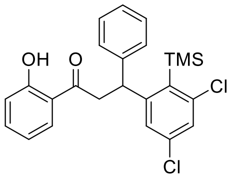

3-(3,5-dichloro-2-(trimethylsilyl)phenyl)-1-(2-hydroxyphenyl)-3-phenylpropan-1-one (99):

Yield 77%; 34.0 mg; yellow oil; TLC (PET:EtOAc, 100:1 v/v): R_f = 0.3; ^{1}H NMR (600 MHz, $CDCl_3$) δ=12.11 (s, 1H), 7.83 (d, *J* = 7.8 Hz, 1H), 7.48 (s, 1H), 7.29 (t, *J* = 7.8 Hz, 2H), 7.22 (d, *J* = 5.4 Hz, 2H), 7.09 (d, *J* = 7.8 Hz, 2H), 7.00–6.95 (m, 2H), 6.92 (t, *J* = 7.8 Hz, 1H), 5.40 (t, *J* = 7.2 Hz, 1H), 3.87 (dd, *J* = 18.0, 7.8 Hz, 1H), 3.53 (dd, *J* = 18.0, 6.6 Hz, 1H), 0.53 (s, 9H). ^{13}C NMR (150 MHz, $CDCl_3$) δ=202.9, 162.3, 153.5, 142.6, 142.3, 136.5, 135.9, 135.3, 129.5, 128.6, 128.1, 127.3, 126.9, 126.4, 126.2, 118.9, 118.5, 44.6, 42.2, 3.2. HRMS (ESI) m/z calcd for $C_{24}H_{25}Cl_2O_2Si+$ $(M+H)^+$ 443.09954, found 443.09985.

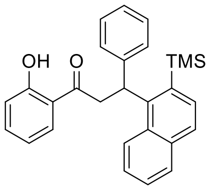

1-(2-hydroxyphenyl)-3-phenyl-3-(2-(trimethylsilyl)naphthalen-1-yl)propan-1-one (100):

Yield 91%; 38.6 mg; yellow solid; mp 231–234°C; TLC (PET:EtOAc, 100:1 v/v): R_f = 0.3; ^{1}H NMR (600 MHz, $CDCl_3$) δ=12.34 (d, *J* = 7.8 Hz, 1H), 7.83–7.71 (m, 3H), 7.67 (d, *J* = 7.2 Hz, 1H), 7.60 (d, *J* = 8.4 Hz, 1H), 7.43 (d, *J* = 7.2 Hz, 1H), 7.34 (d, *J* = 6.6 Hz, 1H), 7.22–7.16 (m, 3H), 7.12 (d, *J* = 6.0 Hz, 1H), 7.05 (d, *J* = 6.6 Hz, 2H), 7.01 (d, *J* = 7.8 Hz, 1H), 6.80 (d, *J* = 6.6 Hz, 1H), 5.73 (d, *J* = 9.0 Hz, 1H), 4.62 (dd, *J* = 18.0, 9.6 Hz, 1H), 3.49 (d, *J* = 18.6 Hz, 1H), 0.42 (d, *J* = 7.2 Hz, 9H). ^{13}C NMR (150 MHz, $CDCl_3$) δ=204.0, 162.6, 147.2, 143.5, 138.0, 136.6, 135.6, 131.5, 131.0, 129.7, 129.0, 128.4, 127.1, 127.0, 126.6, 125.9, 125.6, 125.4, 119.3, 119.1, 118.7, 44.0, 43.4, 0.6. HRMS (ESI) m/z calcd for $C_{28}H_{28}O_2SiNa+$ $(M+Na)^+$ 447.17508, found 447.17515.

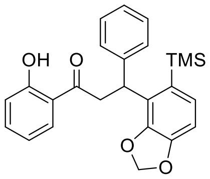

1-(2-hydroxyphenyl)-3-phenyl-3-(5-(trimethylsilyl)benzo[d][1,3]dioxol-4-yl)propan-1-one (101):

Yield 48%; 20.1 mg; white solid; mp 96–98 °C; TLC (PET:EtOAc, 100:1 v/v): R_f = 0.3; ^{1}H NMR (600 MHz, $CDCl_3$) δ=12.32 (s, 1H), 7.83 (d, *J* = 6.6 Hz, 1H), 7.38 (s, 1H), 7.17 (dd, *J* = 32.4, 15.6 Hz, 5H), 7.05 (d, *J* = 6.6 Hz, 1H), 6.93 (d, *J* = 7.8 Hz,

1H), 6.82 (s, 1H), 6.74 (d, *J* = 6.6 Hz, 1H), 5.73 (d, *J* = 13.2 Hz, 2H), 5.14 (s, 1H), 4.07 (dd, *J* = 17.4, 7.2 Hz, 1H), 3.78–3.70 (m, 1H), 0.31 (s, 9H). ^{13}C NMR (150 MHz, $CDCl_3$) δ=204.5, 162.7, 149.0, 146.2, 142.6, 136.5, 132.6, 130.3, 130.0, 129.2, 128.4, 127.7, 126.3, 119.5, 119.0, 118.6, 107.4, 100.3, 42.9, 41.8, 1.0. HRMS (ESI) m/z calcd for $C_{25}H_{26}O_4SiNa+$ $(M+Na)^+$ 441.14926, found 441.14935.

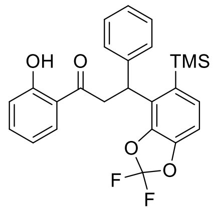

3-(2,2-difluoro-5-(trimethylsilyl)benzo[d][1,3]dioxol-4-yl)-1-(2-hydroxyphenyl)-3-phenylpropan-1-one (102):

Yield 35%; 15.9 mg; yellow solid; mp 120–123°C; TLC (PET:EtOAc, 100:1 v/v): R_f = 0.3; ^{1}H NMR (600 MHz, $CDCl_3$) δ=12.31 (s, 1H), 7.93 (d, *J* = 7.8 Hz, 1H), 7.56–7.51 (m, 1H), 7.41–7.32 (m, 3H), 7.27 (dd, *J* = 12.6, 5.4 Hz, 1H), 7.22 (d, *J* = 7.8 Hz, 2H), 7.04 (dt, *J* = 16.8, 8.4 Hz, 2H), 6.98 (t, *J* = 7.8 Hz, 1H), 5.37–5.29 (m, 1H), 4.15 (dd, *J* = 18.0, 8.4 Hz, 1H), 3.83 (dd, *J* = 18.0, 5.4 Hz, 1H), 0.43 (s, 9H). ^{13}C NMR (150 MHz, $CDCl_3$) δ=203.6, 162.5, 144.9, 142.7, 141.3, 136.6, 135.4, 131.2, 130.6, 129.8, 129.6, 129.0, 128.5, 127.4, 126.6, 119.3, 119.1, 118.5, 107.9, 42.7, 41.4, 0.7. ^{19}F NMR (375 MHz, $CDCl_3$) δ=49.99. HRMS (ESI) m/z calcd for $C_{25}H_{24}F_2O_4SiNa+$ $(M+Na)^+$ 477.13041, found 477.13051.

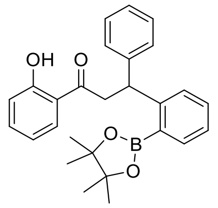

1-(2-hydroxyphenyl)-3-phenyl-3-(2-(4,4,5,5-tetramethyl-1,3,2-dioxaborolan-2-yl) phenyl)propan-1-one (103):

Yield 84%; 36.0 mg; white solid; mp 118–121°C; TLC (PET:EtOAc, 100:1 v/v): R_f = 0.3; ^{1}H NMR (600 MHz, $CDCl_3$) δ=12.26 (s, 1H), 7.80 (t, *J* = 8.4 Hz, 2H), 7.41 (t, *J* = 7.8 Hz, 1H), 7.31 (dd, *J* = 19.2, 7.8 Hz, 3H), 7.26 (t, *J* = 7.8 Hz, 2H), 7.17 (t, *J* = 8.4 Hz, 3H), 6.93 (d, *J* = 8.4 Hz, 1H), 6.84 (t, *J* = 7.8 Hz, 1H), 5.67 (t, *J* = 7.2 Hz, 1H), 3.80 (dd, *J* = 16.8, 7.8 Hz, 1H), 3.68 (dd, *J* = 16.8, 6.6 Hz, 1H), 1.29 (s, 6H), 1.24 (s, 6H). ^{13}C NMR (150 MHz, $CDCl_3$) δ=204.3, 162.4, 150.5, 144.2, 136.2, 136.1, 131.1, 129.9, 128.4, 128.1, 127.8, 127.2, 126.1, 125.6, 119.5, 118.8, 118.5, 83.7, 45.1, 43.3, 24.8. HRMS (ESI) m/z calcd for $C_{27}H_{30}BO_4^+$ $(M+H)^+$ 429.22317, found 429.22372.

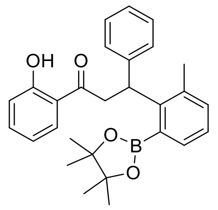

1-(2-hydroxyphenyl)-3-(2-methyl-6-(4,4,5,5-tetramethyl-1,3,2-dioxaborolan-2-yl) phenyl)-3-phenylpropan-1-one (104):

Yield 87%; 38.5 mg; colorless oil; TLC (PET:EtOAc, 100:1 v/v): R_f = 0.3; ^{1}H NMR (600 MHz, $CDCl_3$) δ=12.41 (s, 1H), 7.84 (d, *J* = 7.8 Hz, 1H), 7.60 (d, *J* = 6.6 Hz, 1H), 7.39 (t, *J* = 7.8 Hz, 1H), 7.18 (t, *J* = 7.2 Hz, 2H), 7.16–7.10 (m, 3H), 7.08 (d, *J* = 7.8 Hz, 2H), 6.95 (d, *J* = 8.4 Hz, 1H), 6.84 (t, *J* = 7.2 Hz, 1H), 5.64 (s, 1H), 4.36–4.27 (m, 1H), 3.59 (dd, *J* = 17.4, 4.2 Hz, 1H), 2.14 (s, 3H), 1.17 (d, *J* = 9.0 Hz, 12H). ^{13}C NMR (150 MHz, $CDCl_3$) δ=205.2, 162.6, 148.1, 143.7, 136.6, 136.2, 134.0, 133.6, 130.0, 128.0, 127.6, 126.1, 125.5, 119.6, 118.8, 118.5, 83.7, 41.7, 41.5, 24.9, 24.5, 21.3. HRMS (ESI) m/z calcd for $C_{28}H_{32}BO_4^+$ $(M+H)^+$ 443.23882, found 443.23932.

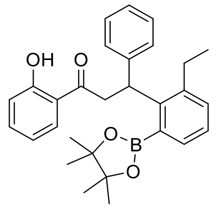

3-(2-ethyl-6-(4,4,5,5-tetramethyl-1,3,2-dioxaborolan-2-yl)phenyl)-1-(2-hydroxyphenyl)-3-phenylpropan-1-one (105):

Yield 62%; 28.3 mg; white solid; mp 105–108 °C; TLC (PET:EtOAc, 100:1 v/v): R_f = 0.3; ^{1}H NMR (600 MHz, $CDCl_3$) δ=12.43 (s, 1H), 7.89 (d, *J* = 7.8 Hz, 1H), 7.59 (d, *J* = 7.2 Hz, 1H), 7.43 (t, *J* = 7.8 Hz, 1H), 7.25 (d, *J* = 7.8 Hz, 1H), 7.19 (dd, *J* = 12.6, 7.2 Hz, 3H), 7.10 (t, *J* = 7.2 Hz, 1H), 7.05 (d, *J* = 4.8 Hz, 2H), 6.98 (d, *J* = 8.4 Hz, 1H), 6.87 (t, *J* = 7.8 Hz, 1H), 5.60 (s, 1H), 4.51 (s, 1H), 3.50 (d, *J* = 18.0 Hz, 1H), 2.62 (d, *J* = 57.0 Hz, 2H), 1.13 (d, *J* = 22.2 Hz, 12H), 0.98 (s, 3H). ^{13}C NMR (150 MHz, $CDCl_3$) δ=205.1, 162.5, 147.5, 144.2, 142.5, 136.1, 133.5, 132.1, 129.8, 127.9, 127.7, 126.2, 125.4, 119.5, 118.8, 118.4, 83.6, 42.8, 40.5, 26.8, 24.9, 24.4, 15.4. HRMS (ESI) m/z calcd for $C_{29}H_{34}BO_4^+$ $(M+H)^+$ 457.25447, found 457.25491.

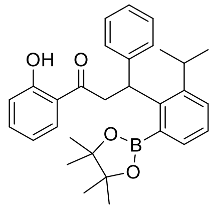

1-(2-hydroxyphenyl)-3-(2-isopropyl-6-(4,4,5,5-tetramethyl-1,3,2-dioxaborolan-2-yl)phenyl)-3-phenylpropan-1-one (106):

Yield 75%; 35.2 mg; white solid; mp 130–133℃; TLC (PET:EtOAc, 100:1 v/v): R_f = 0.3; ^{1}H NMR (600 MHz, $CDCl_3$) δ=12.43 (s, 1H), 7.86 (d, *J* = 6.6 Hz, 1H), 7.59 (d, *J* = 7.2 Hz, 1H), 7.42 (t, *J* = 7.8 Hz, 1H), 7.34 (d, *J* = 7.2 Hz, 1H), 7.24 (t, *J* = 7.8 Hz, 1H), 7.19 (t, *J* = 7.2 Hz, 2H), 7.16–7.00 (m, 3H), 6.97 (d, *J* = 8.4 Hz, 1H), 6.86 (t, *J* = 7.2 Hz, 1H), 5.72 (s, 1H), 4.34 (s, 1H), 3.55 (d, *J* = 15.0 Hz, 1H), 3.09 (s, 1H), 1.41–0.98 (m, 15H), 0.69 (s, 3H). ^{13}C NMR (150 MHz, $CDCl_3$) δ=204.9, 162.7, 147.4, 146.9, 144.4, 136.3, 133.2, 131.5, 129.9, 128.1, 127.6, 126.6, 125.5, 119.6, 119.0, 118.6, 83.8, 42.6, 29.5, 25.0, 24.7, 24.6, 23.1. HRMS (ESI) m/z calcd for $C_{30}H_{36}BO_4^+$ $(M+H)^+$ 471.27012, found 471.27084.

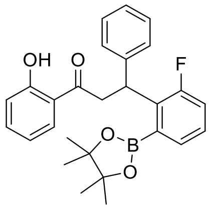

3-(2-fluoro-6-(4,4,5,5-tetramethyl-1,3,2-dioxaborolan-2-yl)phenyl)-1-(2-hydroxyphenyl)-3-phenylpropan-1-one (107):

Yield 81%; 36.1 mg; yellow oil; TLC (PET:EtOAc, 100:1 v/v): R_f = 0.3; ^{1}H NMR (600 MHz, $CDCl_3$) δ=12.27 (s, 1H), 7.82 (d, *J* = 7.8 Hz, 1H), 7.55 (d, *J* = 7.2 Hz, 1H), 7.46–7.33 (m, 3H), 7.25 (t, *J* = 7.8 Hz, 2H), 7.15 (dd, *J* = 13.2, 7.2 Hz, 2H), 6.98 (dd, *J* = 11.4, 8.4 Hz, 1H), 6.91 (d, *J* = 8.4 Hz, 1H), 6.83 (t, *J* = 7.8 Hz, 1H), 5.74 (t, *J* = 7.2 Hz, 1H), 3.97 (dd, *J* = 16.8, 6.6 Hz, 1H), 3.89 (dd, *J* = 16.8, 7.8 Hz, 1H), 1.33 (d, *J* = 9.0 Hz, 12H). ^{13}C NMR (150 MHz, $CDCl_3$) δ=204.8, 162.5, 162.5, 160.9, 143.1, 136.2, 136.2, 131.6, 131.6, 130.0, 128.2, 127.9, 127.9, 127.8, 127.8, 126.1, 119.6, 118.9, 118.9, 118.8, 118.4, 84.2, 41.5, 41.4, 40.4, 24.9. HRMS (ESI) m/z calcd for $C_{27}H_{29}BFO_4^+$ $(M+H)^+$ 447.21374, found 447.21402.

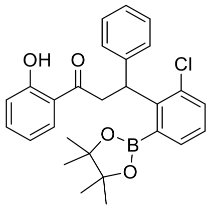

3-(2-chloro-6-(4,4,5,5-tetramethyl-1,3,2-dioxaborolan-2-yl)phenyl)-1-(2-hydroxyphenyl)-3-phenylpropan-1-one (108):

Yield 83%; 38.4 mg; white solid; mp 88–91°C; TLC (PET:EtOAc, 100:1 v/v): R_f = 0.3; ^{1}H NMR (600 MHz, $CDCl_3$) δ=12.34 (s, 1H), 7.88 (dd, *J* = 7.8, 1.2 Hz, 1H), 7.65 (dd, *J* = 7.2, 1.2 Hz, 1H), 7.48–7.35 (m, 1H), 7.34 (d, *J* = 7.8 Hz, 1H), 7.23 (t, *J* = 7.8 Hz, 2H), 7.19–7.12 (m, 4H), 6.95 (d, *J* = 8.4 Hz, 1H), 6.87 (t, *J* = 7.8 Hz, 1H), 5.87 (t, *J* = 6.6 Hz, 1H), 4.34 (d, *J* = 10.8 Hz, 1H), 3.81 (d, *J* = 13.2 Hz, 1H), 1.25 (d, *J* = 10.8 Hz, 12H). ^{13}C NMR (150 MHz, $CDCl_3$) δ=204.8, 162.5, 146.4, 142.3, 136.3, 134.8, 134.0, 133.2, 130.0, 127.9, 127.5, 125.7, 119.6, 118.9, 118.5, 84.2, 42.0, 40.2, 24.9, 24.7. HRMS (ESI) m/z calcd for $C_{27}H_{29}BClO_4^+$ $(M+H)^+$ 463.18419, found 463.18442.

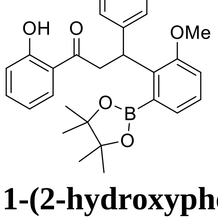

1-(2-hydroxyphenyl)-3-(2-methoxy-6-(4,4,5,5-tetramethyl-1,3,2-dioxaborolan-2-yl)phenyl)-3-phenylpropan-1-one (109):

Yield 89%; 40.8 mg; white solid; mp 147–150°C; TLC (PET:EtOAc, 50:1 v/v): R_f = 0.3; ^{1}H NMR (400 MHz, $CDCl_3$) δ=12.40 (s, 1H), 7.82 (dd, *J* = 8.0, 1.6 Hz, 1H), 7.38–7.32 (m, 4H), 7.21 (t, *J* = 7.6 Hz, 2H), 7.17–7.13 (m, 1H), 7.09 (t, *J* = 7.2 Hz,

1H), 6.89 (d, *J* = 8.4 Hz, 1H), 6.84–6.77 (m, 2H), 5.65 (t, *J* = 7.2 Hz, 1H), 4.05 (dd, *J* = 16.8, 6.8 Hz, 1H), 3.88 (dd, *J* = 16.8, 7.6 Hz, 1H), 3.49 (s, 3H), 1.30 (t, *J* = 6.8 Hz, 12H). ^{13}C NMR (100 MHz, $CDCl_3$) δ=206.0, 162.6, 158.0, 144.1, 137.9, 136.1, 130.3, 128.0, 127.9, 127.8, 127.5, 125.5, 119.9, 118.8, 118.4, 114.9, 84.0, 55.4, 41.5, 40.8, 25.0, 24.9. HRMS (ESI) m/z calcd for $C_{28}H_{32}BO_5^+$ $(M+H)^+$ 459.23373, found 459.23413.

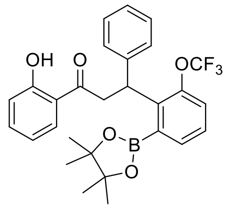

1-(2-hydroxyphenyl)-3-phenyl-3-(2-(4,4,5,5-tetramethyl-1,3,2-dioxaborolan-2-yl)-6-(trifluoromethoxy)phenyl)propan-1-one (110):

Yield 62%; 31.8 mg; white solid; mp 115–118℃; TLC (PET:EtOAc, 100:1 v/v): R_f = 0.3; ^{1}H NMR (600 MHz, $CDCl_3$) δ=12.28 (s, 1H), 7.86 (d, *J* = 7.8 Hz, 1H), 7.70–7.62 (m, 1H), 7.44 (t, *J* = 7.8 Hz, 1H), 7.33–7.19 (m, 6H), 7.15 (t, *J* = 7.2 Hz, 1H), 6.95 (d, *J* = 8.4 Hz, 1H), 6.89 (t, *J* = 7.8 Hz, 1H), 5.78 (t, *J* = 7.2 Hz, 1H), 4.18 (dd, *J* = 17.4, 7.2 Hz, 1H), 3.73 (dd, *J* = 17.4, 6.6 Hz, 1H), 1.30 (s, 12H). ^{13}C NMR (150 MHz, $CDCl_3$) δ=204.5, 162.5, 148.5, 142.4, 140.6, 136.2, 133.3, 129.8, 128.0, 127.6, 125.9, 121.4, 121.2, 119.5, 118.9, 118.5, 84.3, 40.5, 39.9, 24.8, 24.7. HRMS (ESI) m/z calcd for $C_{28}H_{29}BF_3O_5^+$ $(M+H)^+$ 513.20547, found 513.20569.

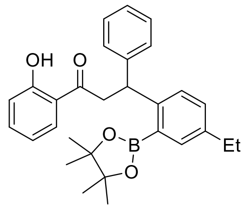

3-(4-ethyl-2-(4,4,5,5-tetramethyl-1,3,2-dioxaborolan-2-yl)phenyl)-1-(2-hydroxyphenyl)-3-phenylpropan-1-one (111):

Yield 68%; 31.0 mg; white solid; mp 137–140℃; TLC (PET:EtOAc, 100:1 v/v): R_f = 0.3; ^{1}H NMR (600 MHz, $CDCl_3$) δ=12.28 (s, 1H), 7.80 (d, *J* = 7.8 Hz, 1H), 7.62 (s, 1H), 7.40 (t, *J* = 7.8 Hz, 1H), 7.29 (d, *J* = 7.8 Hz, 2H), 7.24 (t, *J* = 7.8 Hz, 2H), 7.15 (dd, *J* = 15.6, 7.8 Hz, 2H), 7.09 (d, *J* = 7.8 Hz, 1H), 6.92 (d, *J* = 8.4 Hz, 1H), 6.83 (t, *J* = 7.8 Hz, 1H), 5.64 (t, *J* = 7.2 Hz, 1H), 3.78 (dd, *J* = 16.8, 8.4 Hz, 1H), 3.67 (dd, *J* = 16.8, 6.6 Hz, 1H), 2.59 (dd, *J* = 15.0, 7.8 Hz, 2H), 1.27 (d, *J* = 30.0 Hz, 12H), 1.19 (t, *J* = 7.8 Hz, 3H). ^{13}C NMR (150 MHz, $CDCl_3$) δ=204.4, 162.4, 147.8, 144.4, 141.2, 136.1, 135.7, 130.6, 129.9, 128.4, 128.1, 127.3, 126.1, 119.5, 118.8, 118.5, 83.7, 45.2, 43.0, 28.3, 24.8, 15.6. HRMS (ESI) m/z calcd for $C_{29}H_{34}BO_4^+$ $(M+H)^+$ 457.25447, found 457.25497.

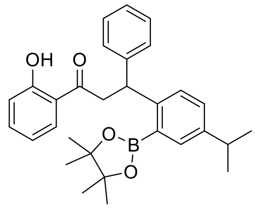

1-(2-hydroxyphenyl)-3-(4-isopropyl-2-(4,4,5,5-tetramethyl-1,3,2-dioxaborolan-2-yl)phenyl)-3-phenylpropan-1-one (112):

Yield 86%; 40.4 mg; white solid; mp 147–150°C; TLC (PET:EtOAc, 100:1 v/v): R_f = 0.3; ^{1}H NMR (600 MHz, $CDCl_3$) δ=12.27 (s, 1H), 7.80 (d, *J* = 7.8 Hz, 1H), 7.62 (s, 1H), 7.41 (t, *J* = 7.2 Hz, 1H), 7.29 (d, *J* = 7.2 Hz, 2H), 7.25 (t, *J* = 7.2 Hz, 2H), 7.21–7.18 (m, 1H), 7.15 (d, *J* = 6.6 Hz, 1H), 7.09 (d, *J* = 7.8 Hz, 1H), 6.93 (d, *J* = 8.4 Hz, 1H), 6.84 (t, *J* = 7.2 Hz, 1H), 5.63 (s, 1H), 3.79 (dd, *J* = 16.8, 8.4 Hz, 1H), 3.67 (dd, *J* = 16.8, 6.0 Hz, 1H), 2.90–2.83 (m, 1H), 1.30 (s, 6H), 1.25 (s, 6H), 1.21 (d, *J* = 3.0 Hz, 6H). ^{13}C NMR (150 MHz, $CDCl_3$) δ=204.3, 162.3, 147.8, 145.7, 144.3, 135.9, 134.3, 129.8, 128.9, 128.2, 128.0, 127.2, 126.0, 119.4, 118.7, 118.3, 83.6, 45.2, 43.0, 33.5, 24.8, 24.7, 23.9. HRMS (ESI) m/z calcd for $C_{30}H_{36}BO_4^+$ $(M+H)^+$ 471.27012, found 471.27045.

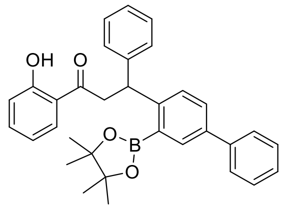

1-(2-hydroxyphenyl)-3-phenyl-3-(3-(4,4,5,5-tetramethyl-1,3,2-dioxaborolan-2-yl)-[1,1'-biphenyl]-4-yl)propan-1-one (113):

Yield 80%; 40.3 mg; white solid; mp 142–145°C; TLC (PET:EtOAc, 100:1 v/v): R_f = 0.3; ^{1}H NMR (600 MHz, $CDCl_3$) δ=12.26 (s, 1H), 8.02 (d, *J* = 1.8 Hz, 1H), 7.82 (d, *J* = 7.2 Hz, 1H), 7.55 (dd, *J* = 16.8, 4.8 Hz, 3H), 7.39 (dd, *J* = 16.8, 9.6 Hz, 3H), 7.33 (d, *J* = 7.2 Hz, 2H), 7.27 (dt, *J* = 17.4, 7.2 Hz, 4H), 7.17 (t, *J* = 7.2 Hz, 1H), 6.94 (d, *J* = 8.4 Hz, 1H), 6.85 (t, *J* = 7.2 Hz, 1H), 5.71 (t, *J* = 7.2 Hz, 1H), 3.83 (dd, *J* = 17.4, 7.8 Hz, 1H), 3.72 (dd, *J* = 17.4, 6.6 Hz, 1H), 1.31 (s, 6H), 1.26 (s, 6H). ^{13}C NMR (150 MHz, $CDCl_3$) δ=204.3, 162.5, 149.6, 144.2, 140.8, 138.4, 136.1, 135.0, 129.9, 129.7, 128.6, 128.4, 128.1, 127.7, 127.1, 127.1, 126.2, 119.5, 118.8, 118.5, 83.9, 45.1, 43.1, 24.9, 24.8. HRMS (ESI) m/z calcd for $C_{33}H_{34}BO_4^+$ $(M+H)^+$ 505.25447, found 505.25424.

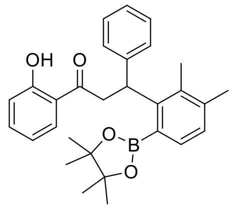

3-(2,3-dimethyl-6-(4,4,5,5-tetramethyl-1,3,2-dioxaborolan-2-yl)phenyl)-1-(2-hydroxyphenyl)-3-phenylpropan-1-one (114):

Yield 79%; 36.0 mg; yellow oil; TLC (PET:EtOAc, 100:1 v/v): R_f = 0.3; ^{1}H NMR (400 MHz, $CDCl_3$) δ=12.44 (s, 1H), 7.82 (d, *J* = 7.2 Hz, 1H), 7.53 (d, *J* = 7.6 Hz, 1H), 7.42–7.36 (m, 1H), 7.22–7.16 (m, 2H), 7.10 (dd, *J* = 7.6, 4.8 Hz, 3H), 7.05 (d, *J* = 7.6 Hz, 1H), 6.94 (d, *J* = 8.4 Hz, 1H), 6.83 (t, *J* = 7.6 Hz, 1H), 5.84–5.68 (m, 1H), 4.28 (dd, *J* = 16.8, 7.6 Hz, 1H), 3.57 (dd, *J* = 17.6, 4.4 Hz, 1H), 2.20 (s, 3H), 2.00 (s, 3H), 1.17 (s, 12H). ^{13}C NMR (100 MHz, $CDCl_3$) δ=205.3, 162.7, 148.5, 144.4, 141.0, 136.3, 135.5, 133.3, 130.1, 128.2, 127.6, 125.5, 119.7, 119.0, 118.6, 83.7, 42.3, 42.1, 25.0, 24.7, 21.5, 16.9. HRMS (ESI) m/z calcd for $C_{29}H_{34}BO_4^+$ $(M+H)^+$ 457.25447, found 457.25491.

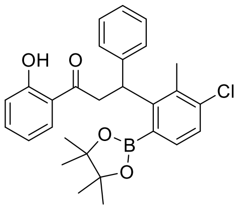

3-(3-chloro-2-methyl-6-(4,4,5,5-tetramethyl-1,3,2-dioxaborolan-2-yl)phenyl)-1-(2-hydroxyphenyl)-3-phenylpropan-1-one (115):

Yield 76%; 36.2 mg; white solid; mp 89–92℃; TLC (PET:EtOAc, 100:1 v/v): R_f = 0.3; ^{1}H NMR (600 MHz, $CDCl_3$) δ=12.33 (s, 1H), 7.83 (d, *J* = 7.8 Hz, 1H), 7.59–7.38 (m, 2H), 7.28 (d, *J* = 7.8 Hz, 1H), 7.23 (t, *J* = 6.6 Hz, 2H), 7.16 (d, *J* = 7.2 Hz, 1H), 7.06 (d, *J* = 7.2 Hz, 2H), 6.99 (d, *J* = 8.4 Hz, 1H), 6.89 (t, *J* = 7.2 Hz, 1H), 5.75 (s, 1H), 4.25 (s, 1H), 3.56 (d, *J* = 17.4 Hz, 1H), 2.15 (s, 3H), 1.20 (s, 12H). ^{13}C NMR (150 MHz, $CDCl_3$) δ=204.5, 162.5, 150.3, 143.2, 138.8, 136.3, 134.8, 133.9, 129.8, 128.2, 127.3, 125.7, 119.4, 118.9, 118.5, 84.0, 42.2, 41.6, 24.8, 24.5, 17.5. HRMS (ESI) m/z calcd for $C_{28}H_{31}BClO_4^+$ $(M+H)^+$ 477.19984, found 477.19937.

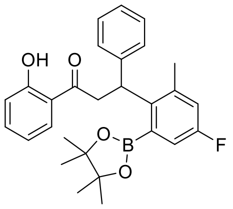

3-(4-fluoro-2-methyl-6-(4,4,5,5-tetramethyl-1,3,2-dioxaborolan-2-yl)phenyl)-1-(2-hydroxyphenyl)-3-phenylpropan-1-one (116):

Yield 83%; 38.2 mg; white solid; mp 113–116℃; TLC (PET:EtOAc, 100:1 v/v): R_f = 0.3; ^{1}H NMR (600 MHz, $CDCl_3$) δ=12.36 (s, 1H), 7.86 (d, *J* = 7.8 Hz, 1H), 7.45 (t, *J* = 7.2 Hz, 1H), 7.27 (dd, *J* = 8.4, 2.4 Hz, 1H), 7.22 (dd, *J* = 9.0, 5.4 Hz, 2H), 7.14 (t, *J* = 7.2 Hz, 1H), 7.07 (d, *J* = 7.2 Hz, 2H), 6.98 (d, *J* = 8.4 Hz, 1H), 6.88 (t, *J* = 7.8 Hz, 2H), 5.61 (s, 1H), 4.27 (dd, *J* = 16.8, 7.2 Hz, 1H), 3.60 (dd, *J* = 17.4, 4.8 Hz, 1H), 2.14 (s, 3H), 1.18 (d, *J* = 9.6 Hz, 12H). ^{13}C NMR (150 MHz, $CDCl_3$) δ=204.9, 162.4, 161.4, 159.8, 143.7, 143.3, 139.2, 139.2, 136.1, 129.8, 127.9, 127.3, 125.5, 120.1, 120.0, 119.4, 119.3, 118.7, 118.4, 83.9, 41.5, 40.6, 24.7, 24.4, 21.1. HRMS (ESI) m/z calcd for $C_{28}H_{31}BFO_4^+$ $(M+H)^+$ 461.22939, found 461.22974.

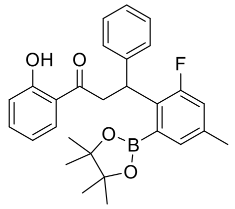

3-(2-fluoro-4-methyl-6-(4,4,5,5-tetramethyl-1,3,2-dioxaborolan-2-yl)phenyl)-1-(2-hydroxyphenyl)-3-phenylpropan-1-one (117):
Yield 81%; 37.3 mg; colorless oil; TLC (PET:EtOAc, 100:1 v/v): R_f = 0.3; ^{1}H NMR (600 MHz, $CDCl_3$) δ=12.29 (s, 1H), 7.84 (d, *J* = 7.8 Hz, 1H), 7.40 (t, *J* = 7.8 Hz, 3H), 7.36 (s, 1H), 7.25 (t, *J* = 7.8 Hz, 2H), 7.16 (d, *J* = 7.2 Hz, 1H), 6.92 (d, *J* = 7.8 Hz, 1H), 6.84 (dd, *J* = 18.6, 10.8 Hz, 2H), 5.69 (t, *J* = 7.2 Hz, 1H), 3.91 (dd, *J* = 22.2, 7.2 Hz, 2H), 2.25 (s, 3H), 1.32 (d, *J* = 9.6 Hz, 12H). ^{13}C NMR (150 MHz, $CDCl_3$) δ=204.9, 162.5, 160.8, 143.4, 138.1, 136.1, 133.0, 133.0, 132.1, 130.0, 128.1, 127.8, 126.0, 119.6, 119.5, 119.3, 118.8, 118.4, 84.1, 41.5, 40.1, 24.8, 20.7. HRMS (ESI) m/z calcd for $C_{28}H_{31}BFO_4^+$ $(M+H)^+$ 461.22939, found 461.22998.

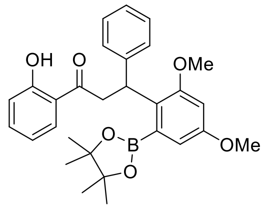

3-(2,4-dimethoxy-6-(4,4,5,5-tetramethyl-1,3,2-dioxaborolan-2-yl)phenyl)-1-(2-hydroxyphenyl)-3-phenylpropan-1-one (118):
Yield 85%; 41.5 mg; yellow oil; TLC (PET:EtOAc, 100:1 v/v): R_f = 0.3; ^{1}H NMR (600 MHz, $CDCl_3$) δ=12.44 (s, 1H), 7.80 (d, *J* = 7.8 Hz, 1H), 7.38–7.28 (m, 3H), 7.20 (t, *J* = 7.8 Hz, 2H), 7.08 (t, *J* = 7.2 Hz, 1H), 6.87 (d, *J* = 9.6 Hz, 2H), 6.77 (t, *J* = 7.8 Hz, 1H), 6.41 (s, 1H), 5.61 (t, *J* = 7.2 Hz, 1H), 3.93 (ddd, *J* = 24.0, 16.2, 7.2 Hz, 2H), 3.70 (s, 3H), 3.47 (s, 3H), 1.28 (d, *J* = 3.6 Hz, 12H). ^{13}C NMR (150 MHz, $CDCl_3$) δ=206.2, 162.6, 159.0, 144.5, 136.0, 132.0, 130.5, 130.3, 127.9, 127.7, 125.4, 119.8, 118.8, 118.4, 110.6, 102.7, 84.0, 55.2, 55.2, 40.9, 24.9, 24.8. HRMS (ESI) m/z calcd for $C_{29}H_{34}BO_6^+$ $(M+H)^+$ 489.24430, found 489.24493.

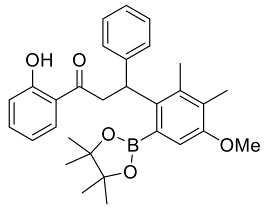

1-(2-hydroxyphenyl)-3-(4-methoxy-2,3-dimethyl-6-(4,4,5,5-tetramethyl-1,3,2-dioxaborolan-2-yl)phenyl)-3-phenylpropan-1-one (119):
Yield 87%; 42.3 mg; white solid; mp 152–155℃; TLC (PET:EtOAc, 100:1 v/v): R_f = 0.3; ^{1}H NMR (600 MHz, $CDCl_3$) δ=12.43 (s, 1H), 7.83 (d, *J* = 7.8 Hz, 1H), 7.42 (t, *J* = 7.8 Hz, 1H), 7.20 (dd, *J* = 14.4, 7.2 Hz, 2H), 7.11 (t, *J* = 7.8 Hz, 4H), 6.96 (d, *J* = 8.4 Hz, 1H), 6.85 (t, *J* = 7.8 Hz, 1H), 5.71 (s, 1H), 4.23 (s, 1H), 3.82 (d, *J* = 15.0 Hz, 3H), 3.56 (dd, *J* = 17.4, 4.2 Hz, 1H), 2.11 (s, 3H), 1.98 (s, 3H), 1.20 (s, 12H). ^{13}C

NMR (150 MHz, $CDCl_3$) δ=205.3, 162.6, 155.5, 144.6, 141.0, 136.9, 136.1, 130.0, 129.3, 128.0, 127.3, 125.3, 119.6, 118.8, 118.5, 114.2, 83.7, 55.5, 42.4, 41.7, 24.9, 24.6, 17.4, 12.4. HRMS (ESI) m/z calcd for $C_{30}H_{36}BO_5^+$ $(M+H)^+$ 487.26503, found 487.26547.

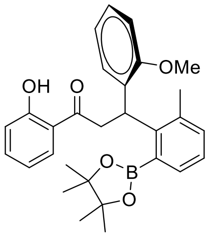

1-(2-hydroxyphenyl)-3-(2-methoxyphenyl)-3-(2-methyl-6-(4,4,5,5-tetramethyl-1,3,2-dioxaborolan-2-yl)phenyl)propan-1-one (120):

Yield 68%; 32.1 mg; white solid; mp 132–134°C; TLC (PET:EtOAc, 50:1 v/v): R_f = 0.3; ^1H NMR (600 MHz, $CDCl_3$) δ=12.42 (s, 1H), 7.86 (d, *J* = 7.8 Hz, 1H), 7.51 (d, *J* = 6.6 Hz, 1H), 7.44 (t, *J* = 7.2 Hz, 1H), 7.16–7.08 (m, 4H), 6.97 (d, *J* = 8.4 Hz, 1H), 6.86 (t, *J* = 7.2 Hz, 1H), 6.81 (t, *J* = 7.2 Hz, 1H), 6.74 (d, *J* = 7.8 Hz, 1H), 5.57 (s, 1H), 4.37 (dd, *J* = 16.8, 9.6 Hz, 1H), 3.51 (s, 3H), 3.45 (dd, *J* = 16.8, 4.2 Hz, 1H), 2.23 (s, 3H), 1.24 (d, *J* = 10.8 Hz, 12H). ^{13}C NMR (150 MHz, $CDCl_3$) δ=205.1, 162.4, 157.5, 147.4, 136.4, 135.8, 133.4, 132.8, 130.9, 129.9, 128.8, 127.1, 125.5, 119.8, 118.7, 118.3, 110.7, 83.7, 54.8, 41.5, 38.8, 25.1, 24.5, 21.3. HRMS (ESI) m/z calcd for $C_{29}H_{34}BO_5^+$ $(M+H)^+$ 473.24938, found 473.24997.

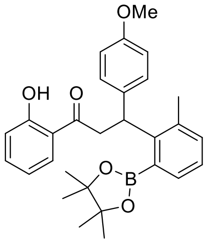

1-(2-hydroxyphenyl)-3-(4-methoxyphenyl)-3-(2-methyl-6-(4,4,5,5-tetramethyl-1,3,2-dioxaborolan-2-yl)phenyl)propan-1-one (121):

Yield 61%; 28.8 mg; yellow oil; TLC (PET:EtOAc, 50:1 v/v): R_f = 0.3; ^1H NMR (600 MHz, $CDCl_3$) δ=12.42 (s, 1H), 7.85 (d, *J* = 7.8 Hz, 1H), 7.57 (d, *J* = 7.2 Hz, 1H), 7.40 (t, *J* = 7.8 Hz, 1H), 7.13 (dt, *J* = 14.4, 7.2 Hz, 2H), 6.99 (d, *J* = 8.4 Hz, 2H), 6.95 (d, *J* = 8.4 Hz, 1H), 6.84 (t, *J* = 7.8 Hz, 1H), 6.75 (d, *J* = 8.4 Hz, 2H), 5.56 (s, 1H), 4.27 (d, *J* = 7.8 Hz, 1H), 3.69 (s, 3H), 3.61–3.54 (m, 1H), 2.15 (s, 3H), 1.19 (d, *J* = 12.6 Hz, 12H). ^{13}C NMR (150 MHz, $CDCl_3$) δ=205.3, 162.6, 157.6, 148.3, 136.6, 136.3, 135.7, 134.1, 133.5, 131.1, 130.1, 128.7, 126.1, 119.6, 118.9, 118.5, 113.5, 83.8, 55.2, 41.8, 41.1, 25.0, 24.6, 21.3. HRMS (ESI) m/z calcd for $C_{29}H_{34}BO_5^+$ $(M+H)^+$ 473.24938, found 473.24991.

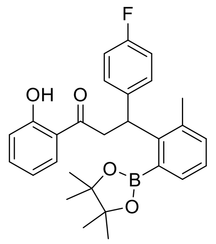

3-(4-fluorophenyl)-1-(2-hydroxyphenyl)-3-(2-methyl-6-(4,4,5,5-tetramethyl-1,3,2-dioxaborolan-2-yl)phenyl)propan-1-one (122):

Yield 56%; 25.7 mg; yellow oil; TLC (PET:EtOAc, 100:1 v/v): R_f = 0.3; ^{1}H NMR (600 MHz, $CDCl_3$) δ=12.35 (s, 1H), 7.85 (d, *J* = 7.8 Hz, 1H), 7.59 (d, *J* = 7.2 Hz, 1H), 7.46 (t, *J* = 7.8 Hz, 1H), 7.17 (dt, *J* = 13.2, 6.6 Hz, 2H), 7.10–7.01 (m, 2H), 6.99 (d, *J* = 8.4 Hz, 1H), 6.89 (dt, *J* = 13.2, 7.8 Hz, 3H), 5.58 (s, 1H), 4.29 (dd, *J* = 16.8, 7.8 Hz, 1H), 3.60 (dd, *J* = 17.4, 3.0 Hz, 1H), 2.15 (s, 3H), 1.19 (d, *J* = 5.4 Hz, 12H). ^{13}C NMR (150 MHz, $CDCl_3$) δ=204.9, 162.5, 161.8, 160.1, 147.9, 139.3, 136.5, 136.3, 134.1, 133.6, 129.9, 129.0, 129.0, 126.2, 119.5, 118.9, 118.5, 114.8, 114.6, 83.8, 41.9, 40.9, 24.9, 24.5, 21.2. ^{19}F NMR (375MHz, $CDCl_3$) δ=118.11. HRMS (ESI) m/z calcd for $C_{28}H_{31}BFO_4^+$ $(M+H)^+$ 461.22939, found 461.22995.

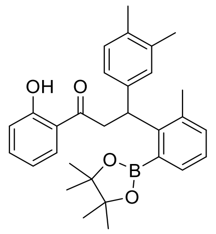

3-(3,4-dimethylphenyl)-1-(2-hydroxyphenyl)-3-(2-methyl-6-(4,4,5,5-tetramethyl-1,3,2-dioxaborolan-2-yl)phenyl)propan-1-one (123):

Yield 73%; 34.3 mg; yellow oil; TLC (PET:EtOAc, 100:1 v/v): R_f = 0.3; ^{1}H NMR (600 MHz, $CDCl_3$) δ=12.43 (s, 1H), 7.86 (d, *J* = 7.8 Hz, 1H), 7.57 (d, *J* = 6.6 Hz, 1H), 7.40 (t, *J* = 7.2 Hz, 1H), 7.12 (dt, *J* = 14.4, 7.2 Hz, 2H), 6.95 (dd, *J* = 10.2, 9.0 Hz, 2H), 6.88–6.80 (m, 3H), 5.57 (s, 1H), 4.27 (dd, *J* = 16.2, 7.2 Hz, 1H), 3.56 (dd, *J* = 17.4, 4.8 Hz, 1H), 2.15 (d, *J* = 18.0 Hz, 9H), 1.18 (d, *J* = 16.8 Hz, 12H). ^{13}C NMR (150 MHz, $CDCl_3$) δ=205.4, 162.6, 148.3, 140.9, 136.7, 136.2, 136.0, 134.1, 133.7, 133.4, 131.2, 130.1, 129.3, 129.1, 126.1, 125.1, 119.7, 118.9, 118.5, 83.8, 41.6, 25.0, 24.6, 21.4, 20.0, 19.3. HRMS (ESI) m/z calcd for $C_{30}H_{36}BO_4^+$ $(M+H)^+$ 471.27012, found 471.27090.

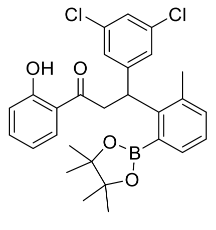

3-(3,5-dichlorophenyl)-1-(2-hydroxyphenyl)-3-(2-methyl-6-(4,4,5,5-tetramethyl-1,3,2-dioxaborolan-2-yl)phenyl)propan-1-one (124):

Yield 51%; 26.0 mg; yellow oil; TLC (PET:EtOAc, 100:1 v/v): R_f = 0.3; ^{1}H NMR (600 MHz, $CDCl_3$) δ=12.25 (s, 1H), 7.81 (d, *J* = 7.8 Hz, 1H), 7.65 (d, *J* = 7.2 Hz, 1H), 7.47 (t, *J* = 7.8 Hz, 1H), 7.23–7.14 (m, 3H), 7.02–6.94 (m, 3H), 6.88 (t, *J* = 7.8 Hz, 1H), 5.58 (s, 1H), 4.19 (s, 1H), 3.68 (d, *J* = 17.4 Hz, 1H), 2.20 (s, 3H), 1.21 (s, 12H). ^{13}C NMR (150 MHz, $CDCl_3$) δ=204.4, 162.5, 147.7, 146.8, 136.4, 134.5, 134.3, 129.8, 126.6, 126.2, 125.8, 119.3, 118.9, 118.6, 83.9, 41.8, 40.8, 24.9, 24.5, 21.3. HRMS (ESI) m/z calcd for $C_{28}H_{30}BCl_2O_4^+$ $(M+H)^+$ 511.16087, found 511.16025.

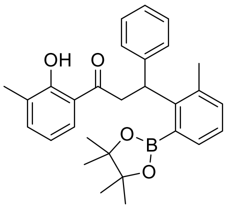

1-(2-hydroxy-3-methylphenyl)-3-(2-methyl-6-(4,4,5,5-tetramethyl-1,3,2-dioxabor olan-2-yl)phenyl)-3-phenylpropan-1-one (125):

Yield 64%; 29.2 mg; orange oil; TLC (PET:EtOAc, 100:1 v/v): R_f = 0.3; ^{1}H NMR (600 MHz, $CDCl_3$) δ=12.73 (s, 1H), 7.72 (d, *J* = 7.8 Hz, 1H), 7.59 (d, *J* = 7.2 Hz, 1H), 7.29 (d, *J* = 7.2 Hz, 1H), 7.19 (t, *J* = 7.2 Hz, 2H), 7.15 (d, *J* = 6.0 Hz, 1H), 7.12 (d, *J* = 7.8 Hz, 1H), 7.10 (d, *J* = 7.2 Hz, 1H), 7.07 (d, *J* = 7.8 Hz, 2H), 6.76 (t, *J* = 7.8 Hz, 1H), 5.65 (s, 1H), 4.35 (d, *J* = 9.0 Hz, 1H), 3.55 (dd, *J* = 17.4, 4.2 Hz, 1H), 2.24 (s, 3H), 2.14 (s, 3H), 1.17 (d, *J* = 11.4 Hz, 12H). ^{13}C NMR (150 MHz, $CDCl_3$) δ=205.3, 161.1, 148.3, 143.8, 137.0, 136.7, 134.1, 133.6, 131.2, 130.9, 128.1, 127.7, 127.6, 126.1, 125.5, 118.9, 118.2, 83.8, 41.8, 41.6, 25.0, 24.6, 21.3, 15.6. HRMS (ESI) m/z calcd for $C_{29}H_{34}BO_4^+$ $(M+H)^+$ 457.25447, found 457.25519.

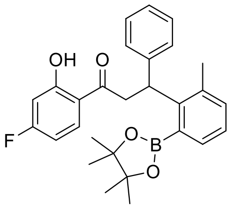

1-(4-fluoro-2-hydroxyphenyl)-3-(2-methyl-6-(4,4,5,5-tetramethyl-1,3,2-dioxaboro lan-2-yl)phenyl)-3-phenylpropan-1-one (126):

Yield 62%; 28.5 mg; colorless oil; TLC (PET:EtOAc, 100:1 v/v): R_f = 0.3; ^{1}H NMR (600 MHz, $CDCl_3$) δ=12.74 (s, 1H), 7.84 (dd, *J* = 8.4, 6.6 Hz, 1H), 7.59 (d, *J* = 6.6 Hz, 1H), 7.20 (t, *J* = 7.8 Hz, 2H), 7.13 (dt, *J* = 12.0, 7.2 Hz, 3H), 7.07 (d, *J* = 7.8 Hz, 2H), 6.62 (dd, *J* = 10.2, 2.4 Hz, 1H), 6.55 (td, *J* = 9.0, 2.4 Hz, 1H), 5.61 (s, 1H), 4.22 (dd, *J* = 16.8, 7.8 Hz, 1H), 3.60 (dd, *J* = 17.4, 4.8 Hz, 1H), 2.14 (s, 3H), 1.17 (d, *J* = 13.2 Hz, 12H). ^{13}C NMR (150 MHz, $CDCl_3$) δ=204.3, 168.1, 166.4, 165.2, 165.1, 147.9, 143.6, 136.6, 134.1, 133.7, 132.5, 132.4, 128.1, 127.6, 126.2, 125.6, 116.8, 107.1, 107.0, 105.1, 104.9, 83.8, 41.8, 25.0, 24.6, 21.3. HRMS (ESI) m/z calcd for $C_{28}H_{31}BFO_4^+$ $(M+H)^+$ 461.22939, found 461.22980.

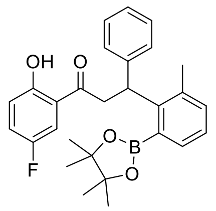

1-(5-fluoro-2-hydroxyphenyl)-3-(2-methyl-6-(4,4,5,5-tetramethyl-1,3,2-dioxaborolan-2-yl)phenyl)-3-phenylpropan-1-one (127):

Yield 58%; 26.7 mg; yellow oil; TLC (PET:EtOAc, 100:1 v/v): R_f = 0.3; ^{1}H NMR (600 MHz, $CDCl_3$) δ=12.13 (s, 1H), 7.60 (d, *J* = 7.2 Hz, 1H), 7.54 (d, *J* = 7.2 Hz, 1H), 7.24–7.13 (m, 3H), 7.13–7.04 (m, 5H), 6.87 (dd, *J* = 9.0, 4.8 Hz, 1H), 5.60 (s, 1H), 4.21 (dd, *J* = 16.8, 7.2 Hz, 1H), 3.70–3.60 (m, 1H), 2.18 (s, 3H), 1.17 (d, *J* = 15.0 Hz, 12H). ^{13}C NMR (150 MHz, $CDCl_3$) δ=204.8, 158.8, 155.6, 154.0, 147.8, 143.6, 136.7, 134.1, 133.9, 131.0, 128.2, 127.7, 126.3, 125.7, 123.9, 123.8, 119.9, 119.8, 119.2, 119.1, 115.3, 115.1, 83.8, 41.9, 41.5, 25.0, 24.6, 21.3. ^{19}F NMR (375 MHz, $CDCl_3$) δ=-123.73. HRMS (ESI) m/z calcd for $C_{28}H_{31}BFO_4^+$ $(M+H)^+$ 461.22939, found 461.22958.

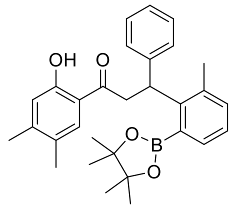

1-(2-hydroxy-4,5-dimethylphenyl)-3-(2-methyl-6-(4,4,5,5-tetramethyl-1,3,2-dioxaborolan-2-yl)phenyl)-3-phenylpropan-1-one (128):

Yield 72%; 33.8 mg; yellow oil; TLC (PET:EtOAc, 100:1 v/v): R_f = 0.3; ^{1}H NMR (600 MHz, $CDCl_3$) δ=12.25 (s, 1H), 7.58 (d, *J* = 10.8 Hz, 2H), 7.19 (dd, *J* = 14.4, 7.2 Hz, 3H), 7.13 (dd, *J* = 15.0, 7.2 Hz, 2H), 7.07 (d, *J* = 7.2 Hz, 2H), 6.77 (s, 1H), 5.60 (s, 1H), 4.33 (d, *J* = 7.2 Hz, 1H), 3.51 (dd, *J* = 17.4, 4.2 Hz, 1H), 2.21 (d, *J* = 34.8 Hz, 9H), 1.17 (d, *J* = 12.0 Hz, 12H). ^{13}C NMR (150 MHz, $CDCl_3$) δ=204.4, 160.9, 148.3, 146.6, 143.9, 136.6, 133.9, 133.5, 130.2, 128.0, 127.7, 127.0, 126.1, 125.5, 119.0, 117.5, 83.7, 41.7, 41.5, 25.0, 24.6, 21.3, 20.5, 19.0. HRMS (ESI) m/z calcd for $C_{30}H_{36}BO_4^+$ $(M+H)^+$ 471.27012, found 471.27075.

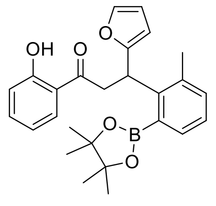

3-(furan-2-yl)-1-(2-hydroxyphenyl)-3-(2-methyl-6-(4,4,5,5-tetramethyl-1,3,2-dioxaborolan-2-yl)phenyl)propan-1-one (129):

Yield 77%; 33.3 mg; yellow solid; mp 130–133°C; TLC (PET:EtOAc, 100:1 v/v): R_f = 0.3; ^{1}H NMR (600 MHz, $CDCl_3$) δ=12.45 (s, 1H), 7.90 (d, *J* = 7.8 Hz, 1H), 7.65 (d, *J* = 7.2 Hz, 1H), 7.47 (t, *J* = 7.8 Hz, 1H), 7.28 (s, 1H), 7.25 (d, *J* = 7.2 Hz, 1H), 7.20 (t, *J* = 7.2 Hz, 1H), 7.01 (d, *J* = 8.4 Hz, 1H), 6.90 (t, *J* = 7.8 Hz, 1H), 6.29 (d, *J* = 1.8 Hz,

1H), 5.85 (s, 1H), 5.58 (s, 1H), 4.42 (d, J = 9.0 Hz, 1H), 3.54 (dd, J = 17.4, 4.8 Hz, 1H), 2.31 (s, 3H), 1.27 (d, J = 34.8 Hz, 12H). ^{13}C NMR (150 MHz, $CDCl_3$) δ=204.8, 162.5, 157.4, 145.3, 140.6, 136.5, 136.1, 133.8, 130.1, 126.4, 119.7, 118.8, 118.4, 110.6, 106.0, 83.7, 41.5, 37.1, 24.9, 24.9, 20.7. HRMS (ESI) m/z calcd for $C_{26}H_{30}BO_5^+$ $(M+H)^+$ 433.21808, found 433.21875.

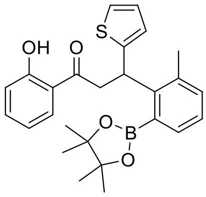

1-(2-hydroxyphenyl)-3-(2-methyl-6-(4,4,5,5-tetramethyl-1,3,2-dioxaborolan-2-yl) phenyl)-3-(thiophen-2-yl)propan-1-one (130):

Yield 63%; 28.2 mg; white solid; mp 135–138℃; TLC (PET:EtOAc, 100:1 v/v): R_f = 0.3; ^{1}H NMR (600 MHz, $CDCl_3$) δ=12.37 (s, 1H), 7.85 (d, J = 7.8 Hz, 1H), 7.59 (d, J = 7.2 Hz, 1H), 7.42 (t, J = 7.8 Hz, 1H), 7.19 (d, J = 7.2 Hz, 1H), 7.15 (t, J = 7.2 Hz, 1H), 7.08 (d, J = 4.8 Hz, 1H), 6.95 (d, J = 8.4 Hz, 1H), 6.85 (t, J = 6.6 Hz, 2H), 6.60 (s, 1H), 5.69 (s, 1H), 4.26 (s, 1H), 3.80 (d, J = 16.2 Hz, 1H), 2.31 (s, 3H), 1.19 (d, J = 7.8 Hz, 12H). ^{13}C NMR (150 MHz, $CDCl_3$) δ=204.8, 162.6, 149.4, 147.0, 136.6, 136.3, 133.8, 130.6, 130.1, 126.5, 126.4, 124.2, 123.5, 119.7, 118.9, 118.5, 83.8, 43.9, 38.5, 24.9, 24.6, 21.1. HRMS (ESI) m/z calcd for $C_{26}H_{30}BO_4S^+$ $(M+H)^+$ 449.19524, found 449.19592.

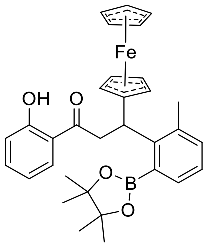

1-(2-hydroxyphenyl)-3-(2-methyl-6-(4,4,5,5-tetramethyl-1,3,2-dioxaborolan-2-yl) phenyl)-3-ferroceneylpropan-1-one (131):

Yield 91%; 50.0 mg; brown solid; mp 138–142℃; TLC (PET:EtOAc, 100:1 v/v): R_f = 0.3; ^{1}H NMR (600 MHz, $CDCl_3$) δ=12.69 (s, 0.25H), 12.49 (s, 0.75H), 7.91 (d, J = 7.2 Hz, 0.25H), 7.67 (d, J = 7.8 Hz, 0.75H), 7.52 (dd, J = 25.2, 6.6 Hz, 1H), 7.38 (dd, J = 19.2, 12.0 Hz, 1H), 7.22–7.16 (m, 0.25H), 7.09–6.95 (m, 2H), 6.92 (d, J = 8.4 Hz, 0.75H), 6.80 (dd, J = 18.0, 10.8 Hz, 1H), 5.84 (dd, J = 8.4, 4.8 Hz, 0.75H), 5.51 (s, 0.25H), 4.25 (d, J = 62.4 Hz, 5H), 4.06 (dd, J = 24.6, 8.4 Hz, 3.5H), 3.92 (d, J = 12.0 Hz, 0.5H), 3.80–3.69 (m, 1H), 3.58 (dd, J = 15.0, 9.0 Hz, 1H), 2.57 (s, 0.75H), 2.07 (s, 2.25H), 1.38 (d, J = 4.2 Hz, 9H), 1.16 (d, J = 16.2 Hz, 3H). ^{13}C NMR (150 MHz, $CDCl_3$) δ=206.6, 205.3, 162.7, 148.5, 147.7, 136.2, 136.0, 135.5, 134.9, 134.2, 133.4, 132.7, 131.1, 130.6, 130.1, 126.0, 125.6, 120.0, 119.8, 118.8, 118.5, 93.2, 92.7, 83.8, 83.6, 69.6, 69.5, 69.2, 69.1, 68.9, 68.0, 67.2, 66.4, 65.9, 43.6, 40.8, 39.5, 36.8, 25.5, 25.1, 25.0, 24.9, 21.5, 21.2. HRMS (ESI) m/z calcd for $C_{32}H_{35}BFeO_4^+$ $(M)^+$ 550.19723, found 550.19769.

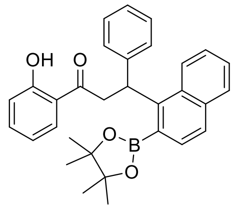

1-(2-hydroxyphenyl)-3-phenyl-3-(2-(4,4,5,5-tetramethyl-1,3,2-dioxaborolan-2-yl) naphthalen-1-yl)propan-1-one (132):

Yield 66%; 31.5 mg; yellow solid; mp 130–134°C; TLC (PET:EtOAc, 100:1 v/v): R_f = 0.3; ^{1}H NMR (600 MHz, $CDCl_3$) δ=12.40 (s, 1H), 7.83 (d, *J* = 36.0 Hz, 1H), 7.79 (d, *J* = 8.4 Hz, 1H), 7.74 (d, *J* = 8.4 Hz, 1H), 7.70 (d, *J* = 8.4 Hz, 1H), 7.61 (d, *J* = 7.2 Hz, 1H), 7.32 (t, *J* = 7.8 Hz, 2H), 7.20 (dt, *J* = 15.0, 7.8 Hz, 5H), 7.10 (t, *J* = 7.2 Hz, 1H), 6.91 (d, *J* = 8.4 Hz, 1H), 6.68 (t, *J* = 7.8 Hz, 1H), 6.24–6.18 (m, 1H), 4.39 (s, 1H), 3.71 (dd, *J* = 17.4, 4.8 Hz, 1H), 1.28 (s, 12H). ^{13}C NMR (150 MHz, $CDCl_3$) δ=204.9, 162.6, 147.5, 144.3, 136.3, 136.1, 131.4, 131.0, 130.0, 129.2, 128.4, 127.3, 127.0, 126.1, 125.7, 125.6, 119.5, 118.9, 118.5, 84.1, 42.7, 25.0, 24.9. HRMS (ESI) m/z calcd for $C_{31}H_{32}BO_4^+$ $(M+H)^+$ 479.23882, found 479.23895.

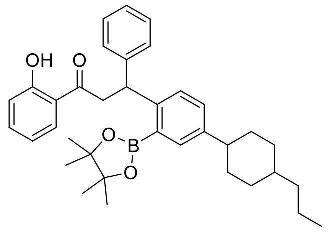

1-(2-hydroxyphenyl)-3-phenyl-3-(4-(4-propylcyclohexyl)-2-(4,4,5,5-tetramethyl-1,3,2-dioxaborolan-2-yl)phenyl)propan-1-one (133):

Yield 74%; 40.8 mg; white solid; mp 193–196°C; TLC (PET:EtOAc, 100:1 v/v): R_f = 0.3; ^{1}H NMR (600 MHz, $CDCl_3$) δ=12.28 (s, 1H), 7.80 (d, *J* = 7.8 Hz, 1H), 7.61 (s, 1H), 7.41 (t, *J* = 7.8 Hz, 1H), 7.29 (d, *J* = 7.8 Hz, 2H), 7.25 (t, *J* = 7.8 Hz, 2H), 7.15 (dd, *J* = 16.8, 8.4 Hz, 2H), 7.07 (d, *J* = 8.4 Hz, 1H), 6.93 (d, *J* = 8.4 Hz, 1H), 6.84 (t, *J* = 7.8 Hz, 1H), 5.65–5.61 (m, 1H), 3.78 (dd, *J* = 17.4, 8.4 Hz, 1H), 3.66 (dd, *J* = 16.8, 6.0 Hz, 1H), 2.43 (t, *J* = 12.0 Hz, 1H), 1.84 (t, *J* = 11.4 Hz, 4H), 1.41 (dd, *J* = 17.4, 9.0 Hz, 2H), 1.36–1.31 (m, 2H), 1.31–1.22 (m, 13H), 1.19 (dd, *J* = 15.0, 7.2 Hz, 2H), 1.00 (dd, *J* = 22.8, 11.4 Hz, 2H), 0.89 (t, *J* = 7.2 Hz, 3H). ^{13}C NMR (150 MHz, $CDCl_3$) δ=204.4, 162.4, 148.0, 144.8, 144.3, 136.0, 134.9, 129.9, 129.4, 128.3, 128.1, 127.2, 126.0, 119.5, 118.8, 118.4, 83.6, 45.3, 44.1, 43.0, 39.8, 37.0, 34.2, 33.6, 24.8, 24.8, 20.0, 14.5. HRMS (ESI) m/z calcd for $C_{36}H_{46}BO_4^+$ $(M+H)^+$ 553.34837, found 553.34808.

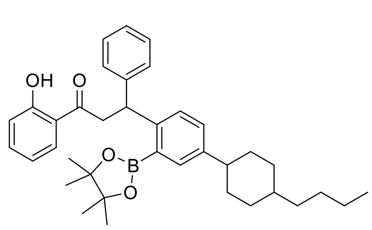

3-(4-(4-butylcyclohexyl)-2-(4,4,5,5-tetramethyl-1,3,2-dioxaborolan-2-yl)phenyl)-1-(2-hydroxyphenyl)-3-phenylpropan-1-one (134):

Yield 71%; 40.2 mg; white solid; mp 175–178℃; TLC (PET:EtOAc, 100:1 v/v): R_f = 0.3; ^{1}H NMR (600 MHz, $CDCl_3$) δ=12.28 (s, 1H), 7.76 (d, *J* = 7.8 Hz, 1H), 7.63 (s, 1H), 7.37 (t, *J* = 7.8 Hz, 1H), 7.28 (d, *J* = 7.8 Hz, 2H), 7.23 (t, *J* = 7.8 Hz, 2H), 7.17–7.10 (m, 2H), 7.07 (d, *J* = 7.8 Hz, 1H), 6.91 (d, *J* = 8.4 Hz, 1H), 6.80 (t, *J* = 7.8 Hz, 1H), 5.64 (t, *J* = 7.2 Hz, 1H), 3.71 (ddd, *J* = 22.8, 17.4, 7.2 Hz, 2H), 2.42 (t, *J* = 12.0 Hz, 1H), 1.84 (s, 4H), 1.47–1.39 (m, 2H), 1.35–1.11 (m, 19H), 1.00 (dd, *J* = 22.8, 11.4 Hz, 2H), 0.89 (s, 3H). ^{13}C NMR (150 MHz, $CDCl_3$) δ=204.5, 162.5, 148.0, 144.8, 144.4, 136.1, 135.0, 130.0, 129.4, 128.4, 128.2, 127.3, 126.1, 119.6, 118.8, 118.5, 83.7, 45.3, 44.2, 43.1, 37.3, 37.2, 34.3, 33.7, 29.3, 24.9, 24.9, 23.1, 14.3. HRMS (ESI) m/z calcd for $C_{37}H_{48}BO_4^+$ $(M+H)^+$ 567.36402, found 567.36377.

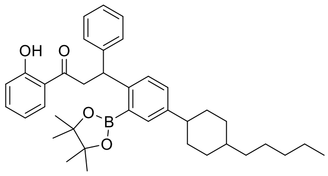

1-(2-hydroxyphenyl)-3-(4-(4-pentylcyclohexyl)-2-(4,4,5,5-tetramethyl-1,3,2-dioxaborolan-2-yl)phenyl)-3-phenylpropan-1-one (135):

Yield 59%; 34.2 mg; white solid; mp 90–93℃; TLC (PET:EtOAc, 100:1 v/v): R_f = 0.3; ^{1}H NMR (600 MHz, $CDCl_3$) δ=12.28 (s, 1H), 7.80 (d, *J* = 7.2 Hz, 1H), 7.61 (s, 1H), 7.41 (t, *J* = 7.2 Hz, 1H), 7.32–7.23 (m, 4H), 7.15 (dd, *J* = 15.6, 7.8 Hz, 2H), 7.07 (d, *J* = 8.4 Hz, 1H), 6.93 (d, *J* = 8.4 Hz, 1H), 6.84 (t, *J* = 7.8 Hz, 1H), 5.65–5.60 (m, 1H), 3.78 (dd, *J* = 17.4, 8.4 Hz, 1H), 3.66 (dd, *J* = 17.4, 6.0 Hz, 1H), 2.43 (t, *J* = 12.0 Hz, 1H), 1.84 (s, 4H), 1.41 (dd, *J* = 17.4, 8.4 Hz, 2H), 1.31–1.19 (m, 21H), 1.00 (dd, *J* = 23.4, 11.4 Hz, 2H), 0.88 (t, *J* = 7.2 Hz, 3H). ^{13}C NMR (150 MHz, $CDCl_3$) δ=204.4, 162.4, 147.9, 144.8, 144.3, 136.0, 134.9, 129.9, 129.4, 128.3, 128.1, 127.2, 126.0, 119.5, 118.7, 118.4, 83.6, 45.3, 44.1, 43.0, 37.4, 37.3, 34.2, 33.6, 32.2, 26.7, 24.8, 24.8, 22.7, 14.2. HRMS (ESI) m/z calcd for $C_{38}H_{50}BO_4^+$ $(M+H)^+$ 581.37967, found 581.37952.

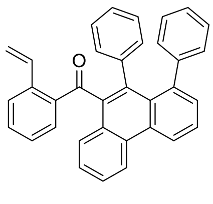

(1,10-diphenylphenanthren-9-yl)(2-vinylphenyl)methanone (136):

Yield 90%; 41.4 mg; white solid; mp 192–195 ℃; TLC (PET:EtOAc, 100:1 v/v): R_f = 0.3; ^{1}H NMR (600 MHz, $CDCl_3$) δ=8.83 (d, *J* = 7.8 Hz, 2H), 7.86 (d, *J* = 7.8 Hz, 1H), 7.69 (d, *J* = 6.6 Hz, 2H), 7.56 (d, *J* = 7.2 Hz, 1H), 7.38 (d, *J* = 6.6 Hz, 1H), 7.26–7.06 (m, 3H), 7.01–6.71 (m, 9H), 6.61 (s, 2H), 6.51–6.36 (m, 1H), 5.42 (d, *J* = 17.4 Hz, 1H), 5.17 (d, *J* = 10.8 Hz, 1H). ^{13}C NMR (150 MHz, $CDCl_3$) δ=200.8, 143.4, 142.7, 139.2, 138.9, 136.5, 136.3, 135.6, 132.4, 131.9, 131.2, 129.9, 129.0, 128.9, 127.6,

127.3, 127.1, 126.9, 126.6, 126.5, 126.3, 126.1, 125.7, 123.3, 122.1, 115.5. HRMS (ESI) m/z calcd for $C_{35}H_{25}O+$ $(M+H)^+$ 461.18999, found 461.19237.

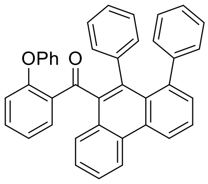

(1,10-diphenylphenanthren-9-yl)(2-phenoxyphenyl)methanone (137):

Yield 85%; 44.7 mg; white solid; mp 153–156°C; TLC (PET:EtOAc, 50:1 v/v): R_f = 0.3; ^1H NMR (600 MHz, $CDCl_3$) δ=8.69 (dd, *J* = 18.0, 8.4 Hz, 2H), 7.76 (d, *J* = 7.8 Hz, 1H), 7.59 (dd, *J* = 34.2, 7.2 Hz, 2H), 7.42 (d, *J* = 7.8 Hz, 1H), 7.35 (d, *J* = 7.2 Hz, 1H), 7.29 (d, *J* = 7.8 Hz, 1H), 7.11 (d, *J* = 7.8 Hz, 1H), 6.98 (t, *J* = 7.2 Hz, 2H), 6.85 (d, *J* = 12.6 Hz, 6H), 6.75 (dd, *J* = 28.2, 21.0 Hz, 5H), 6.51 (d, *J* = 8.4 Hz, 2H), 6.21 (d, *J* = 7.8 Hz, 2H). ^{13}C NMR (150 MHz, $CDCl_3$) δ=198.1, 155.9, 155.2, 143.6, 142.5, 140.1, 139.4, 134.1, 133.4, 132.3, 131.5, 131.4, 130.5, 130.0, 129.1, 129.1, 128.4, 127.0, 126.8, 126.2, 126.0, 126.0, 125.6, 123.1, 122.8, 122.7, 121.8, 118.8, 117.7. HRMS (ESI) m/z calcd for $C_{39}H_{27}O_2+$ $(M+H)^+$ 527.20056, found 527.20060.

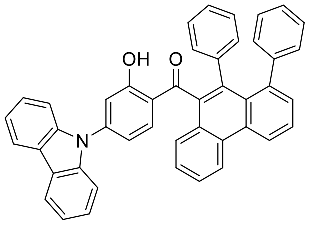

(4-(9H-carbazol-9-yl)-2-hydroxyphenyl)(1,10-diphenylphenanthren-9-yl)methanone (138):

Yield 93%; 57.2 mg; yellow solid; mp 205–208 °C; TLC (PET:EtOAc, 100:1 v/v): R_f = 0.3; ^1H NMR (600 MHz, $CDCl_3$) δ=12.14 (s, 1H), 8.86 (d, *J* = 8.4 Hz, 2H), 8.01 (d, *J* = 7.8 Hz, 2H), 7.83 (d, *J* = 7.8 Hz, 1H), 7.74 (s, 2H), 7.62 (t, *J* = 7.2 Hz, 1H), 7.47 (d, *J* = 6.6 Hz, 1H), 7.33 (s, 4H), 7.22 (s, 2H), 7.13 (d, *J* = 7.2 Hz, 1H), 7.07 (d, *J* = 7.2 Hz, 1H), 7.02–6.93 (m, 2H), 6.87 (d, *J* = 19.8 Hz, 4H), 6.81 (s, 1H), 6.74 (t, *J* = 6.6 Hz, 1H), 6.66–6.55 (m, 3H). ^{13}C NMR (150 MHz, $CDCl_3$) δ=203.9, 163.1, 144.3, 143.0, 142.6, 139.3, 138.9, 135.8, 135.5, 134.2, 132.2, 131.7, 130.8, 130.6, 129.6, 128.7, 128.4, 127.9, 127.4, 127.2, 126.8, 126.7, 126.6, 126.3, 125.8, 125.6, 123.6, 123.2, 121.8, 120.4, 120.0, 118.7, 115.9, 114.1, 109.8. HRMS (ESI) m/z calcd for $C_{45}H_{30}NO_2+$ $(M+H)^+$ 616.22711, found 616.22715.

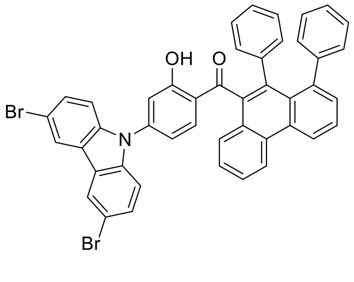

(4-(3,6-dibromo-9H-carbazol-9-yl)-2-hydroxyphenyl)(1,10-diphenylphenanthren-9-yl)methanone (139):

Yield 95%; 73.2 mg; yellow solid; mp 221–223 °C; TLC (PET:EtOAc, 100:1 v/v): R_f = 0.3; ^{1}H NMR (600 MHz, $CDCl_3$) δ=12.14 (s, 1H), 8.82 (d, *J* = 8.4 Hz, 2H), 7.97 (s, 2H), 7.79 (d, *J* = 7.8 Hz, 1H), 7.71 (s, 2H), 7.59 (t, *J* = 7.2 Hz, 1H), 7.44 (d, *J* = 6.6 Hz, 1H), 7.35 (d, *J* = 8.4 Hz, 2H), 7.16–7.01 (m, 4H), 6.95 (d, *J* = 6.6 Hz, 1H), 6.93–6.79 (m, 5H), 6.79–6.70 (m, 2H), 6.64–6.53 (m, 2H), 6.42 (d, *J* = 8.4 Hz, 1H). ^{13}C NMR (150 MHz, $CDCl_3$) δ=204.3, 163.3, 143.3, 143.1, 142.8, 139.1, 138.5, 136.0, 135.5, 134.6, 132.5, 132.0, 131.0, 130.8, 129.8, 129.4, 128.9, 128.5, 128.1, 127.7, 127.5, 127.1, 126.9, 126.8, 126.6, 125.9, 125.7, 124.2, 123.5, 123.1, 122.0, 119.3, 115.8, 114.3, 113.7, 111.6. HRMS (ESI) m/z calcd for $C_{45}H_{27}Br_2NO_2$+ $(M)^+$ 773.03877, found 773.03834.

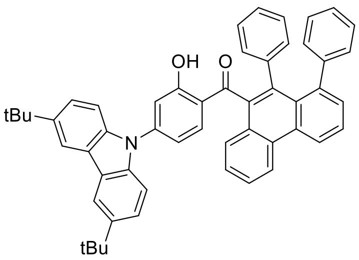

(4-(3,6-di-tert-butyl-9H-carbazol-9-yl)-2-hydroxyphenyl)(1,10-diphenylphenanthren-9-yl)methanone (140):

Yield 92%; 66.9 mg; yellow solid; mp 187–191 °C; TLC (PET:EtOAc, 100:1 v/v): R_f = 0.3; ^{1}H NMR (600 MHz, $CDCl_3$) δ=12.16 (s, 1H), 8.85 (d, *J* = 8.4 Hz, 2H), 8.06 (s, 2H), 7.84 (d, *J* = 7.8 Hz, 1H), 7.73 (dd, *J* = 13.2, 7.2 Hz, 2H), 7.62 (d, *J* = 7.2 Hz, 1H), 7.46 (d, *J* = 7.2 Hz, 1H), 7.39 (d, *J* = 8.4 Hz, 2H), 7.31 (d, *J* = 8.4 Hz, 2H), 7.13 (d, *J* = 7.2 Hz, 1H), 7.07 (d, *J* = 6.6 Hz, 1H), 6.97 (dd, *J* = 16.2, 9.6 Hz, 2H), 6.87 (dd, *J* = 26.4, 7.2 Hz, 4H), 6.81 (d, *J* = 6.6 Hz, 1H), 6.75 (t, *J* = 7.2 Hz, 1H), 6.62 (dd, *J* = 26.4, 7.2 Hz, 3H), 1.42 (s, 18H). ^{13}C NMR (150 MHz, $CDCl_3$) δ=204.0, 163.5, 145.1, 143.7, 143.2, 142.8, 139.1, 137.9, 136.0, 135.8, 134.3, 132.5, 132.0, 131.0, 130.8, 129.8, 128.9, 128.7, 128.2, 127.7, 127.4, 127.0, 126.9, 126.8, 126.7, 126.6, 125.9, 125.8, 123.9, 123.7, 123.4, 122.0, 118.6, 116.2, 115.7, 113.6, 109.6, 34.7, 31.9. HRMS (ESI) m/z calcd for $C_{53}H_{46}NO_2$+ $(M+H)^+$ 728.35231, found 728.35134.

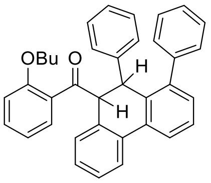

(2-butoxyphenyl)(1,10-diphenyl-9,10-dihydrophenanthren-9-yl)methanone (141):

white solid; mp 125–128 °C; TLC (PET:EtOAc, 30:1 v/v): R_f = 0.3; ^{1}H NMR (600 MHz, $CDCl_3$) δ=7.85 (dd, *J* = 14.4, 7.8 Hz, 2H), 7.36 (t, *J* = 7.8 Hz, 1H), 7.27 (d, *J* = 4.2 Hz, 2H), 7.16–7.10 (m, 2H), 7.08–7.03 (m, 3H), 6.98 (s, 4H), 6.93 (d, *J* = 7.2 Hz, 1H), 6.80 (dd, *J* = 13.8, 7.8 Hz, 3H), 6.74 (d, *J* = 25.8 Hz, 3H), 4.92 (s, 1H), 4.65 (s,

1H), 4.03–3.91 (m, 2H), 1.70 (dd, J = 14.4, 7.2 Hz, 2H), 1.39 (dd, J = 13.8, 7.2 Hz, 2H), 0.89 (t, J = 7.2 Hz, 3H). ^{13}C NMR (150 MHz, $CDCl_3$) δ=203.0, 156.2, 143.1, 142.7, 140.9, 135.3, 134.8, 133.9, 132.3, 130.4, 129.9, 129.7, 129.0, 129.0, 128.1, 128.0, 127.9, 127.8, 127.5, 127.3, 126.7, 126.0, 124.0, 123.5, 120.9, 111.9, 68.3, 59.9, 43.2, 31.2, 19.4, 13.9. HRMS (ESI) m/z calcd for $C_{37}H_{32}O_2Na+$ $(M+Na)^+$ 531.22945, found 531.22948.

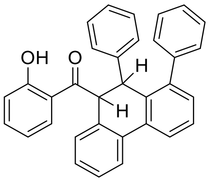

(1,10-diphenyl-9,10-dihydrophenanthren-9-yl)(2-hydroxyphenyl)methanone (142):

white solid; mp 139–143°C; TLC (PET:EtOAc, 20:1 v/v): R_f = 0.3; ^{1}H NMR (600 MHz, $CDCl_3$) δ=11.92 (s, 1H), 7.96 (dd, J = 15.6, 7.8 Hz, 3H), 7.46 (t, J = 7.8 Hz, 1H), 7.38 (dt, J = 15.6, 7.8 Hz, 2H), 7.15 (dd, J = 15.0, 7.8 Hz, 2H), 7.10 (d, J = 7.2 Hz, 1H), 7.00 (ddd, J = 24.0, 13.2, 5.4 Hz, 7H), 6.89 (t, J = 7.2 Hz, 1H), 6.75 (d, J = 3.0 Hz, 2H), 6.69 (d, J = 6.0 Hz, 2H), 4.94 (s, 1H), 4.67 (s, 1H). ^{13}C NMR (150 MHz, $CDCl_3$) δ=204.4, 163.2, 142.8, 142.2, 140.7, 136.5, 136.3, 134.7, 132.7, 131.1, 129.9, 129.7, 129.7, 128.9, 128.7, 128.4, 128.2, 127.7, 127.6, 126.9, 126.7, 124.3, 123.4, 119.2, 119.0, 117.6, 54.3, 44.9. HRMS (ESI) m/z calcd for $C_{33}H_{25}O_2+$ $(M+H)^+$ 453.18491, found 453.18501.

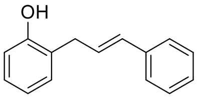

2-cinnamylphenol (143):

yellow oil; ^{1}H NMR (600 MHz, $CDCl_3$) δ=7.31 (d, J = 7.8 Hz, 2H), 7.25 (t, J = 7.8 Hz, 2H), 7.20–7.12 (m, 2H), 7.10 (t, J = 7.8 Hz, 1H), 6.88 (t, J = 7.2 Hz, 1H), 6.76 (d, J = 7.8 Hz, 1H), 6.46 (d, J = 16.2 Hz, 1H), 6.36 (dd, J = 14.4, 7.8 Hz, 1H), 5.13 (s, 1H), 3.52 (d, J = 6.6 Hz, 2H). ^{13}C NMR (150 MHz, $CDCl_3$) δ=153.9, 137.2, 131.5, 130.5, 128.6, 128.0, 127.9, 127.4, 126.3, 125.9, 121.1, 115.8, 34.0. HRMS (ESI) m/z calcd for $C_{15}H_{15}O^+$ $(M+H)^+$ 211.11174, found 211.11148.

4.7 Crystallographic data and molecular structure of compounds

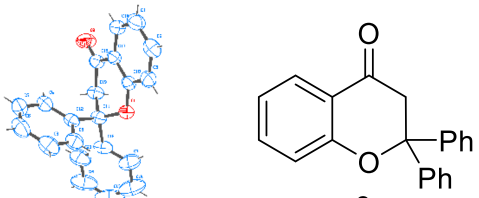

3

Supplementary Figure 13. Crystal structure. The X-ray crystal structure of **3.**

Crystal Data for Compound **3**: CCDC 1970136 contains the supplementary crystallographic data for this paper. These data can be obtained free of charge from The Cambridge Crystallographic.

Bond precision: C-C = 0.0034 A Wavelength=0.71073

Cell:	a=13.205(11)	b=11.829(10)	c=20.597(17)
	alpha=90	beta=95.475(12)	gamma=90
Temperature:	296 K		

	Calculated	Reported
Volume	3203(5)	3203(5)
Space group	C 2/c	C 1 2/c 1
Hall group	-C 2yc	-C 2yc
Moiety formula	C21 H16 O2	C21 H16 O2
Sum formula	C21 H16 O2	C21 H16 O2
Mr	300.34	300.34
Dx,g cm-3	1.246	1.246
Z	8	8
Mu (mm-1)	0.079	0.079
F000	1264.0	1264.0
F000'	1264.57	
h,k,lmax	19,17,30	19,17,29
Nref	5380	4884
Tmin,Tmax		0.622,0.746
Tmin'		

Correction method= # Reported T Limits: Tmin=0.622 Tmax=0.746
AbsCorr = MULTI-SCAN

Data completeness= 0.908 Theta(max)= 31.619

R(reflections)= 0.0729(3587) wR2(reflections)= 0.1953(4884)

S = 1.042 Npar= 209

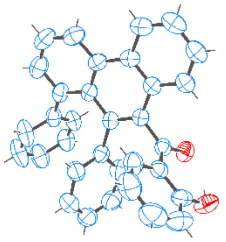

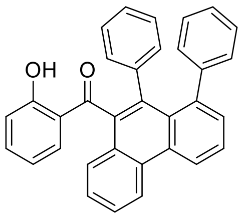

4

Supplementary Figure 14. Crystal structure. The X-ray crystal structure of **4.**

Crystal Data for Compound **4**: CCDC 1964456 contains the supplementary crystallographic data for this paper. These data can be obtained free of charge from The Cambridge Crystallographic.

```
Bond precision:   C-C = 0.0044 A          Wavelength=0.71073

Cell:              a=9.9499(19)       b=10.724(2)       c=22.211(4)
                   alpha=90           beta=90           gamma=90
Temperature:       273 K

                 Calculated                  Reported
Volume           2370.0(8)                   2370.0(8)
Space group      P 21 21 21                  P 21 21 21
Hall group       P 2ac 2ab                   P 2ac 2ab
Moiety formula   C33 H22 O2                  ?
Sum formula      C33 H22 O2                  C33 H22 O2
Mr               450.51                      450.50
Dx,g cm-3        1.263                       1.263
Z                4                           4
Mu (mm-1)        0.077                       0.077
F000             944.0                       944.0
F000'            944.41
h,k,lmax         12,13,28                    12,13,28
Nref             5388[ 3050]                 5363
Tmin,Tmax        0.983,0.986                 0.864,0.864
Tmin'            0.983

Correction method= # Reported T Limits: Tmin=0.864 Tmax=0.864
AbsCorr = MULTI-SCAN

Data completeness= 1.76/1.00       Theta(max)= 27.426

R(reflections)= 0.0435( 3852)      wR2(reflections)= 0.1153( 5363)

S = 0.968                   Npar= 319
```

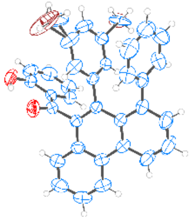

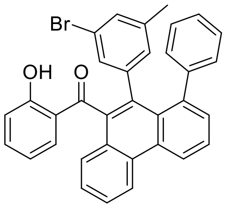

44

Supplementary Figure 15. Crystal structure. The X-ray crystal structure of **44.**

Crystal Data for Compound **44**: CCDC 1961529 contains the supplementary crystallographic data for this paper. These data can be obtained free of charge from The Cambridge Crystallographic.

Bond precision:	C-C = 0.0042 A		Wavelength=0.71073
Cell:	a=13.325(3)	b=9.7824(19)	c=20.708(4)
	alpha=90	beta=98.532(3)	gamma=90
Temperature:	296 K		

	Calculated	Reported
Volume	2669.4(9)	2669.4(9)
Space group	P 21/c	P 1 21/c 1
Hall group	-P 2ybc	-P 2ybc
Moiety formula	C34 H23 Br O2	C34 H23 Br O2
Sum formula	C34 H23 Br O2	C34 H23 Br O2
Mr	543.42	543.43
Dx,g cm-3	1.352	1.352
Z	4	4
Mu (mm-1)	1.569	1.569
F000	1112.0	1112.0
F000’	1111.27	
h,k,lmax	18,13,28	18,13,28
Nref	7036	6958
Tmin,Tmax		0.643,0.746
Tmin’		

Correction method= # Reported T Limits: Tmin=0.643 Tmax=0.746
AbsCorr = MULTI-SCAN

Data completeness= 0.989 Theta(max)= 28.909

R(reflections)= 0.0651(3938) wR2(reflections)= 0.1908(6958)

S = 1.029 Npar= 356

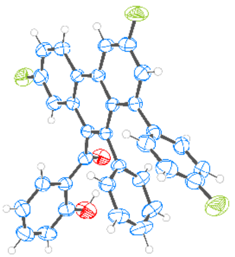

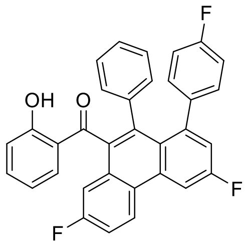

50

Supplementary Figure 16. Crystal structure. The X-ray crystal structure of **50.**

Crystal Data for Compound **50**: CCDC 1961532 contains the supplementary crystallographic data for this paper. These data can be obtained free of charge from The Cambridge Crystallographic.

Bond precision: C-C = 0.0026 A Wavelength=0.71073

Cell:	a=10.524(2)	b=10.546(2)	c=11.504(3)
	alpha=82.680(4)	beta=79.677(3)	gamma=69.908(3)
Temperature:	296 K		

	Calculated	Reported
Volume	1176.7(4)	1176.7(4)
Space group	P -1	P -1
Hall group	-P 1	-P 1
Moiety formula	C33 H19 F3 O2	?
Sum formula	C33 H19 F3 O2	C33 H19 F3 O2
Mr	504.48	504.48
Dx,g cm-3	1.424	1.424
Z	2	2
Mu (mm-1)	0.104	0.104
F000	520.0	520.0
F000'	520.30	
h,k,lmax	13,13,14	13,13,14
Nref	4711	4634
Tmin,Tmax	0.977,0.981	0.864,0.864
Tmin'	0.977	

Correction method= # Reported T Limits: Tmin=0.864 Tmax=0.864
AbsCorr = MULTI-SCAN

Data completeness= 0.984 Theta(max)= 26.184

R(reflections)= 0.0472(3383) wR2(reflections)= 0.1668(4634)

S = 0.994 Npar= 346

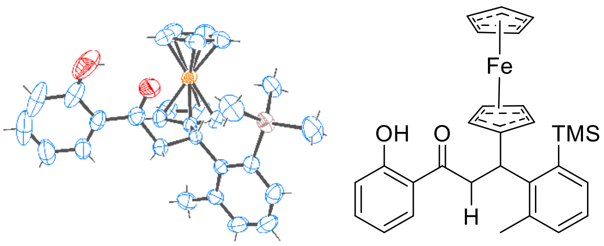

73

Supplementary Figure 17. Crystal structure. The X-ray crystal structure of **73.**

Crystal Data for Compound **73**: CCDC 1969923 contains the supplementary crystallographic data for this paper. These data can be obtained free of charge from The Cambridge Crystallographic.

Bond precision:	C-C = 0.0044 A		Wavelength=0.71073
Cell:	a=10.5887(16)	b=11.2010(17)	c=11.3216(17)
	alpha=83.063(2)	beta=88.838(2)	gamma=72.895(2)
Temperature:	296 K		

	Calculated	Reported
Volume	1273.8(3)	1273.8(3)
Space group	P -1	P -1
Hall group	-P 1	-P 1
Moiety formula	C29 H32 Fe O2 Si	C29 H32 Fe O2 Si
Sum formula	C29 H32 Fe O2 Si	C29 H32 Fe O2 Si
Mr	496.49	496.48
Dx,g cm-3	1.294	1.294
Z	2	2
Mu (mm-1)	0.662	0.662
F000	524.0	524.0
F000'	525.02	
h,k,lmax	15,16,16	15,16,16
Nref	8771	7786
Tmin,Tmax	0.924,0.936	0.545,0.746
Tmin'	0.924	

Correction method= # Reported T Limits: Tmin=0.545 Tmax=0.746
AbsCorr = MULTI-SCAN

Data completeness= 0.888   Theta(max)= 31.888

R(reflections)= 0.0590(6034)   wR2(reflections)= 0.1792(7786)

S = 1.116   Npar= 304

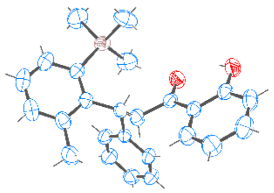

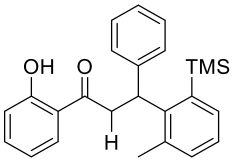

82

Supplementary Figure 18. Crystal structure. The X-ray crystal structure of **82.**

Crystal Data for Compound **82**: CCDC 1969916 contains the supplementary crystallographic data for this paper. These data can be obtained free of charge from The Cambridge Crystallographic.

Bond precision: C-C = 0.0035 A Wavelength=0.71073

Cell:	a=8.756(4)	b=9.898(4)	c=13.339(5)
	alpha=86.809(6)	beta=88.469(6)	gamma=70.617(6)
Temperature:	296 K		

	Calculated	Reported
Volume	1088.8(8)	1088.8(7)
Space group	P -1	P -1
Hall group	-P 1	-P 1
Moiety formula	C25 H28 O2 Si	?
Sum formula	C25 H28 O2 Si	C25 H28 O2 Si
Mr	388.56	388.56
Dx,g cm-3	1.185	1.185
Z	2	2
Mu (mm-1)	0.125	0.125
F000	416.0	416.0
F000'	416.32	
h,k,lmax	10,11,16	10,11,16
Nref	4050	3993
Tmin,Tmax	0.970,0.975	
Tmin'	0.963	

Correction method= Not given

Data completeness= 0.986 Theta(max)= 25.499

R(reflections)= 0.0559(3265) wR2(reflections)= 0.1817(3993)

S = 1.070 Npar= 259

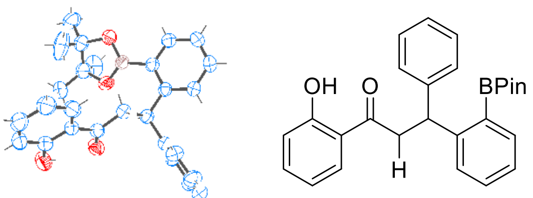

103

Supplementary Figure 19. Crystal structure. The X-ray crystal structure of **103.**

Crystal Data for Compound **103**: CCDC 1969930 contains the supplementary crystallographic data for this paper. These data can be obtained free of charge from The Cambridge Crystallographic.

```
Bond precision:   C-C = 0.0051 A          Wavelength=0.71073

Cell:             a=10.274(2)     b=24.480(5)        c=10.241(2)
                  alpha=90        beta=117.997(5)    gamma=90
Temperature:      296 K

                 Calculated                   Reported
Volume           2274.3(8)                    2274.2(9)
Space group      P 21/c                       P 1 21/c 1
Hall group       -P 2ybc                      -P 2ybc
Moiety formula   C27 H29 B O4                 C27 H29 B O4
Sum formula      C27 H29 B O4                 C27 H29 B O4
Mr               428.31                       428.31
Dx,g cm-3        1.251                        1.251
Z                4                            4
Mu (mm-1)        0.082                        0.082
F000             912.0                        912.0
F000'            912.42
h,k,lmax         12,29,12                     12,28,12
Nref             4008                         3894
Tmin,Tmax        0.990,0.992                  0.621,0.745
Tmin'            0.990

Correction method= # Reported T Limits: Tmin=0.621 Tmax=0.745
AbsCorr = MULTI-SCAN

Data completeness= 0.972          Theta(max)= 24.994

R(reflections)= 0.0638( 2523)     wR2(reflections)= 0.1681( 3894)

S = 1.029                  Npar= 294
```

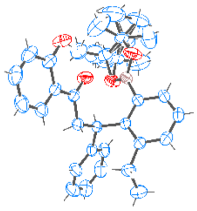

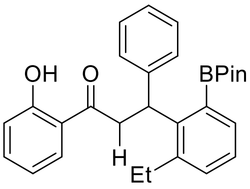

105

Supplementary Figure 20. Crystal structure. The X-ray crystal structure of **105.**

Crystal Data for Compound **105**: CCDC 1969931 contains the supplementary crystallographic data for this paper. These data can be obtained free of charge from The Cambridge Crystallographic.

Bond precision: C-C = 0.0028 A Wavelength=0.71073

Cell: a=9.9824(13) b=11.2719(14) c=12.8941(16)
alpha=76.855(2) beta=78.253(2) gamma=67.266(2)

Temperature: 291 K

	Calculated	Reported
Volume	1292.2(3)	1292.2(3)
Space group	P -1	P -1
Hall group	-P 1	-P 1
Moiety formula	C29 H33 B O4	C29 H33 B O4
Sum formula	C29 H33 B O4	C29 H33 B O4
Mr	456.36	456.36
Dx,g cm-3	1.173	1.173
Z	2	2
Mu (mm-1)	0.076	0.076
F000	488.0	488.0
F000'	488.22	
h,k,lmax	12,13,15	12,13,15
Nref	4811	4751
Tmin,Tmax	0.982,0.985	0.687,0.746
Tmin'	0.977	

Correction method= # Reported T Limits: Tmin=0.687 Tmax=0.746
AbsCorr = MULTI-SCAN

Data completeness= 0.988 Theta(max)= 25.500

R(reflections)= 0.0517(3966) wR2(reflections)= 0.1707(4751)

S = 1.055 Npar= 374

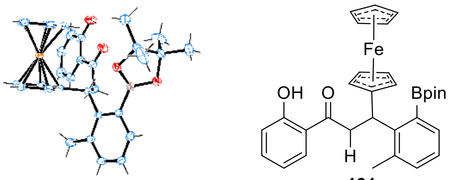

131

Supplementary Figure 21. Crystal structure. The X-ray crystal structure of **131.**

Crystal Data for Compound **131**: CCDC 1974984 contains the supplementary crystallographic data for this paper. These data can be obtained free of charge from The Cambridge Crystallographic.

Bond precision: C-C = 0.0032 A Wavelength=0.71073

Cell:	a=10.1618(13)	b=11.4078(15)	c=13.2712(17)
	alpha=88.528(2)	beta=79.496(2)	gamma=64.493(2)
Temperature:	296 K		

	Calculated	Reported
Volume	1362.8(3)	1362.8(3)
Space group	P -1	P -1
Hall group	-P 1	-P 1
Moiety formula	C32 H35 B Fe O4	C32 H35 B Fe O4
Sum formula	C32 H35 B Fe O4	C32 H35 B Fe O4
Mr	550.26	550.26
Dx,g cm-3	1.341	1.341
Z	2	2
Mu (mm-1)	0.589	0.589
F000	580.0	580.0
F000’	580.91	
h,k,lmax	12,13,16	12,13,16
Nref	5065	5002
Tmin,Tmax	0.932,0.943	0.644,0.746
Tmin’	0.932	

Correction method= # Reported T Limits: Tmin=0.644 Tmax=0.746
AbsCorr = MULTI-SCAN

Data completeness= 0.988 Theta(max)= 25.500

R(reflections)= 0.0376(4574) wR2(reflections)= 0.1197(5002)

S = 1.157 Npar= 350

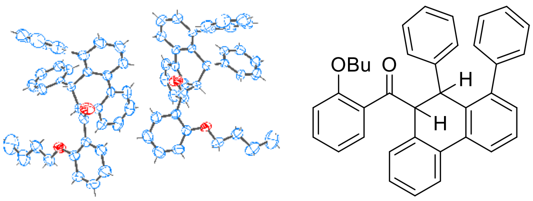

141

Supplementary Figure 22. Crystal structure. The X-ray crystal structure of **141.**

Crystal Data for Compound **141**: CCDC 1961844 contains the supplementary crystallographic data for this paper. These data can be obtained free of charge from The Cambridge Crystallographic.

Bond precision: C-C = 0.0051 A Wavelength=0.71073

Cell:	a=10.146(2)	b=14.310(3)	c=20.839(4)
	alpha=105.420(4)	beta=97.063(5)	gamma=104.487(4)
Temperature:	296 K		

	Calculated	Reported
Volume	2765.2(10)	2765.2(10)
Space group	P -1	P -1
Hall group	-P 1	-P 1
Moiety formula	C37 H32 O2	C37 H32 O2
Sum formula	C37 H32 O2	C37 H32 O2
Mr	508.63	508.62
Dx,g cm-3	1.222	1.222
Z	4	4
Mu (mm-1)	0.074	0.074
F000	1080.0	1080.0
F000'	1080.44	
h,k,lmax	11,16,24	11,16,24
Nref	9203	8883
Tmin,Tmax	0.991,0.993	0.603,0.745
Tmin'	0.991	

Correction method= # Reported T Limits: Tmin=0.603 Tmax=0.745
AbsCorr = MULTI-SCAN

Data completeness= 0.965 Theta(max)= 24.499

R(reflections)= 0.0596(4358) wR2(reflections)= 0.1423(8883)

S = 0.988 Npar= 705

4.8 ^{1}H and ^{13}C NMR spectra of compounds

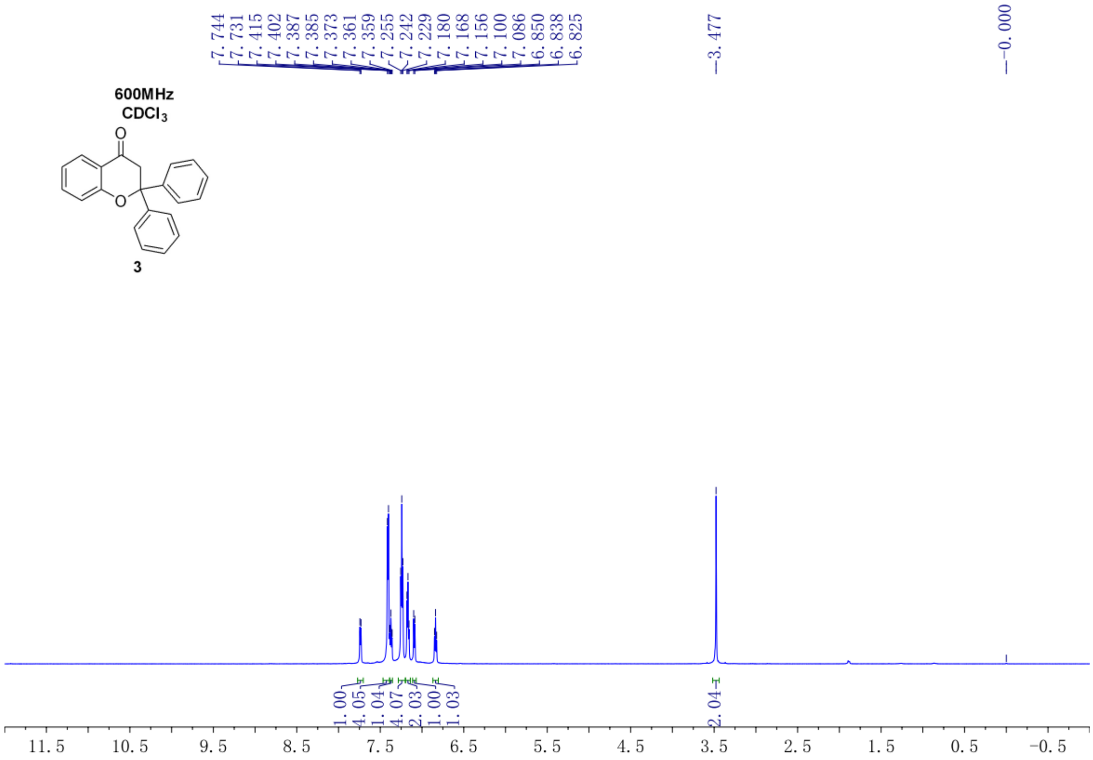

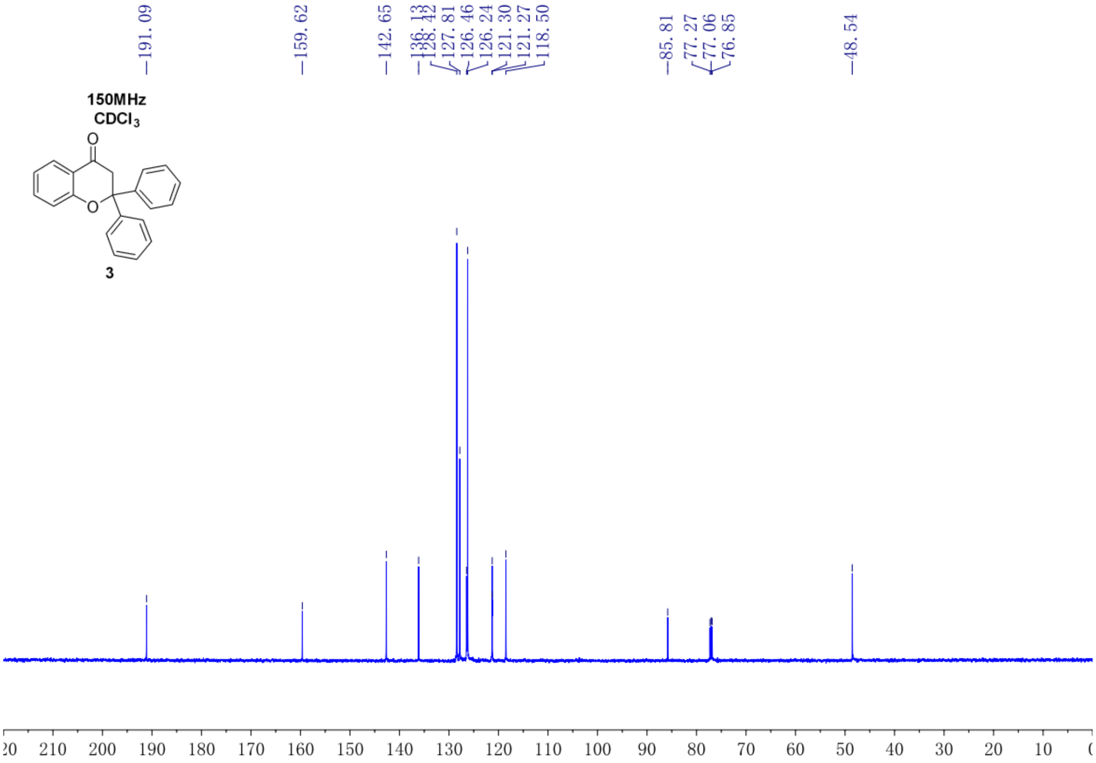

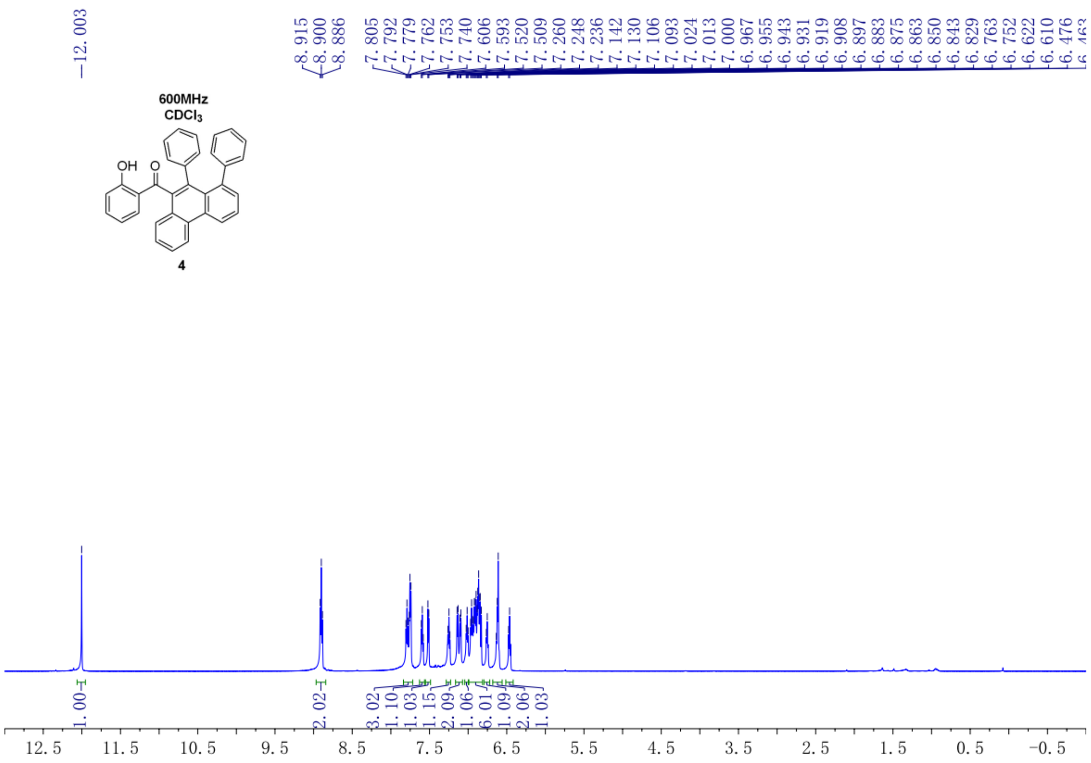
600MHz
CDCl3
OH
O
4

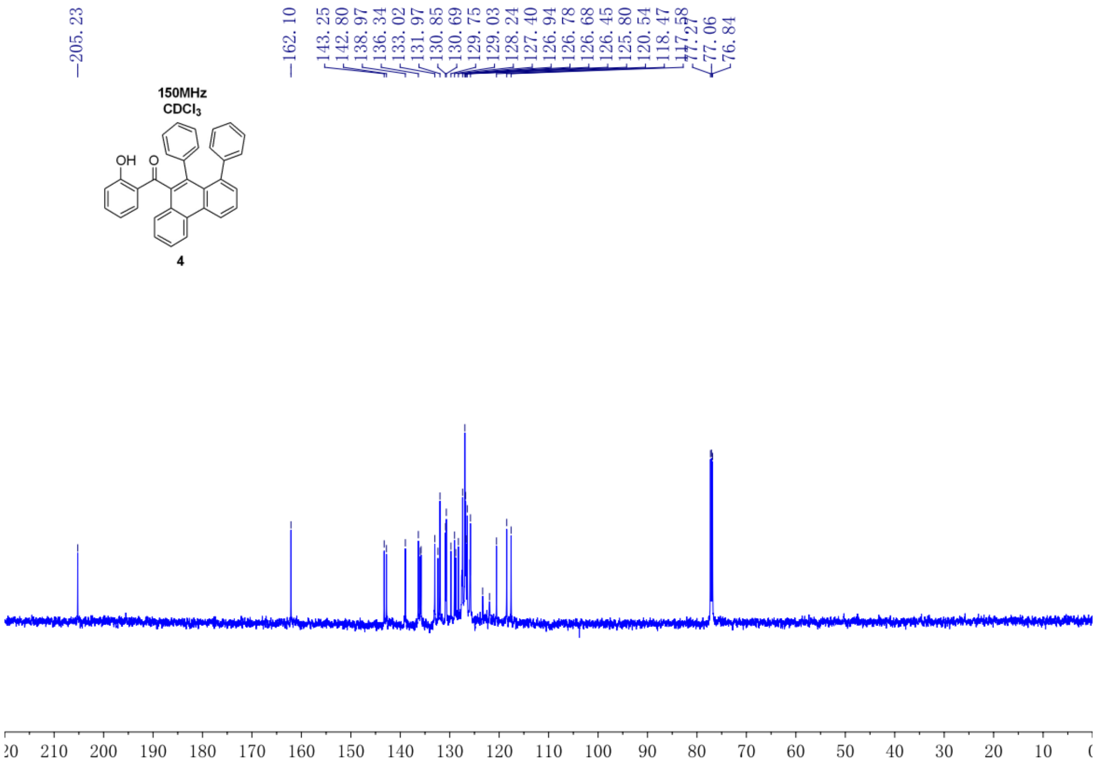
150MHz
CDCl3
OH
O
4

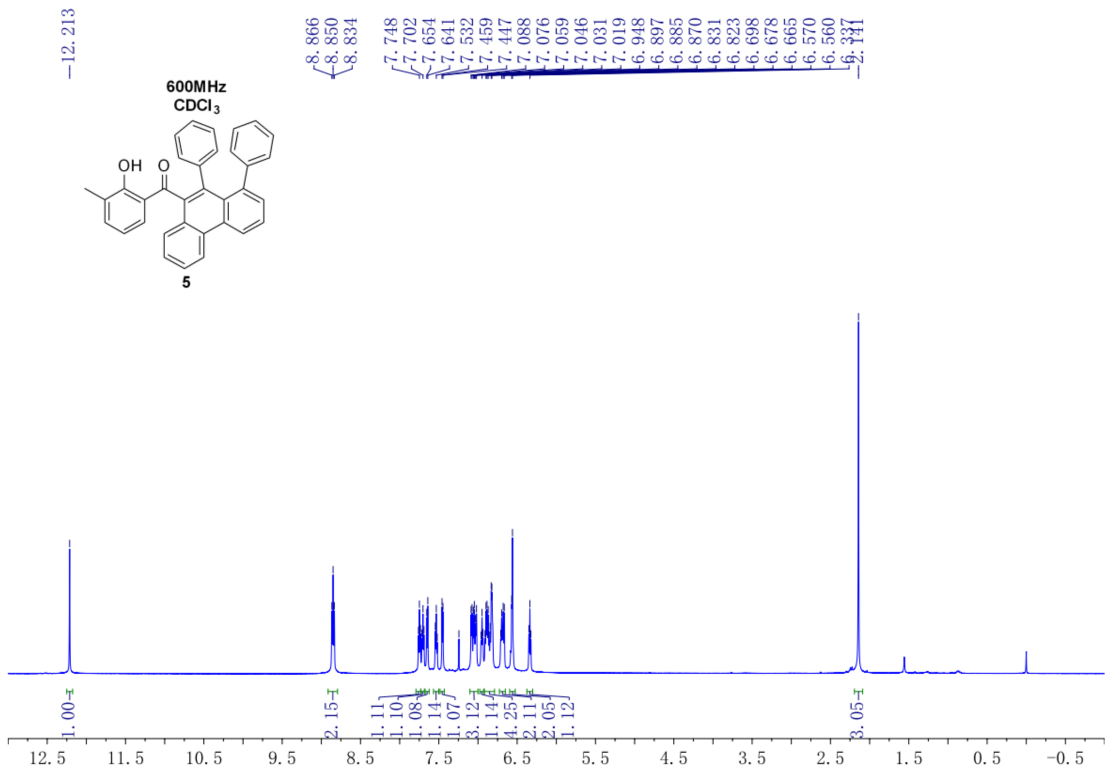

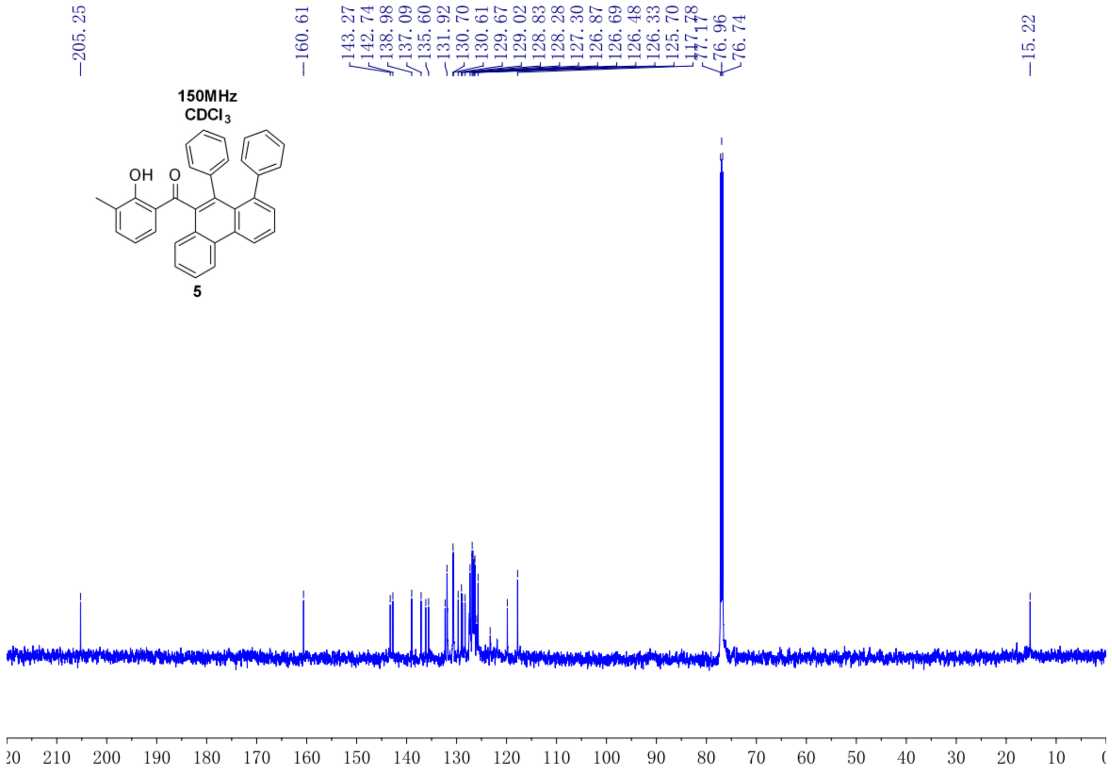

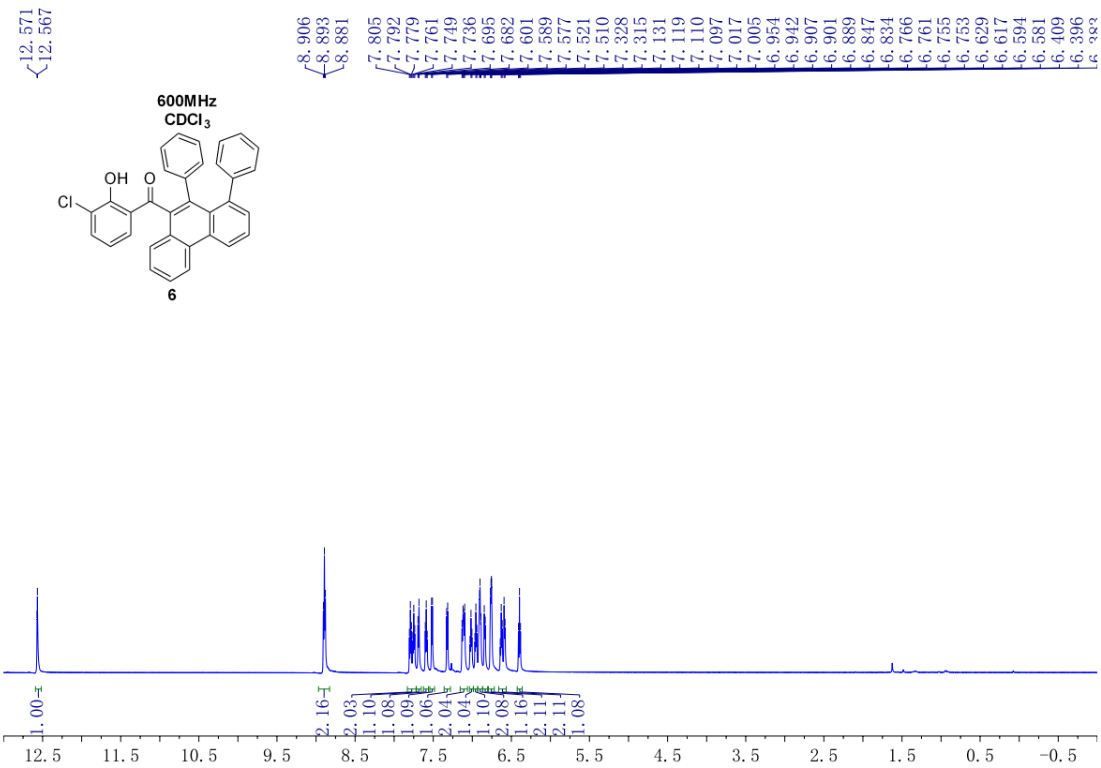

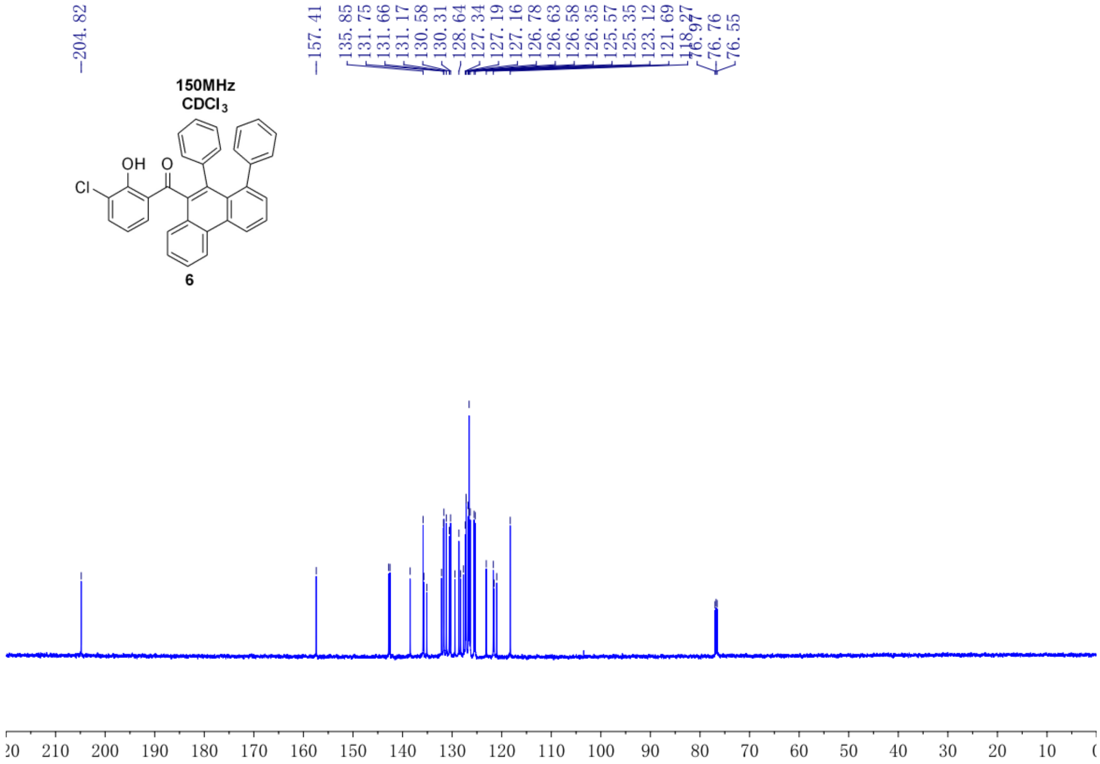

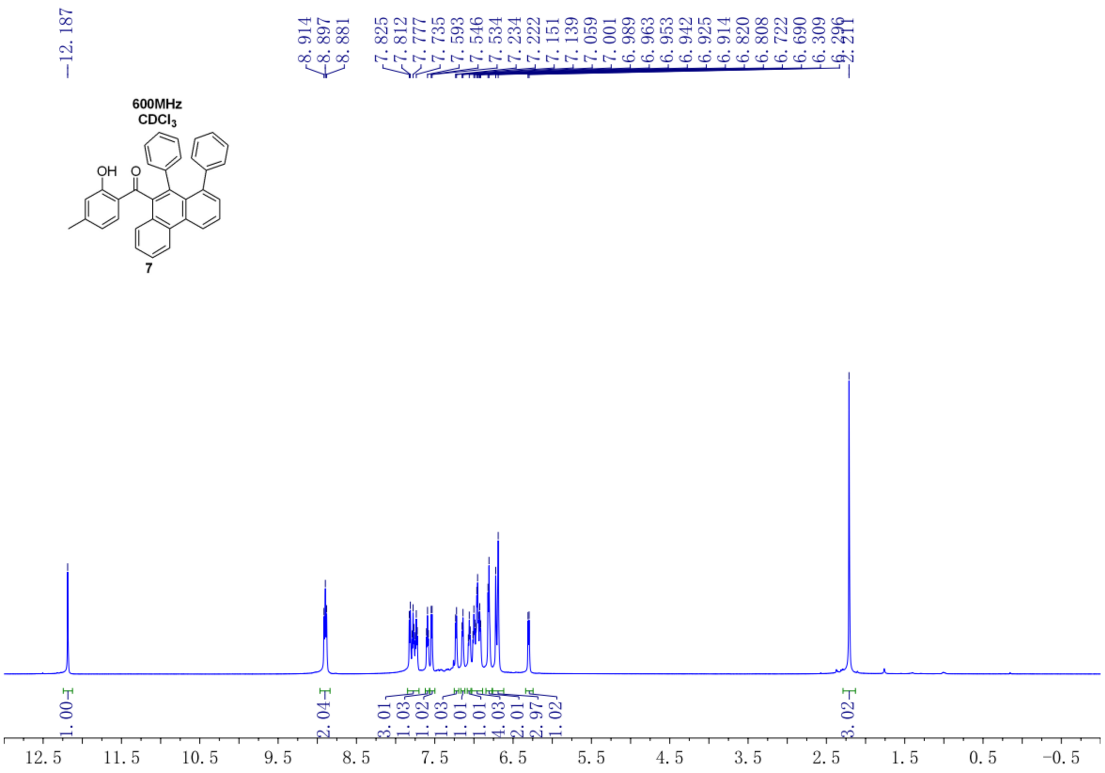
600MHz
CDCl3
7

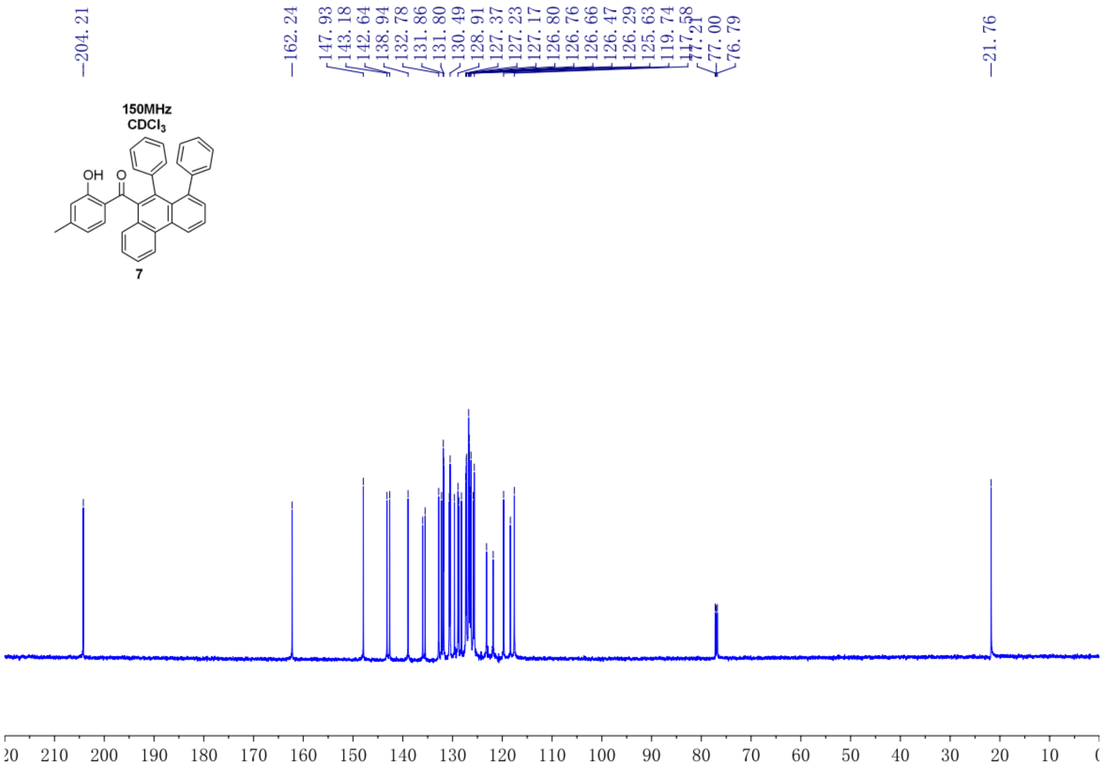
150MHz
CDCl3
7

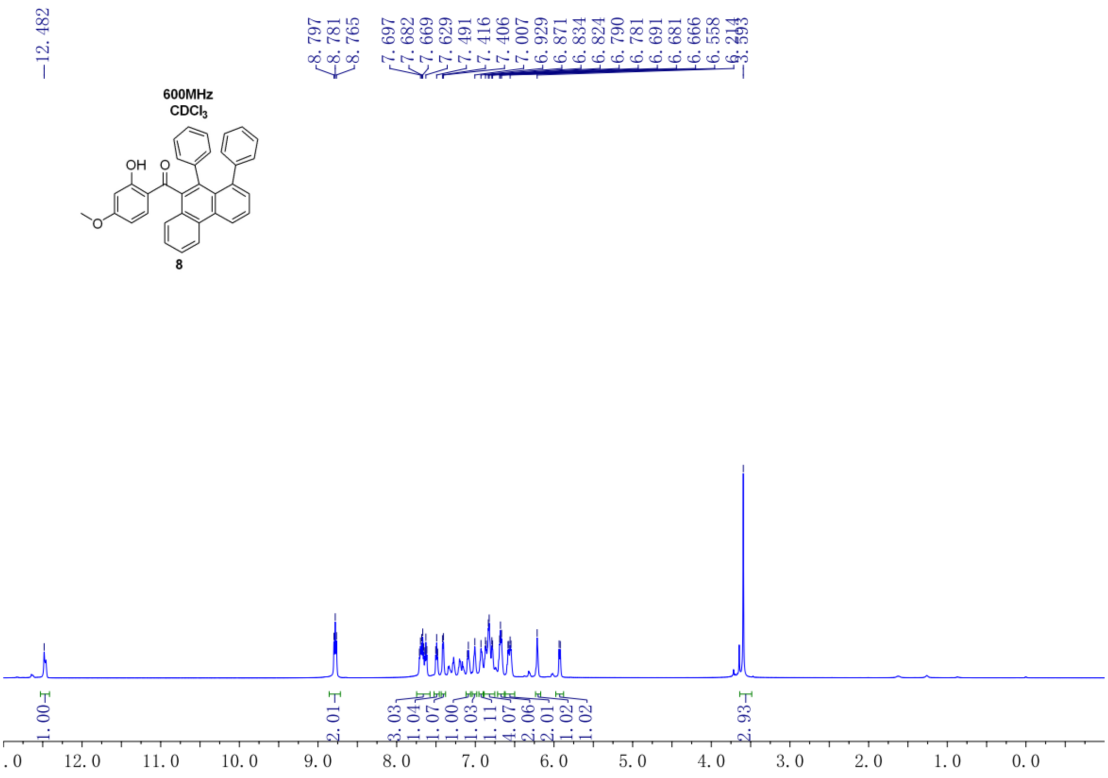
12.482
8.797
8.781
8.765
7.697
7.682
7.669
7.629
7.491
7.416
7.406
7.007
6.929
6.871
6.834
6.824
6.790
6.781
6.691
6.681
6.666
6.558
6.214
3.593
600MHz
CDCl3
OH
O
O
8
1.00
2.01
3.03
1.04
1.07
1.00
1.03
1.11
4.07
2.06
2.01
1.02
1.02
2.93
12.0 11.0 10.0 9.0 8.0 7.0 6.0 5.0 4.0 3.0 2.0 1.0 0.0

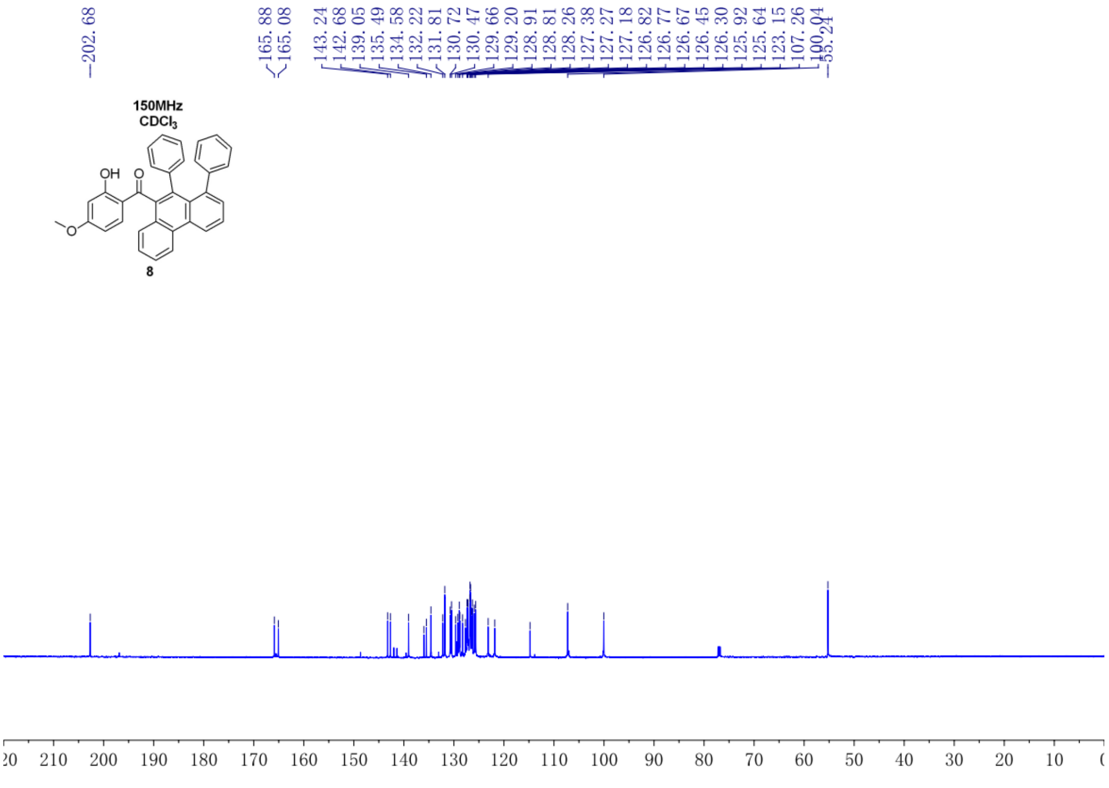
202.68
165.88
165.08
143.24
142.68
139.05
135.49
134.58
132.22
131.81
130.72
130.47
129.66
129.20
128.91
128.81
128.26
127.38
127.27
127.18
126.82
126.77
126.67
126.45
126.30
125.92
125.64
123.15
107.26
100.04
55.24
150MHz
CDCl3
OH
O
O
8
210 200 190 180 170 160 150 140 130 120 110 100 90 80 70 60 50 40 30 20 10

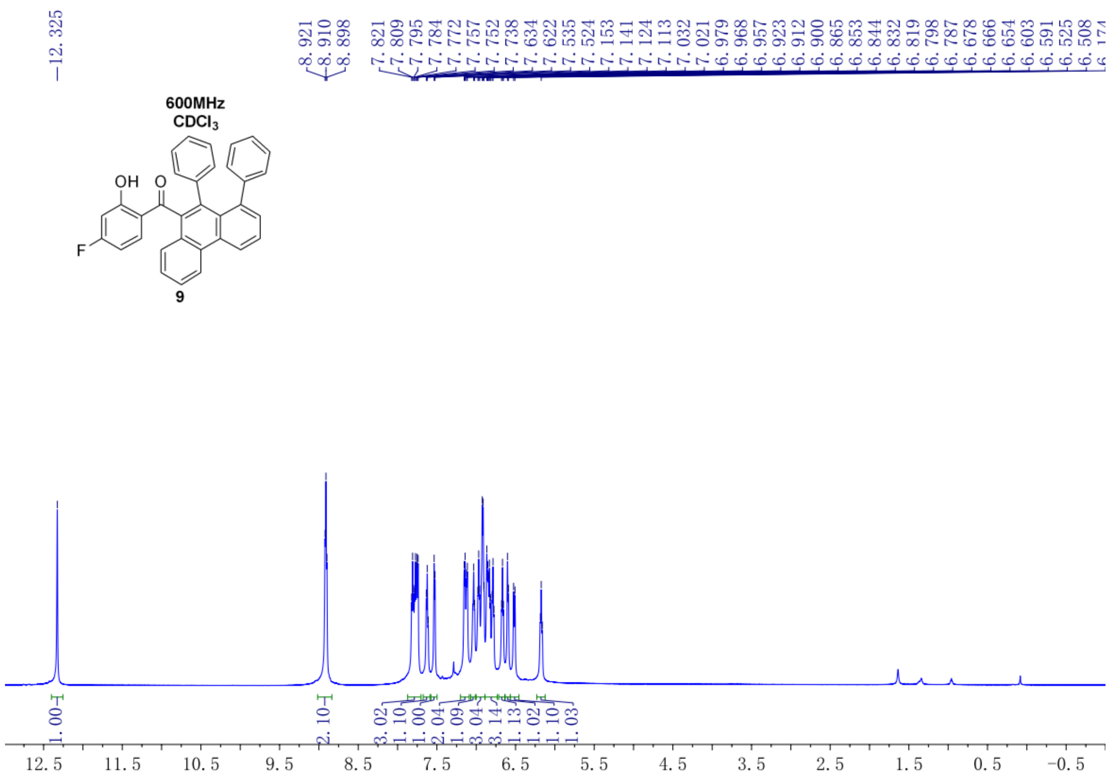
600MHz
CDCl3
OH
O
F
9

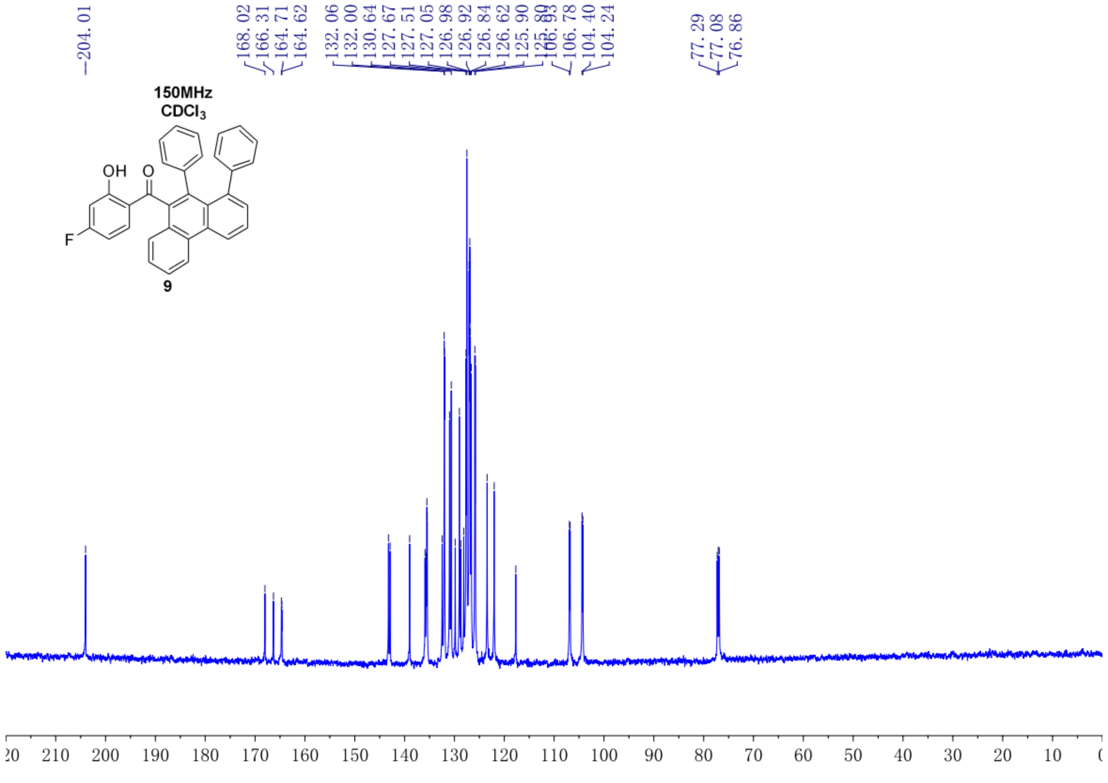
150MHz
CDCl3
OH
O
F
9

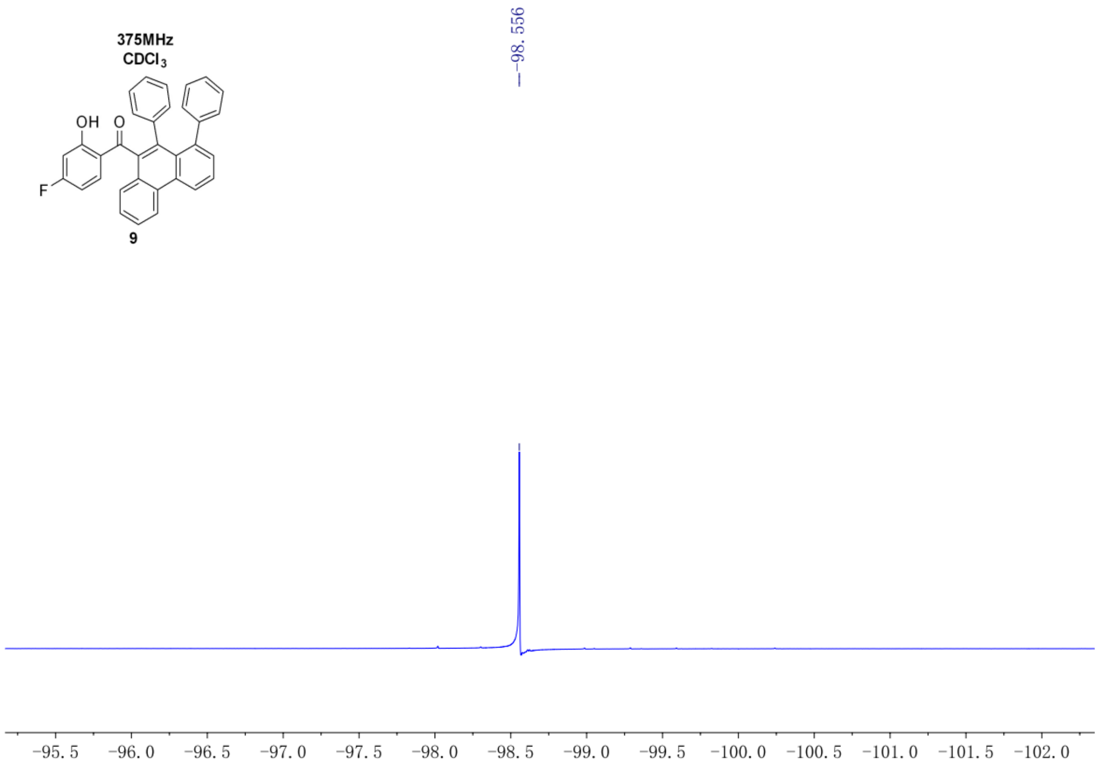
375MHz
CDCl3
OH
O
F
9
−98.556
−95.5 −96.0 −96.5 −97.0 −97.5 −98.0 −98.5 −99.0 −99.5 −100.0 −100.5 −101.0 −101.5 −102.0

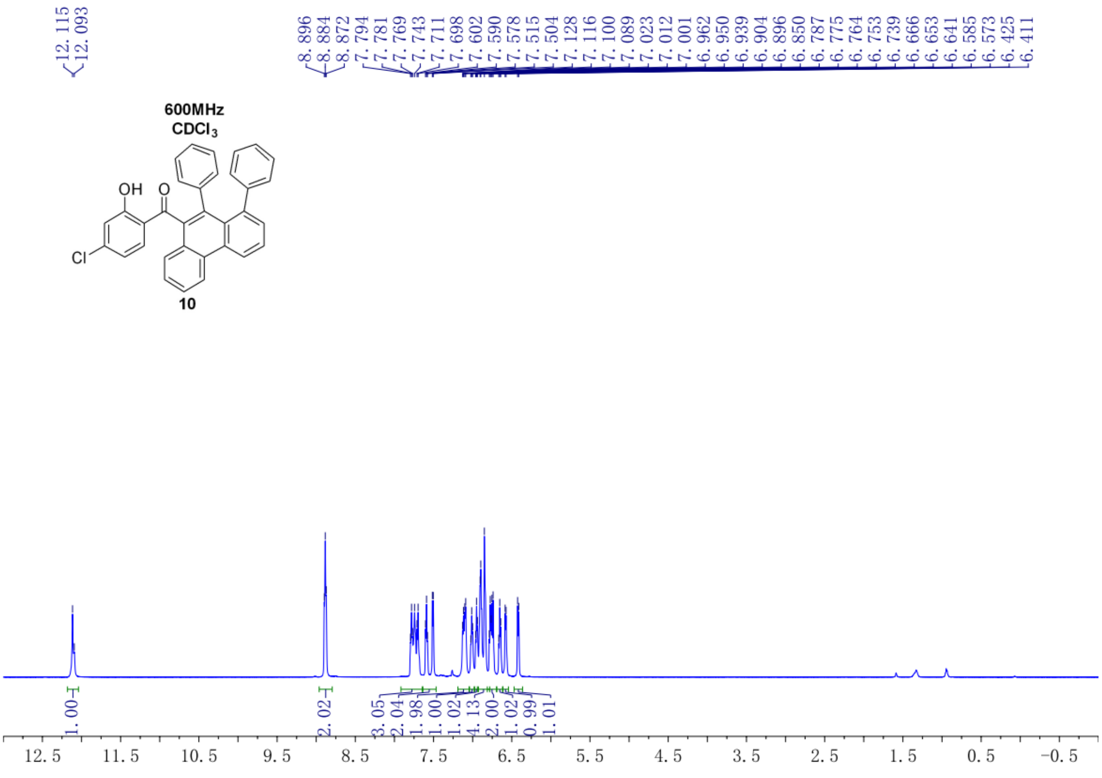
12.115
12.093
8.896
8.884
8.872
7.794
7.781
7.769
7.743
7.711
7.698
7.602
7.590
7.578
7.515
7.504
7.128
7.116
7.100
7.089
7.023
7.012
7.001
6.962
6.950
6.939
6.904
6.896
6.850
6.787
6.775
6.764
6.753
6.739
6.666
6.653
6.641
6.585
6.573
6.425
6.411
600MHz
CDCl3
OH
O
Cl
10
1.00
2.02
3.05
2.04
1.98
1.00
1.02
4.13
2.00
1.02
0.99
1.01
12.5 11.5 10.5 9.5 8.5 7.5 6.5 5.5 4.5 3.5 2.5 1.5 0.5 −0.5

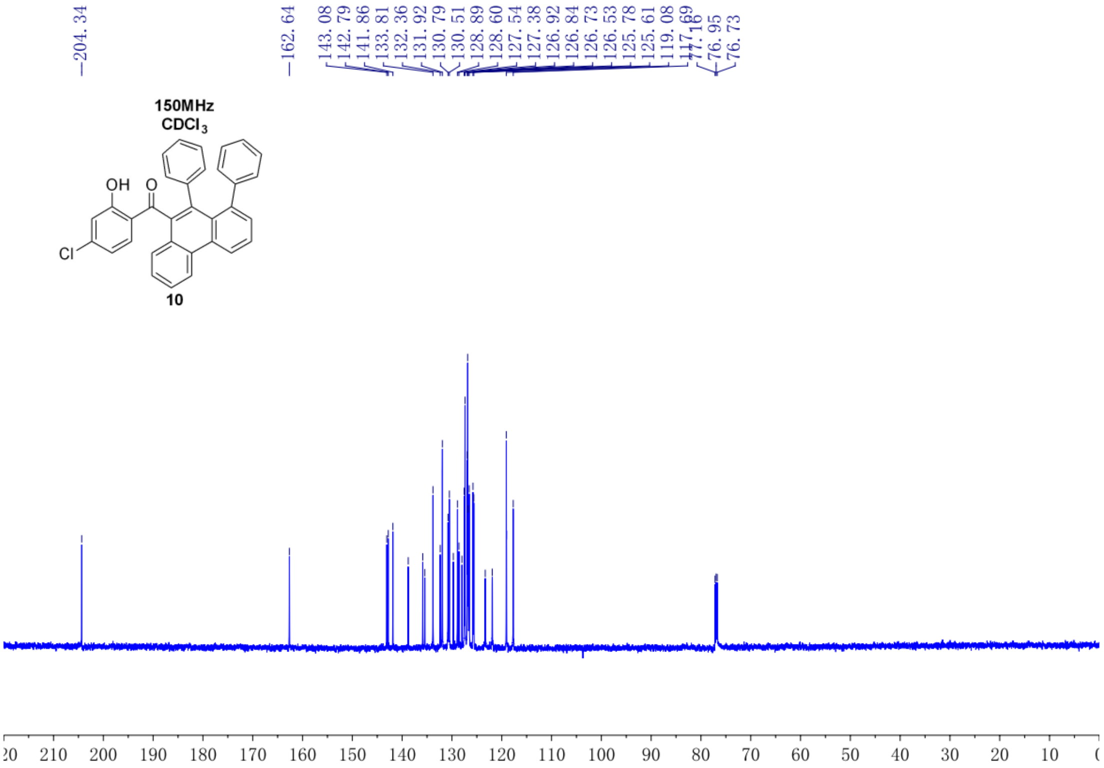
204.34
162.64
143.08
142.79
141.86
133.81
132.36
131.92
130.79
130.51
128.89
128.60
127.54
127.38
126.92
126.84
126.73
126.53
125.78
125.61
119.08
76.95
76.73
150MHz
CDCl3
OH
O
Cl
10
210 200 190 180 170 160 150 140 130 120 110 100 90 80 70 60 50 40 30 20 10

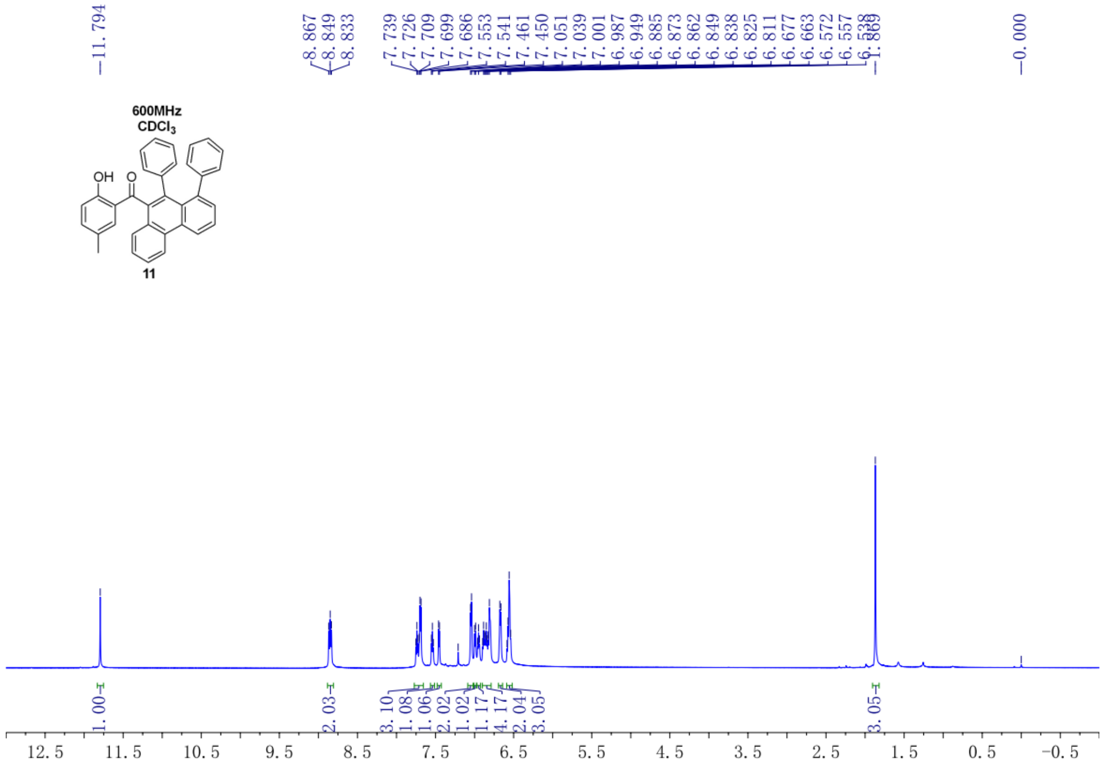
11.794
8.867
8.849
8.833
7.739
7.726
7.709
7.699
7.686
7.553
7.541
7.461
7.450
7.051
7.039
7.001
6.987
6.949
6.885
6.873
6.862
6.849
6.838
6.825
6.811
6.677
6.663
6.572
6.557
0.000
600MHz
CDCl3
OH
O
11
1.00
2.03
3.10
1.08
1.06
2.02
1.02
1.17
4.17
2.04
3.05
3.05
12.5 11.5 10.5 9.5 8.5 7.5 6.5 5.5 4.5 3.5 2.5 1.5 0.5 -0.5

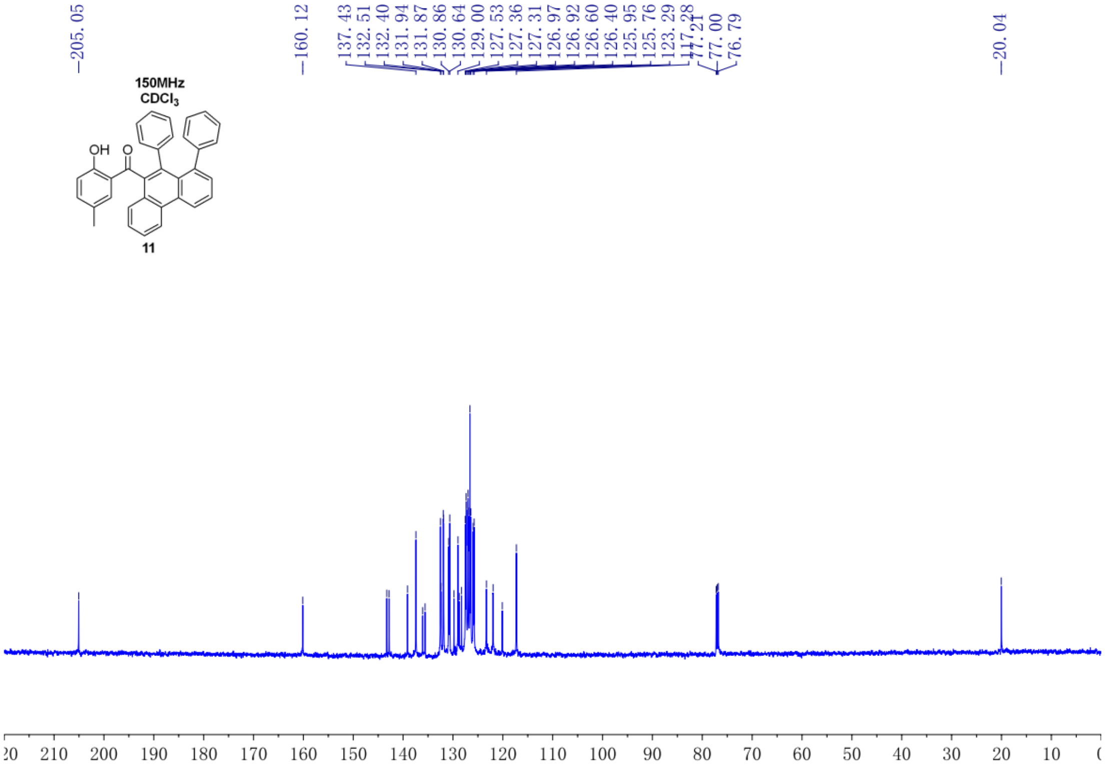
205.05
160.12
137.43
132.51
132.40
131.94
131.87
130.86
130.64
129.00
127.53
127.36
127.31
126.97
126.92
126.60
126.40
125.95
125.76
123.29
117.28
77.21
77.00
76.79
20.04
150MHz
$CDCl_3$
OH
O
11
20 210 200 190 180 170 160 150 140 130 120 110 100 90 80 70 60 50 40 30 20 10 0

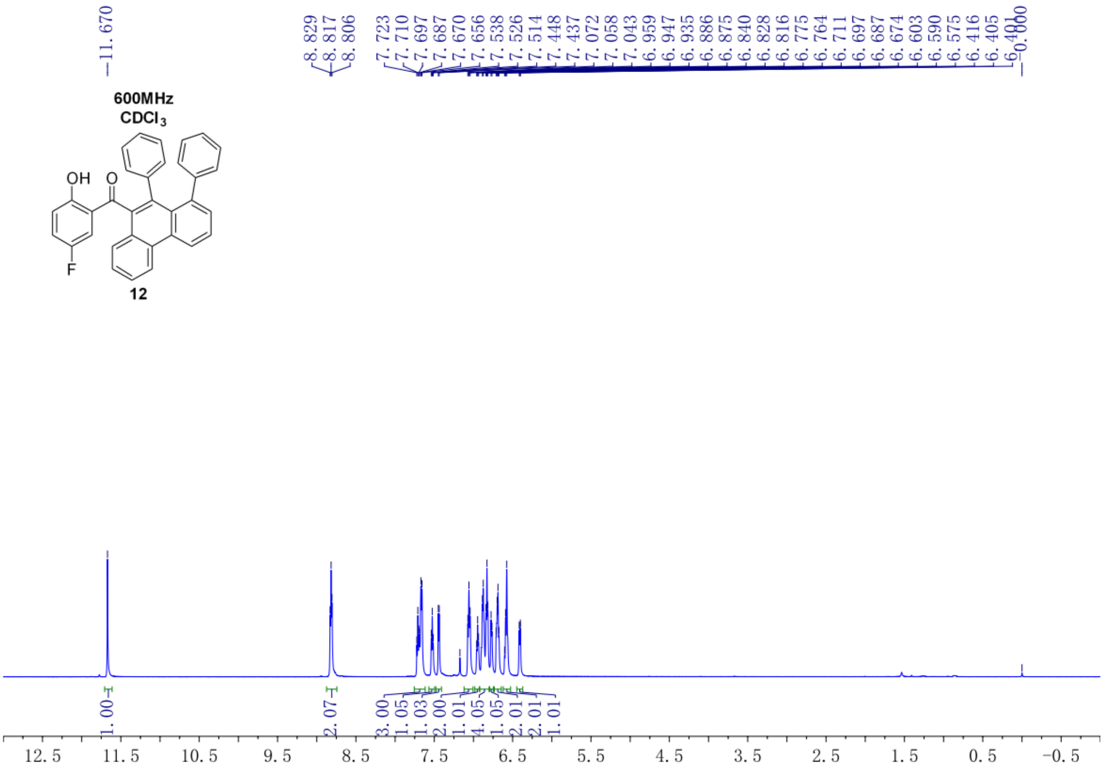
11.670
8.829
8.817
8.806
7.723
7.710
7.697
7.687
7.670
7.656
7.538
7.526
7.514
7.448
7.437
7.072
7.058
7.043
6.959
6.947
6.935
6.886
6.875
6.840
6.828
6.816
6.775
6.764
6.711
6.697
6.687
6.674
6.603
6.590
6.575
6.416
6.405
6.401
0.000
600MHz
$CDCl_3$
OH
O
F
12
1.00
2.07
3.00
1.05
1.03
2.00
1.01
4.05
1.05
2.01
2.01
1.01
12.5 11.5 10.5 9.5 8.5 7.5 6.5 5.5 4.5 3.5 2.5 1.5 0.5 -0.5

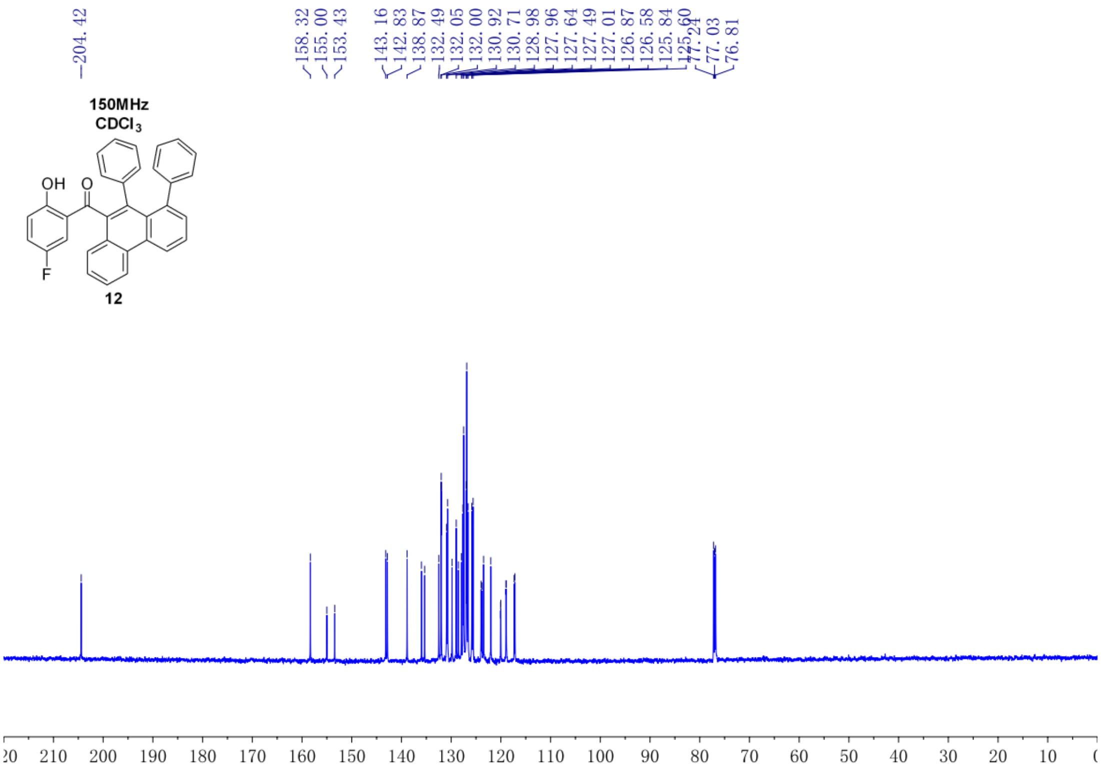

204.42
158.32
155.00
153.43
143.16
142.83
138.87
132.49
132.05
132.00
130.92
130.71
128.98
127.96
127.64
127.49
127.01
126.87
126.58
125.84
125.60
77.24
77.03
76.81
150MHz
$CDCl_3$
OH
O
F
12
210
200
190
180
170
160
150
140
130
120
110
100
90
80
70
60
50
40
30
20
10

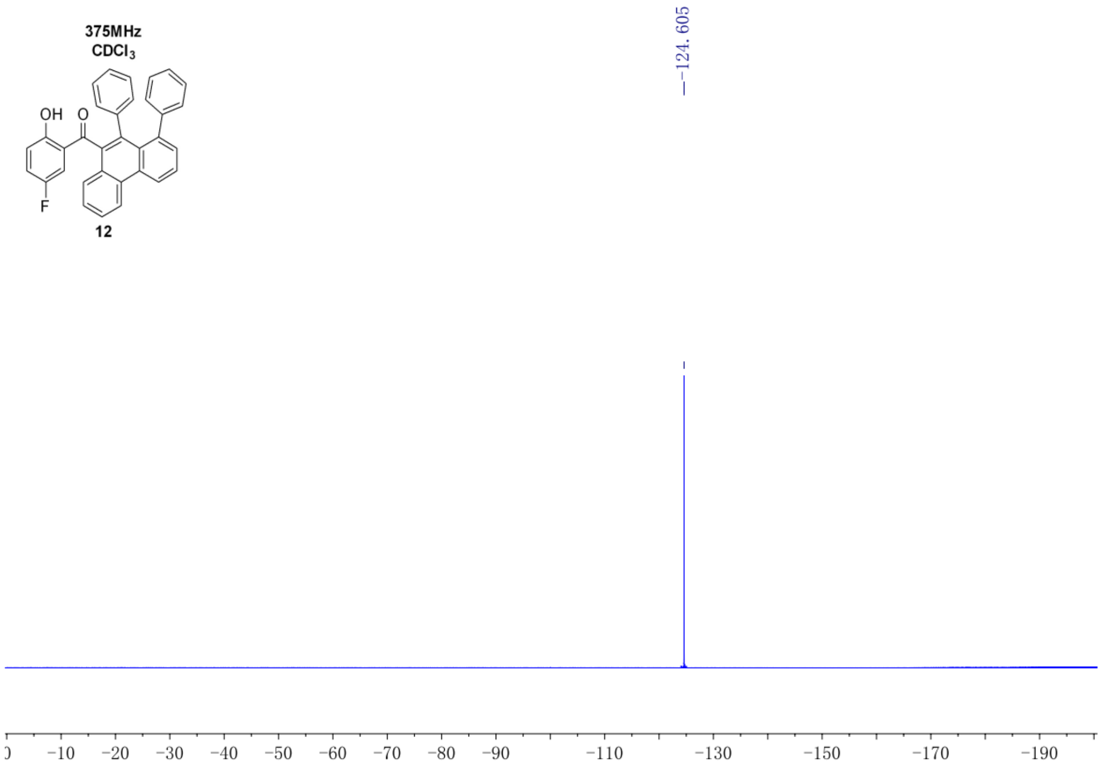

375MHz
$CDCl_3$
OH
O
F
12
−124.605
−10
−20
−30
−40
−50
−60
−70
−80
−90
−110
−130
−150
−170
−190

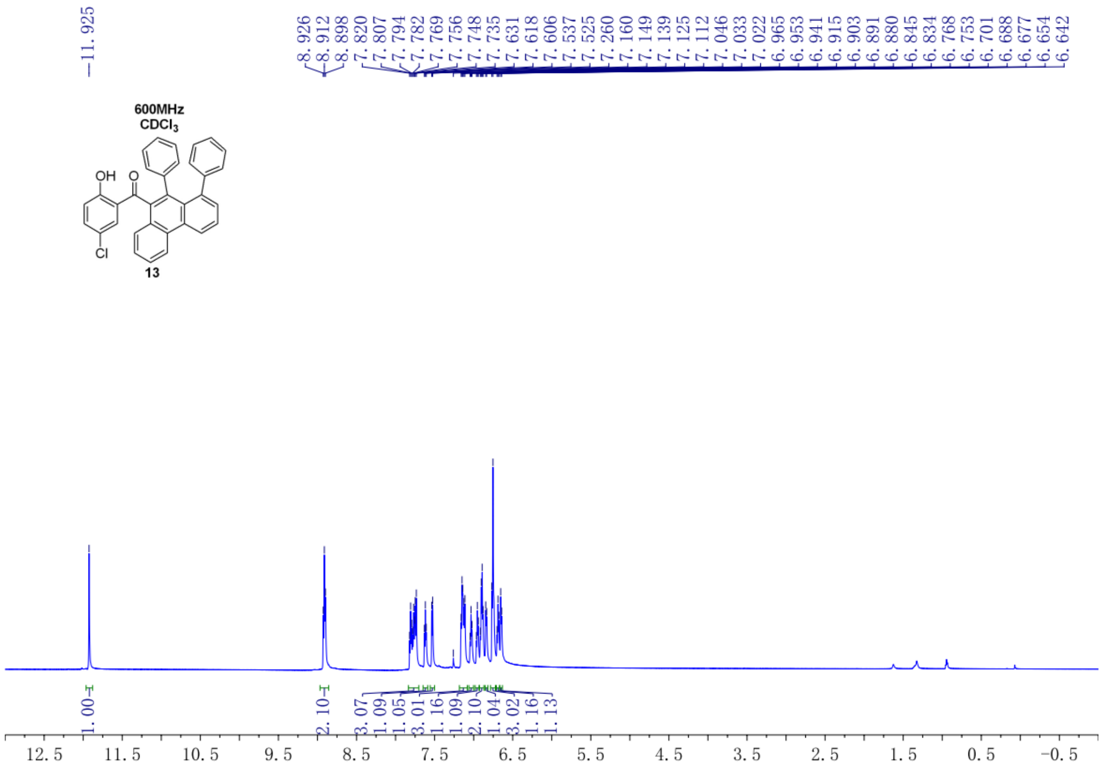
11.925
8.926
8.912
8.898
7.820
7.807
7.794
7.782
7.769
7.756
7.748
7.735
7.631
7.618
7.606
7.537
7.525
7.260
7.160
7.149
7.139
7.125
7.112
7.046
7.033
7.022
6.965
6.953
6.941
6.915
6.903
6.891
6.880
6.845
6.834
6.768
6.753
6.701
6.688
6.677
6.654
6.642
600MHz
$CDCl_3$
OH
O
Cl
13
1.00
2.10
3.07
1.09
1.05
3.01
1.16
1.09
2.10
1.04
3.02
1.16
1.13
12.5
11.5
10.5
9.5
8.5
7.5
6.5
5.5
4.5
3.5
2.5
1.5
0.5
-0.5

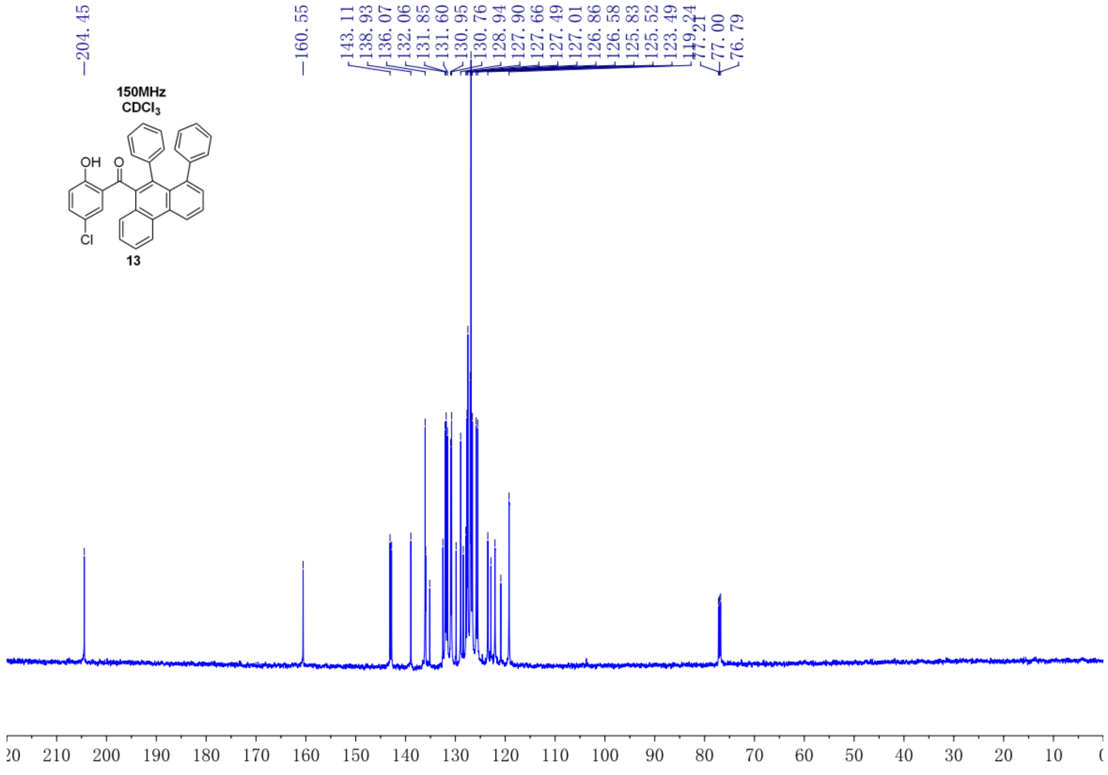
204.45
160.55
143.11
138.93
136.07
132.06
131.85
131.60
130.95
130.76
128.94
127.90
127.66
127.49
127.01
126.86
126.58
125.83
125.52
123.49
119.24
77.21
77.00
76.79
150MHz
$CDCl_3$
OH
O
Cl
13
210
200
190
180
170
160
150
140
130
120
110
100
90
80
70
60
50
40
30
20
10

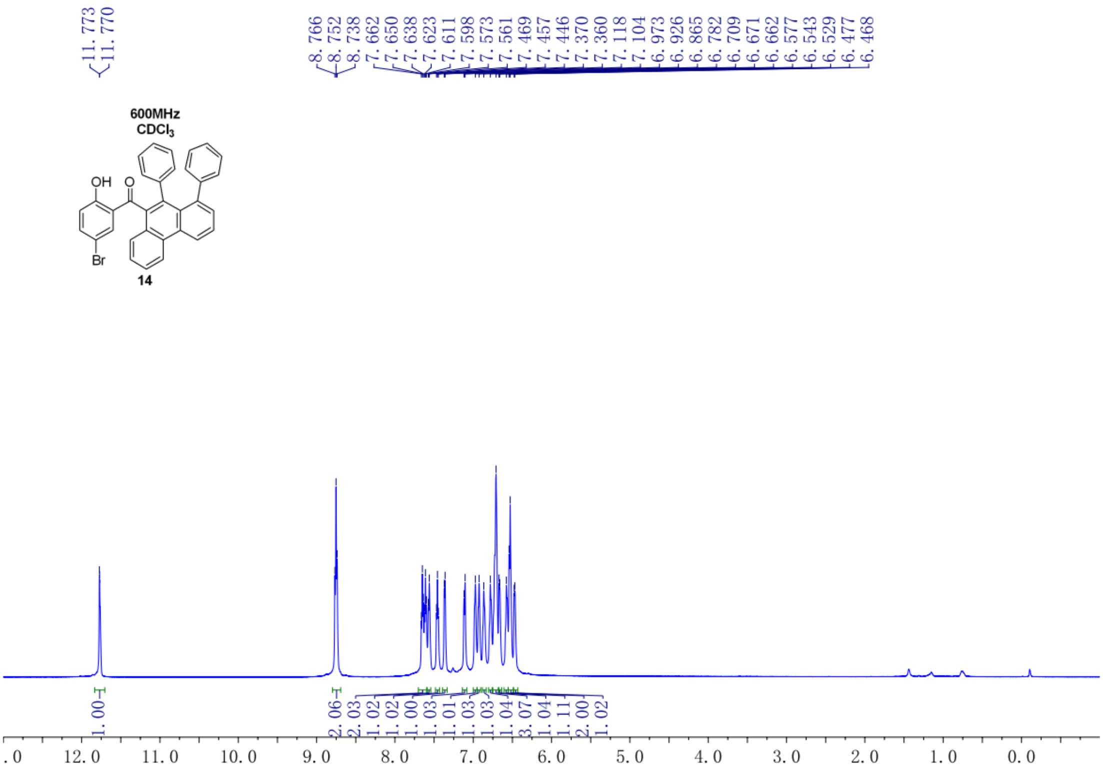
11.773
11.770
8.766
8.752
8.738
7.662
7.650
7.638
7.623
7.611
7.598
7.573
7.561
7.469
7.457
7.446
7.370
7.360
7.118
7.104
6.973
6.926
6.865
6.782
6.709
6.671
6.662
6.577
6.543
6.529
6.477
6.468
600MHz
CDCl3
OH
O
Br
14
1.00
2.06
2.03
1.02
1.02
1.00
1.03
1.01
1.03
1.03
1.04
3.07
1.04
1.11
2.00
1.02
12.0
11.0
10.0
9.0
8.0
7.0
6.0
5.0
4.0
3.0
2.0
1.0
0.0

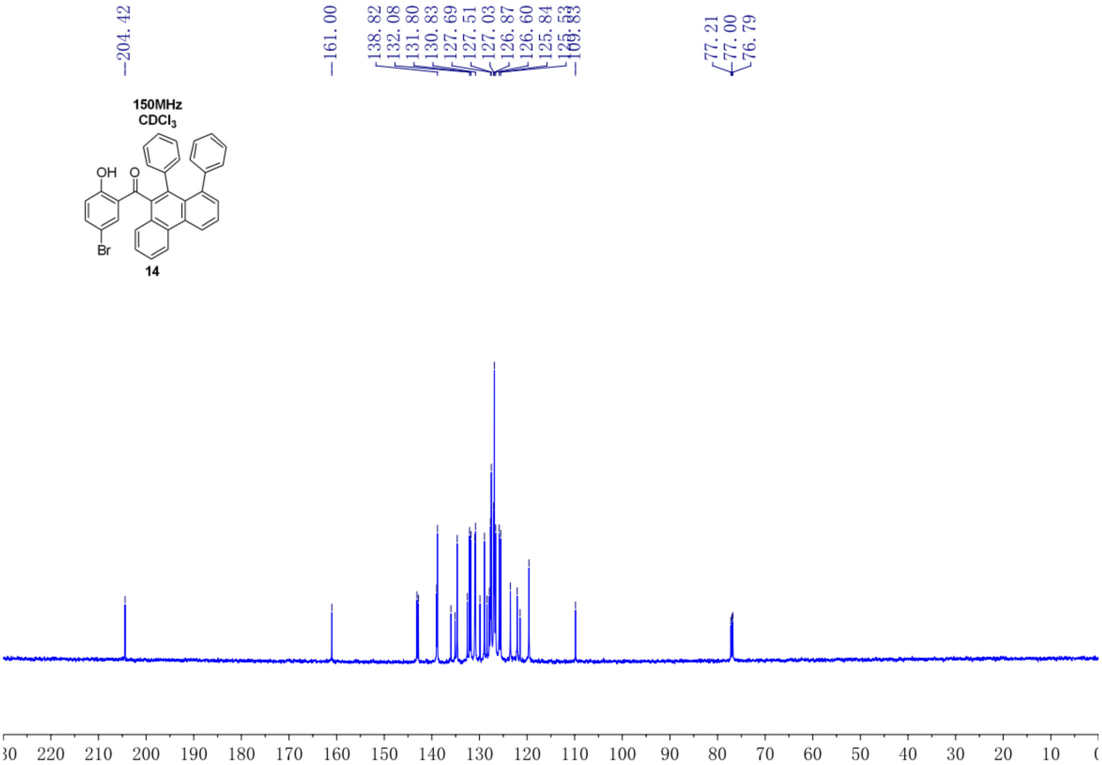
204.42
161.00
138.82
132.08
131.80
130.83
127.69
127.51
127.03
126.87
126.60
125.84
77.21
77.00
76.79
150MHz
CDCl3
OH
O
Br
14
220
210
200
190
180
170
160
150
140
130
120
110
100
90
80
70
60
50
40
30
20
10

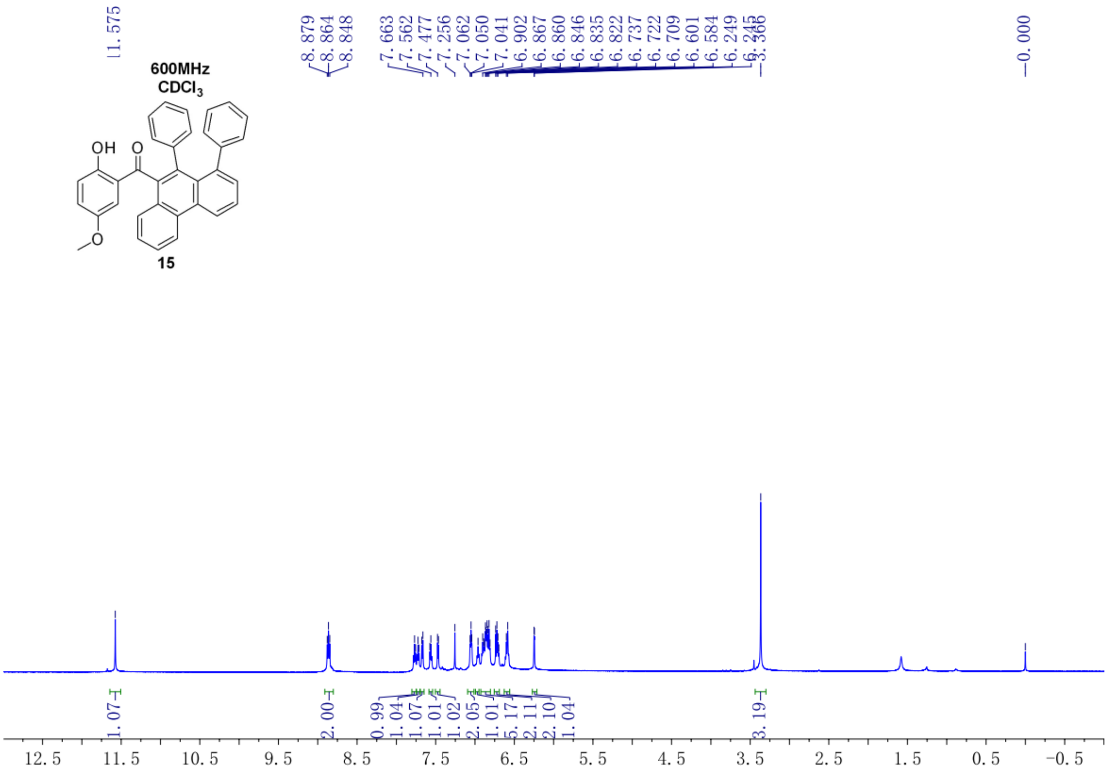
11.575
8.879
8.864
8.848
7.663
7.562
7.477
7.256
7.062
7.050
7.041
6.902
6.867
6.860
6.846
6.835
6.822
6.737
6.722
6.709
6.601
6.584
6.249
3.366
0.000
600MHz
CDCl3
OH
O
O
15
1.07
2.00
0.99
1.04
1.07
1.01
1.02
2.05
1.01
5.17
2.11
2.10
1.04
3.19
12.5 11.5 10.5 9.5 8.5 7.5 6.5 5.5 4.5 3.5 2.5 1.5 0.5 -0.5

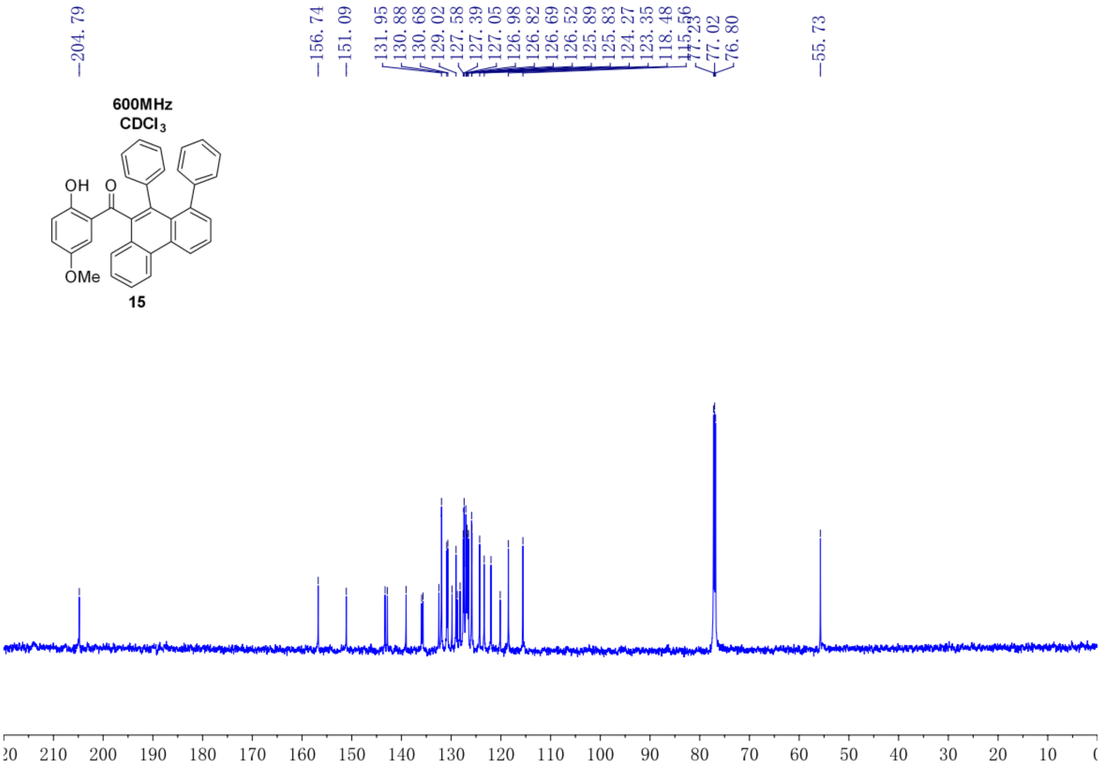
204.79
156.74
151.09
131.95
130.88
130.68
129.02
127.58
127.39
127.05
126.98
126.82
126.69
126.52
125.89
125.83
124.27
123.35
118.48
115.56
77.23
77.02
76.80
55.73
600MHz
CDCl3
OH
O
OMe
15
210 200 190 180 170 160 150 140 130 120 110 100 90 80 70 60 50 40 30 20 10

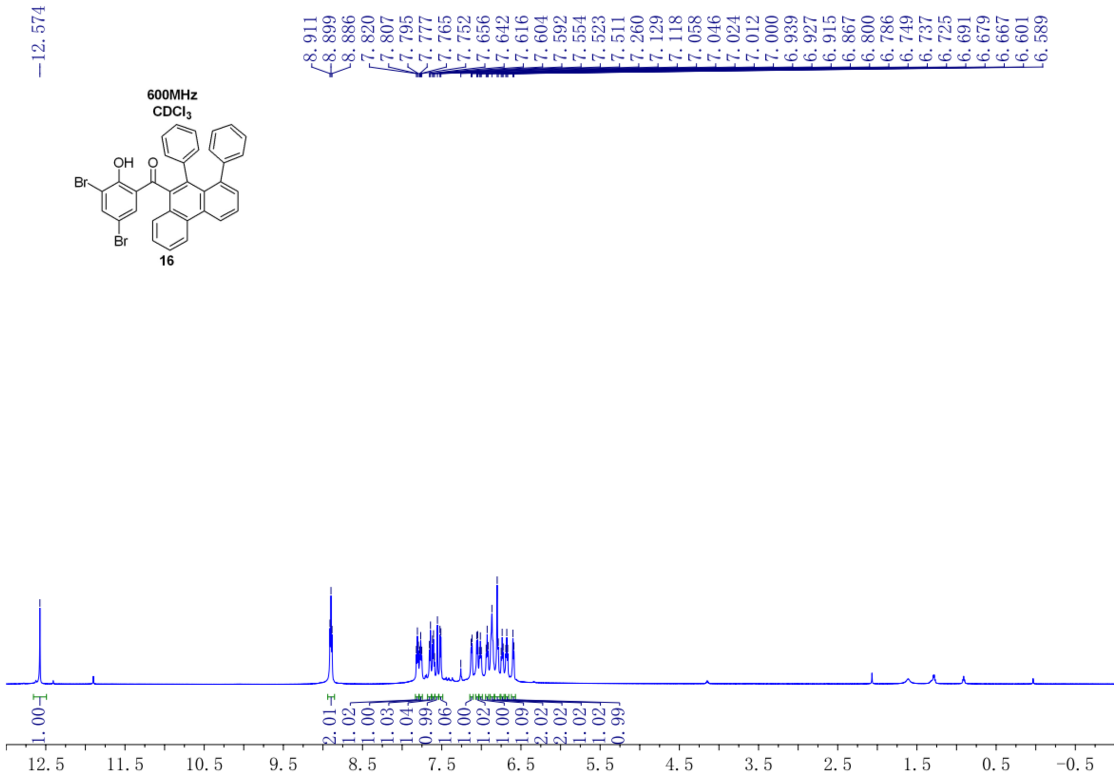

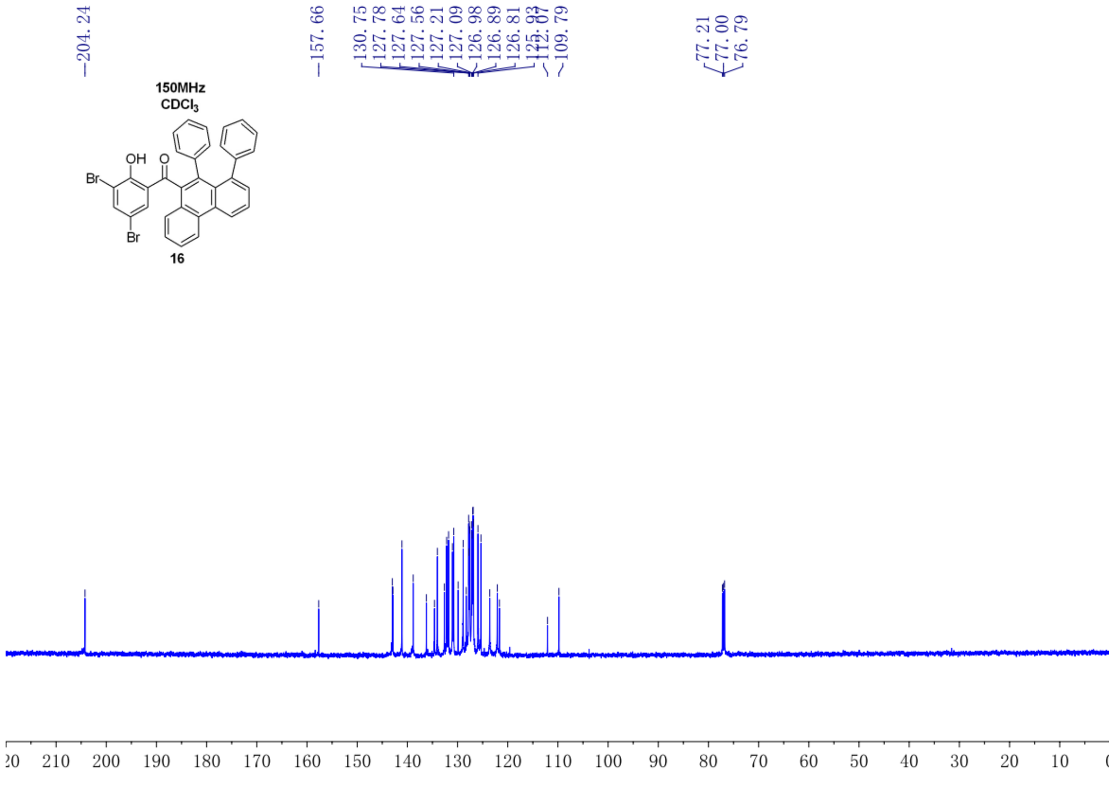

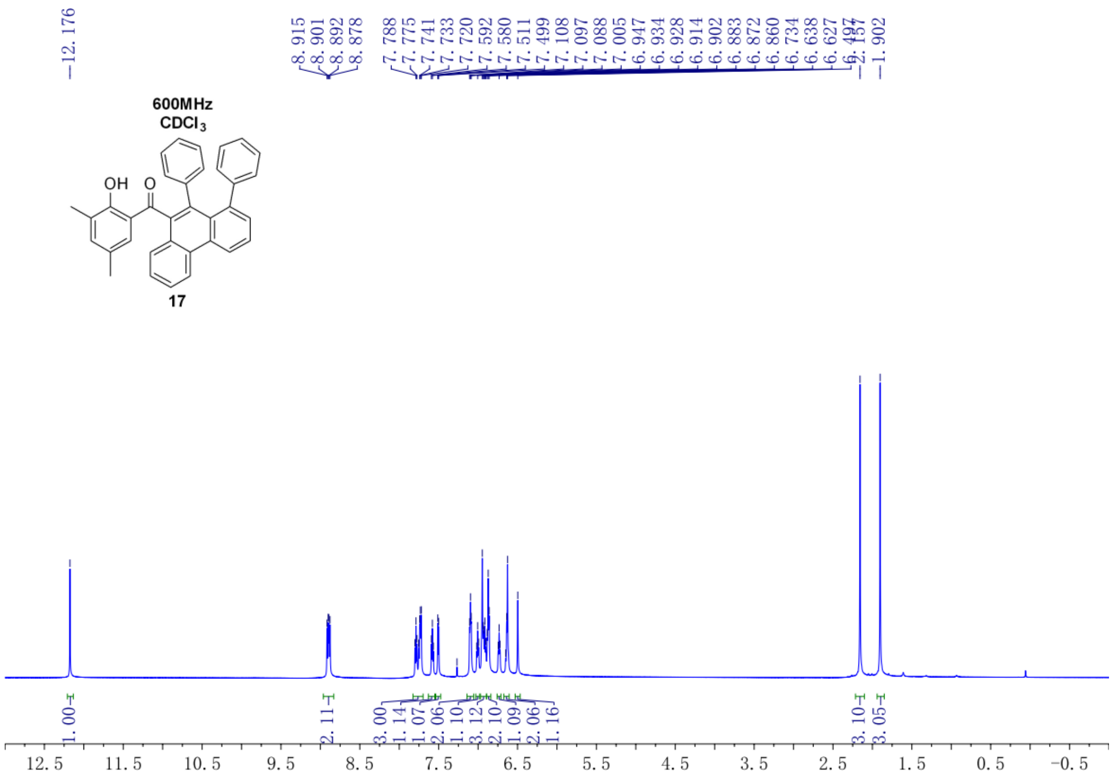
12.176
8.915
8.901
8.892
8.878
7.788
7.775
7.741
7.733
7.720
7.592
7.580
7.511
7.499
7.108
7.097
7.088
7.005
6.947
6.934
6.928
6.914
6.902
6.883
6.872
6.860
6.734
6.638
6.627
6.497
2.157
1.902
600MHz
CDCl3
OH
O
17
1.00
2.11
3.00
1.14
1.07
2.06
1.10
3.12
2.10
1.09
2.06
1.16
3.10
3.05

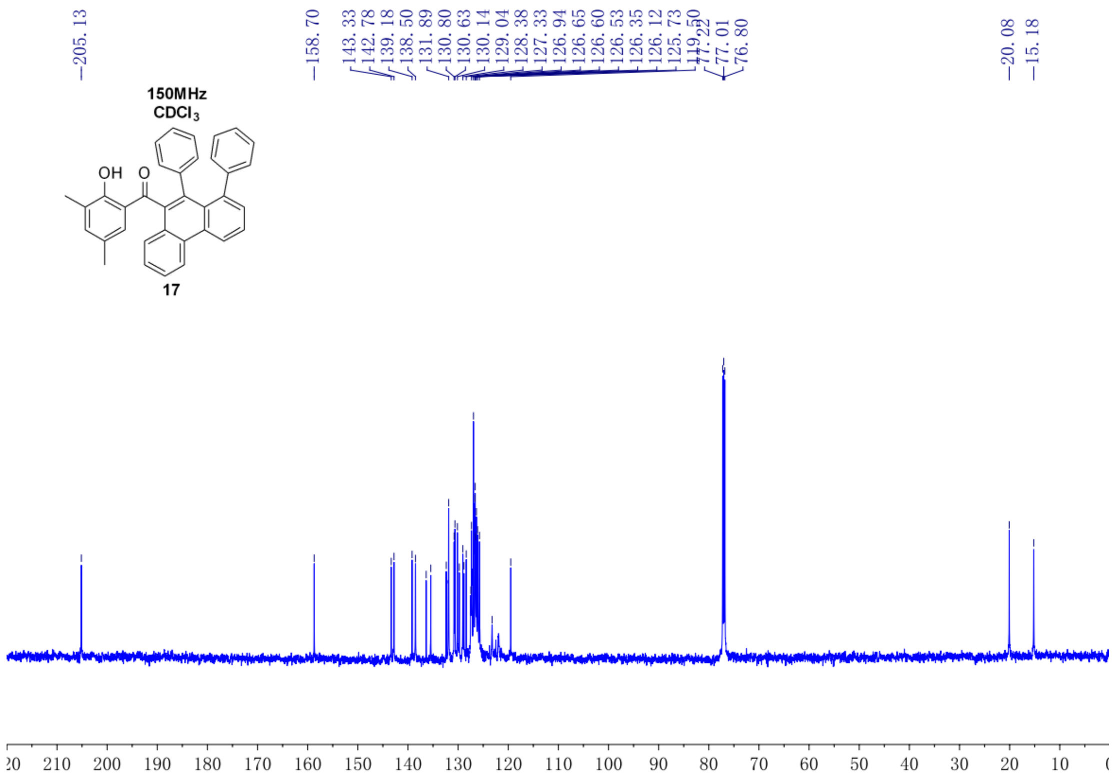
205.13
158.70
143.33
142.78
139.18
138.50
131.89
130.80
130.63
130.14
129.04
128.38
127.33
126.94
126.65
126.60
126.53
126.35
126.12
125.73
119.50
77.22
77.01
76.80
20.08
15.18
150MHz
CDCl3
OH
O
17

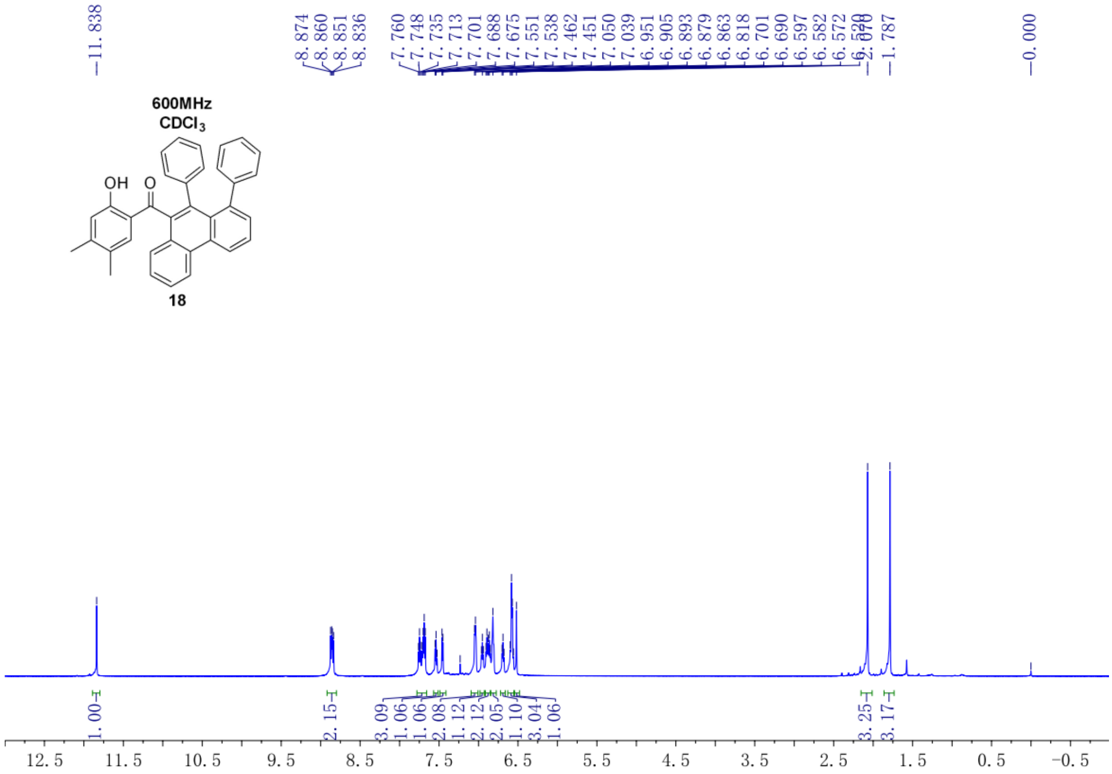
600MHz
$CDCl_3$
OH
O
18
11.838
8.874
8.860
8.851
8.836
7.760
7.748
7.735
7.713
7.701
7.688
7.675
7.551
7.538
7.462
7.451
7.050
7.039
6.951
6.905
6.893
6.879
6.863
6.818
6.701
6.690
6.597
6.582
6.572
1.787
0.000
1.00
2.15
3.09
1.06
2.08
1.12
2.12
2.05
1.10
3.04
1.06
3.25
3.17
12.5
11.5
10.5
9.5
8.5
7.5
6.5
5.5
4.5
3.5
2.5
1.5
0.5
-0.5

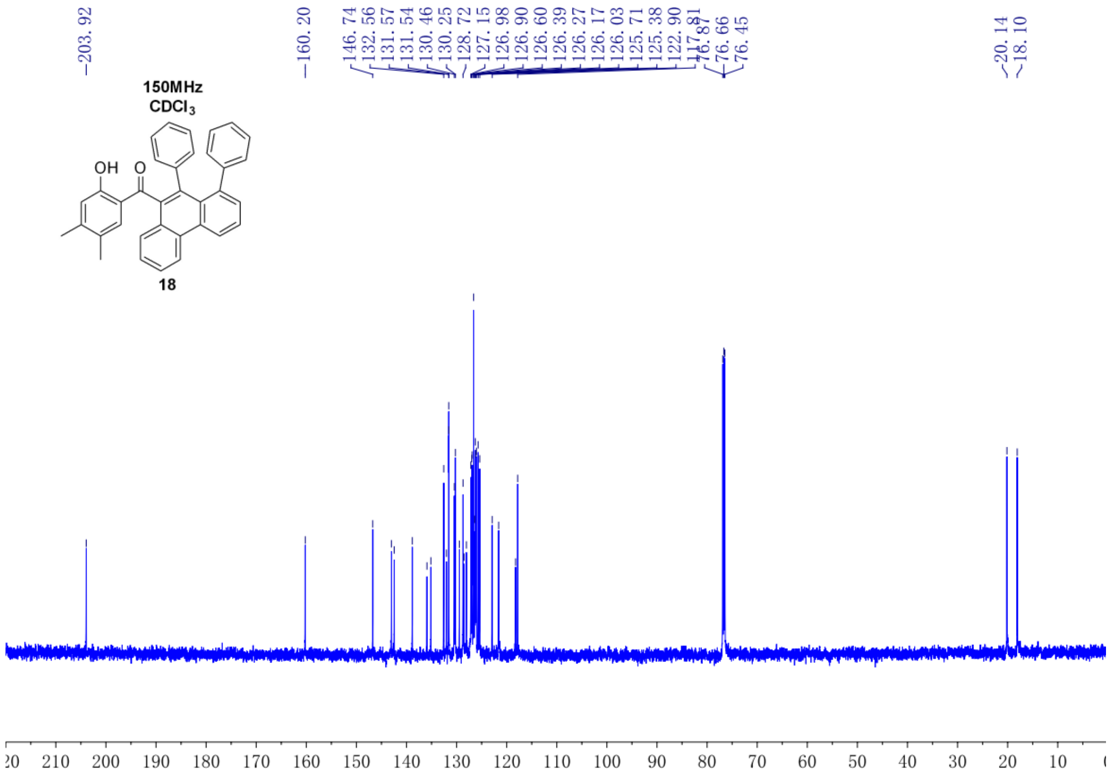
150MHz
$CDCl_3$
OH
O
18
203.92
160.20
146.74
132.56
131.57
131.54
130.46
130.25
128.72
127.15
126.98
126.90
126.60
126.39
126.27
126.17
126.03
125.71
125.38
122.90
76.87
76.66
76.45
20.14
18.10
210
200
190
180
170
160
150
140
130
120
110
100
90
80
70
60
50
40
30
20
10

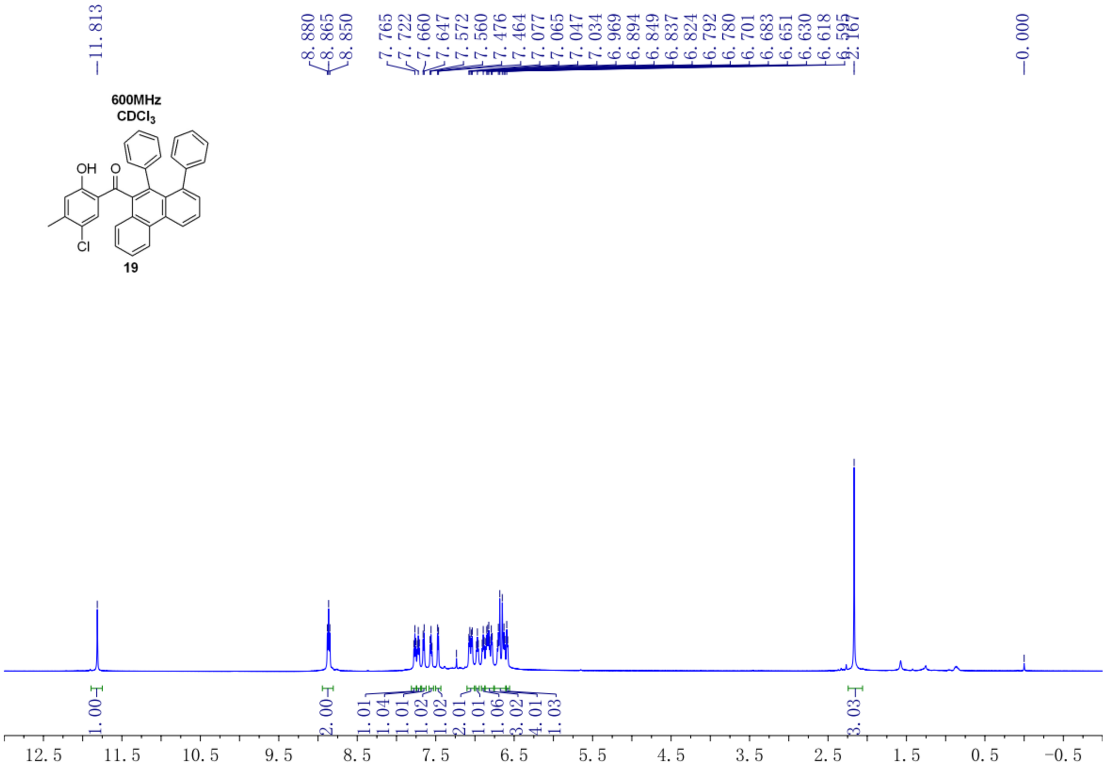
600MHz
CDCl3
OH
O
Cl
19
11.813
8.880
8.865
8.850
7.765
7.722
7.660
7.647
7.572
7.560
7.476
7.464
7.077
7.065
7.047
7.034
6.969
6.894
6.849
6.837
6.824
6.792
6.780
6.701
6.683
6.651
6.630
6.618
6.595
2.167
0.000
1.00
2.00
1.01
1.04
1.01
1.02
1.02
2.01
1.01
1.06
3.02
4.01
1.03
3.03
12.5
11.5
10.5
9.5
8.5
7.5
6.5
5.5
4.5
3.5
2.5
1.5
0.5
-0.5

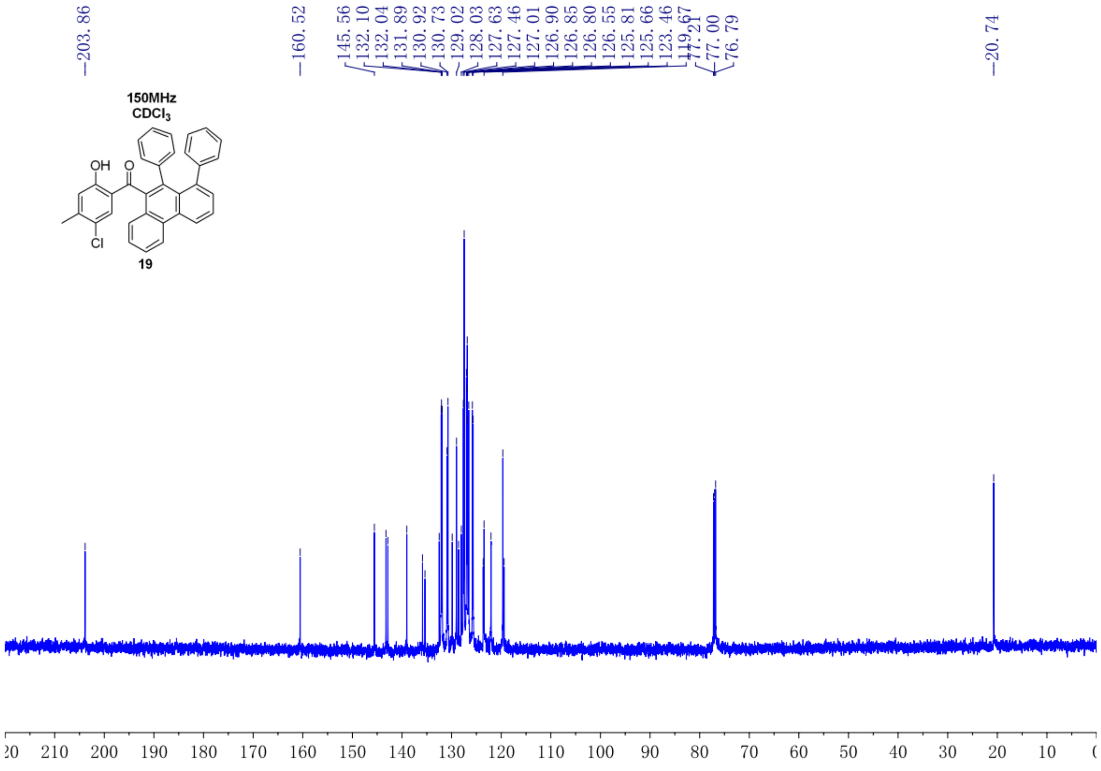
150MHz
CDCl3
OH
O
Cl
19
203.86
160.52
145.56
132.10
132.04
131.89
130.92
130.73
129.02
128.03
127.63
127.46
127.01
126.90
126.85
126.80
126.55
125.81
125.66
123.46
119.67
77.21
77.00
76.79
20.74
210
200
190
180
170
160
150
140
130
120
110
100
90
80
70
60
50
40
30
20
10

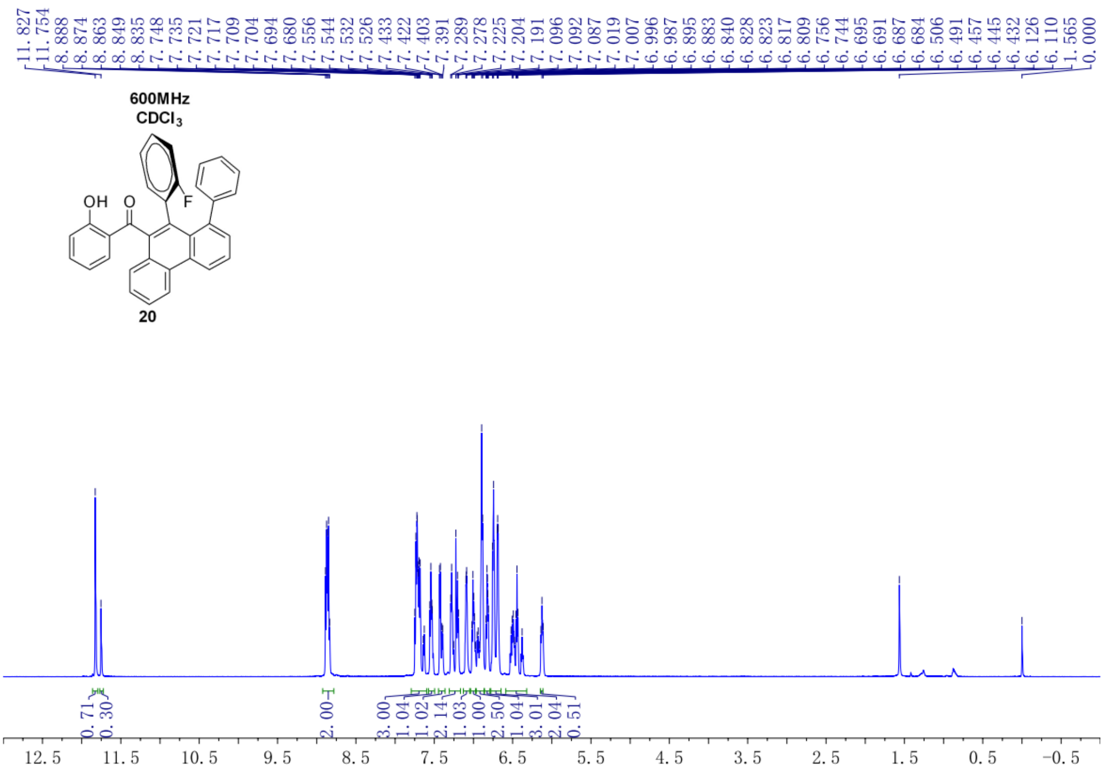
11.827
11.754
8.888
8.874
8.863
8.849
8.835
7.748
7.735
7.721
7.717
7.709
7.704
7.694
7.680
7.556
7.544
7.532
7.526
7.433
7.422
7.403
7.391
7.289
7.278
7.225
7.204
7.191
7.096
7.092
7.087
7.019
7.007
6.996
6.987
6.895
6.883
6.840
6.828
6.823
6.817
6.809
6.756
6.744
6.695
6.691
6.687
6.684
6.506
6.491
6.457
6.445
6.432
6.126
6.110
1.565
0.000
600MHz
CDCl3
OH
O
F
20
0.71
0.30
2.00
3.00
1.04
1.02
2.14
1.03
1.00
2.50
1.04
3.01
2.04
0.51
12.5 11.5 10.5 9.5 8.5 7.5 6.5 5.5 4.5 3.5 2.5 1.5 0.5 -0.5

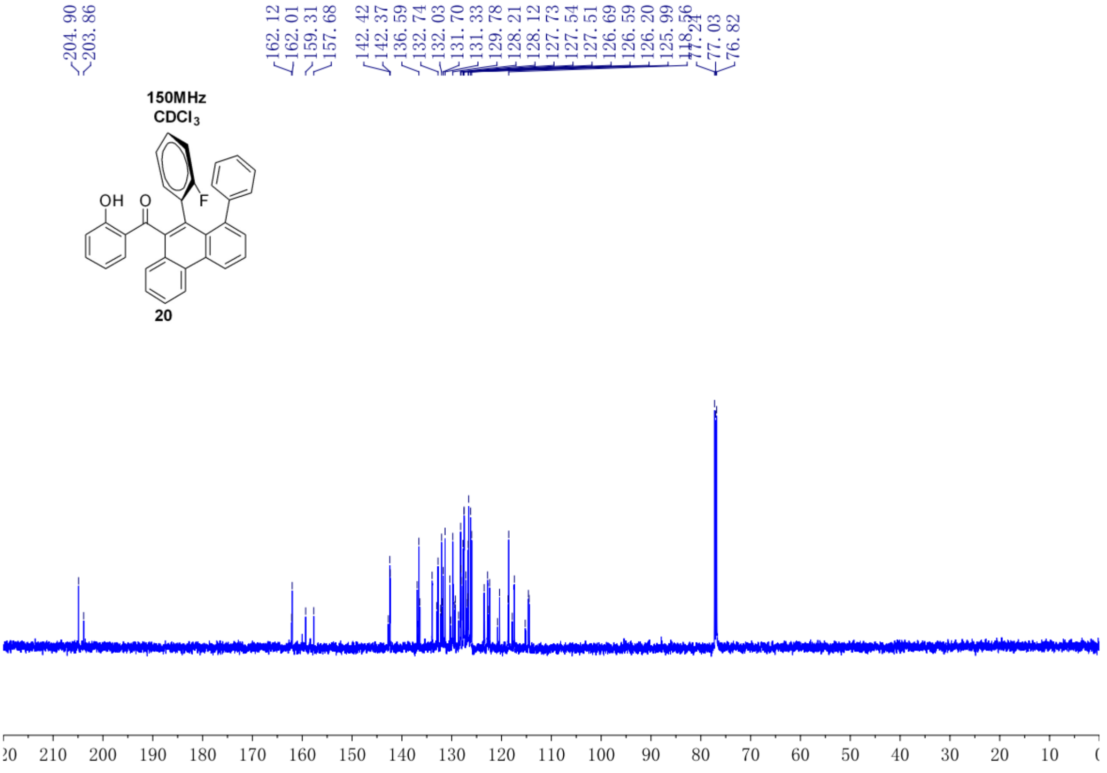
204.90
203.86
162.12
162.01
159.31
157.68
142.42
142.37
136.59
132.74
132.03
131.70
131.33
129.78
128.21
128.12
127.73
127.54
127.51
126.69
126.59
126.20
125.99
118.56
77.24
77.03
76.82
150MHz
CDCl3
OH
O
F
20
210 200 190 180 170 160 150 140 130 120 110 100 90 80 70 60 50 40 30 20 10

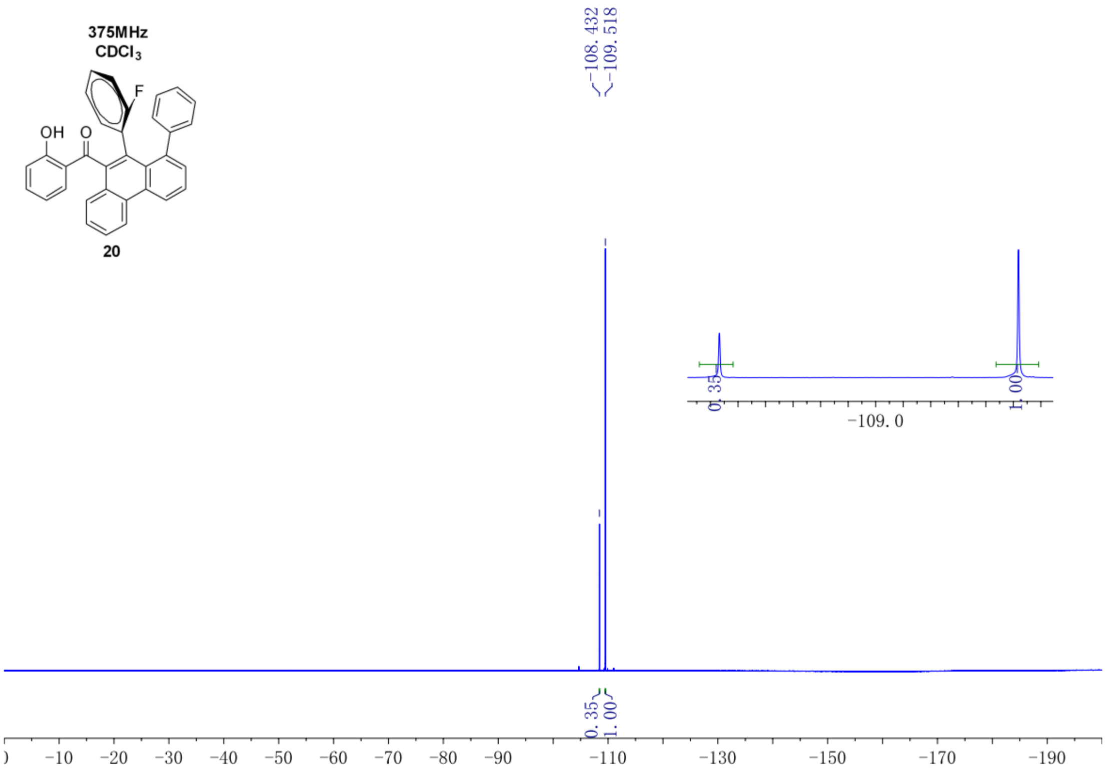
375MHz
CDCl3
F
OH
O
20
-108.432
-109.518
0.35
1.00
-109.0
-10 -20 -30 -40 -50 -60 -70 -80 -90 -110 -130 -150 -170 -190

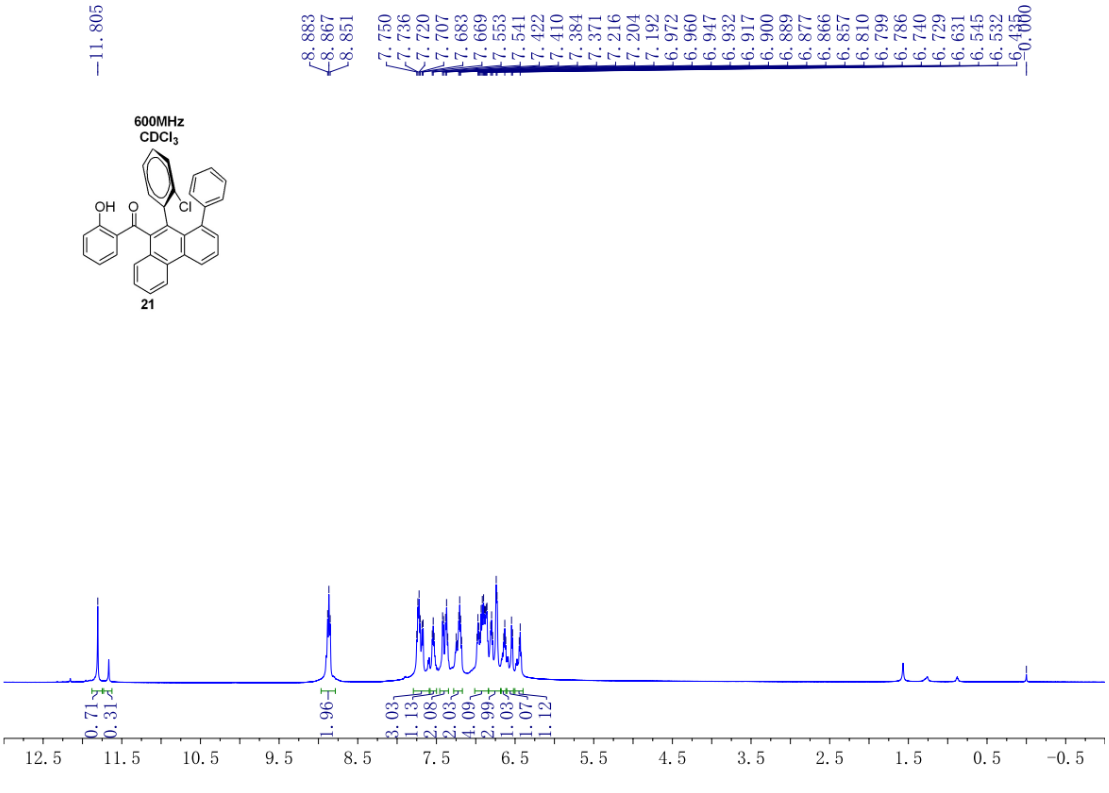
600MHz
CDCl3
Cl
OH
O
21
11.805
8.883
8.867
8.851
7.750
7.736
7.720
7.707
7.683
7.669
7.553
7.541
7.422
7.410
7.384
7.371
7.216
7.204
7.192
6.972
6.960
6.947
6.932
6.917
6.900
6.889
6.877
6.866
6.857
6.810
6.799
6.786
6.740
6.729
6.631
6.545
6.532
6.435
0.000
0.71
0.31
1.96
3.03
1.13
2.08
2.03
4.09
2.99
1.03
1.07
1.12
12.5 11.5 10.5 9.5 8.5 7.5 6.5 5.5 4.5 3.5 2.5 1.5 0.5 -0.5

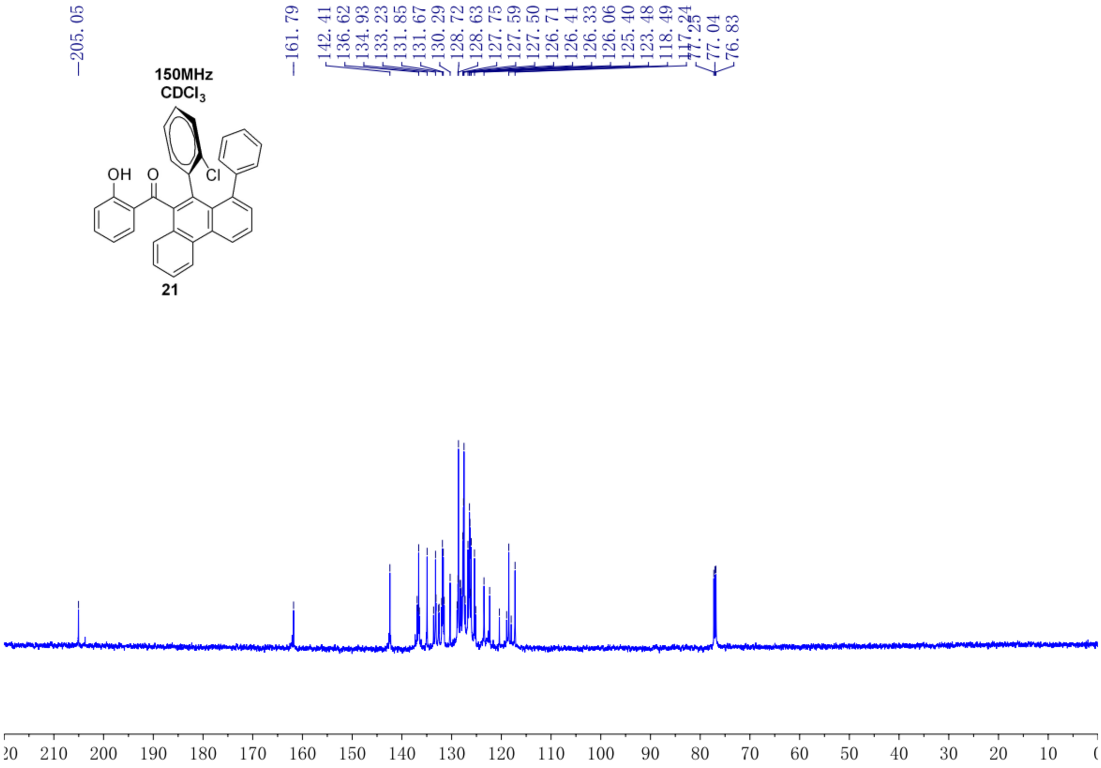
205.05
161.79
142.41
136.62
134.93
133.23
131.85
131.67
130.29
128.72
128.63
127.75
127.59
127.50
126.71
126.41
126.33
126.06
125.40
123.48
118.49
117.24
77.25
77.04
76.83
150MHz
$CDCl_3$
OH
O
Cl
21
210 200 190 180 170 160 150 140 130 120 110 100 90 80 70 60 50 40 30 20 10

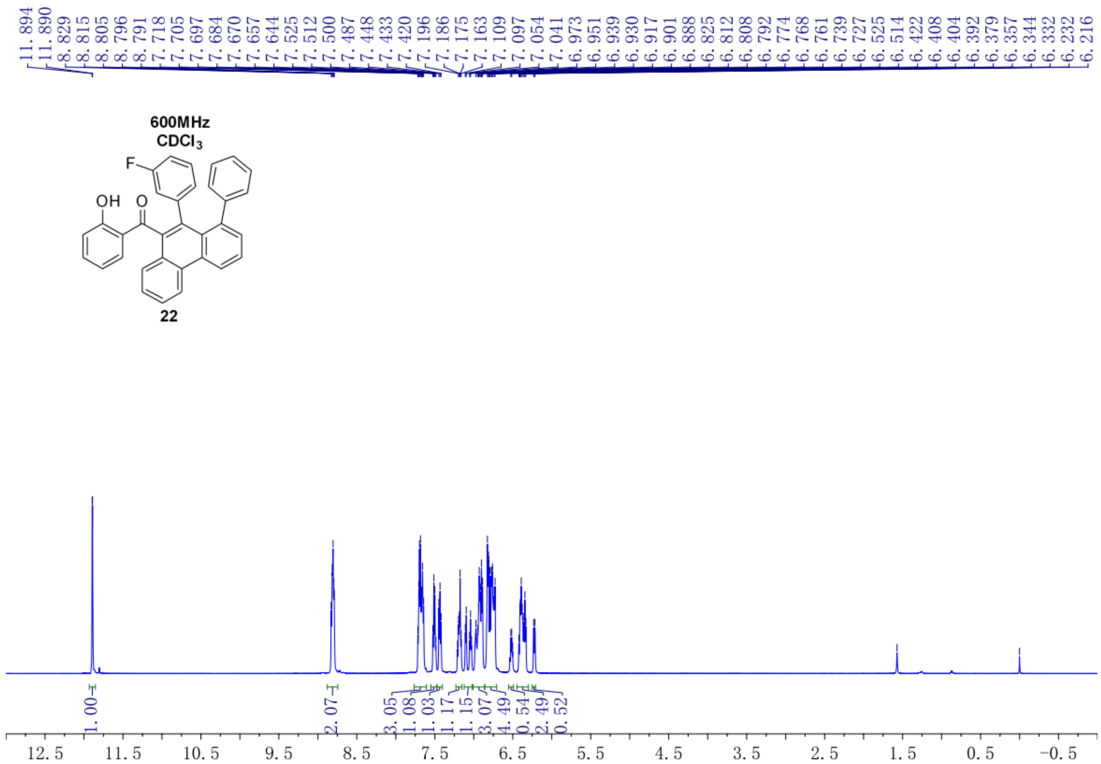
11.894
11.890
8.829
8.815
8.805
8.796
8.791
7.718
7.705
7.697
7.684
7.670
7.657
7.644
7.525
7.512
7.500
7.487
7.448
7.433
7.420
7.196
7.186
7.175
7.163
7.109
7.097
7.054
7.041
6.973
6.951
6.939
6.930
6.917
6.901
6.888
6.825
6.812
6.808
6.792
6.774
6.768
6.761
6.739
6.727
6.525
6.514
6.422
6.408
6.404
6.392
6.379
6.357
6.344
6.332
6.232
6.216
600MHz
$CDCl_3$
F
OH
O
22
1.00
2.07
3.05
1.08
1.03
1.17
1.15
3.07
4.49
0.54
2.49
0.52
12.5 11.5 10.5 9.5 8.5 7.5 6.5 5.5 4.5 3.5 2.5 1.5 0.5 -0.5

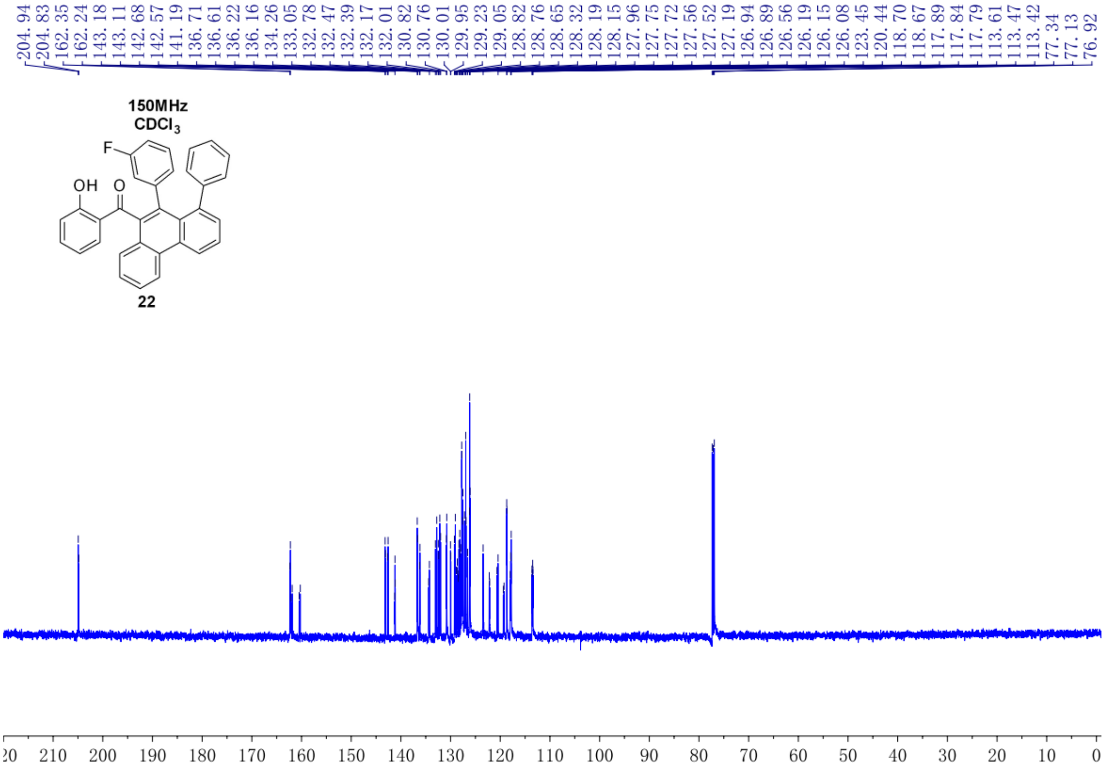
204.94
204.83
162.35
162.24
143.18
143.11
142.68
142.57
141.19
136.71
136.61
136.22
136.16
134.26
133.05
132.78
132.47
132.39
132.17
132.01
130.82
130.76
130.01
129.95
129.23
129.05
128.82
128.76
128.65
128.32
128.19
128.15
127.96
127.75
127.72
127.56
127.52
127.19
126.94
126.89
126.56
126.19
126.15
126.08
123.45
120.44
118.70
118.67
117.89
117.84
117.79
113.61
113.47
113.42
77.34
77.13
76.92
150MHz
CDCl3
F
OH
O
22
20 210 200 190 180 170 160 150 140 130 120 110 100 90 80 70 60 50 40 30 20 10 0

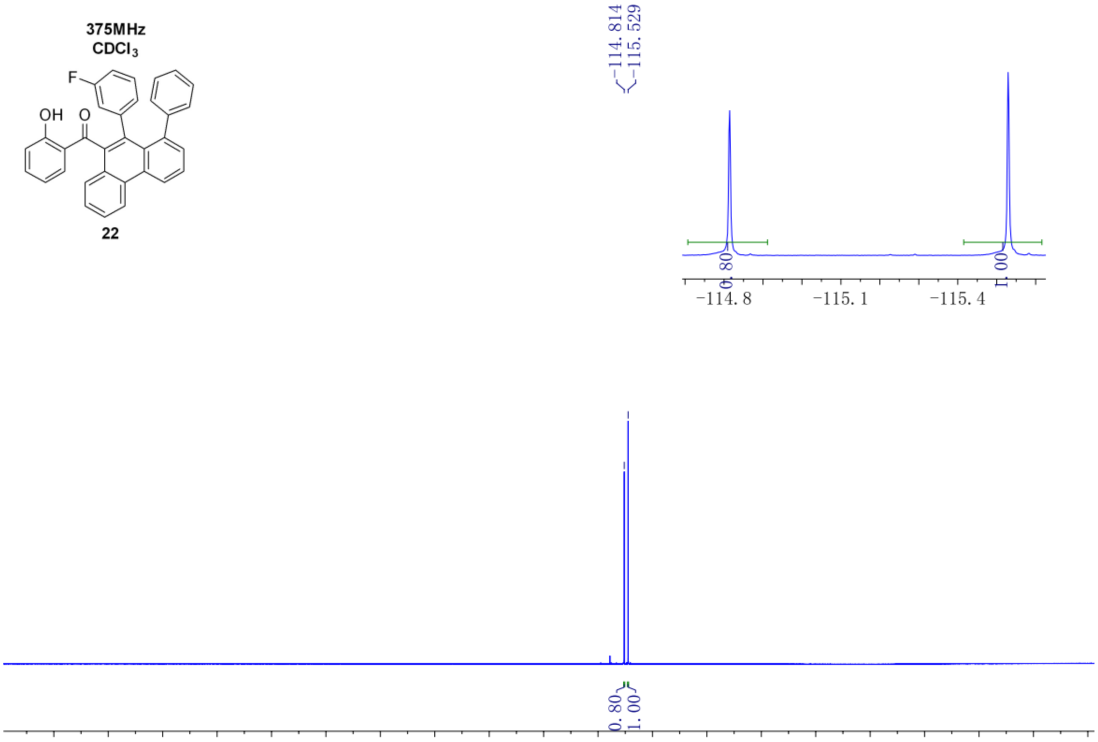
375MHz
CDCl3
F
OH
O
22
-114.814
-115.529
0.80
1.00
-114.8
-115.1
-115.4
-10 -20 -30 -40 -50 -60 -70 -80 -90 -110 -130 -150 -170 -190

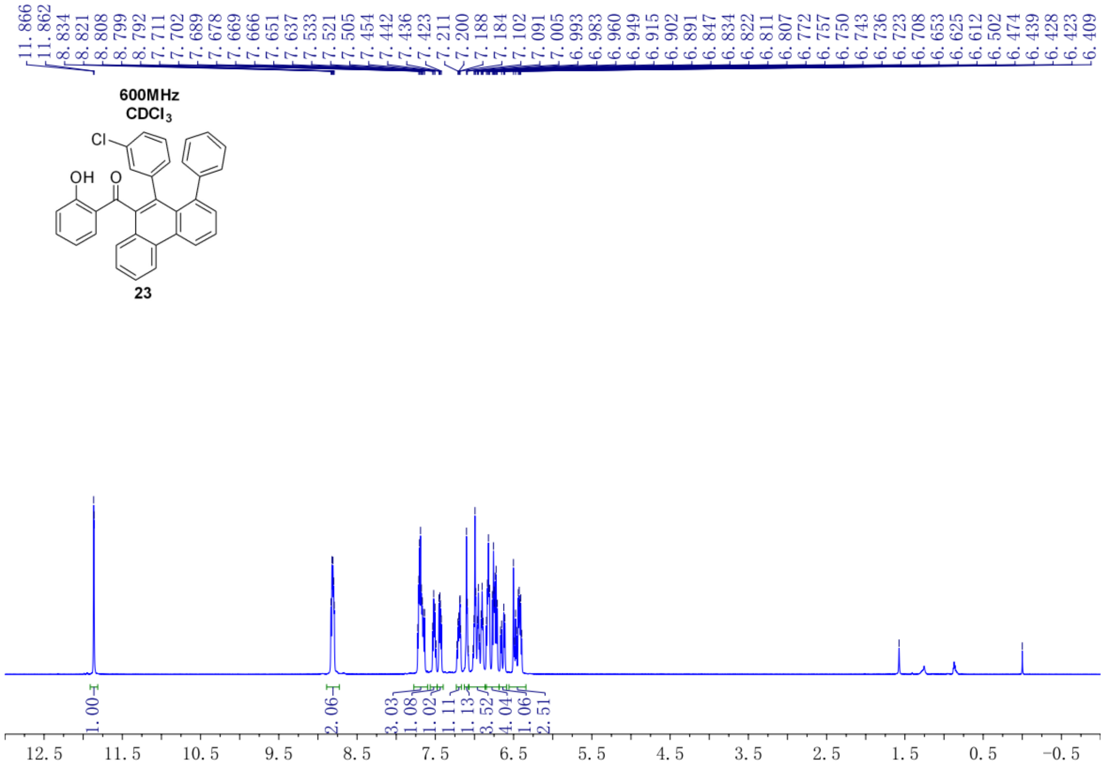
11.866
11.862
8.834
8.821
8.808
8.799
8.792
7.711
7.702
7.689
7.678
7.669
7.666
7.651
7.637
7.533
7.521
7.505
7.454
7.442
7.436
7.423
7.211
7.200
7.188
7.184
7.102
7.091
7.005
6.993
6.983
6.960
6.949
6.915
6.902
6.891
6.847
6.834
6.822
6.811
6.807
6.772
6.757
6.750
6.743
6.736
6.723
6.708
6.653
6.625
6.612
6.502
6.474
6.439
6.428
6.423
6.409
600MHz
CDCl3
Cl
OH
O
23
1.00
2.06
3.03
1.08
1.02
1.11
1.13
3.52
4.04
1.06
2.51
12.5
11.5
10.5
9.5
8.5
7.5
6.5
5.5
4.5
3.5
2.5
1.5
0.5
-0.5

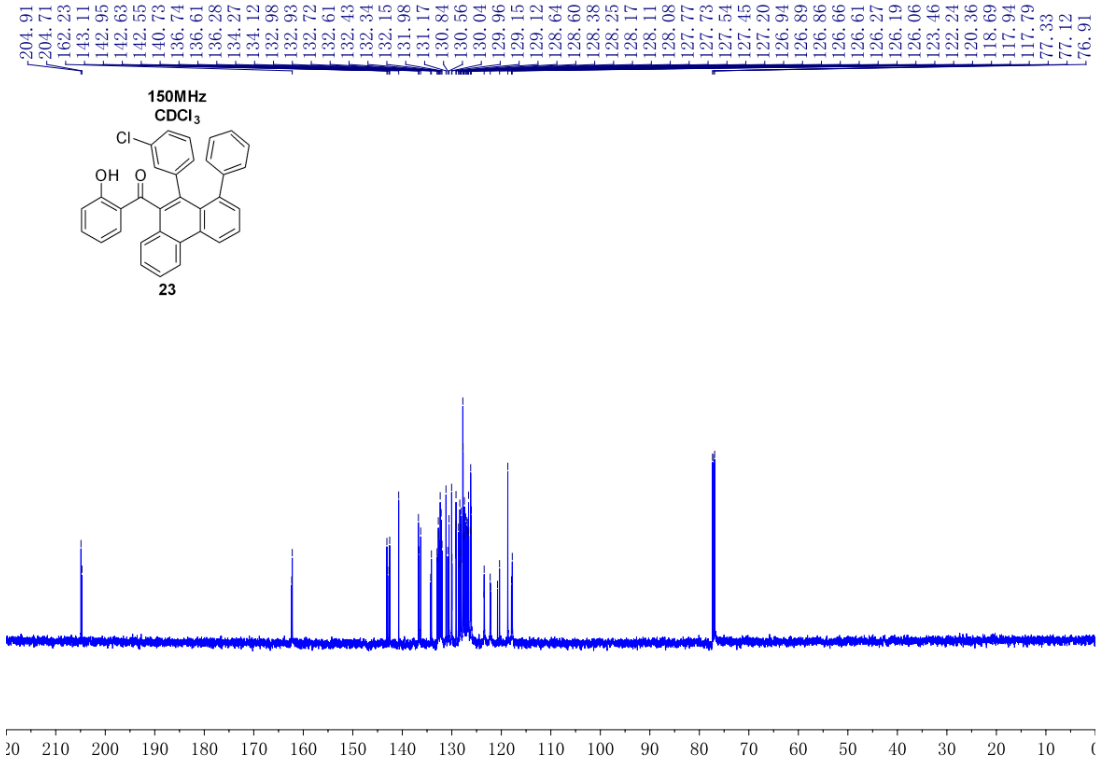
204.91
204.71
162.23
143.11
142.95
142.63
142.55
140.73
136.74
136.61
136.28
134.27
134.12
132.98
132.93
132.72
132.61
132.43
132.34
132.15
131.98
131.17
130.84
130.56
130.04
129.96
129.15
129.12
128.64
128.60
128.38
128.25
128.17
128.11
128.08
127.77
127.73
127.54
127.45
127.20
126.94
126.89
126.86
126.66
126.61
126.27
126.19
126.06
123.46
122.24
120.36
118.69
117.94
117.79
77.33
77.12
76.91
150MHz
CDCl3
Cl
OH
O
23
210
200
190
180
170
160
150
140
130
120
110
100
90
80
70
60
50
40
30
20
10

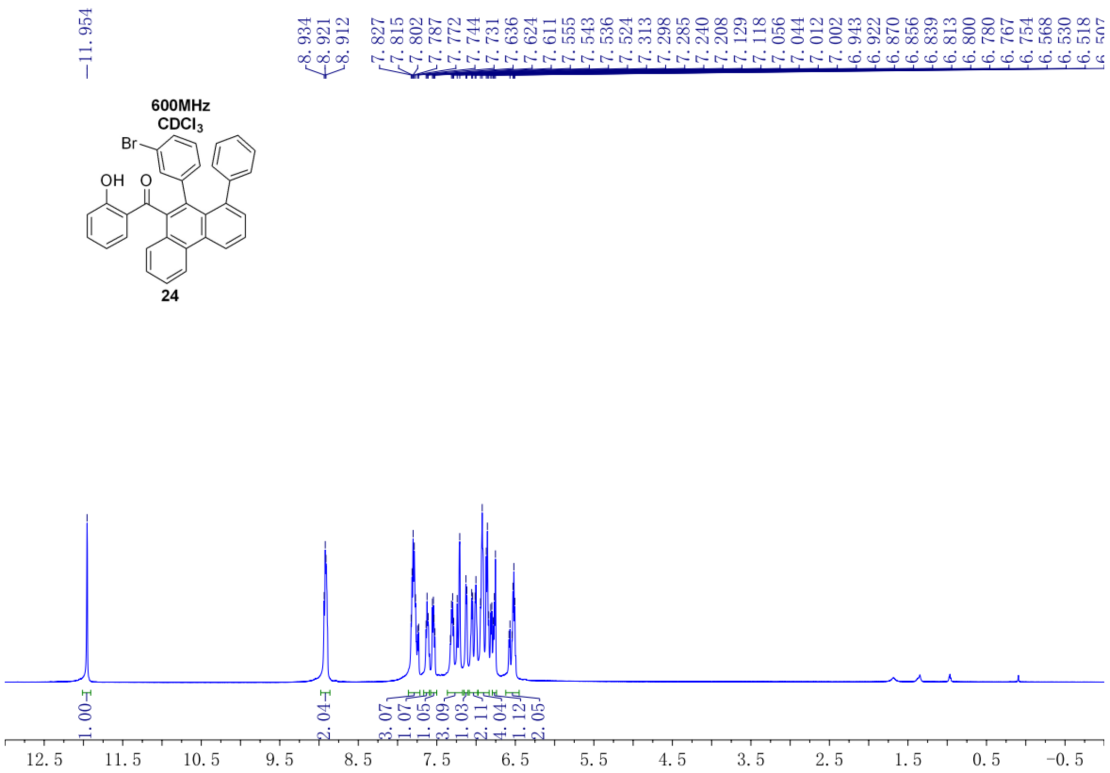
11.954
8.934
8.921
8.912
7.827
7.815
7.802
7.787
7.772
7.744
7.731
7.636
7.624
7.611
7.555
7.543
7.536
7.524
7.313
7.298
7.285
7.240
7.208
7.129
7.118
7.056
7.044
7.012
7.002
6.943
6.922
6.870
6.856
6.839
6.813
6.800
6.780
6.767
6.754
6.568
6.530
6.518
600MHz
$CDCl_3$
Br
OH
O
24
1.00
2.04
3.07
1.07
1.05
3.09
1.03
2.11
4.04
1.12
2.05
12.5
11.5
10.5
9.5
8.5
7.5
6.5
5.5
4.5
3.5
2.5
1.5
0.5
-0.5

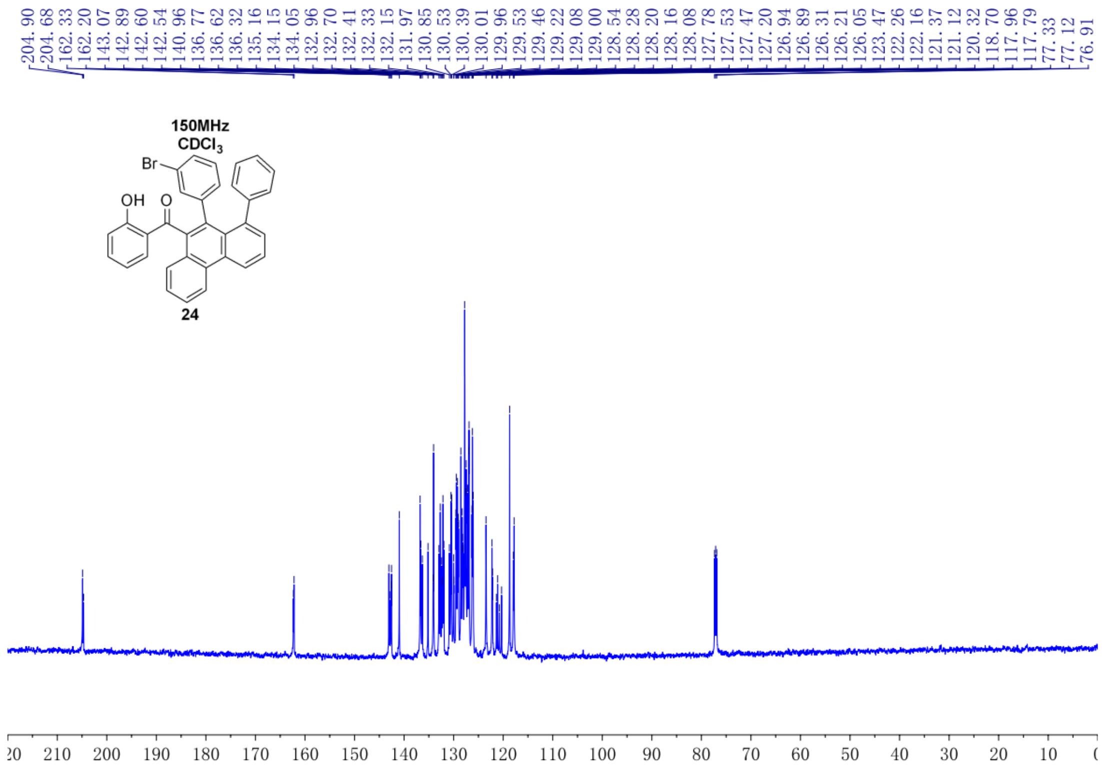
204.90
204.68
162.33
162.20
143.07
142.89
142.60
142.54
140.96
136.77
136.62
136.32
135.16
134.15
134.05
132.96
132.70
132.41
132.33
132.15
131.97
130.85
130.53
130.39
130.01
129.96
129.53
129.46
129.22
129.08
129.00
128.54
128.28
128.20
128.16
128.08
127.78
127.53
127.47
127.20
126.94
126.89
126.31
126.21
126.05
123.47
122.26
122.16
121.37
121.12
120.32
118.70
117.96
117.79
77.33
77.12
76.91
150MHz
$CDCl_3$
Br
OH
O
24
20
210
200
190
180
170
160
150
140
130
120
110
100
90
80
70
60
50
40
30
20
10
(

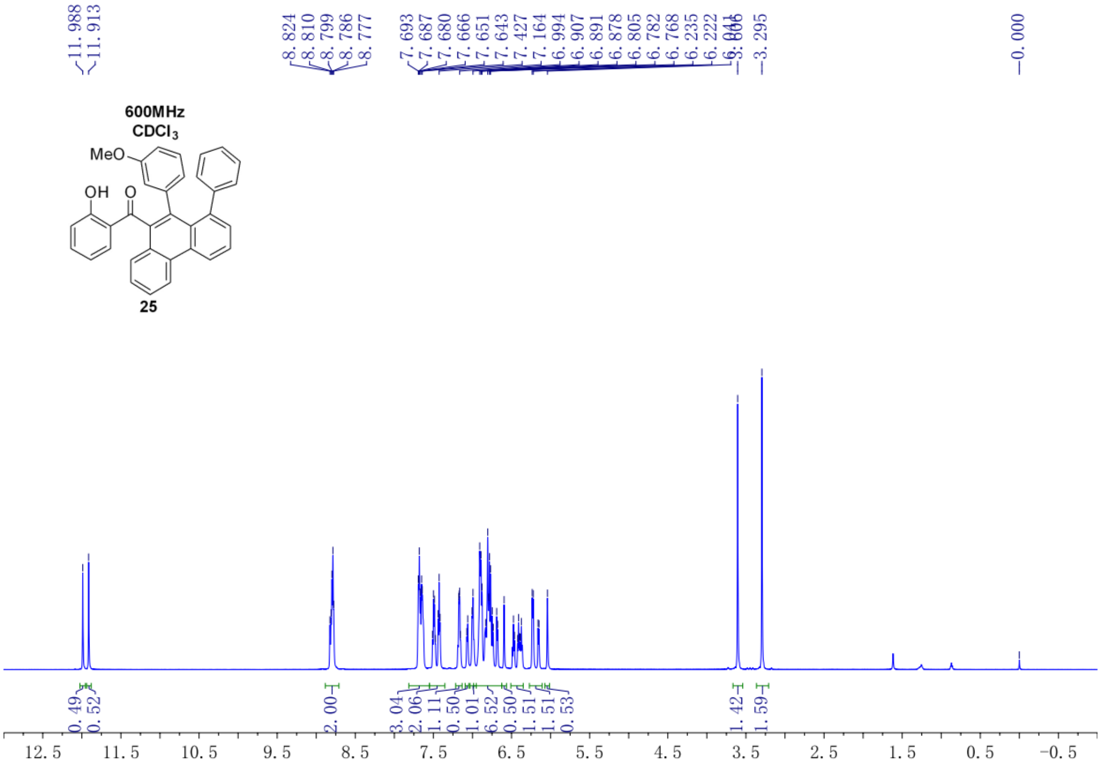
11.988
11.913
8.824
8.810
8.799
8.786
8.777
7.693
7.687
7.680
7.666
7.651
7.643
7.427
7.164
6.994
6.907
6.891
6.878
6.805
6.782
6.768
6.235
6.222
3.295
0.000
600MHz
$CDCl_3$
MeO
OH
O
25
0.49
0.52
2.00
3.04
2.06
1.11
0.50
1.01
6.52
0.50
1.51
1.51
0.53
1.42
1.59
12.5 11.5 10.5 9.5 8.5 7.5 6.5 5.5 4.5 3.5 2.5 1.5 0.5 -0.5

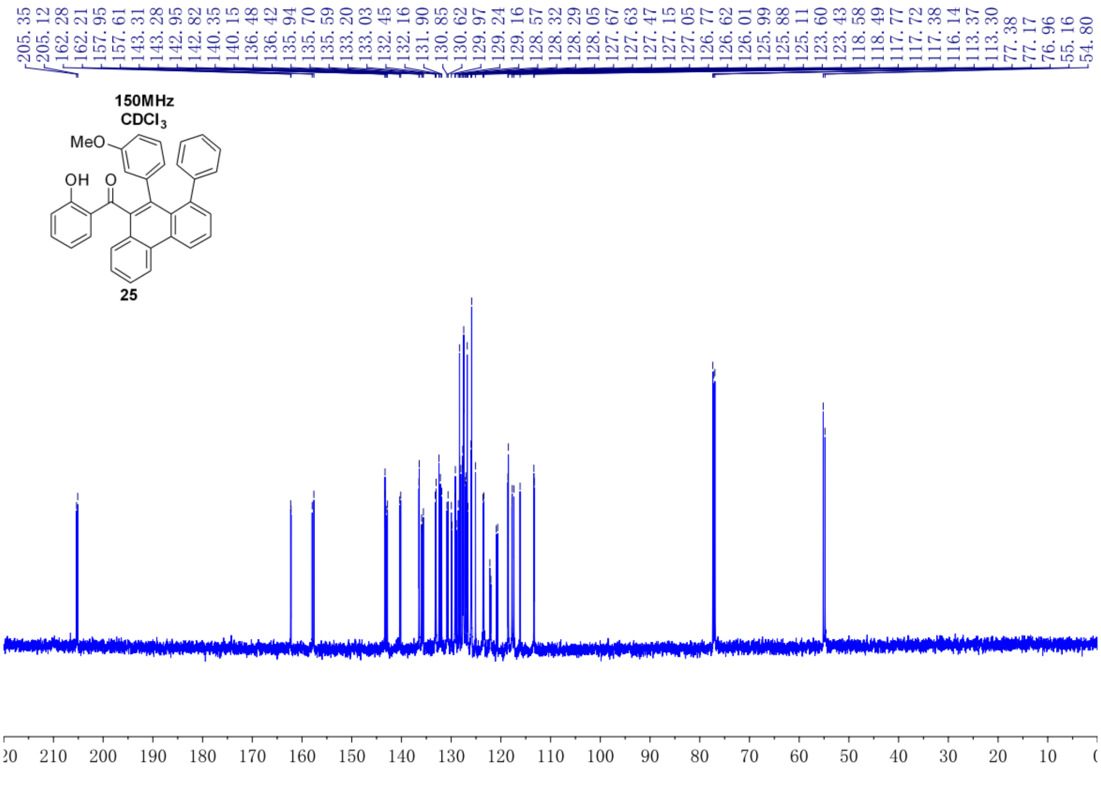
205.35
205.12
162.28
162.21
157.95
157.61
143.31
143.28
142.95
142.82
140.35
140.15
136.48
136.42
135.94
135.70
135.59
133.20
133.03
132.45
132.16
131.90
130.85
130.62
129.97
129.24
129.16
128.57
128.32
128.29
128.05
127.67
127.63
127.47
127.15
127.05
126.77
126.62
126.01
125.99
125.88
125.11
123.60
123.43
118.58
118.49
117.77
117.72
117.38
116.14
113.37
113.30
77.38
77.17
76.96
55.16
54.80
150MHz
$CDCl_3$
MeO
OH
O
25
210 200 190 180 170 160 150 140 130 120 110 100 90 80 70 60 50 40 30 20 10

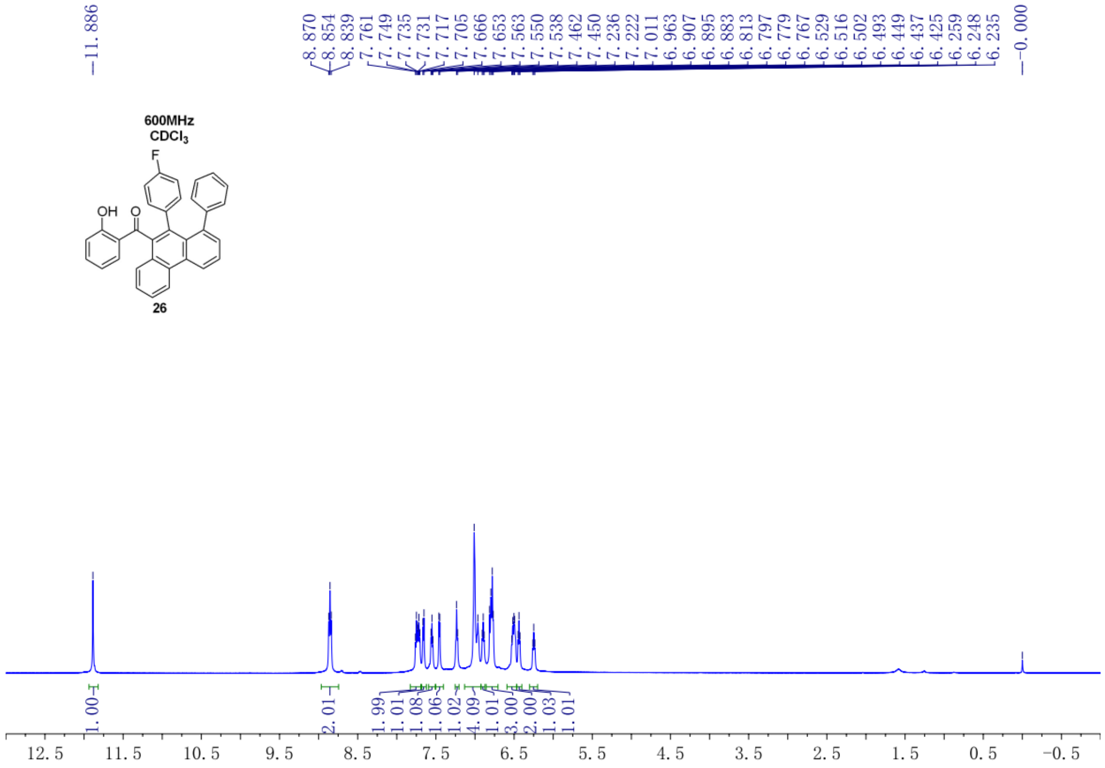
600MHz
CDCl3
26

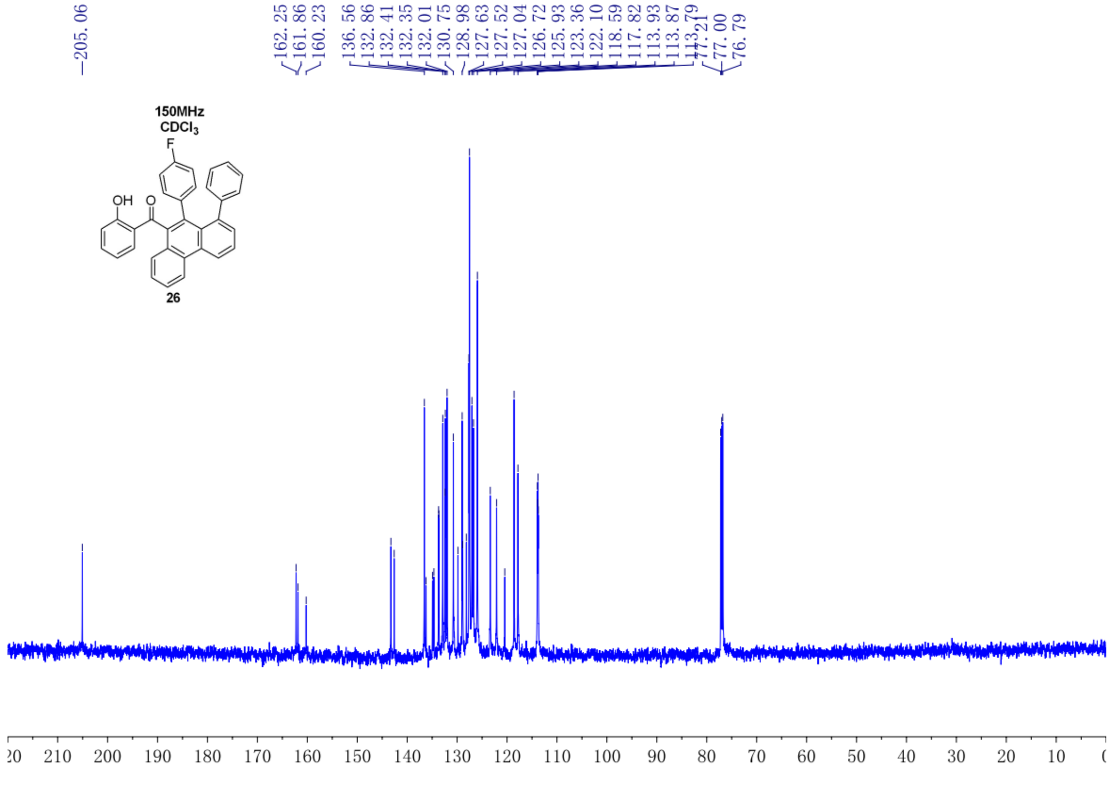
150MHz
CDCl3
26

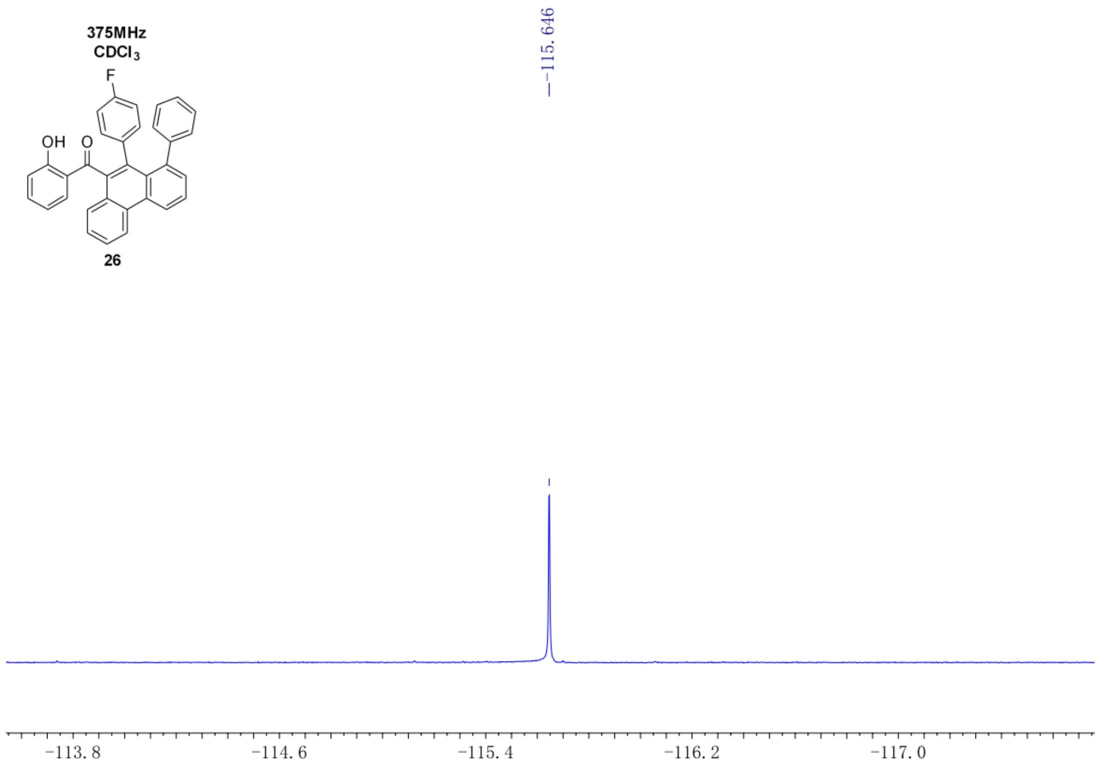
375MHz
$CDCl_3$
F
OH
O
26
−115.646
−113.8
−114.6
−115.4
−116.2
−117.0

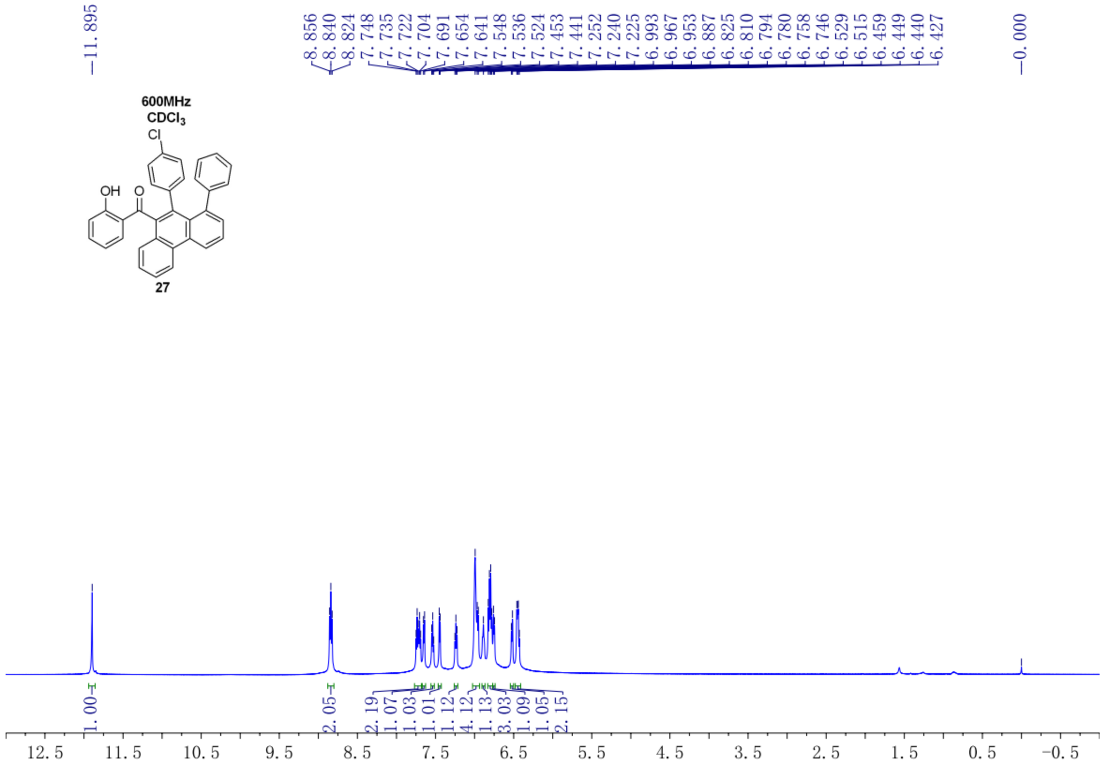
−11.895
8.856
8.840
8.824
7.748
7.735
7.722
7.704
7.691
7.654
7.641
7.548
7.536
7.524
7.453
7.441
7.252
7.240
7.225
6.993
6.967
6.953
6.887
6.825
6.810
6.794
6.780
6.758
6.746
6.529
6.515
6.459
6.449
6.440
6.427
−0.000
600MHz
$CDCl_3$
Cl
OH
O
27
1.00
2.05
2.19
1.07
1.03
1.01
1.12
4.12
1.13
3.03
1.09
1.05
2.15
12.5
11.5
10.5
9.5
8.5
7.5
6.5
5.5
4.5
3.5
2.5
1.5
0.5
−0.5

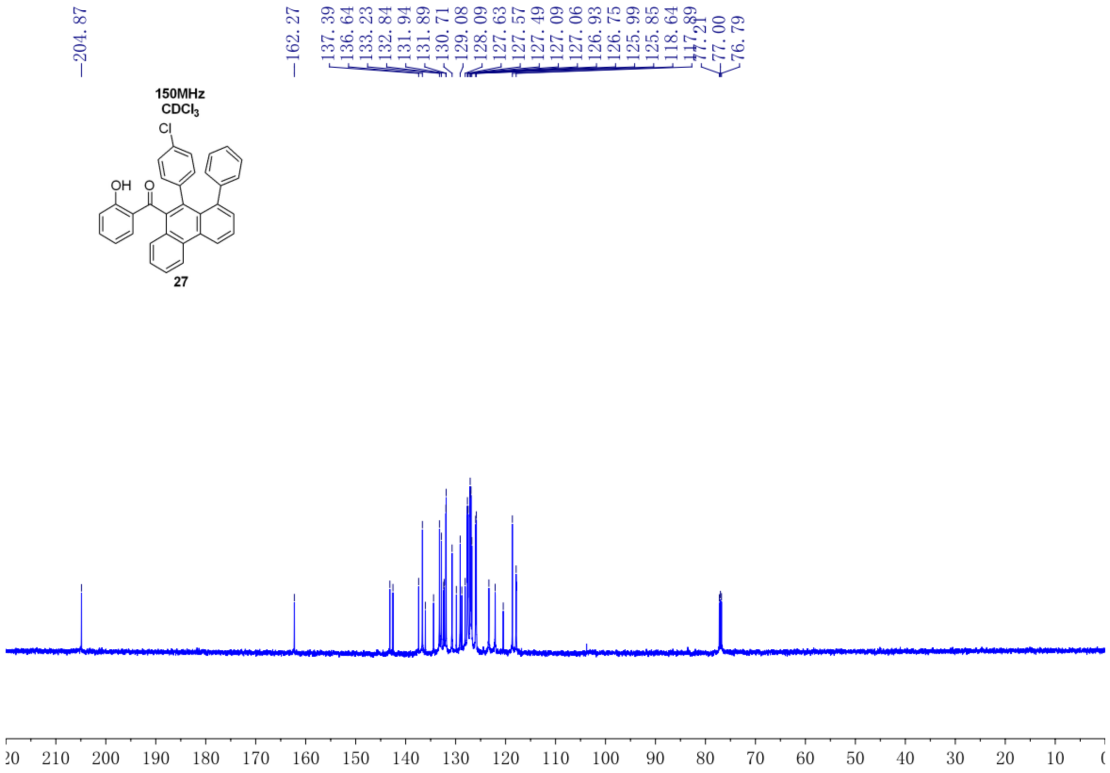
204.87
162.27
137.39
136.64
133.23
132.84
131.94
131.89
130.71
129.08
128.09
127.63
127.57
127.49
127.09
127.06
126.93
126.75
125.99
125.85
118.64
117.89
77.21
77.00
76.79
150MHz
$CDCl_3$
Cl
OH
O
27
20 210 200 190 180 170 160 150 140 130 120 110 100 90 80 70 60 50 40 30 20 10

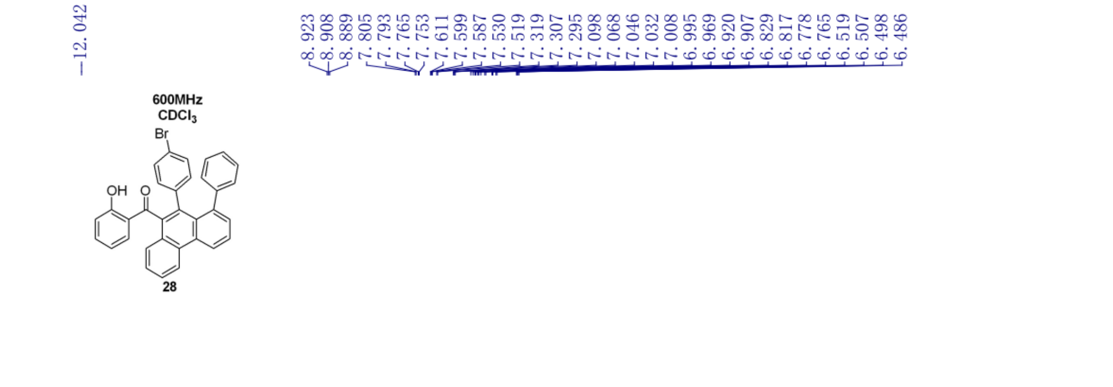
12.042
8.923
8.908
8.889
7.805
7.793
7.765
7.753
7.611
7.599
7.587
7.530
7.519
7.307
7.295
7.098
7.068
7.046
7.032
7.008
6.995
6.969
6.920
6.907
6.829
6.817
6.778
6.765
6.519
6.507
6.498
6.486
600MHz
$CDCl_3$
Br
OH
O
28
1.00
2.04
3.07
1.11
1.07
1.07
4.19
2.07
2.00
1.06
1.01
2.00
12.5 11.5 10.5 9.5 8.5 7.5 6.5 5.5 4.5 3.5 2.5 1.5 0.5 −0.5

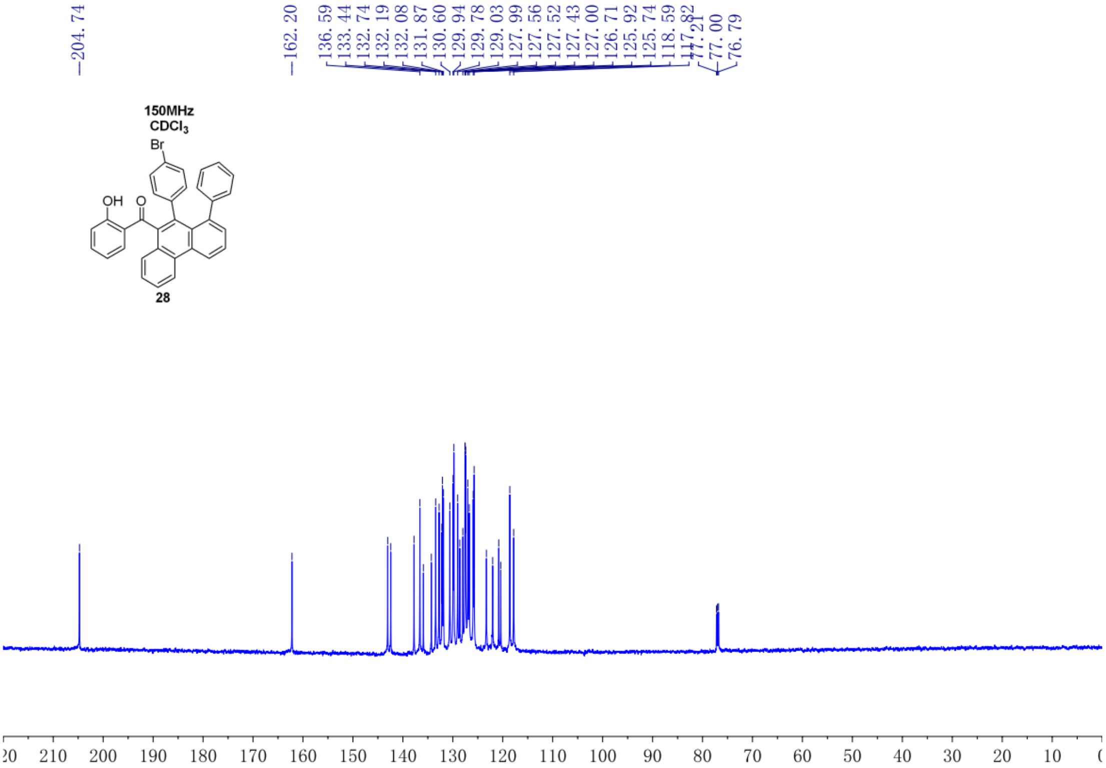
204.74
162.20
136.59
133.44
132.74
132.19
132.08
131.87
130.60
129.94
129.78
129.03
127.99
127.56
127.52
127.43
127.00
126.71
125.92
125.74
118.59
117.82
77.21
77.00
76.79
150MHz
$CDCl_3$
Br
OH
O
28
220 210 200 190 180 170 160 150 140 130 120 110 100 90 80 70 60 50 40 30 20 10 0

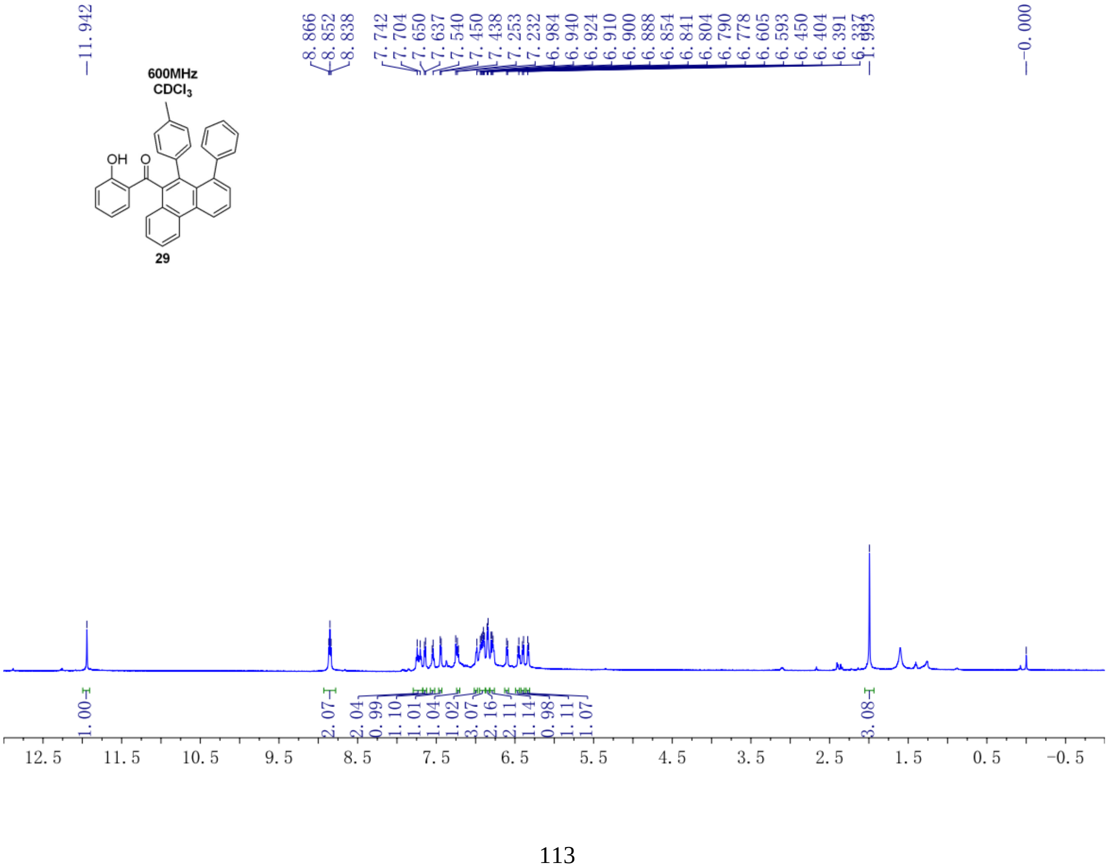
11.942
8.866
8.852
8.838
7.742
7.704
7.650
7.637
7.540
7.450
7.438
7.253
7.232
6.984
6.940
6.924
6.910
6.900
6.888
6.854
6.841
6.804
6.790
6.778
6.605
6.593
6.450
6.404
6.391
6.337
1.993
0.000
600MHz
$CDCl_3$
OH
O
29
1.00
2.07
2.04
0.99
1.10
1.01
1.04
1.02
3.07
2.16
2.11
1.14
0.98
1.11
1.07
3.08
12.5 11.5 10.5 9.5 8.5 7.5 6.5 5.5 4.5 3.5 2.5 1.5 0.5 -0.5

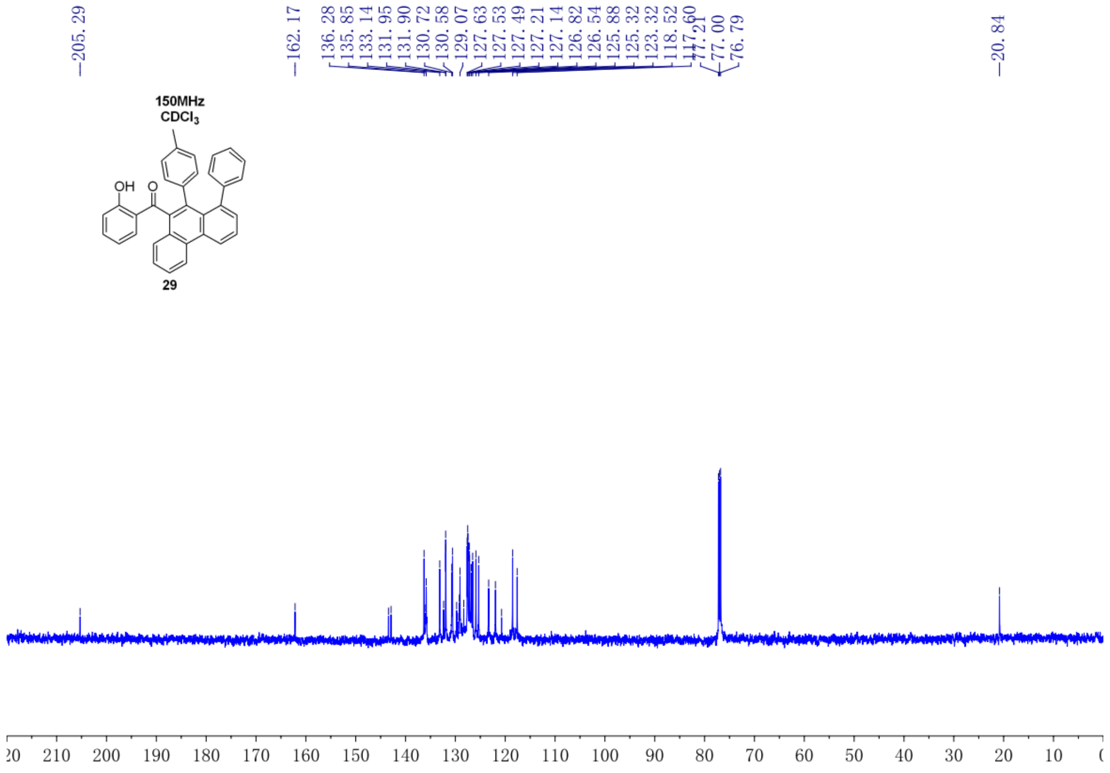
150MHz
CDCl3
OH
O
29

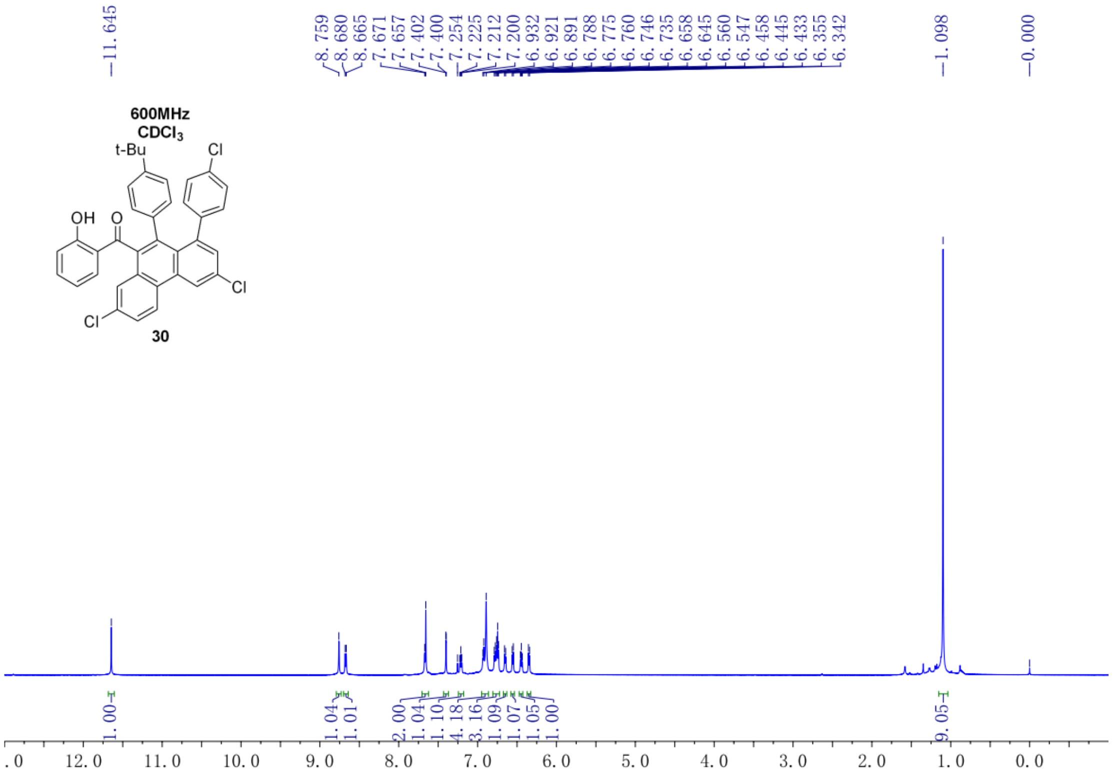
600MHz
CDCl3
t-Bu
Cl
OH
O
Cl
Cl
30

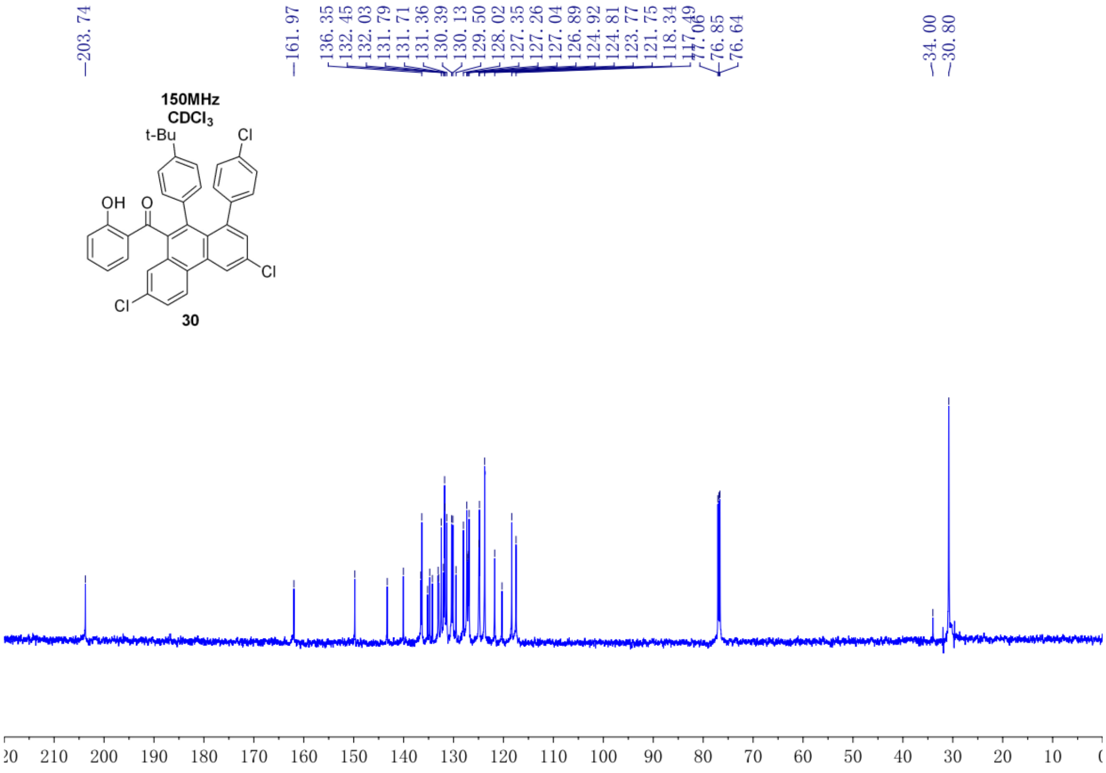
150MHz
CDCl3
t-Bu
Cl
OH
O
Cl
Cl
30
203.74
161.97
136.35
132.45
132.03
131.79
131.71
131.36
130.39
130.13
129.50
128.02
127.35
127.26
127.04
126.89
124.92
124.81
123.77
121.75
118.34
117.49
77.06
76.85
76.64
34.00
30.80
210 200 190 180 170 160 150 140 130 120 110 100 90 80 70 60 50 40 30 20 10

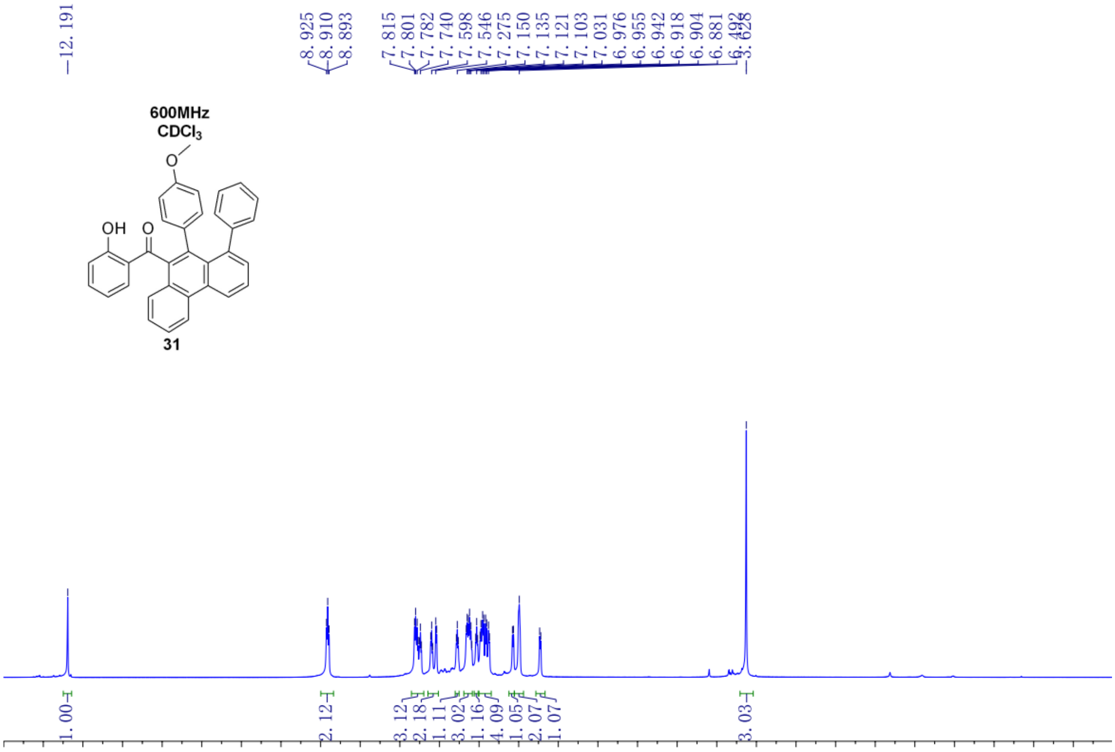
600MHz
CDCl3
O
OH
O
31
12.191
8.925
8.910
8.893
7.815
7.801
7.782
7.740
7.598
7.546
7.275
7.150
7.135
7.121
7.103
7.031
6.976
6.955
6.942
6.918
6.904
6.881
6.492
3.628
1.00
2.12
3.12
2.18
1.11
3.02
1.16
4.09
1.05
2.07
1.07
3.03
12.5 11.5 10.5 9.5 8.5 7.5 6.5 5.5 4.5 3.5 2.5 1.5 0.5 -0.5

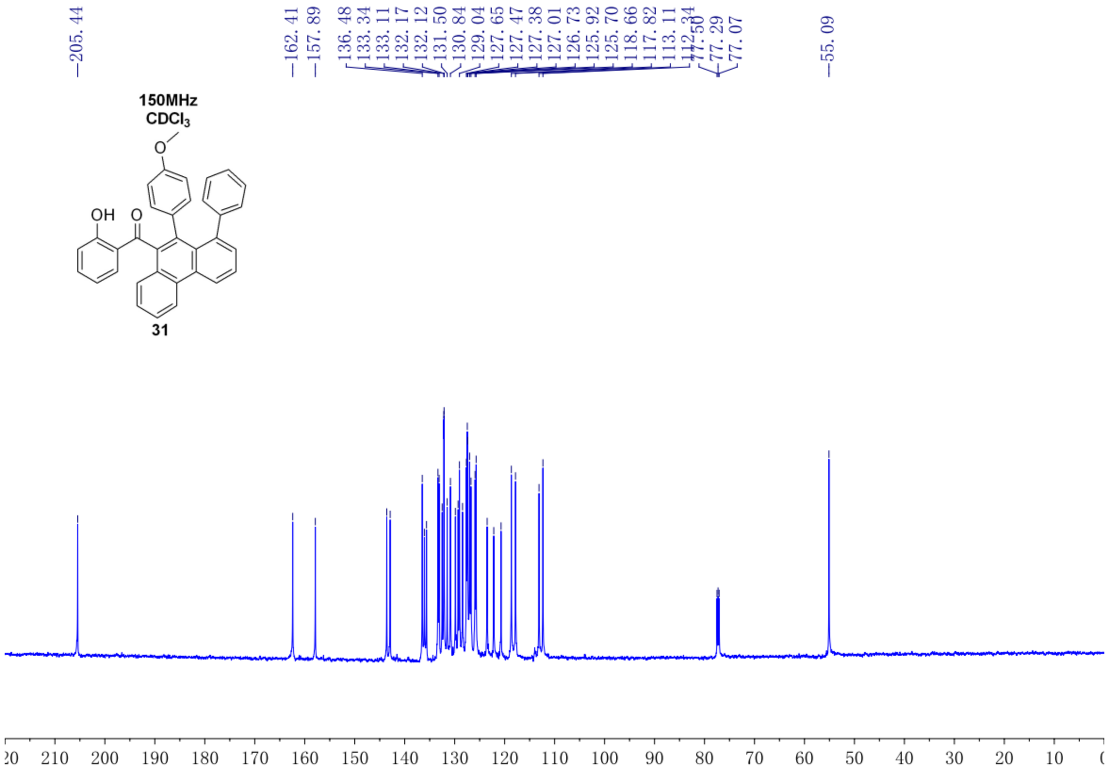
205.44
162.41
157.89
136.48
133.34
133.11
132.17
132.12
131.50
130.84
129.04
127.65
127.47
127.38
127.01
126.73
125.92
125.70
118.66
117.82
113.11
112.34
77.50
77.29
77.07
55.09
150MHz
$CDCl_3$
O
OH
O
31
210 200 190 180 170 160 150 140 130 120 110 100 90 80 70 60 50 40 30 20 10

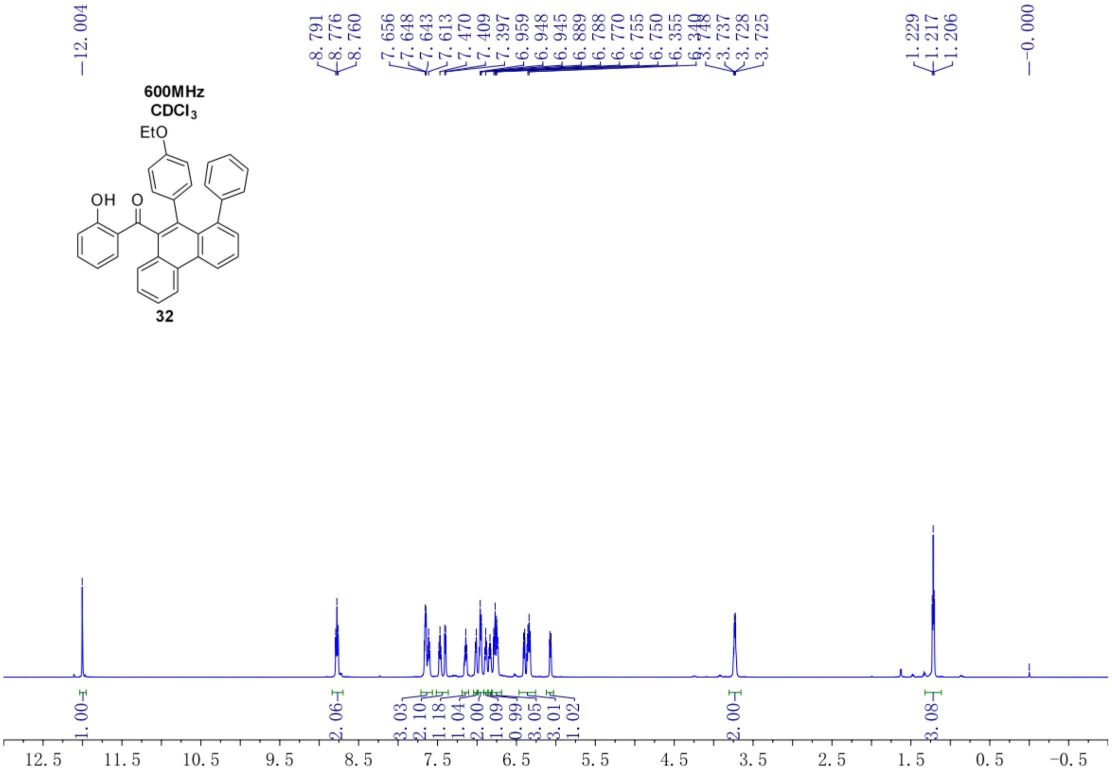
12.004
8.791
8.776
8.760
7.656
7.648
7.643
7.613
7.470
7.409
7.397
6.959
6.948
6.945
6.889
6.788
6.770
6.755
6.750
6.355
3.737
3.728
3.725
1.229
1.217
1.206
-0.000
600MHz
$CDCl_3$
EtO
OH
O
32
1.00
2.06
3.03
2.10
1.18
1.04
2.00
1.09
0.99
3.05
3.01
1.02
2.00
3.08
12.5 11.5 10.5 9.5 8.5 7.5 6.5 5.5 4.5 3.5 2.5 1.5 0.5 -0.5

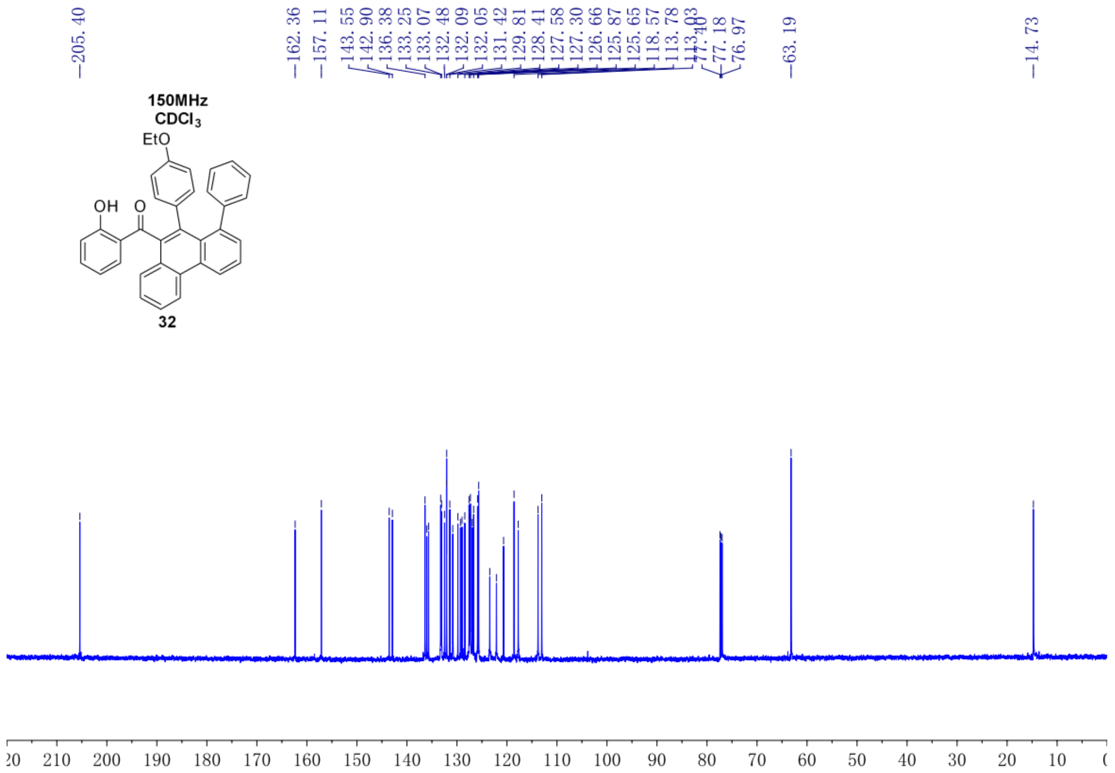

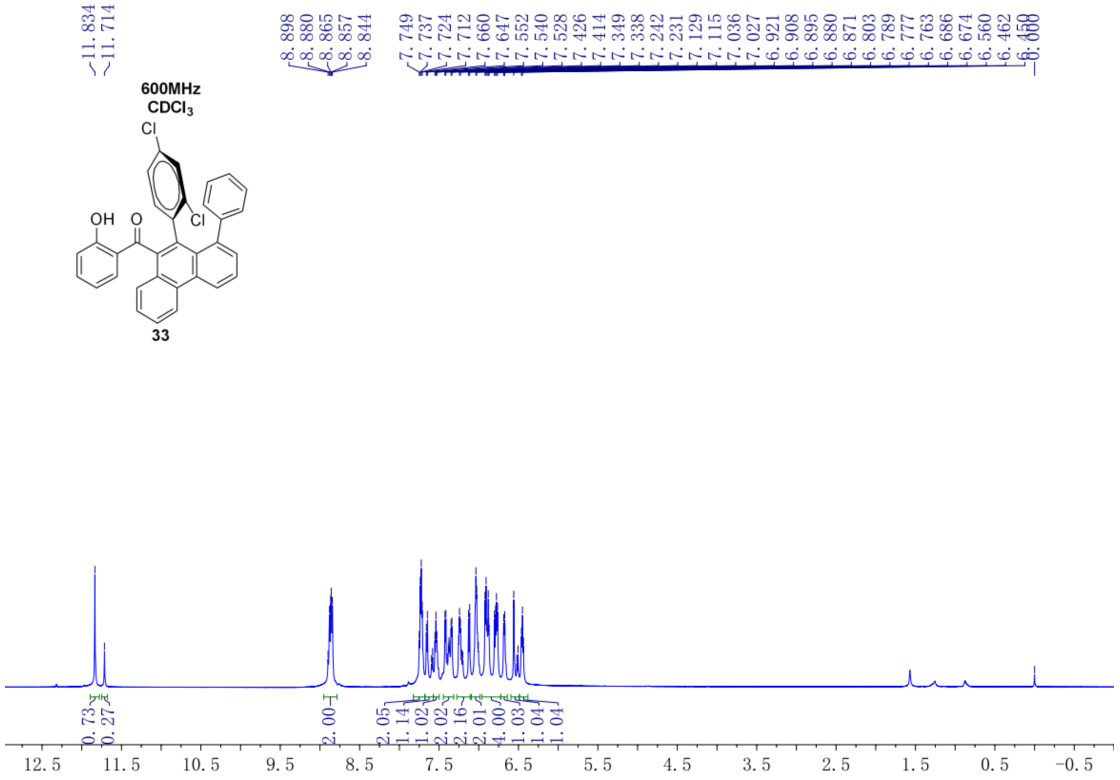

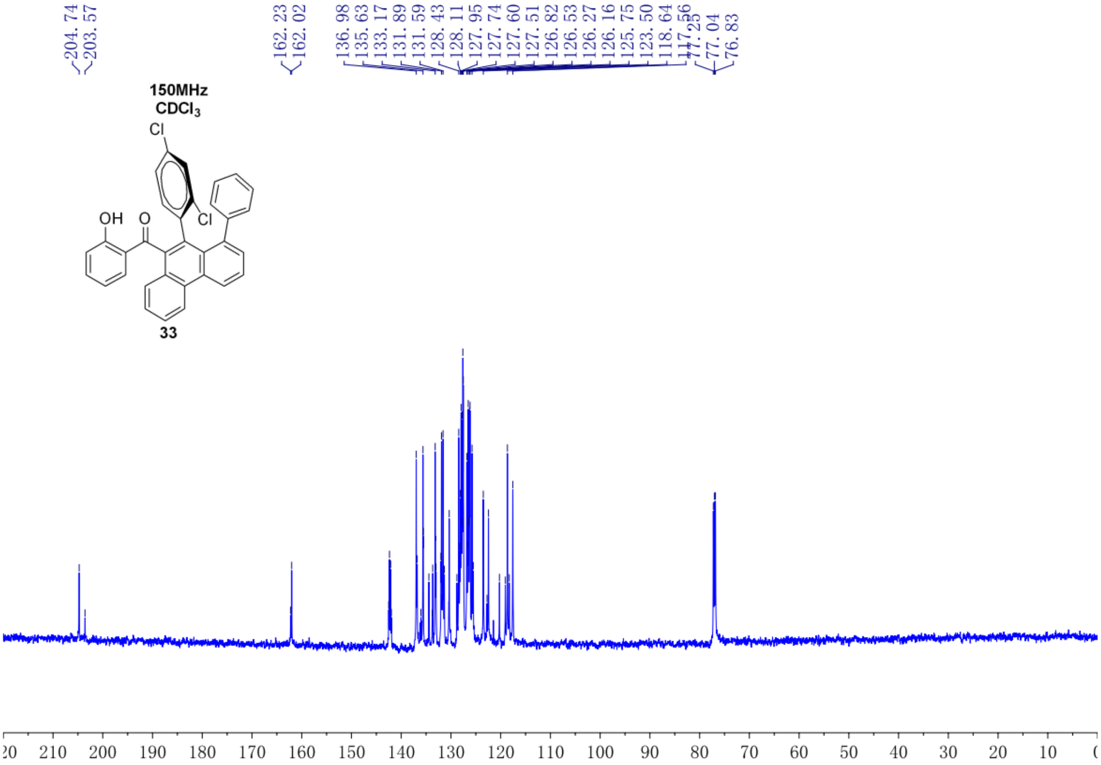
204.74
203.57
162.23
162.02
136.98
135.63
133.17
131.89
131.59
128.43
128.11
127.95
127.74
127.60
127.51
126.82
126.53
126.27
126.16
125.75
123.50
118.64
117.56
77.25
77.04
76.83
150MHz
$CDCl_3$
Cl
Cl
OH
O
33
210 200 190 180 170 160 150 140 130 120 110 100 90 80 70 60 50 40 30 20 10

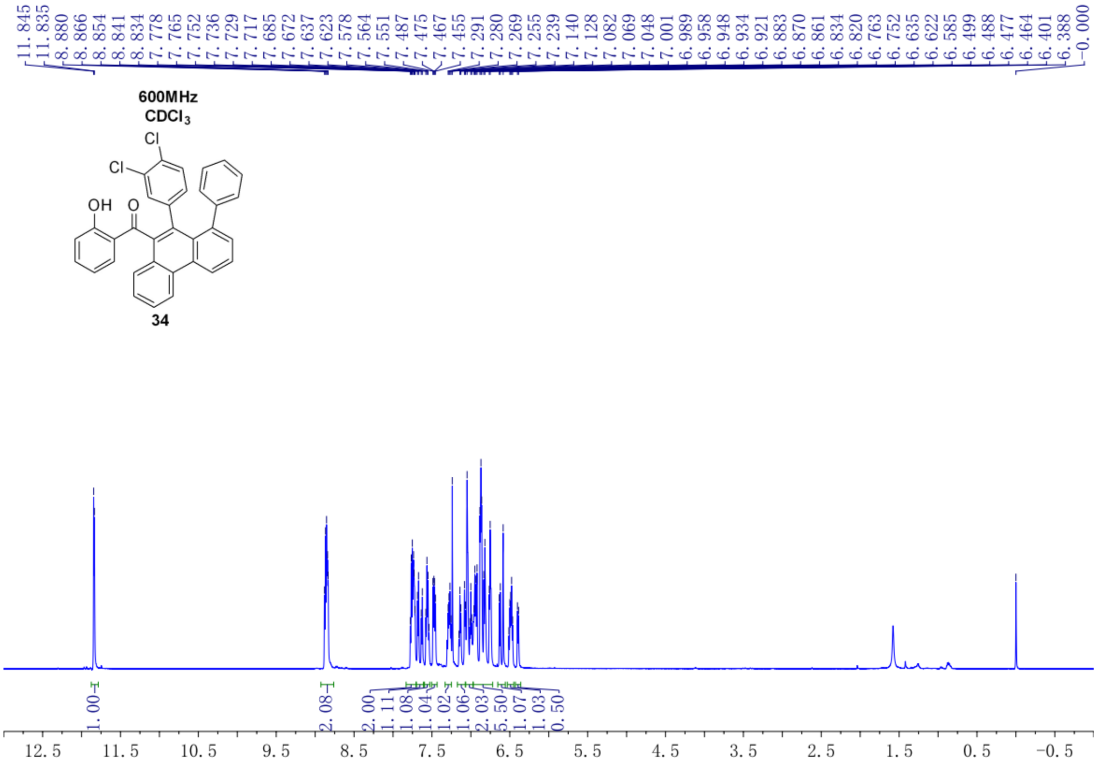
11.845
11.835
8.880
8.866
8.854
8.841
8.834
7.778
7.765
7.752
7.736
7.729
7.717
7.685
7.672
7.637
7.623
7.578
7.564
7.551
7.487
7.475
7.467
7.455
7.291
7.280
7.269
7.255
7.239
7.140
7.128
7.082
7.069
7.048
7.001
6.989
6.958
6.948
6.934
6.921
6.883
6.870
6.861
6.834
6.820
6.763
6.752
6.635
6.622
6.585
6.499
6.488
6.477
6.464
6.401
6.388
-0.000
600MHz
$CDCl_3$
Cl
Cl
OH
O
34
1.00
2.08
2.00
1.11
1.08
1.04
1.02
1.06
2.03
5.50
1.07
1.03
0.50
12.5 11.5 10.5 9.5 8.5 7.5 6.5 5.5 4.5 3.5 2.5 1.5 0.5 -0.5

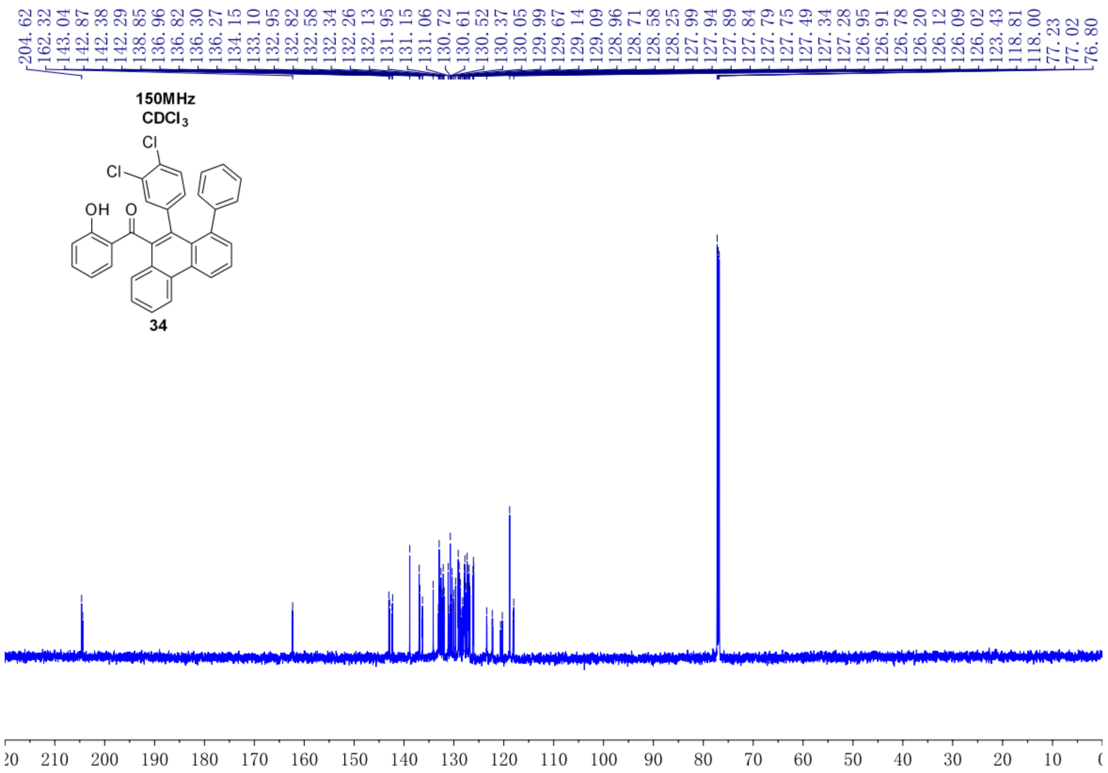
204.62 162.32 143.04 142.87 142.38 142.29 138.85 136.96 136.82 136.30 136.27 134.15 133.10 132.95 132.82 132.58 132.34 132.26 132.13 131.95 131.15 131.06 130.72 130.61 130.52 130.37 130.05 129.99 129.67 129.14 129.09 128.96 128.71 128.58 128.25 127.99 127.94 127.89 127.84 127.79 127.75 127.49 127.34 127.28 126.95 126.91 126.78 126.20 126.12 126.09 126.02 123.43 118.81 118.00 77.23 77.02 76.80
150MHz
CDCl3
Cl
Cl
OH
O
34
210 200 190 180 170 160 150 140 130 120 110 100 90 80 70 60 50 40 30 20 10

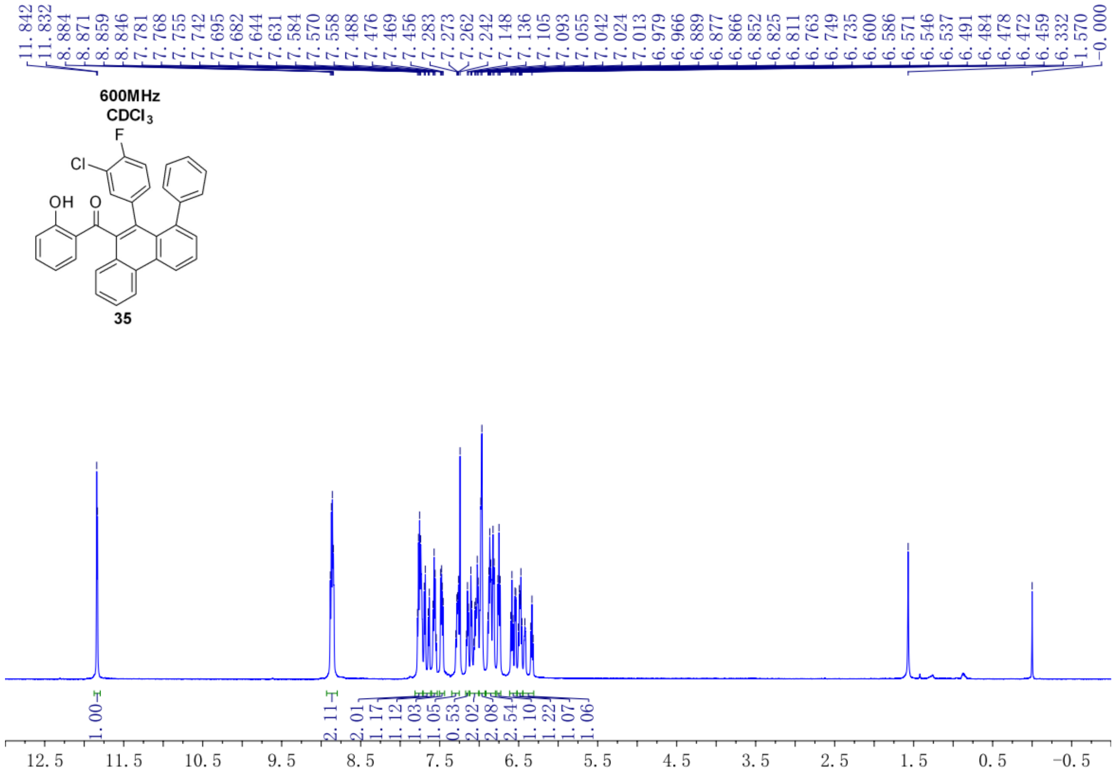
11.842 11.832 8.884 8.871 8.859 8.846 7.781 7.768 7.755 7.742 7.695 7.682 7.644 7.631 7.584 7.570 7.558 7.488 7.476 7.469 7.456 7.283 7.273 7.262 7.242 7.148 7.136 7.105 7.093 7.055 7.042 7.024 7.013 6.979 6.966 6.889 6.877 6.866 6.852 6.825 6.811 6.763 6.749 6.735 6.600 6.586 6.571 6.546 6.537 6.491 6.484 6.478 6.472 6.459 6.332 1.570 -0.000
600MHz
CDCl3
F
Cl
OH
O
35
1.00 2.11 2.01 1.17 1.12 1.03 1.05 0.53 2.02 2.08 2.54 1.10 1.22 1.07 1.06
12.5 11.5 10.5 9.5 8.5 7.5 6.5 5.5 4.5 3.5 2.5 1.5 0.5 -0.5

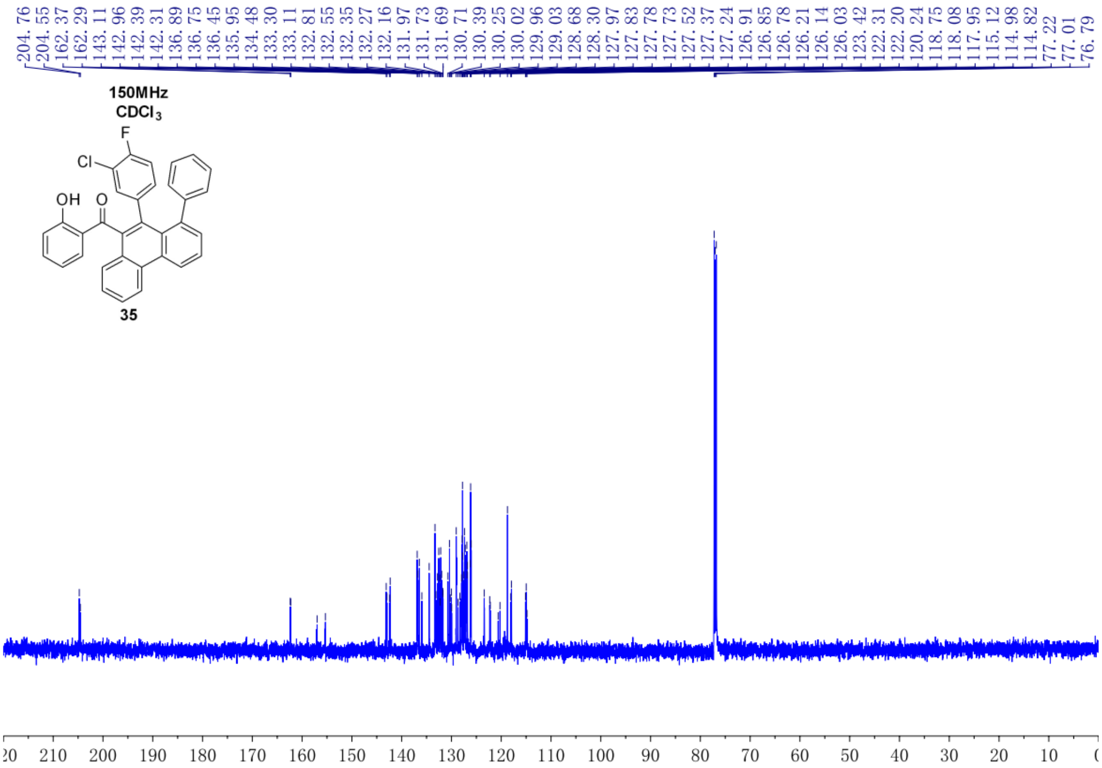
204.76
204.55
162.37
162.29
143.11
142.96
142.39
142.31
136.89
136.75
136.45
135.95
134.48
133.30
133.11
132.81
132.55
132.35
132.27
132.16
131.97
131.73
131.69
130.71
130.39
130.25
130.02
129.96
129.03
128.68
128.30
127.97
127.83
127.78
127.73
127.52
127.37
127.24
126.91
126.85
126.78
126.21
126.14
126.03
123.42
122.31
122.20
120.24
118.75
118.08
117.95
115.12
114.98
114.82
77.22
77.01
76.79
150MHz
CDCl3
F
Cl
OH
O
35
210 200 190 180 170 160 150 140 130 120 110 100 90 80 70 60 50 40 30 20 10

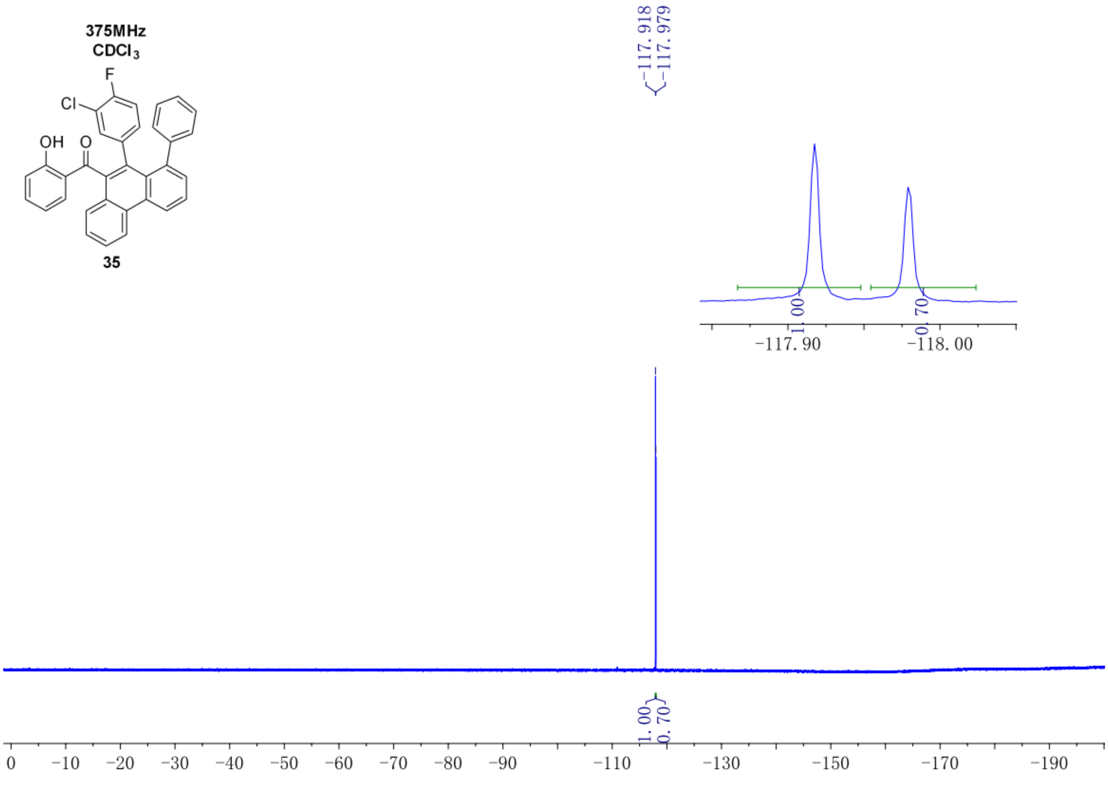
375MHz
CDCl3
F
Cl
OH
O
35
-117.918
-117.979
1.00
0.70
-117.90
-118.00
0 -10 -20 -30 -40 -50 -60 -70 -80 -90 -110 -130 -150 -170 -190

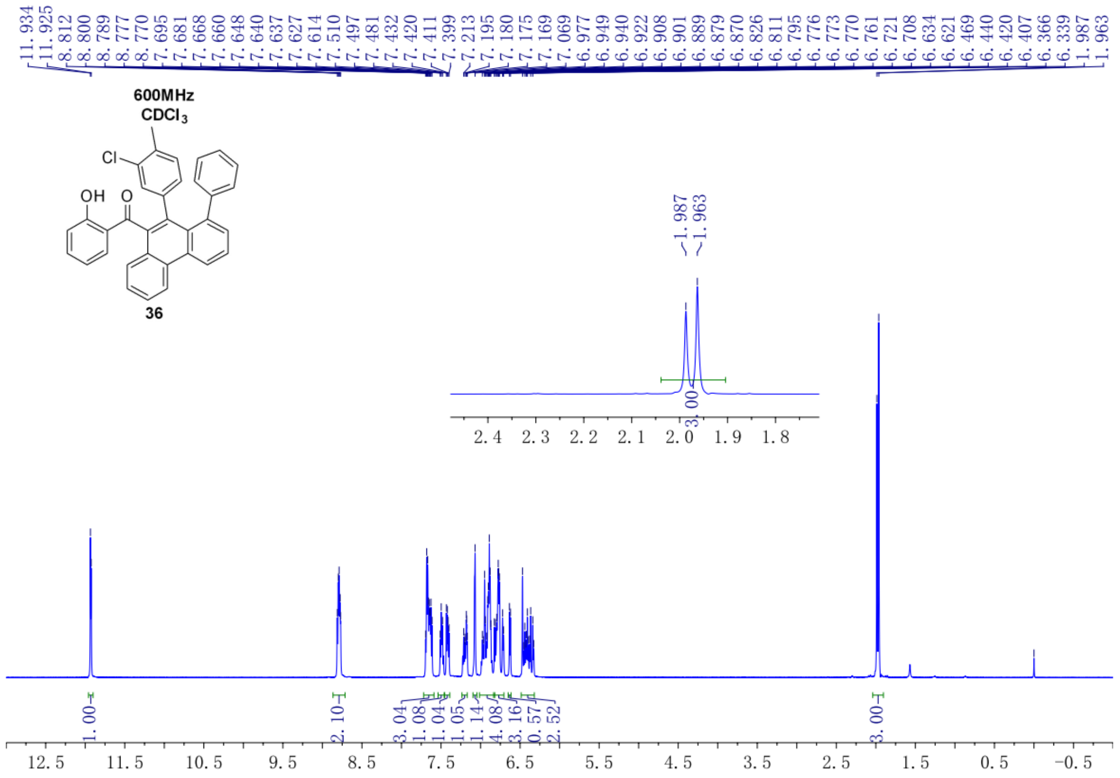

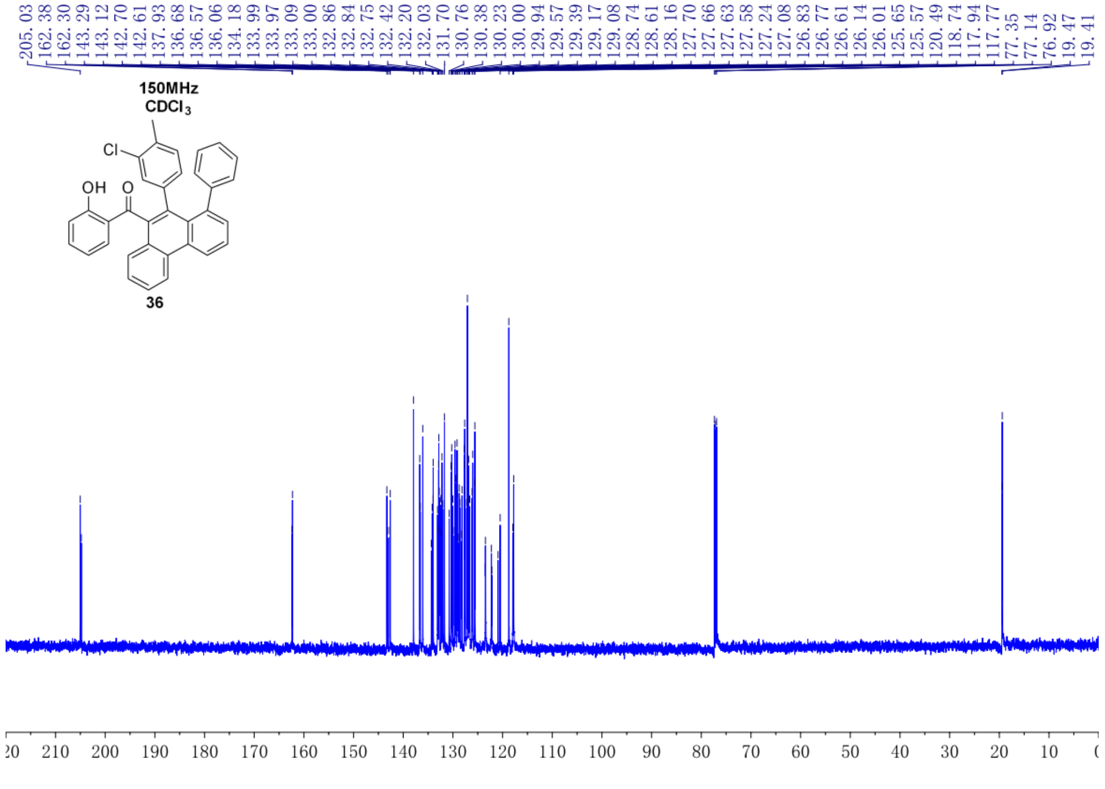

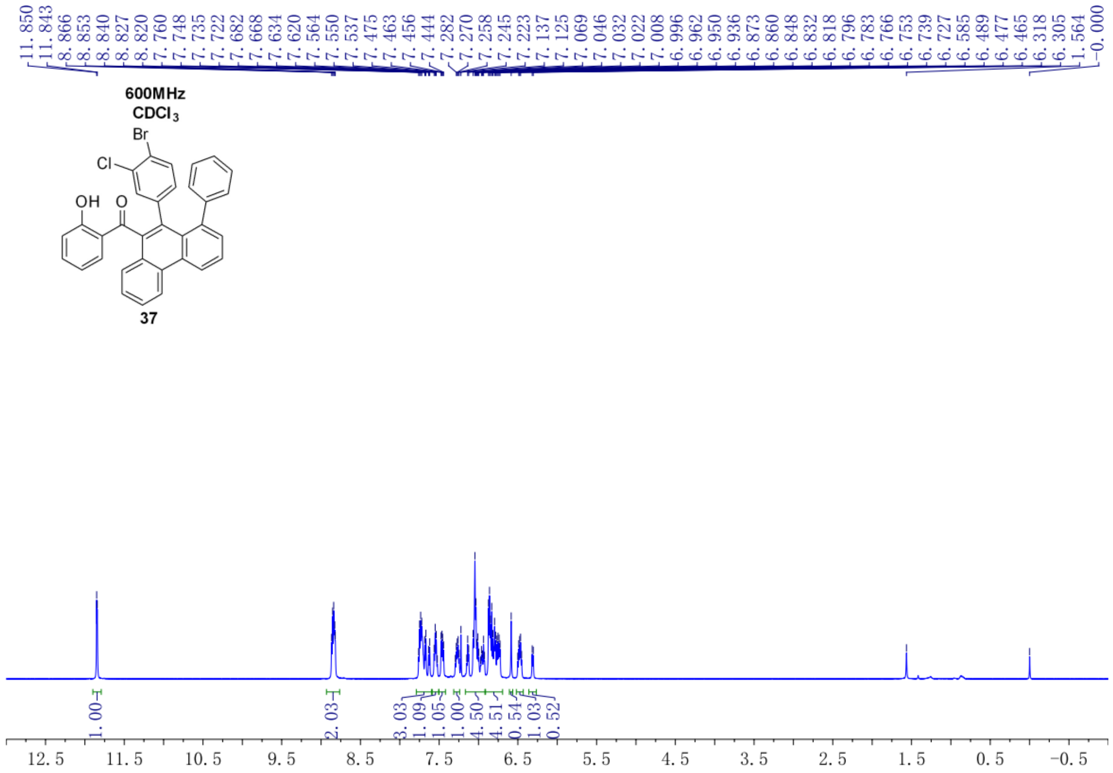
11.850
11.843
8.866
8.853
8.840
8.827
8.820
7.760
7.748
7.735
7.722
7.682
7.668
7.634
7.620
7.564
7.550
7.537
7.475
7.463
7.456
7.444
7.282
7.270
7.258
7.245
7.223
7.137
7.125
7.069
7.046
7.032
7.022
7.008
6.996
6.962
6.950
6.936
6.873
6.860
6.848
6.832
6.818
6.796
6.783
6.766
6.753
6.739
6.727
6.585
6.489
6.477
6.465
6.318
6.305
1.564
-0.000
600MHz
CDCl3
Br
Cl
OH
O
37
1.00
2.03
3.03
1.09
1.05
1.00
4.50
4.51
0.54
1.03
0.52
12.5
11.5
10.5
9.5
8.5
7.5
6.5
5.5
4.5
3.5
2.5
1.5
0.5
-0.5

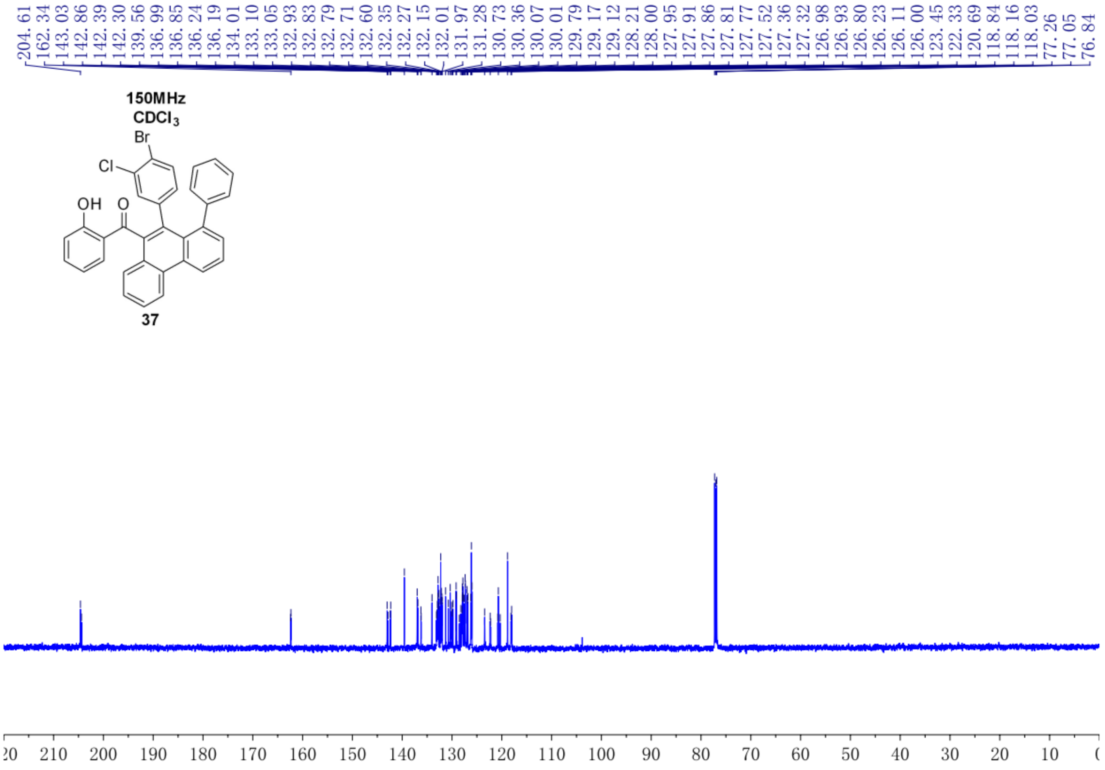
204.61
162.34
143.03
142.86
142.39
142.30
139.56
136.99
136.85
136.24
136.19
134.01
133.10
133.05
132.93
132.83
132.79
132.71
132.60
132.35
132.27
132.15
132.01
131.97
131.28
130.73
130.36
130.07
130.01
129.79
129.17
129.12
128.21
128.00
127.95
127.91
127.86
127.81
127.77
127.52
127.36
127.32
126.98
126.93
126.80
126.23
126.11
126.00
123.45
122.33
120.69
118.84
118.16
118.03
77.26
77.05
76.84
150MHz
CDCl3
Br
Cl
OH
O
37
210
200
190
180
170
160
150
140
130
120
110
100
90
80
70
60
50
40
30
20
10

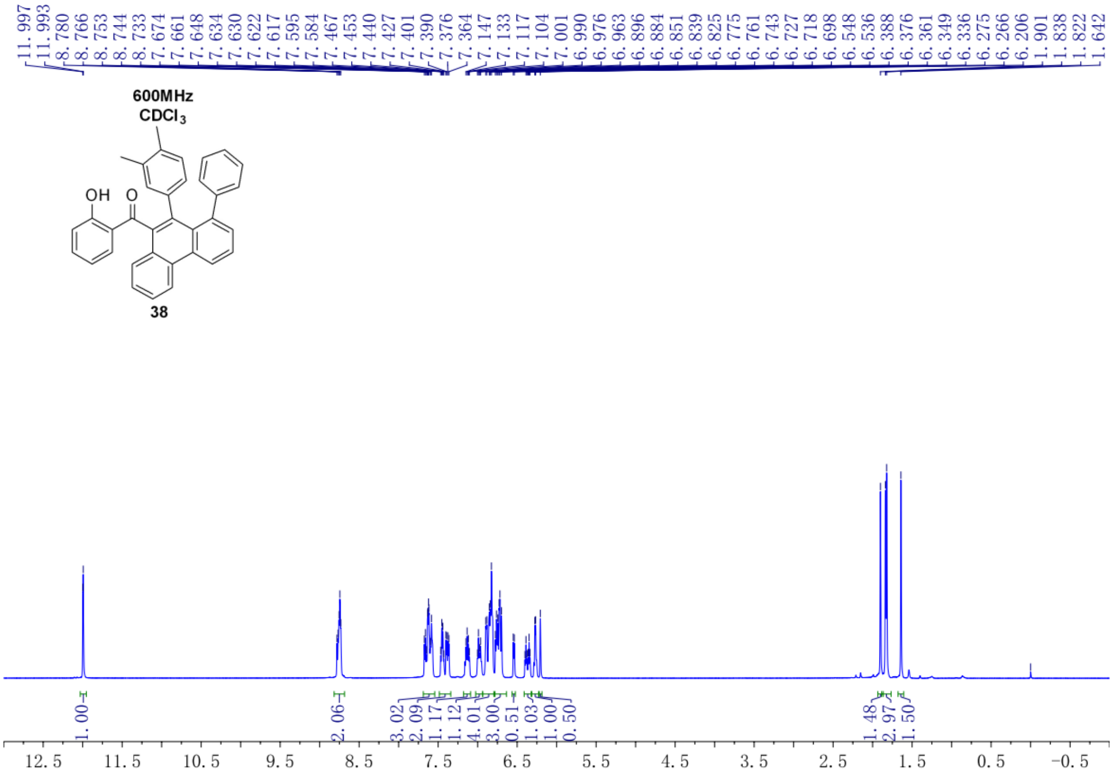
11.997
11.993
8.780
8.766
8.753
8.744
8.733
7.674
7.661
7.648
7.634
7.630
7.622
7.617
7.595
7.584
7.467
7.453
7.440
7.427
7.401
7.390
7.376
7.364
7.147
7.133
7.117
7.104
7.001
6.990
6.976
6.963
6.896
6.884
6.851
6.839
6.825
6.775
6.761
6.743
6.727
6.718
6.698
6.548
6.536
6.388
6.376
6.361
6.349
6.336
6.275
6.266
6.206
1.901
1.838
1.822
1.642
600MHz
CDCl3
OH
O
38
1.00
2.06
3.02
2.09
1.17
1.12
4.01
3.00
0.51
1.03
1.00
0.50
1.48
2.97
1.50
12.5
11.5
10.5
9.5
8.5
7.5
6.5
5.5
4.5
3.5
2.5
1.5
0.5
-0.5

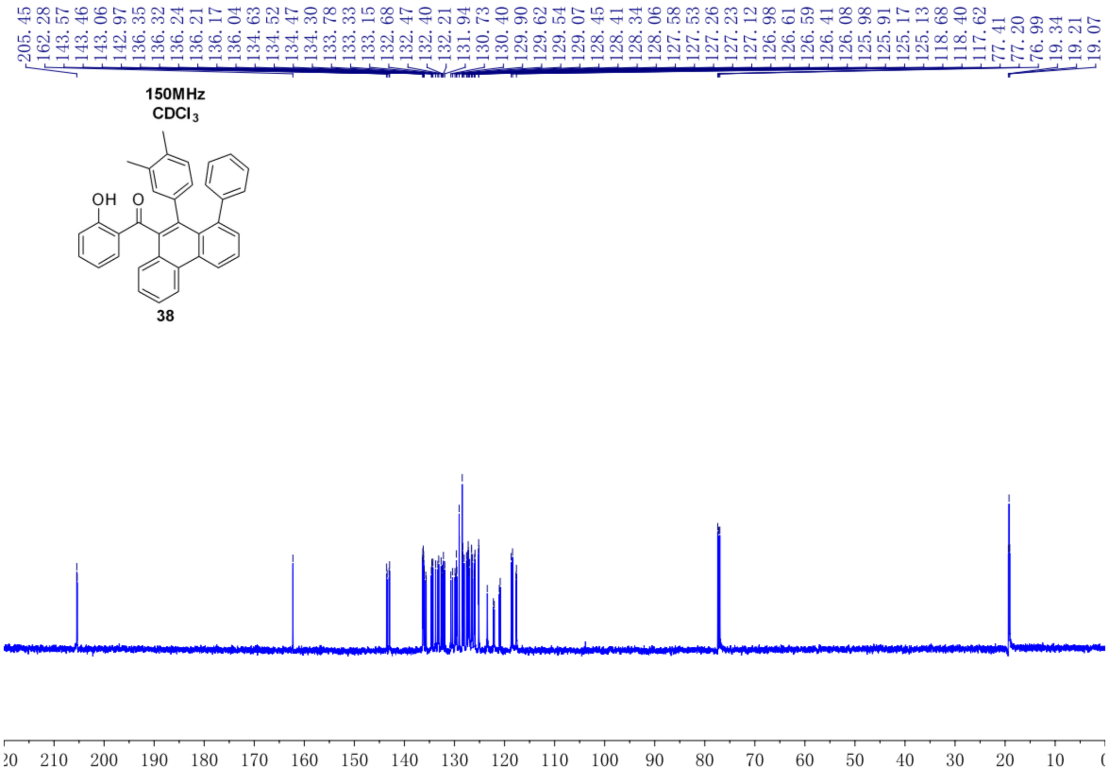
205.45
162.28
143.57
143.46
143.06
142.97
136.35
136.32
136.24
136.21
136.17
136.04
134.63
134.52
134.47
134.30
133.78
133.33
133.15
132.68
132.47
132.40
132.21
131.94
130.73
130.40
129.90
129.62
129.54
129.07
128.45
128.41
128.34
128.06
127.58
127.53
127.26
127.23
127.12
126.98
126.61
126.59
126.41
126.08
125.98
125.91
125.17
125.13
118.68
118.40
117.62
77.41
77.20
76.99
19.34
19.21
19.07
150MHz
CDCl3
OH
O
38
210
200
190
180
170
160
150
140
130
120
110
100
90
80
70
60
50
40
30
20
10

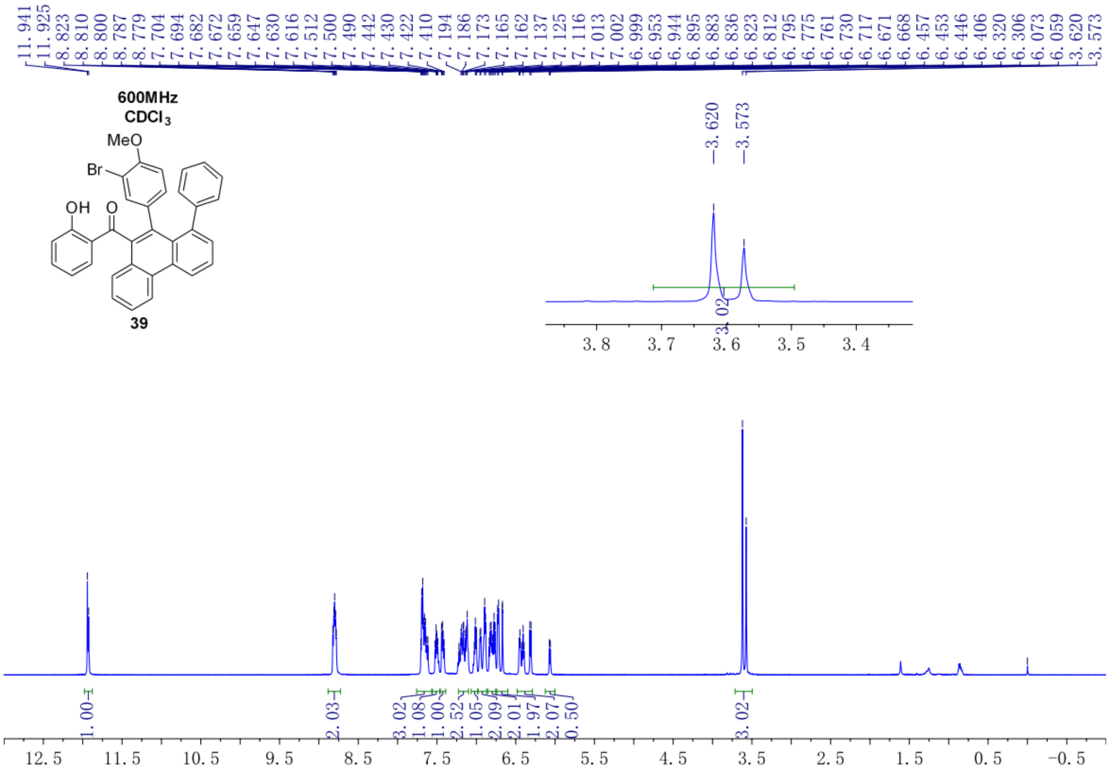
11.941
11.925
8.823
8.810
8.800
8.787
8.779
7.704
7.694
7.682
7.672
7.659
7.647
7.630
7.616
7.512
7.500
7.490
7.442
7.430
7.422
7.410
7.194
7.186
7.173
7.165
7.162
7.137
7.125
7.116
7.013
7.002
6.999
6.953
6.944
6.895
6.883
6.836
6.823
6.812
6.795
6.775
6.761
6.730
6.717
6.671
6.668
6.457
6.453
6.446
6.406
6.320
6.306
6.073
6.059
3.620
3.573
600MHz
CDCl3
MeO
Br
OH
O
39
3.620
3.573
3.02
1.00
2.03
3.02
1.08
1.00
2.52
1.05
2.09
2.01
1.97
2.07
0.50
3.02

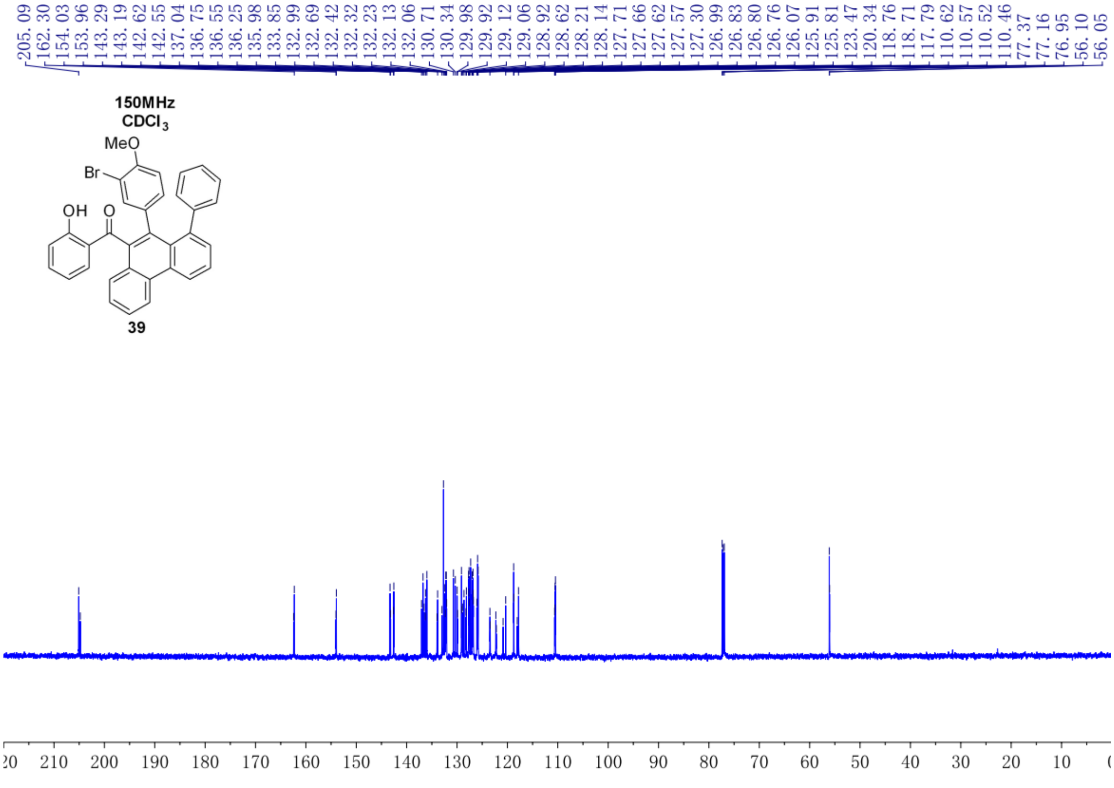
205.09
162.30
154.03
153.96
143.29
143.19
142.62
142.55
137.04
136.75
136.55
136.25
135.98
133.85
132.99
132.69
132.42
132.32
132.23
132.13
132.06
130.71
130.34
129.98
129.92
129.12
129.06
128.92
128.62
128.21
128.14
127.71
127.66
127.62
127.57
127.30
126.99
126.83
126.80
126.76
126.07
125.91
125.81
123.47
120.34
118.76
118.71
117.79
110.62
110.57
110.52
110.46
77.37
77.16
76.95
56.10
56.05
150MHz
CDCl3
MeO
Br
OH
O
39

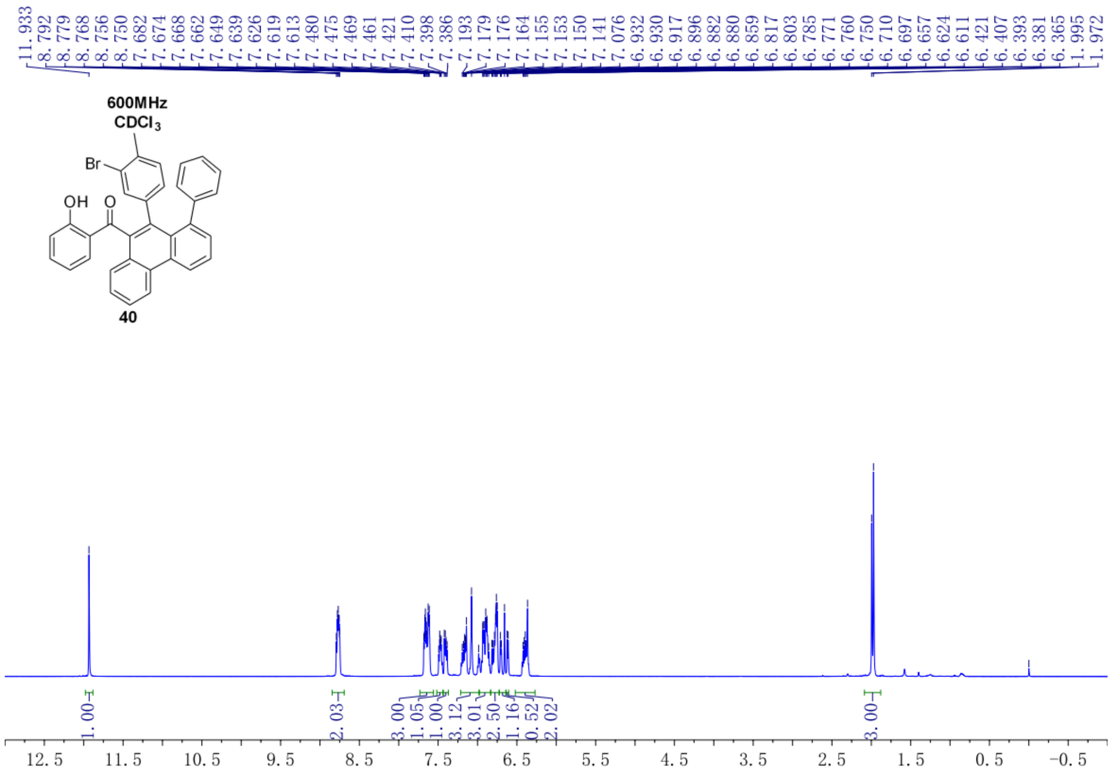
11.933
8.792
8.779
8.768
8.756
8.750
7.682
7.674
7.668
7.662
7.649
7.639
7.626
7.619
7.613
7.480
7.475
7.469
7.461
7.421
7.410
7.398
7.386
7.193
7.179
7.176
7.164
7.155
7.153
7.150
7.141
7.076
6.932
6.930
6.917
6.896
6.882
6.880
6.859
6.817
6.803
6.785
6.771
6.760
6.750
6.710
6.697
6.657
6.624
6.611
6.421
6.407
6.393
6.381
6.365
1.995
1.972
600MHz
CDCl3
Br
OH
O
40
1.00
2.03
3.00
1.05
1.00
3.12
3.01
2.50
1.16
0.52
2.02
3.00
12.5
11.5
10.5
9.5
8.5
7.5
6.5
5.5
4.5
3.5
2.5
1.5
0.5
-0.5

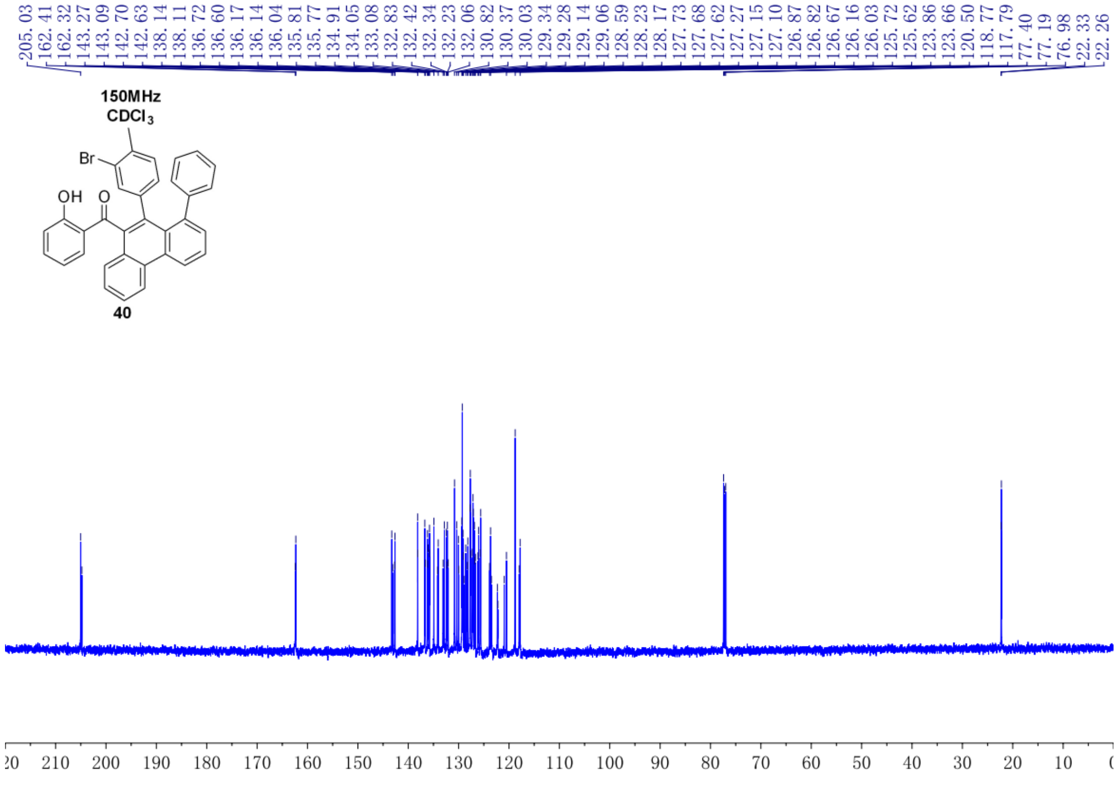
205.03
162.41
162.32
143.27
143.09
142.70
142.63
138.14
138.11
136.72
136.60
136.17
136.14
136.04
135.81
135.77
134.91
134.05
133.08
132.83
132.42
132.34
132.23
132.06
130.82
130.37
130.03
129.34
129.28
129.14
129.06
128.59
128.23
128.17
127.73
127.68
127.62
127.27
127.15
127.10
126.87
126.82
126.67
126.16
126.03
125.72
125.62
123.86
123.66
120.50
120.77
118.77
117.79
77.40
77.19
76.98
22.33
22.26
150MHz
CDCl3
Br
OH
O
40
210
200
190
180
170
160
150
140
130
120
110
100
90
80
70
60
50
40
30
20
10

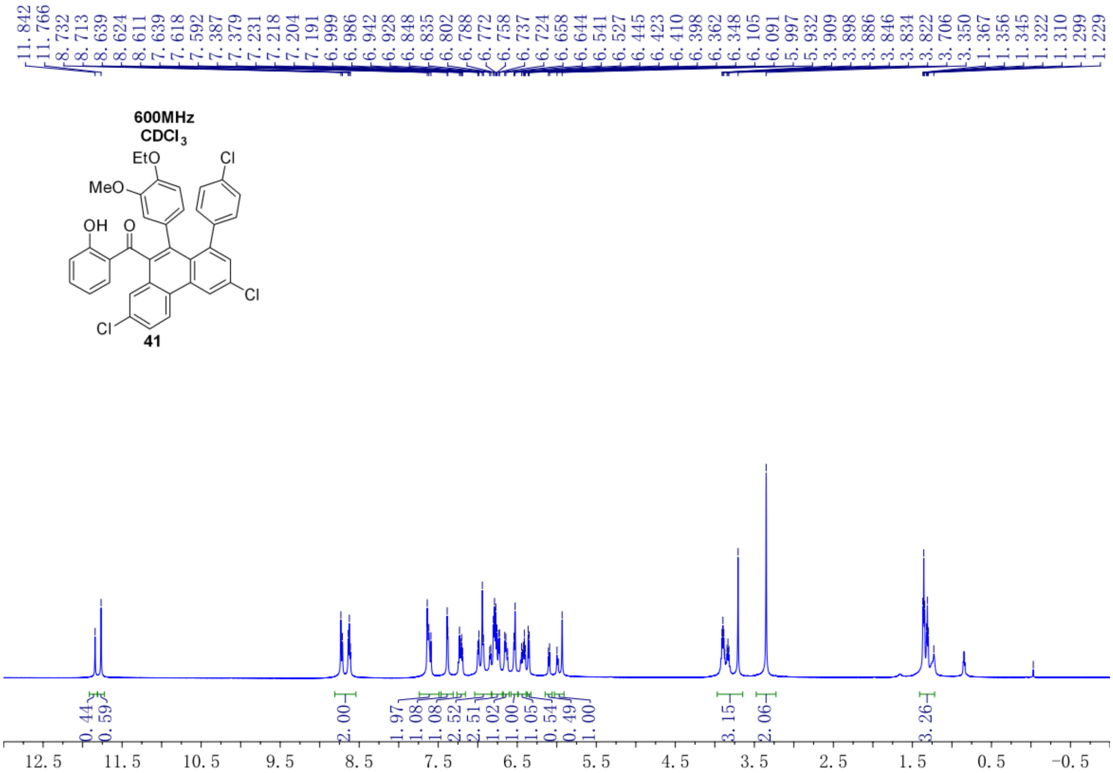
11.842 11.766 8.732 8.713 8.639 8.624 8.611 7.639 7.618 7.592 7.387 7.379 7.231 7.218 7.204 7.191 6.999 6.986 6.942 6.928 6.848 6.835 6.802 6.788 6.772 6.758 6.737 6.724 6.658 6.644 6.541 6.527 6.445 6.423 6.410 6.398 6.362 6.348 6.105 6.091 5.997 5.932 3.909 3.898 3.886 3.846 3.834 3.822 3.706 3.350 1.367 1.356 1.345 1.322 1.310 1.299 1.229
600MHz
CDCl3
EtO
MeO
OH
O
Cl
Cl
Cl
Cl
41
0.44 0.59 2.00 1.97 1.08 1.08 2.52 2.51 1.02 1.00 1.05 0.54 0.49 1.00 3.15 2.06 3.26
12.5 11.5 10.5 9.5 8.5 7.5 6.5 5.5 4.5 3.5 2.5 1.5 0.5 -0.5

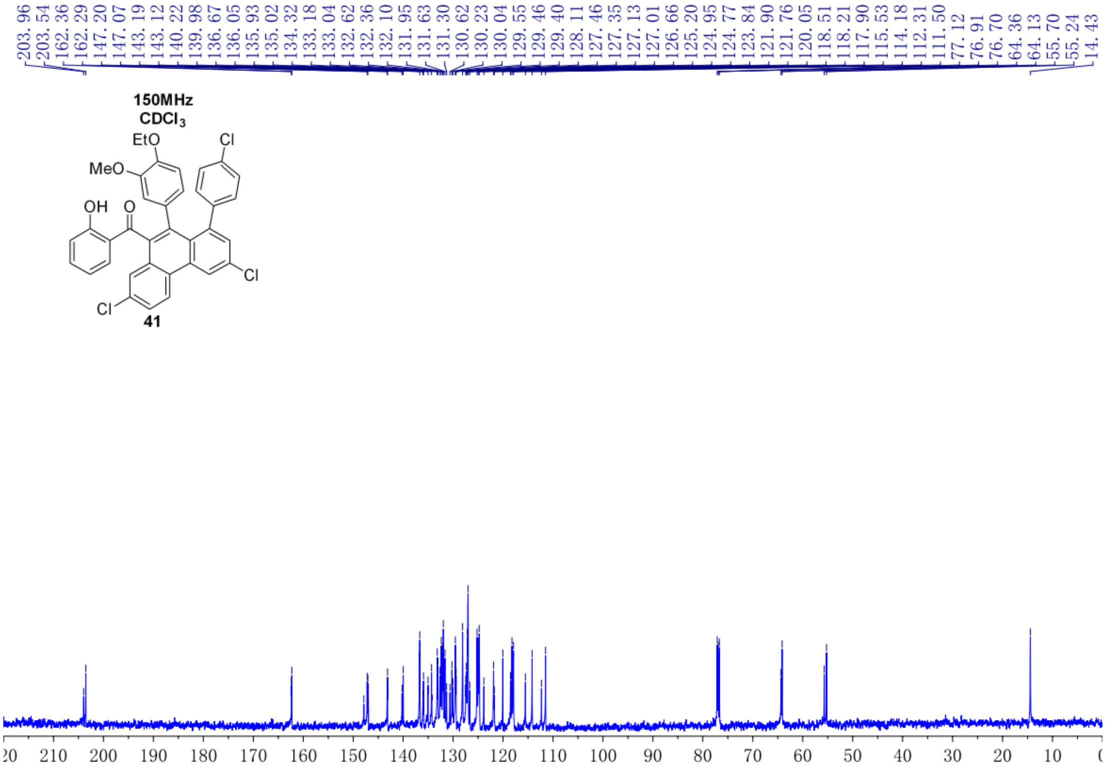
203.96 203.54 162.36 162.29 147.20 147.07 143.19 143.12 140.22 139.98 136.67 136.05 135.93 135.02 134.32 133.18 133.04 132.62 132.36 132.10 131.95 131.63 131.30 130.62 130.23 130.04 129.55 129.46 129.40 128.11 127.46 127.35 127.13 127.01 126.66 125.20 124.95 124.77 123.84 121.90 121.76 120.05 118.51 118.21 117.90 115.53 114.18 112.31 111.50 77.12 76.91 76.70 64.36 64.13 55.70 55.24 14.43
150MHz
CDCl3
EtO
MeO
OH
O
Cl
Cl
Cl
Cl
41
210 200 190 180 170 160 150 140 130 120 110 100 90 80 70 60 50 40 30 20 10

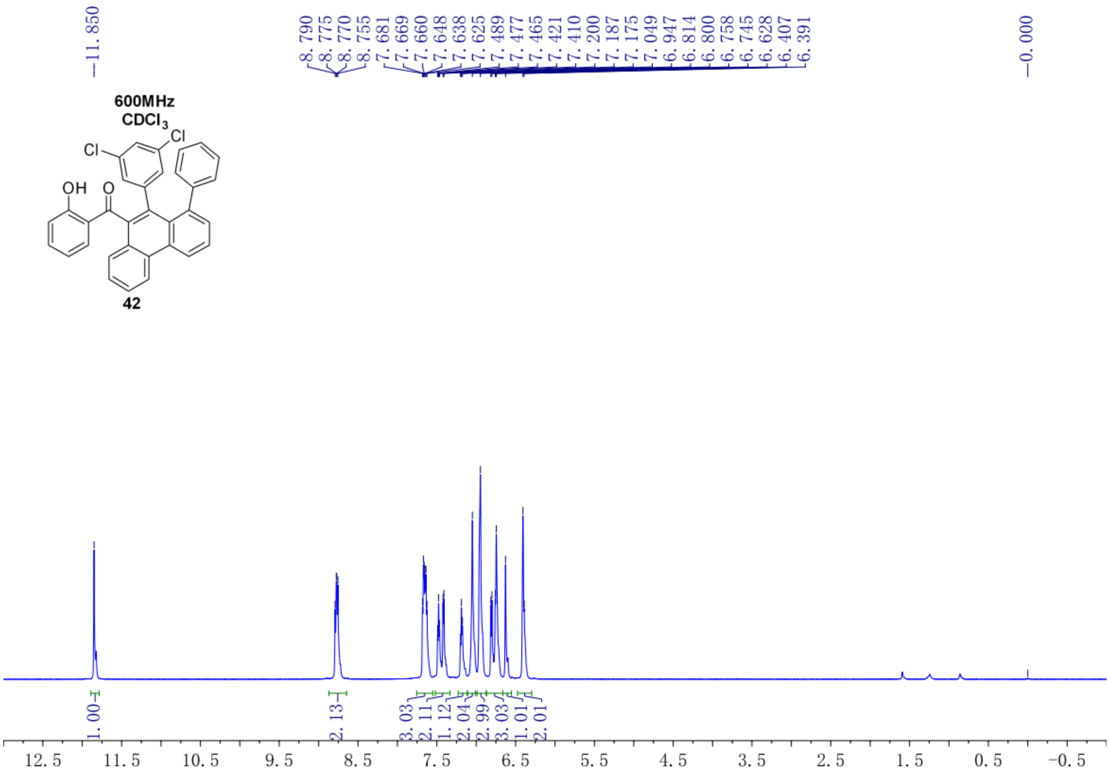

11.850
8.790
8.775
8.770
8.755
7.681
7.669
7.660
7.648
7.638
7.625
7.489
7.477
7.465
7.421
7.410
7.200
7.187
7.175
7.049
6.947
6.814
6.800
6.758
6.745
6.628
6.407
6.391
0.000
600MHz
CDCl3
Cl
Cl
OH
O
42
1.00
2.13
3.03
2.11
1.12
2.04
2.99
3.03
1.01
2.01
12.5
11.5
10.5
9.5
8.5
7.5
6.5
5.5
4.5
3.5
2.5
1.5
0.5
-0.5

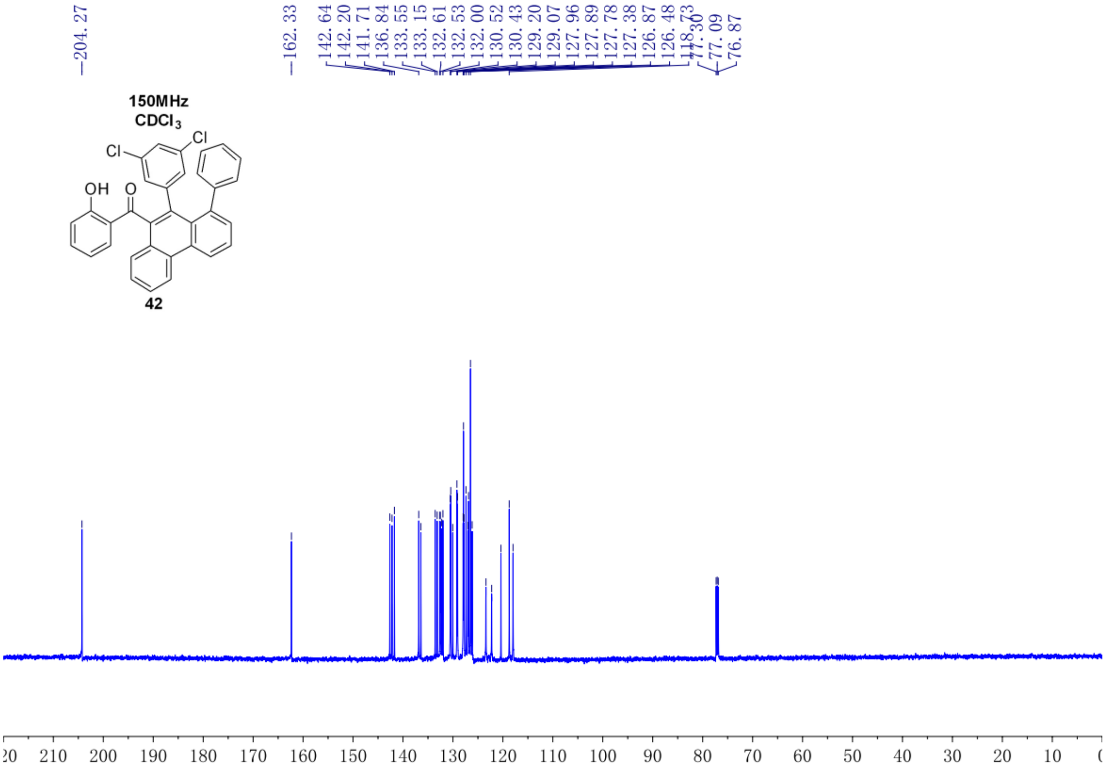

204.27
162.33
142.64
142.20
141.71
136.84
133.55
133.15
132.61
132.53
132.00
130.52
130.43
129.20
129.07
127.96
127.89
127.78
127.38
126.87
126.48
118.73
77.30
77.09
76.87
150MHz
CDCl3
Cl
Cl
OH
O
42
210
200
190
180
170
160
150
140
130
120
110
100
90
80
70
60
50
40
30
20
10

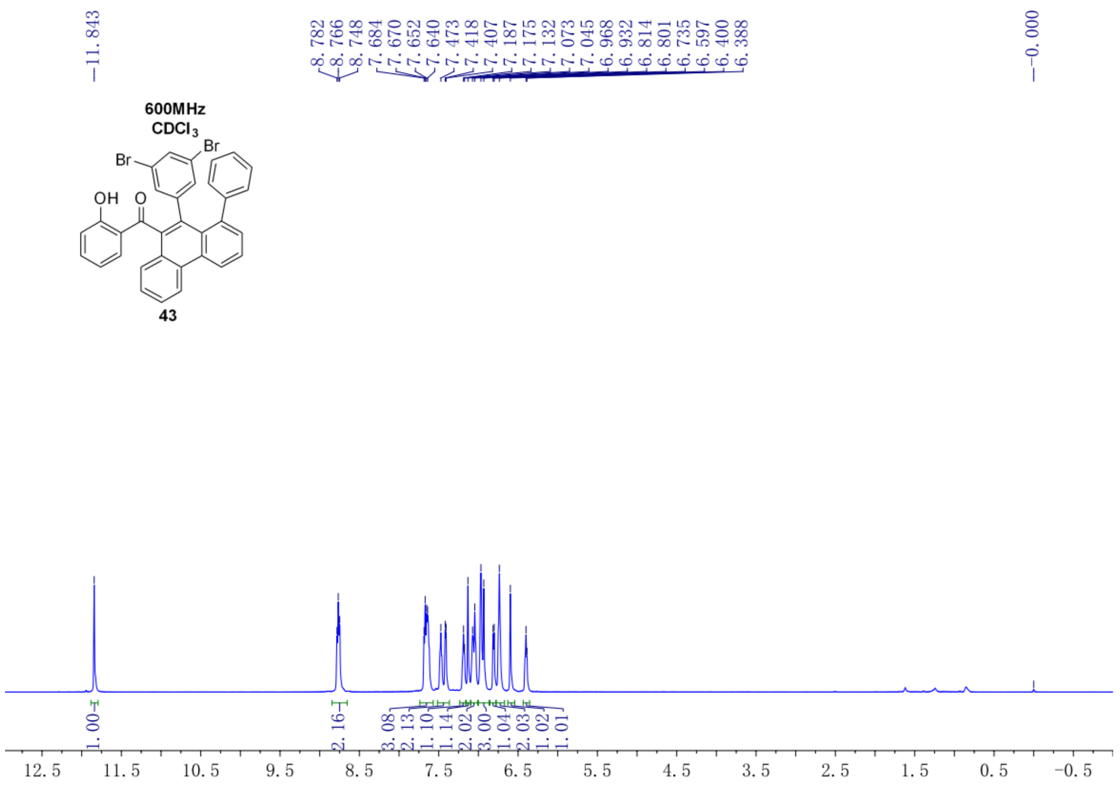

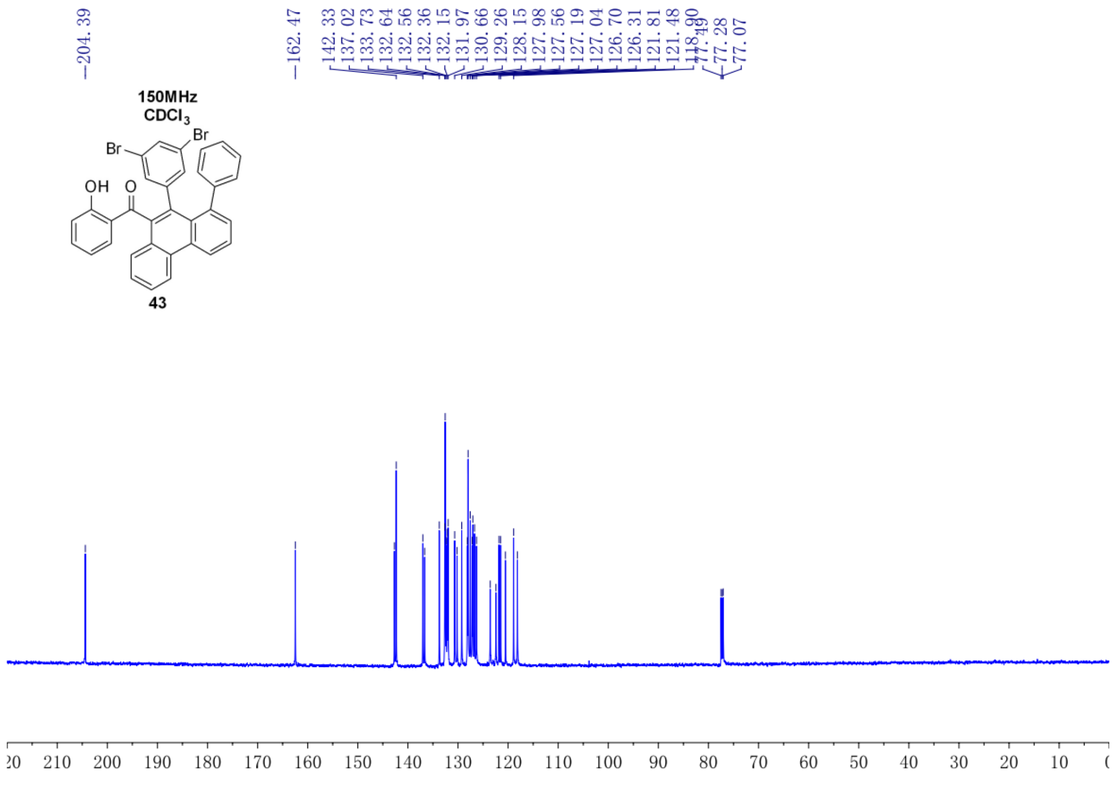

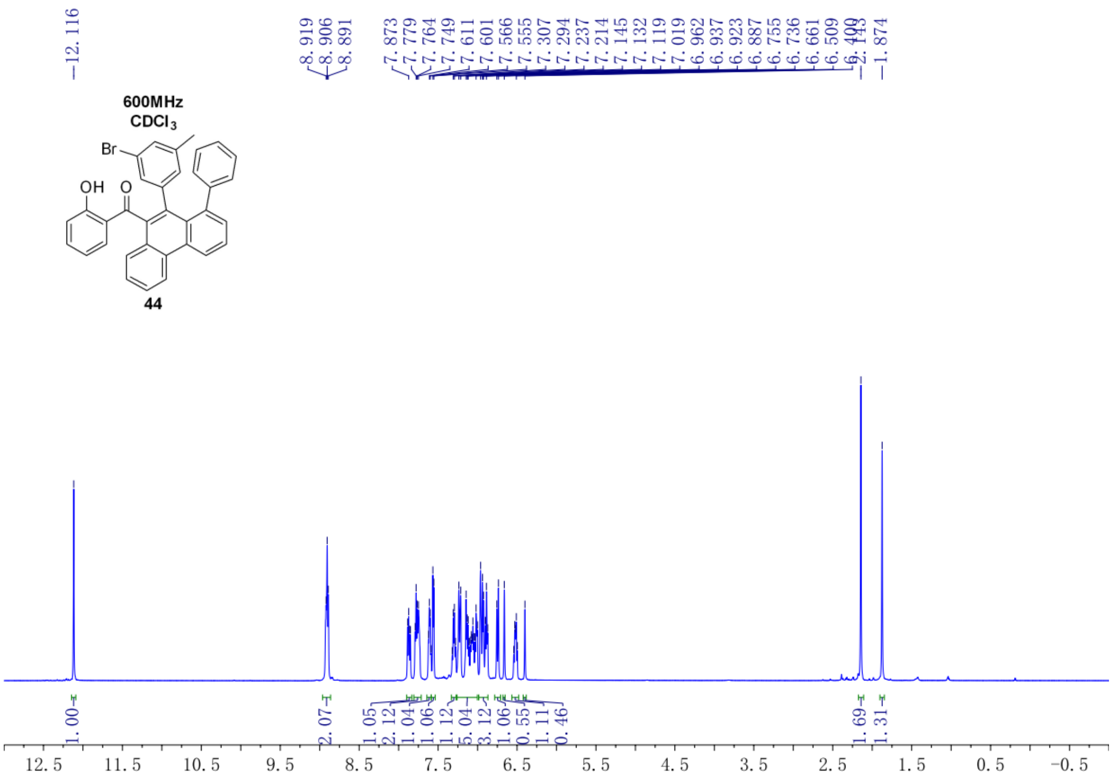
600MHz
CDCl3
Br
OH
O
44
12.116
8.919
8.906
8.891
7.873
7.779
7.764
7.749
7.611
7.601
7.566
7.555
7.307
7.294
7.237
7.214
7.145
7.132
7.119
7.019
6.962
6.937
6.923
6.887
6.755
6.736
6.661
6.509
2.143
1.874
1.00
2.07
1.05
2.12
1.04
1.06
1.12
5.04
3.12
1.06
0.55
1.11
0.46
1.69
1.31
12.5
11.5
10.5
9.5
8.5
7.5
6.5
5.5
4.5
3.5
2.5
1.5
0.5
-0.5

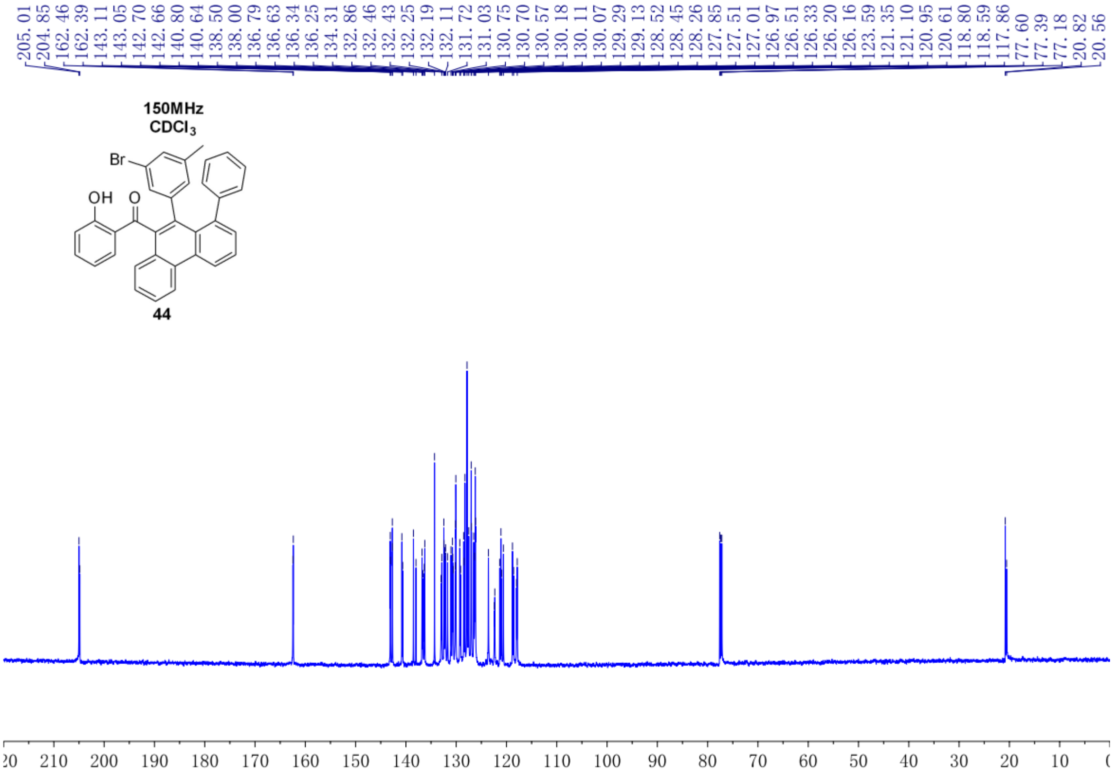
150MHz
CDCl3
Br
OH
O
44
205.01
204.85
162.46
162.39
143.11
143.05
142.70
142.66
140.80
140.64
138.50
138.00
136.79
136.63
136.34
136.25
134.31
132.86
132.46
132.43
132.25
132.19
132.11
131.72
131.03
130.75
130.70
130.57
130.18
130.11
130.07
129.29
129.13
128.52
128.45
128.26
127.85
127.51
127.01
126.97
126.51
126.33
126.20
126.16
123.59
121.35
121.10
120.95
120.61
118.80
118.59
117.86
77.60
77.39
77.18
20.82
20.56
210
200
190
180
170
160
150
140
130
120
110
100
90
80
70
60
50
40
30
20
10

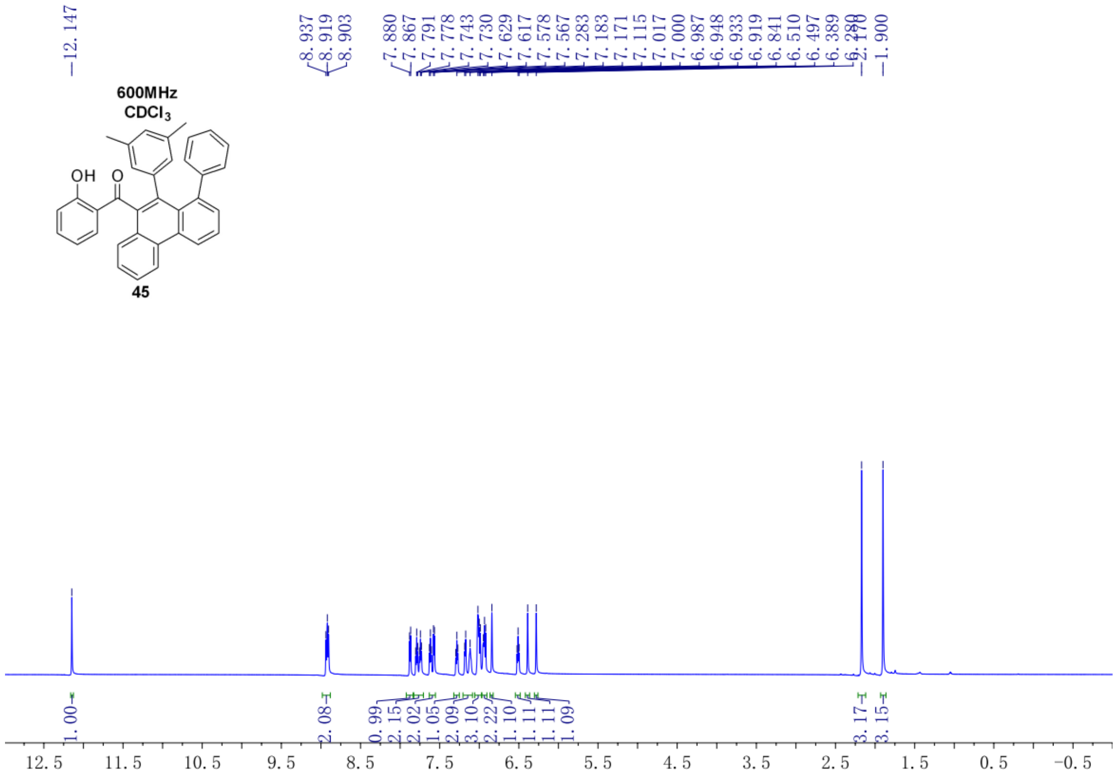
12.147
8.937
8.919
8.903
7.880
7.867
7.791
7.778
7.743
7.730
7.629
7.617
7.578
7.567
7.283
7.183
7.171
7.115
7.017
7.000
6.987
6.948
6.933
6.919
6.841
6.510
6.497
6.389
1.900
600MHz
CDCl3
OH
O
45
1.00
2.08
0.99
2.15
2.02
1.05
2.09
3.10
2.22
1.10
1.11
1.11
1.09
3.17
3.15
12.5
11.5
10.5
9.5
8.5
7.5
6.5
5.5
4.5
3.5
2.5
1.5
0.5
-0.5

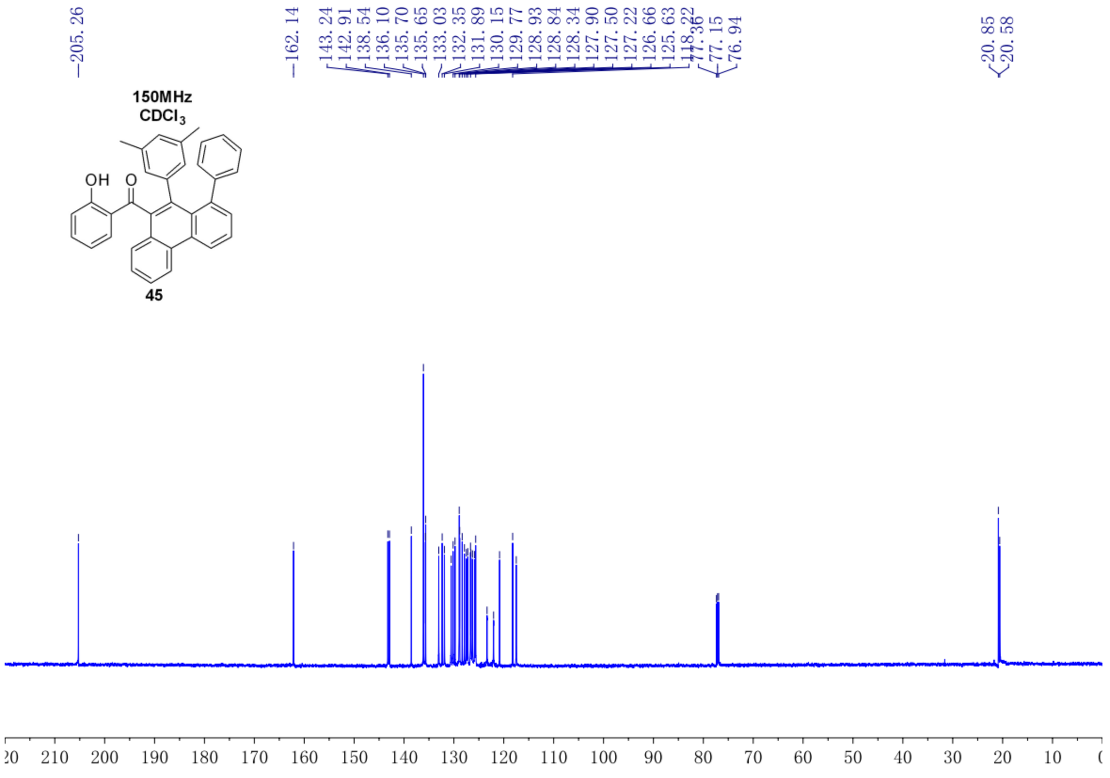
205.26
162.14
143.24
142.91
138.54
136.10
135.70
135.65
133.03
132.35
131.89
130.15
129.77
128.93
128.84
128.34
127.90
127.50
127.22
126.66
125.63
118.22
77.15
76.94
20.85
20.58
150MHz
CDCl3
OH
O
45
210
200
190
180
170
160
150
140
130
120
110
100
90
80
70
60
50
40
30
20
10

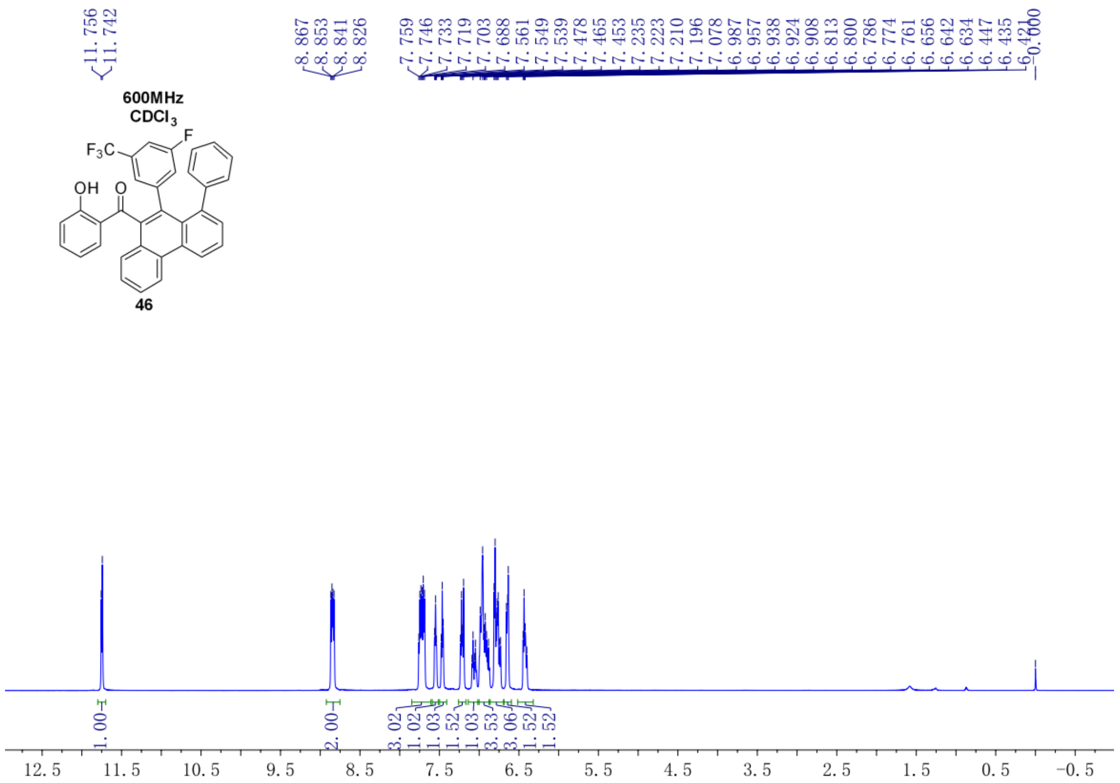
11.756
11.742
8.867
8.853
8.841
8.826
7.759
7.746
7.733
7.719
7.703
7.688
7.561
7.549
7.539
7.478
7.465
7.453
7.235
7.223
7.210
7.196
7.078
6.987
6.957
6.938
6.924
6.908
6.813
6.800
6.786
6.774
6.761
6.656
6.642
6.634
6.447
6.435
6.421
0.000
600MHz
$CDCl_3$
F_3C
F
OH
O
46
1.00
2.00
3.02
1.02
1.03
1.52
1.03
3.53
3.06
1.52
1.52
12.5 11.5 10.5 9.5 8.5 7.5 6.5 5.5 4.5 3.5 2.5 1.5 0.5 -0.5

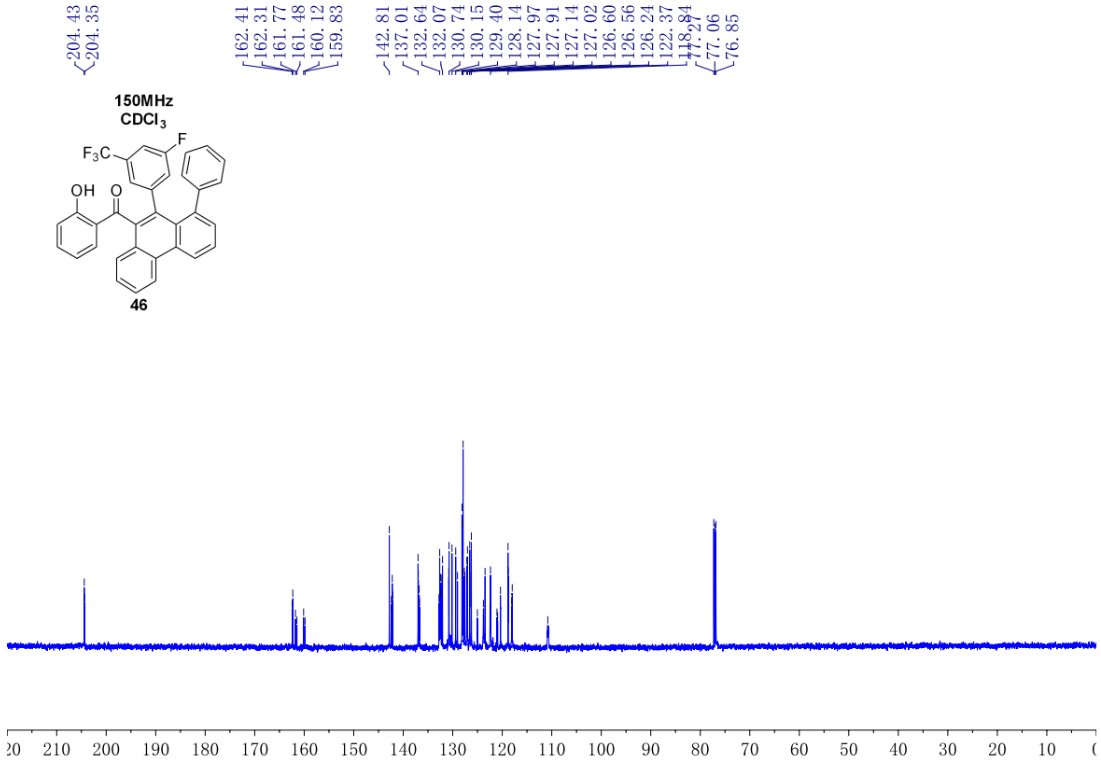
204.43
204.35
162.41
162.31
161.77
161.48
160.12
159.83
142.81
137.01
132.64
132.07
130.74
130.15
129.40
128.14
127.97
127.91
127.14
127.02
126.60
126.56
126.24
122.37
118.84
77.27
77.06
76.85
150MHz
$CDCl_3$
F_3C
F
OH
O
46
210 200 190 180 170 160 150 140 130 120 110 100 90 80 70 60 50 40 30 20 10

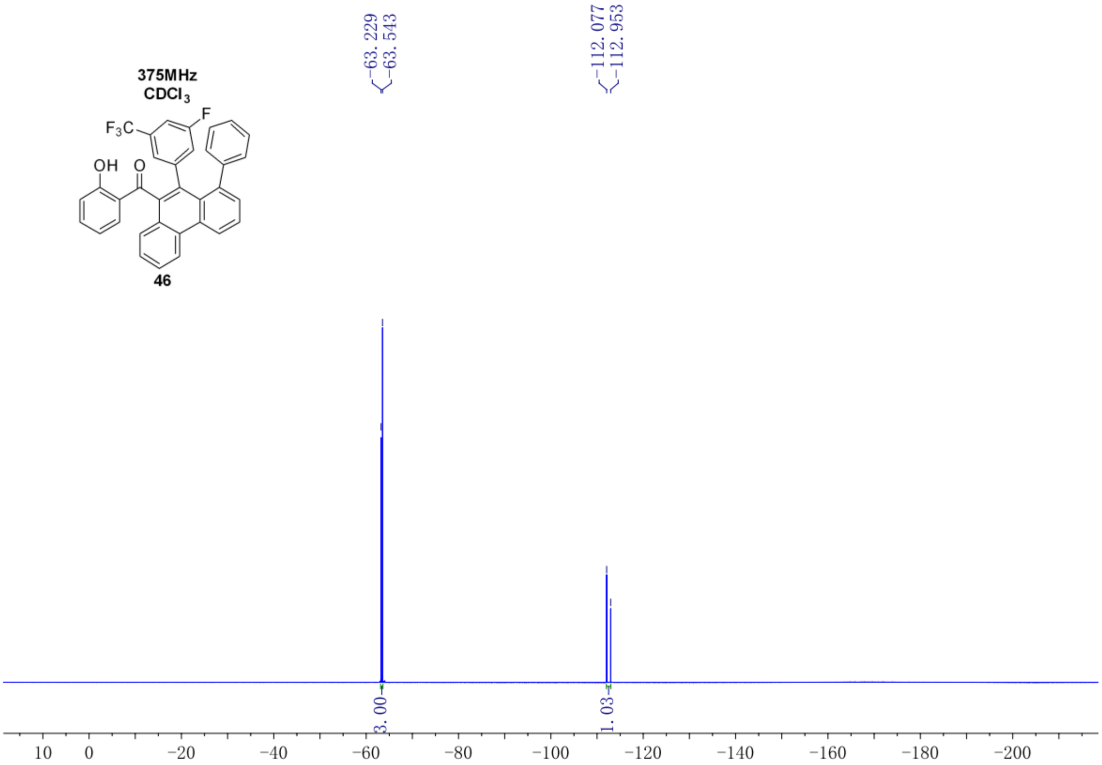

375MHz
$CDCl_3$
-63.229
-63.543
-112.077
-112.953
3.00
1.03
10 0 −20 −40 −60 −80 −100 −120 −140 −160 −180 −200

46

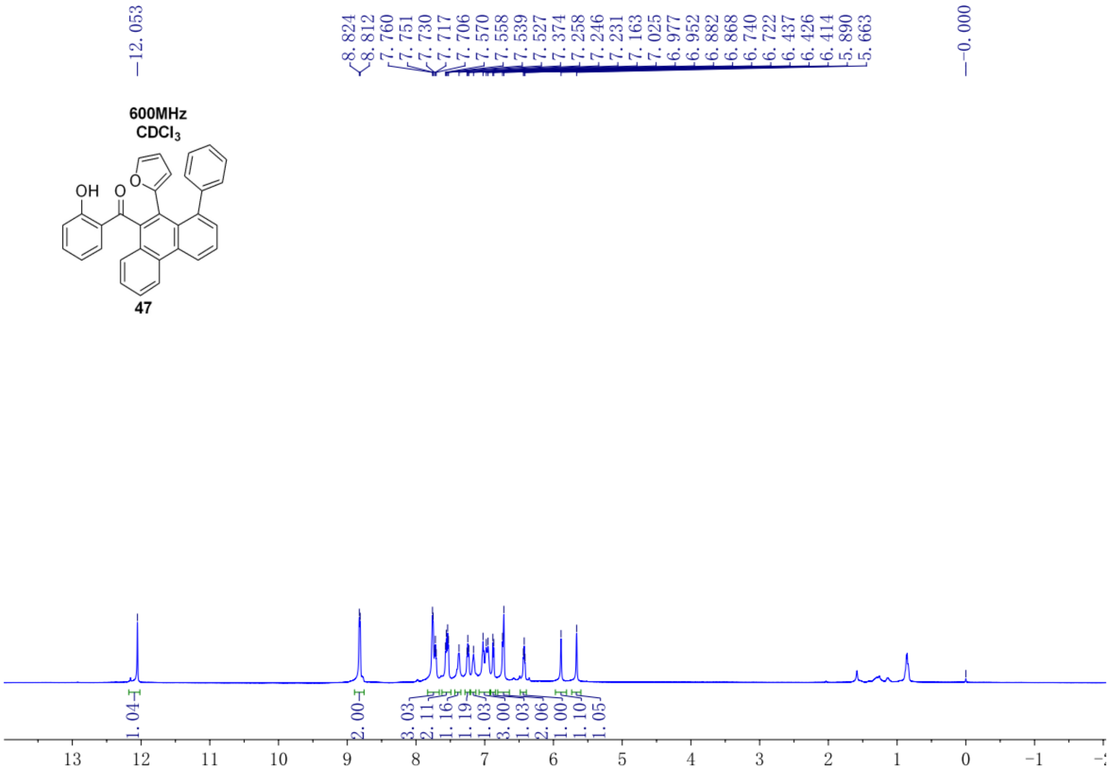

600MHz
$CDCl_3$
12.053
8.824
8.812
7.760
7.751
7.730
7.717
7.706
7.570
7.558
7.539
7.527
7.374
7.258
7.246
7.231
7.163
7.025
6.977
6.952
6.882
6.868
6.740
6.722
6.437
6.426
6.414
5.890
5.663
0.000
1.04
2.00
3.03
2.11
1.16
1.19
1.03
3.00
1.03
2.06
1.00
1.10
1.05
13 12 11 10 9 8 7 6 5 4 3 2 1 0 −1

47

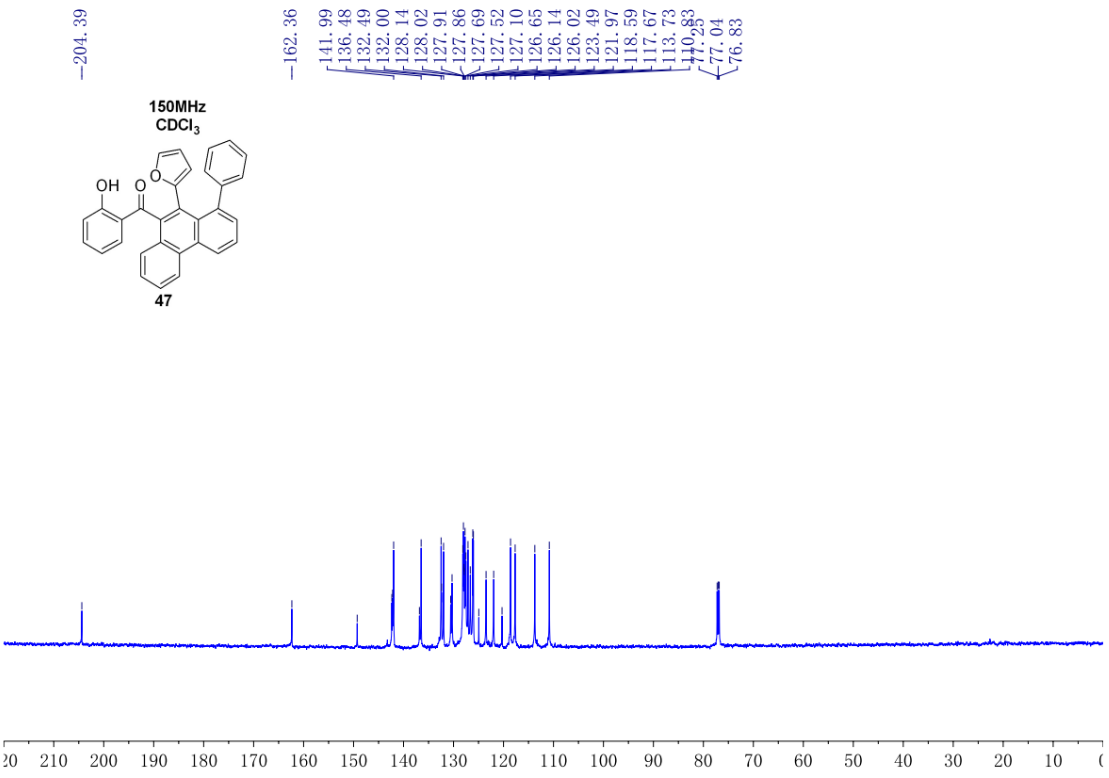
204.39
162.36
141.99
136.48
132.49
132.00
128.14
128.02
127.91
127.86
127.69
127.52
127.10
126.65
126.14
126.02
123.49
121.97
118.59
117.67
113.73
110.83
77.25
77.04
76.83
150MHz
CDCl3
OH
O
47
20
210
200
190
180
170
160
150
140
130
120
110
100
90
80
70
60
50
40
30
20
10

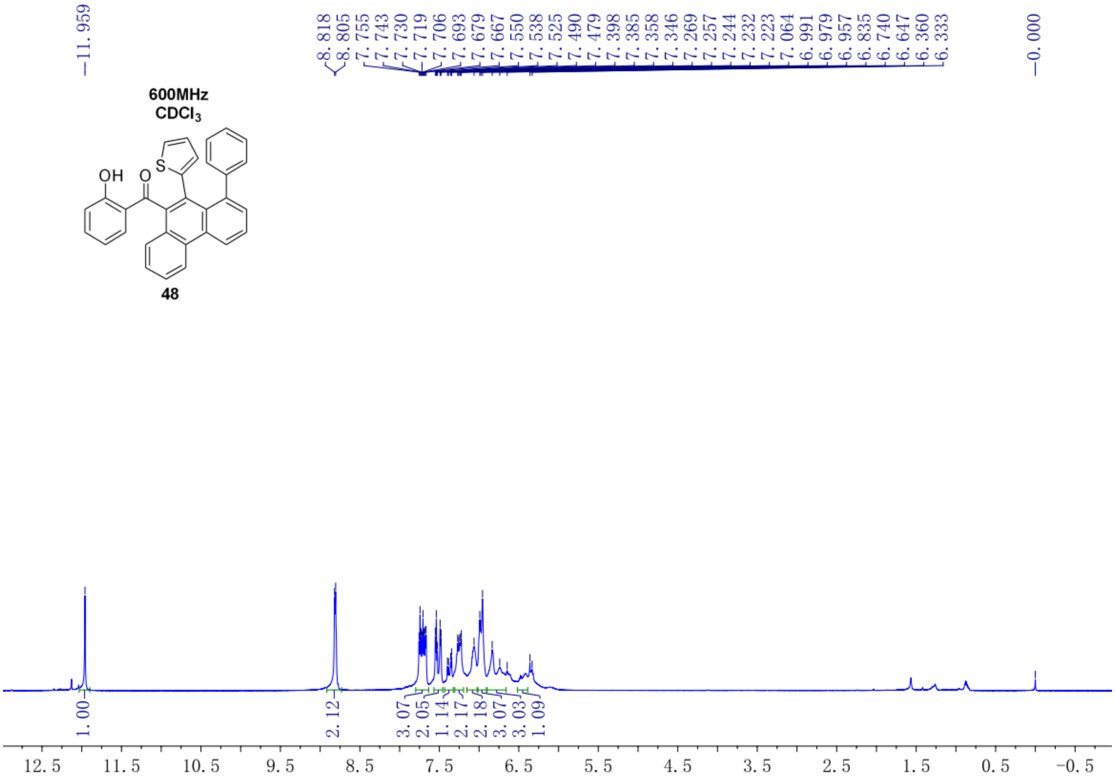
11.959
8.818
8.805
7.755
7.743
7.730
7.719
7.706
7.693
7.679
7.667
7.550
7.538
7.525
7.490
7.479
7.398
7.385
7.358
7.346
7.269
7.257
7.244
7.232
7.223
7.064
6.991
6.979
6.957
6.835
6.740
6.647
6.360
6.333
0.000
600MHz
CDCl3
OH
O
S
48
1.00
2.12
3.07
2.05
1.14
2.17
2.18
3.07
3.03
1.09
12.5
11.5
10.5
9.5
8.5
7.5
6.5
5.5
4.5
3.5
2.5
1.5
0.5
-0.5

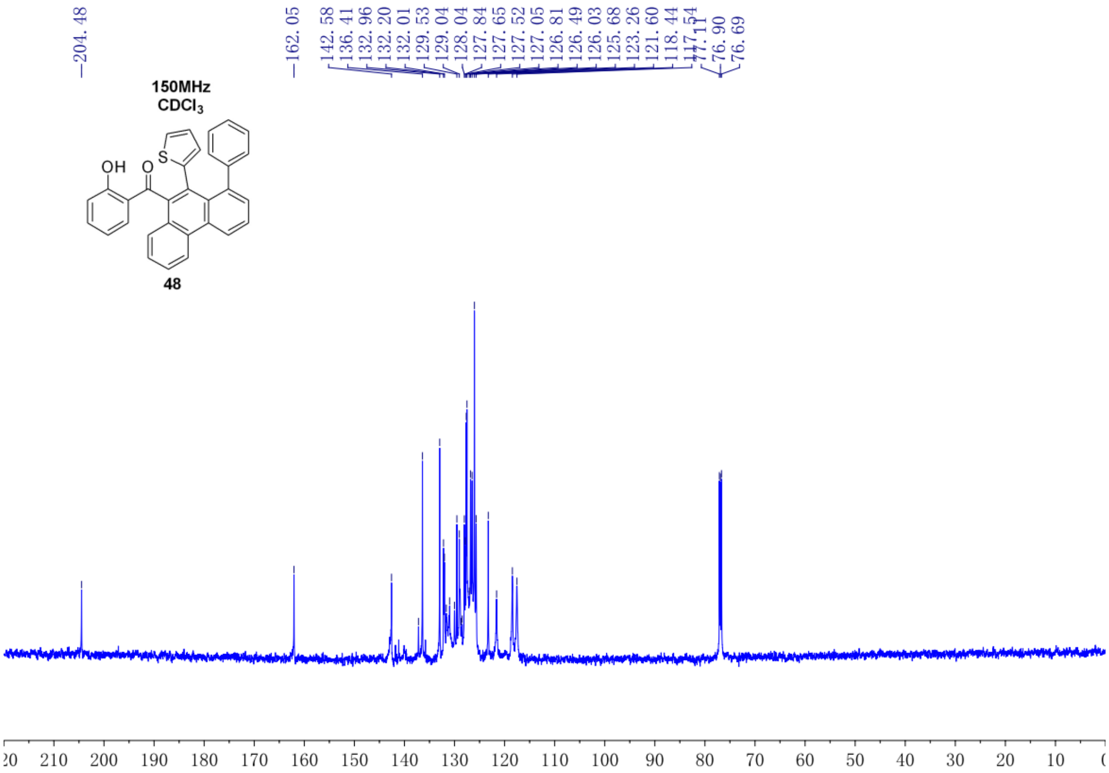
204.48
162.05
142.58
136.41
132.96
132.20
132.01
129.53
129.04
128.04
127.84
127.65
127.52
127.05
126.81
126.49
126.03
125.68
123.26
121.60
118.44
117.54
77.11
76.90
76.69
150MHz
$CDCl_3$
OH
O
S
48
20 210 200 190 180 170 160 150 140 130 120 110 100 90 80 70 60 50 40 30 20 10

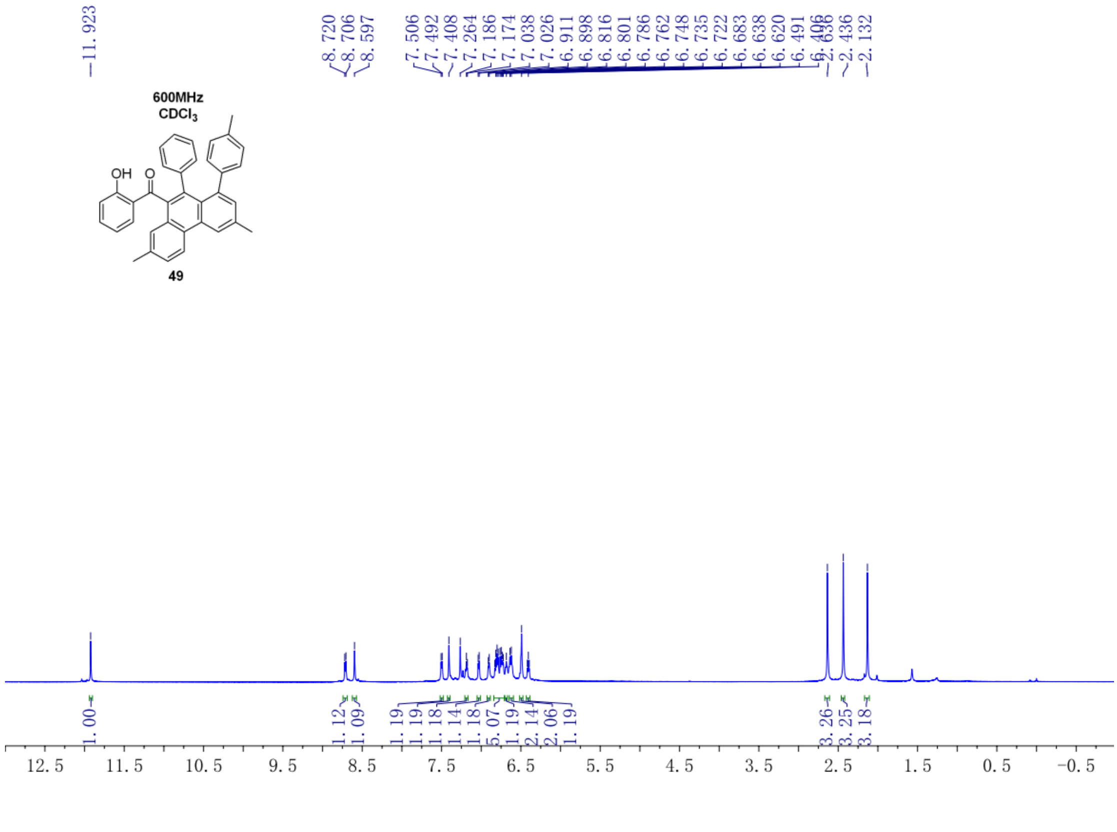
11.923
8.720
8.706
8.597
7.506
7.492
7.408
7.264
7.186
7.174
7.038
7.026
6.911
6.898
6.816
6.801
6.786
6.762
6.748
6.735
6.722
6.683
6.638
6.620
6.491
6.496
2.636
2.436
2.132
600MHz
$CDCl_3$
OH
O
49
1.00
1.12
1.09
1.19
1.19
1.18
1.14
1.18
5.07
1.19
2.14
2.06
1.19
3.26
3.25
3.18
12.5 11.5 10.5 9.5 8.5 7.5 6.5 5.5 4.5 3.5 2.5 1.5 0.5 -0.5

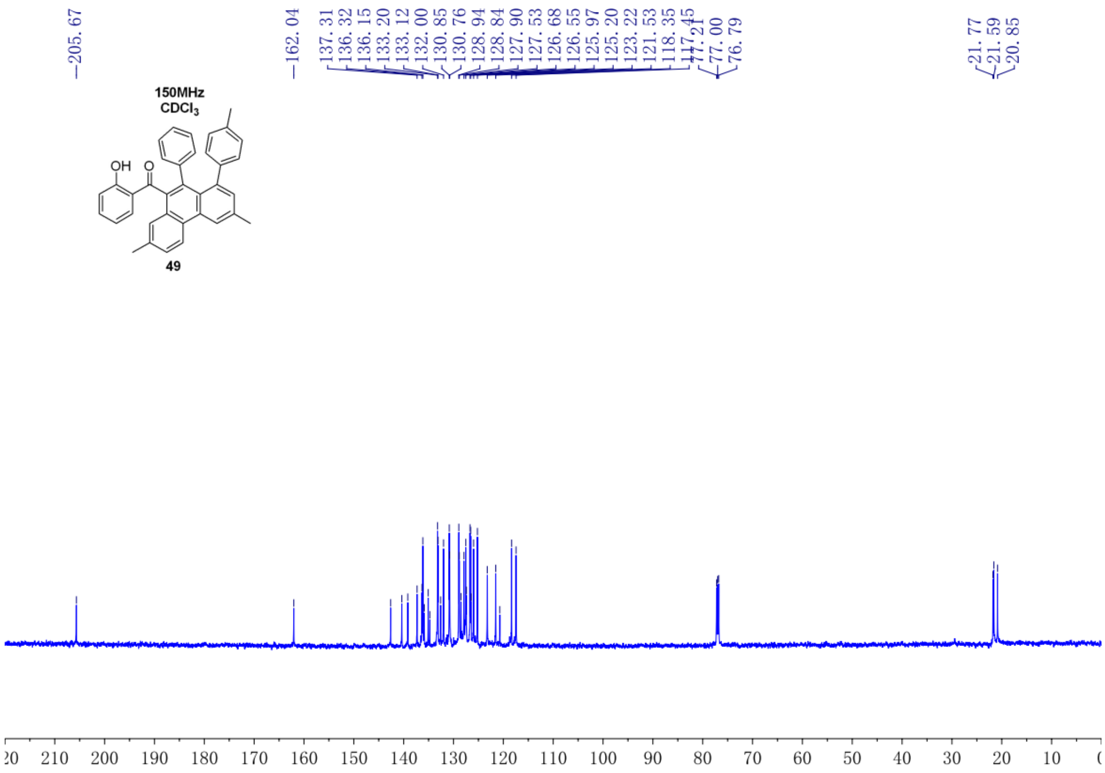
205.67
162.04
137.31
136.32
136.15
133.20
133.12
132.00
130.85
130.76
128.94
128.84
127.90
127.53
126.68
126.55
125.97
125.20
123.22
121.53
118.35
117.45
77.21
77.00
76.79
21.77
21.59
20.85
150MHz
CDCl3
OH
O
49
210 200 190 180 170 160 150 140 130 120 110 100 90 80 70 60 50 40 30 20 10

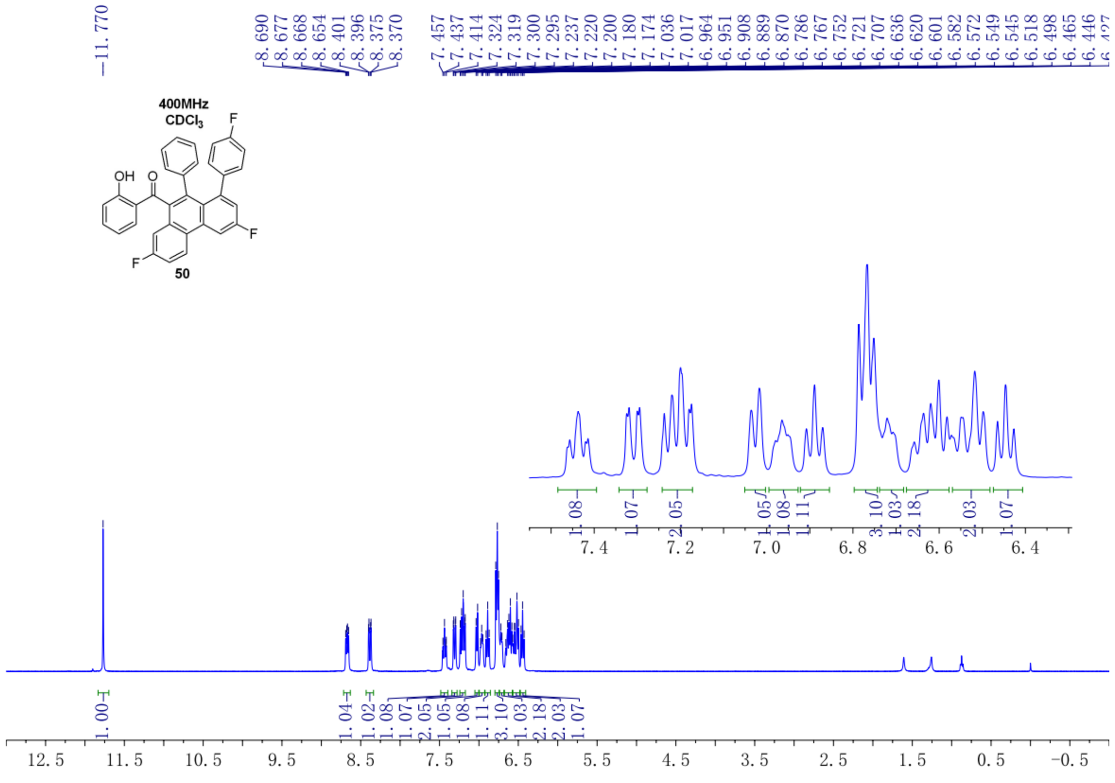
11.770
8.690
8.677
8.668
8.654
8.401
8.396
8.375
8.370
7.457
7.437
7.414
7.324
7.319
7.300
7.295
7.237
7.220
7.200
7.180
7.174
7.036
7.017
6.964
6.951
6.908
6.889
6.870
6.786
6.767
6.752
6.721
6.707
6.636
6.620
6.601
6.582
6.572
6.549
6.545
6.518
6.498
6.465
6.446
400MHz
CDCl3
F
OH
O
F
F
50
1.08 1.07 2.05 1.05 1.08 1.11 3.10 1.03 2.18 2.03 1.07
7.4 7.2 7.0 6.8 6.6 6.4
1.00 1.04 1.02 1.08 1.07 2.05 1.05 1.08 1.11 3.10 1.03 2.18 2.03 1.07
12.5 11.5 10.5 9.5 8.5 7.5 6.5 5.5 4.5 3.5 2.5 1.5 0.5 −0.5

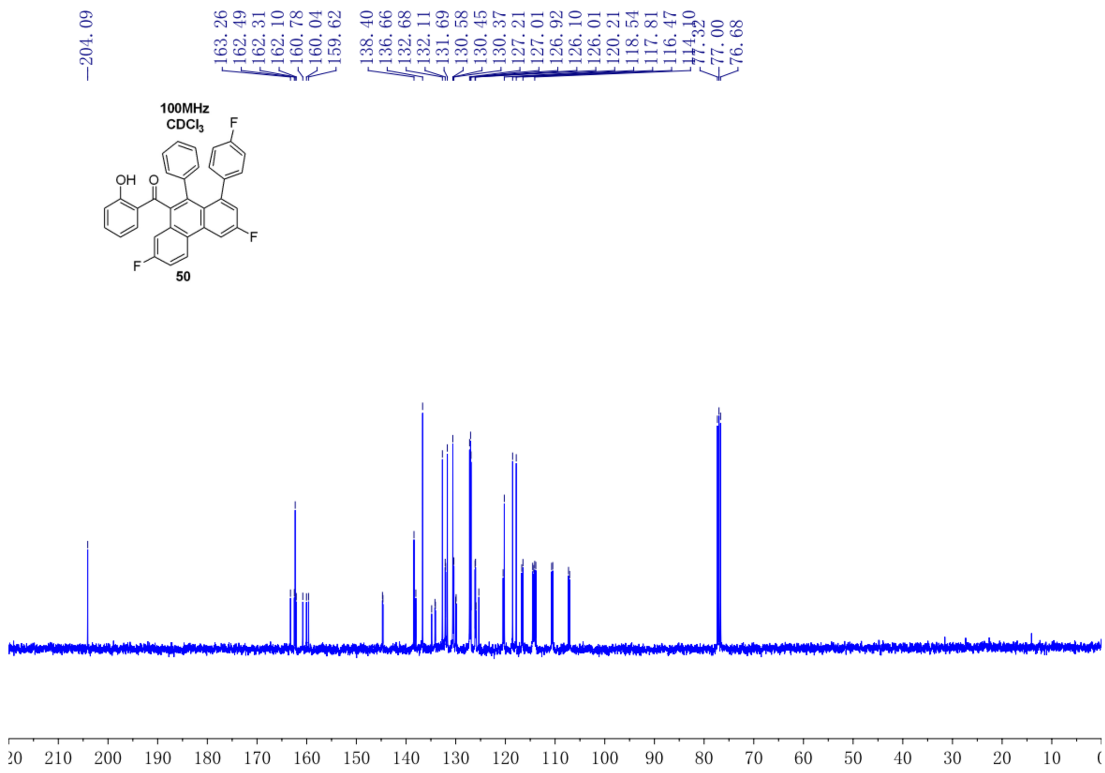

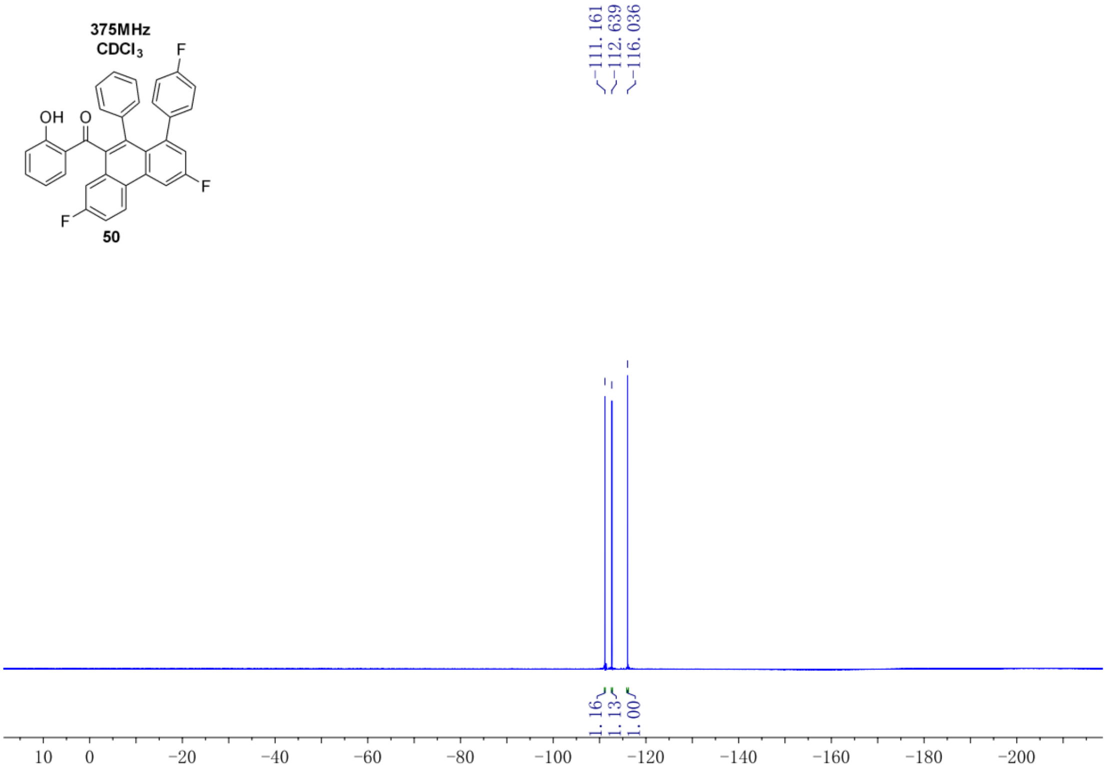

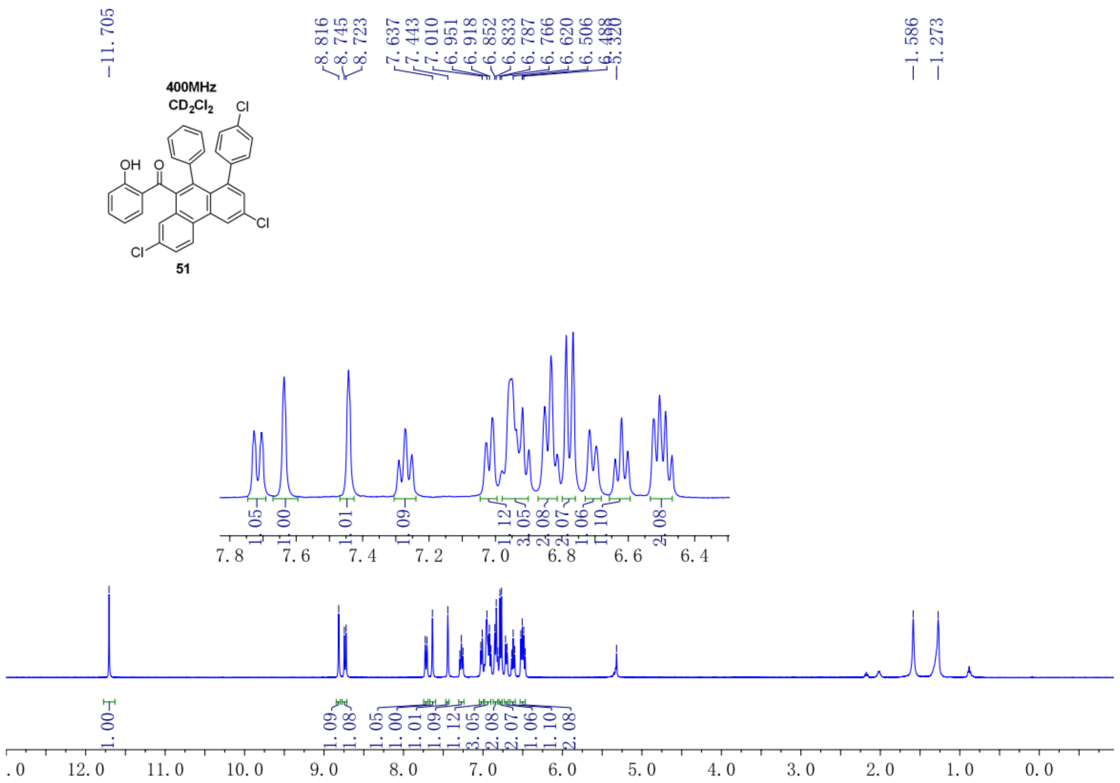

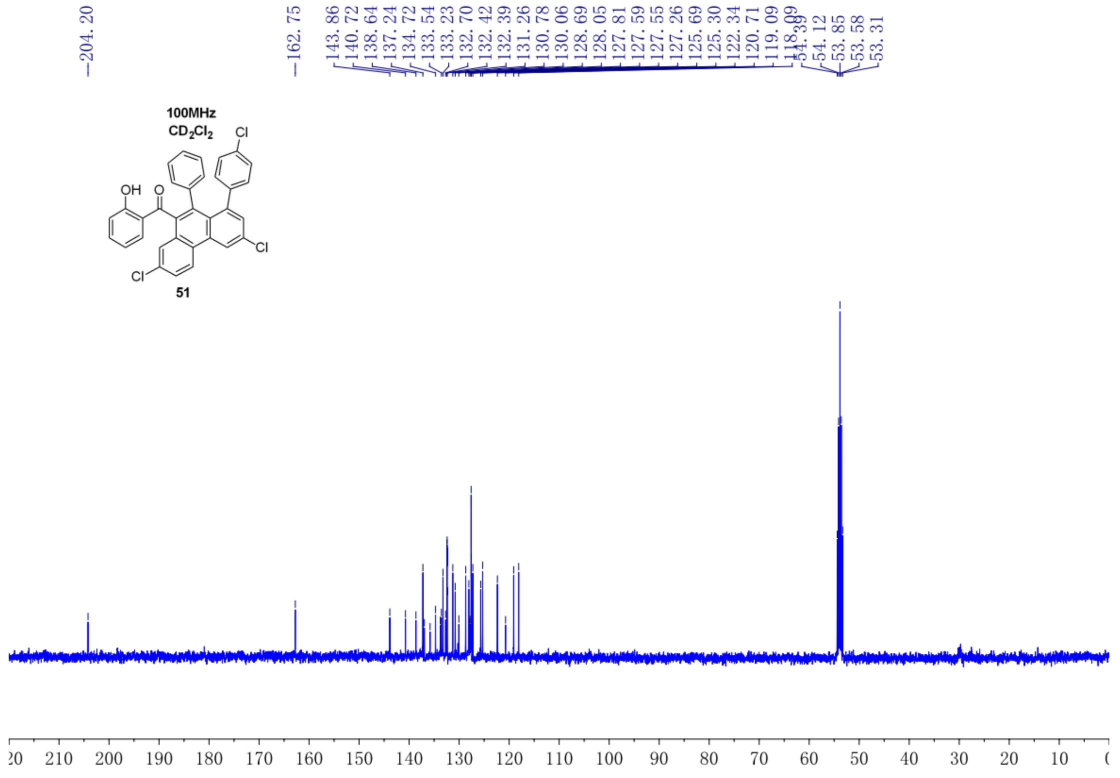

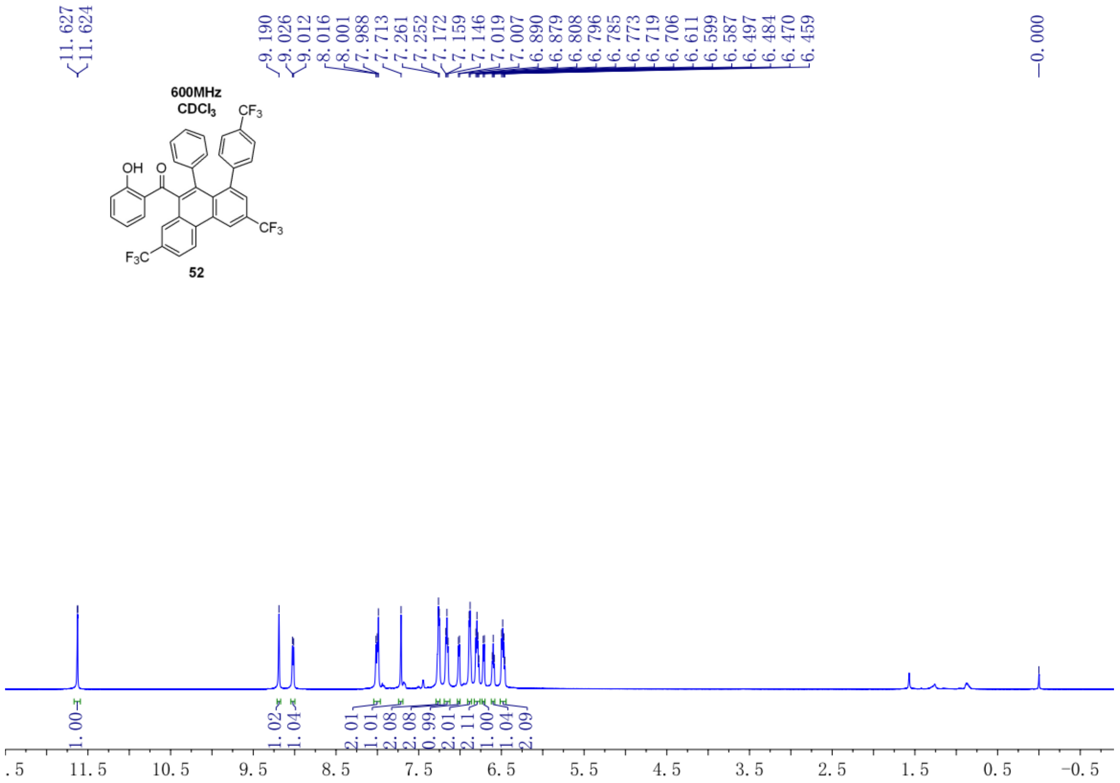

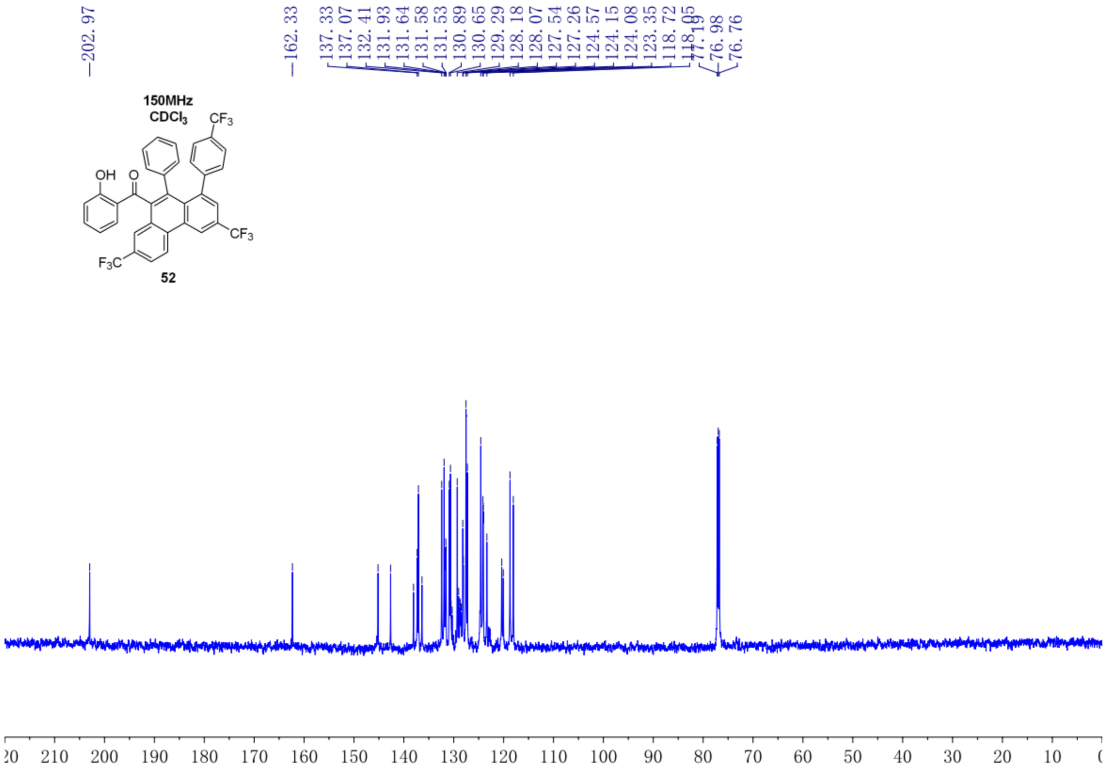

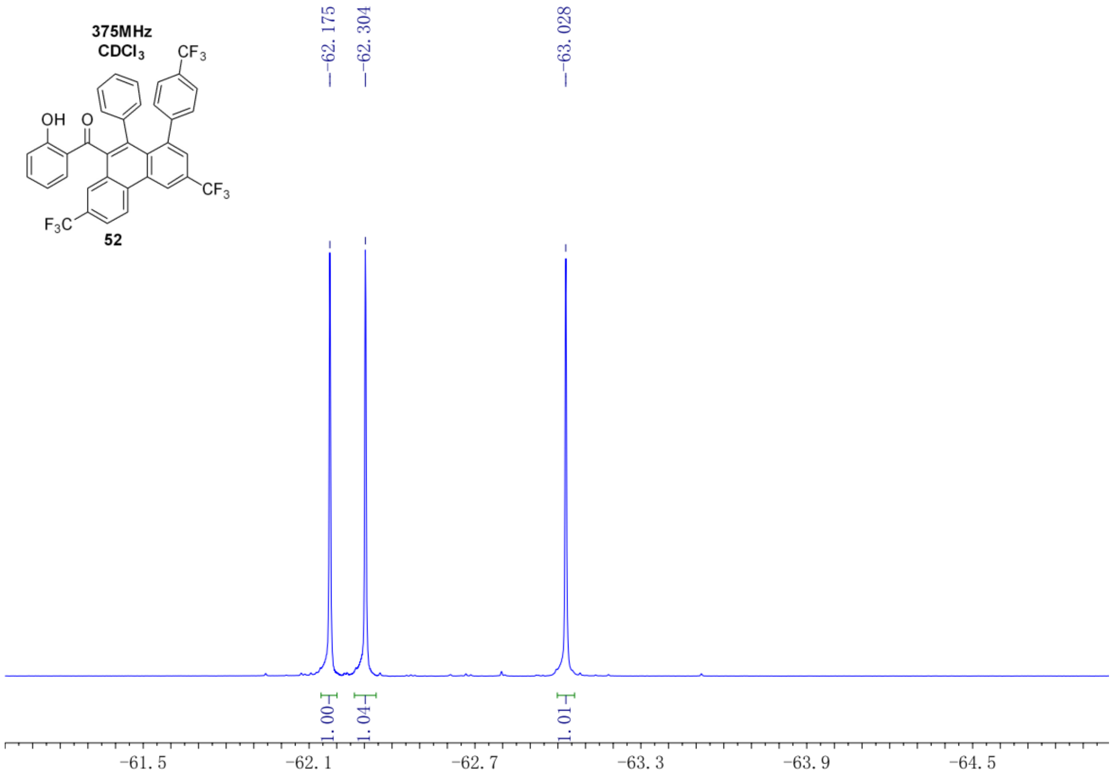
375MHz
CDCl3
52
-62.175
-62.304
-63.028
1.00
1.04
1.01
-61.5
-62.1
-62.7
-63.3
-63.9
-64.5

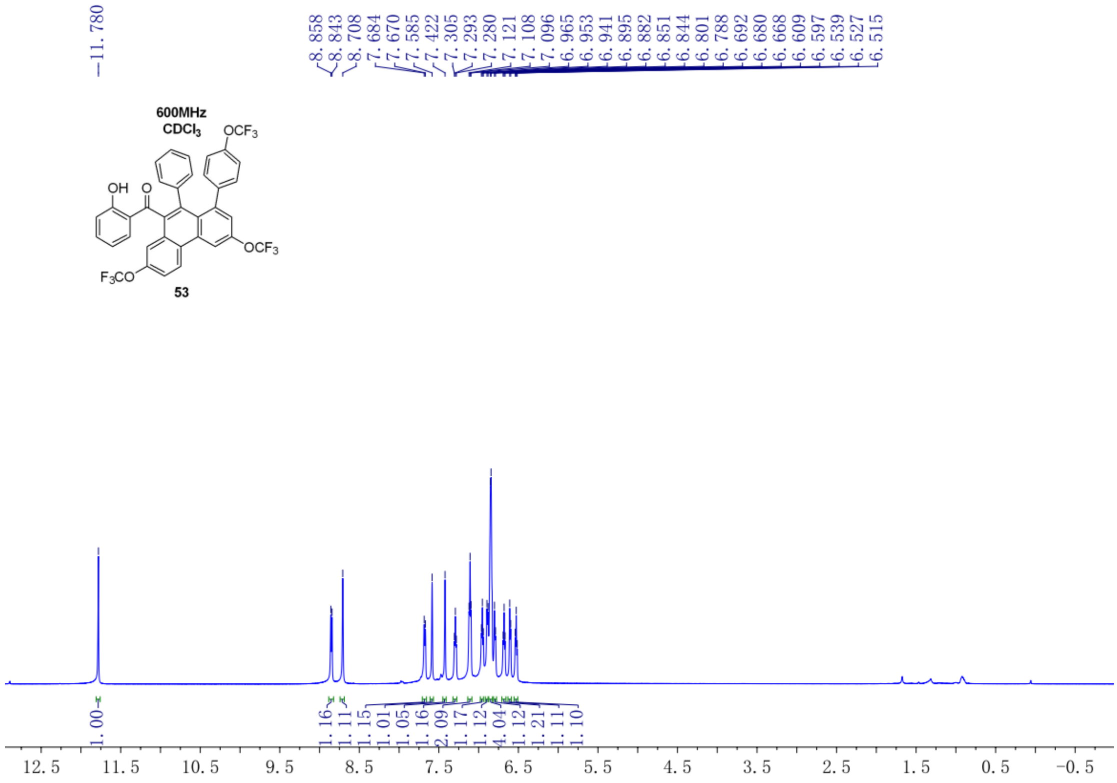
600MHz
CDCl3
53
11.780
8.858
8.843
8.708
7.684
7.670
7.585
7.422
7.305
7.293
7.280
7.121
7.108
7.096
6.965
6.953
6.941
6.895
6.882
6.851
6.844
6.801
6.788
6.692
6.680
6.668
6.609
6.597
6.539
6.527
6.515
1.00
1.16
1.11
1.15
1.01
1.05
1.16
2.09
1.17
1.12
4.04
1.12
1.21
1.11
1.10
12.5
11.5
10.5
9.5
8.5
7.5
6.5
5.5
4.5
3.5
2.5
1.5
0.5
-0.5

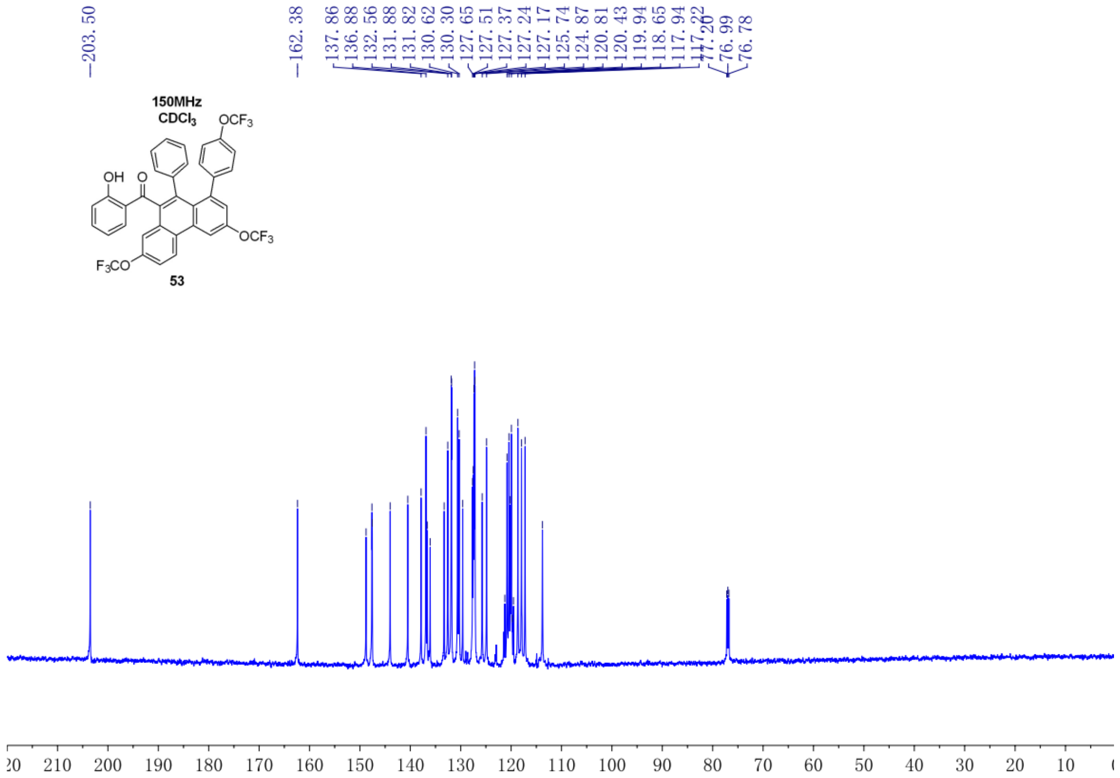

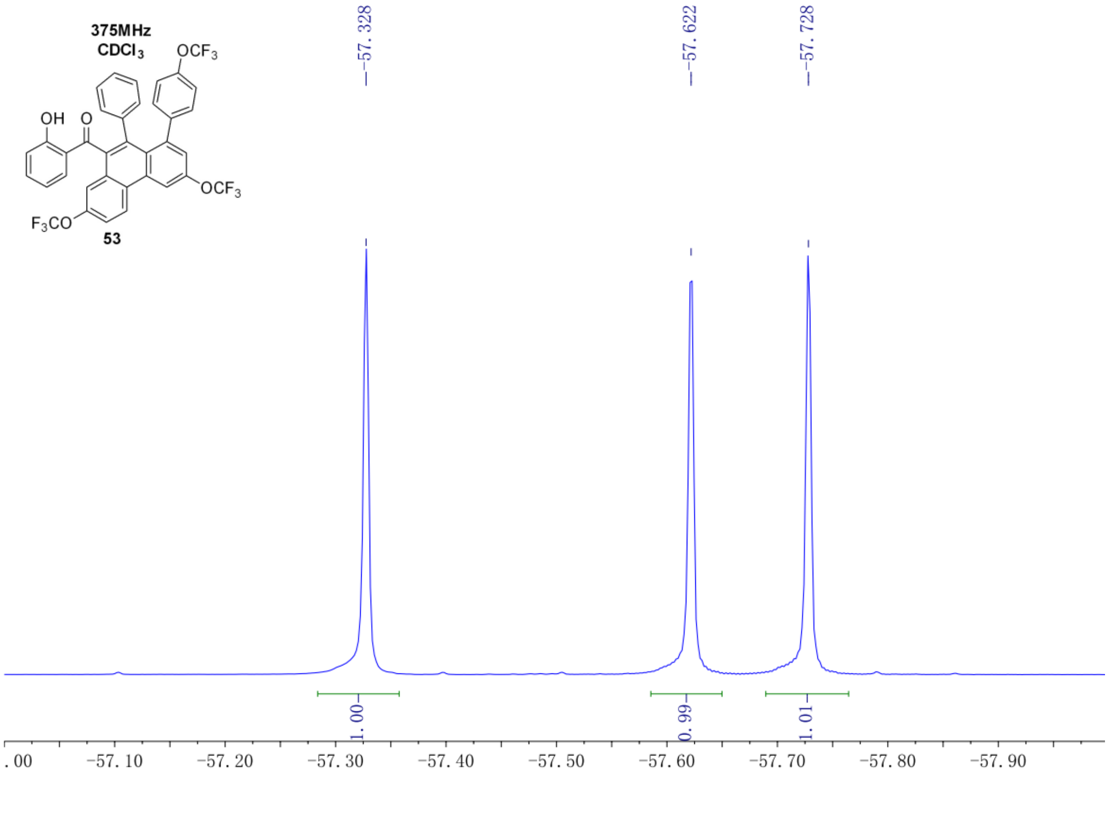

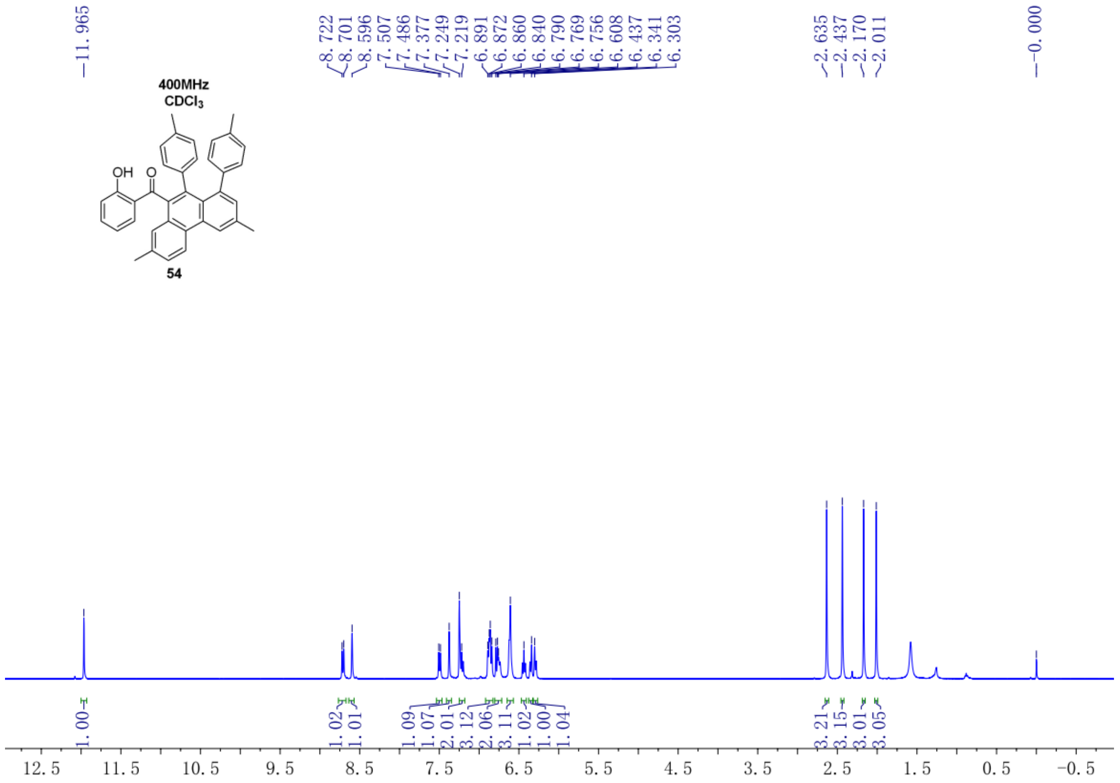
400MHz
CDCl3
OH
O
54
11.965
8.722
8.701
8.596
7.507
7.486
7.377
7.249
7.219
6.891
6.872
6.860
6.840
6.790
6.769
6.756
6.608
6.437
6.341
6.303
2.635
2.437
2.170
2.011
0.000
1.00
1.02
1.01
1.09
1.07
2.01
3.12
2.06
3.11
1.02
1.00
1.04
3.21
3.15
3.01
3.05
12.5
11.5
10.5
9.5
8.5
7.5
6.5
5.5
4.5
3.5
2.5
1.5
0.5
-0.5

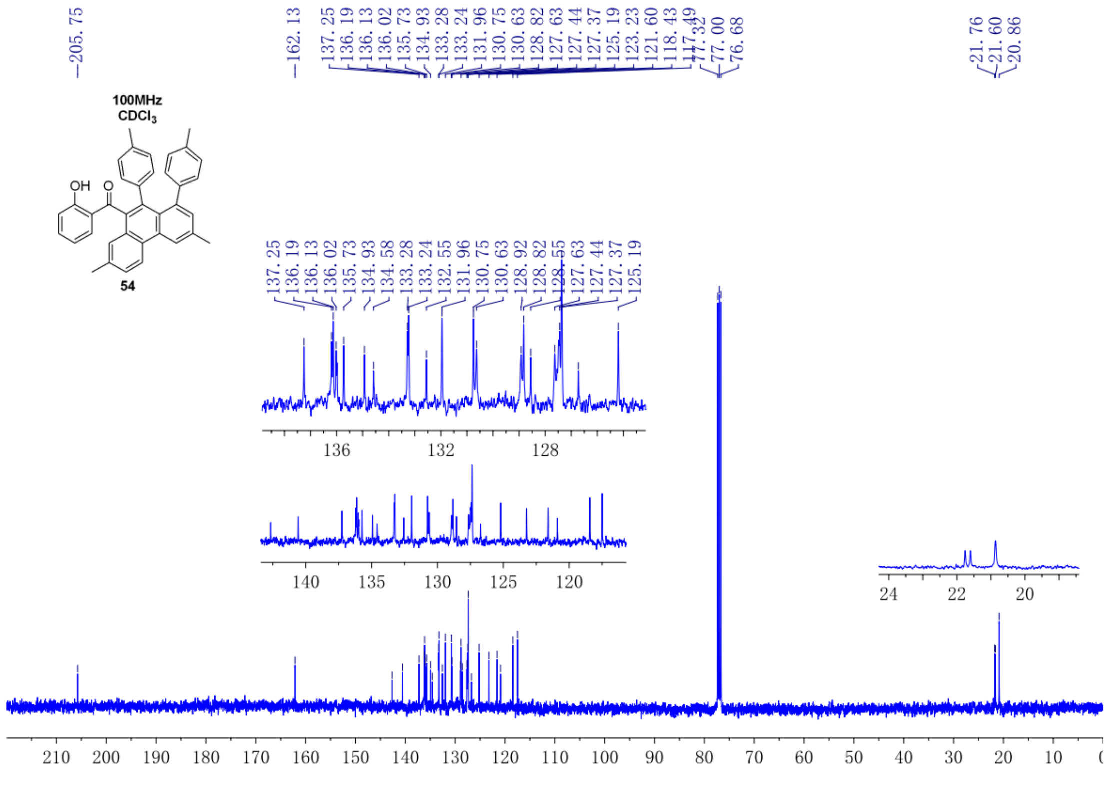
100MHz
CDCl3
OH
O
54
205.75
162.13
137.25
136.19
136.13
136.02
135.73
134.93
133.28
133.24
131.96
130.75
130.63
128.92
128.82
127.63
127.44
127.37
125.19
123.23
121.60
118.43
117.49
77.32
77.00
76.68
21.76
21.60
20.86
134.58
132.55
128.55
136
132
128
140
135
130
125
120
24
22
20
210
200
190
180
170
160
150
140
130
120
110
100
90
80
70
60
50
40
30
20
10

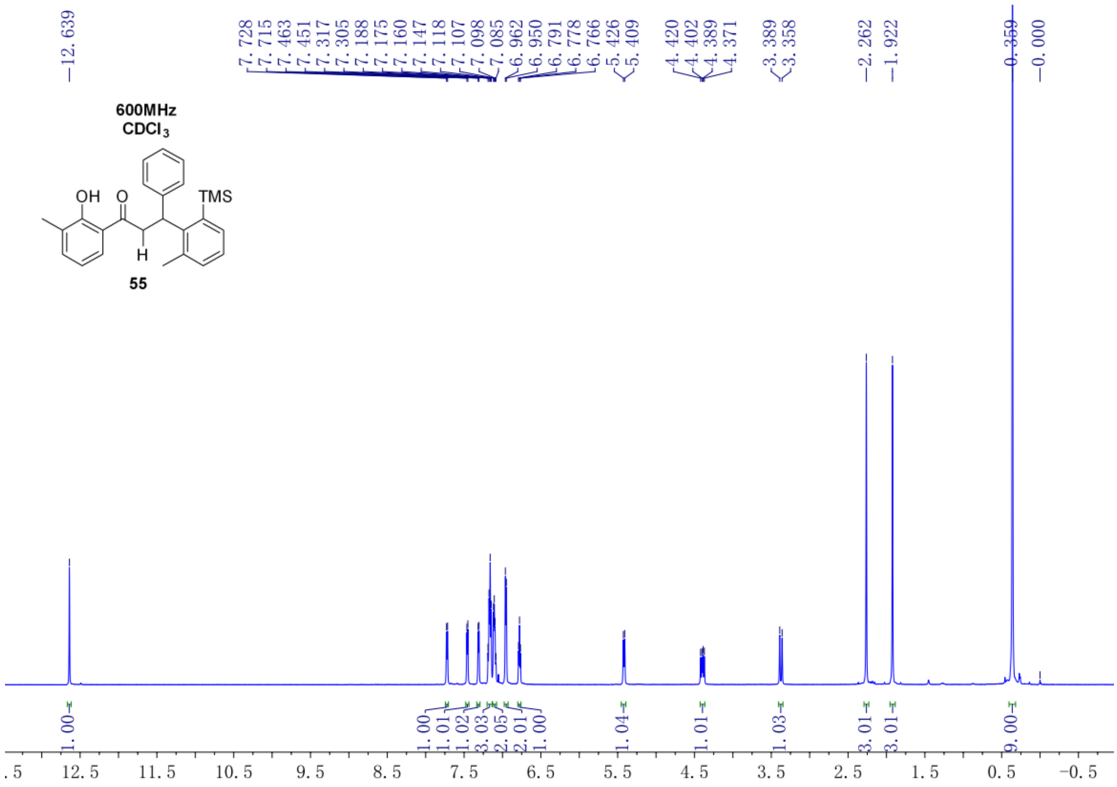
600MHz
CDCl3
OH
O
TMS
H
55

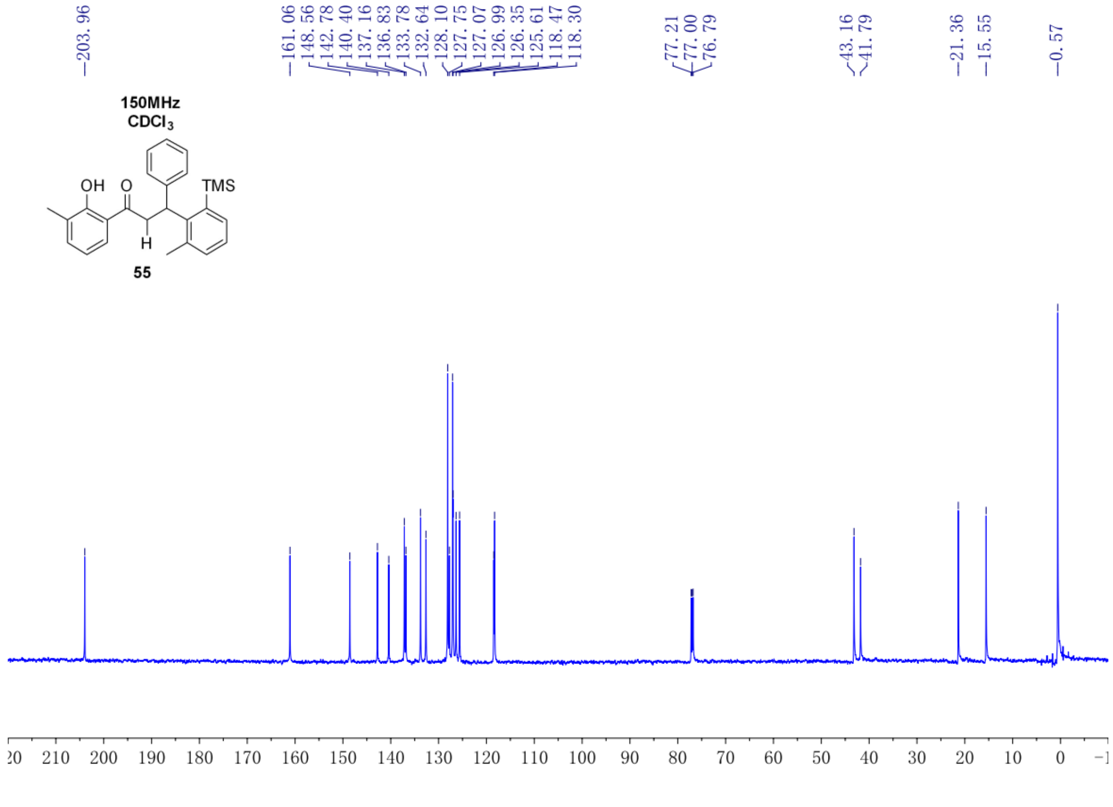
150MHz
CDCl3
OH
O
TMS
H
55

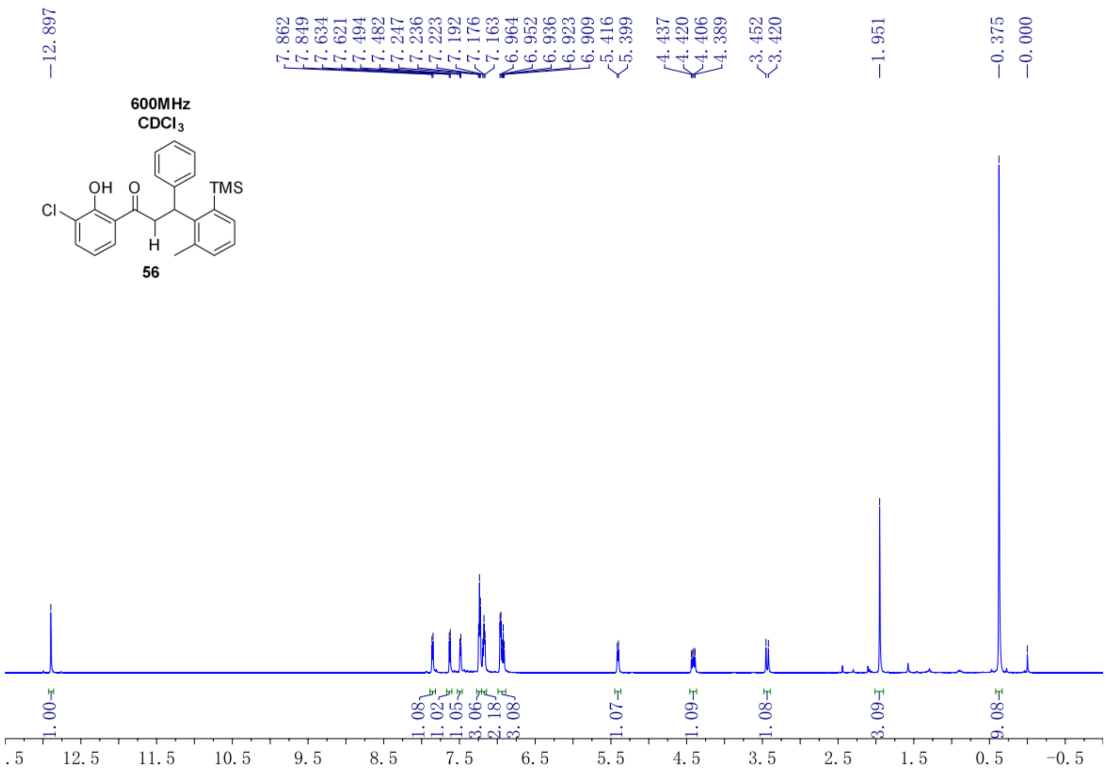

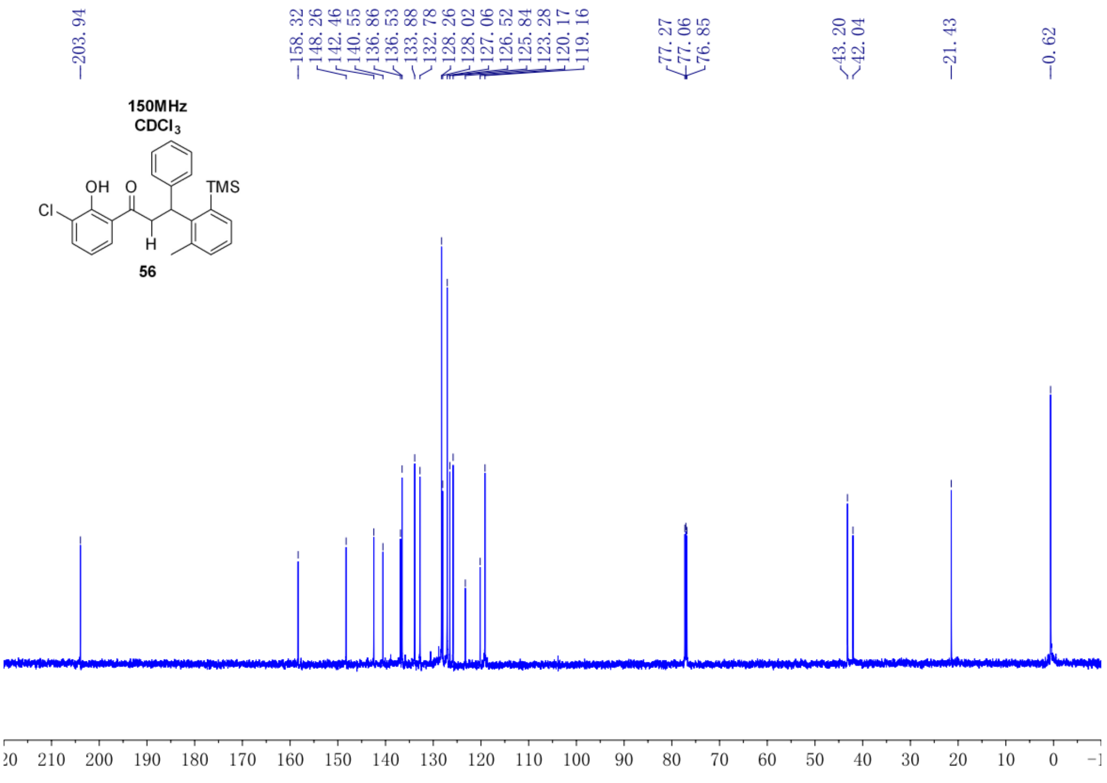

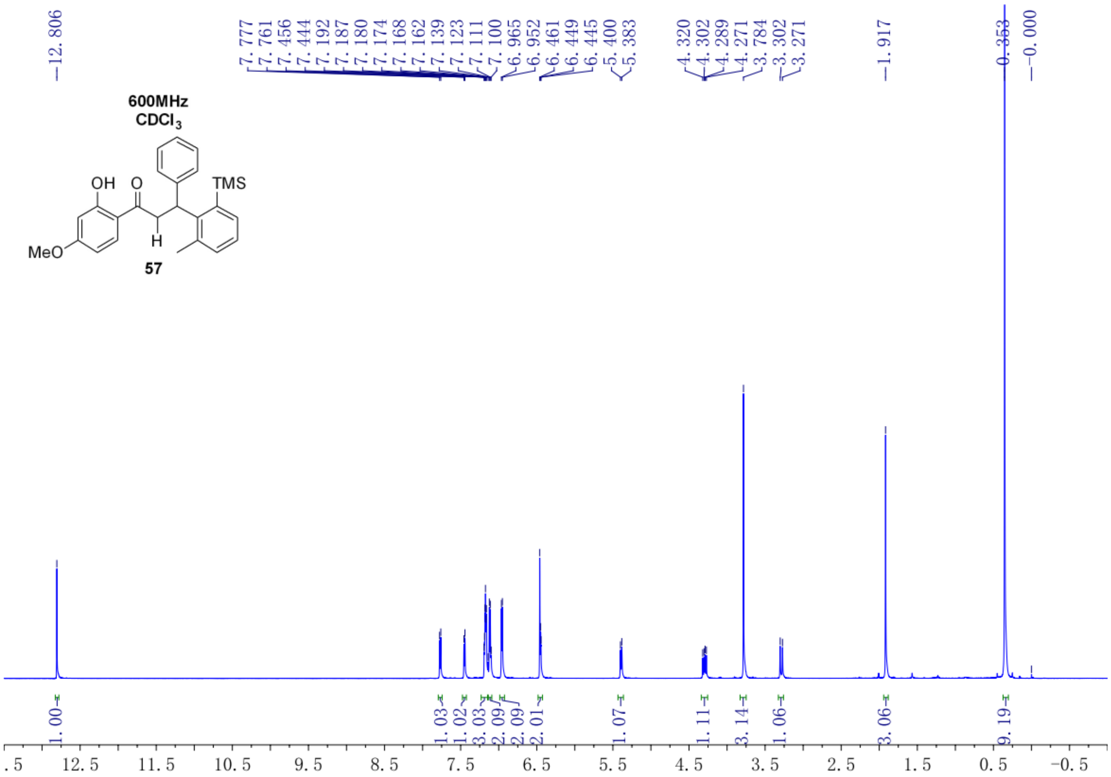
600MHz
CDCl3
OH
O
TMS
H
MeO
57
12.806
7.777
7.761
7.456
7.444
7.192
7.187
7.180
7.174
7.168
7.162
7.139
7.123
7.111
7.100
6.965
6.952
6.461
6.449
6.445
5.400
5.383
4.320
4.302
4.289
4.271
3.784
3.302
3.271
1.917
0.353
0.000
1.00
1.03
1.02
3.03
2.09
2.09
2.01
1.07
1.11
3.14
1.06
3.06
9.19
12.5
11.5
10.5
9.5
8.5
7.5
6.5
5.5
4.5
3.5
2.5
1.5
0.5
-0.5

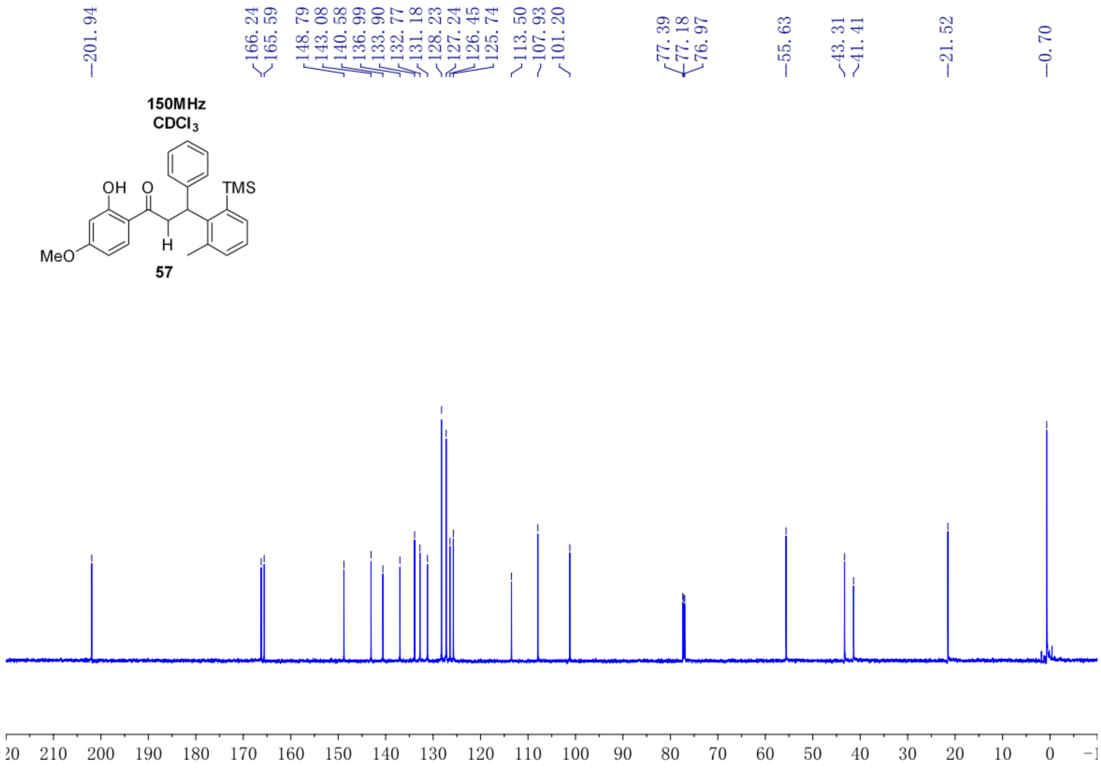
150MHz
CDCl3
OH
O
TMS
H
MeO
57
201.94
166.24
165.59
148.79
143.08
140.58
136.99
133.90
132.77
131.18
128.23
127.24
126.45
125.74
113.50
107.93
101.20
77.39
77.18
76.97
55.63
43.31
41.41
21.52
0.70
210
200
190
180
170
160
150
140
130
120
110
100
90
80
70
60
50
40
30
20
10
0

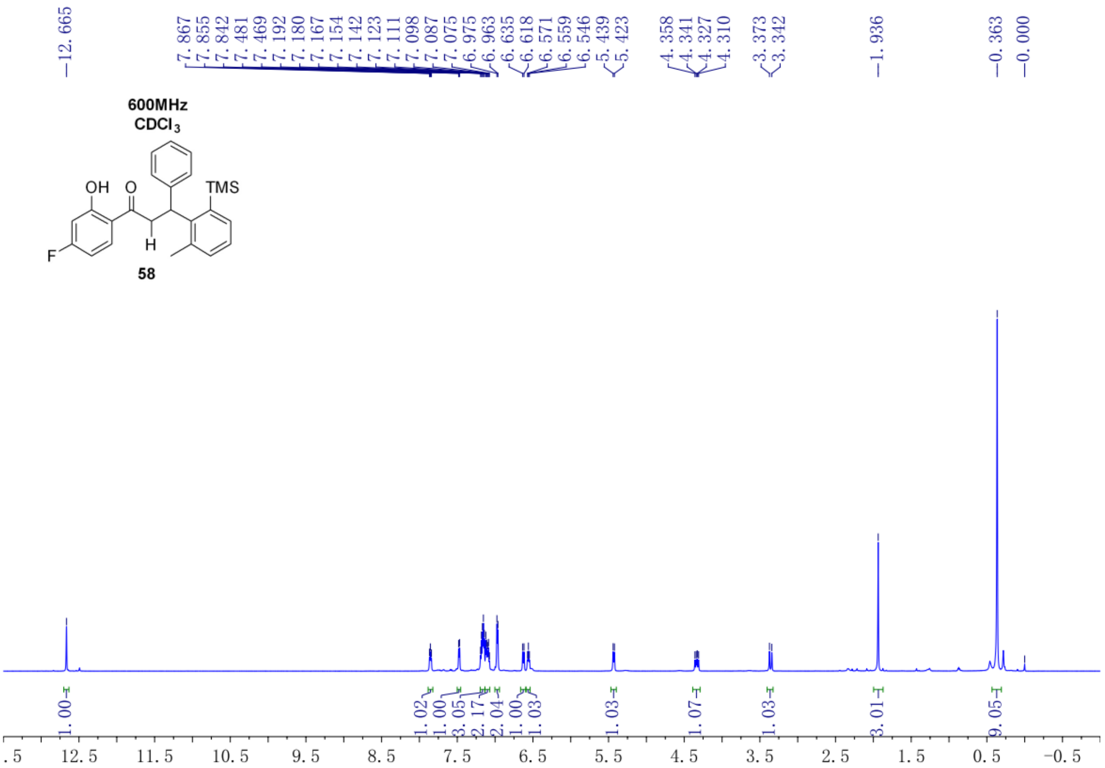
12.665
7.867
7.855
7.842
7.481
7.469
7.192
7.180
7.167
7.154
7.142
7.123
7.111
7.098
7.087
7.075
6.975
6.963
6.635
6.618
6.571
6.559
6.546
5.439
5.423
4.358
4.341
4.327
4.310
3.373
3.342
1.936
0.363
0.000
600MHz
CDCl3
OH
O
TMS
F
H
58
1.00
1.02
1.00
3.05
2.17
2.04
1.00
1.03
1.03
1.07
1.03
3.01
9.05
12.5
11.5
10.5
9.5
8.5
7.5
6.5
5.5
4.5
3.5
2.5
1.5
0.5
-0.5

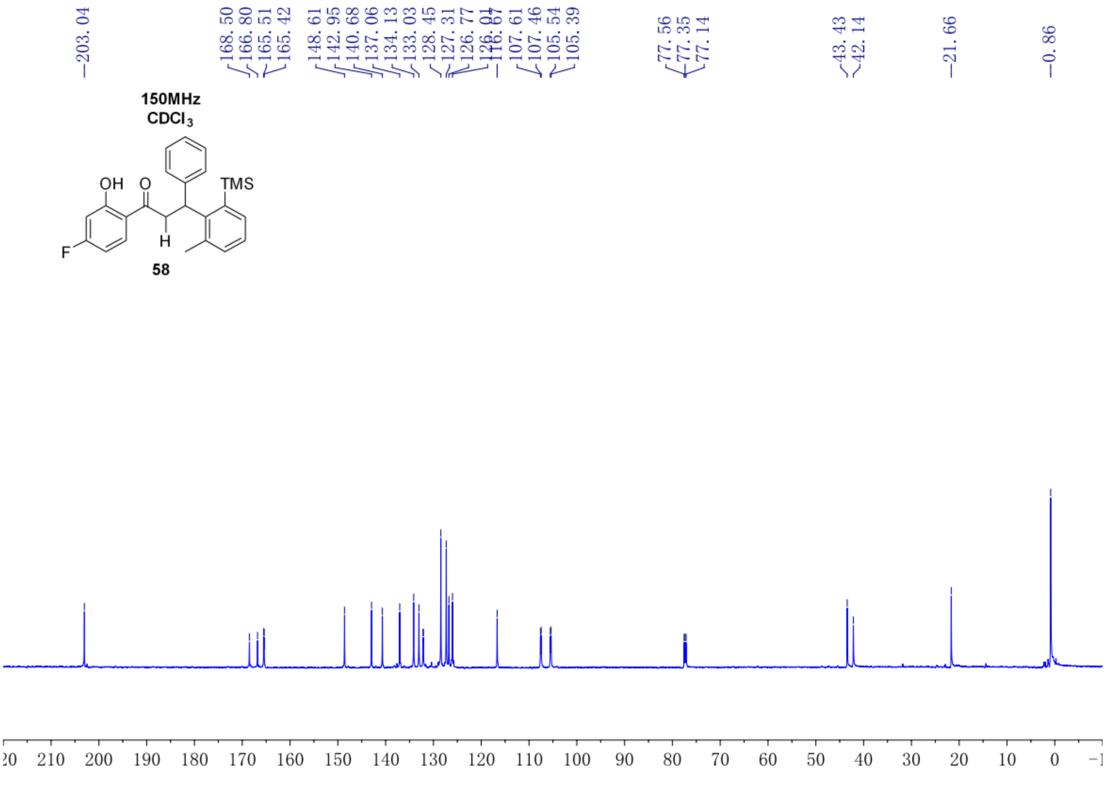
203.04
168.50
166.80
165.51
165.42
148.61
142.95
140.68
137.06
134.13
133.03
128.45
127.31
126.77
126.01
116.67
107.61
107.46
105.54
105.39
77.56
77.35
77.14
43.43
42.14
21.66
0.86
150MHz
CDCl3
OH
O
TMS
F
H
58
210
200
190
180
170
160
150
140
130
120
110
100
90
80
70
60
50
40
30
20
10
0

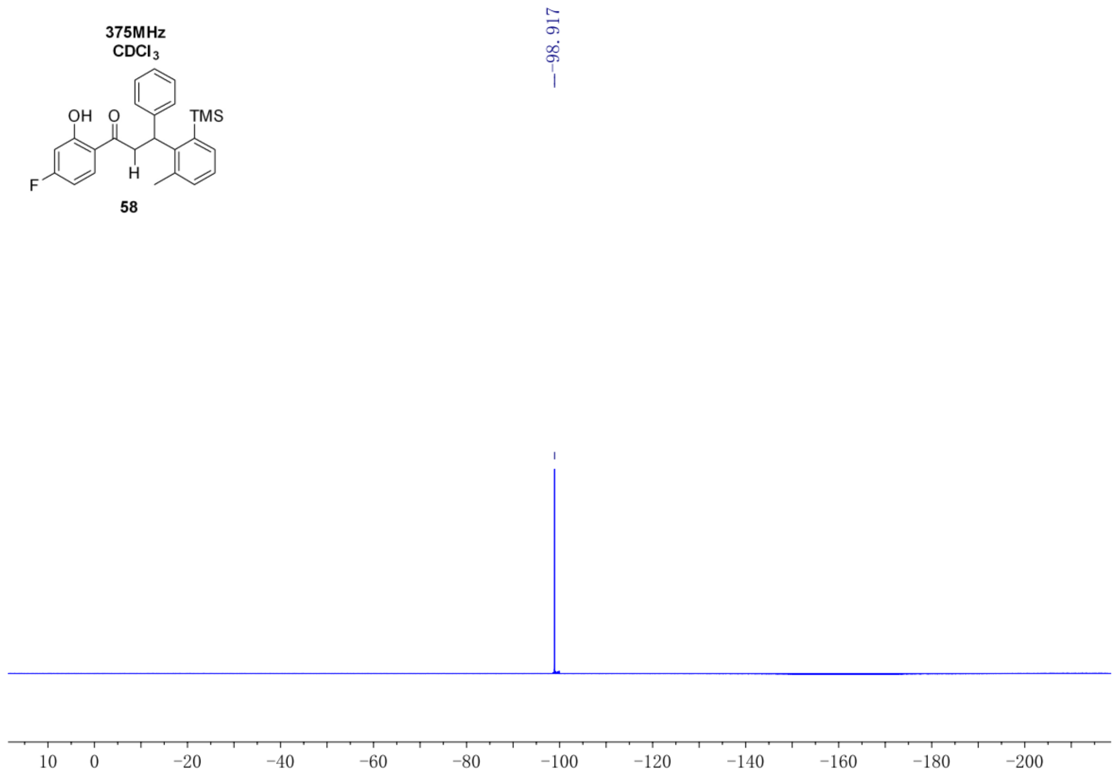
375MHz
CDCl3
OH
O
TMS
H
F
58
−98.917
10
0
−20
−40
−60
−80
−100
−120
−140
−160
−180
−200

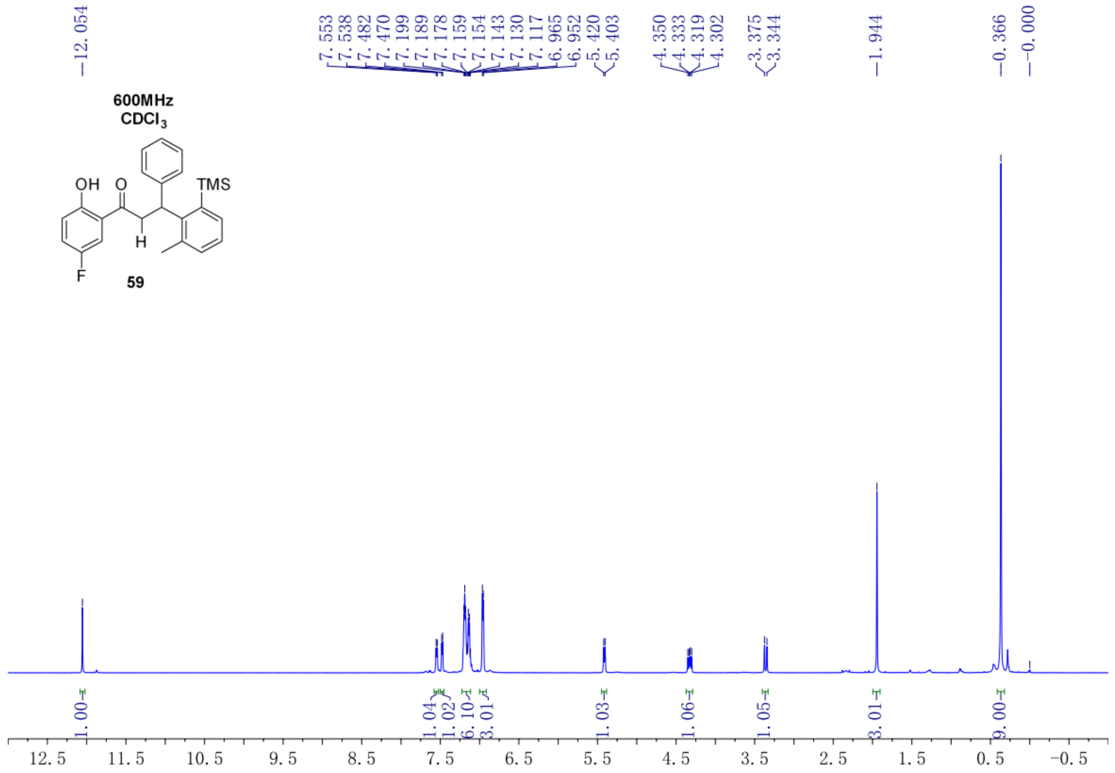
600MHz
CDCl3
OH
O
TMS
H
F
59
12.054
7.553
7.538
7.482
7.470
7.199
7.189
7.178
7.159
7.154
7.143
7.130
7.117
6.965
6.952
5.420
5.403
4.350
4.333
4.319
4.302
3.375
3.344
1.944
0.366
0.000
1.00
1.04
1.02
6.10
3.01
1.03
1.06
1.05
3.01
9.00
12.5
11.5
10.5
9.5
8.5
7.5
6.5
5.5
4.5
3.5
2.5
1.5
0.5
−0.5

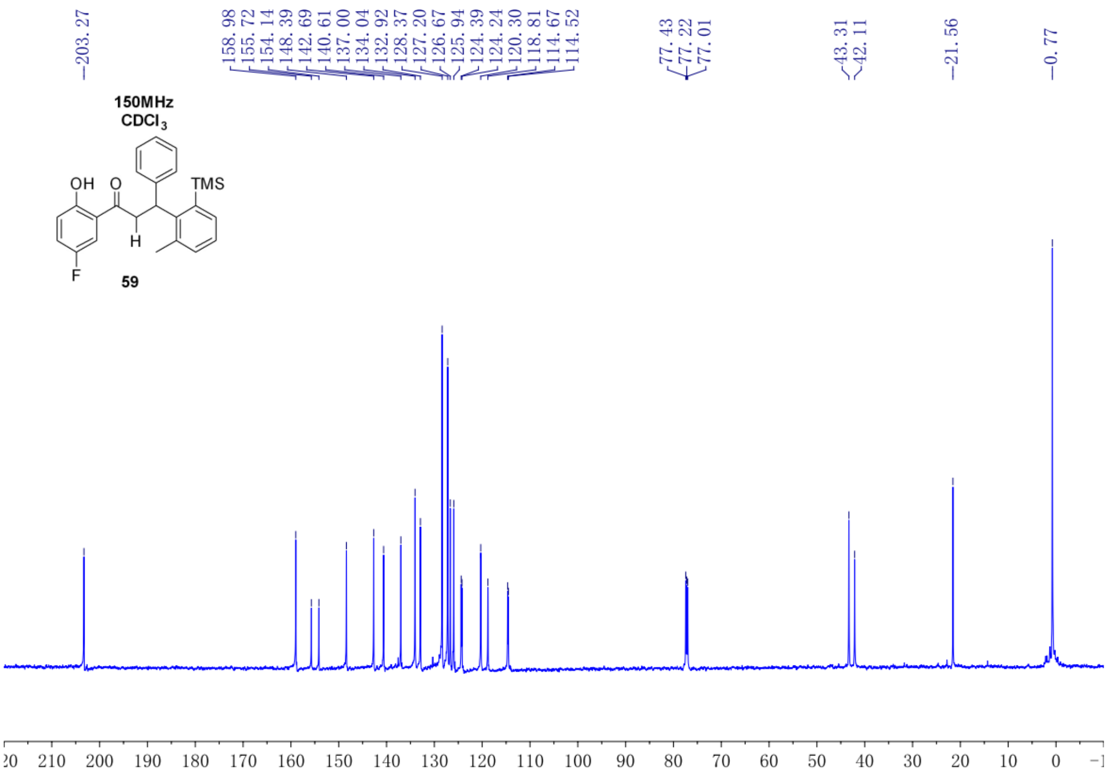
150MHz
CDCl3
OH O
TMS
H
F
59
203.27
158.98
155.72
154.14
148.39
142.69
140.61
137.00
134.04
132.92
128.37
127.20
126.67
125.94
124.39
124.24
120.30
118.81
114.67
114.52
77.43
77.22
77.01
43.31
42.11
21.56
0.77

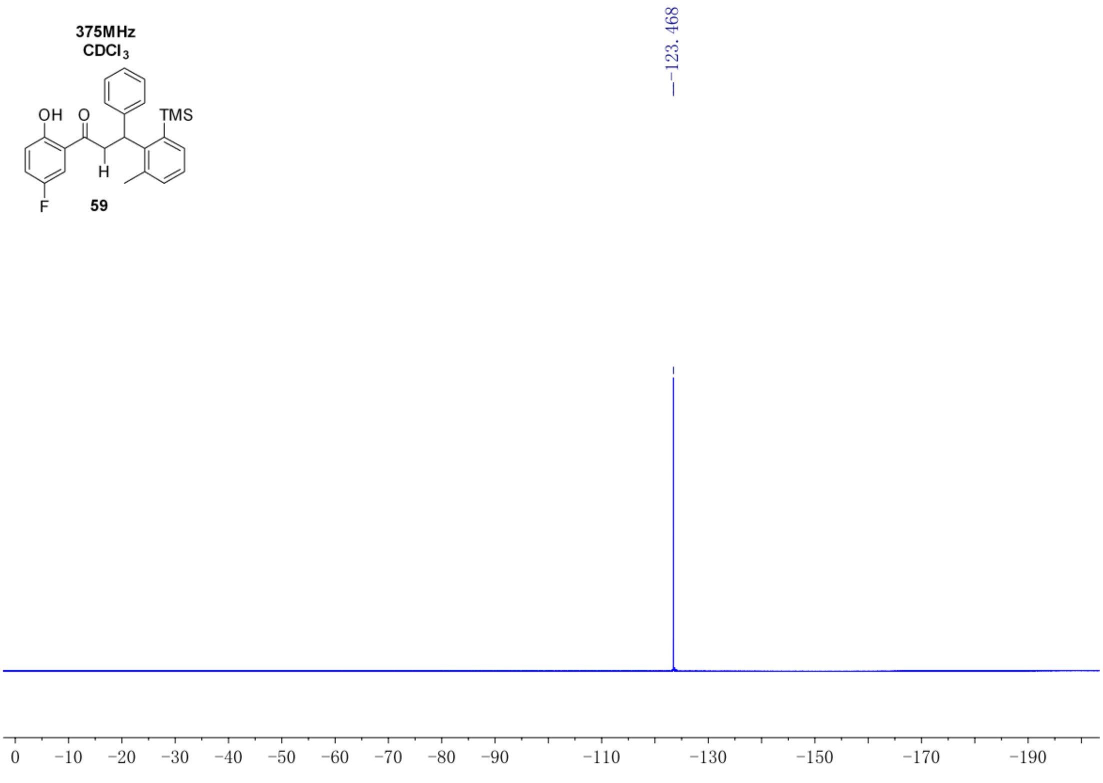
375MHz
CDCl3
OH O
TMS
H
F
59
-123.468

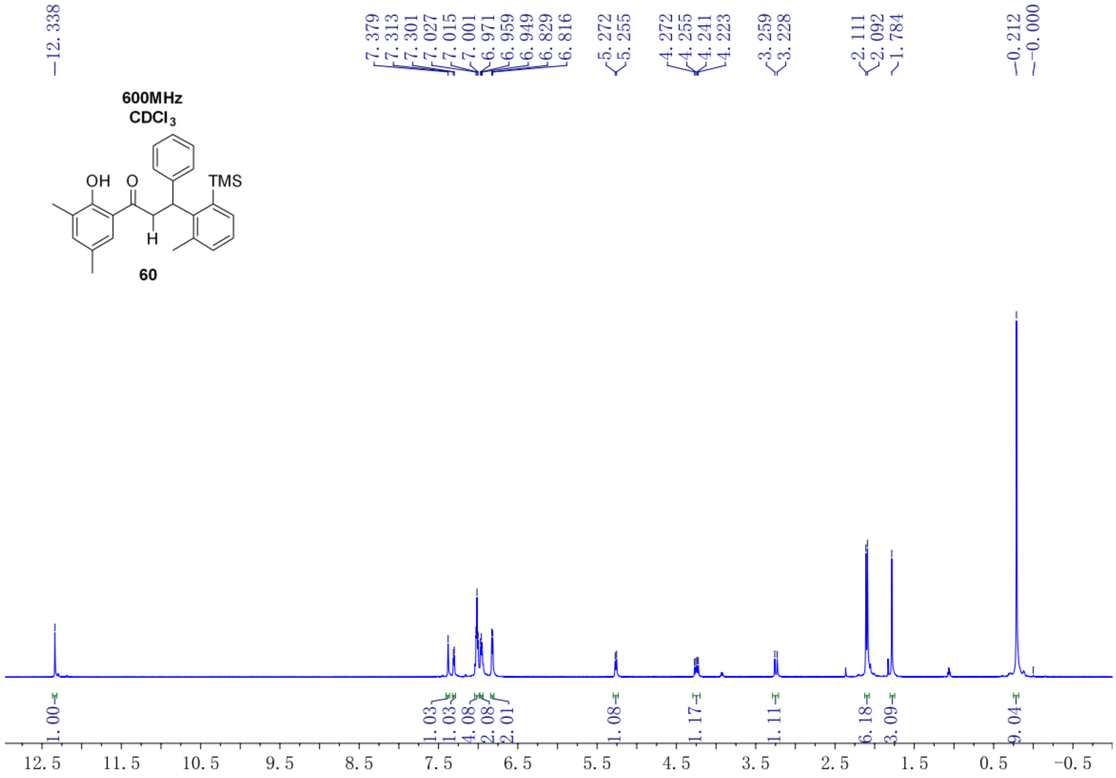
12.338
7.379
7.313
7.301
7.027
7.015
7.001
6.971
6.959
6.949
6.829
6.816
5.272
5.255
4.272
4.255
4.241
4.223
3.259
3.228
2.111
2.092
1.784
0.212
0.000
600MHz
CDCl3
OH
O
TMS
H
60
1.00
1.03
1.03
4.08
2.08
2.01
1.08
1.17
1.11
6.18
3.09
9.04
12.5
11.5
10.5
9.5
8.5
7.5
6.5
5.5
4.5
3.5
2.5
1.5
0.5
-0.5

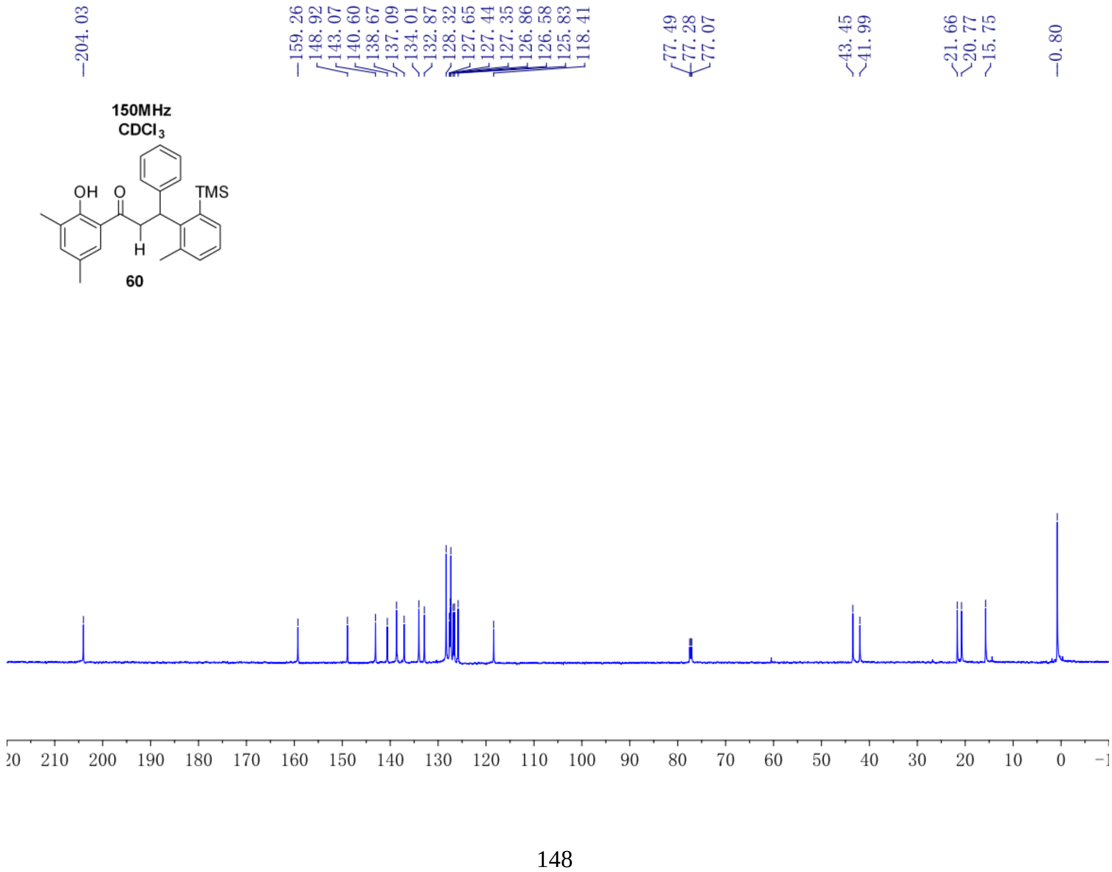
204.03
159.26
148.92
143.07
140.60
138.67
137.09
134.01
132.87
128.32
127.65
127.44
127.35
126.86
126.58
125.83
118.41
77.49
77.28
77.07
43.45
41.99
21.66
20.77
15.75
0.80
150MHz
CDCl3
OH
O
TMS
H
60
210
200
190
180
170
160
150
140
130
120
110
100
90
80
70
60
50
40
30
20
10
0

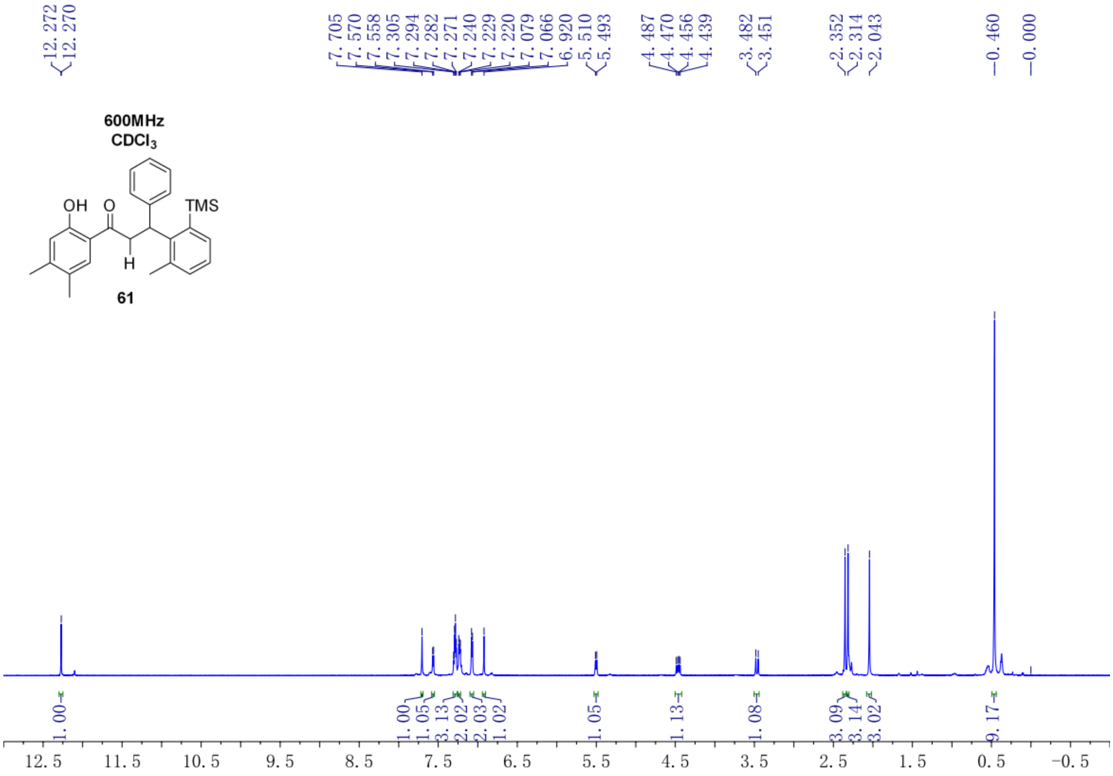
12.272 12.270
7.705 7.570 7.558 7.305 7.294 7.282 7.271 7.240 7.229 7.220 7.079 7.066 6.920 5.510 5.493
4.487 4.470 4.456 4.439
3.482 3.451
2.352 2.314 2.043
0.460 0.000
600MHz
CDCl3
OH O
TMS
H
61
1.00 1.00 1.05 3.13 2.02 2.03 1.02 1.05 1.13 1.08 3.09 3.14 3.02 9.17
12.5 11.5 10.5 9.5 8.5 7.5 6.5 5.5 4.5 3.5 2.5 1.5 0.5 -0.5

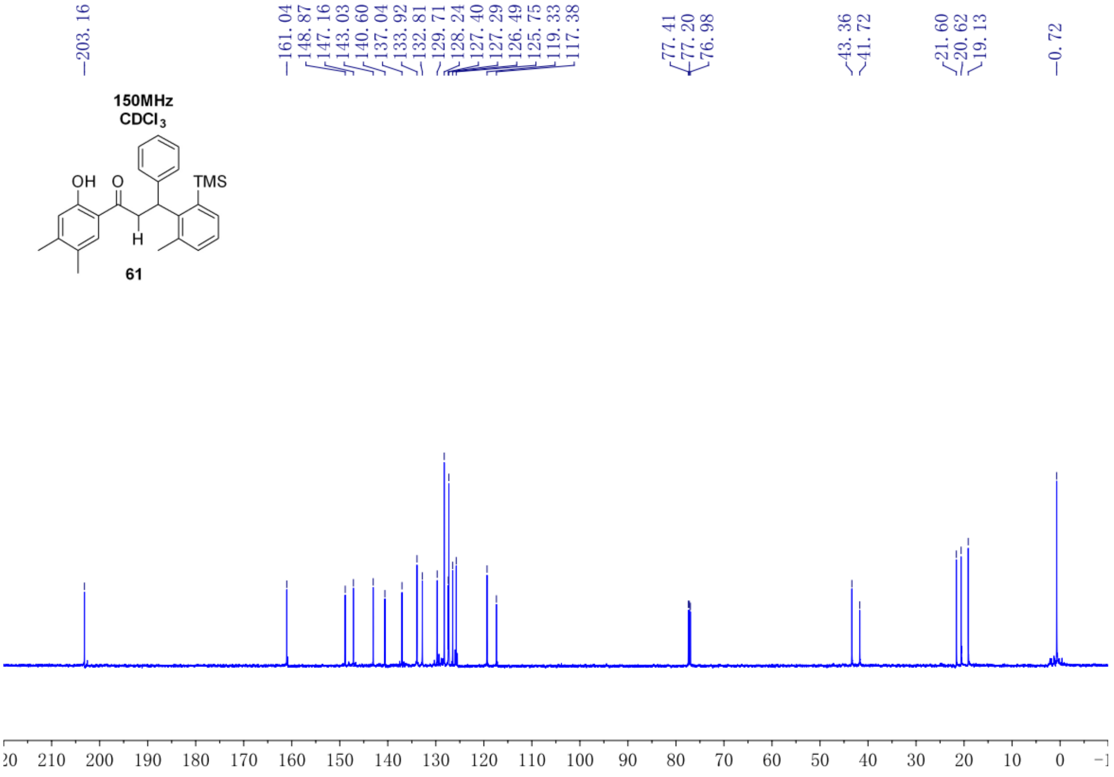
203.16
161.04 148.87 147.16 143.03 140.60 137.04 133.92 132.81 129.71 128.24 127.40 127.29 126.49 125.75 119.33 117.38
77.41 77.20 76.98
43.36 41.72
21.60 20.62 19.13
0.72
150MHz
CDCl3
OH O
TMS
H
61
210 200 190 180 170 160 150 140 130 120 110 100 90 80 70 60 50 40 30 20 10 0

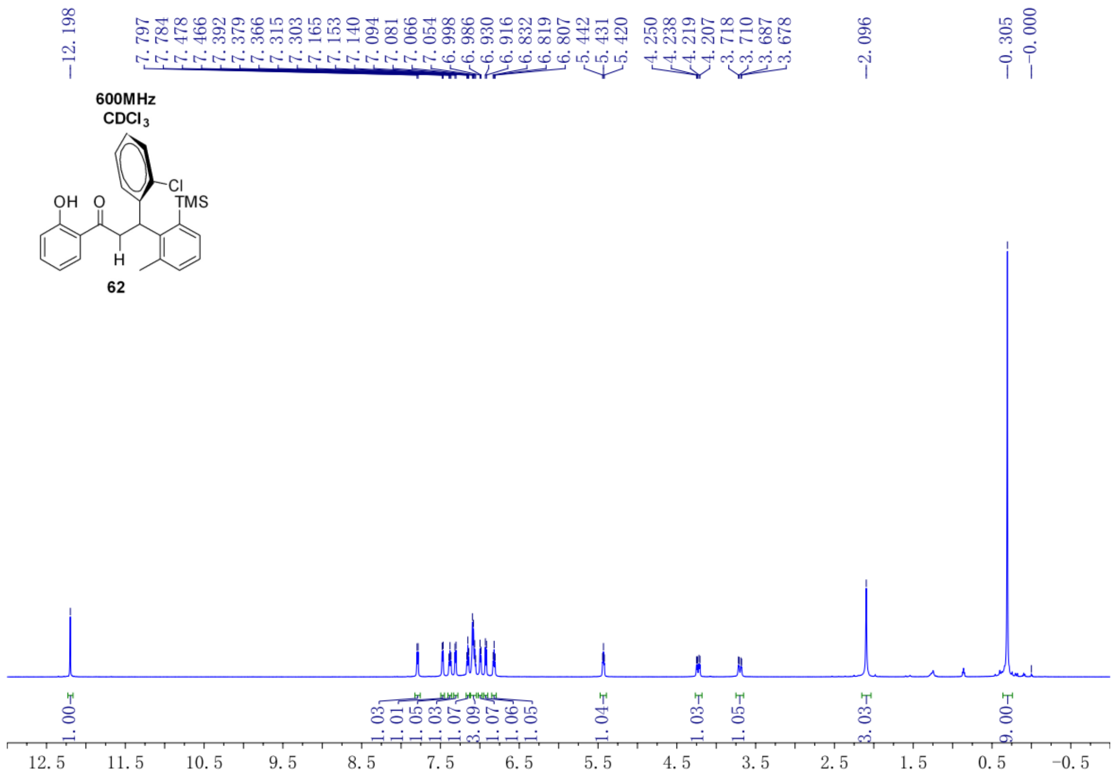

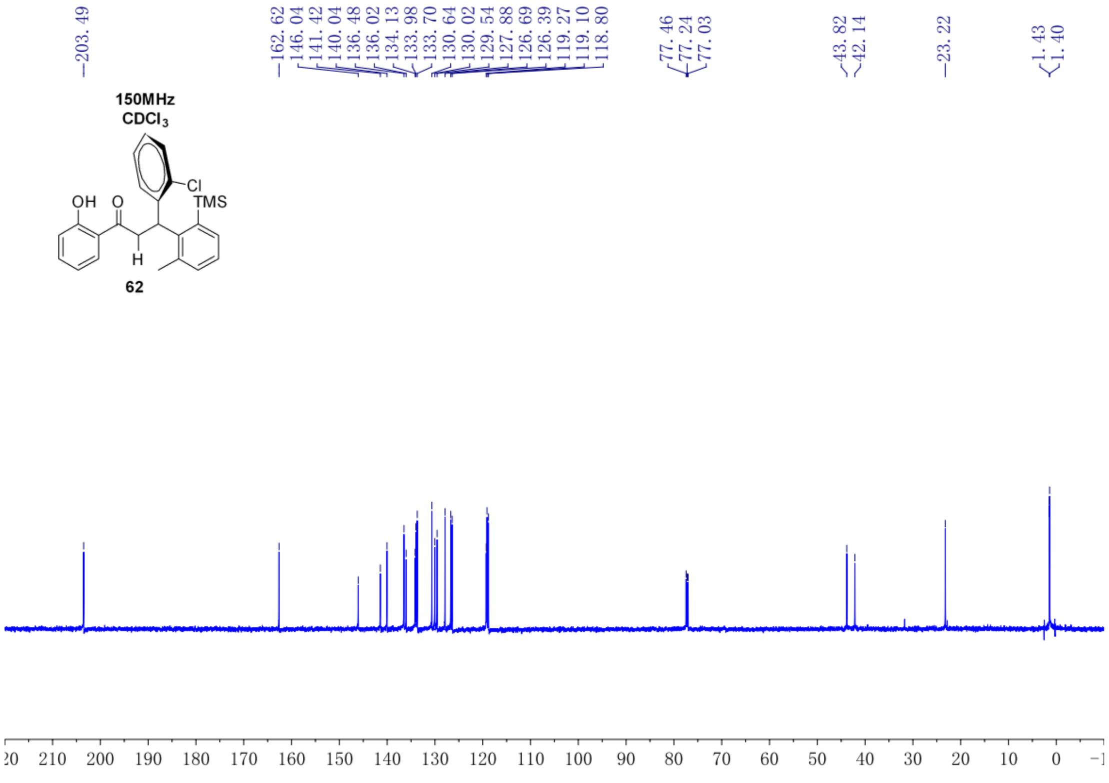

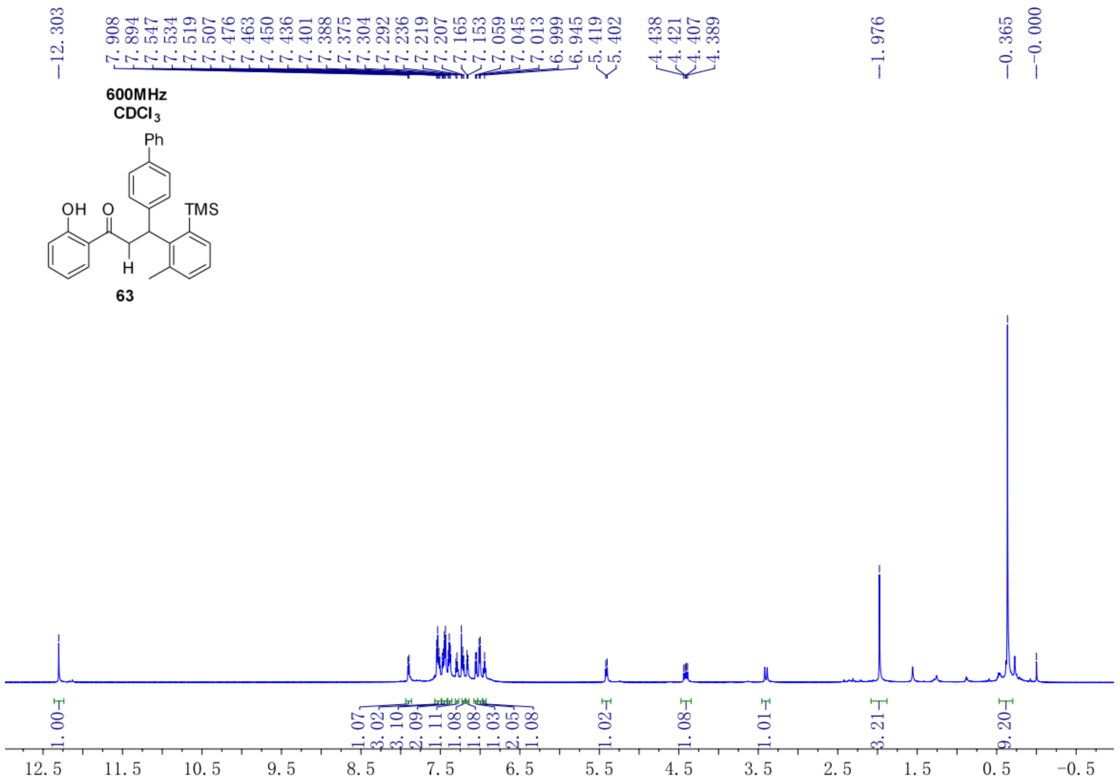
12.303
7.908
7.894
7.547
7.534
7.519
7.507
7.476
7.463
7.450
7.436
7.401
7.388
7.375
7.304
7.292
7.236
7.219
7.207
7.165
7.153
7.059
7.045
7.013
6.999
6.945
5.419
5.402
4.438
4.421
4.407
4.389
1.976
0.365
-0.000
600MHz
CDCl3
Ph
OH
O
TMS
H
63
1.00
1.07
3.02
3.10
2.09
1.11
1.08
1.08
1.03
2.05
1.08
1.02
1.08
1.01
3.21
9.20
12.5
11.5
10.5
9.5
8.5
7.5
6.5
5.5
4.5
3.5
2.5
1.5
0.5
-0.5

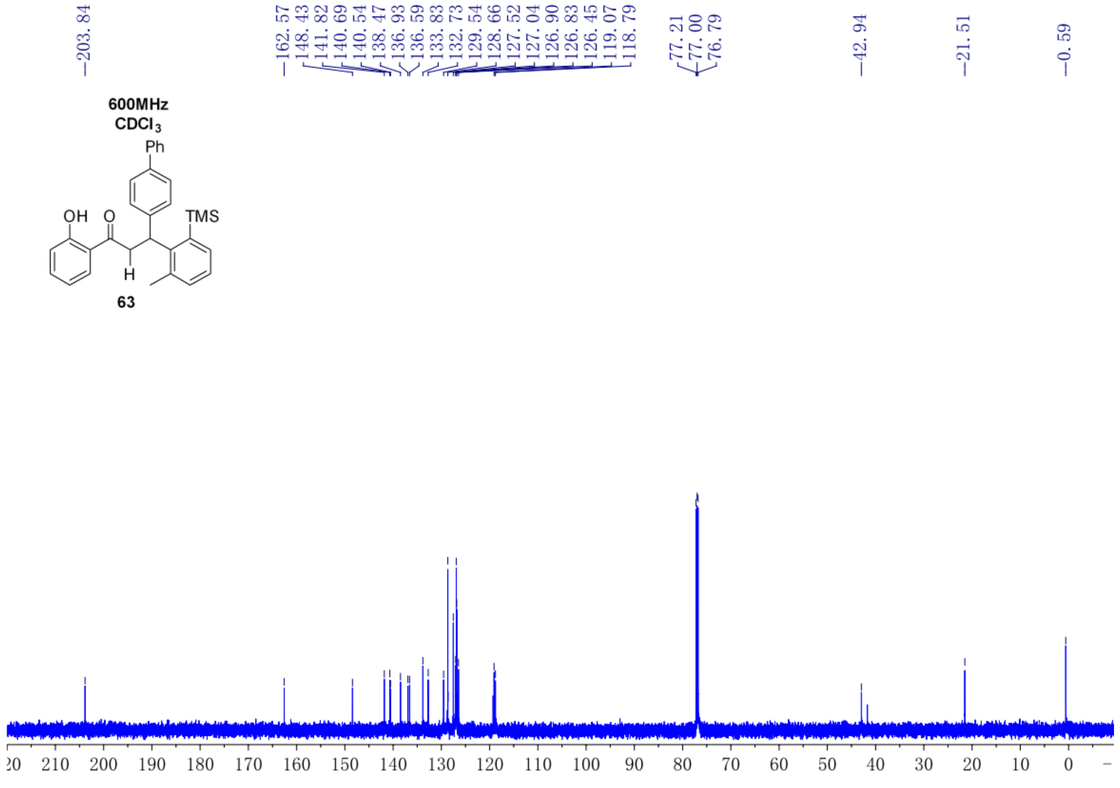
203.84
162.57
148.43
141.82
140.69
140.54
138.47
136.93
136.59
133.83
132.73
129.54
128.66
127.52
127.04
126.90
126.83
126.45
119.07
118.79
77.21
77.00
76.79
42.94
21.51
0.59
600MHz
CDCl3
Ph
OH
O
TMS
H
63
210
200
190
180
170
160
150
140
130
120
110
100
90
80
70
60
50
40
30
20
10
0

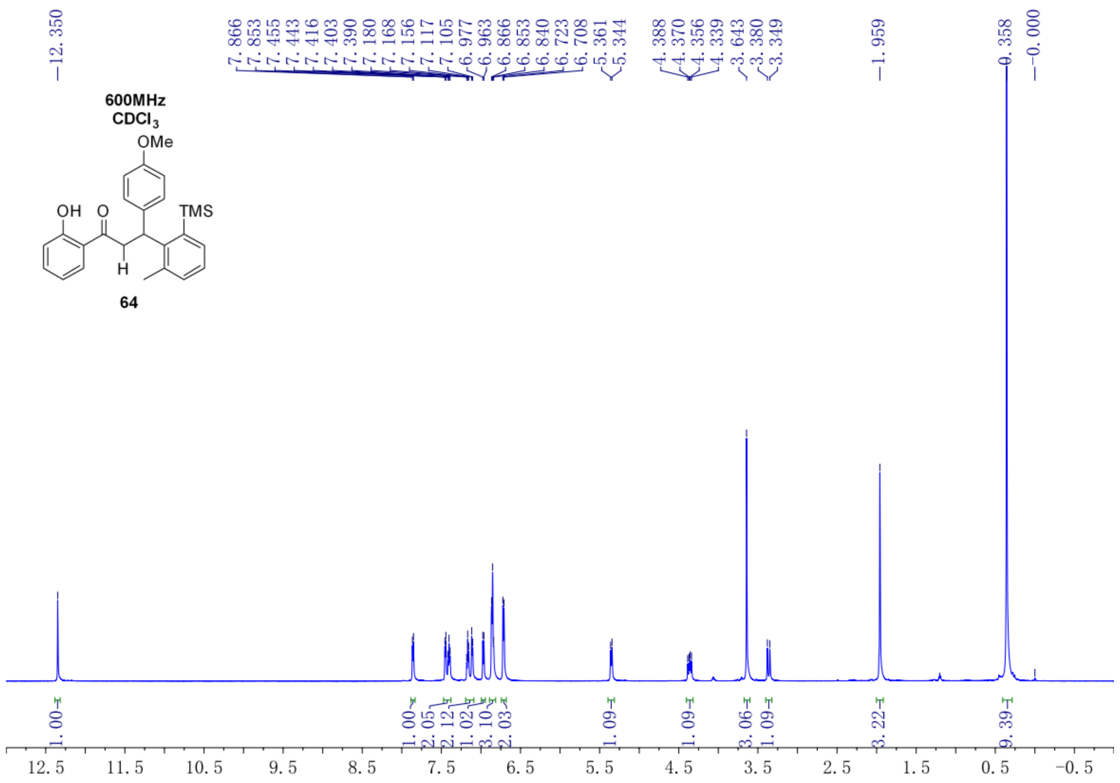
600MHz
CDCl3
OMe
OH O
TMS
H
64
12.350
7.866 7.853 7.455 7.443 7.416 7.403 7.390 7.180 7.168 7.156 7.117 7.105 6.977 6.963 6.866 6.853 6.840 6.723 6.708 5.361 5.344
4.388 4.370 4.356 4.339 3.643 3.380 3.349
1.959
0.358 0.000
1.00 1.00 2.05 2.12 1.02 3.10 2.03 1.09 1.09 3.06 1.09 3.22 9.39
12.5 11.5 10.5 9.5 8.5 7.5 6.5 5.5 4.5 3.5 2.5 1.5 0.5 -0.5

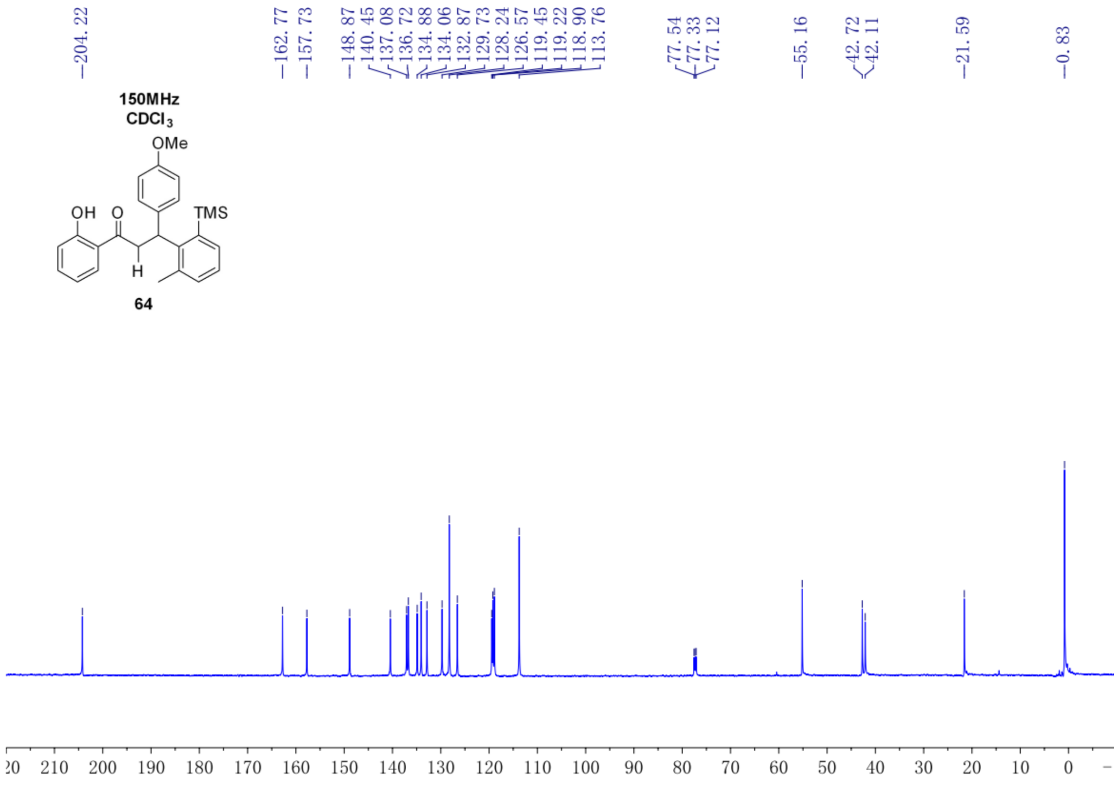
150MHz
CDCl3
OMe
OH O
TMS
H
64
204.22
162.77 157.73
148.87 140.45 137.08 136.72 134.88 134.06 132.87 129.73 128.24 126.57 119.45 119.22 118.90 113.76
77.54 77.33 77.12
55.16
42.72 42.11
21.59
0.83
210 200 190 180 170 160 150 140 130 120 110 100 90 80 70 60 50 40 30 20 10 0

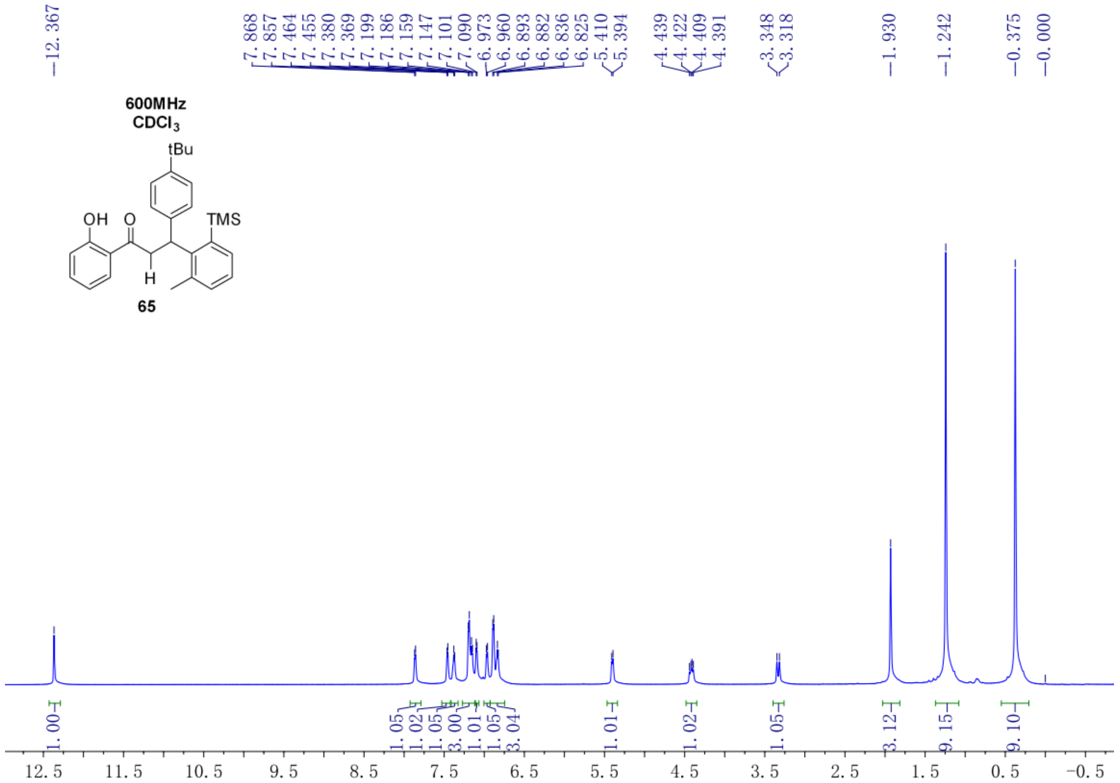

12.367
7.868
7.857
7.464
7.455
7.380
7.369
7.199
7.186
7.159
7.147
7.101
7.090
6.973
6.960
6.893
6.882
6.836
6.825
5.410
5.394
4.439
4.422
4.409
4.391
3.348
3.318
1.930
1.242
0.375
0.000
600MHz
CDCl3
tBu
OH
O
TMS
H
65
1.00
1.05
1.02
1.05
3.00
1.01
1.05
3.04
1.01
1.02
1.05
3.12
9.15
9.10
12.5
11.5
10.5
9.5
8.5
7.5
6.5
5.5
4.5
3.5
2.5
1.5
0.5
-0.5

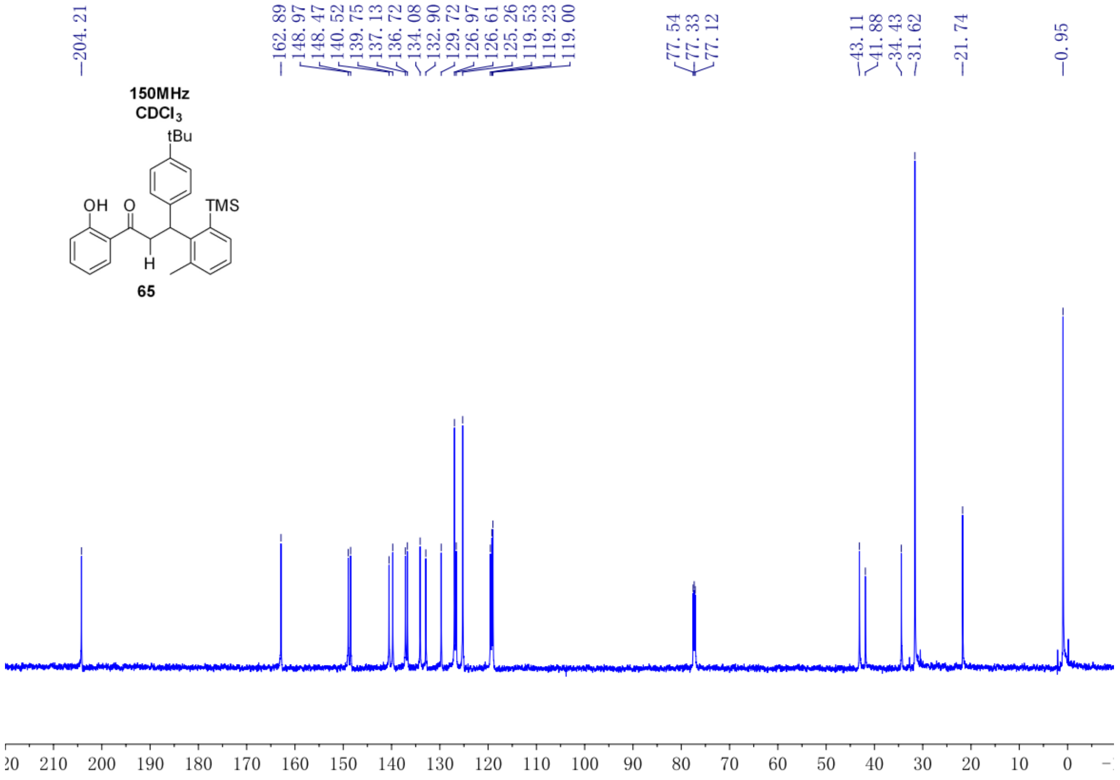

204.21
162.89
148.97
148.47
140.52
139.75
137.13
136.72
134.08
132.90
129.72
126.97
126.61
125.26
119.53
119.23
119.00
77.54
77.33
77.12
43.11
41.88
34.43
31.62
21.74
0.95
150MHz
CDCl3
tBu
OH
O
TMS
H
65
210
200
190
180
170
160
150
140
130
120
110
100
90
80
70
60
50
40
30
20
10
0

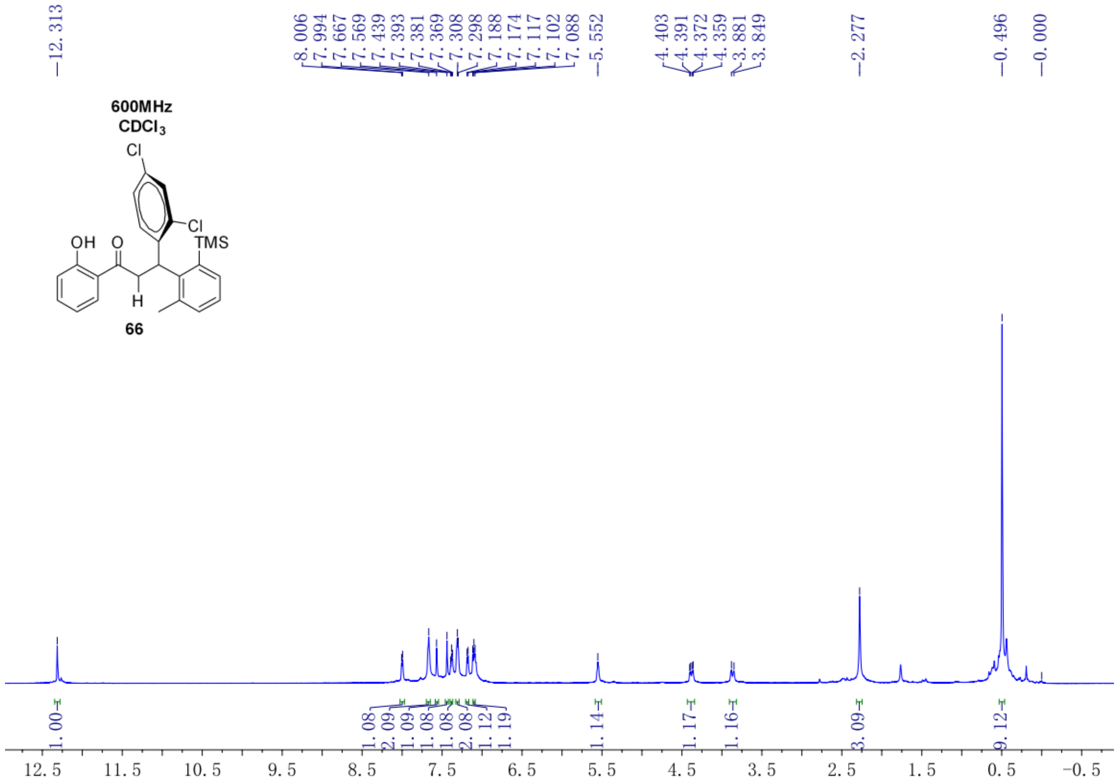

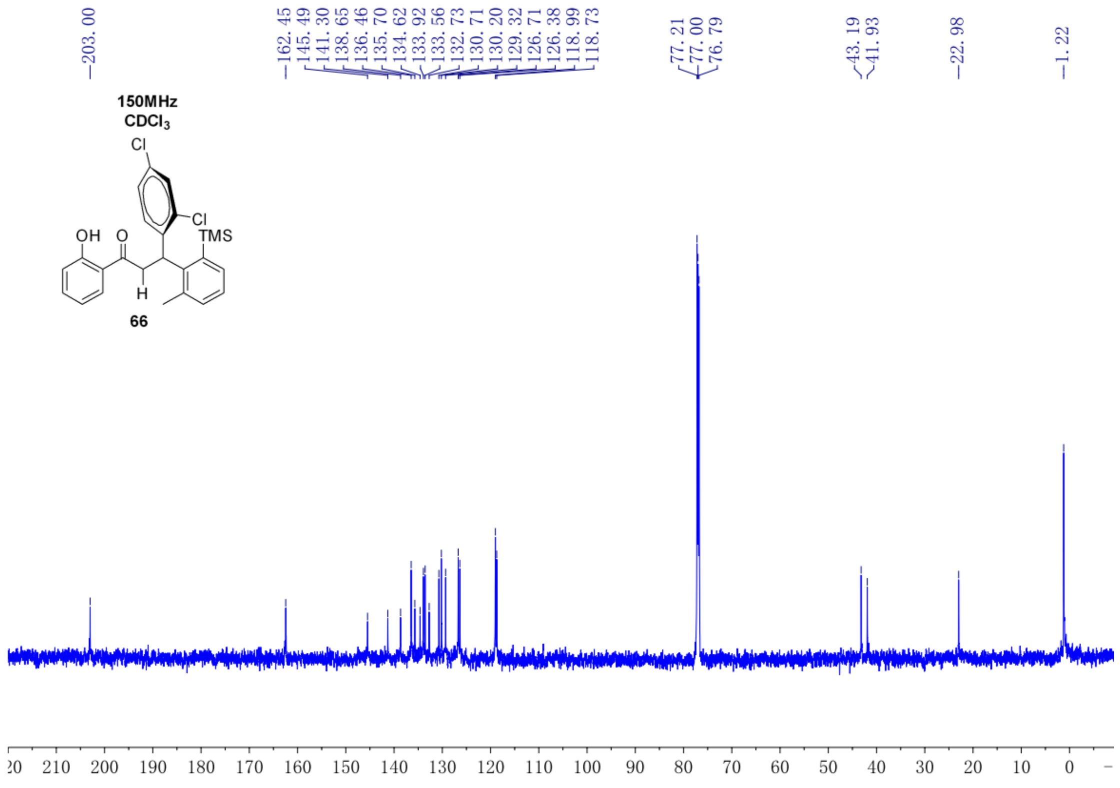

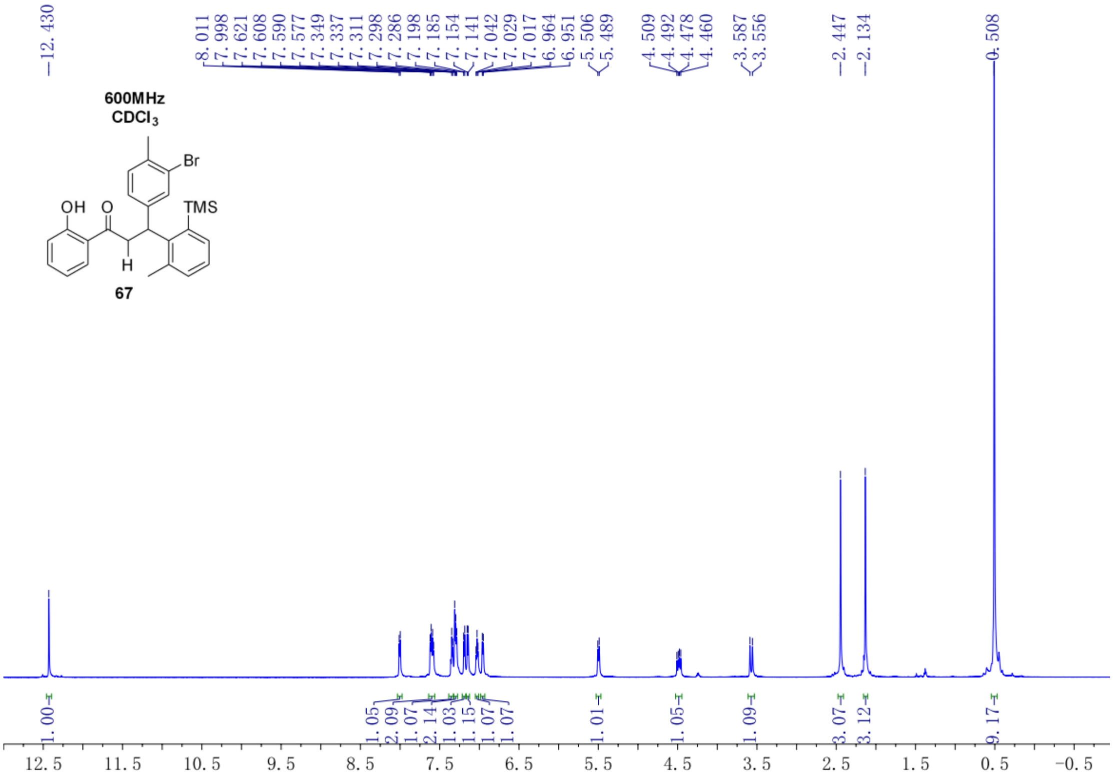
600MHz
CDCl3
Br
OH
O
TMS
H
67
12.430
8.011 7.998 7.621 7.608 7.590 7.577 7.349 7.337 7.311 7.298 7.286 7.198 7.185 7.154 7.141 7.042 7.029 7.017 6.964 6.951
5.506 5.489
4.509 4.492 4.478 4.460
3.587 3.556
2.447
2.134
0.508
1.00 1.05 2.09 1.07 2.14 1.03 1.15 1.07 1.07 1.01 1.05 1.09 3.07 3.12 9.17
12.5 11.5 10.5 9.5 8.5 7.5 6.5 5.5 4.5 3.5 2.5 1.5 0.5 −0.5

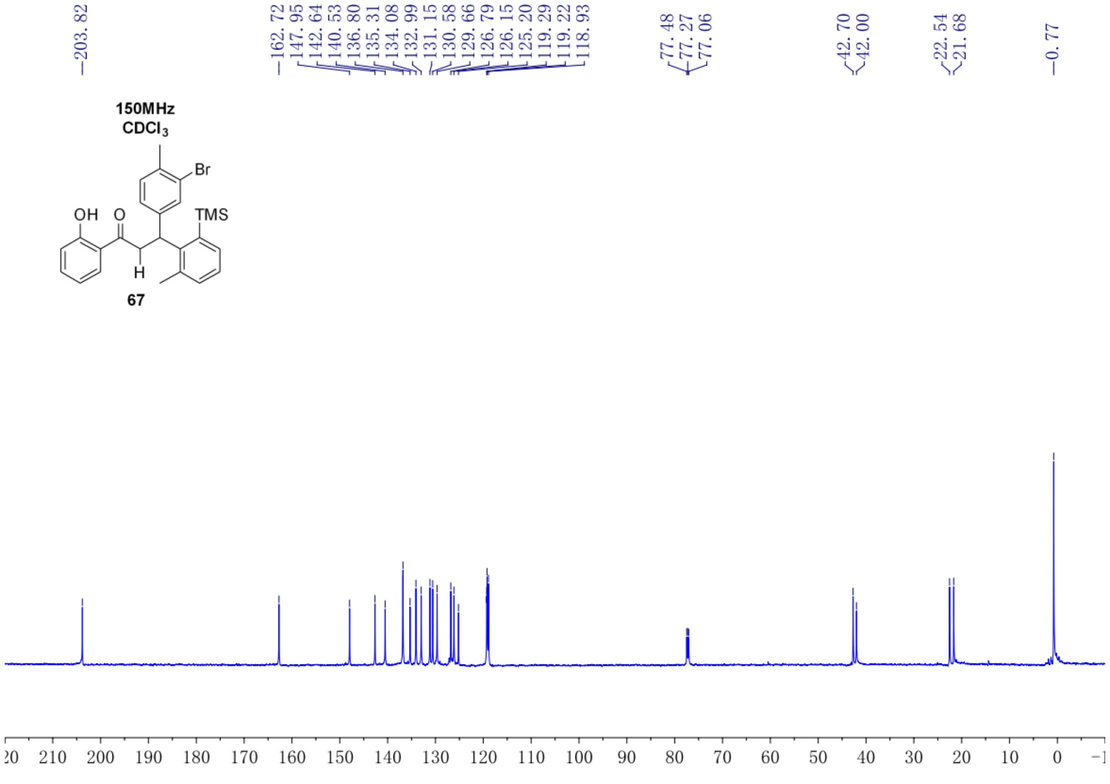
150MHz
CDCl3
Br
OH
O
TMS
H
67
203.82
162.72 147.95 142.64 140.53 136.80 135.31 134.08 132.99 131.15 130.58 129.66 126.79 126.15 125.20 119.29 119.22 118.93
77.48 77.27 77.06
42.70 42.00
22.54 21.68
0.77
210 200 190 180 170 160 150 140 130 120 110 100 90 80 70 60 50 40 30 20 10 0

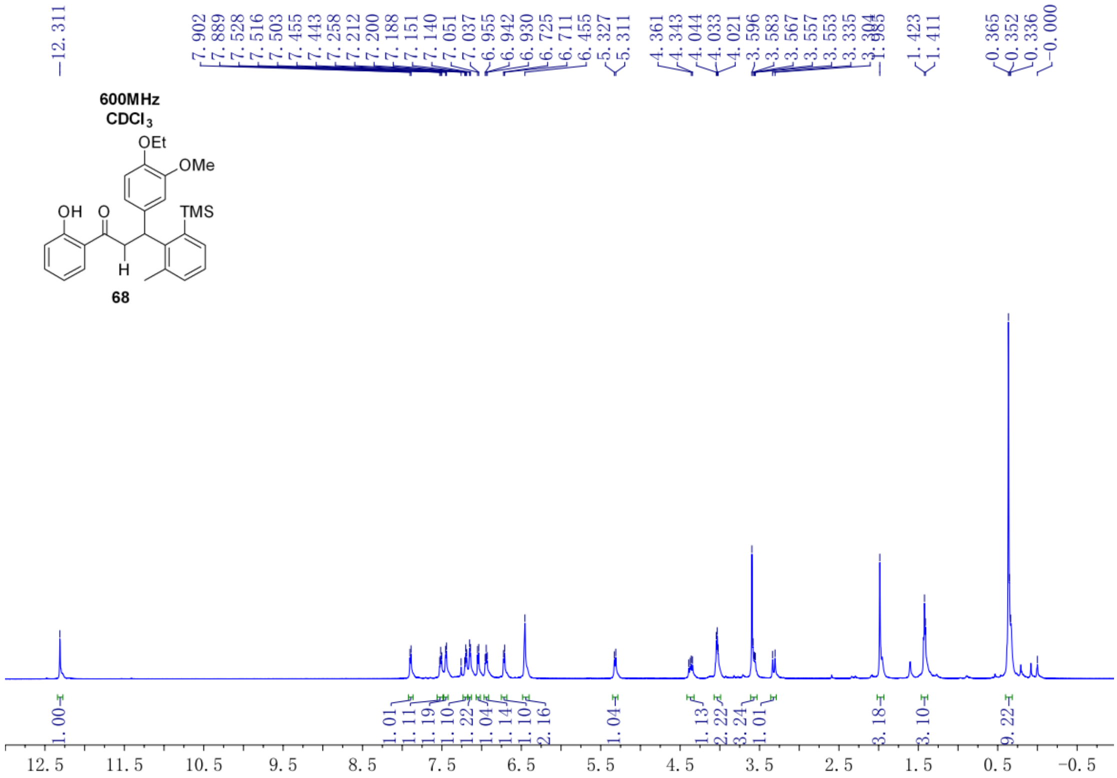

600MHz
CDCl3
OEt
OMe
OH
O
TMS
H
68
12.311
7.902 7.889 7.528 7.516 7.503 7.455 7.443 7.258 7.212 7.200 7.188 7.151 7.140 7.051 7.037 6.955 6.942 6.930 6.725 6.711 6.455 5.327 5.311
4.361 4.343 4.044 4.033 4.021 3.596 3.583 3.567 3.557 3.553 3.335 3.304 1.985
1.423 1.411
0.365 0.352 0.336 -0.000
1.00 1.01 1.11 1.19 1.10 1.22 1.04 1.14 1.10 2.16 1.04 1.13 2.22 3.24 1.01 3.18 3.10 9.22
12.5 11.5 10.5 9.5 8.5 7.5 6.5 5.5 4.5 3.5 2.5 1.5 0.5 -0.5

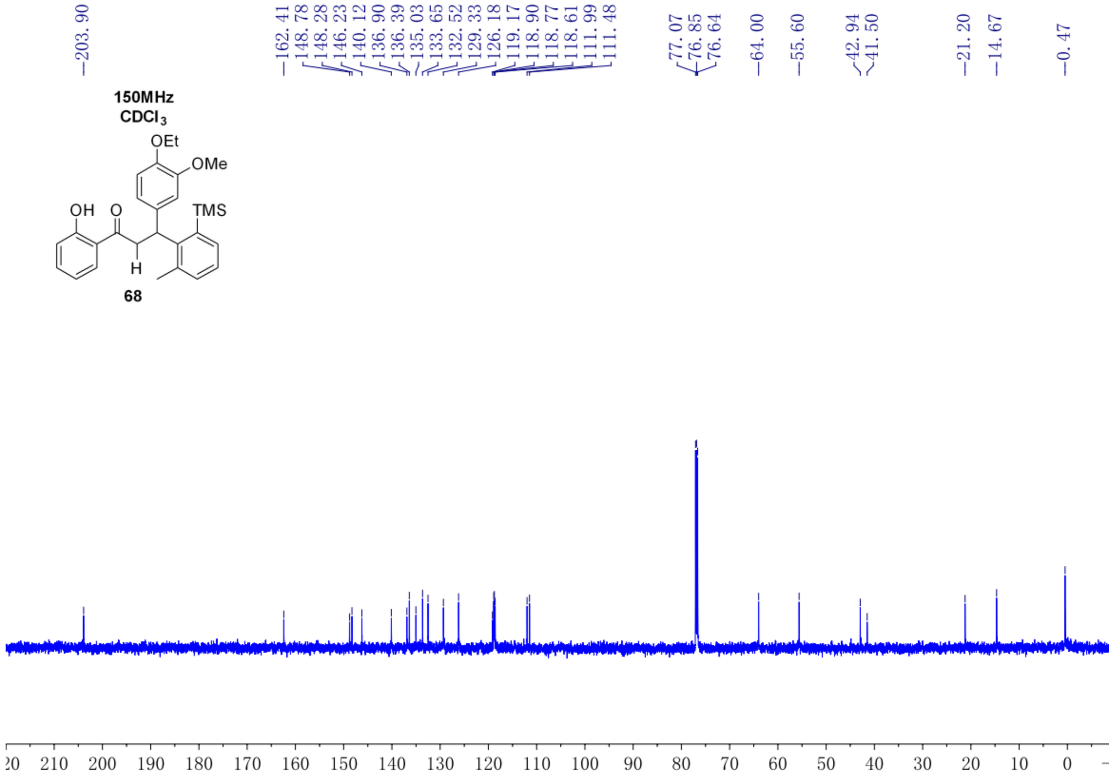

150MHz
CDCl3
OEt
OMe
OH
O
TMS
H
68
203.90
162.41 148.78 148.28 146.23 140.12 136.90 136.39 135.03 133.65 132.52 129.33 126.18 119.17 118.90 118.77 118.61 111.99 111.48
77.07 76.85 76.64
64.00
55.60
42.94 41.50
21.20
14.67
0.47
210 200 190 180 170 160 150 140 130 120 110 100 90 80 70 60 50 40 30 20 10 0

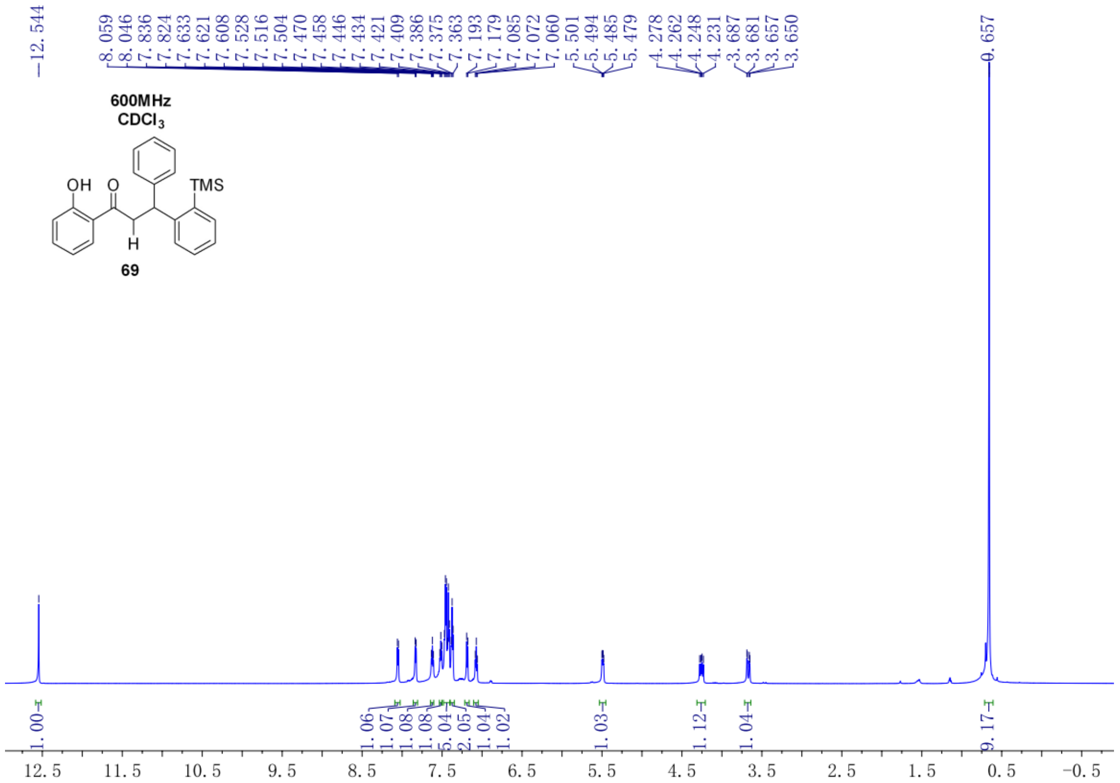

12.544
8.059 8.046 7.836 7.824 7.633 7.621 7.608 7.528 7.516 7.504 7.470 7.458 7.446 7.434 7.421 7.409 7.386 7.375 7.363 7.193 7.179 7.085 7.072 7.060 5.501 5.494 5.485 5.479 4.278 4.262 4.248 4.231 3.687 3.681 3.657 3.650
0.657
600MHz
CDCl3
OH
O
TMS
H
69
1.00 1.06 1.07 1.08 1.08 5.04 2.05 1.04 1.02 1.03 1.12 1.04 9.17
12.5 11.5 10.5 9.5 8.5 7.5 6.5 5.5 4.5 3.5 2.5 1.5 0.5 -0.5

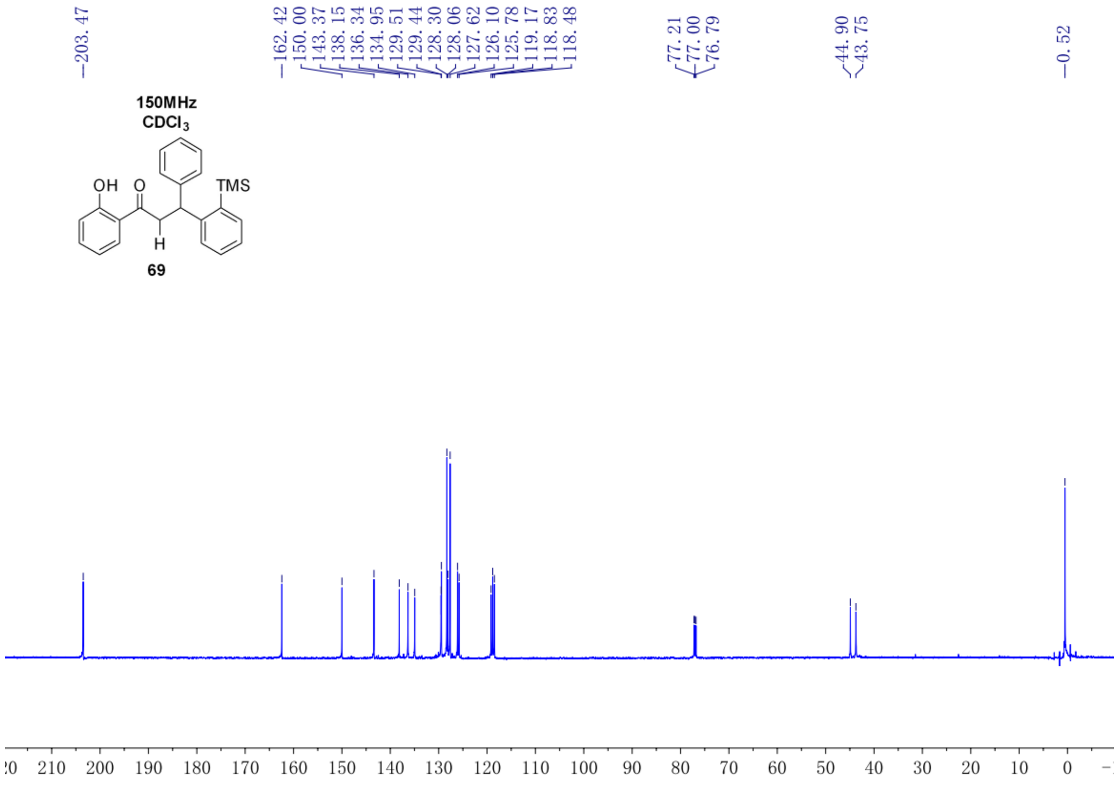

203.47
162.42 150.00 143.37 138.15 136.34 134.95 129.51 129.44 128.30 128.06 127.62 126.10 125.78 119.17 118.83 118.48
77.21 77.00 76.79
44.90 43.75
0.52
150MHz
CDCl3
OH
O
TMS
H
69
210 200 190 180 170 160 150 140 130 120 110 100 90 80 70 60 50 40 30 20 10 0

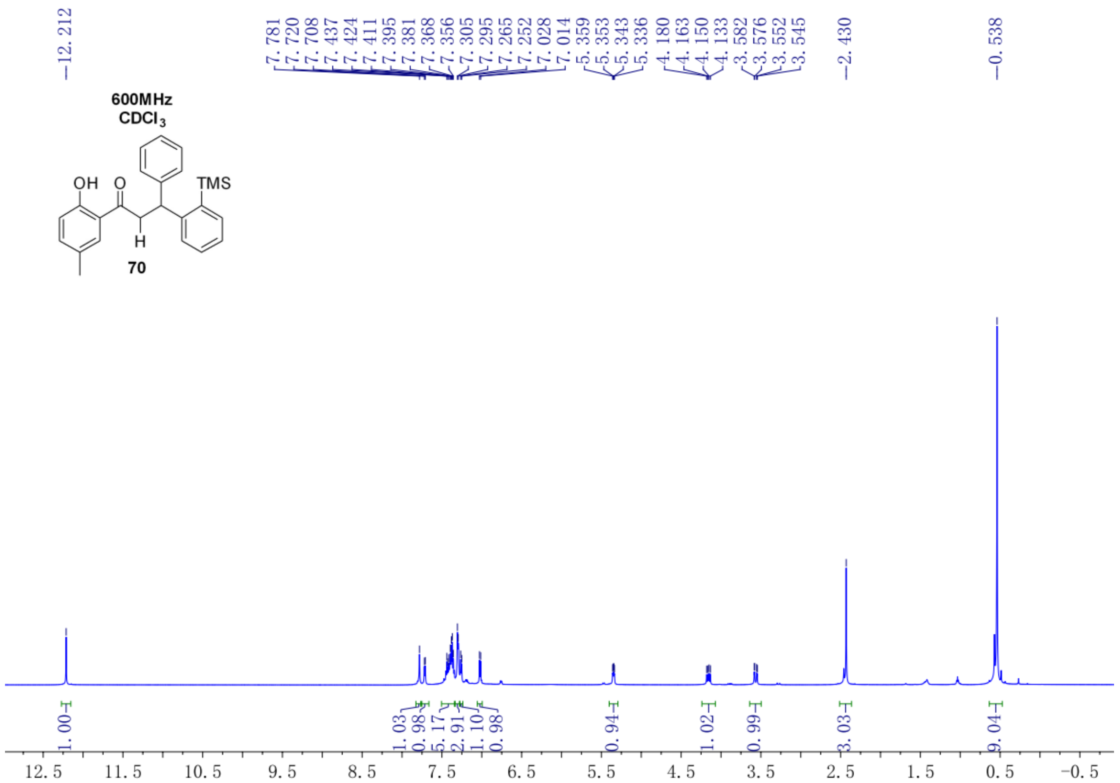
600MHz
CDCl3
OH
O
TMS
H
70
12.212
7.781 7.720 7.708 7.437 7.424 7.411 7.395 7.381 7.368 7.356 7.305 7.295 7.265 7.252 7.028 7.014
5.359 5.353 5.343 5.336
4.180 4.163 4.150 4.133
3.582 3.576 3.552 3.545
2.430
0.538
1.00 1.03 0.98 5.17 2.91 1.10 0.98 0.94 1.02 0.99 3.03 9.04
12.5 11.5 10.5 9.5 8.5 7.5 6.5 5.5 4.5 3.5 2.5 1.5 0.5 -0.5

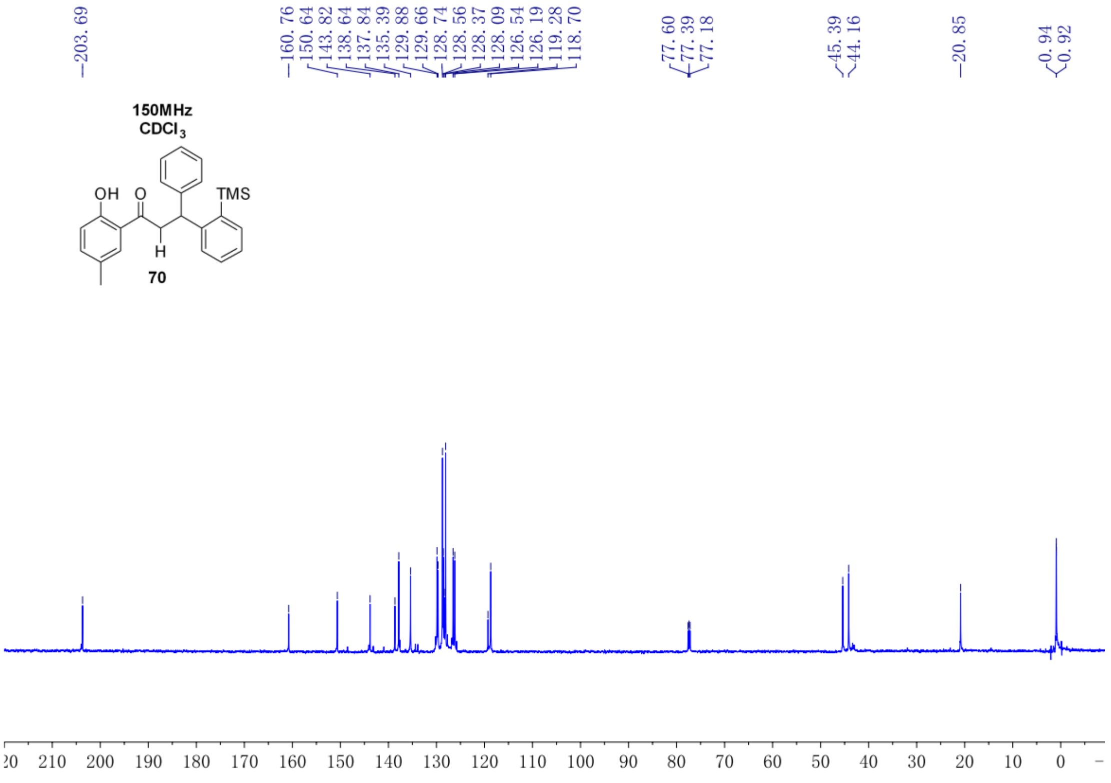
150MHz
CDCl3
OH
O
TMS
H
70
203.69
160.76 150.64 143.82 138.64 137.84 135.39 129.88 129.66 128.74 128.56 128.37 128.09 126.54 126.19 119.28 118.70
77.60 77.39 77.18
45.39 44.16
20.85
0.94 0.92
210 200 190 180 170 160 150 140 130 120 110 100 90 80 70 60 50 40 30 20 10 0

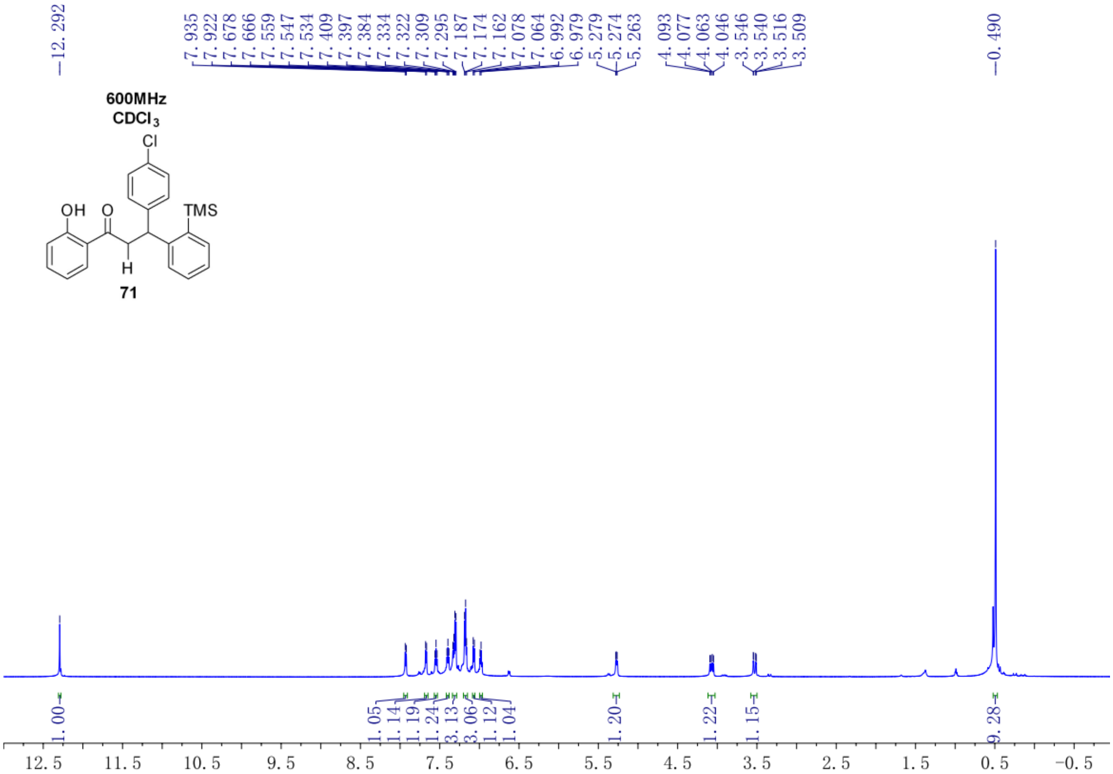
12.292
7.935
7.922
7.678
7.666
7.559
7.547
7.534
7.409
7.397
7.384
7.334
7.322
7.309
7.295
7.187
7.174
7.162
7.078
7.064
6.992
6.979
5.279
5.274
5.263
4.093
4.077
4.063
4.046
3.546
3.540
3.516
3.509
0.490
600MHz
CDCl3
Cl
OH
O
TMS
H
71
1.00
1.05
1.14
1.19
1.24
3.13
3.06
1.12
1.04
1.20
1.22
1.15
9.28
12.5
11.5
10.5
9.5
8.5
7.5
6.5
5.5
4.5
3.5
2.5
1.5
0.5
-0.5

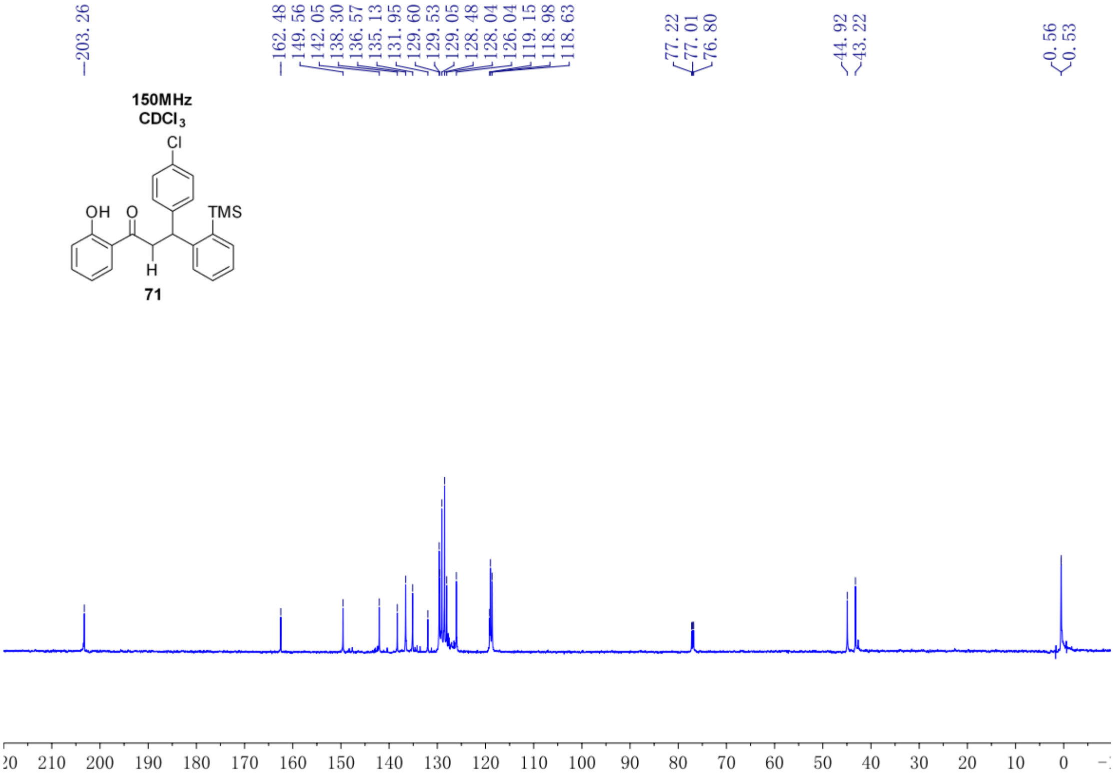
203.26
162.48
149.56
142.05
138.30
136.57
135.13
131.95
129.60
129.53
129.05
128.48
128.04
126.04
119.15
118.98
118.63
77.22
77.01
76.80
44.92
43.22
0.56
0.53
150MHz
CDCl3
Cl
OH
O
TMS
H
71
210
200
190
180
170
160
150
140
130
120
110
100
90
80
70
60
50
40
30
20
10
0

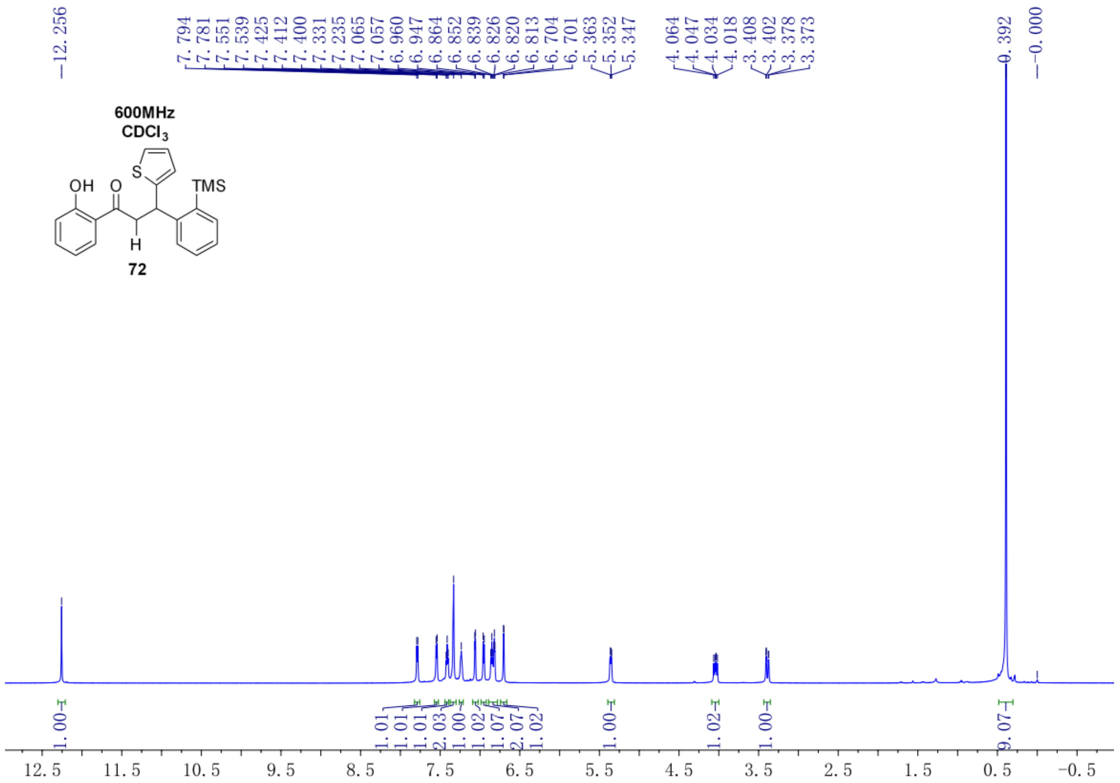
12.256
7.794
7.781
7.551
7.539
7.425
7.412
7.400
7.331
7.235
7.065
7.057
6.960
6.947
6.864
6.852
6.839
6.826
6.820
6.813
6.704
6.701
5.363
5.352
5.347
4.064
4.047
4.034
4.018
3.408
3.402
3.378
3.373
0.392
-0.000
600MHz
CDCl3
OH
O
S
TMS
H
72
1.00
1.01
1.01
1.01
2.03
1.00
1.02
1.07
2.07
1.02
1.00
1.02
1.00
9.07
12.5
11.5
10.5
9.5
8.5
7.5
6.5
5.5
4.5
3.5
2.5
1.5
0.5
-0.5

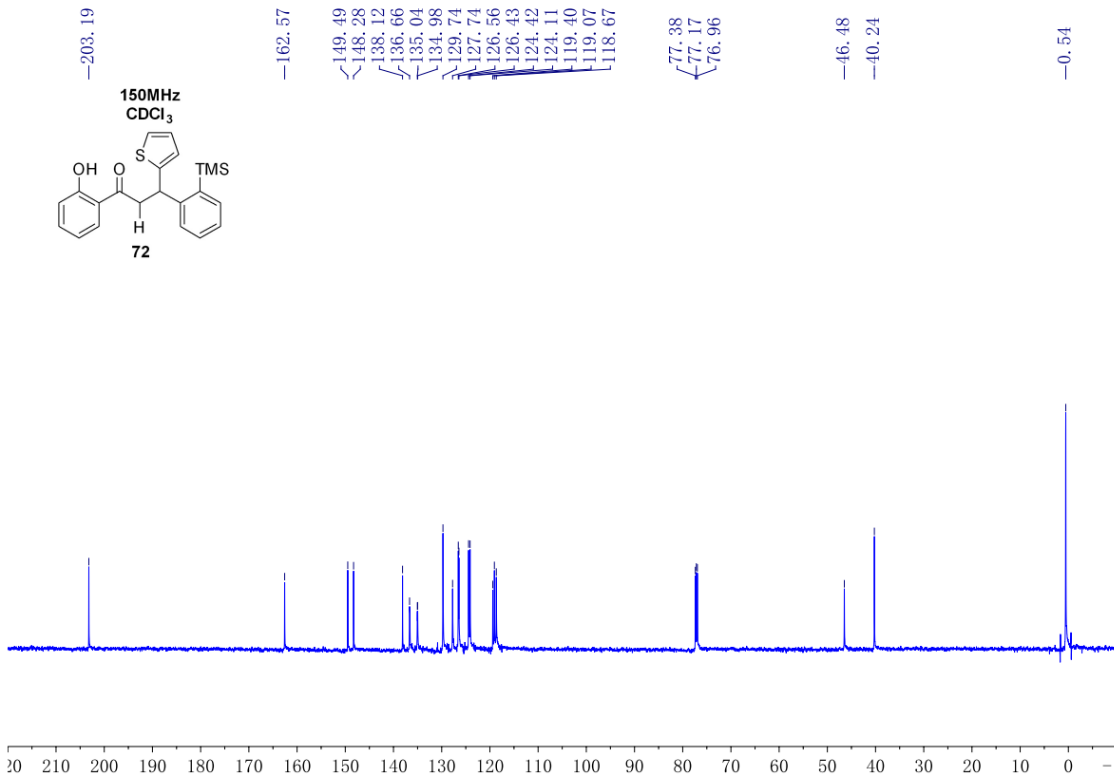
203.19
162.57
149.49
148.28
138.12
136.66
135.04
134.98
129.74
127.74
126.56
126.43
124.42
124.11
119.40
119.07
118.67
77.38
77.17
76.96
46.48
40.24
0.54
150MHz
CDCl3
OH
O
S
TMS
H
72
210
200
190
180
170
160
150
140
130
120
110
100
90
80
70
60
50
40
30
20
10
0

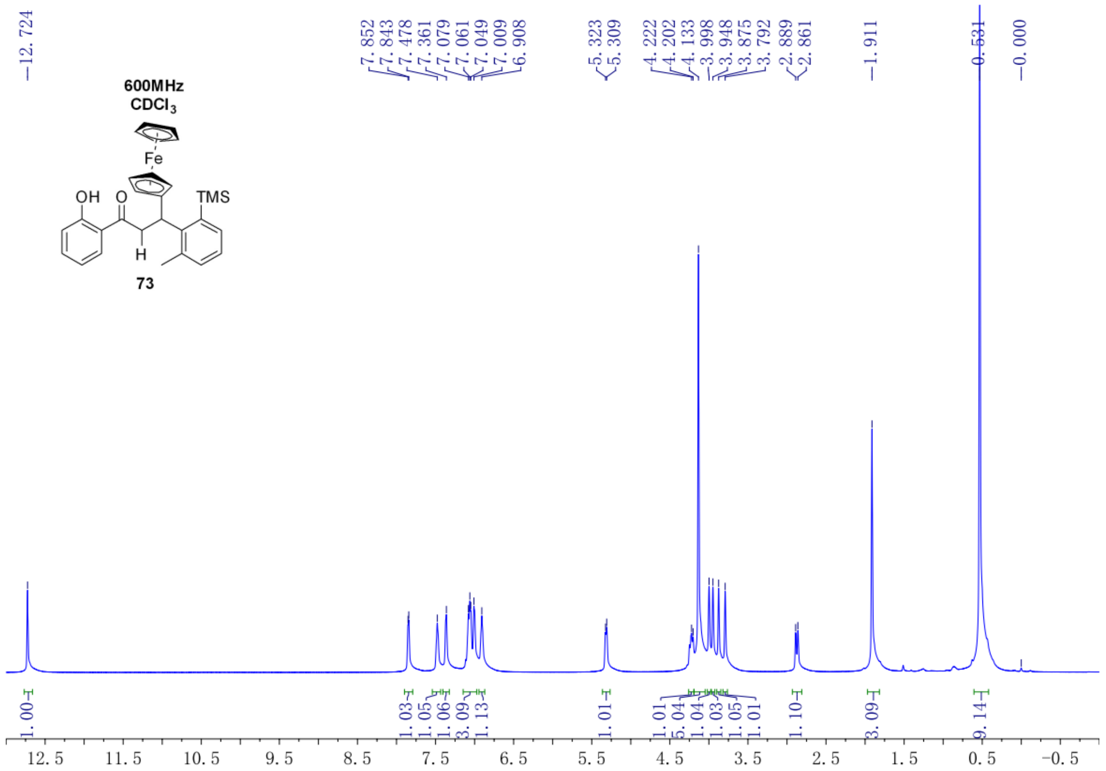
600MHz
CDCl3
Fe
OH
O
TMS
H
73
12.724
7.852 7.843 7.478 7.361 7.079 7.061 7.049 7.009 6.908
5.323 5.309
4.222 4.202 4.133 3.998 3.948 3.875 3.792
2.889 2.861
1.911
0.531
0.000
1.00 1.03 1.05 1.06 3.09 1.13 1.01 1.01 5.04 1.04 1.03 1.05 1.01 1.10 3.09 9.14
12.5 11.5 10.5 9.5 8.5 7.5 6.5 5.5 4.5 3.5 2.5 1.5 0.5 -0.5

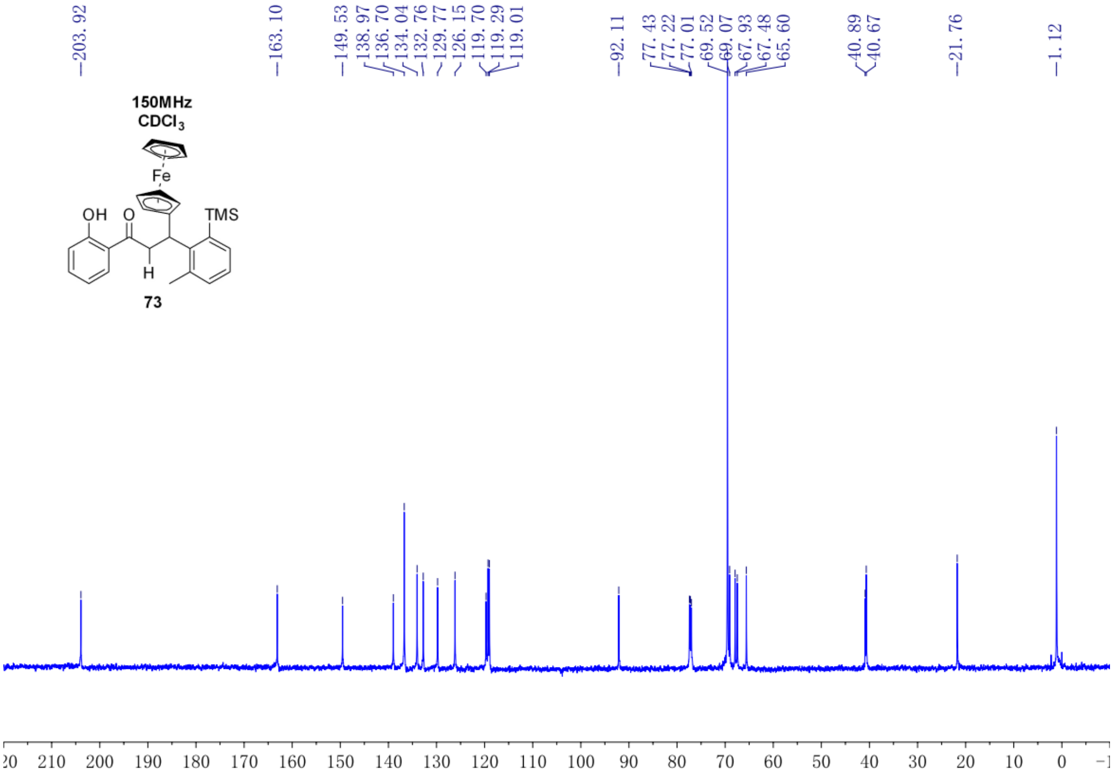
150MHz
CDCl3
Fe
OH
O
TMS
H
73
203.92
163.10
149.53 138.97 136.70 134.04 132.76 129.77 126.15 119.70 119.29 119.01
92.11
77.43 77.22 77.01 69.52 69.07 67.93 67.48 65.60
40.89 40.67
21.76
1.12
210 200 190 180 170 160 150 140 130 120 110 100 90 80 70 60 50 40 30 20 10 0

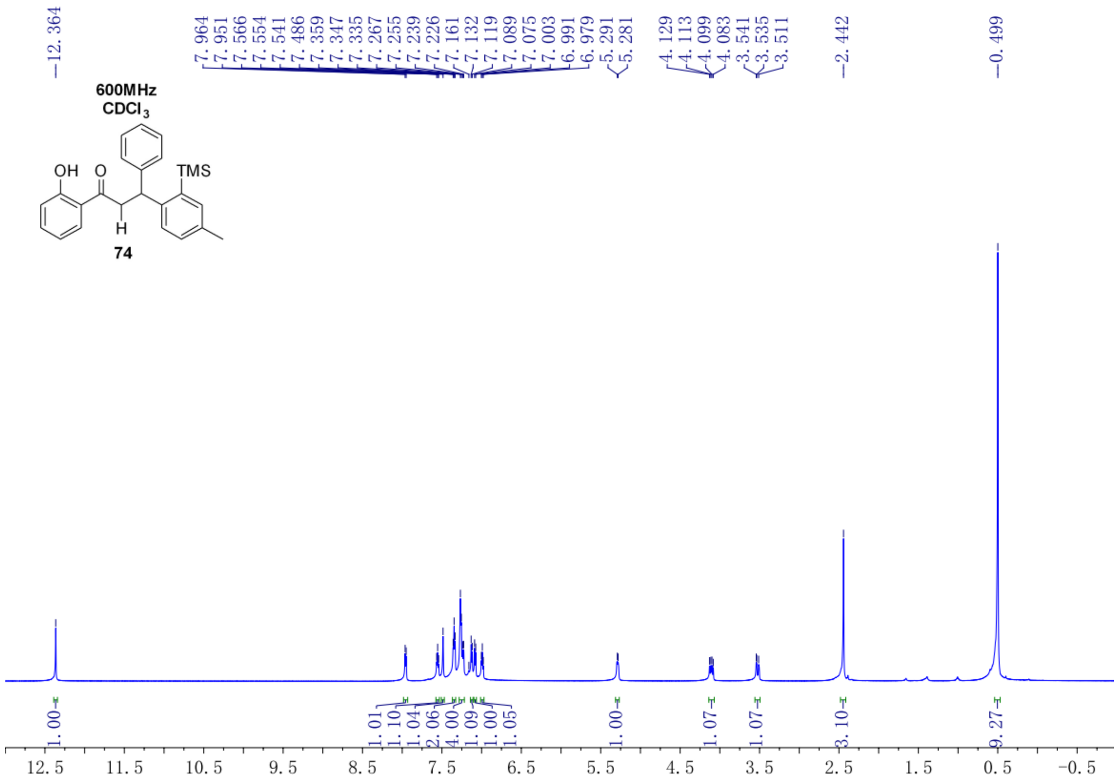
600MHz
CDCl3
OH
O
TMS
H
74

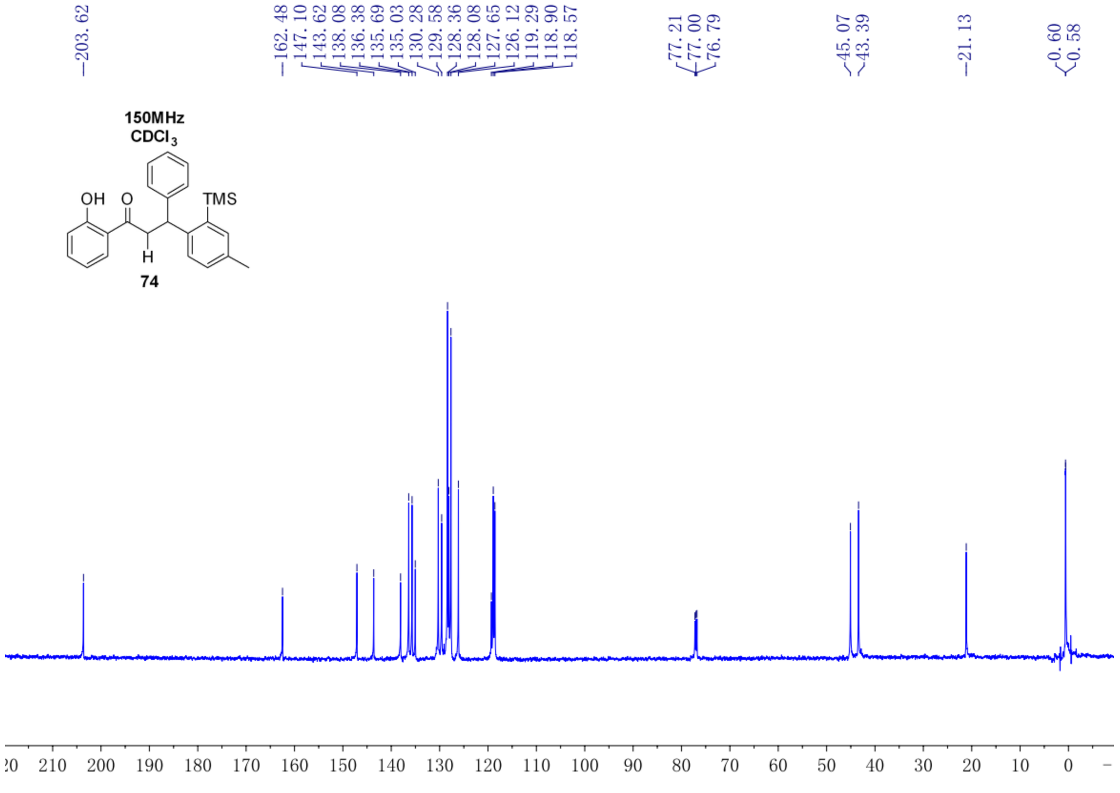
150MHz
CDCl3
OH
O
TMS
H
74

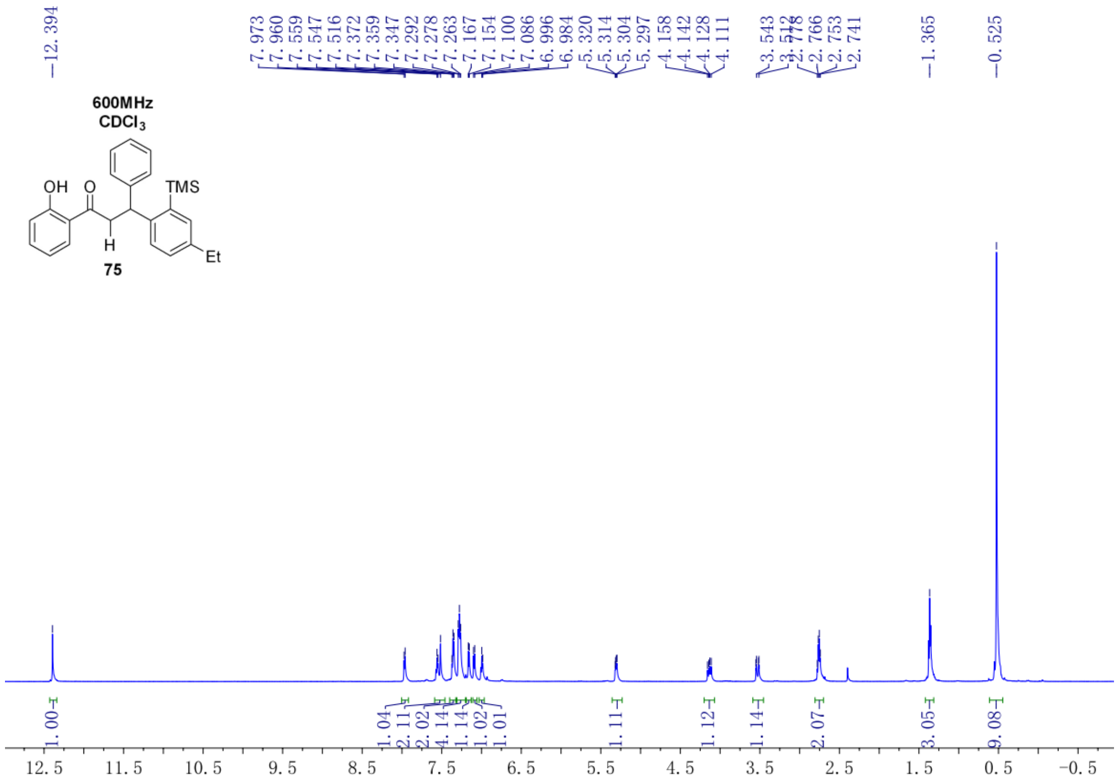
600MHz
CDCl3
OH
O
TMS
H
Et
75
12.394
7.973
7.960
7.559
7.547
7.516
7.372
7.359
7.347
7.292
7.278
7.263
7.167
7.154
7.100
7.086
6.996
6.984
5.320
5.314
5.304
5.297
4.158
4.142
4.128
4.111
3.543
2.766
2.753
2.741
1.365
0.525
12.5
11.5
10.5
9.5
8.5
7.5
6.5
5.5
4.5
3.5
2.5
1.5
0.5
-0.5

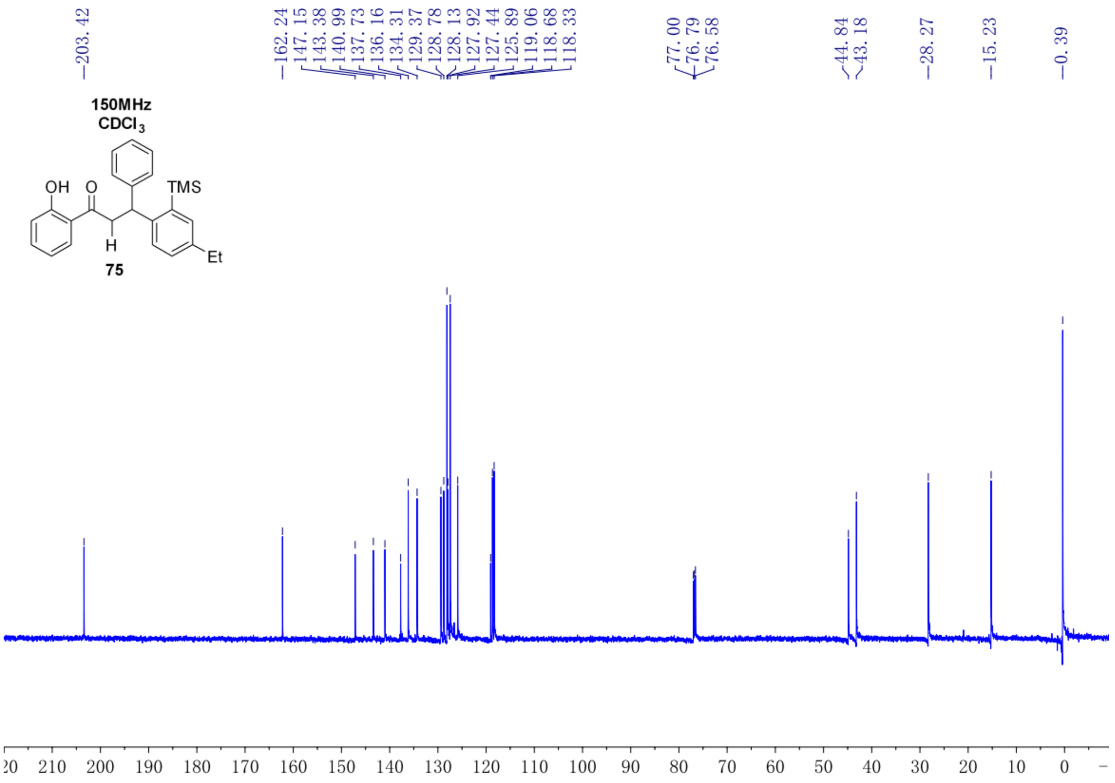
150MHz
CDCl3
OH
O
TMS
H
Et
75
203.42
162.24
147.15
143.38
140.99
137.73
136.16
134.31
129.37
128.78
128.13
127.92
127.44
125.89
119.06
118.68
118.33
77.00
76.79
76.58
44.84
43.18
28.27
15.23
0.39
210
200
190
180
170
160
150
140
130
120
110
100
90
80
70
60
50
40
30
20
10
0

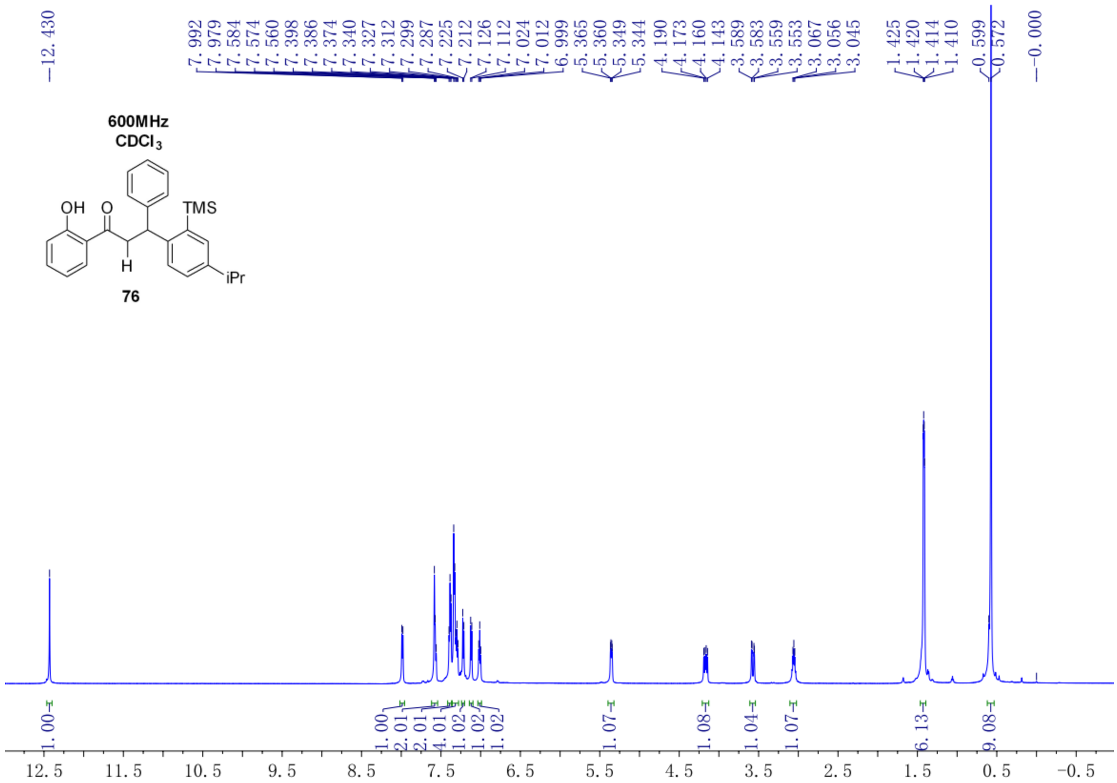
600MHz
CDCl3
OH O
TMS
H
iPr
76
12.430
7.992 7.979 7.584 7.574 7.560 7.398 7.386 7.374 7.340 7.327 7.312 7.299 7.287 7.225 7.212 7.126 7.112 7.024 7.012 6.999
5.365 5.360 5.349 5.344
4.190 4.173 4.160 4.143
3.589 3.583 3.559 3.553
3.067 3.056 3.045
1.425 1.420 1.414 1.410
0.599 0.572
0.000
1.00
1.00 2.01 2.01 4.01 1.02 1.02 1.02
1.07
1.08
1.04
1.07
6.13
9.08
12.5 11.5 10.5 9.5 8.5 7.5 6.5 5.5 4.5 3.5 2.5 1.5 0.5 -0.5

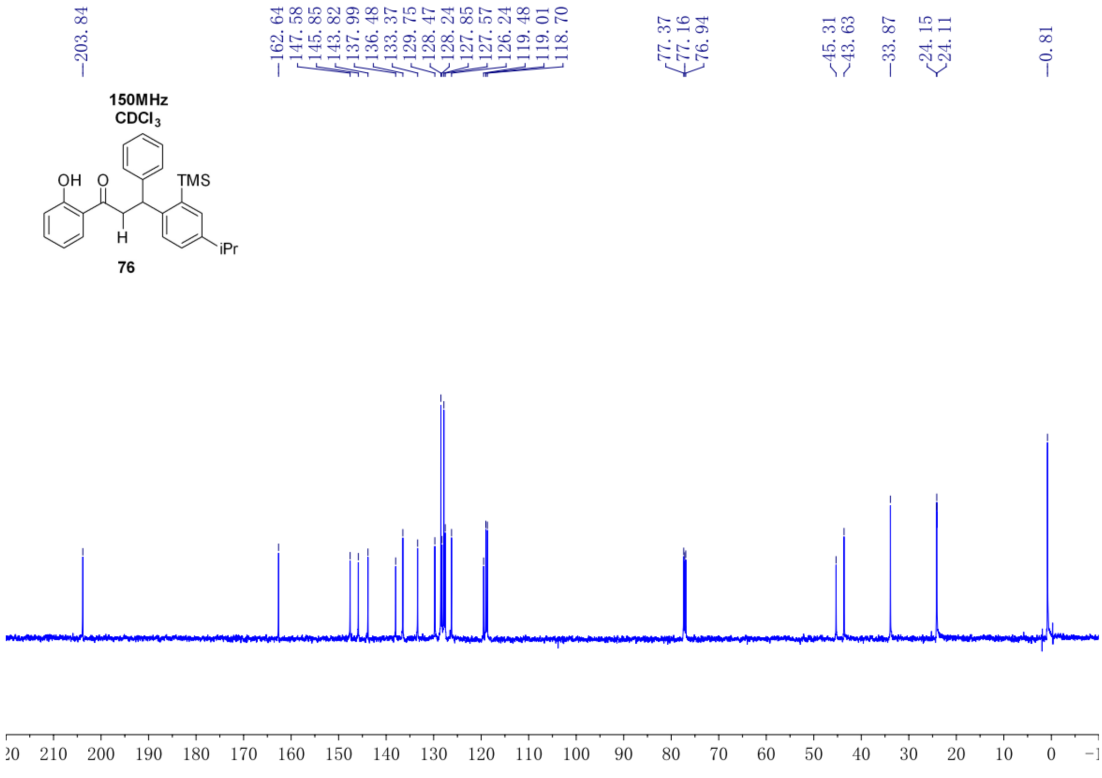
150MHz
CDCl3
OH O
TMS
H
iPr
76
203.84
162.64 147.58 145.85 143.82 137.99 136.48 133.37 129.75 128.47 128.24 127.85 127.57 126.24 119.48 119.01 118.70
77.37 77.16 76.94
45.31 43.63
33.87
24.15 24.11
0.81
210 200 190 180 170 160 150 140 130 120 110 100 90 80 70 60 50 40 30 20 10 0

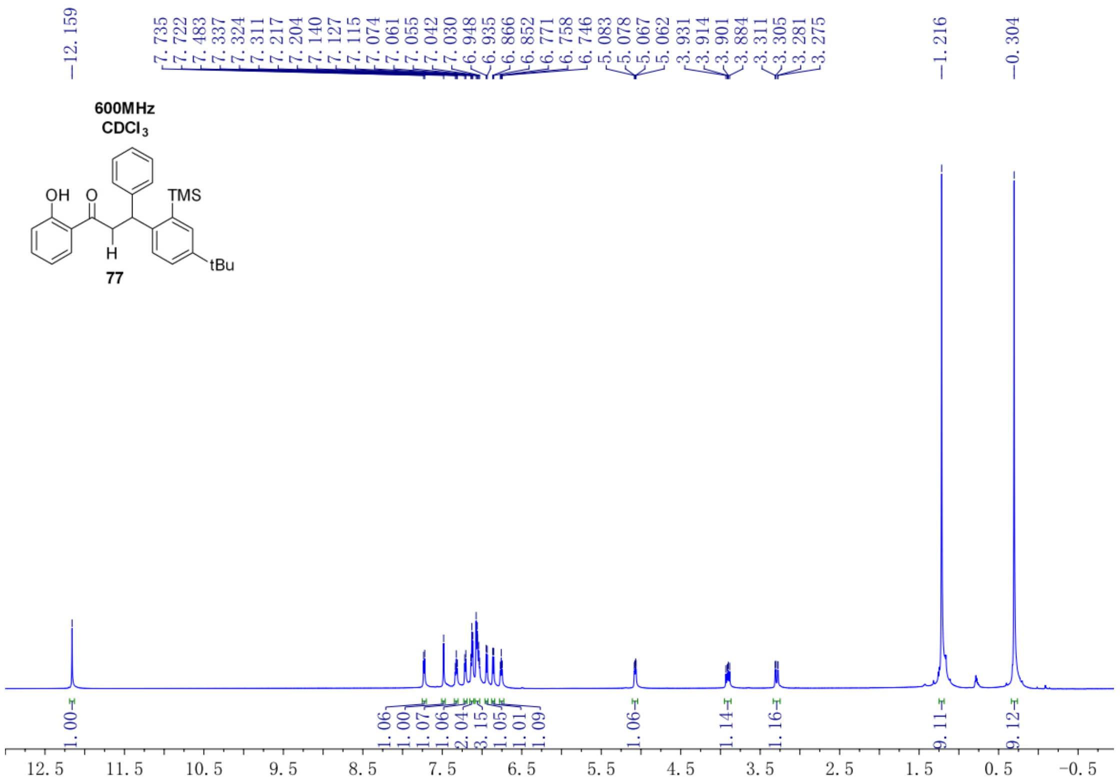
600MHz
CDCl3
OH O
TMS
H
tBu
77
12.159
7.735 7.722 7.483 7.337 7.324 7.311 7.217 7.204 7.140 7.127 7.115 7.074 7.061 7.055 7.042 7.030 6.948 6.935 6.866 6.852 6.771 6.758 6.746
5.083 5.078 5.067 5.062
3.931 3.914 3.901 3.884
3.311 3.305 3.281 3.275
1.216
0.304
1.00 1.06 1.00 1.07 1.06 2.04 3.15 1.05 1.01 1.09 1.06 1.14 1.16 9.11 9.12
12.5 11.5 10.5 9.5 8.5 7.5 6.5 5.5 4.5 3.5 2.5 1.5 0.5 −0.5

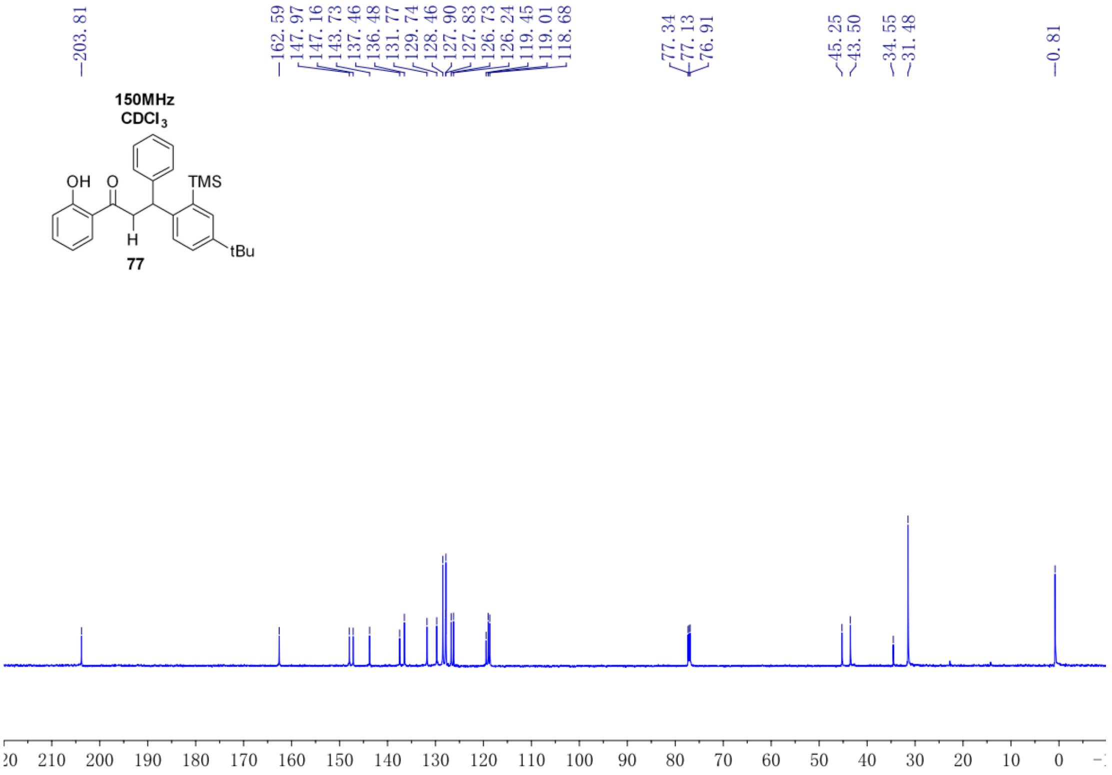
150MHz
CDCl3
OH O
TMS
H
tBu
77
203.81
162.59 147.97 147.16 143.73 137.46 136.48 131.77 129.74 128.46 127.90 127.83 126.73 126.24 119.45 119.01 118.68
77.34 77.13 76.91
45.25 43.50
34.55 31.48
0.81
210 200 190 180 170 160 150 140 130 120 110 100 90 80 70 60 50 40 30 20 10 0

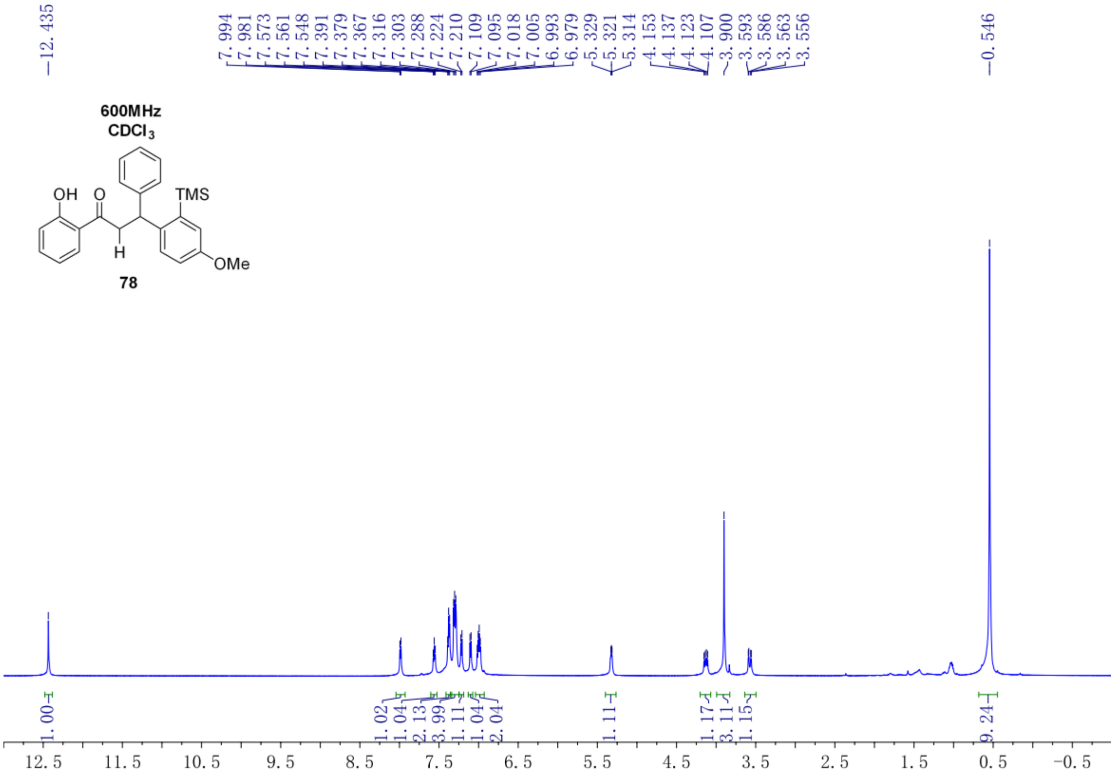
12.435
7.994
7.981
7.573
7.561
7.548
7.391
7.379
7.367
7.316
7.303
7.288
7.224
7.210
7.109
7.095
7.018
7.005
6.993
6.979
5.329
5.321
5.314
4.153
4.137
4.123
4.107
3.900
3.593
3.586
3.563
3.556
0.546
600MHz
CDCl3
OH
O
TMS
H
OMe
78
1.00
1.02
1.04
2.13
3.99
1.11
1.04
2.04
1.11
1.17
3.11
1.15
9.24
12.5
11.5
10.5
9.5
8.5
7.5
6.5
5.5
4.5
3.5
2.5
1.5
0.5
-0.5

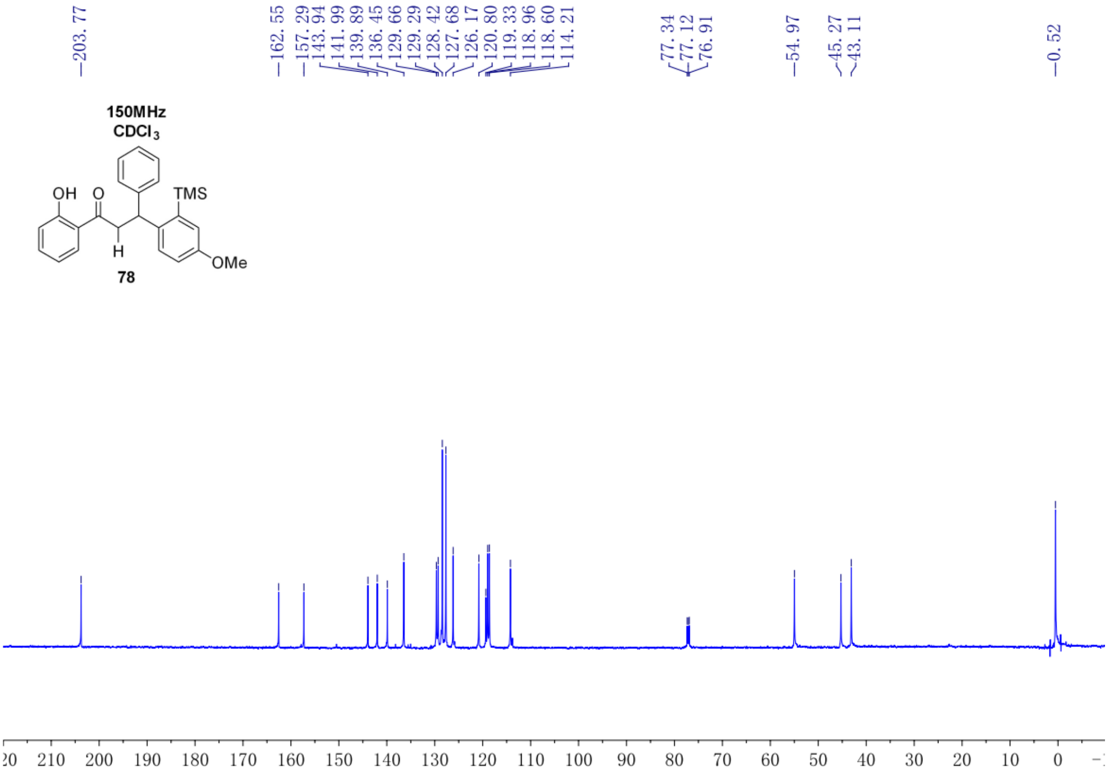
203.77
162.55
157.29
143.94
141.99
139.89
136.45
129.66
129.29
128.42
127.68
126.17
120.80
119.33
118.96
118.60
114.21
77.34
77.12
76.91
54.97
45.27
43.11
0.52
150MHz
CDCl3
OH
O
TMS
H
OMe
78
210
200
190
180
170
160
150
140
130
120
110
100
90
80
70
60
50
40
30
20
10
0

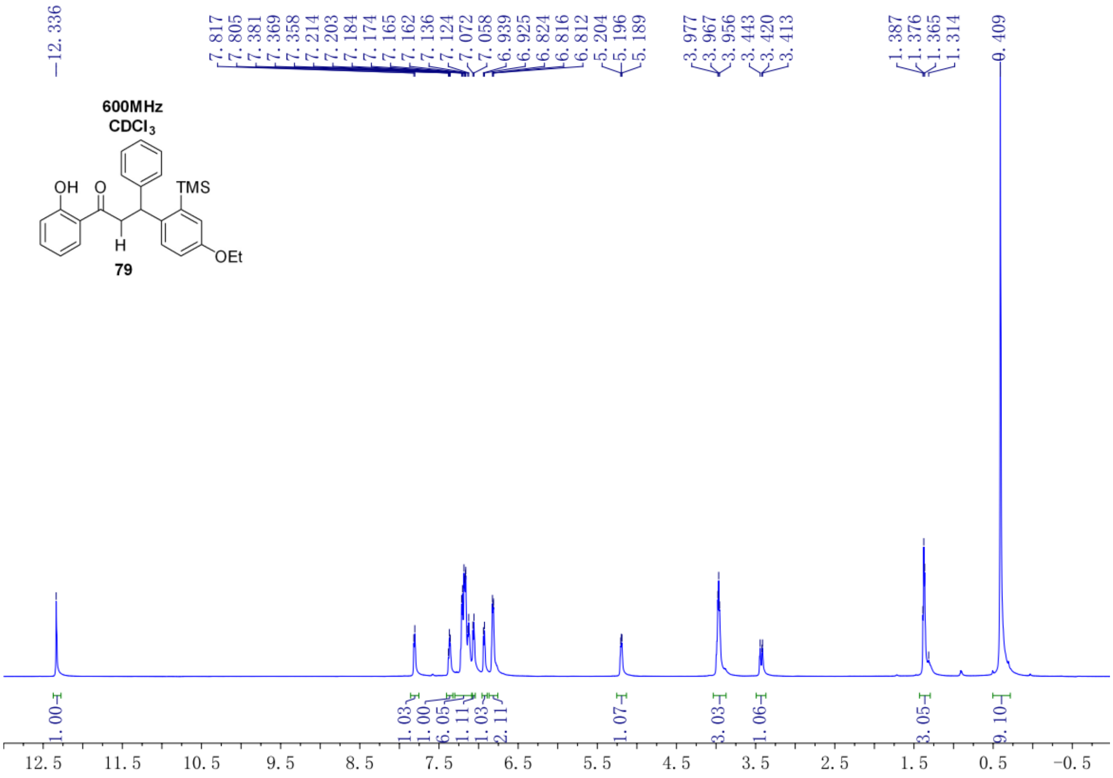
600MHz
$CDCl_3$
OH
O
TMS
H
OEt
79

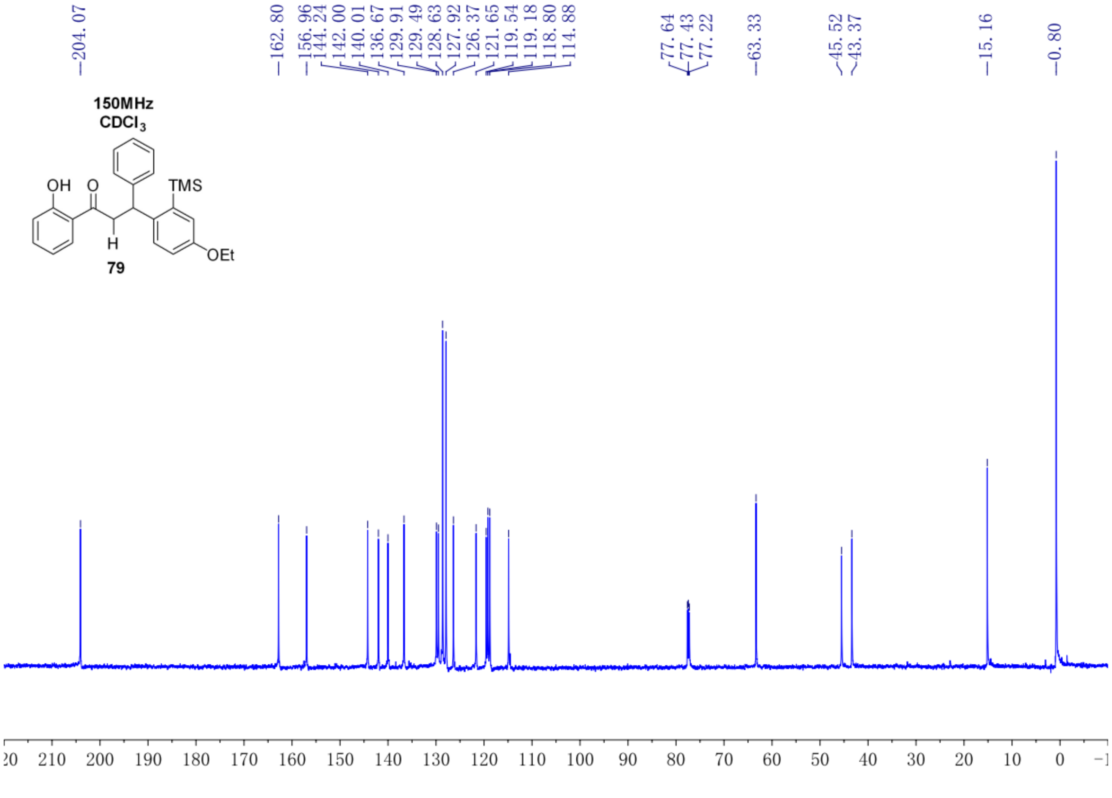
150MHz
$CDCl_3$
OH
O
TMS
H
OEt
79

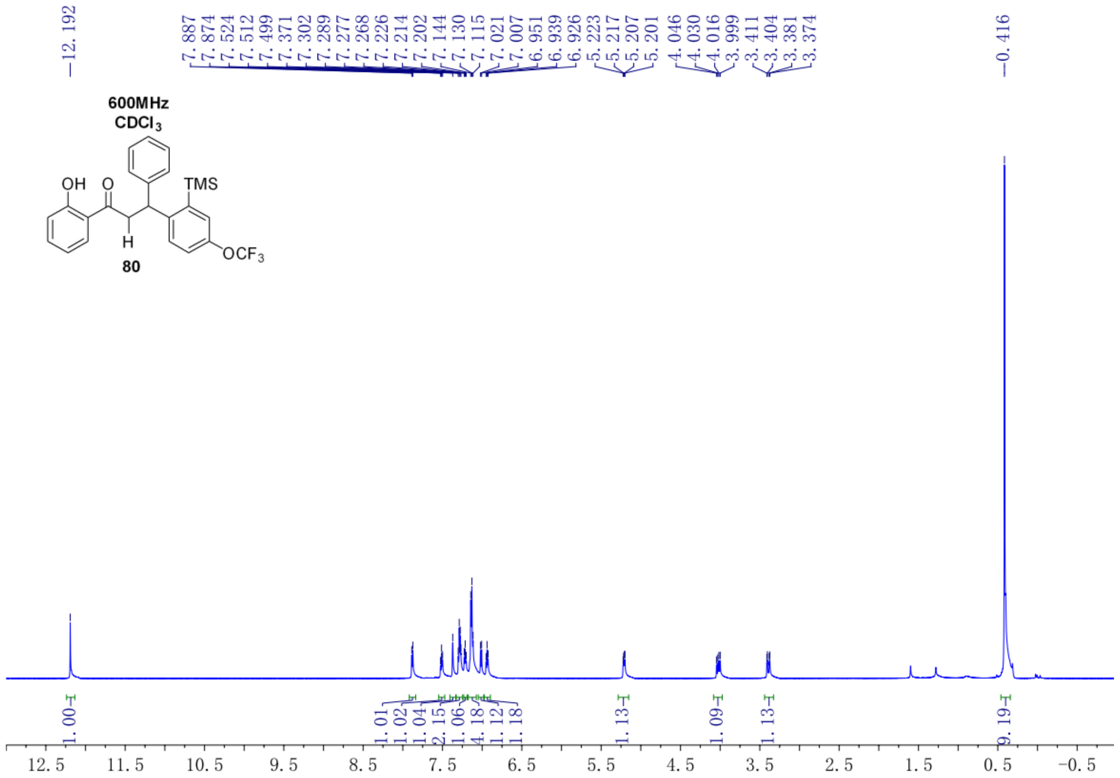
600MHz
CDCl3
OH O
TMS
H
OCF3
80
12.192
7.887 7.874 7.524 7.512 7.499 7.371 7.302 7.289 7.277 7.268 7.226 7.214 7.202 7.144 7.130 7.115 7.021 7.007 6.951 6.939 6.926
5.223 5.217 5.207 5.201
4.046 4.030 4.016 3.999
3.411 3.404 3.381 3.374
0.416
1.00 1.01 1.02 1.04 2.15 1.06 4.18 1.12 1.18 1.13 1.09 1.13 9.19
12.5 11.5 10.5 9.5 8.5 7.5 6.5 5.5 4.5 3.5 2.5 1.5 0.5 -0.5

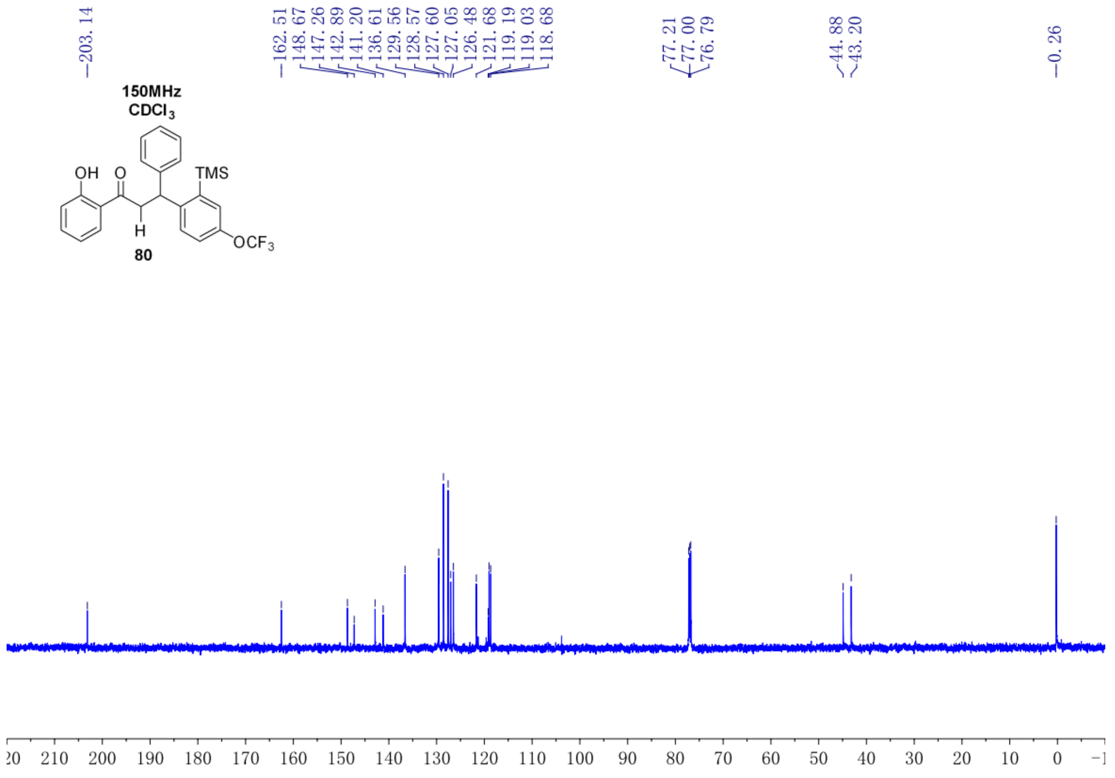
150MHz
CDCl3
OH O
TMS
H
OCF3
80
203.14
162.51 148.67 147.26 142.89 141.20 136.61 129.56 128.57 127.60 127.05 126.48 121.68 119.19 119.03 118.68
77.21 77.00 76.79
44.88 43.20
0.26
210 200 190 180 170 160 150 140 130 120 110 100 90 80 70 60 50 40 30 20 10 0

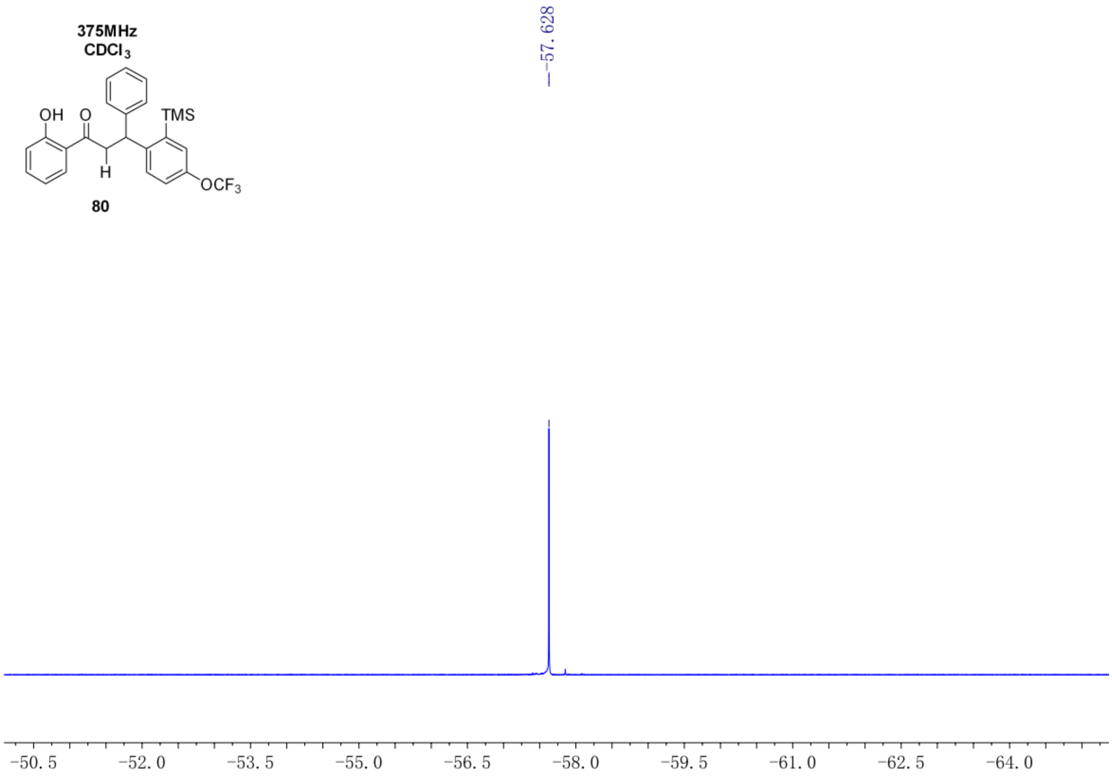
375MHz
CDCl3
OH
O
TMS
H
OCF3
80
-57.628
-50.5
-52.0
-53.5
-55.0
-56.5
-58.0
-59.5
-61.0
-62.5
-64.0

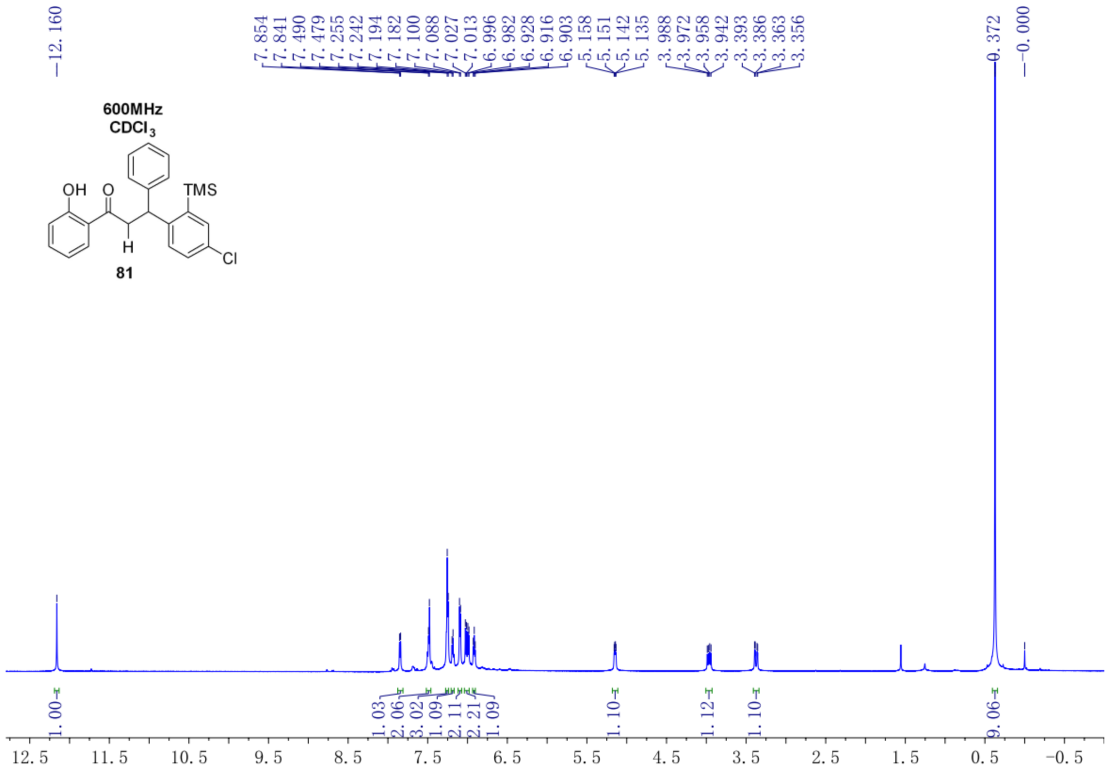
600MHz
CDCl3
OH
O
TMS
H
Cl
81
12.160
7.854
7.841
7.490
7.479
7.255
7.242
7.194
7.182
7.100
7.088
7.027
7.013
6.996
6.982
6.928
6.916
6.903
5.158
5.151
5.142
5.135
3.988
3.972
3.958
3.942
3.393
3.386
3.363
3.356
0.372
-0.000
1.00
1.03
2.06
3.02
1.09
2.11
2.21
1.09
1.10
1.12
1.10
9.06
12.5
11.5
10.5
9.5
8.5
7.5
6.5
5.5
4.5
3.5
2.5
1.5
0.5
-0.5

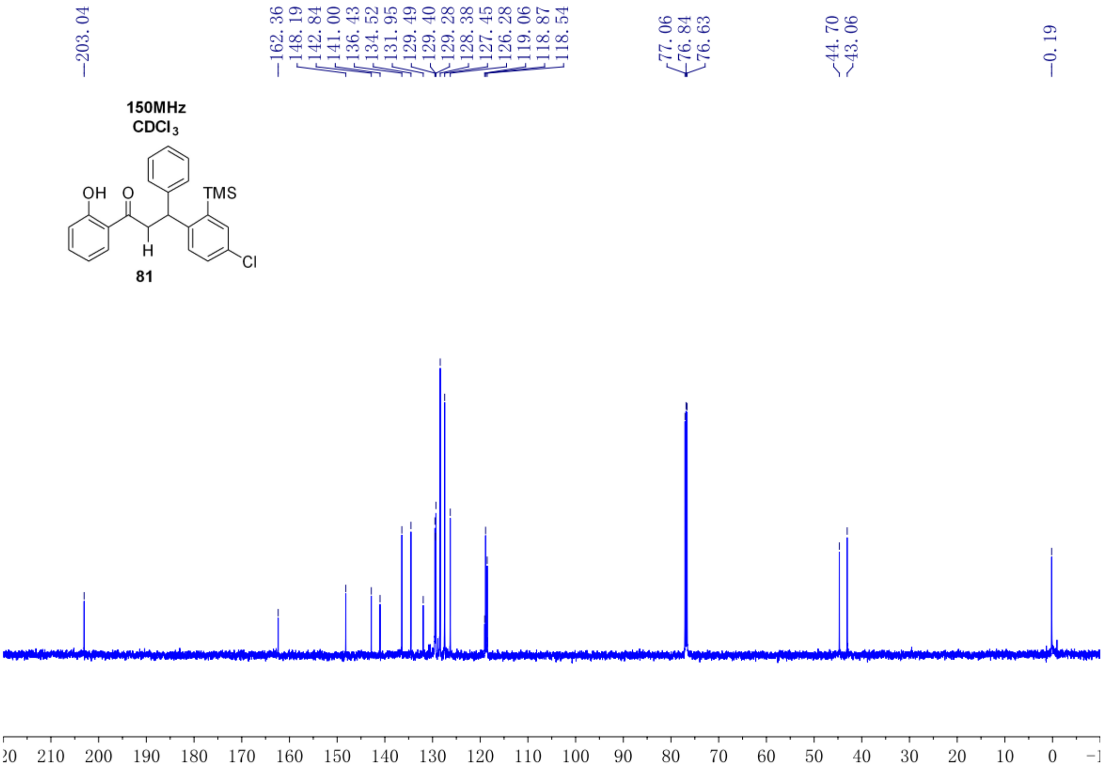

203.04
162.36
148.19
142.84
141.00
136.43
134.52
131.95
129.49
129.40
129.28
128.38
127.45
126.28
119.06
118.87
118.54
77.06
76.84
76.63
44.70
43.06
0.19
150MHz
CDCl3
OH
O
TMS
H
Cl
81

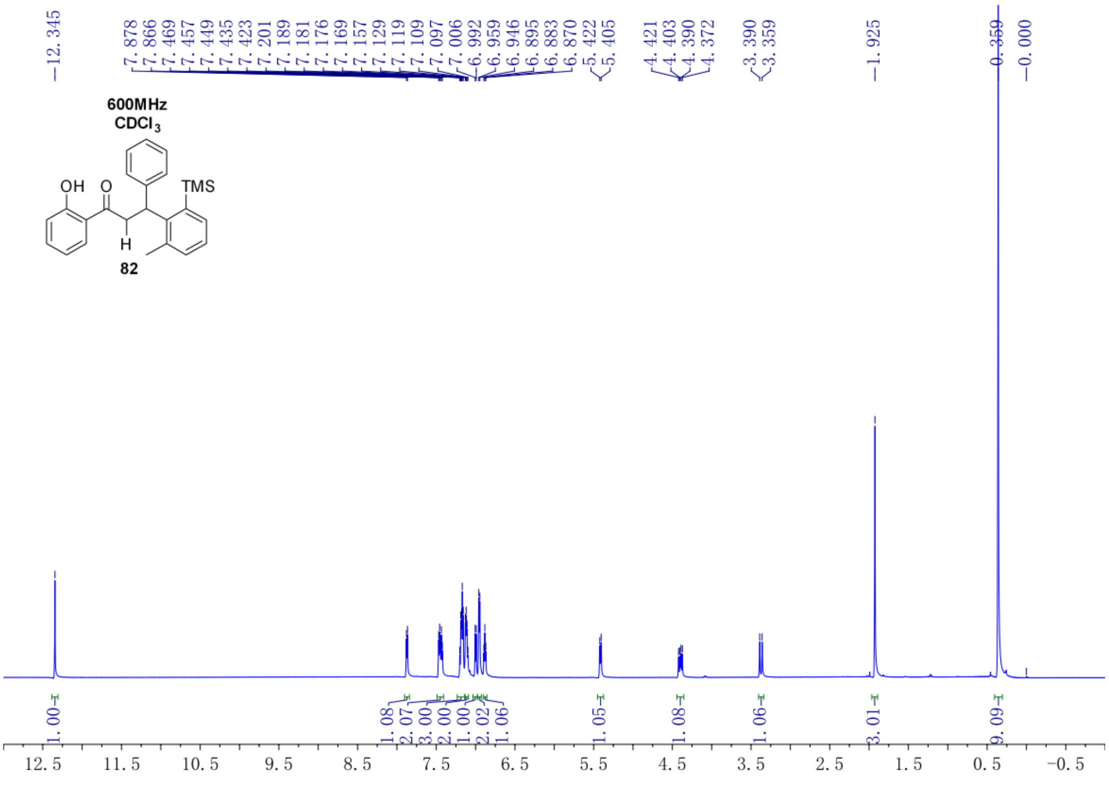

12.345
7.878
7.866
7.469
7.457
7.449
7.435
7.423
7.201
7.189
7.181
7.176
7.169
7.157
7.129
7.119
7.109
7.097
7.006
6.992
6.959
6.946
6.895
6.883
6.870
5.422
5.405
4.421
4.403
4.390
4.372
3.390
3.359
1.925
0.359
0.000
600MHz
CDCl3
OH
O
TMS
H
82
1.00
1.08
2.07
3.00
2.00
1.00
2.02
1.06
1.05
1.08
1.06
3.01
9.09

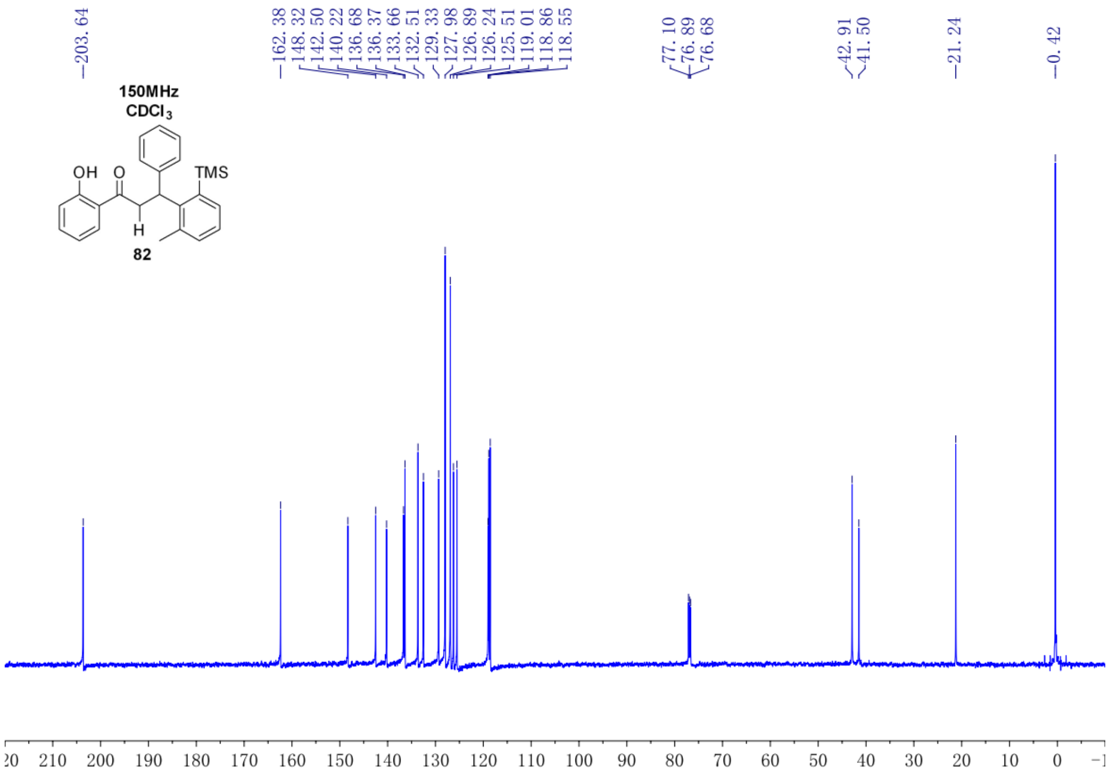

150MHz
CDCl3
OH
O
TMS
H
82
203.64
162.38
148.32
142.50
140.22
136.68
136.37
133.66
132.51
129.33
127.98
126.89
126.24
125.51
119.01
118.86
118.55
77.10
76.89
76.68
42.91
41.50
21.24
0.42

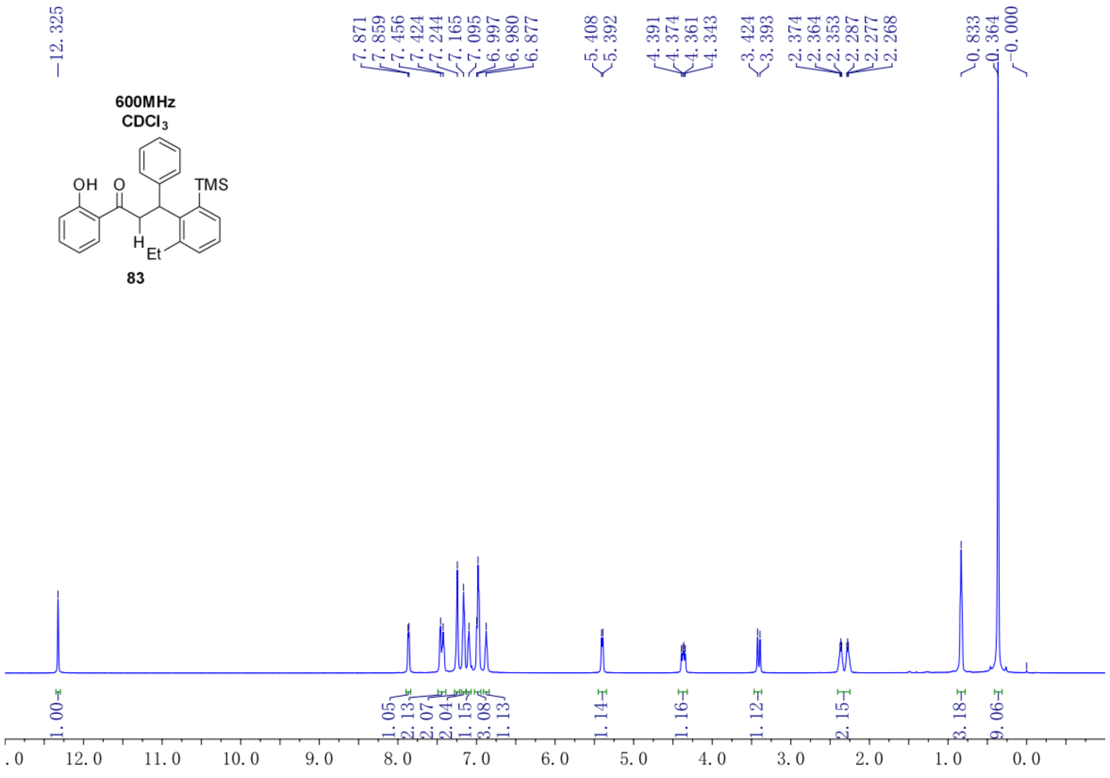

600MHz
CDCl3
OH
O
TMS
H
Et
83
12.325
7.871
7.859
7.456
7.424
7.244
7.165
7.095
6.997
6.980
6.877
5.408
5.392
4.391
4.374
4.361
4.343
3.424
3.393
2.374
2.364
2.353
2.287
2.277
2.268
0.833
0.364
0.000
1.00
1.05
2.13
2.07
2.04
1.15
3.08
1.13
1.14
1.16
1.12
2.15
3.18
9.06

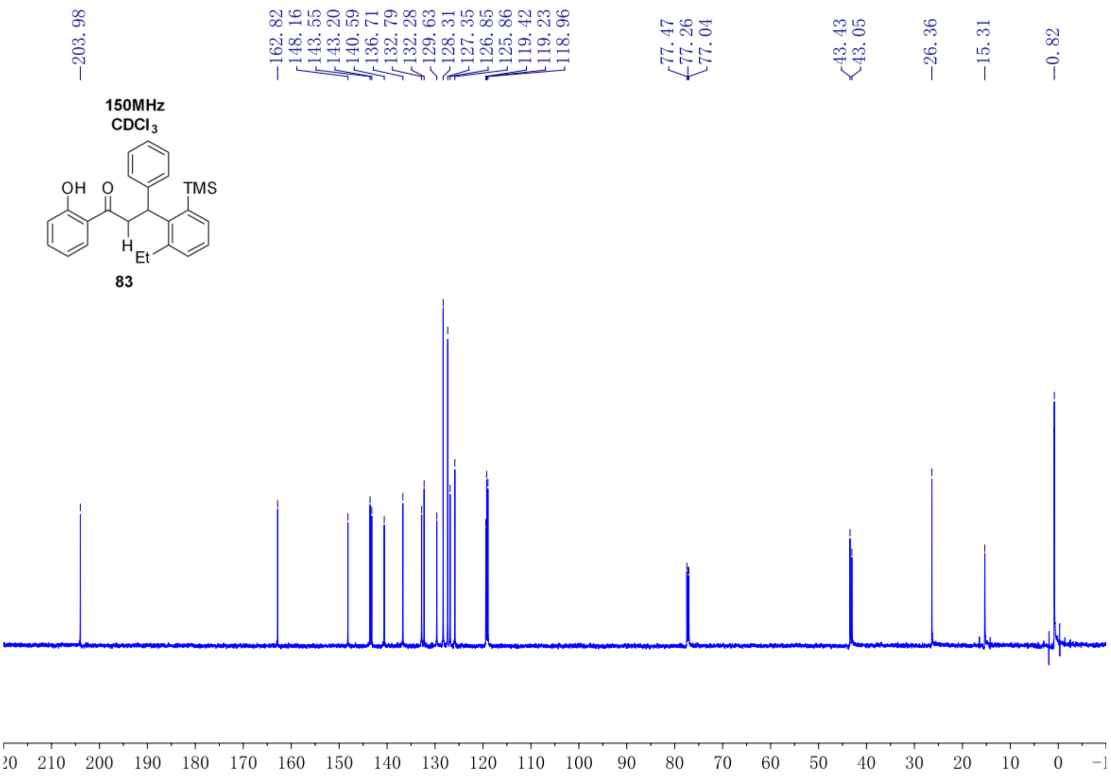
203.98
162.82
148.16
143.55
143.20
140.59
136.71
132.79
132.28
129.63
128.31
127.35
126.85
125.86
119.42
119.23
118.96
77.47
77.26
77.04
43.43
43.05
26.36
15.31
0.82
150MHz
CDCl3
OH
O
TMS
H
Et
83
210 200 190 180 170 160 150 140 130 120 110 100 90 80 70 60 50 40 30 20 10 0

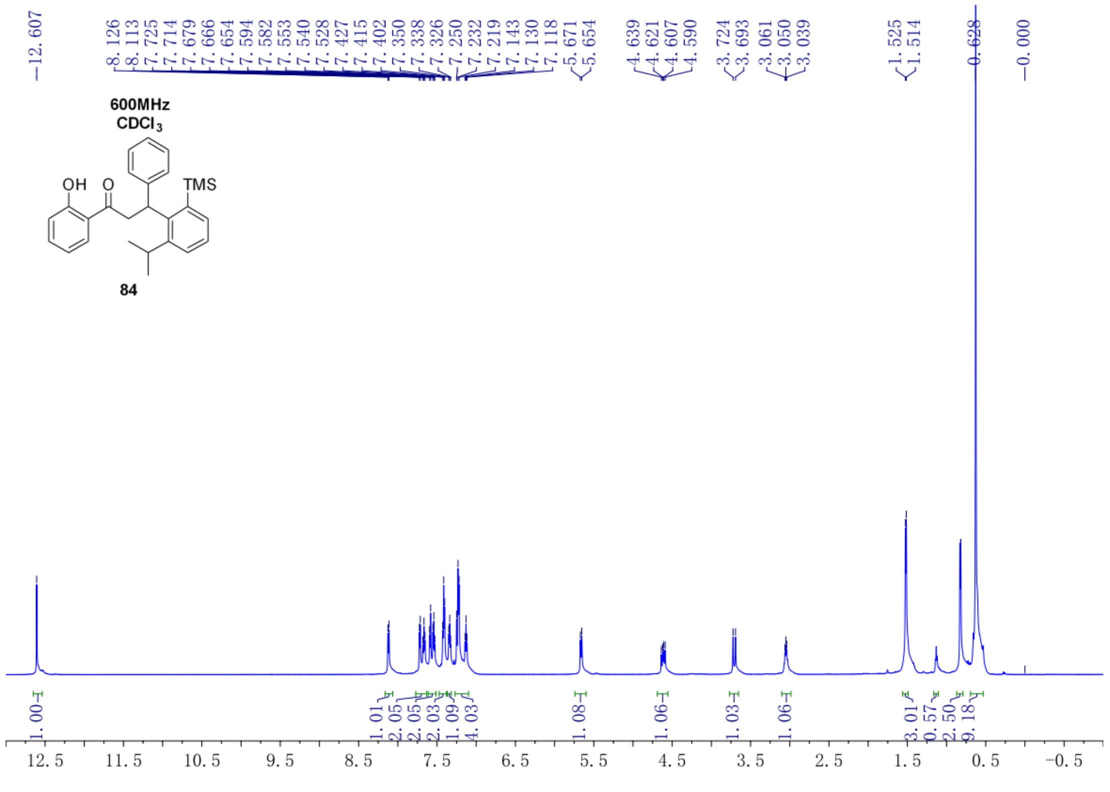
12.607
8.126
8.113
7.725
7.714
7.679
7.666
7.654
7.594
7.582
7.553
7.540
7.528
7.427
7.415
7.402
7.350
7.338
7.326
7.250
7.232
7.219
7.143
7.130
7.118
5.671
5.654
4.639
4.621
4.607
4.590
3.724
3.693
3.061
3.050
3.039
1.525
1.514
0.628
0.000
600MHz
CDCl3
OH
O
TMS
84
1.00
1.01
2.05
2.05
2.03
1.09
4.03
1.08
1.06
1.03
1.06
3.01
0.57
2.50
9.18
12.5 11.5 10.5 9.5 8.5 7.5 6.5 5.5 4.5 3.5 2.5 1.5 0.5 -0.5

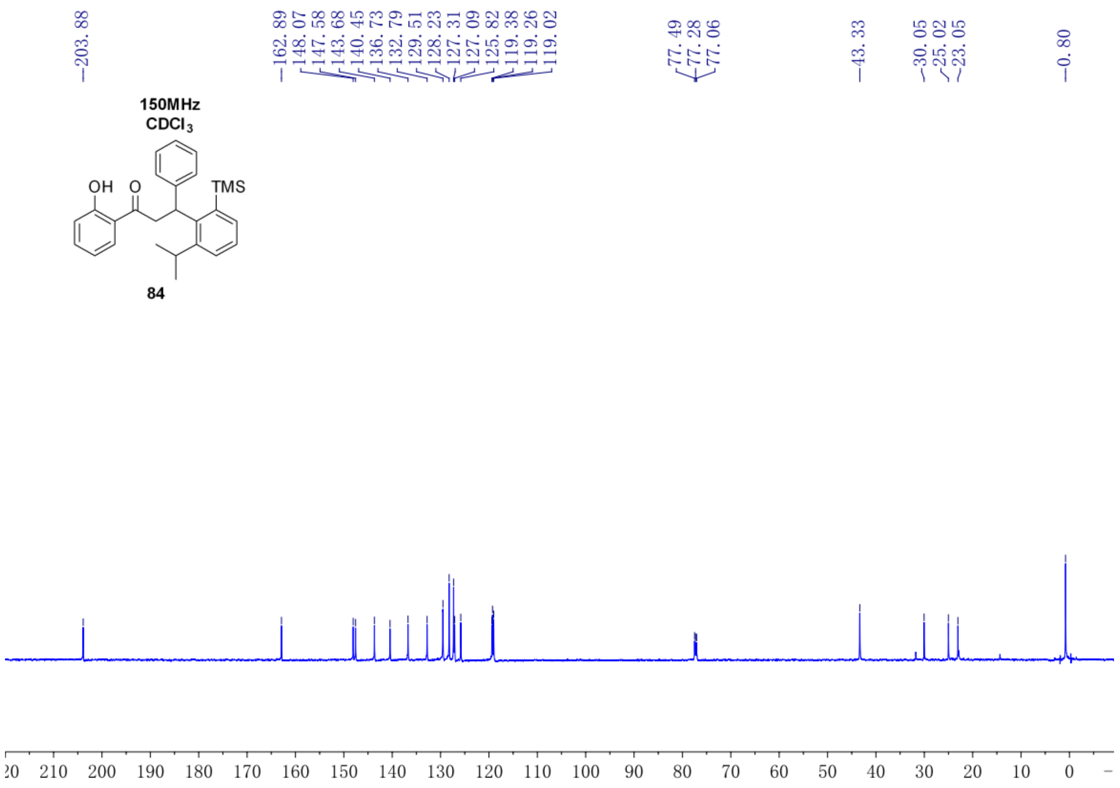
203.88
162.89
148.07
147.58
143.68
140.45
136.73
132.79
129.51
128.23
127.31
127.09
125.82
119.38
119.26
119.02
77.49
77.28
77.06
43.33
30.05
25.02
23.05
0.80
150MHz
CDCl3
OH
O
TMS
84

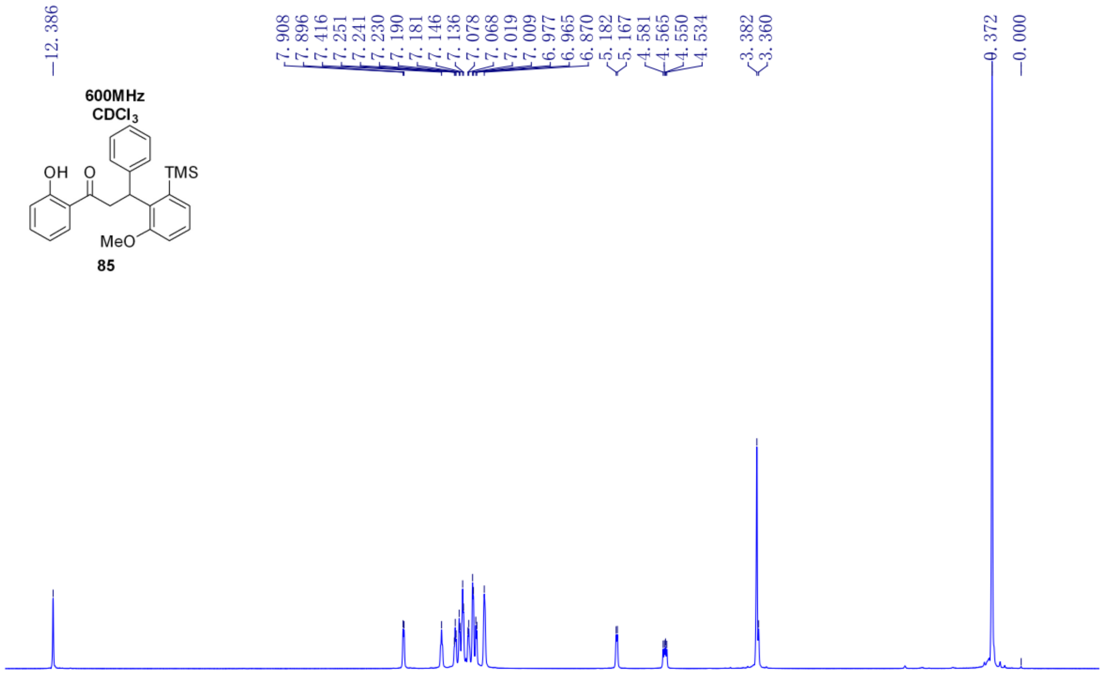
12.386
7.908
7.896
7.416
7.251
7.241
7.230
7.190
7.181
7.146
7.136
7.078
7.068
7.019
7.009
6.977
6.965
6.870
5.182
5.167
4.581
4.565
4.550
4.534
3.382
3.360
0.372
0.000
600MHz
CDCl3
OH
O
TMS
MeO
85

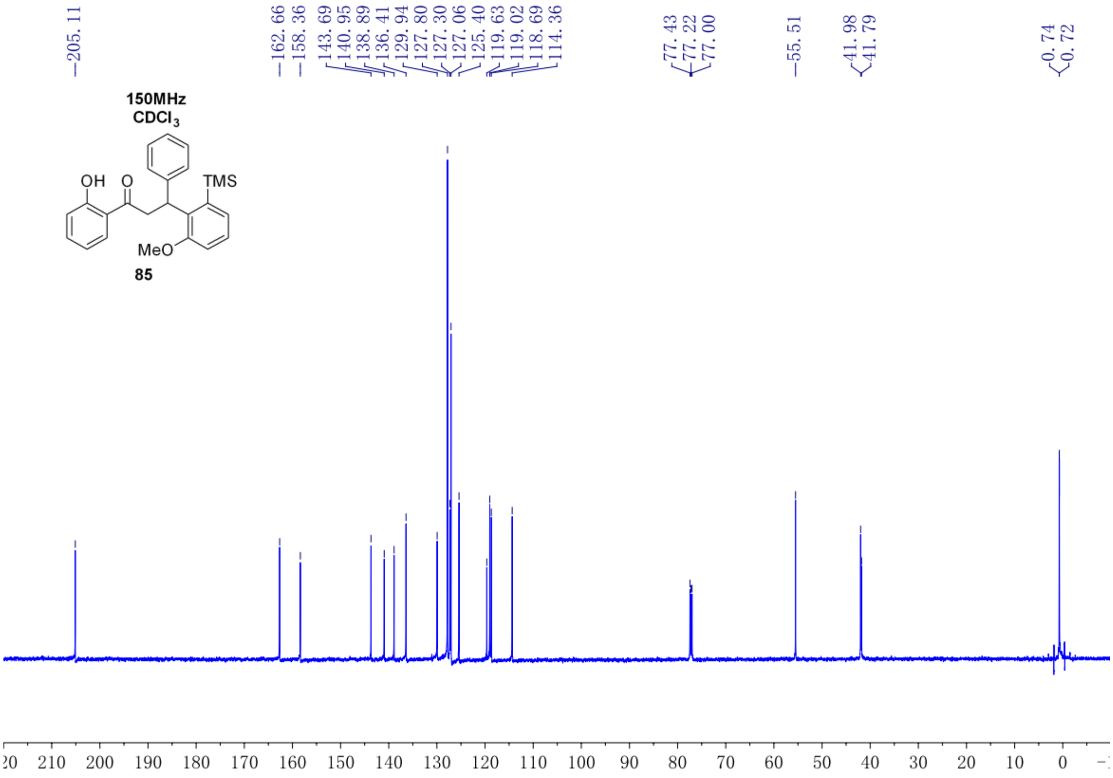
205.11
162.66
158.36
143.69
140.95
138.89
136.41
129.94
127.80
127.30
127.06
125.40
119.63
119.02
118.69
114.36
77.43
77.22
77.00
55.51
41.98
41.79
0.74
0.72
150MHz
CDCl3
OH O
TMS
MeO
85
210 200 190 180 170 160 150 140 130 120 110 100 90 80 70 60 50 40 30 20 10 0

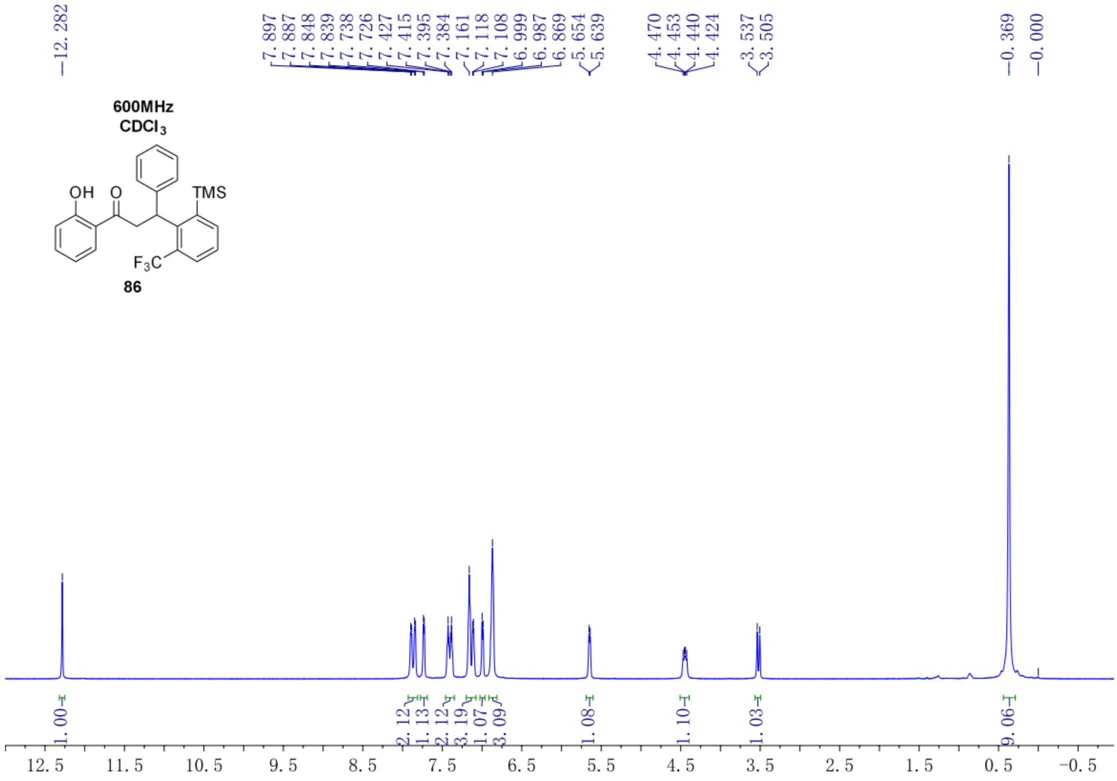
12.282
7.897
7.887
7.848
7.839
7.738
7.726
7.427
7.415
7.395
7.384
7.161
7.118
7.108
6.999
6.987
6.869
5.654
5.639
4.470
4.453
4.440
4.424
3.537
3.505
0.369
0.000
600MHz
CDCl3
OH O
TMS
F3C
86
1.00
2.12
1.13
2.12
3.19
1.07
3.09
1.08
1.10
1.03
9.06
12.5 11.5 10.5 9.5 8.5 7.5 6.5 5.5 4.5 3.5 2.5 1.5 0.5 -0.5

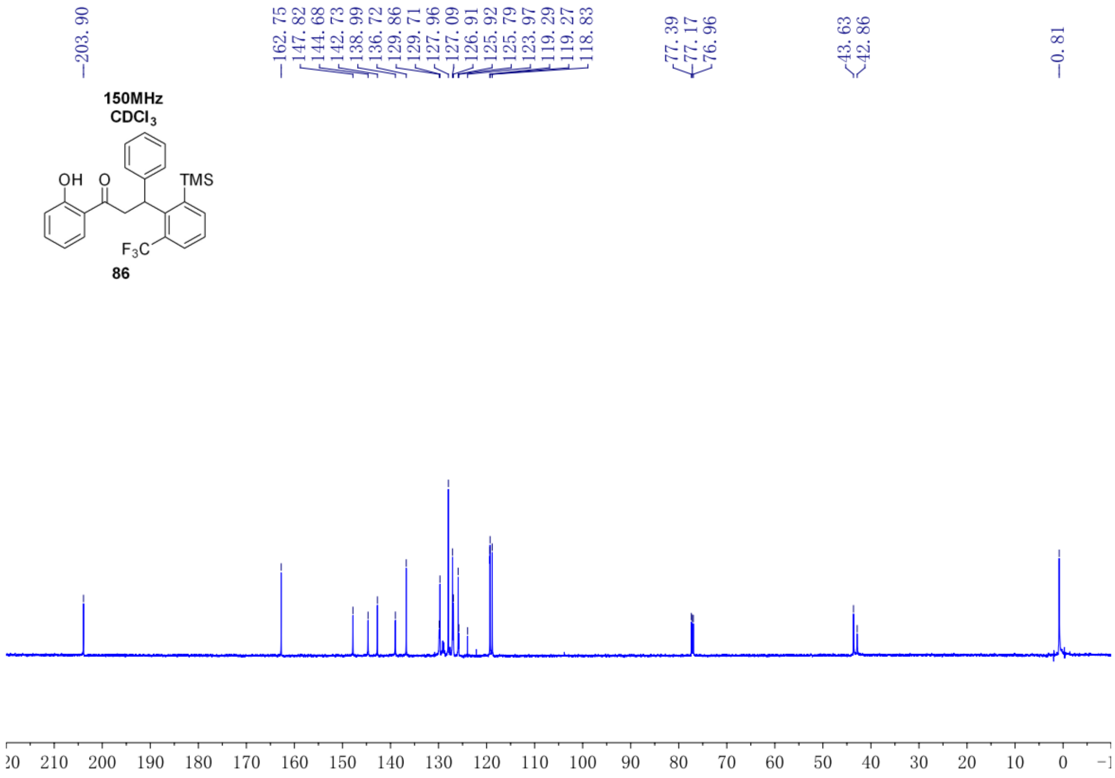
203.90
162.75
147.82
144.68
142.73
138.99
136.72
129.86
129.71
127.96
127.09
126.91
125.92
125.79
123.97
119.29
119.27
118.83
77.39
77.17
76.96
43.63
42.86
0.81
150MHz
CDCl3
OH
O
TMS
F3C
86
210 200 190 180 170 160 150 140 130 120 110 100 90 80 70 60 50 40 30 20 10 0

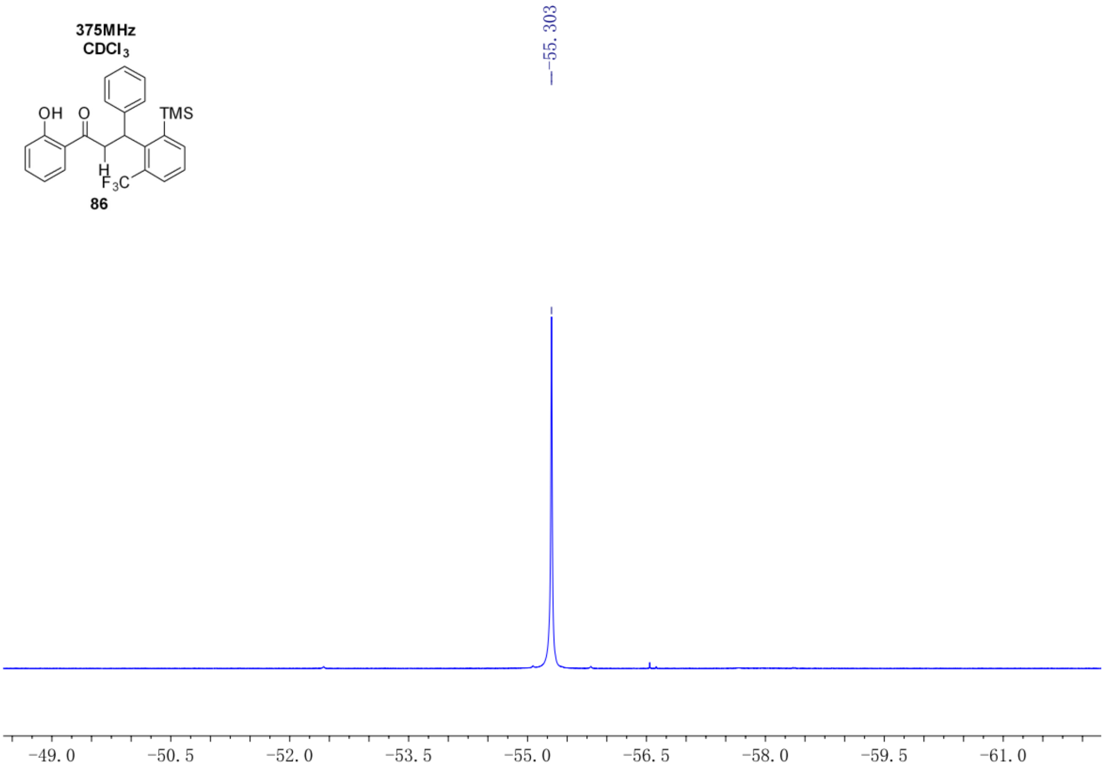
-55.303
375MHz
CDCl3
OH
O
TMS
H
F3C
86
-49.0 -50.5 -52.0 -53.5 -55.0 -56.5 -58.0 -59.5 -61.0

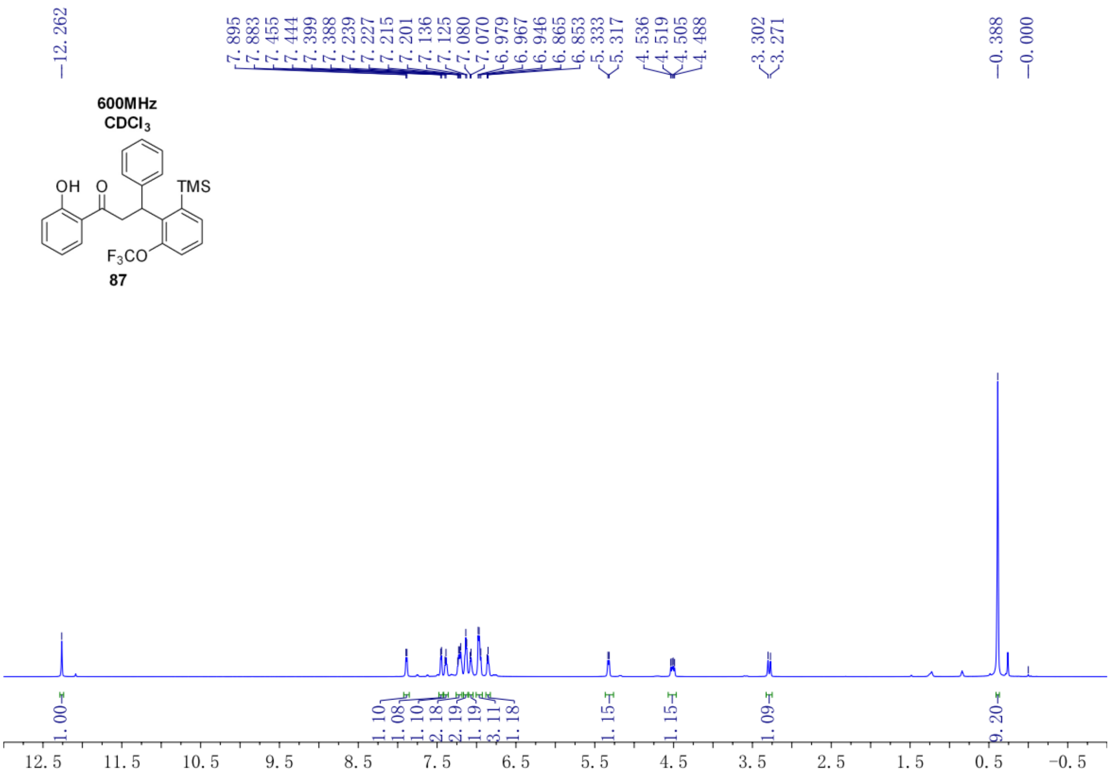
12.262
7.895 7.883 7.455 7.444 7.399 7.388 7.239 7.227 7.215 7.201 7.136 7.125 7.080 7.070 6.979 6.967 6.946 6.865 6.853
5.333 5.317
4.536 4.519 4.505 4.488
3.302 3.271
0.388
0.000
600MHz
CDCl3
OH
O
TMS
F3CO
87
1.00
1.10 1.08 1.10 2.18 2.19 1.19 3.11 1.18
1.15
1.15
1.09
9.20
12.5 11.5 10.5 9.5 8.5 7.5 6.5 5.5 4.5 3.5 2.5 1.5 0.5 -0.5

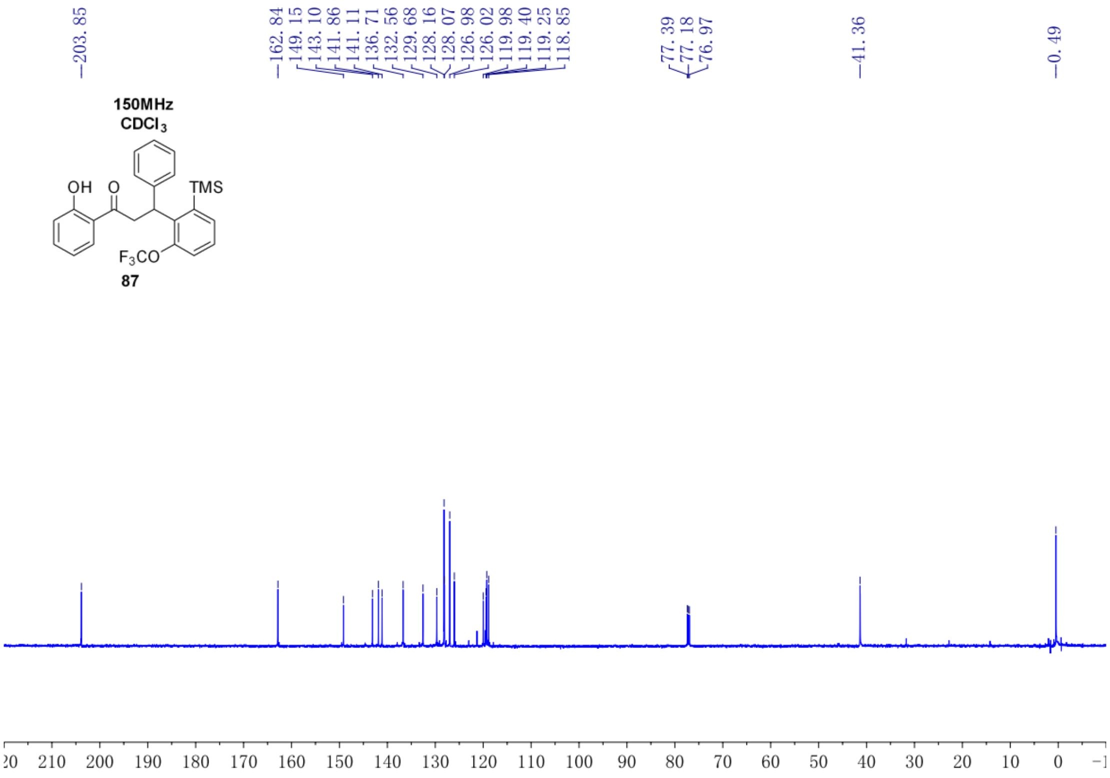
203.85
162.84 149.15 143.10 141.86 141.11 136.71 132.56 129.68 128.16 128.07 126.98 126.02 119.98 119.40 119.25 118.85
77.39 77.18 76.97
41.36
0.49
150MHz
CDCl3
OH
O
TMS
F3CO
87
210 200 190 180 170 160 150 140 130 120 110 100 90 80 70 60 50 40 30 20 10 0

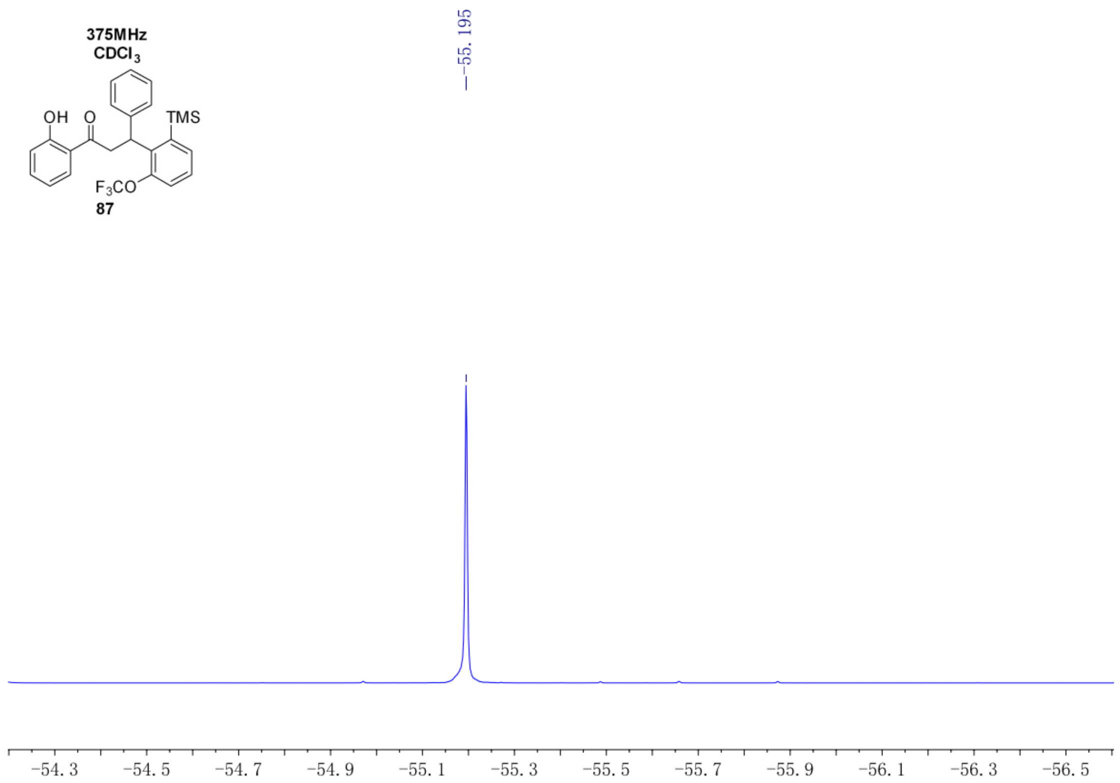
375MHz
CDCl3
OH
O
TMS
F3CO
87
-55.195
-54.3
-54.5
-54.7
-54.9
-55.1
-55.3
-55.5
-55.7
-55.9
-56.1
-56.3
-56.5

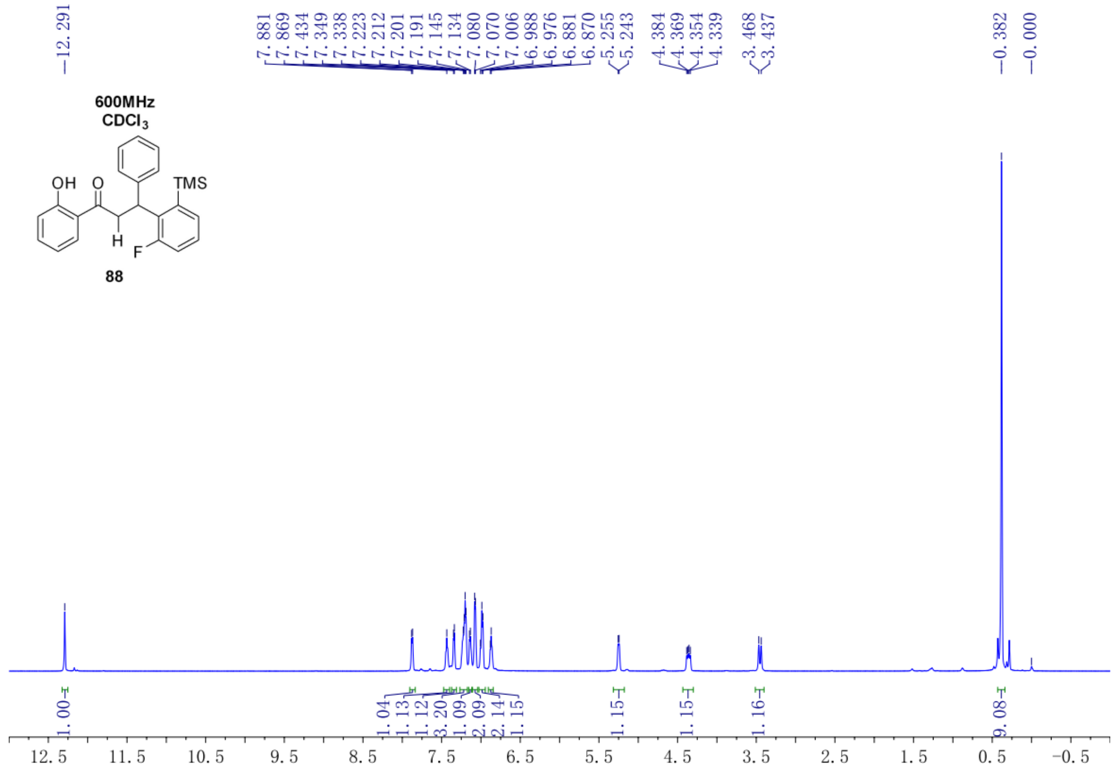
12.291
7.881
7.869
7.434
7.349
7.338
7.223
7.212
7.201
7.191
7.145
7.134
7.080
7.070
7.006
6.988
6.976
6.881
6.870
5.255
5.243
4.384
4.369
4.354
4.339
3.468
3.437
0.382
0.000
600MHz
CDCl3
OH
O
TMS
H
F
88
1.00
1.04
1.13
1.12
3.20
1.09
2.09
2.14
1.15
1.15
1.15
1.16
9.08
12.5
11.5
10.5
9.5
8.5
7.5
6.5
5.5
4.5
3.5
2.5
1.5
0.5
-0.5

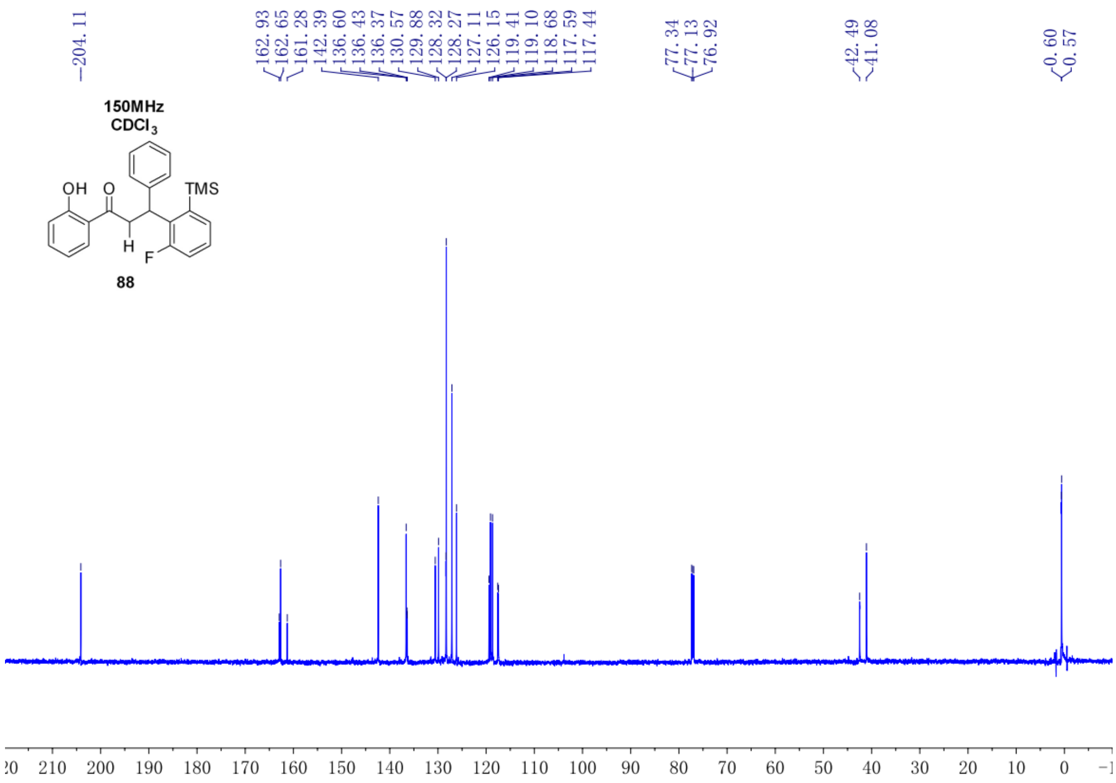
204.11
162.93
162.65
161.28
142.39
136.60
136.43
136.37
130.57
129.88
128.32
128.27
127.11
126.15
119.41
119.10
118.68
117.59
117.44
77.34
77.13
76.92
42.49
41.08
0.60
0.57
150MHz
$CDCl_3$
OH
O
TMS
H
F
88
210 200 190 180 170 160 150 140 130 120 110 100 90 80 70 60 50 40 30 20 10 0

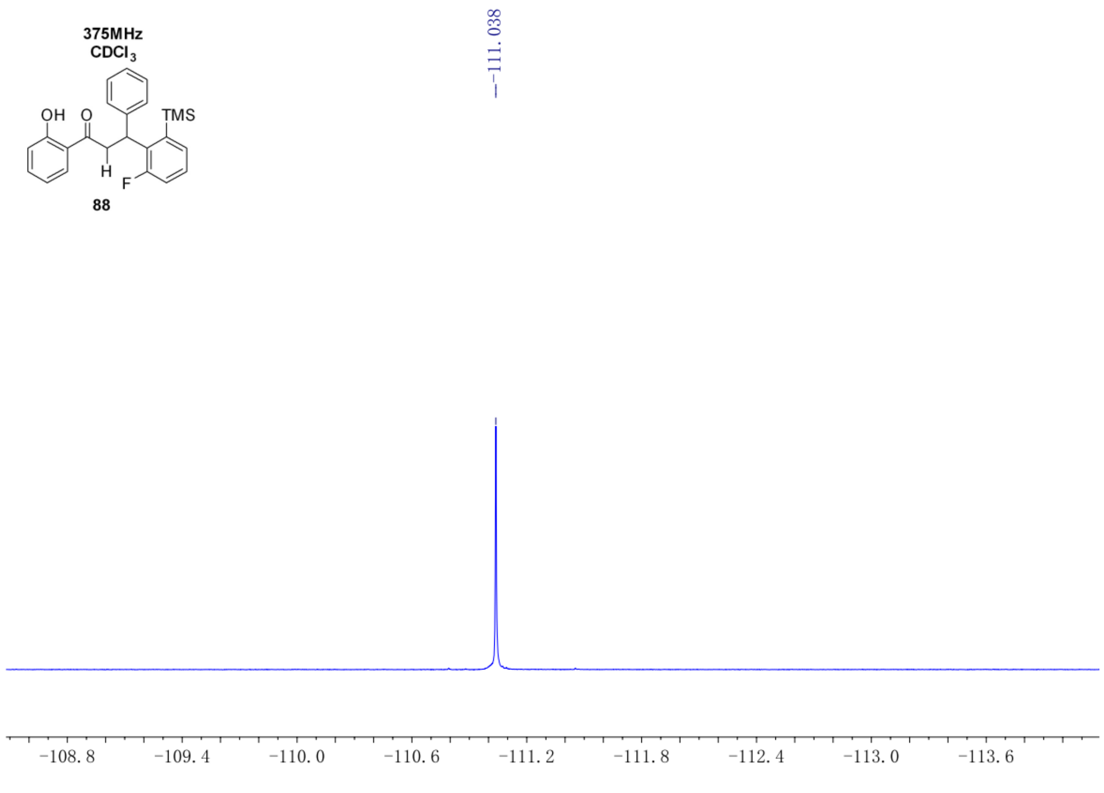
-111.038
375MHz
$CDCl_3$
OH
O
TMS
H
F
88
-108.8 -109.4 -110.0 -110.6 -111.2 -111.8 -112.4 -113.0 -113.6

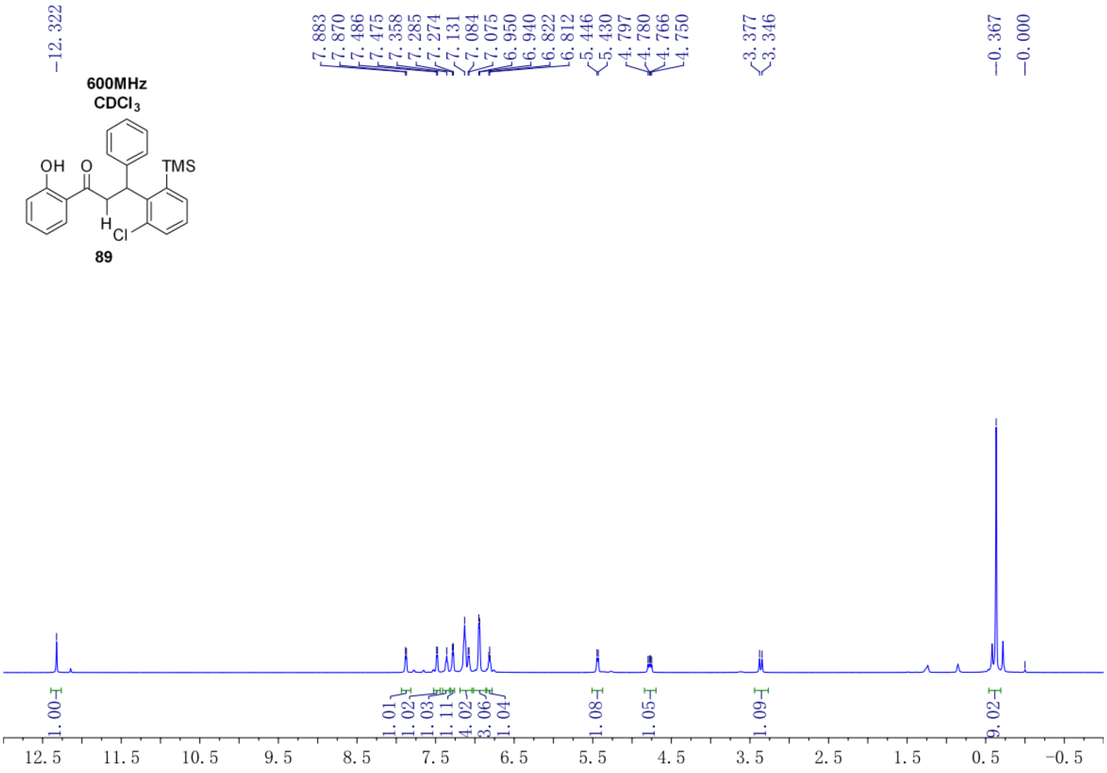
600MHz
CDCl3
OH O
TMS
H
Cl
89
12.322
7.883 7.870 7.486 7.475 7.358 7.285 7.274 7.131 7.084 7.075 6.950 6.940 6.822 6.812
5.446 5.430 4.797 4.780 4.766 4.750
3.377 3.346
0.367 0.000
1.00 1.01 1.02 1.03 1.11 4.02 3.06 1.04 1.08 1.05 1.09 9.02
12.5 11.5 10.5 9.5 8.5 7.5 6.5 5.5 4.5 3.5 2.5 1.5 0.5 -0.5

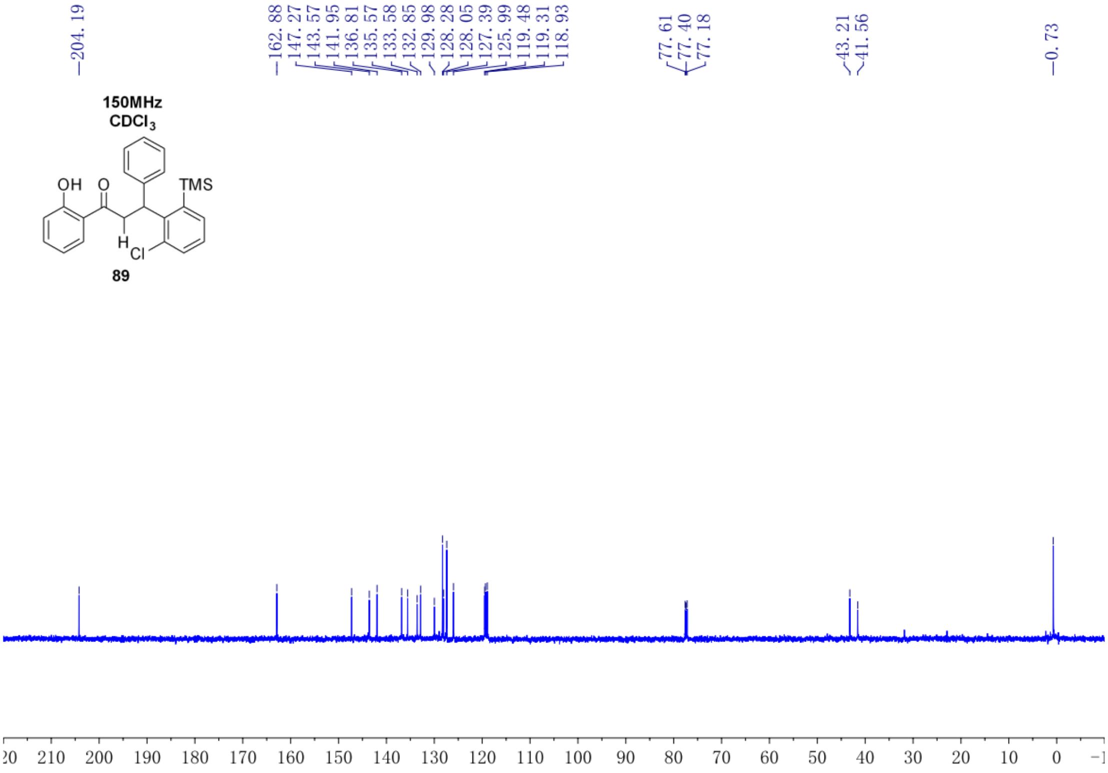
150MHz
CDCl3
OH O
TMS
H
Cl
89
204.19
162.88 147.27 143.57 141.95 136.81 135.57 133.58 132.85 129.98 128.28 128.05 127.39 125.99 119.48 119.31 118.93
77.61 77.40 77.18
43.21 41.56
0.73
210 200 190 180 170 160 150 140 130 120 110 100 90 80 70 60 50 40 30 20 10 0

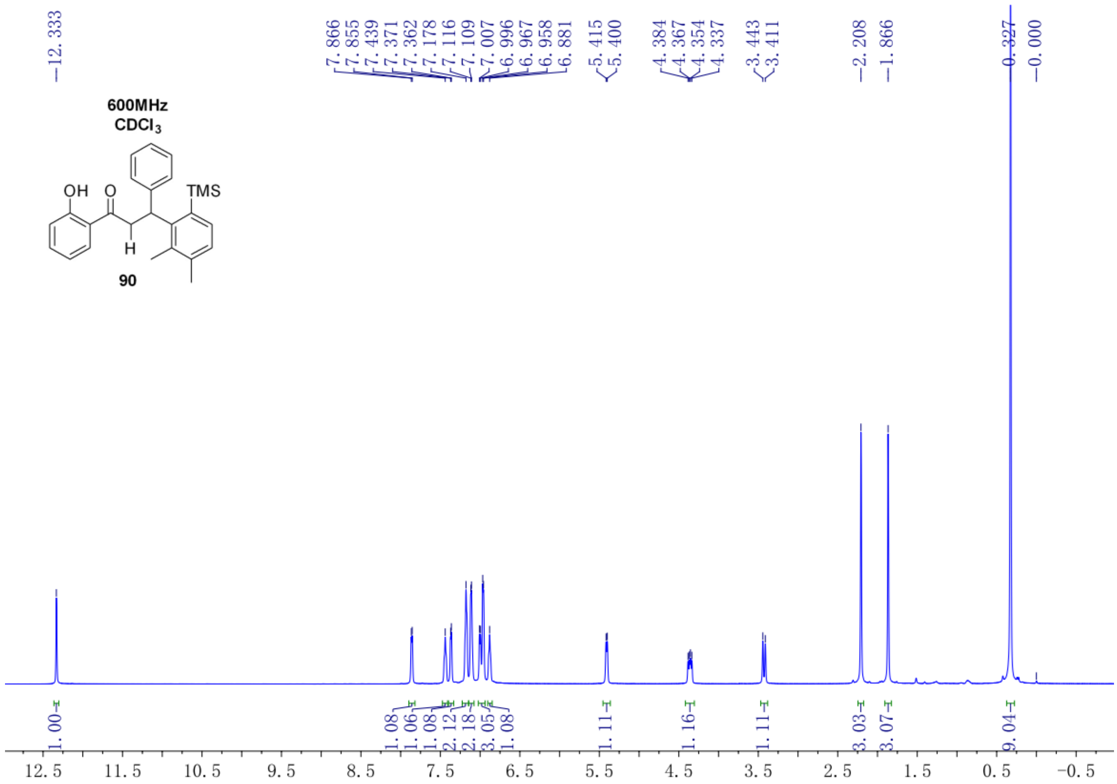
12.333
7.866 7.855 7.439 7.371 7.362 7.178 7.116 7.109 7.007 6.996 6.967 6.958 6.881
5.415 5.400
4.384 4.367 4.354 4.337
3.443 3.411
2.208
1.866
0.327
0.000
600MHz
$CDCl_3$
OH O TMS
H
90
1.00 1.08 1.06 1.08 2.12 2.18 3.05 1.08 1.11 1.16 1.11 3.03 3.07 9.04
12.5 11.5 10.5 9.5 8.5 7.5 6.5 5.5 4.5 3.5 2.5 1.5 0.5 −0.5

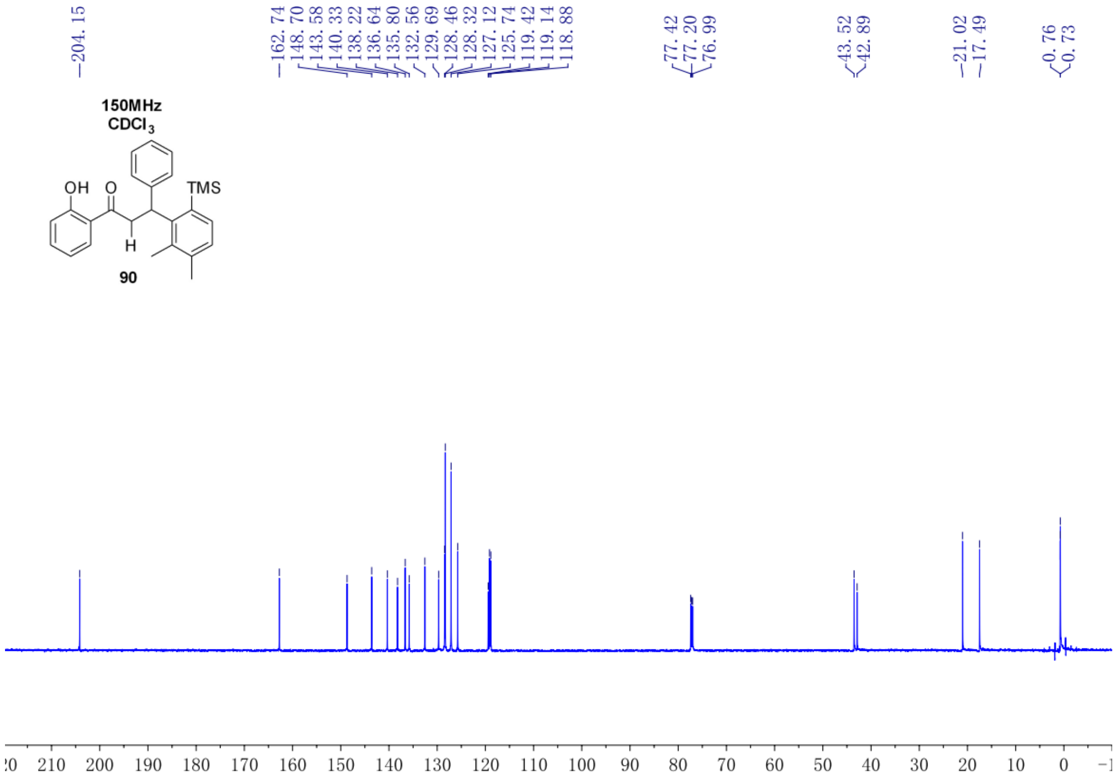
204.15
162.74 148.70 143.58 140.33 138.22 136.64 135.80 132.56 129.69 128.46 128.32 127.12 125.74 119.42 119.14 118.88
77.42 77.20 76.99
43.52 42.89
21.02 17.49
0.76 0.73
150MHz
$CDCl_3$
OH O TMS
H
90
210 200 190 180 170 160 150 140 130 120 110 100 90 80 70 60 50 40 30 20 10 0

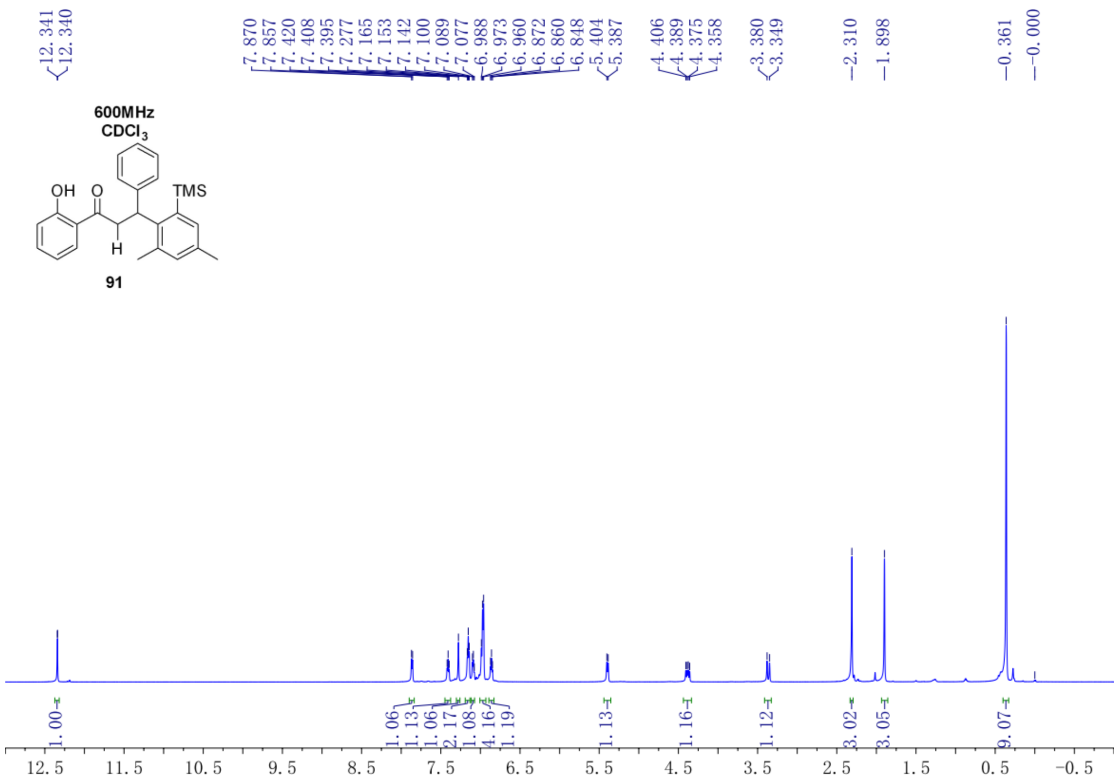

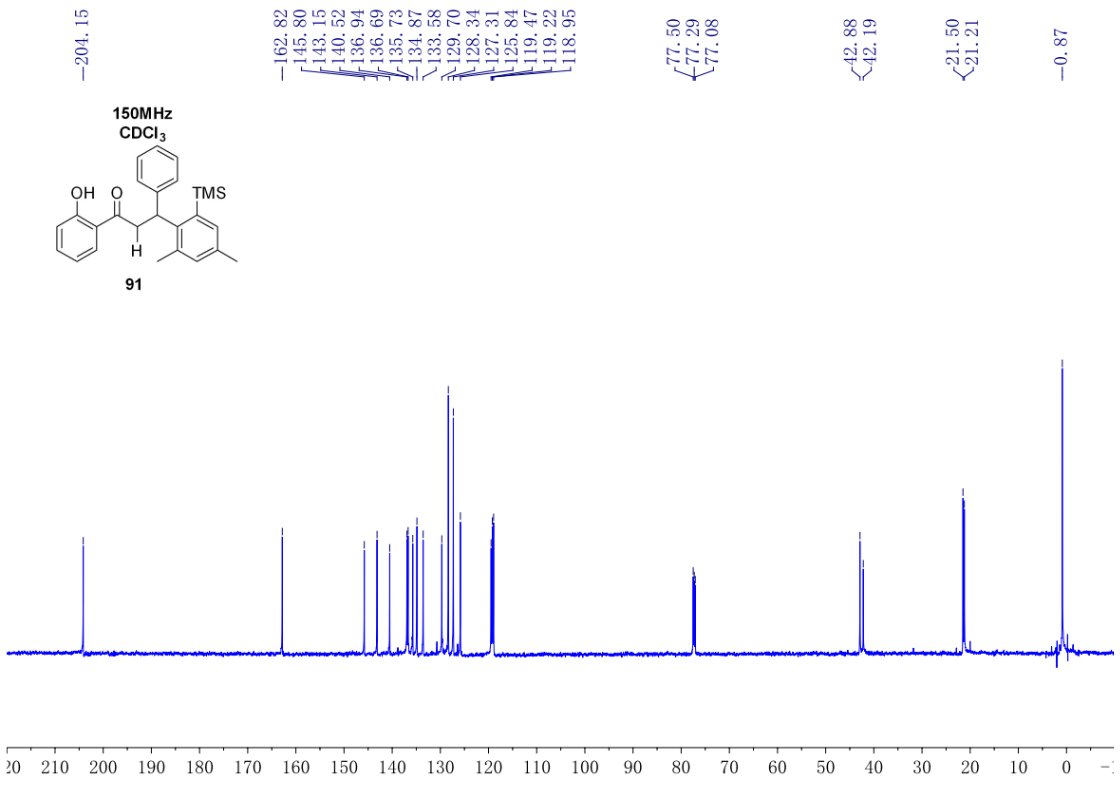

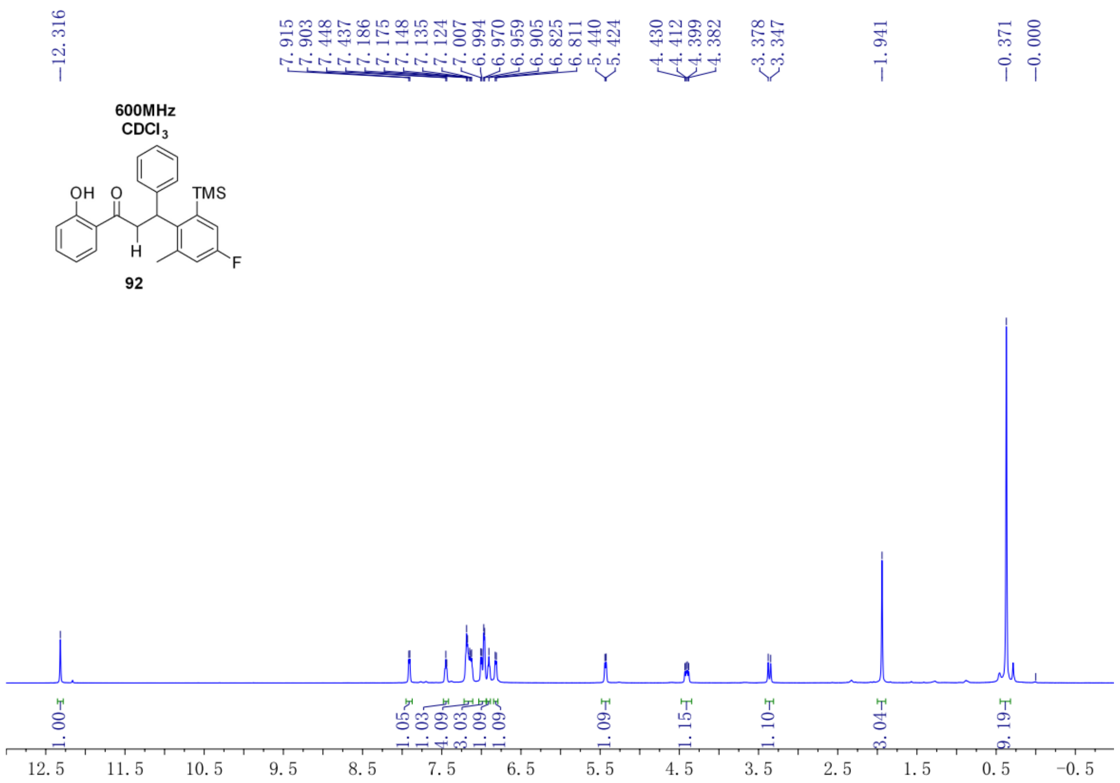
600MHz
CDCl3
92

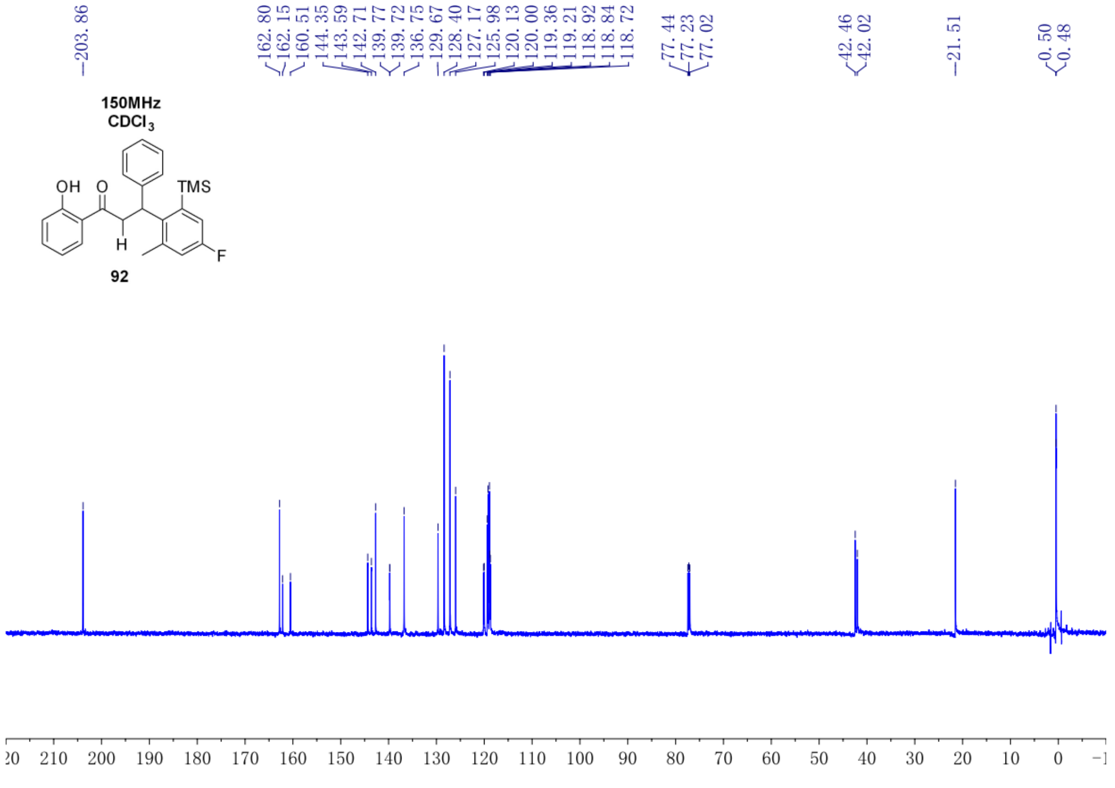
150MHz
CDCl3
92

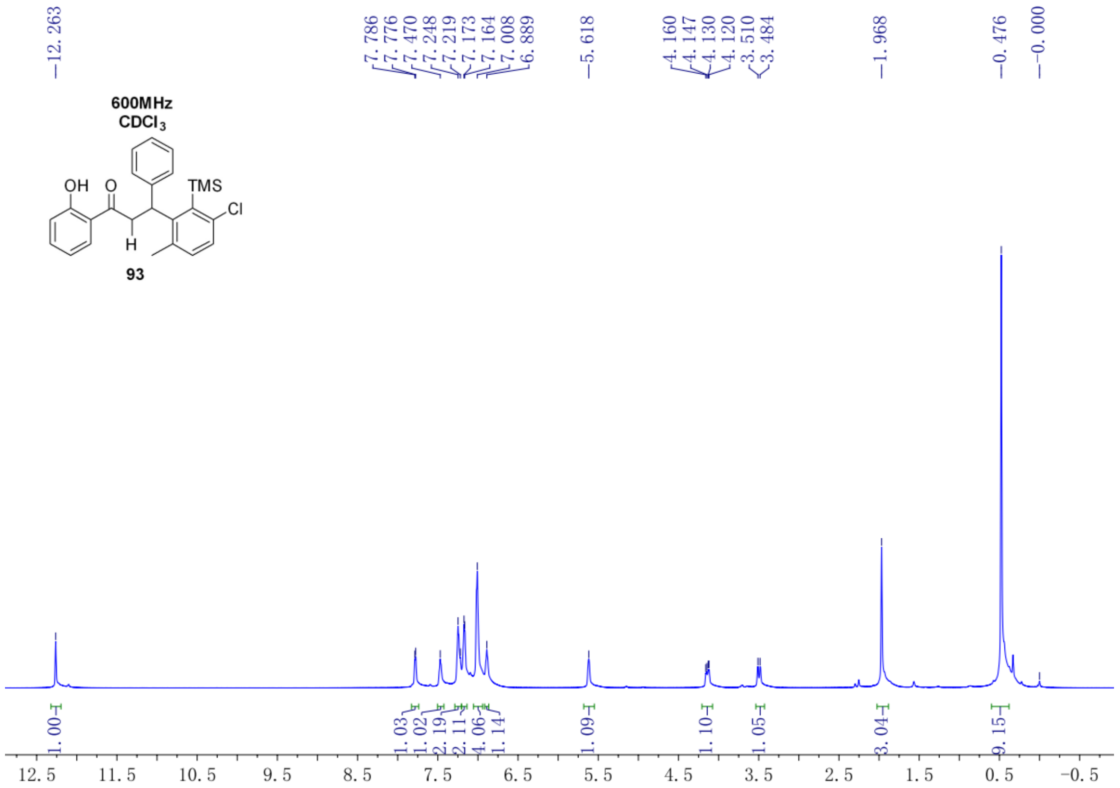
12.263
7.786
7.776
7.470
7.248
7.219
7.173
7.164
7.008
6.889
5.618
4.160
4.147
4.130
4.120
3.510
3.484
1.968
0.476
0.000
600MHz
CDCl$_3$
OH
O
TMS
Cl
H
93
1.00
1.03
1.02
2.19
2.11
4.06
1.14
1.09
1.10
1.05
3.04
9.15
12.5 11.5 10.5 9.5 8.5 7.5 6.5 5.5 4.5 3.5 2.5 1.5 0.5 -0.5

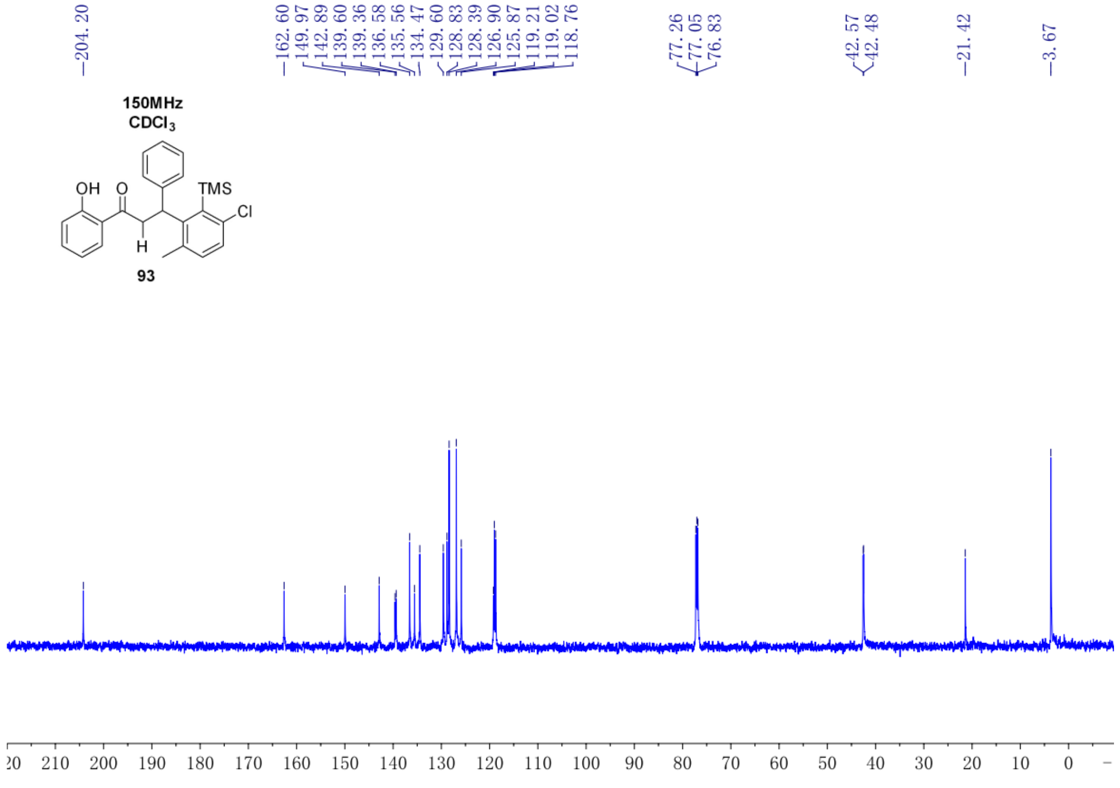
204.20
162.60
149.97
142.89
139.60
139.36
136.58
135.56
134.47
129.60
128.83
128.39
126.90
125.87
119.21
119.02
118.76
77.26
77.05
76.83
42.57
42.48
21.42
3.67
150MHz
CDCl$_3$
OH
O
TMS
Cl
H
93
210 200 190 180 170 160 150 140 130 120 110 100 90 80 70 60 50 40 30 20 10 0

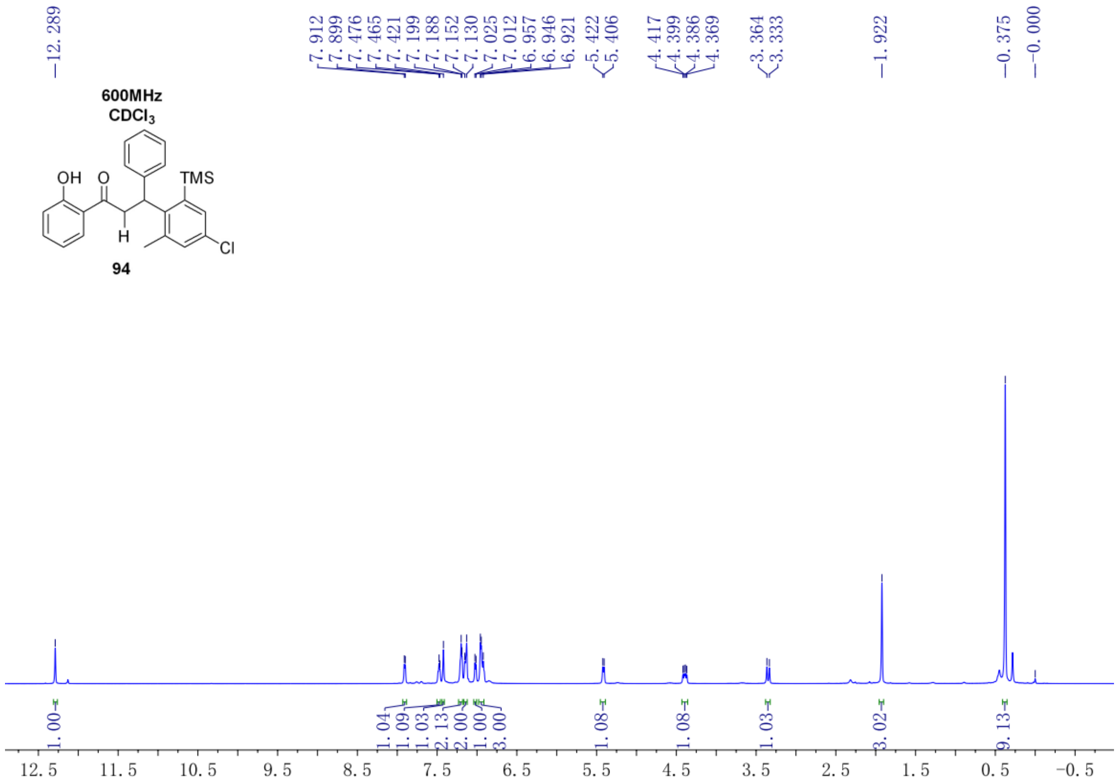
12.289
7.912 7.899 7.476 7.465 7.421 7.199 7.188 7.152 7.130 7.025 7.012 6.957 6.946 6.921
5.422 5.406
4.417 4.399 4.386 4.369
3.364 3.333
1.922
0.375
0.000
600MHz
CDCl3
OH
O
TMS
H
Cl
94
1.00 1.04 1.09 1.03 2.13 2.00 1.00 3.00 1.08 1.08 1.03 3.02 9.13
12.5 11.5 10.5 9.5 8.5 7.5 6.5 5.5 4.5 3.5 2.5 1.5 0.5 -0.5

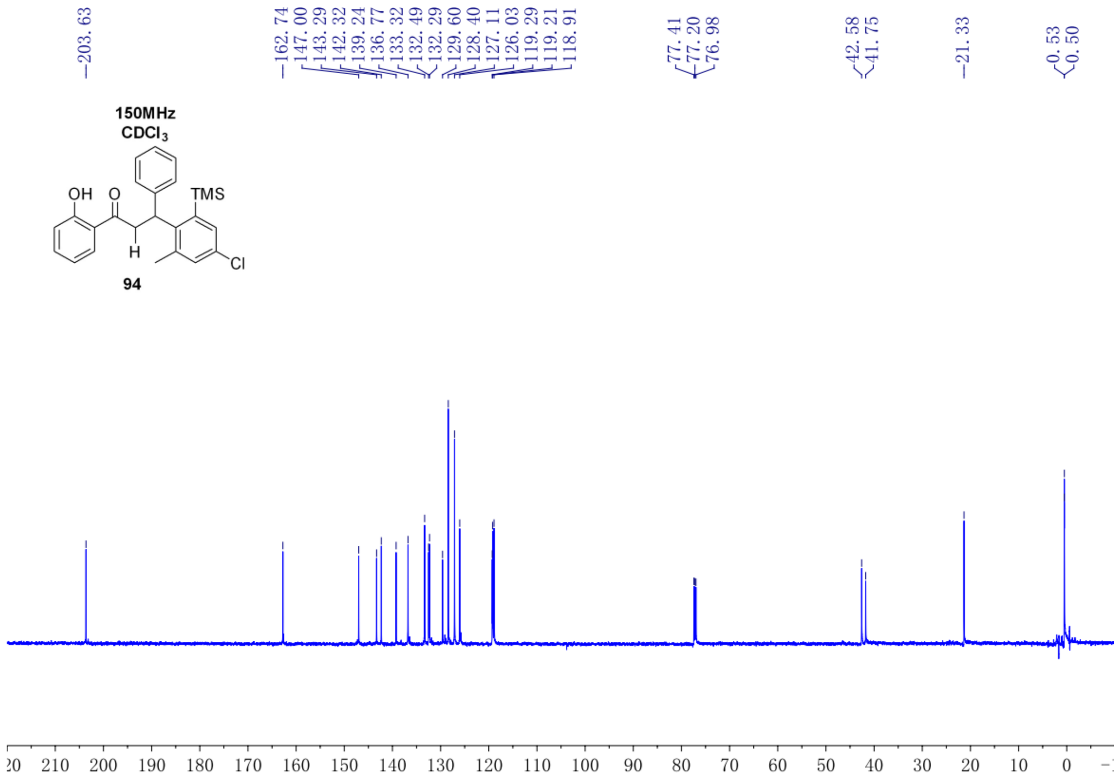
203.63
162.74 147.00 143.29 142.32 139.24 136.77 133.32 132.49 132.29 129.60 128.40 127.11 126.03 119.29 119.21 118.91
77.41 77.20 76.98
42.58 41.75
21.33
0.53 0.50
150MHz
CDCl3
OH
O
TMS
H
Cl
94
210 200 190 180 170 160 150 140 130 120 110 100 90 80 70 60 50 40 30 20 10 0

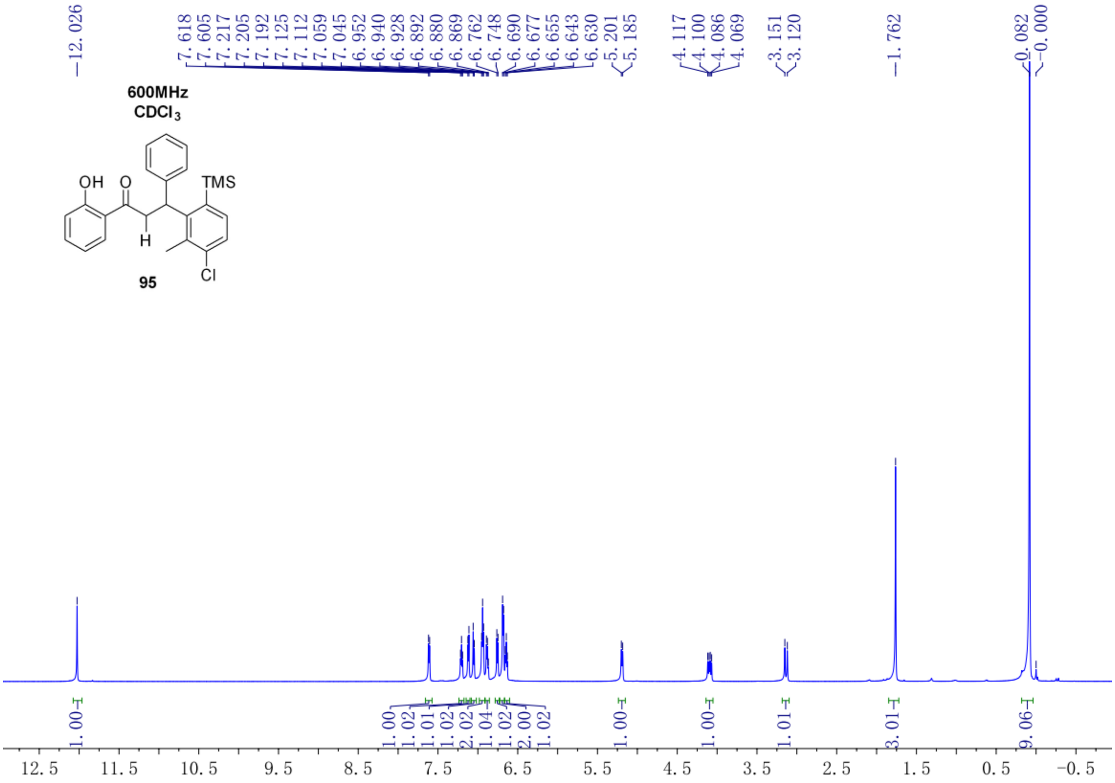
12.026
7.618 7.605 7.217 7.205 7.192 7.125 7.112 7.059 7.045 6.952 6.940 6.928 6.892 6.880 6.869 6.762 6.748 6.690 6.677 6.655 6.643 6.630 5.201 5.185
4.117 4.100 4.086 4.069
3.151 3.120
1.762
0.082 -0.000
600MHz
CDCl3
OH
O
TMS
H
Cl
95
1.00 1.00 1.02 1.01 1.02 2.02 1.04 1.02 2.00 1.02 1.00 1.00 1.01 3.01 9.06
12.5 11.5 10.5 9.5 8.5 7.5 6.5 5.5 4.5 3.5 2.5 1.5 0.5 -0.5

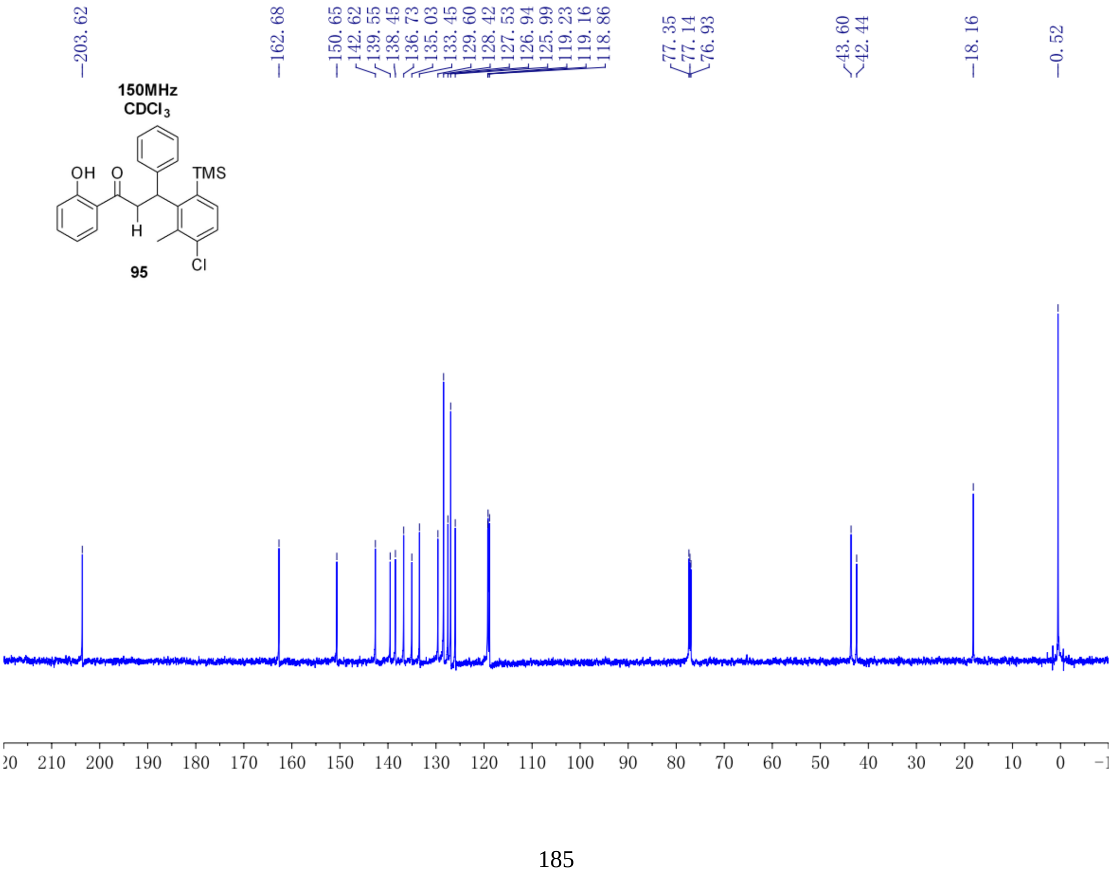
203.62
162.68
150.65 142.62 139.55 138.45 136.73 135.03 133.45 129.60 128.42 127.53 126.94 125.99 119.23 119.16 118.86
77.35 77.14 76.93
43.60 42.44
18.16
0.52
150MHz
CDCl3
OH
O
TMS
H
Cl
95
210 200 190 180 170 160 150 140 130 120 110 100 90 80 70 60 50 40 30 20 10 0

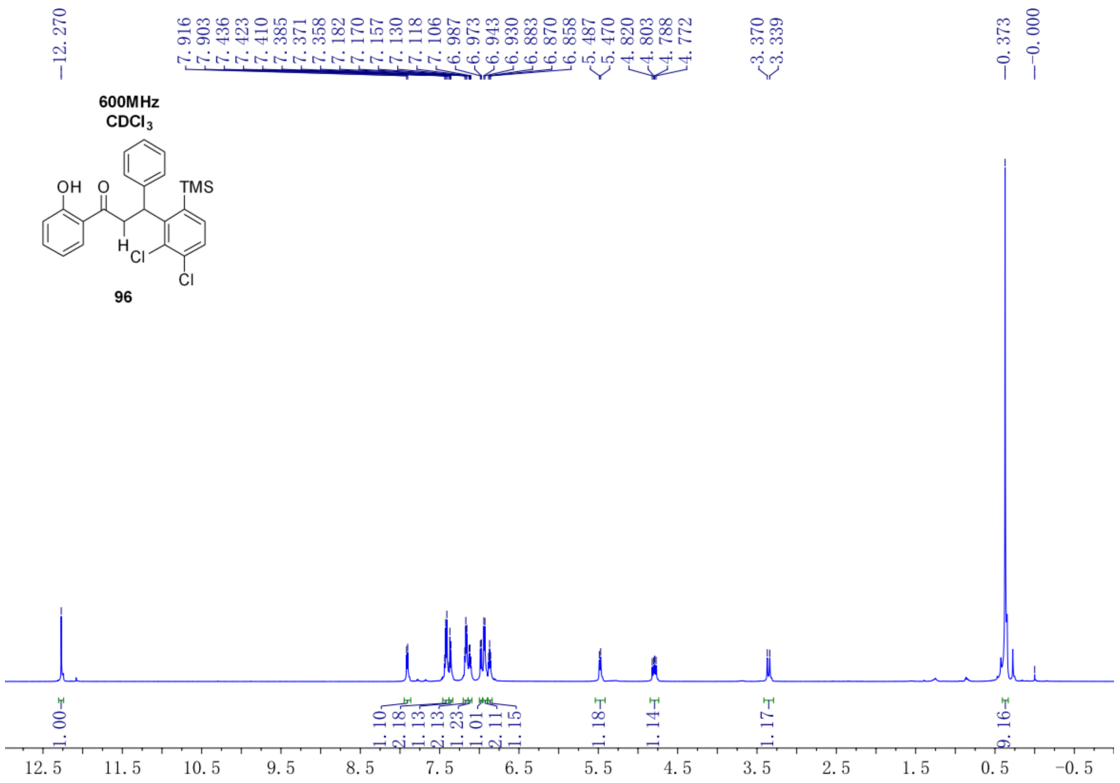
12.270
7.916 7.903 7.436 7.423 7.410 7.385 7.371 7.358 7.182 7.170 7.157 7.130 7.118 7.106 6.987 6.973 6.943 6.930 6.883 6.870 6.858 5.487 5.470 4.820 4.803 4.788 4.772
3.370 3.339
0.373
0.000
600MHz
CDCl3
OH
O
TMS
H
Cl
Cl
96
1.00
1.10 2.18 1.13 2.13 1.23 1.01 2.11 1.15
1.18
1.14
1.17
9.16
12.5 11.5 10.5 9.5 8.5 7.5 6.5 5.5 4.5 3.5 2.5 1.5 0.5 -0.5

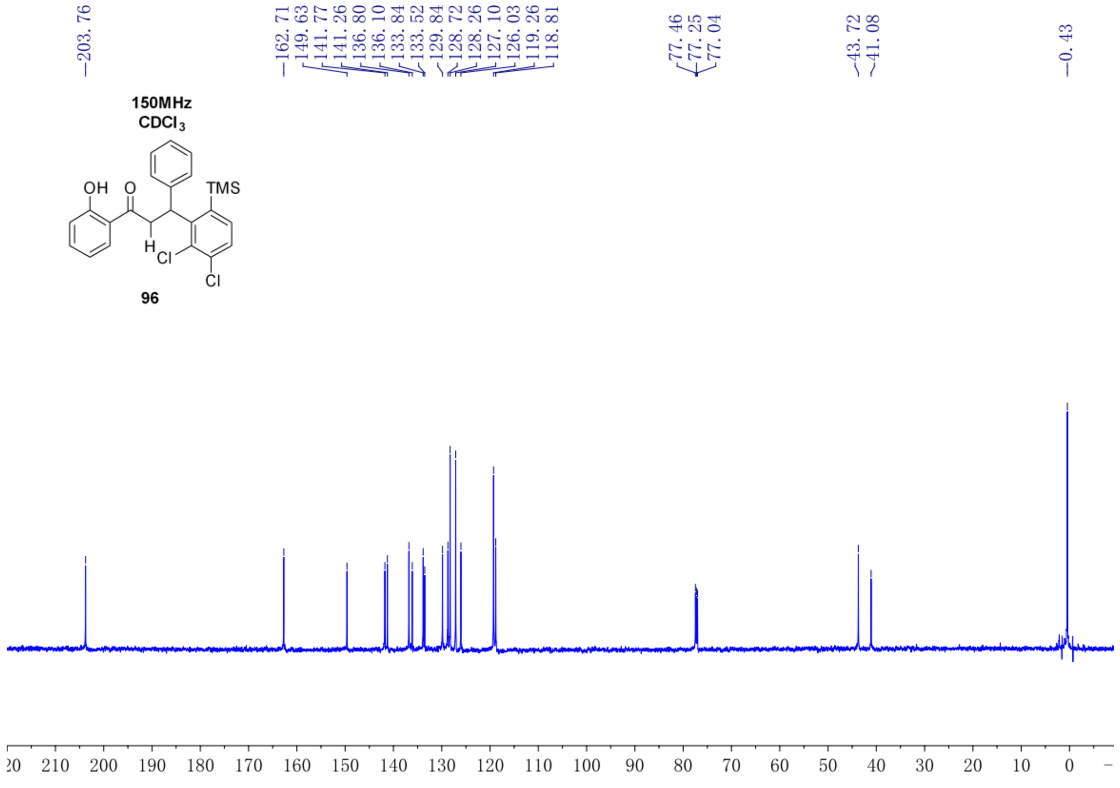
203.76
162.71 149.63 141.77 141.26 136.80 136.10 133.84 133.52 129.84 128.72 128.26 127.10 126.03 119.26 118.81
77.46 77.25 77.04
43.72 41.08
0.43
150MHz
CDCl3
OH
O
TMS
H
Cl
Cl
96
210 200 190 180 170 160 150 140 130 120 110 100 90 80 70 60 50 40 30 20 10 0

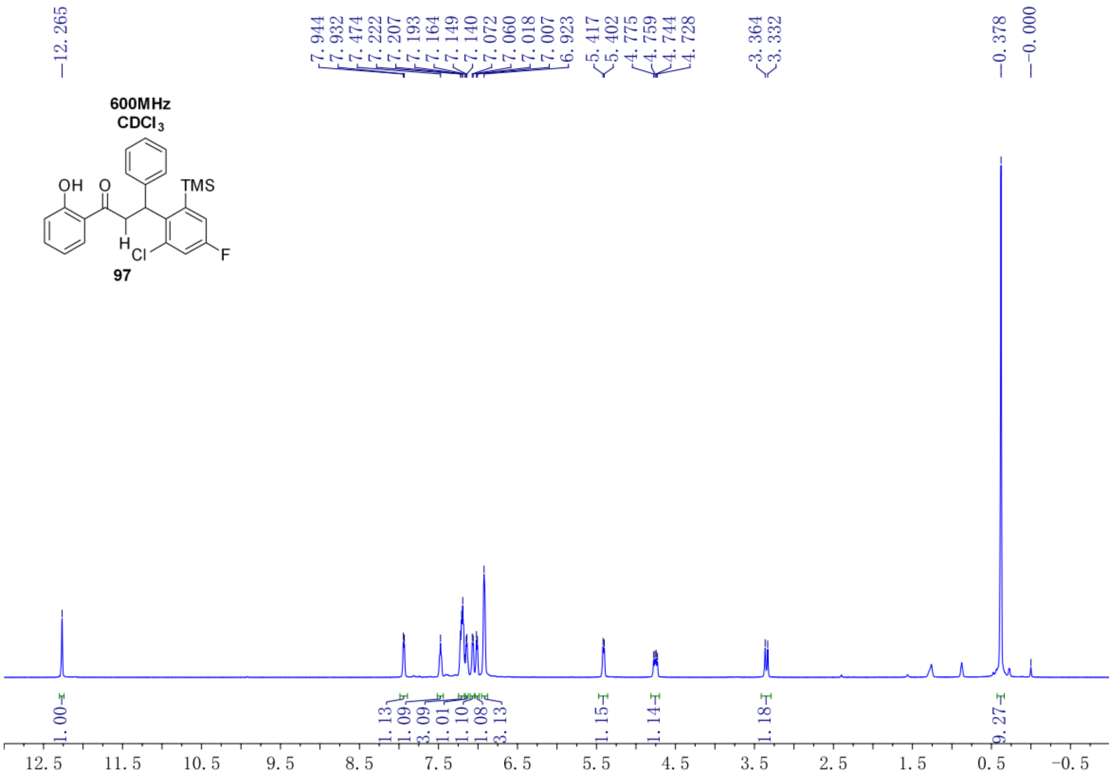
12.265
7.944
7.932
7.474
7.222
7.207
7.193
7.164
7.149
7.140
7.072
7.060
7.018
7.007
6.923
5.417
5.402
4.775
4.759
4.744
4.728
3.364
3.332
0.378
0.000
600MHz
CDCl3
OH
O
TMS
H
Cl
F
97
1.00
1.13
1.09
3.09
1.01
1.10
1.08
3.13
1.15
1.14
1.18
9.27
12.5
11.5
10.5
9.5
8.5
7.5
6.5
5.5
4.5
3.5
2.5
1.5
0.5
-0.5

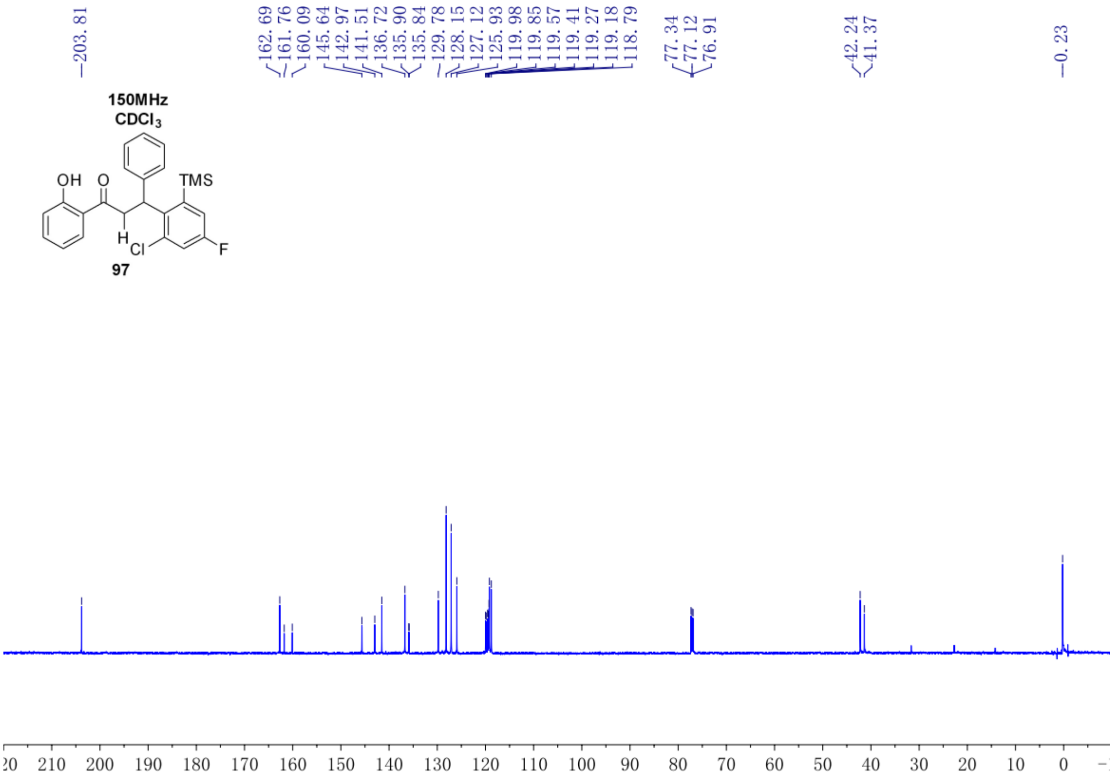
203.81
162.69
161.76
160.09
145.64
142.97
141.51
136.72
135.90
135.84
129.78
128.15
127.12
125.93
119.98
119.85
119.57
119.41
119.27
119.18
118.79
77.34
77.12
76.91
42.24
41.37
0.23
150MHz
CDCl3
OH
O
TMS
H
Cl
F
97
210
200
190
180
170
160
150
140
130
120
110
100
90
80
70
60
50
40
30
20
10
0

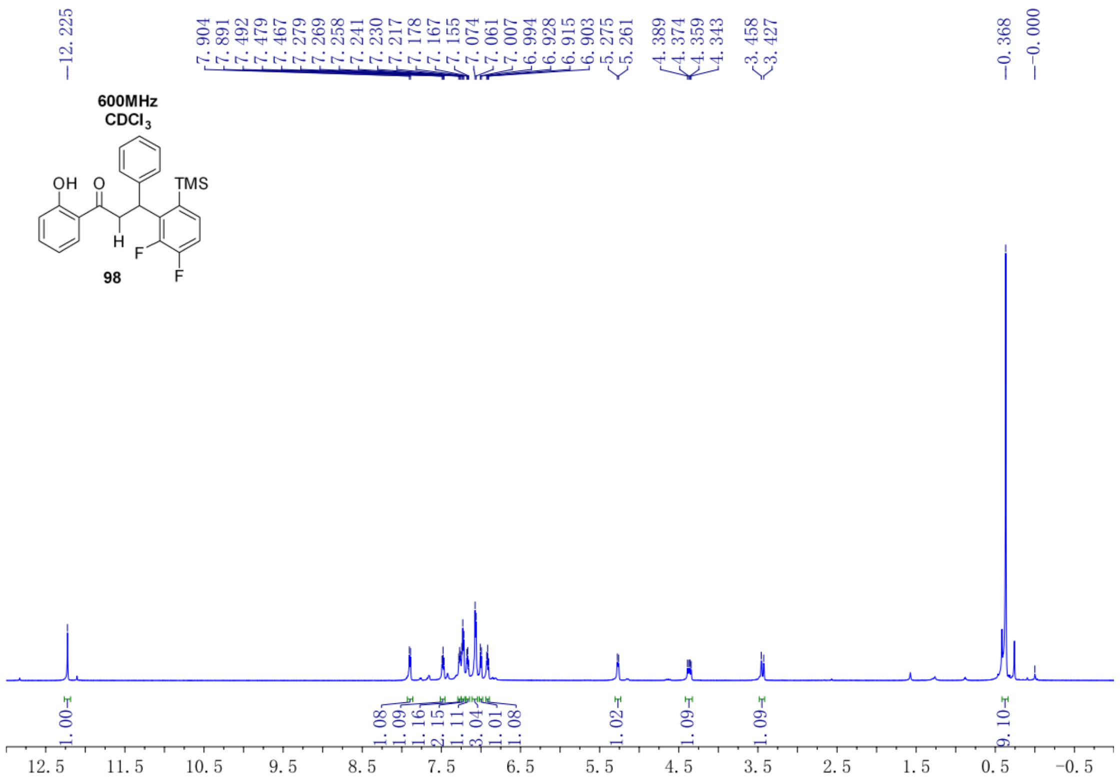

600MHz
CDCl3
98

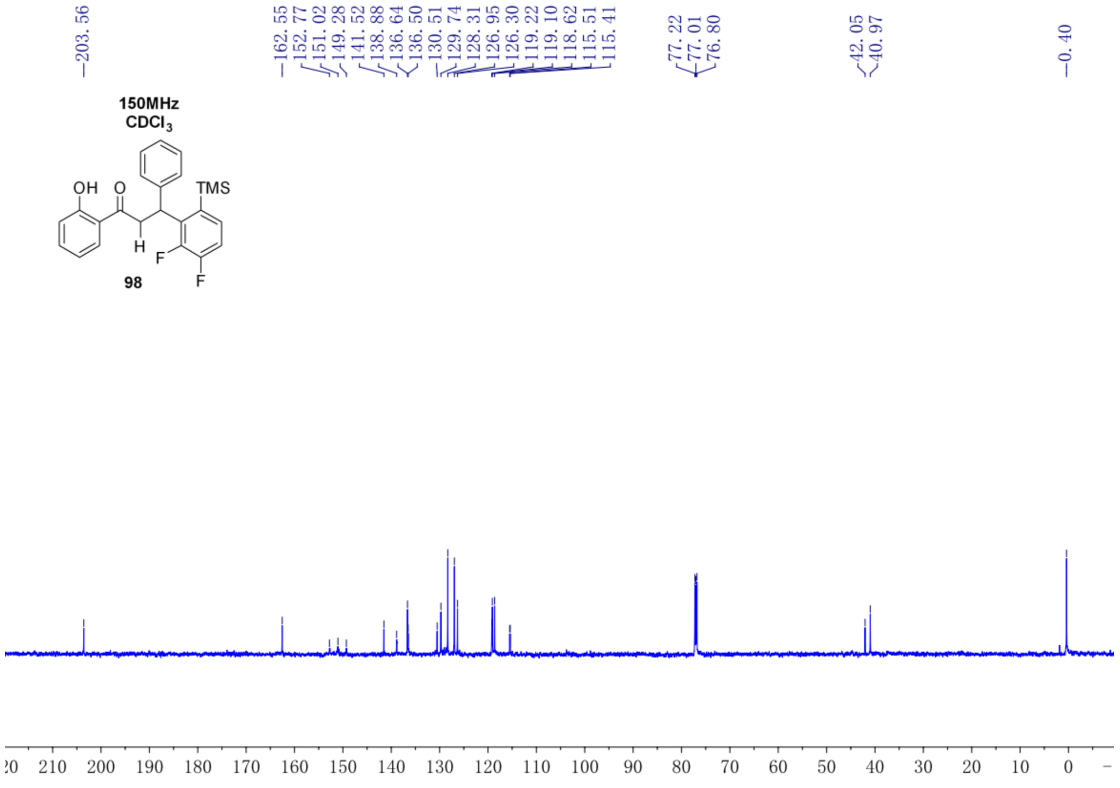

150MHz
CDCl3
98

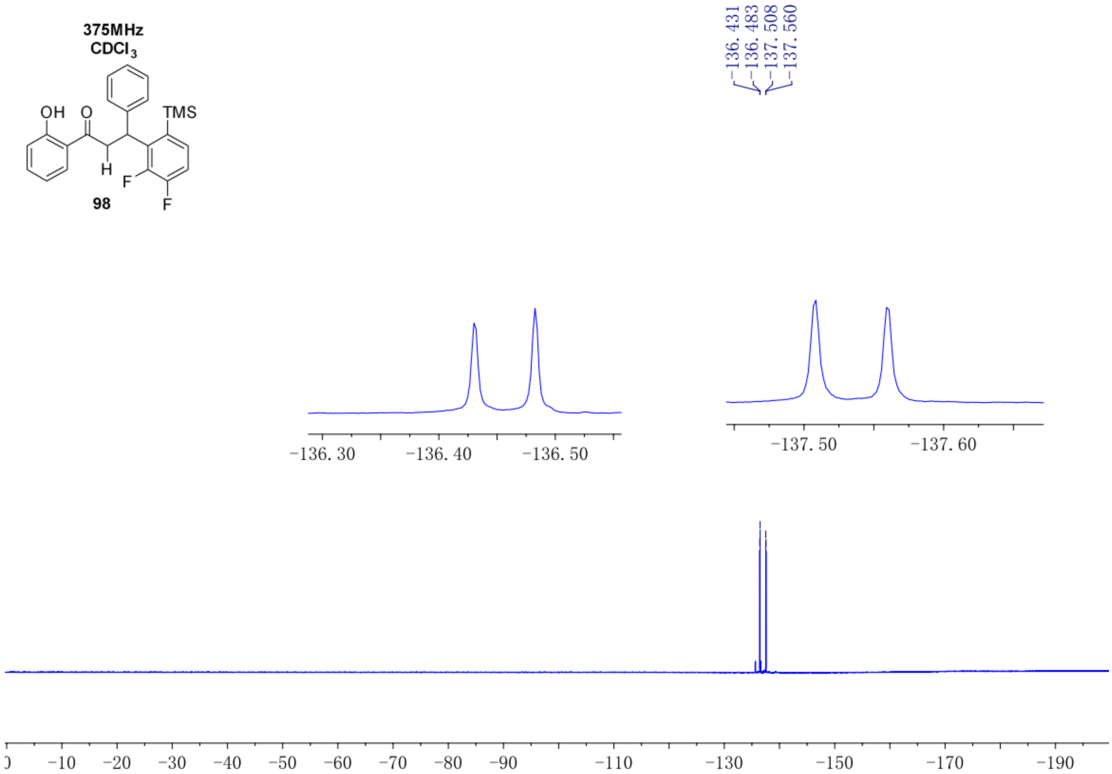
375MHz
CDCl3
OH O
TMS
H
F
F
98
-136.431
-136.483
-137.508
-137.560
-136.30 -136.40 -136.50
-137.50 -137.60
-10 -20 -30 -40 -50 -60 -70 -80 -90 -110 -130 -150 -170 -190

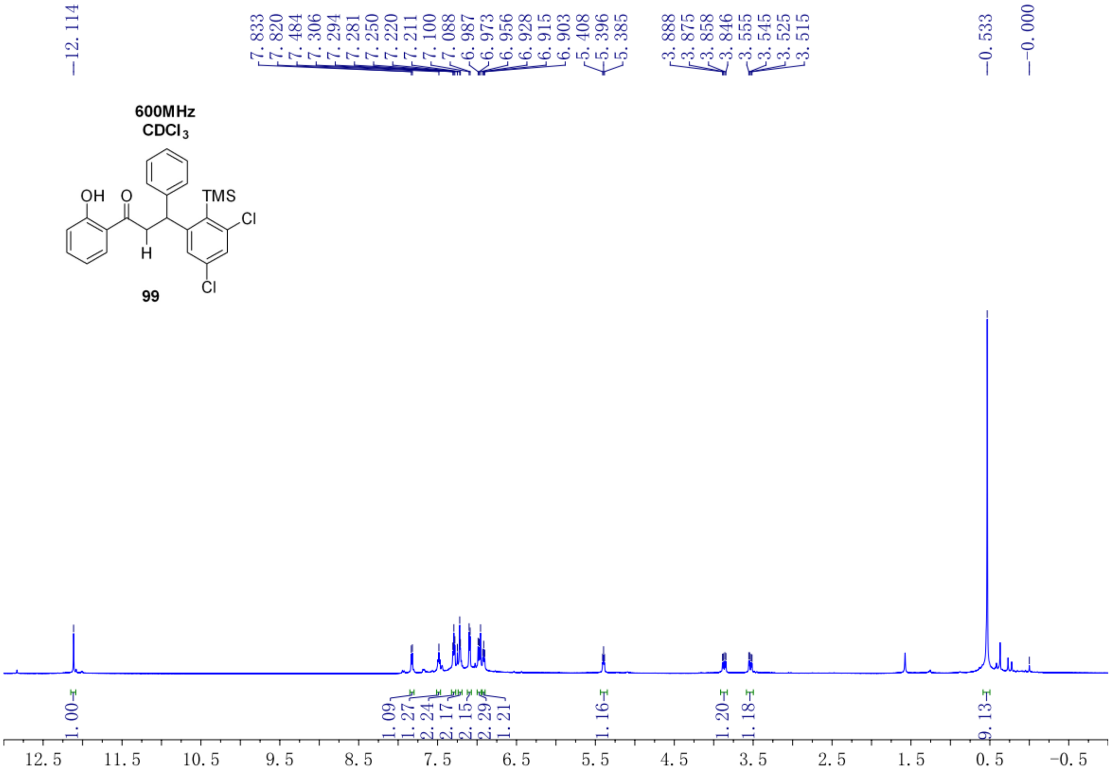
600MHz
CDCl3
OH O
TMS
Cl
H
Cl
99
12.114
7.833 7.820 7.484 7.306 7.294 7.281 7.250 7.220 7.211 7.100 7.088 6.987 6.973 6.956 6.928 6.915 6.903
5.408 5.396 5.385
3.888 3.875 3.858 3.846 3.555 3.545 3.525 3.515
0.533
0.000
1.00
1.09 1.27 2.24 2.17 2.15 2.29 1.21
1.16
1.20 1.18
9.13
12.5 11.5 10.5 9.5 8.5 7.5 6.5 5.5 4.5 3.5 2.5 1.5 0.5 -0.5

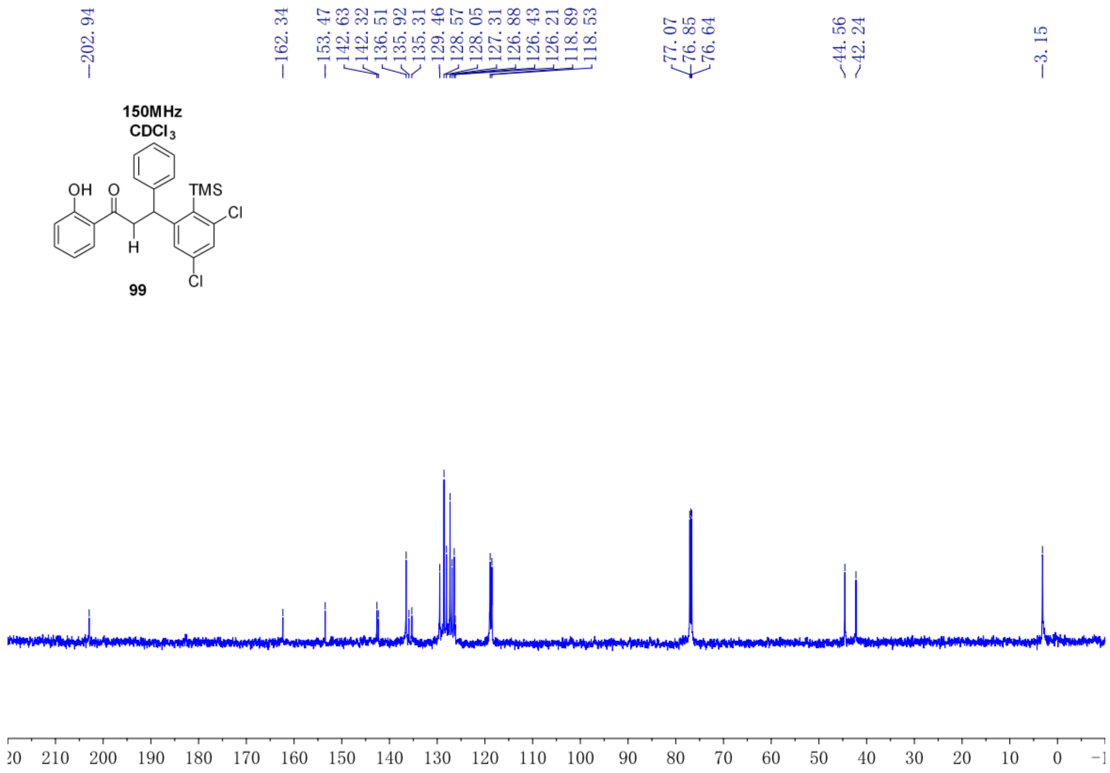
202.94
162.34
153.47
142.63
142.32
136.51
135.92
135.31
129.46
128.57
128.05
127.31
126.88
126.43
126.21
118.89
118.53
77.07
76.85
76.64
44.56
42.24
3.15
150MHz
$CDCl_3$
OH
O
TMS
Cl
H
Cl
99
20 210 200 190 180 170 160 150 140 130 120 110 100 90 80 70 60 50 40 30 20 10 0 −1

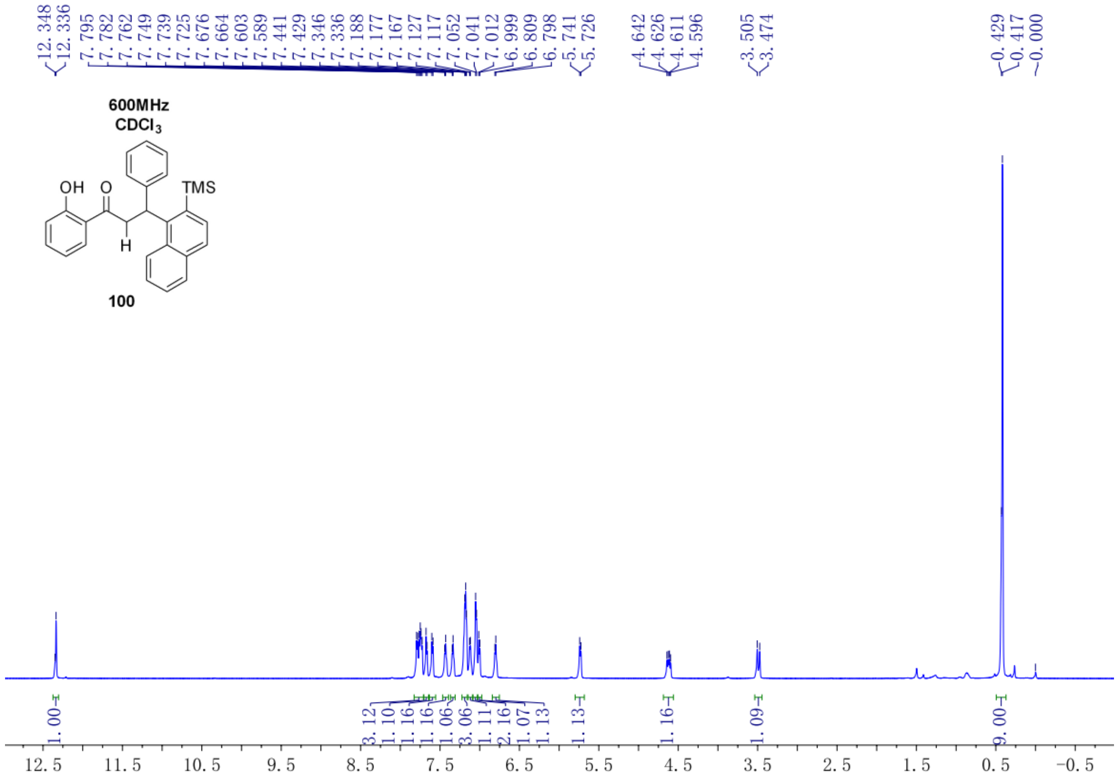
12.348
12.336
7.795
7.782
7.762
7.749
7.739
7.725
7.676
7.664
7.603
7.589
7.441
7.429
7.346
7.336
7.188
7.177
7.167
7.127
7.117
7.052
7.041
7.012
6.999
6.809
6.798
5.741
5.726
4.642
4.626
4.611
4.596
3.505
3.474
0.429
0.417
0.000
600MHz
$CDCl_3$
OH
O
TMS
H
100
1.00
3.12
1.10
1.16
1.16
1.06
3.06
1.11
2.16
1.07
1.13
1.13
1.16
1.09
9.00
12.5 11.5 10.5 9.5 8.5 7.5 6.5 5.5 4.5 3.5 2.5 1.5 0.5 −0.5

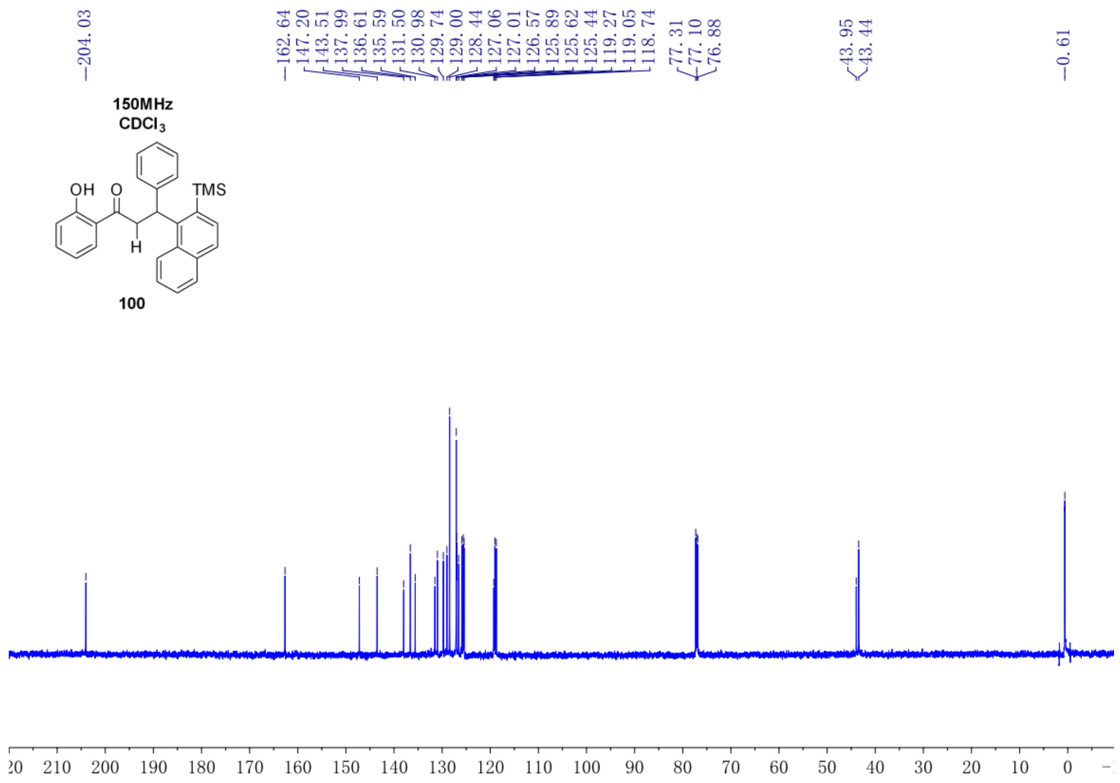
204.03
162.64 147.20 143.51 137.99 136.61 135.59 131.50 130.98 129.74 129.00 128.44 127.06 127.01 126.57 125.89 125.62 125.44 119.27 119.05 118.74
77.31 77.10 76.88
43.95 43.44
0.61
150MHz
CDCl3
OH O
TMS
H
100
210 200 190 180 170 160 150 140 130 120 110 100 90 80 70 60 50 40 30 20 10 0

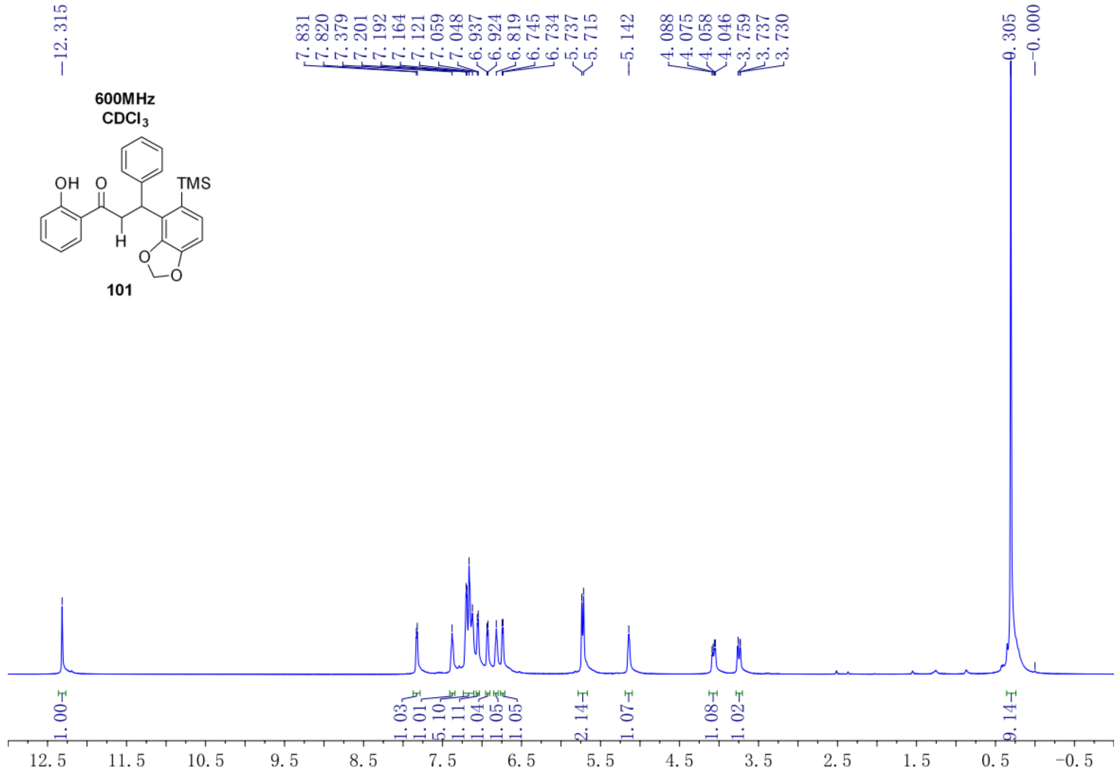
12.315
7.831 7.820 7.379 7.201 7.192 7.164 7.121 7.059 7.048 6.937 6.924 6.819 6.745 6.734 5.737 5.715
5.142
4.088 4.075 4.058 4.046 3.759 3.737 3.730
0.305
0.000
600MHz
CDCl3
OH O
TMS
H
O
O
101
1.00
1.03 1.01 5.10 1.11 1.04 1.05 1.05
2.14
1.07
1.08
1.02
9.14
12.5 11.5 10.5 9.5 8.5 7.5 6.5 5.5 4.5 3.5 2.5 1.5 0.5 -0.5

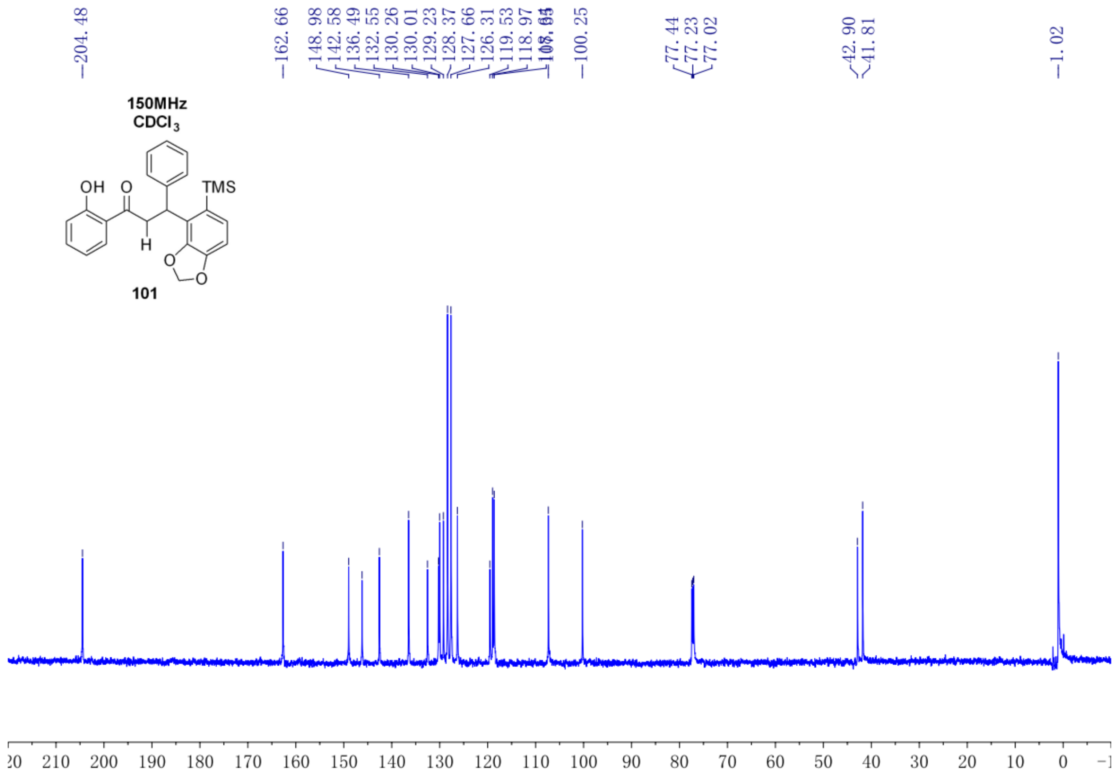
204.48
162.66
148.98
142.58
136.49
132.55
130.26
130.01
129.23
128.37
127.66
126.31
119.53
118.97
100.25
77.44
77.23
77.02
42.90
41.81
1.02
150MHz
CDCl3
OH
O
TMS
H
O
O
101

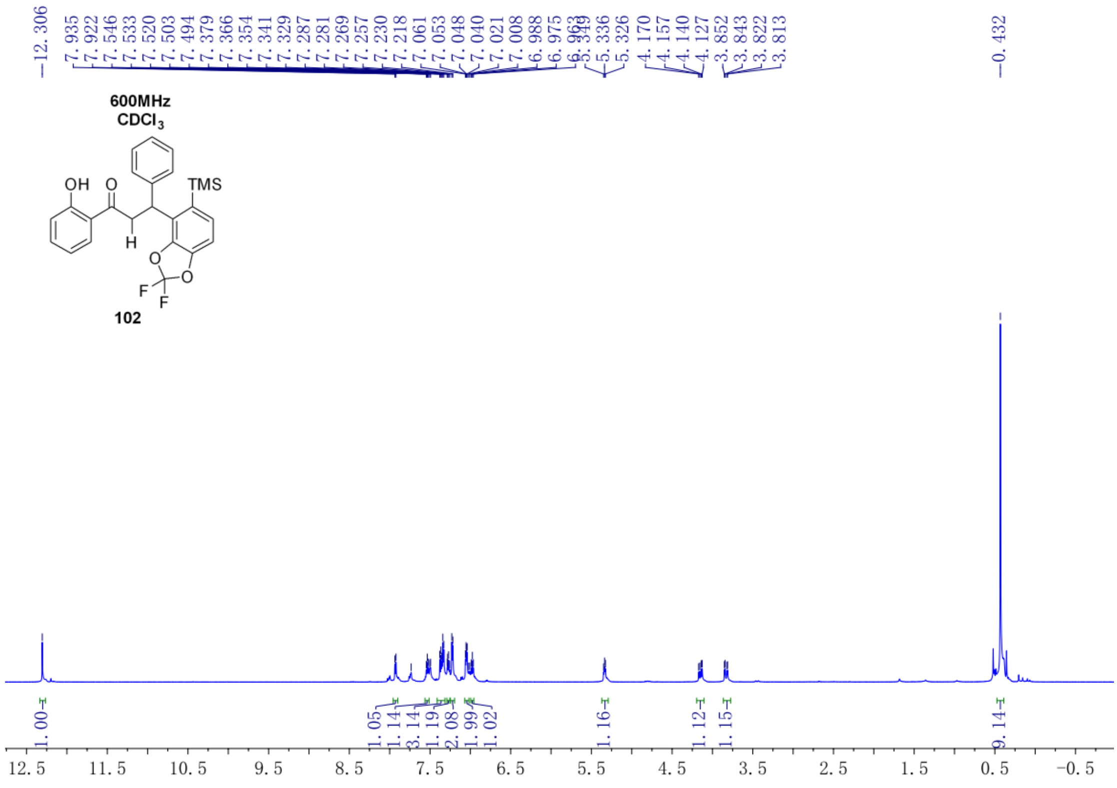
12.306
7.935
7.922
7.546
7.533
7.520
7.503
7.494
7.379
7.366
7.354
7.341
7.329
7.287
7.281
7.269
7.257
7.230
7.218
7.061
7.053
7.048
7.040
7.021
7.008
6.988
6.975
5.336
5.326
4.170
4.157
4.140
4.127
3.852
3.843
3.822
3.813
0.432
600MHz
CDCl3
OH
O
TMS
H
O
O
F
F
102
1.00
1.05
1.14
3.14
1.19
2.08
1.99
1.02
1.16
1.12
1.15
9.14

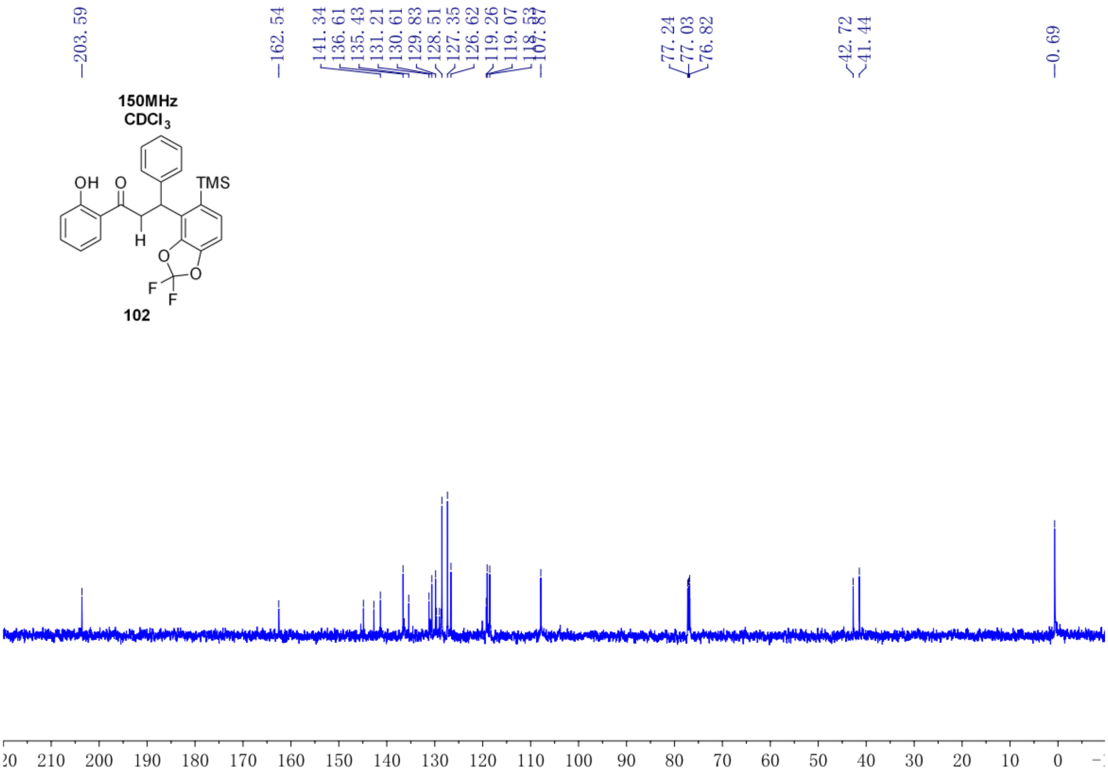
203.59
162.54
141.34
136.61
135.43
131.21
130.61
129.83
128.51
127.35
126.62
119.26
119.07
107.87
77.24
77.03
76.82
42.72
41.44
0.69
150MHz
CDCl3
OH
O
TMS
H
O
O
F
F
102

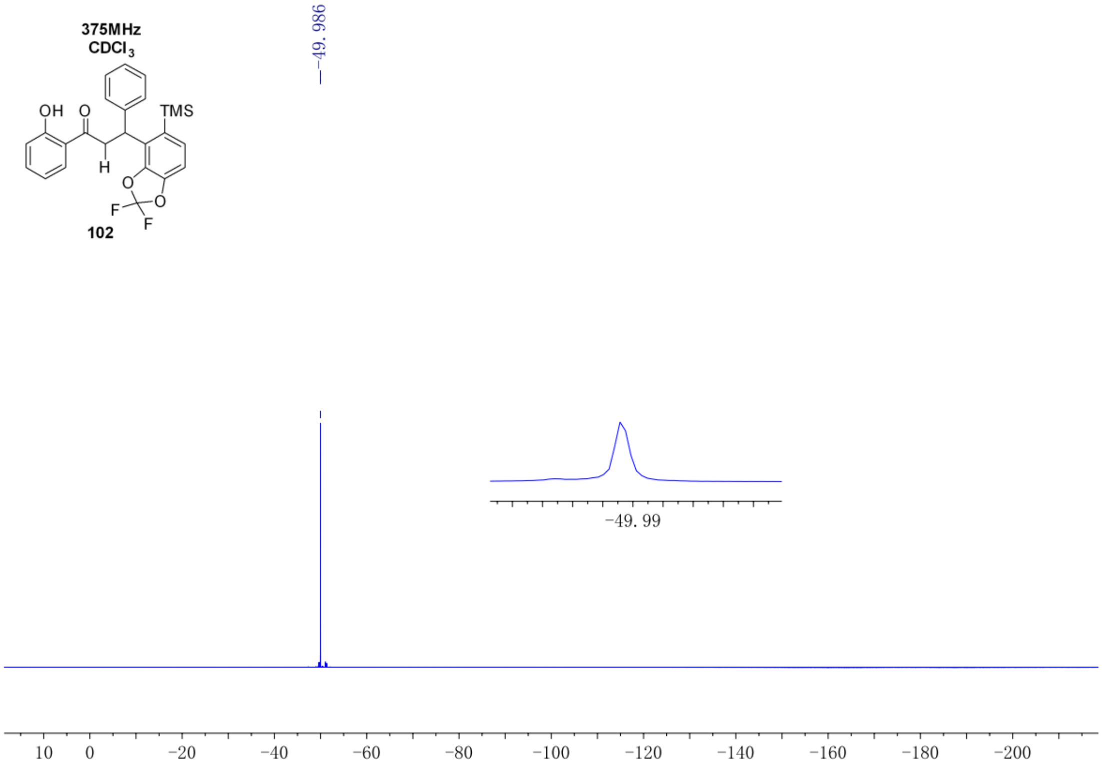
375MHz
CDCl3
-49.986
OH
O
TMS
H
O
O
F
F
102
-49.99

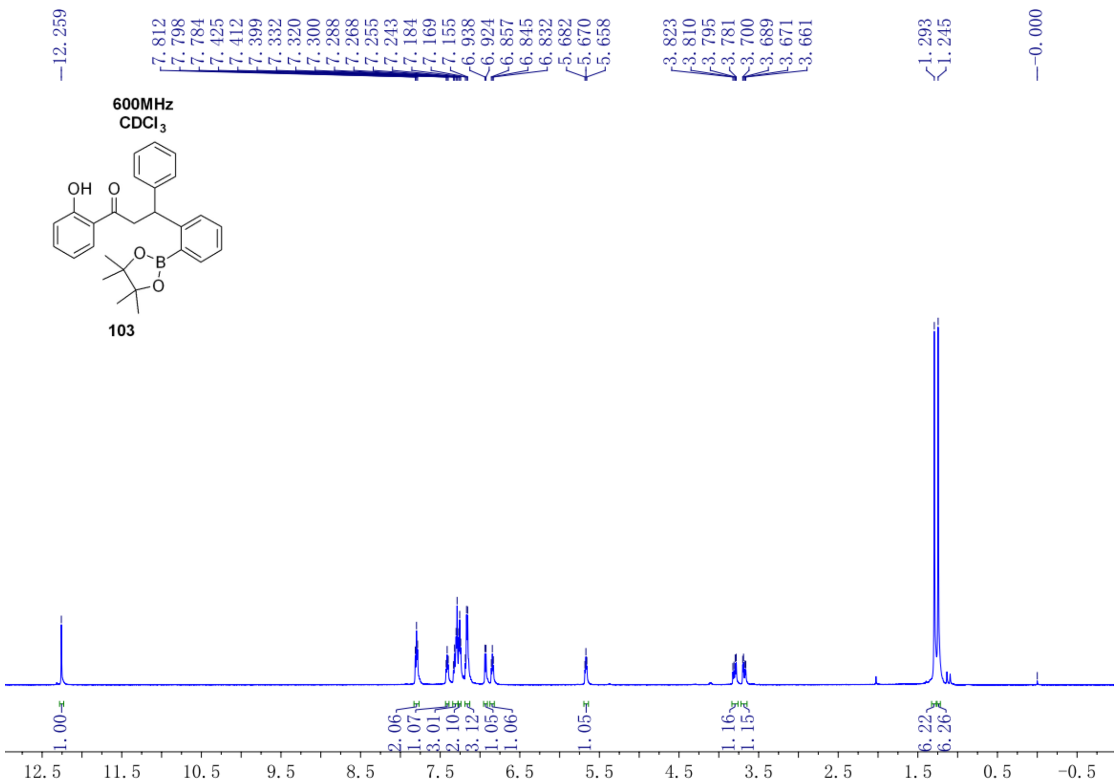

600MHz
CDCl3
103
12.259
7.812 7.798 7.784 7.425 7.412 7.399 7.332 7.320 7.300 7.288 7.268 7.255 7.243 7.184 7.169 7.155 6.938 6.924 6.857 6.845 6.832 5.682 5.670 5.658
3.823 3.810 3.795 3.781 3.700 3.689 3.671 3.661
1.293 1.245
0.000
1.00 2.06 1.07 3.01 2.10 3.12 1.05 1.06 1.05 1.16 1.15 6.22 6.26
12.5 11.5 10.5 9.5 8.5 7.5 6.5 5.5 4.5 3.5 2.5 1.5 0.5 -0.5

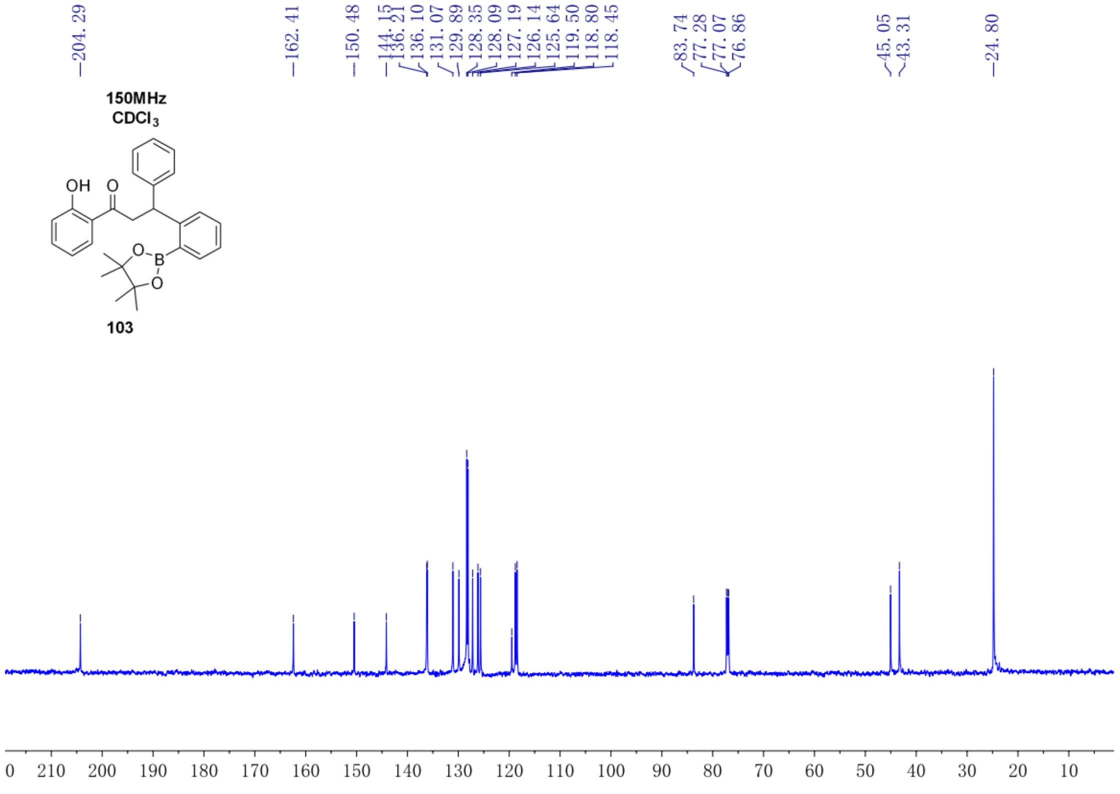

150MHz
CDCl3
103
204.29
162.41
150.48
144.15 136.21 136.10 131.07 129.89 128.35 128.09 127.19 126.14 125.64 119.50 118.80 118.45
83.74 77.28 77.07 76.86
45.05 43.31
24.80
0 210 200 190 180 170 160 150 140 130 120 110 100 90 80 70 60 50 40 30 20 10

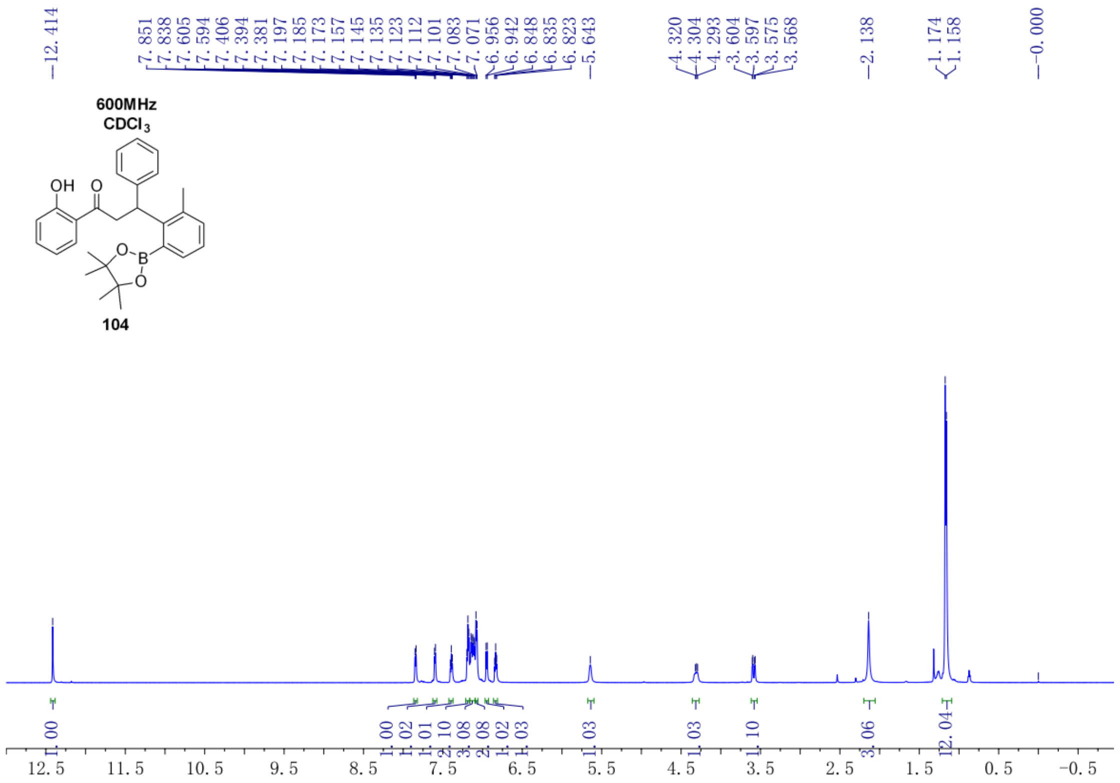
12.414
7.851 7.838 7.605 7.594 7.406 7.394 7.381 7.197 7.185 7.173 7.157 7.145 7.135 7.123 7.112 7.101 7.083 7.071 6.956 6.942 6.848 6.835 6.823 5.643
4.320 4.304 4.293 3.604 3.597 3.575 3.568
2.138
1.174 1.158
0.000
600MHz
CDCl3
OH
O
B
O
O
104
1.00 1.00 1.02 1.01 2.10 3.08 2.08 1.02 1.03 1.03 1.03 1.10 3.06 12.04
12.5 11.5 10.5 9.5 8.5 7.5 6.5 5.5 4.5 3.5 2.5 1.5 0.5 -0.5

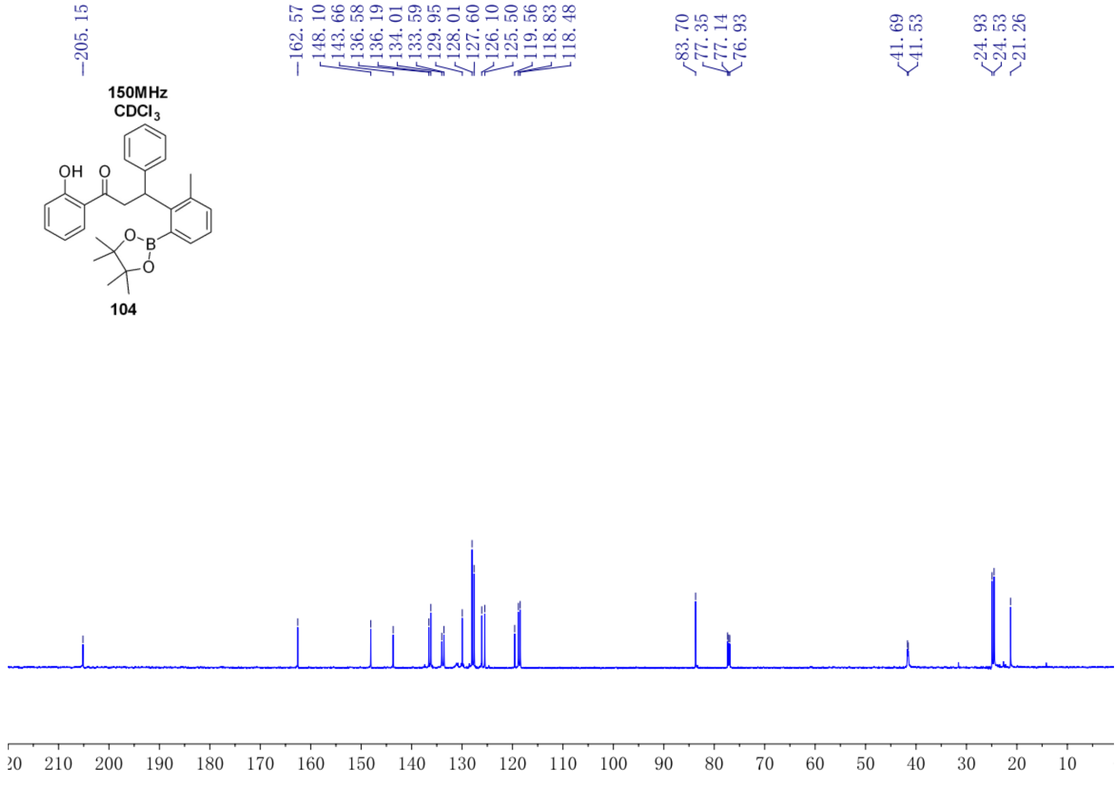
205.15
162.57 148.10 143.66 136.58 136.19 134.01 133.59 129.95 128.01 127.60 126.10 125.50 119.56 118.83 118.48
83.70 77.35 77.14 76.93
41.69 41.53
24.93 24.53 21.26
150MHz
CDCl3
OH
O
B
O
O
104
20 210 200 190 180 170 160 150 140 130 120 110 100 90 80 70 60 50 40 30 20 10

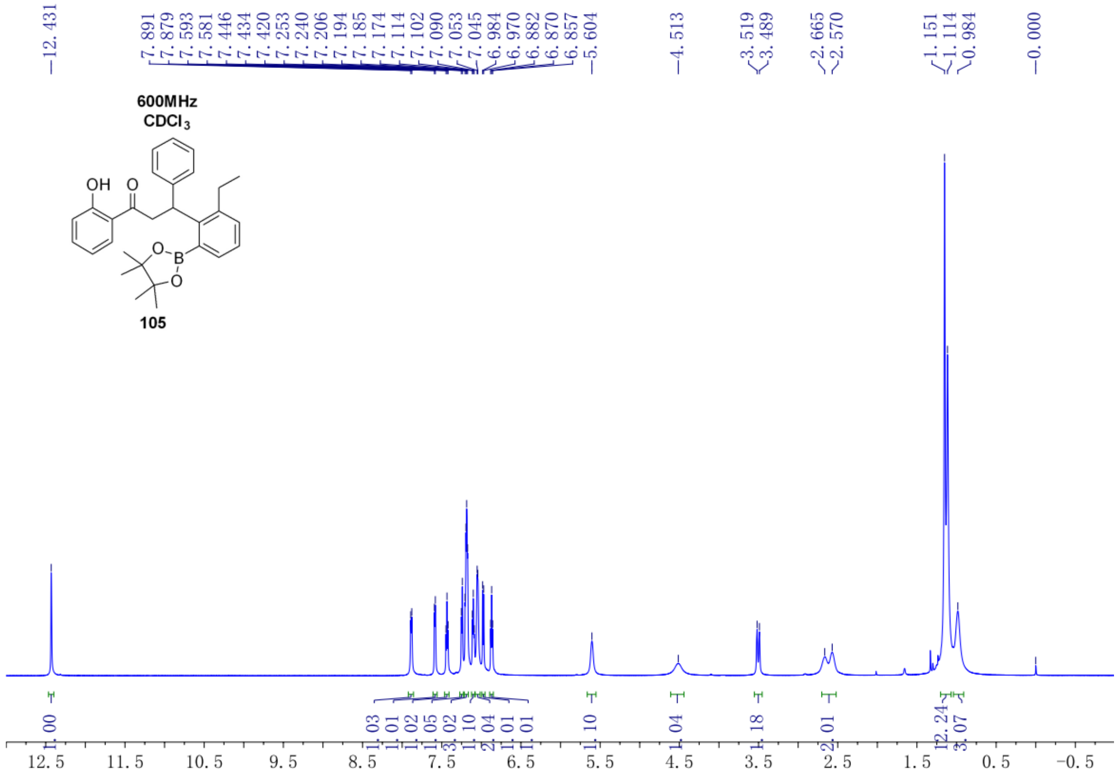

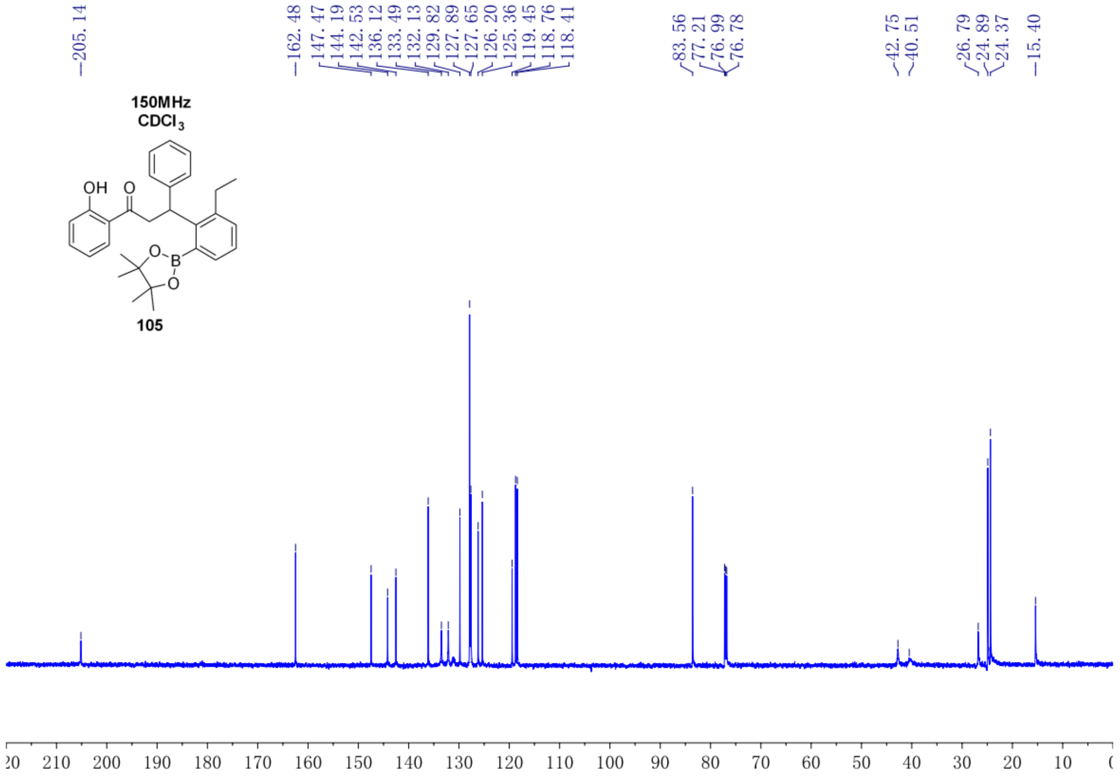

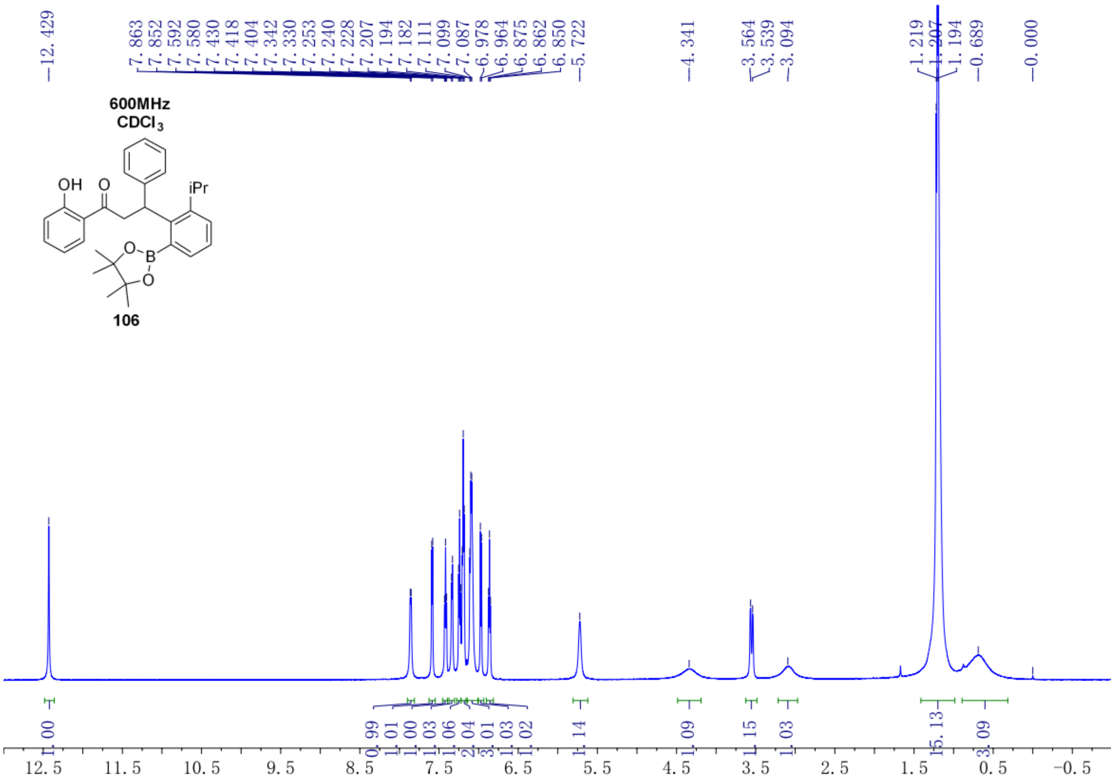
600MHz
CDCl3
OH
O
iPr
O
B
O
106

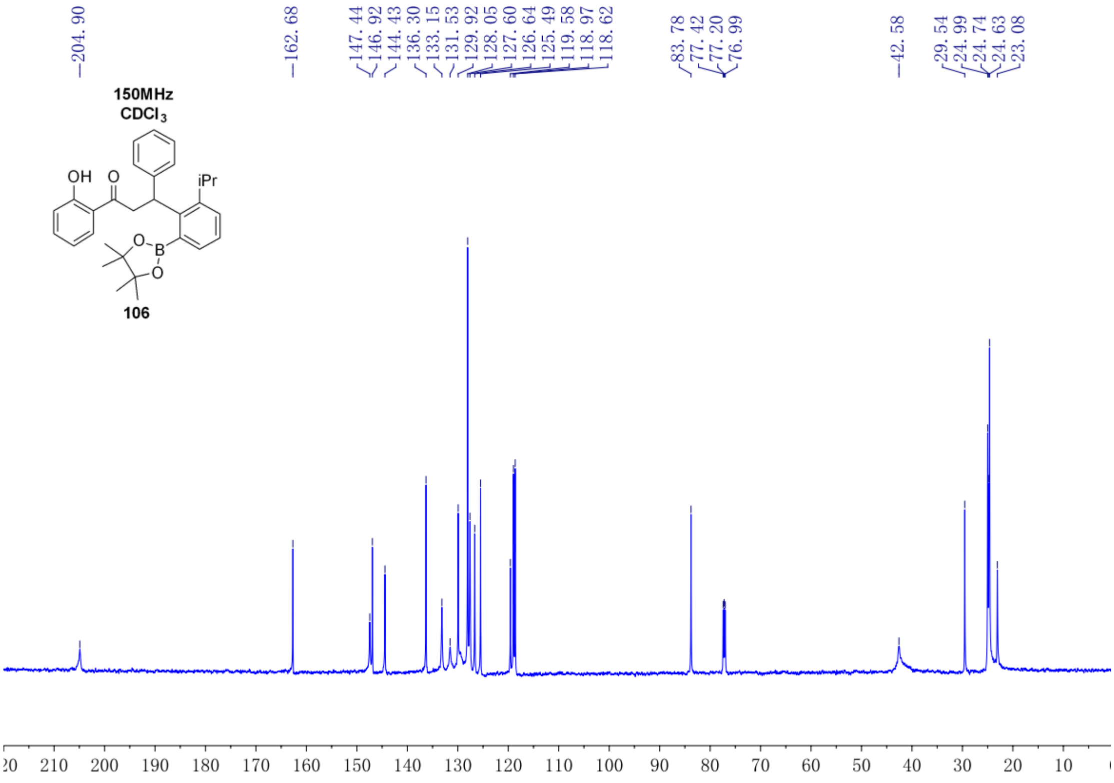
150MHz
CDCl3
OH
O
iPr
O
B
O
106

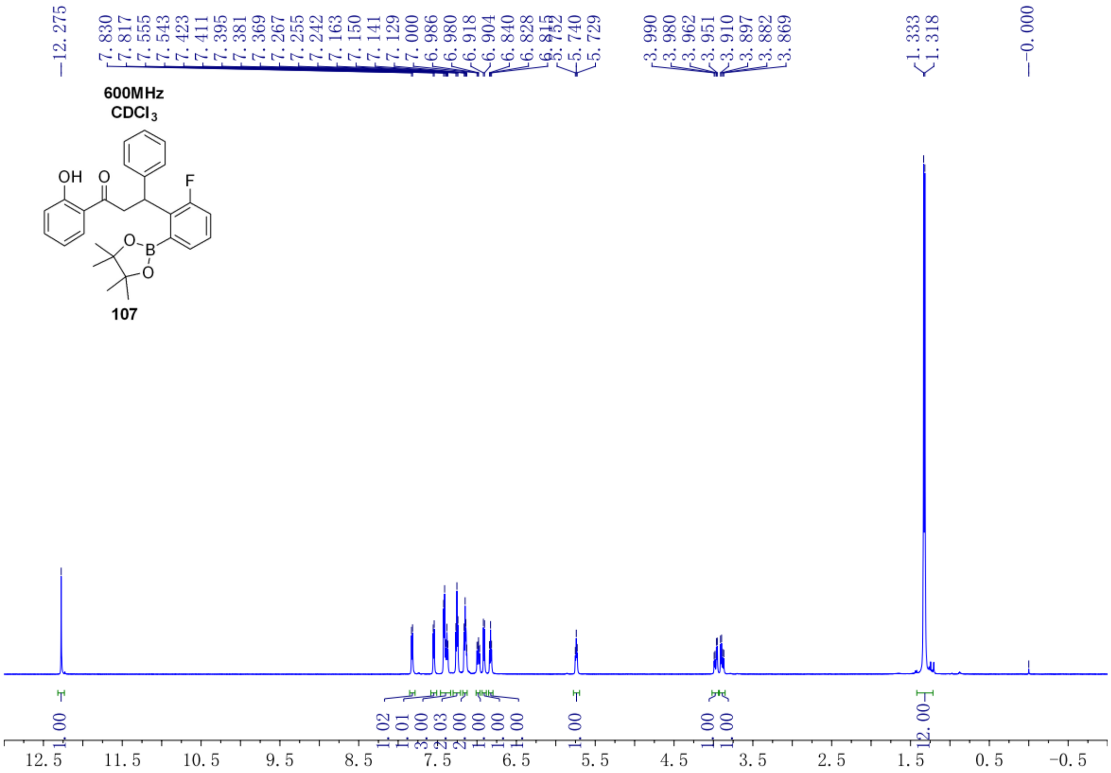
600MHz
CDCl3
OH
O
F
O
B
O
107

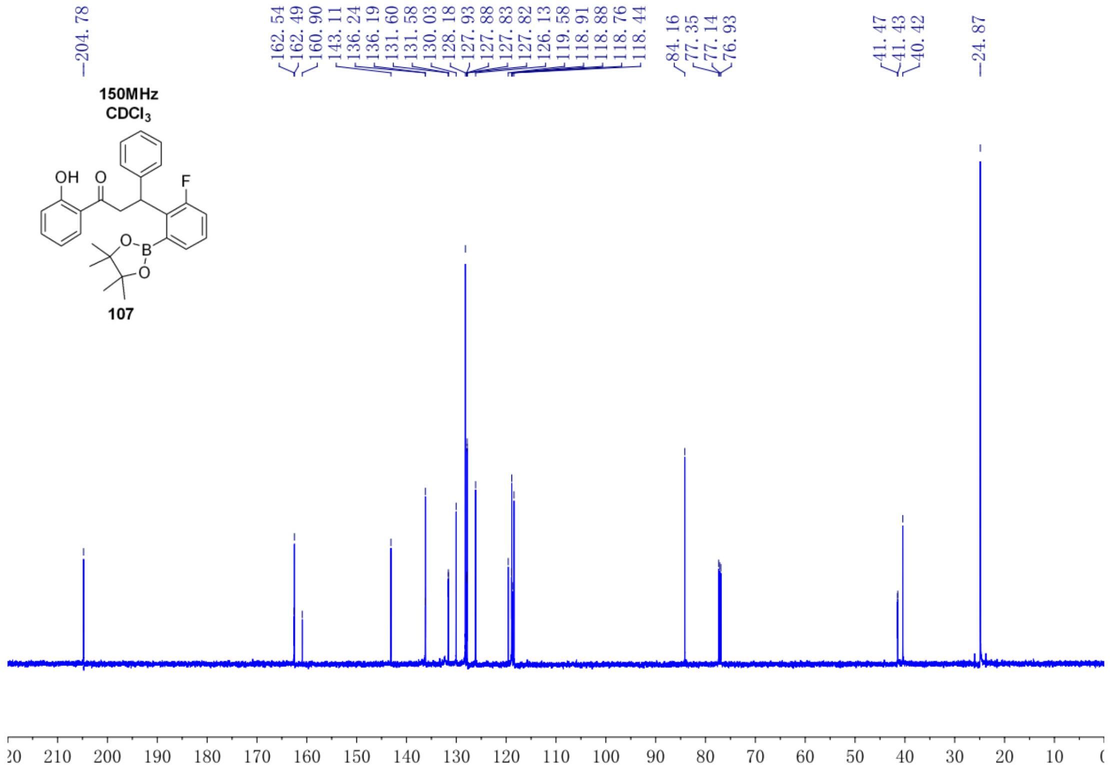
150MHz
CDCl3
OH
O
F
O
B
O
107

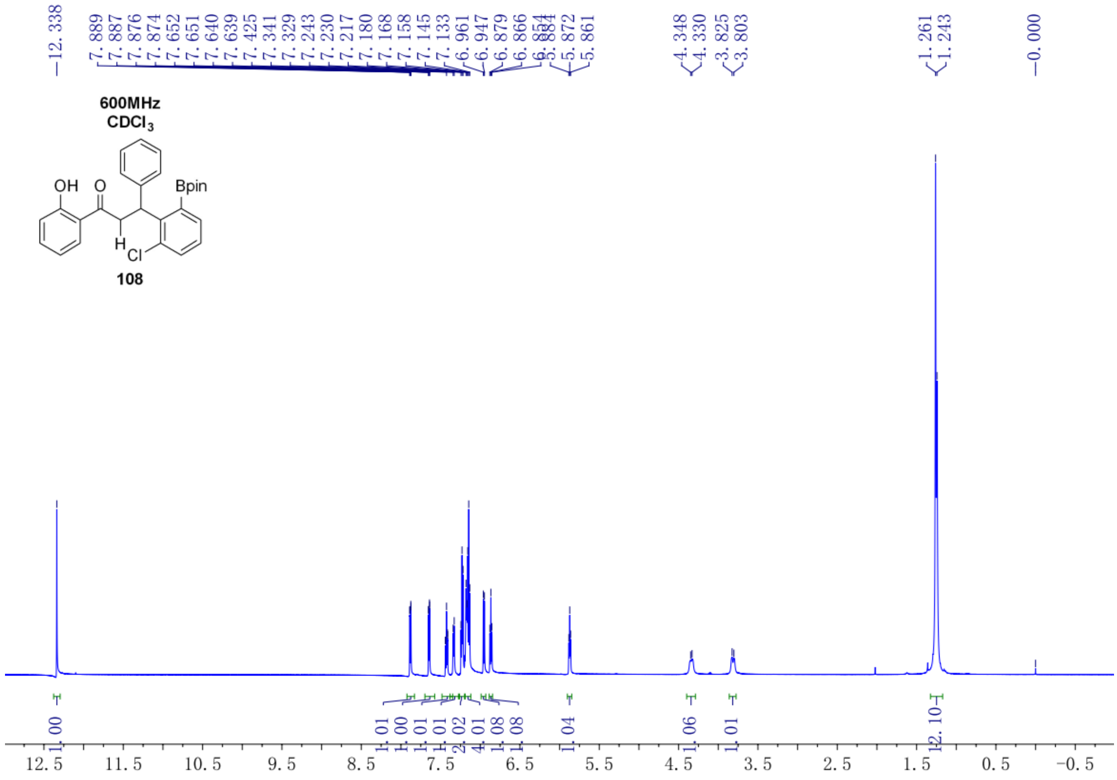
12.338
7.889 7.887 7.876 7.874 7.652 7.651 7.640 7.639 7.425 7.341 7.329 7.243 7.230 7.217 7.180 7.168 7.158 7.145 7.133 6.961 6.947 6.879 6.866 6.854 5.884 5.872 5.861
4.348 4.330 3.825 3.803
1.261 1.243
0.000
600MHz
CDCl3
OH O
Bpin
H
Cl
108
1.00
1.01 1.00 1.01 1.01 2.02 4.01 1.08 1.08
1.04
1.06
1.01
12.10
12.5 11.5 10.5 9.5 8.5 7.5 6.5 5.5 4.5 3.5 2.5 1.5 0.5 -0.5

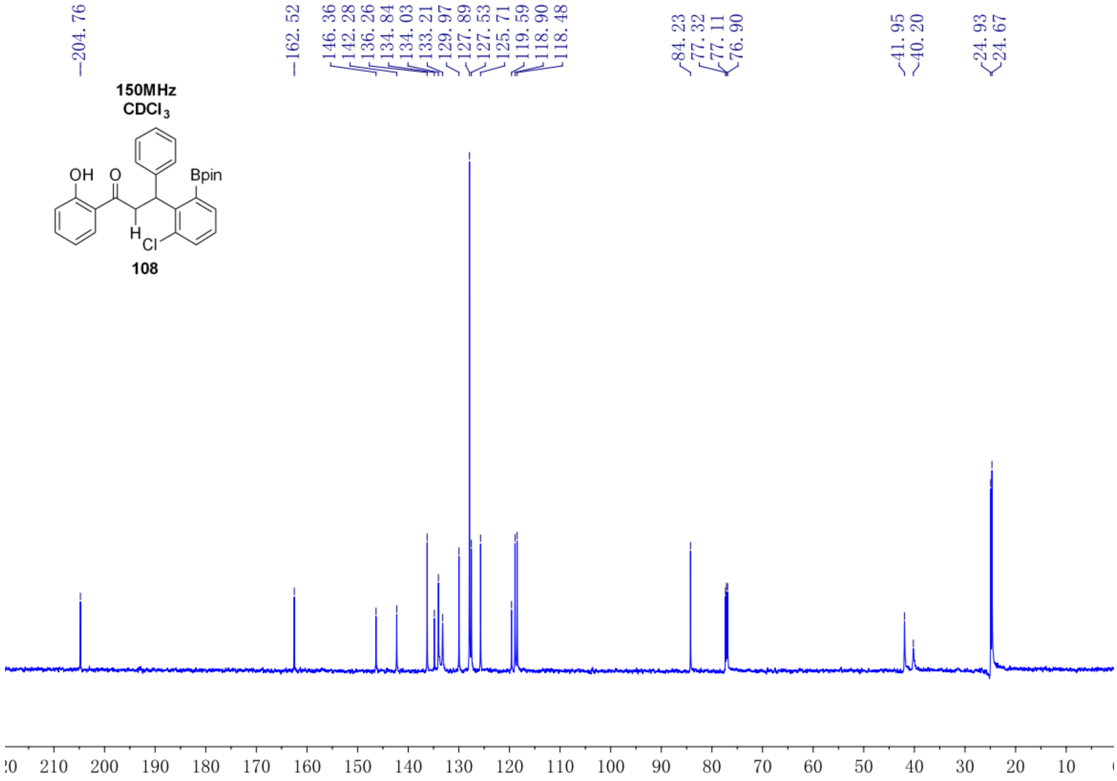
204.76
162.52
146.36 142.28 136.26 134.84 134.03 133.21 129.97 127.89 127.53 125.71 119.59 118.90 118.48
84.23 77.32 77.11 76.90
41.95 40.20
24.93 24.67
150MHz
CDCl3
OH O
Bpin
H
Cl
108
210 200 190 180 170 160 150 140 130 120 110 100 90 80 70 60 50 40 30 20 10

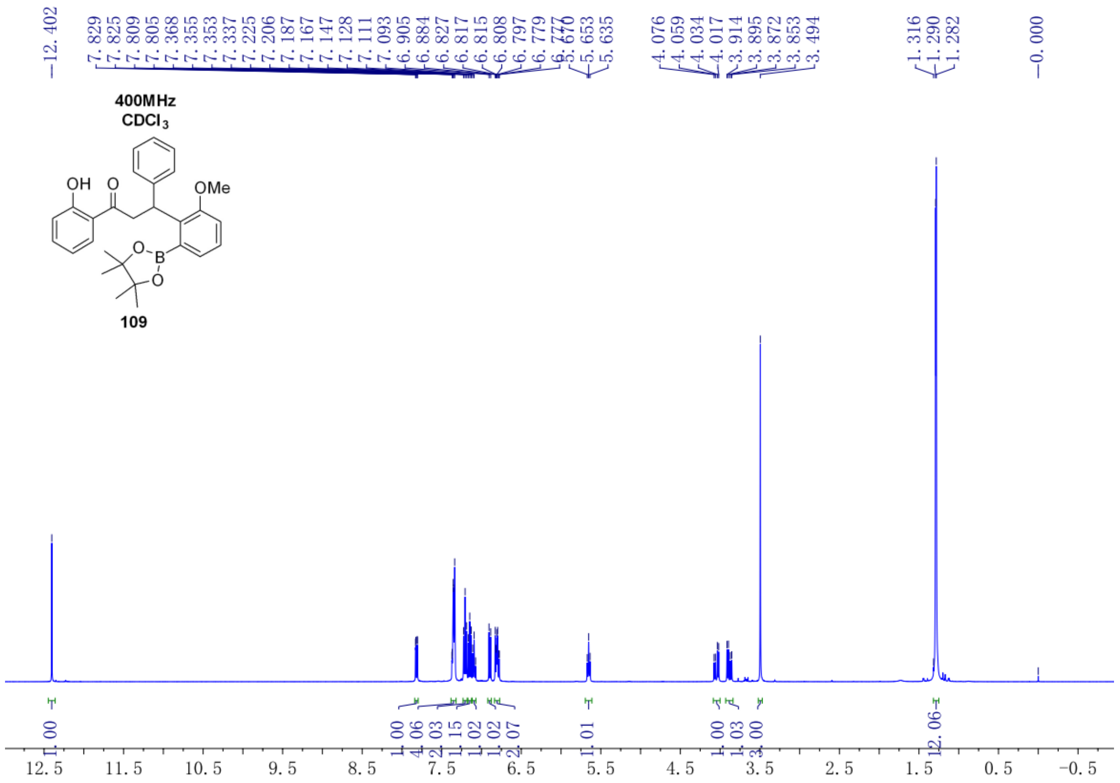
12.402
7.829 7.825 7.809 7.805 7.368 7.355 7.353 7.337 7.225 7.206 7.187 7.167 7.147 7.128 7.111 7.093 6.905 6.884 6.827 6.817 6.815 6.808 6.797 6.779
5.653 5.635
4.076 4.059 4.034 4.017 3.914 3.895 3.872 3.853 3.494
1.316 1.290 1.282
0.000
400MHz
CDCl3
OH
O
OMe
O
B
O
109
1.00
1.00
4.06
2.03
1.15
1.02
1.02
2.07
1.01
1.00
1.03
3.00
12.06
12.5 11.5 10.5 9.5 8.5 7.5 6.5 5.5 4.5 3.5 2.5 1.5 0.5 -0.5

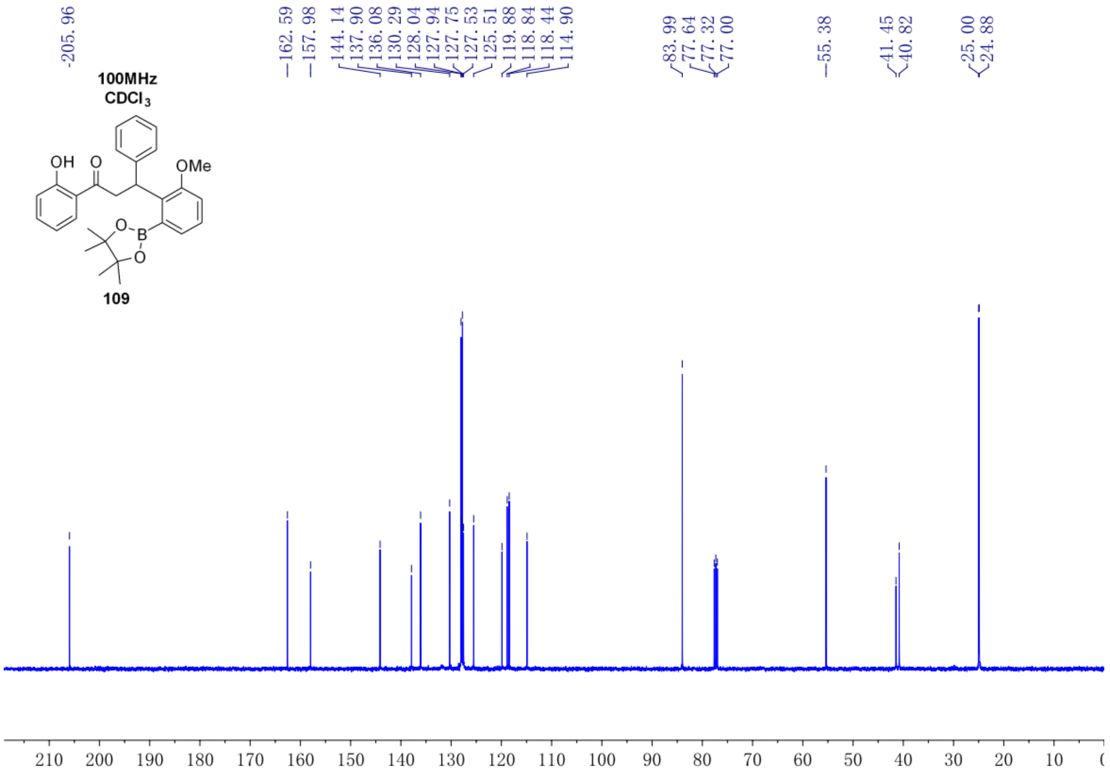
205.96
162.59 157.98 144.14 137.90 136.08 130.29 128.04 127.94 127.75 127.53 125.51 119.88 118.84 118.44 114.90
83.99 77.64 77.32 77.00
55.38
41.45 40.82
25.00 24.88
100MHz
CDCl3
OH
O
OMe
O
B
O
109
210 200 190 180 170 160 150 140 130 120 110 100 90 80 70 60 50 40 30 20 10

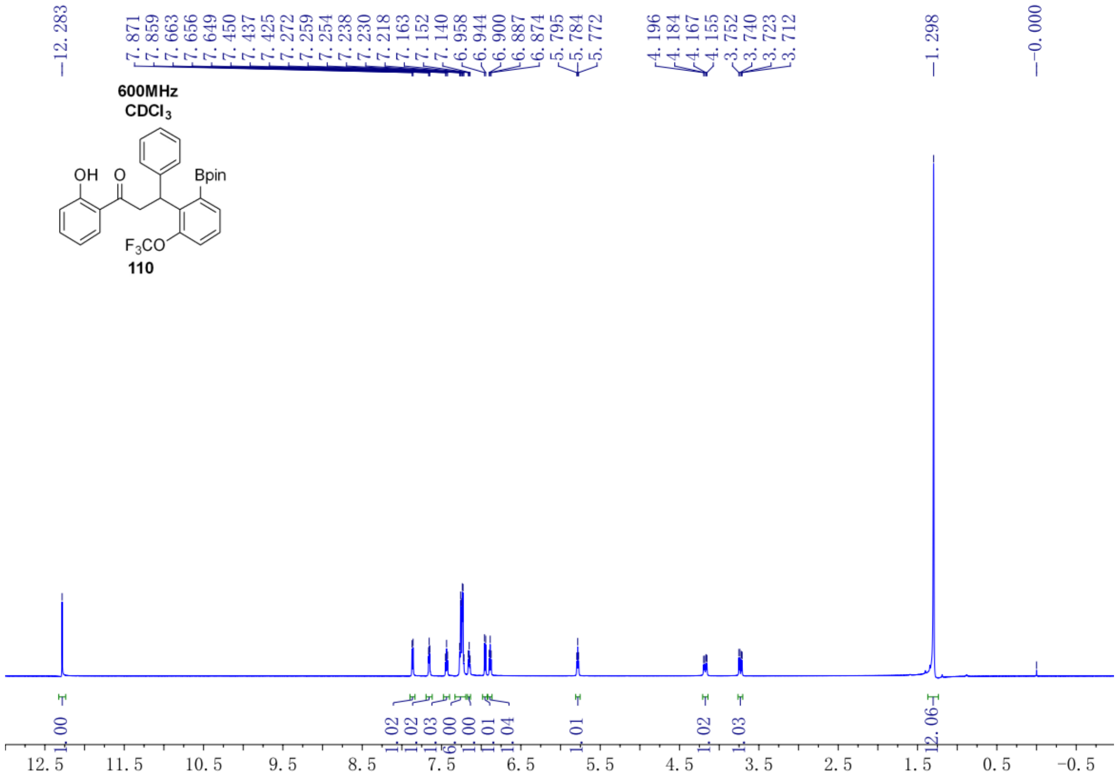
12.283
7.871 7.859 7.663 7.656 7.649 7.450 7.437 7.425 7.272 7.259 7.254 7.238 7.230 7.218 7.163 7.152 7.140 6.958 6.944 6.900 6.887 6.874 5.795 5.784 5.772
4.196 4.184 4.167 4.155 3.752 3.740 3.723 3.712
1.298
0.000
600MHz
CDCl3
OH O
Bpin
F3CO
110
1.00 1.02 1.02 1.03 6.00 1.00 1.01 1.04 1.01 1.02 1.03 12.06
12.5 11.5 10.5 9.5 8.5 7.5 6.5 5.5 4.5 3.5 2.5 1.5 0.5 -0.5

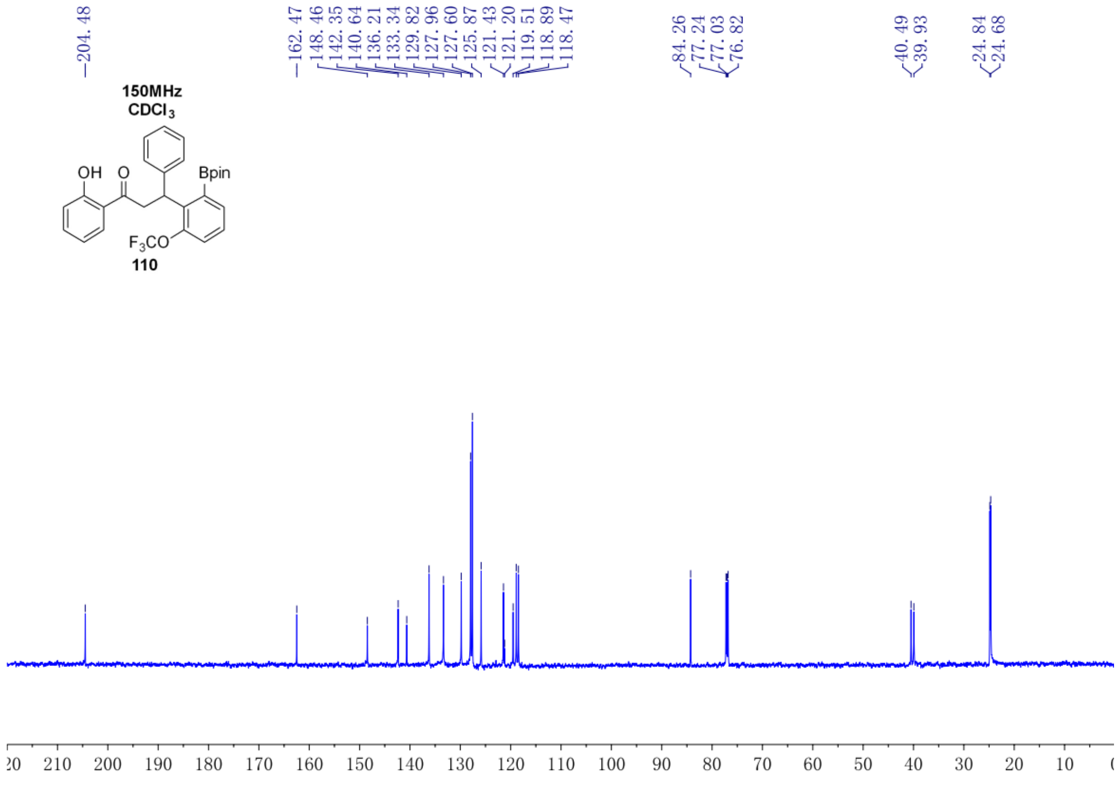
204.48
162.47 148.46 142.35 140.64 136.21 133.34 129.82 127.96 127.60 125.87 121.43 121.20 119.51 118.89 118.47
84.26 77.24 77.03 76.82
40.49 39.93
24.84 24.68
150MHz
CDCl3
OH O
Bpin
F3CO
110
210 200 190 180 170 160 150 140 130 120 110 100 90 80 70 60 50 40 30 20 10

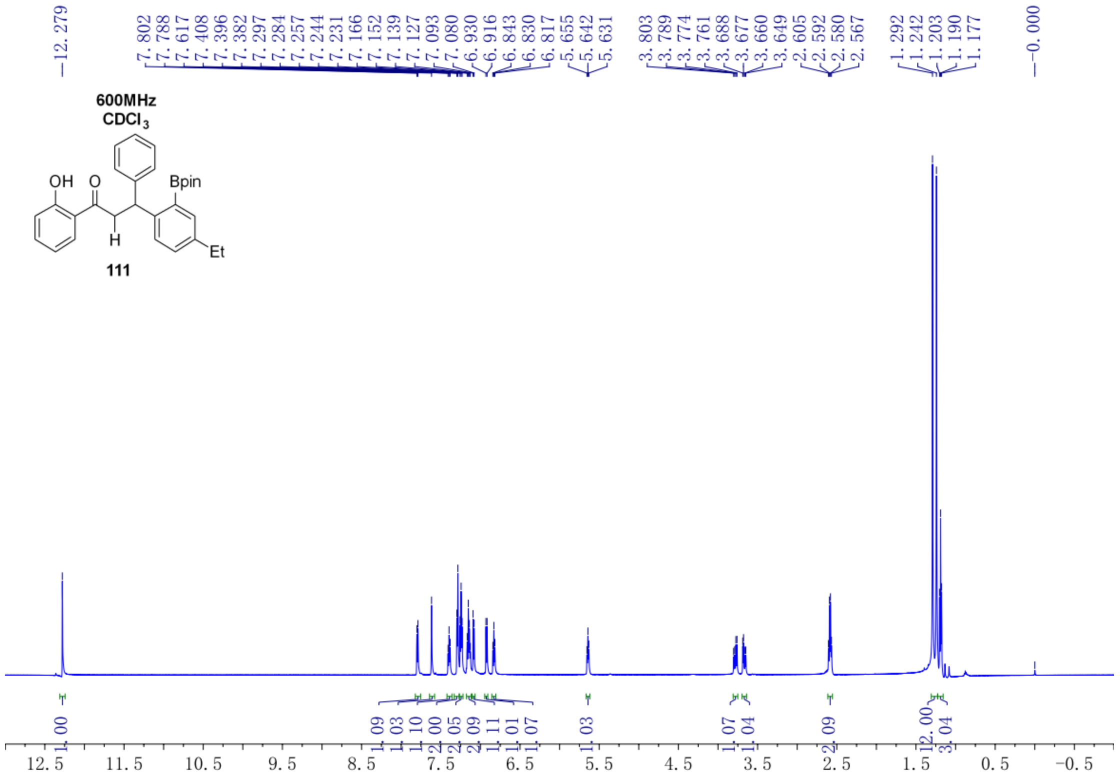
12.279
7.802 7.788 7.617 7.408 7.396 7.382 7.297 7.284 7.257 7.244 7.231 7.166 7.152 7.139 7.127 7.093 7.080 6.930 6.916 6.843 6.830 6.817 5.655 5.642 5.631
3.803 3.789 3.774 3.761 3.688 3.677 3.660 3.649 2.605 2.592 2.580 2.567
1.292 1.242 1.203 1.190 1.177
0.000
600MHz
CDCl3
OH
O
Bpin
H
Et
111
1.00
1.09 1.03 1.10 2.00 2.05 2.09 1.11 1.01 1.07
1.03
1.07 1.04
2.09
12.00 3.04
12.5 11.5 10.5 9.5 8.5 7.5 6.5 5.5 4.5 3.5 2.5 1.5 0.5 -0.5

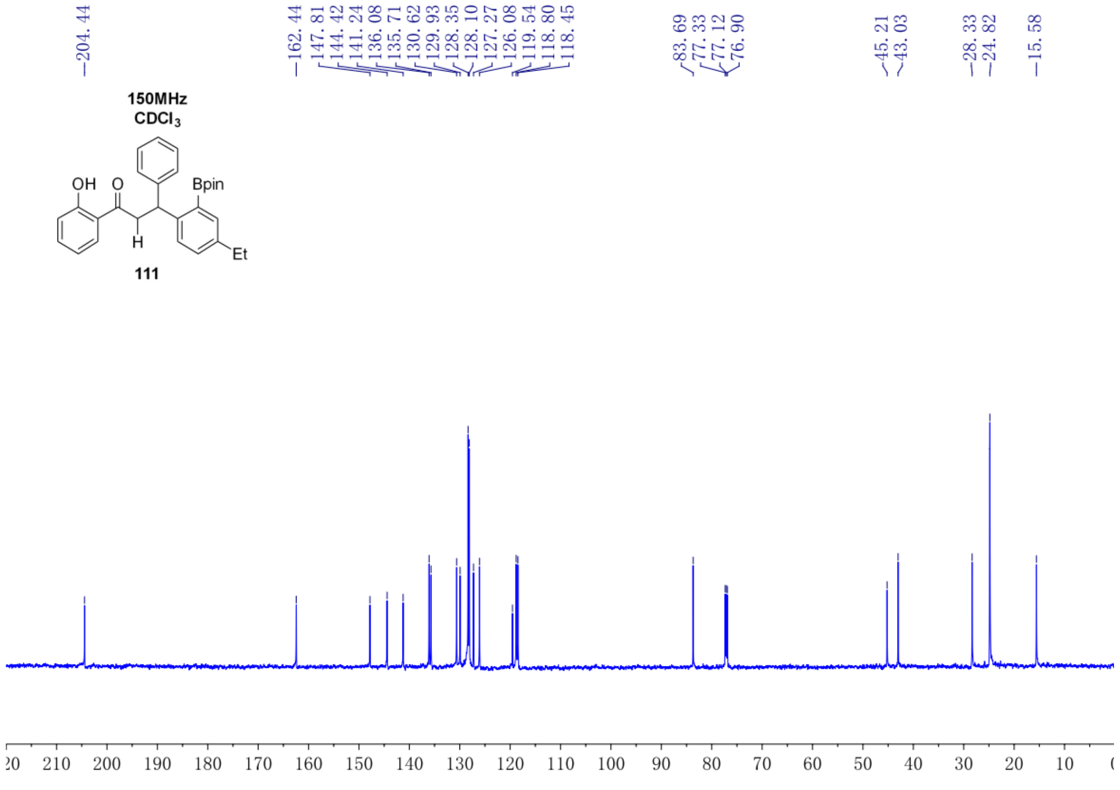
204.44
162.44 147.81 144.42 141.24 136.08 135.71 130.62 129.93 128.35 128.10 127.27 126.08 119.54 118.80 118.45
83.69 77.33 77.12 76.90
45.21 43.03
28.33 24.82
15.58
150MHz
CDCl3
OH
O
Bpin
H
Et
111
210 200 190 180 170 160 150 140 130 120 110 100 90 80 70 60 50 40 30 20 10

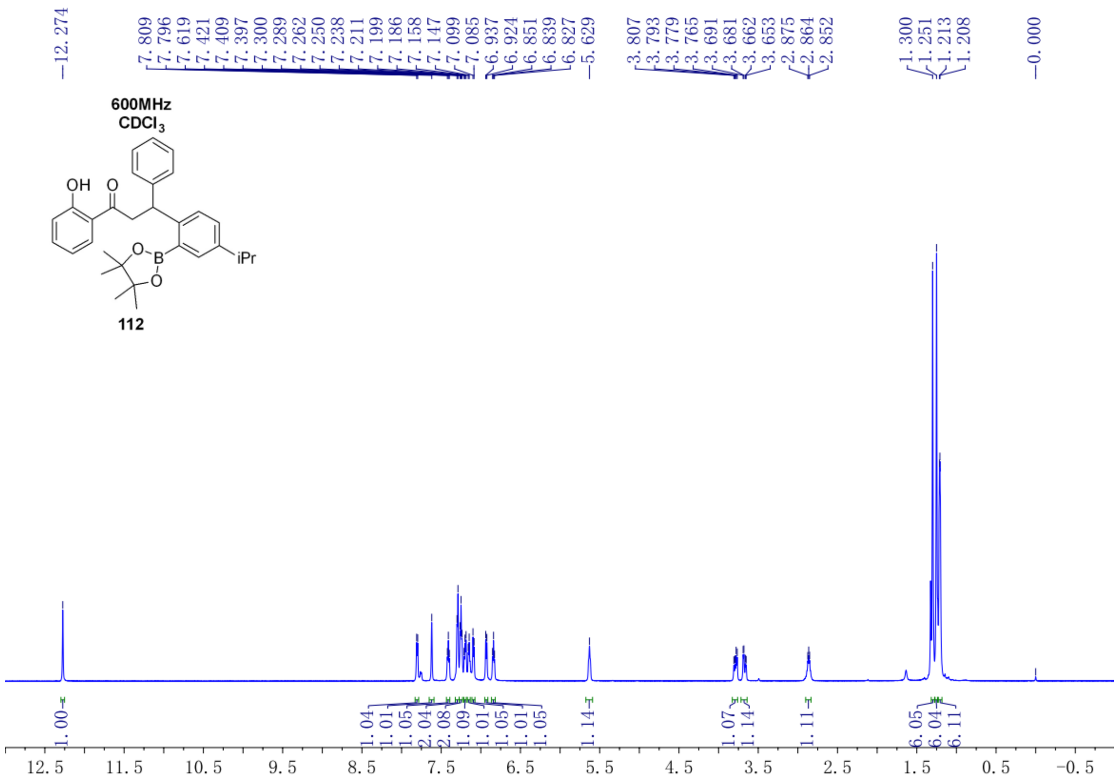

600MHz
CDCl3
OH O
iPr
O B O
112
12.274
7.809 7.796 7.619 7.421 7.409 7.397 7.300 7.289 7.262 7.250 7.238 7.211 7.199 7.186 7.158 7.147 7.099 7.085 6.937 6.924 6.851 6.839 6.827
5.629
3.807 3.793 3.779 3.765 3.691 3.681 3.662 3.653 2.875 2.864 2.852
1.300 1.251 1.213 1.208
0.000
1.00 1.04 1.01 1.05 2.04 2.08 1.09 1.01 1.05 1.01 1.05 1.14 1.07 1.14 1.11 6.05 6.04 6.11
12.5 11.5 10.5 9.5 8.5 7.5 6.5 5.5 4.5 3.5 2.5 1.5 0.5 -0.5

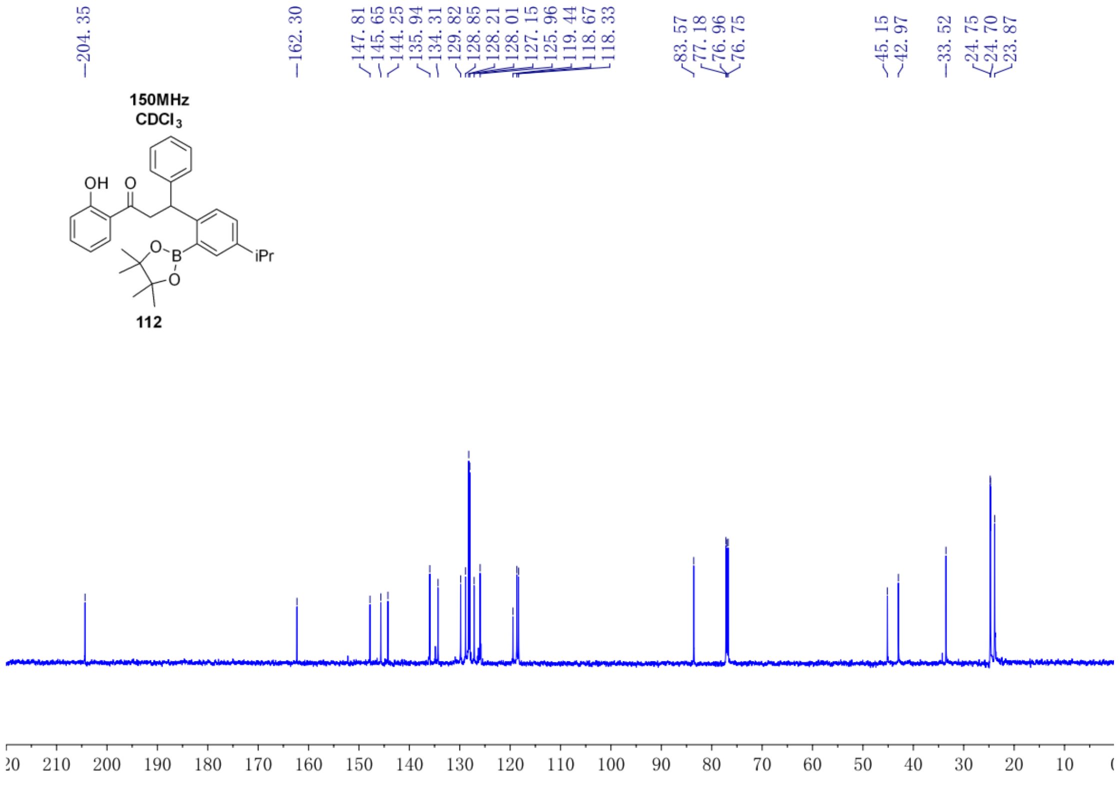

150MHz
CDCl3
OH O
iPr
O B O
112
204.35
162.30
147.81 145.65 144.25 135.94 134.31 129.82 128.85 128.21 128.01 127.15 125.96 119.44 118.67 118.33
83.57 77.18 76.96 76.75
45.15 42.97
33.52 24.75 24.70 23.87
210 200 190 180 170 160 150 140 130 120 110 100 90 80 70 60 50 40 30 20 10

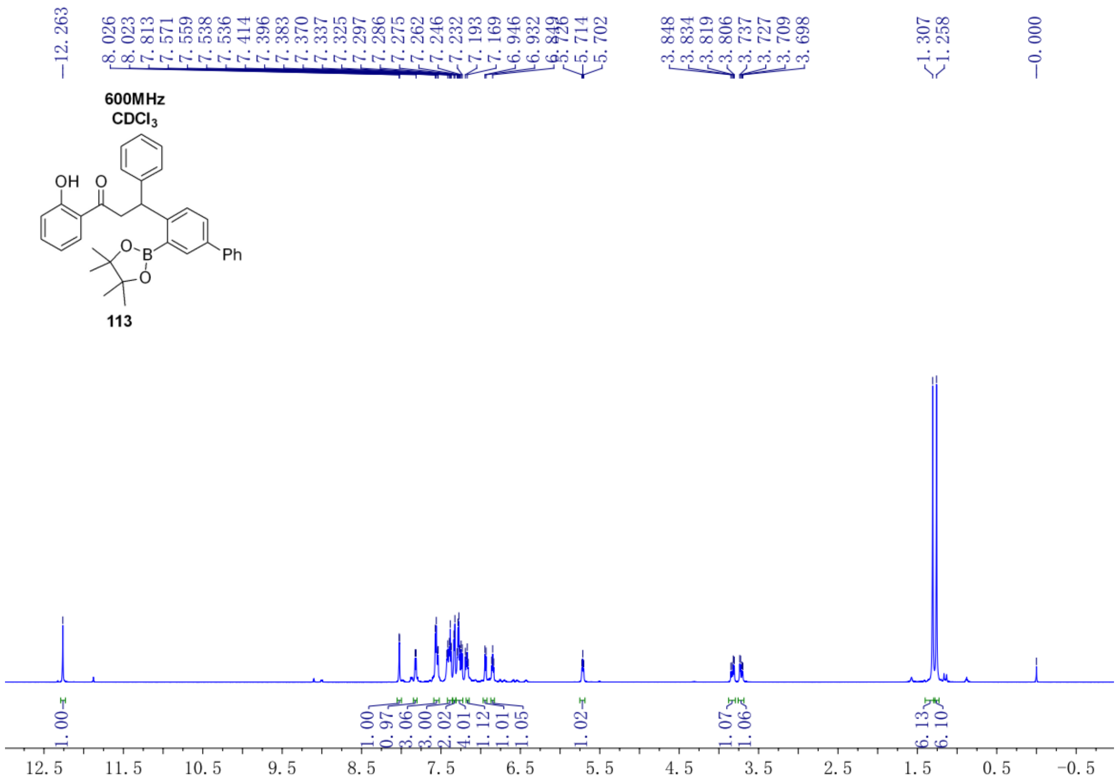
600MHz
CDCl3
113

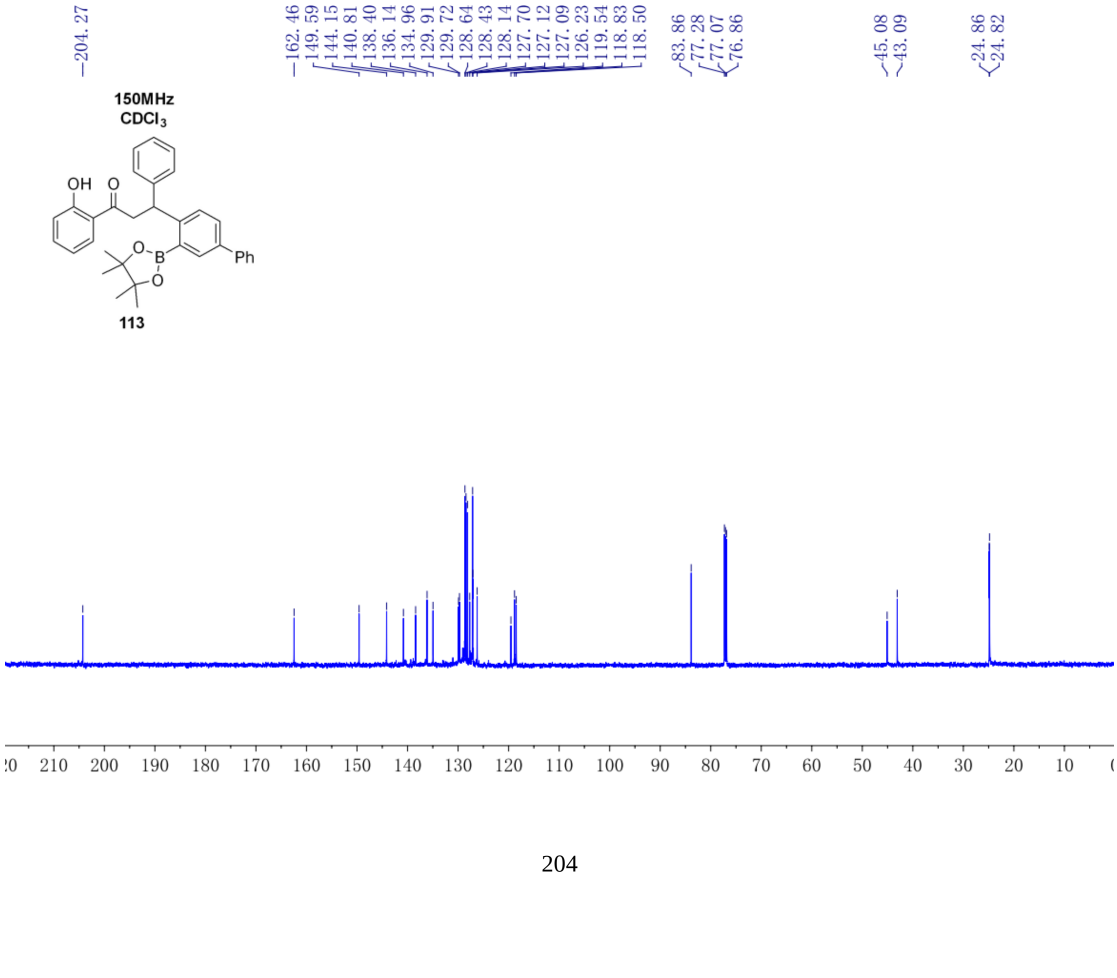
150MHz
CDCl3
113

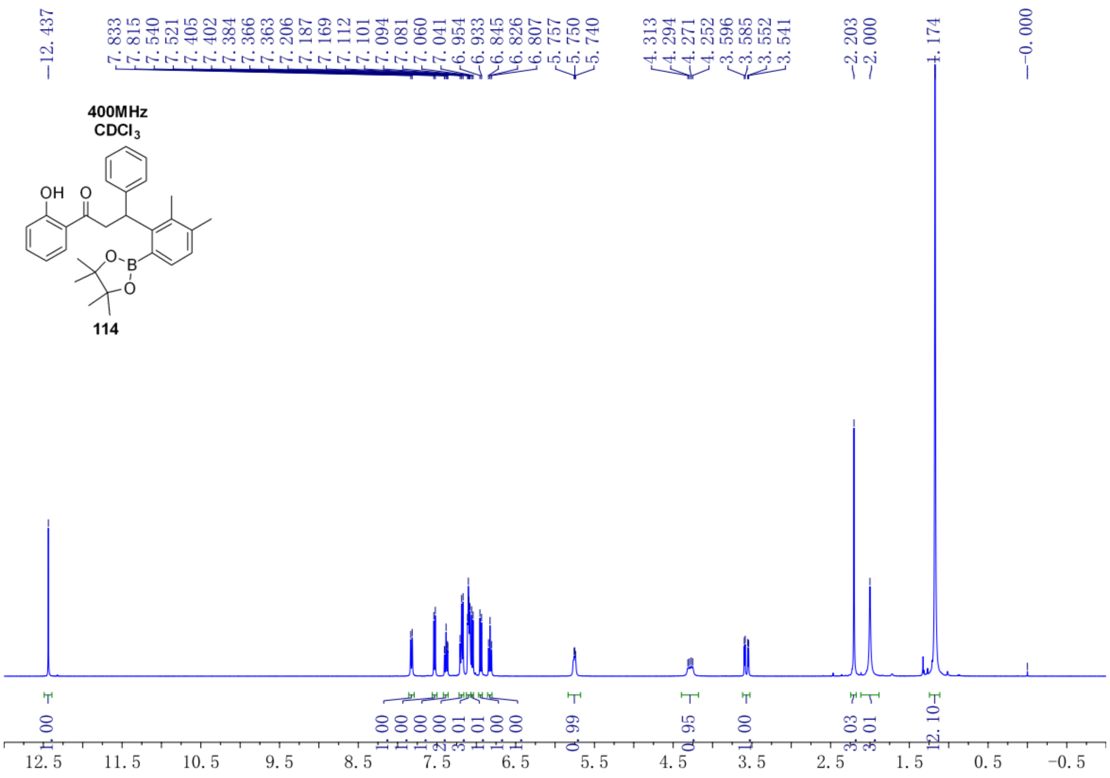
400MHz
CDCl3
OH
O
B
O
O
114
12.437
7.833 7.815 7.540 7.521 7.405 7.402 7.384 7.366 7.363 7.206 7.187 7.169 7.112 7.101 7.094 7.081 7.060 7.041 6.954 6.933 6.845 6.826 6.807
5.757 5.750 5.740
4.313 4.294 4.271 4.252 3.596 3.585 3.552 3.541
2.203 2.000
1.174
0.000
1.00 1.00 1.00 1.00 2.00 3.01 1.01 1.00 1.00 0.99 0.95 1.00 3.03 3.01 12.10
12.5 11.5 10.5 9.5 8.5 7.5 6.5 5.5 4.5 3.5 2.5 1.5 0.5 -0.5

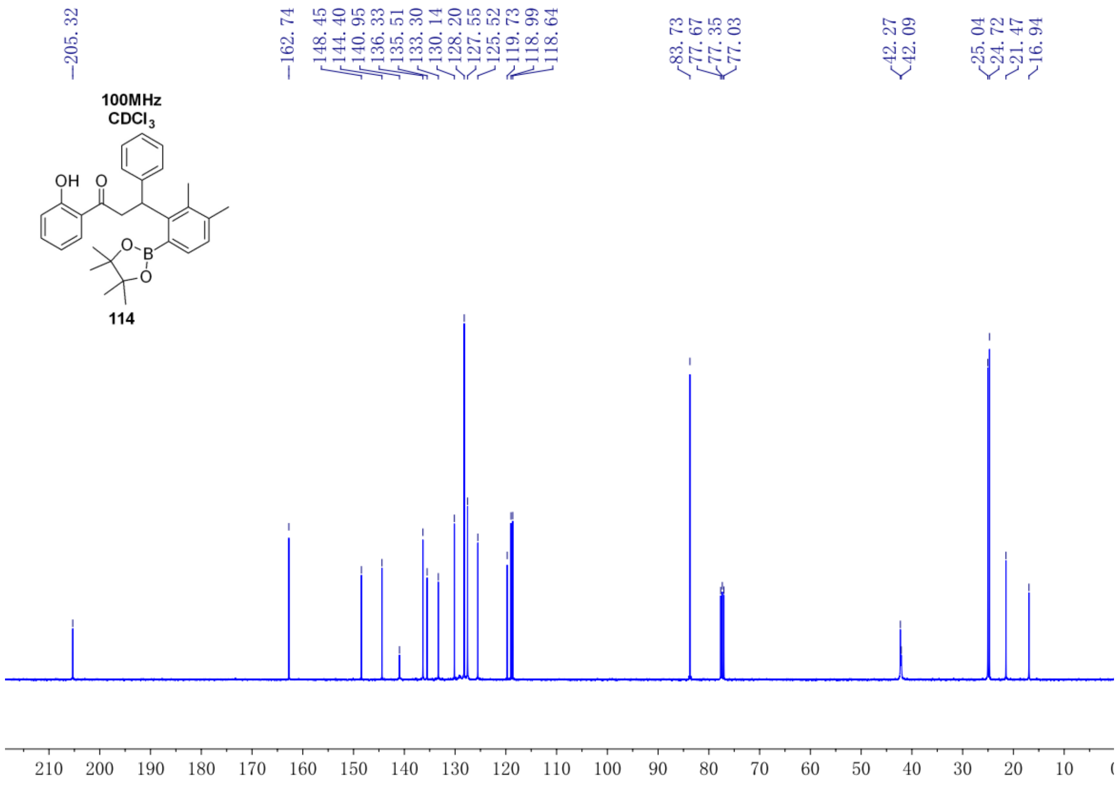
100MHz
CDCl3
OH
O
B
O
O
114
205.32
162.74
148.45 144.40 140.95 136.33 135.51 133.30 130.14 128.20 127.55 125.52 119.73 118.99 118.64
83.73 77.67 77.35 77.03
42.27 42.09
25.04 24.72 21.47 16.94
210 200 190 180 170 160 150 140 130 120 110 100 90 80 70 60 50 40 30 20 10

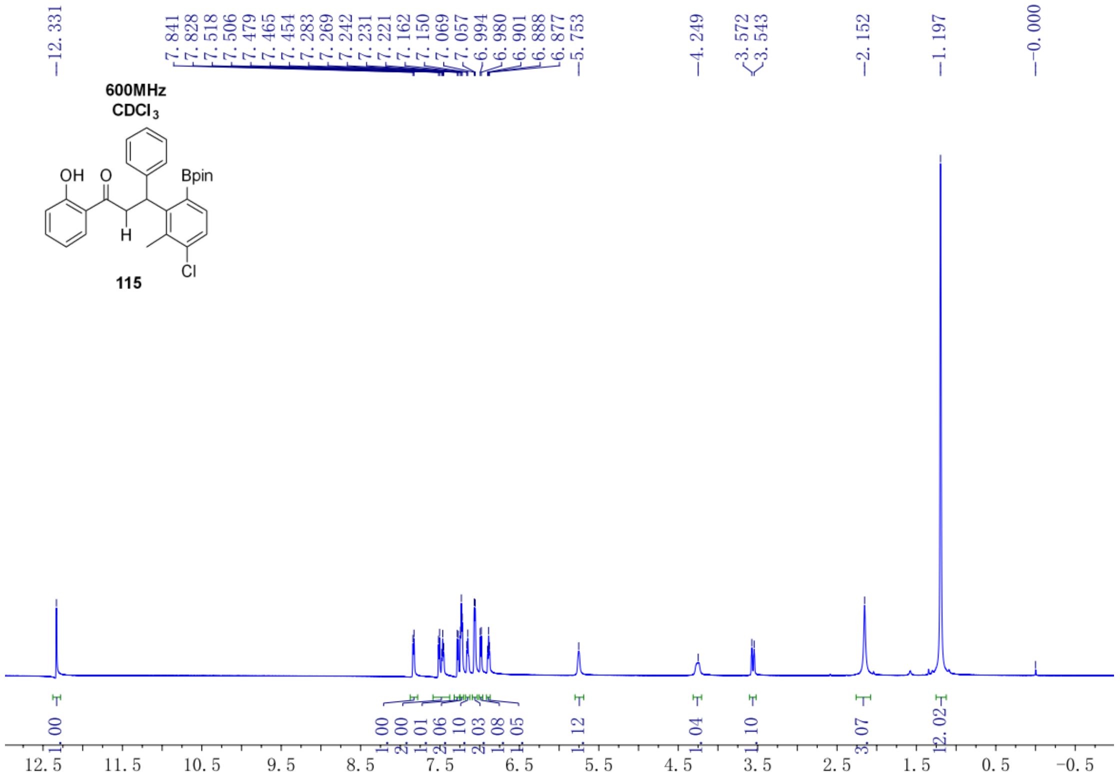
12.331
7.841 7.828 7.518 7.506 7.479 7.465 7.454 7.283 7.269 7.242 7.231 7.221 7.162 7.150 7.069 7.057 6.994 6.980 6.901 6.888 6.877 5.753
4.249
3.572 3.543
2.152
1.197
0.000
600MHz
CDCl3
OH
O
Bpin
H
Cl
115
1.00
1.00 2.00 1.01 2.06 1.10 2.03 1.08 1.05
1.12
1.04
1.10
3.07
12.02
12.5 11.5 10.5 9.5 8.5 7.5 6.5 5.5 4.5 3.5 2.5 1.5 0.5 -0.5

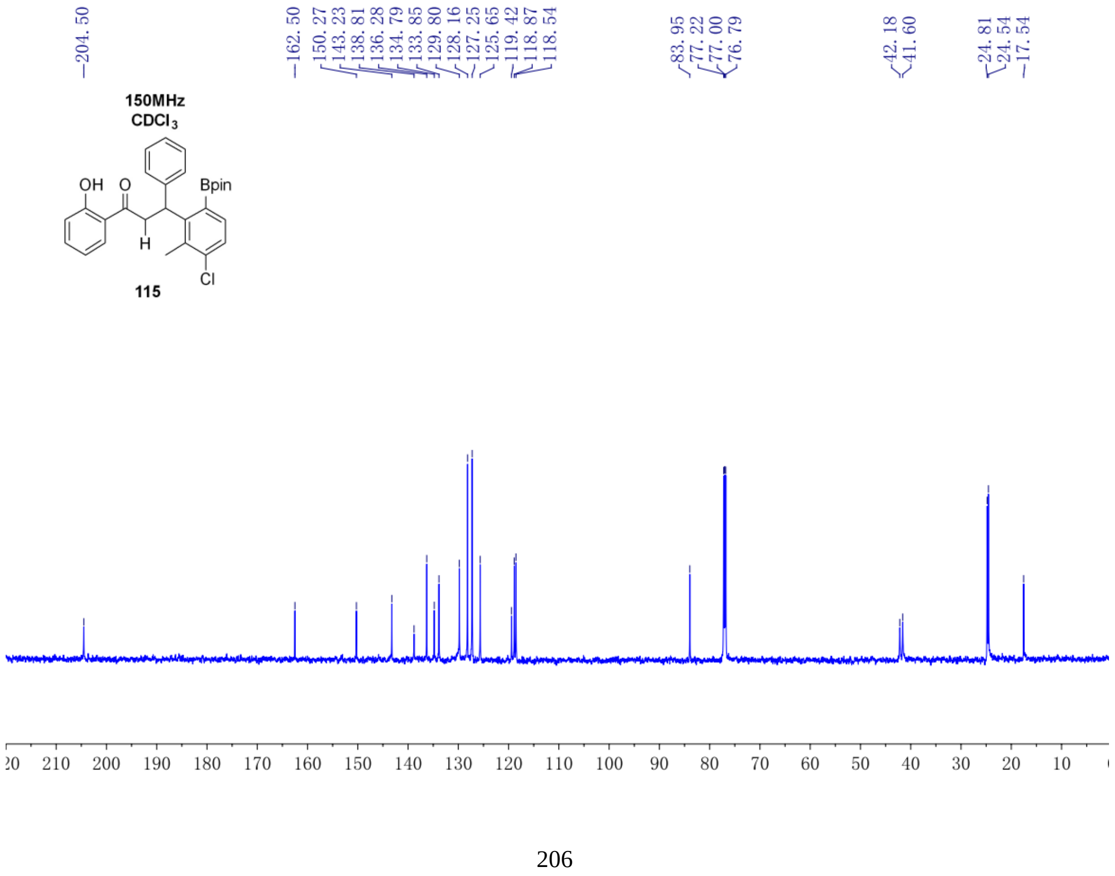
204.50
162.50 150.27 143.23 138.81 136.28 134.79 133.85 129.80 128.16 127.25 125.65 119.42 118.87 118.54
83.95 77.22 77.00 76.79
42.18 41.60
24.81 24.54 17.54
150MHz
CDCl3
OH
O
Bpin
H
Cl
115
210 200 190 180 170 160 150 140 130 120 110 100 90 80 70 60 50 40 30 20 10

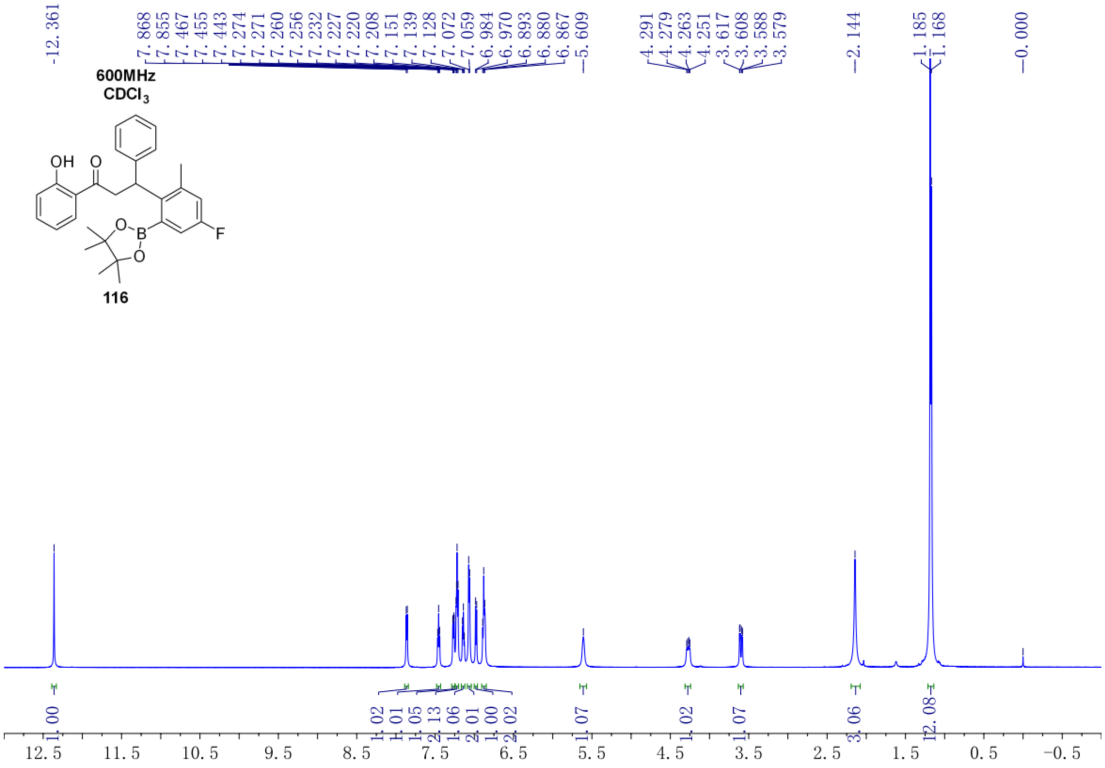

600MHz
CDCl3
OH
O
B
O
O
F
116
-12.361
7.868 7.855 7.467 7.455 7.443 7.274 7.271 7.260 7.256 7.232 7.227 7.220 7.208 7.151 7.139 7.128 7.072 7.059 6.984 6.970 6.893 6.880 6.867 5.609
4.291 4.279 4.263 4.251 3.617 3.608 3.588 3.579
2.144
1.185 1.168
0.000
1.00
1.02 1.01 1.05 2.13 1.06 2.01 1.00 2.02
1.07
1.02
1.07
3.06
12.08
12.5 11.5 10.5 9.5 8.5 7.5 6.5 5.5 4.5 3.5 2.5 1.5 0.5 -0.5

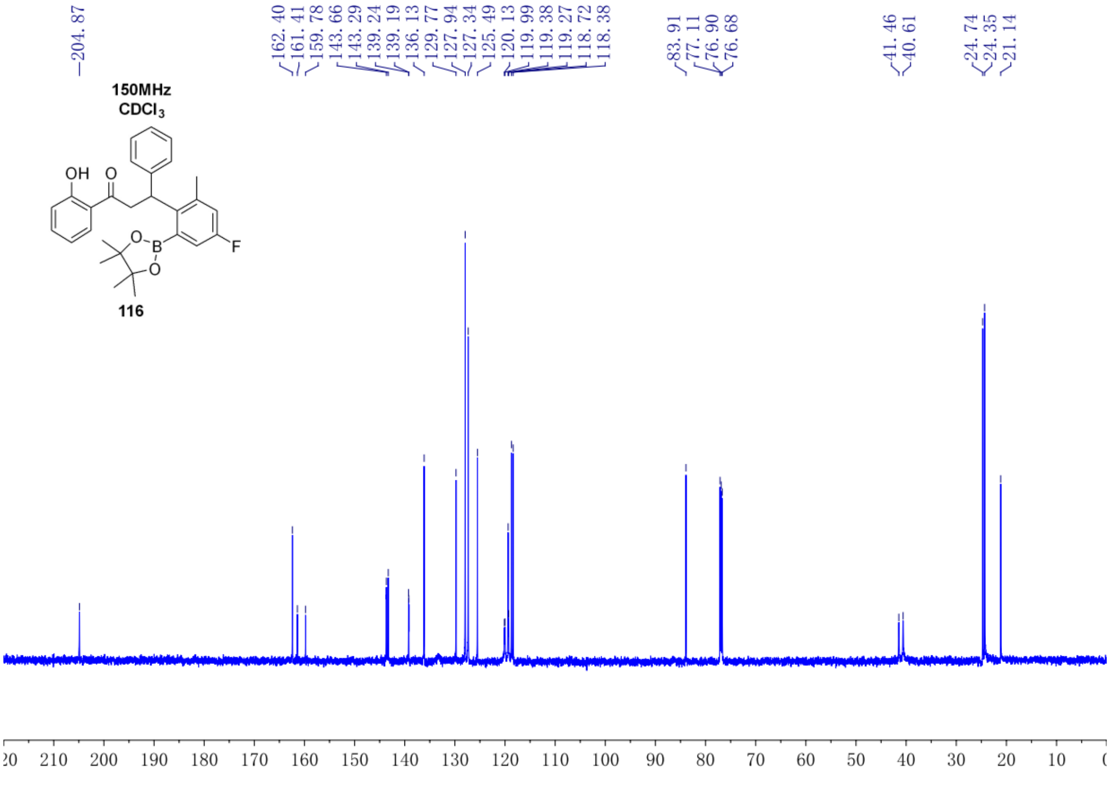

150MHz
CDCl3
OH
O
B
O
O
F
116
204.87
162.40 161.41 159.78 143.66 143.29 139.24 139.19 136.13 129.77 127.94 127.34 125.49 120.13 119.99 119.38 119.27 118.72 118.38
83.91 77.11 76.90 76.68
41.46 40.61
24.74 24.35 21.14
210 200 190 180 170 160 150 140 130 120 110 100 90 80 70 60 50 40 30 20 10

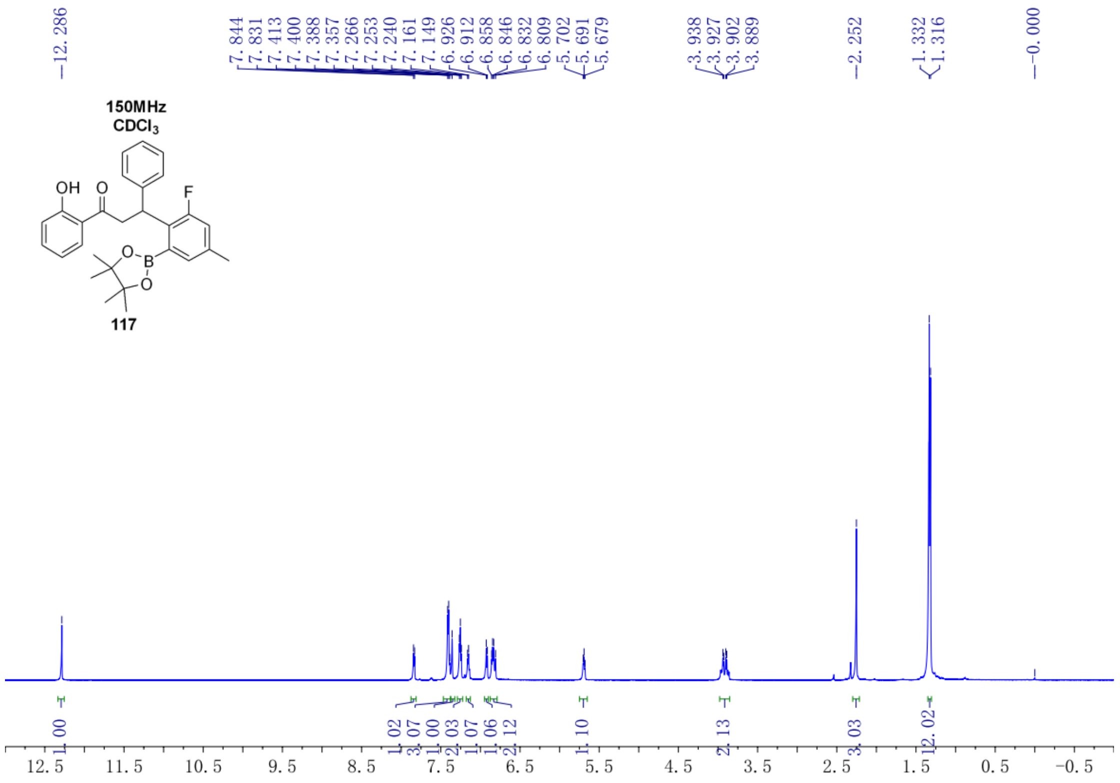
12.286
7.844
7.831
7.413
7.400
7.388
7.357
7.266
7.253
7.240
7.161
7.149
6.926
6.912
6.858
6.846
6.832
6.809
5.702
5.691
5.679
3.938
3.927
3.902
3.889
2.252
1.332
1.316
0.000
150MHz
$CDCl_3$
OH
O
F
O
B
O
117
1.00
1.02
3.07
1.00
2.03
1.07
1.06
2.12
1.10
2.13
3.03
12.02
12.5
11.5
10.5
9.5
8.5
7.5
6.5
5.5
4.5
3.5
2.5
1.5
0.5
-0.5

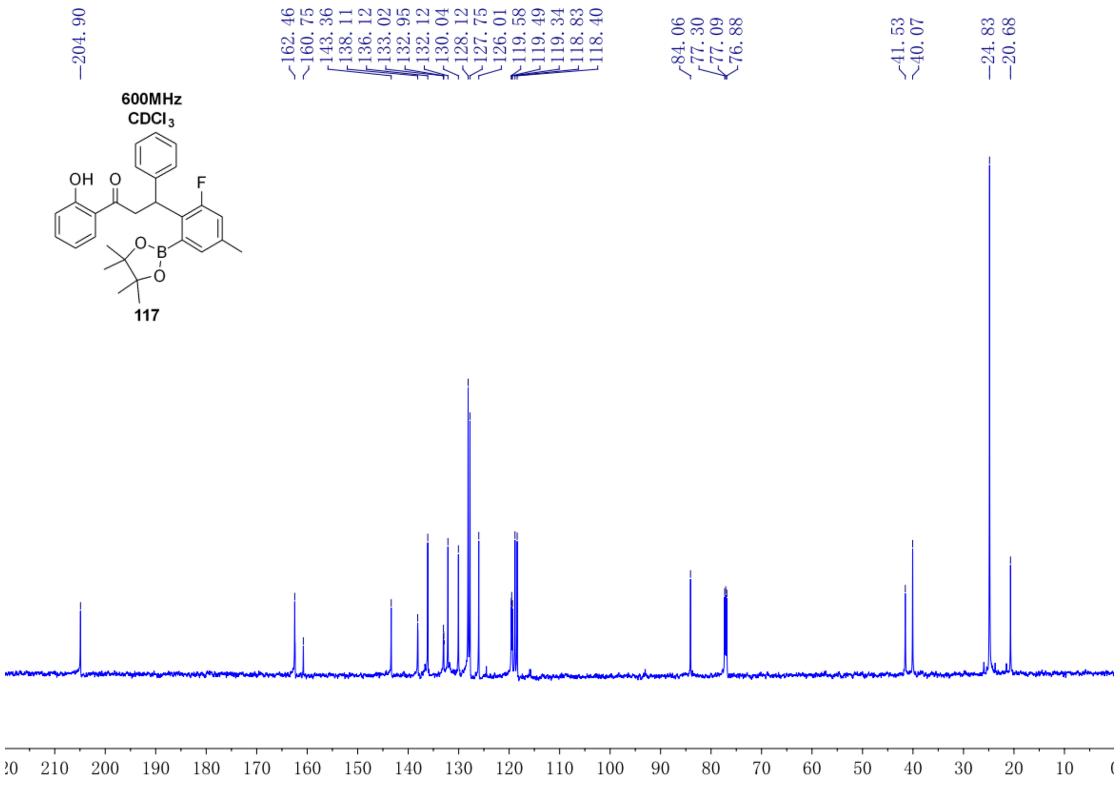
204.90
162.46
160.75
143.36
138.11
136.12
133.02
132.95
132.12
130.04
128.12
127.75
126.01
119.58
119.49
119.34
118.83
118.40
84.06
77.30
77.09
76.88
41.53
40.07
24.83
20.68
600MHz
$CDCl_3$
OH
O
F
O
B
O
117
210
200
190
180
170
160
150
140
130
120
110
100
90
80
70
60
50
40
30
20
10

600MHz
CDCl3

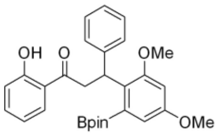

118

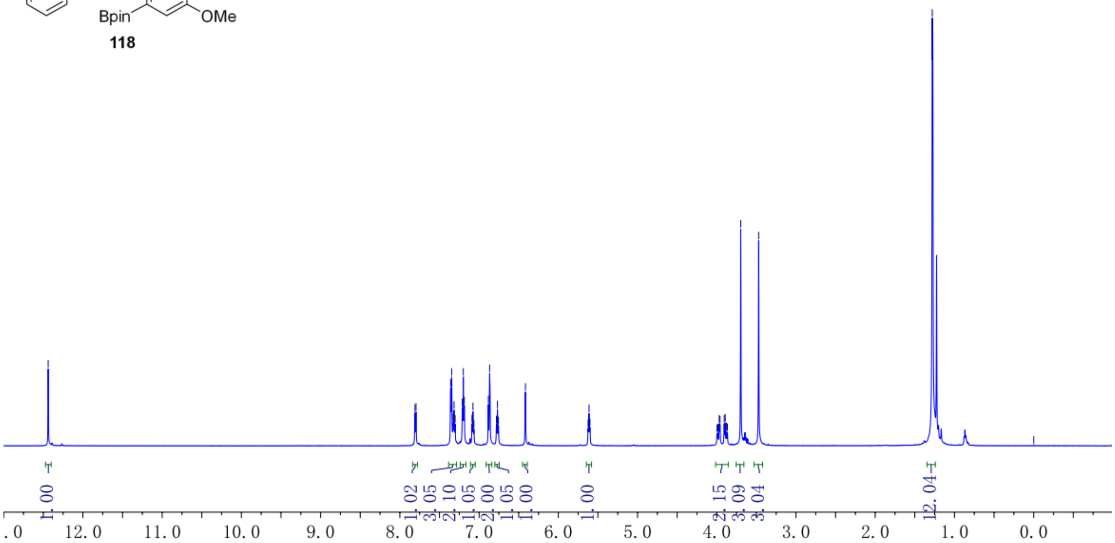

150MHz
CDCl3

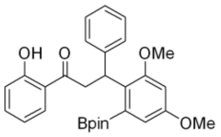

118

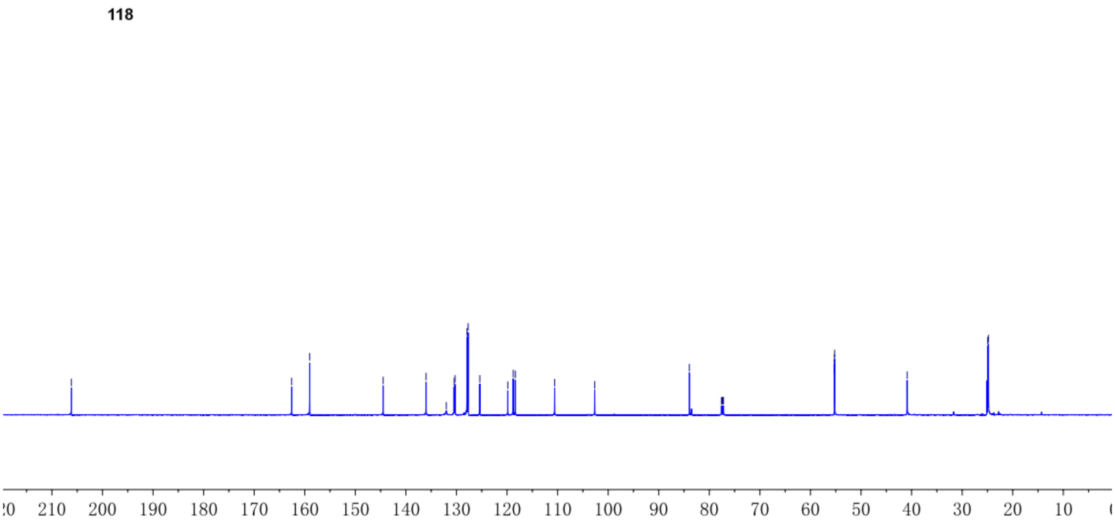

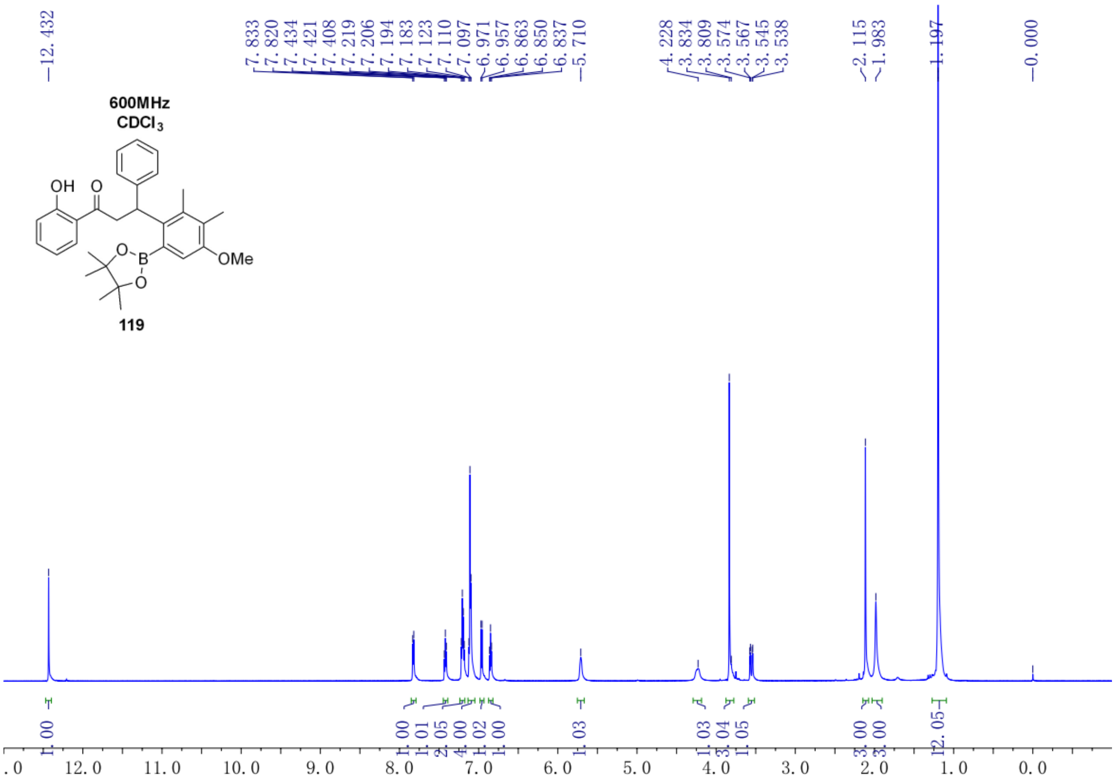
600MHz
CDCl$_3$
OH
O
B
O
O
OMe
119
12.432
7.833
7.820
7.434
7.421
7.408
7.219
7.206
7.194
7.183
7.123
7.110
7.097
6.971
6.957
6.863
6.850
6.837
5.710
4.228
3.834
3.809
3.574
3.567
3.545
3.538
2.115
1.983
1.197
0.000
1.00
1.00
1.01
2.05
4.00
1.02
1.00
1.03
1.03
3.04
1.05
3.00
3.00
12.05

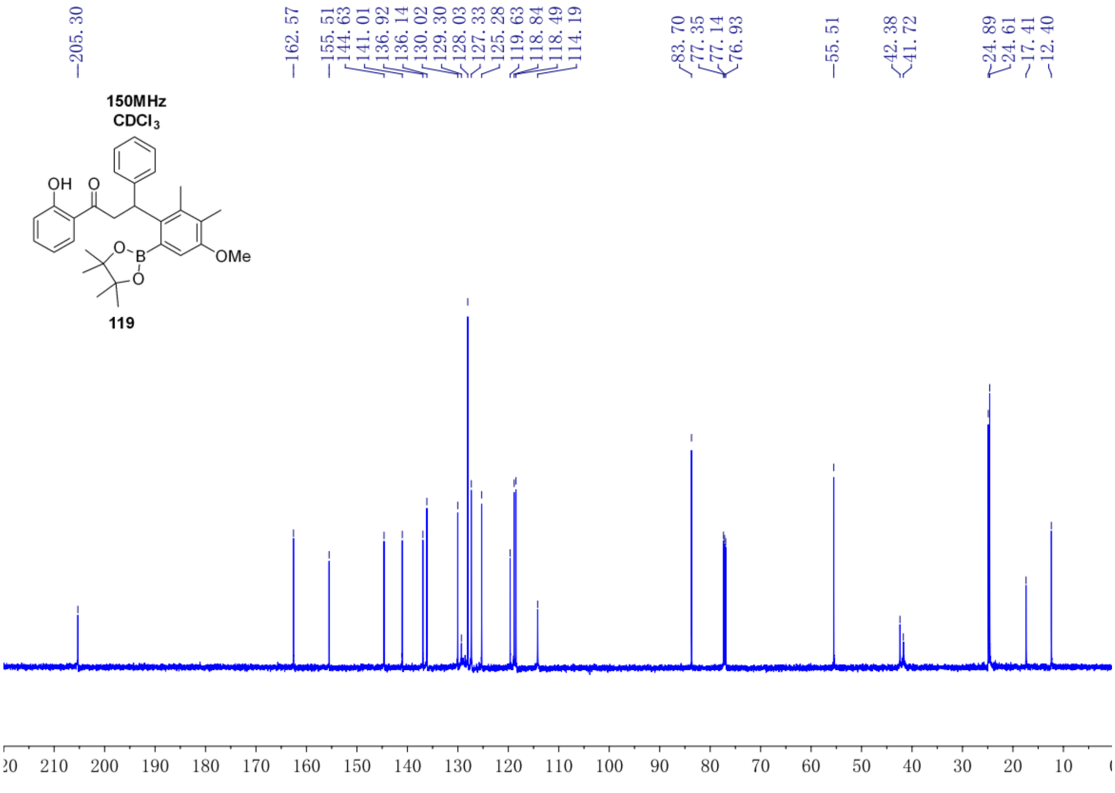
150MHz
CDCl$_3$
OH
O
B
O
O
OMe
119
205.30
162.57
155.51
144.63
141.01
136.92
136.14
130.02
129.30
128.03
127.33
125.28
119.63
118.84
118.49
114.19
83.70
77.35
77.14
76.93
55.51
42.38
41.72
24.89
24.61
17.41
12.40

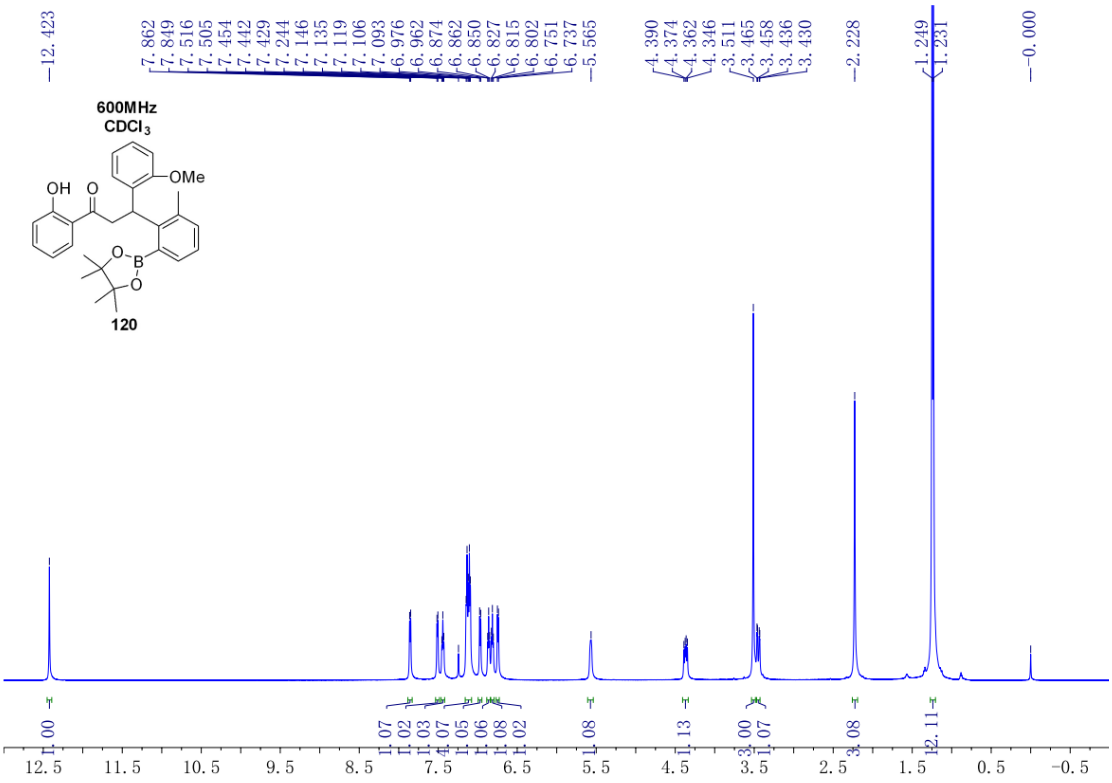
600MHz
CDCl3
OH
O
OMe
O
B
O
120
12.423
7.862 7.849 7.516 7.505 7.454 7.442 7.429 7.244 7.146 7.135 7.119 7.106 7.093 6.976 6.962 6.874 6.862 6.850 6.827 6.815 6.802 6.751 6.737
5.565
4.390 4.374 4.362 4.346 3.511 3.465 3.458 3.436 3.430
2.228
1.249 1.231
0.000
1.00 1.07 1.02 1.03 4.07 1.05 1.06 1.08 1.02 1.08 1.13 3.00 1.07 3.08 12.11
12.5 11.5 10.5 9.5 8.5 7.5 6.5 5.5 4.5 3.5 2.5 1.5 0.5 -0.5

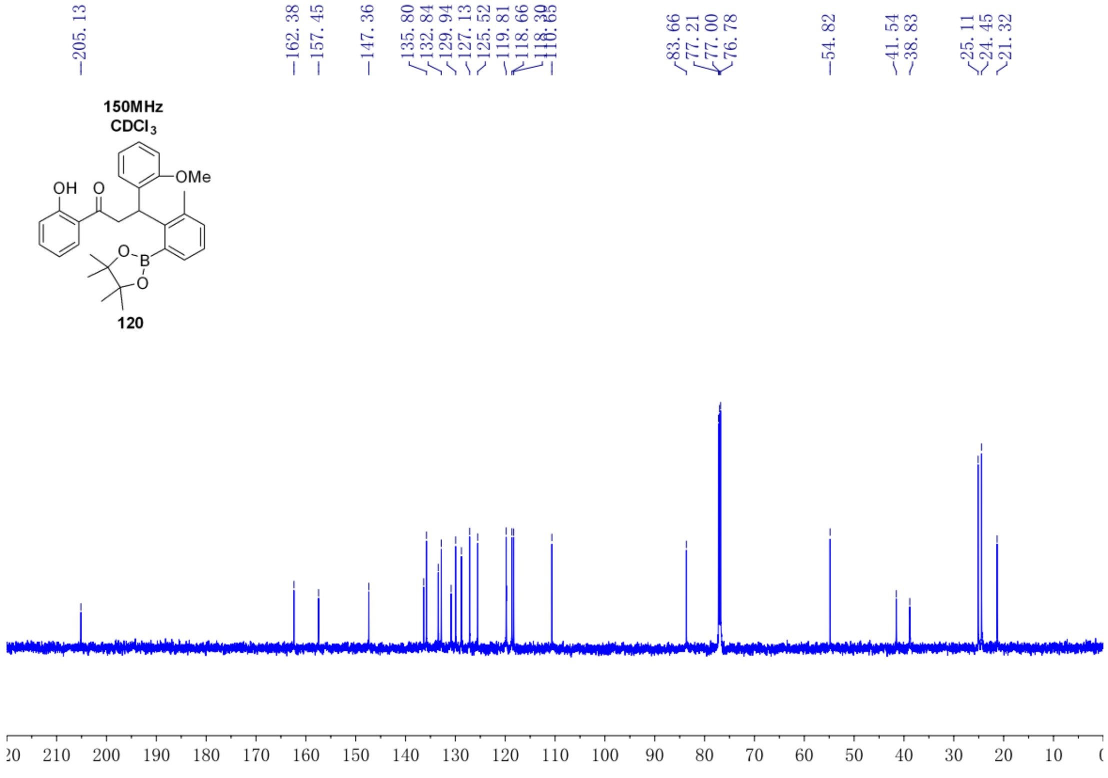
150MHz
CDCl3
OH
O
OMe
O
B
O
120
205.13
162.38 157.45 147.36 135.80 132.84 129.94 127.13 125.52 119.81 118.66
83.66 77.21 77.00 76.78
54.82
41.54 38.83
25.11 24.45 21.32
210 200 190 180 170 160 150 140 130 120 110 100 90 80 70 60 50 40 30 20 10

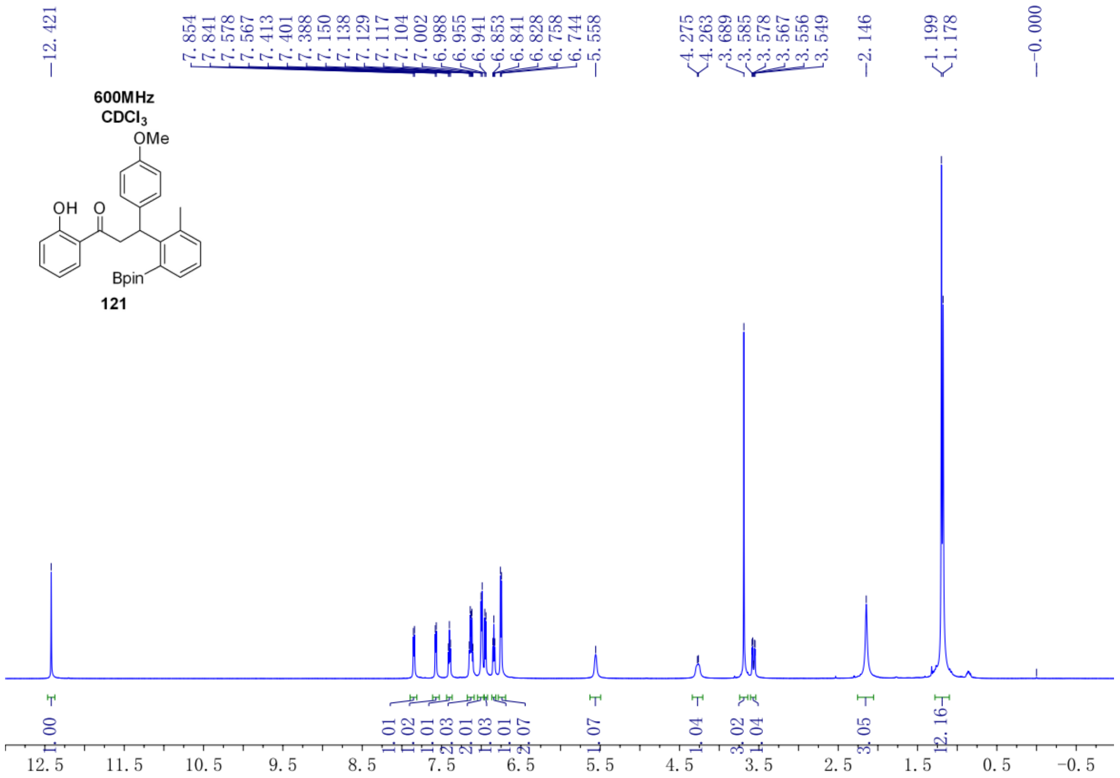

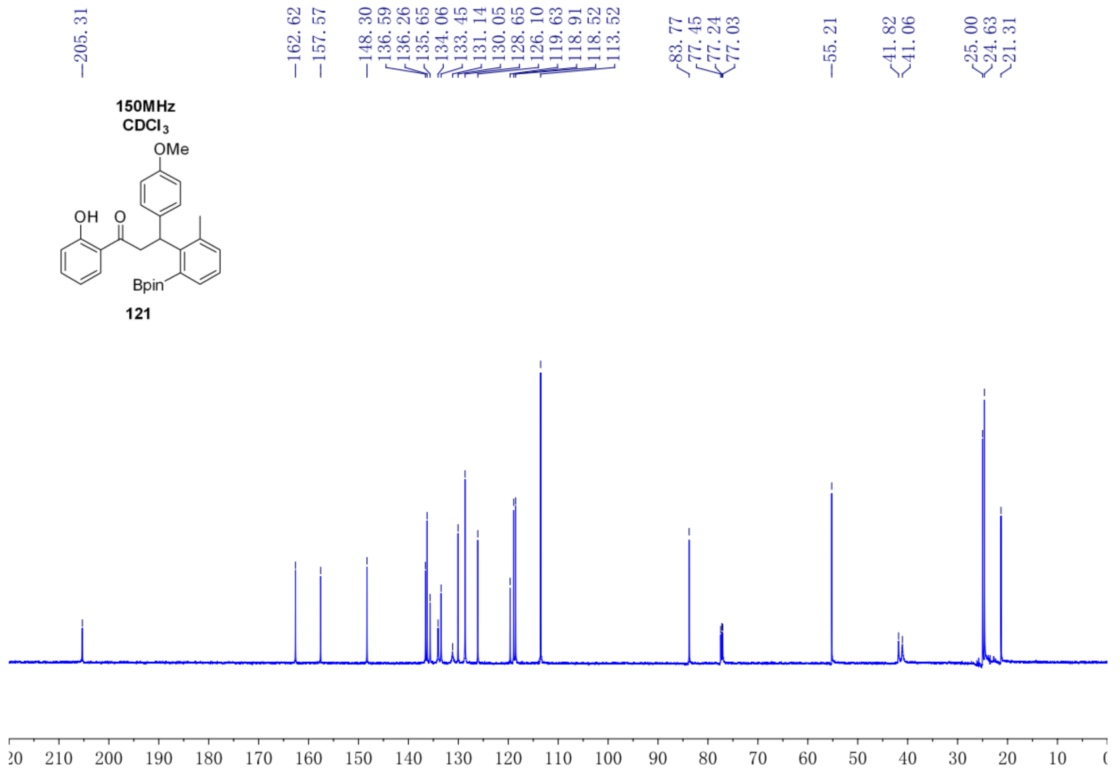

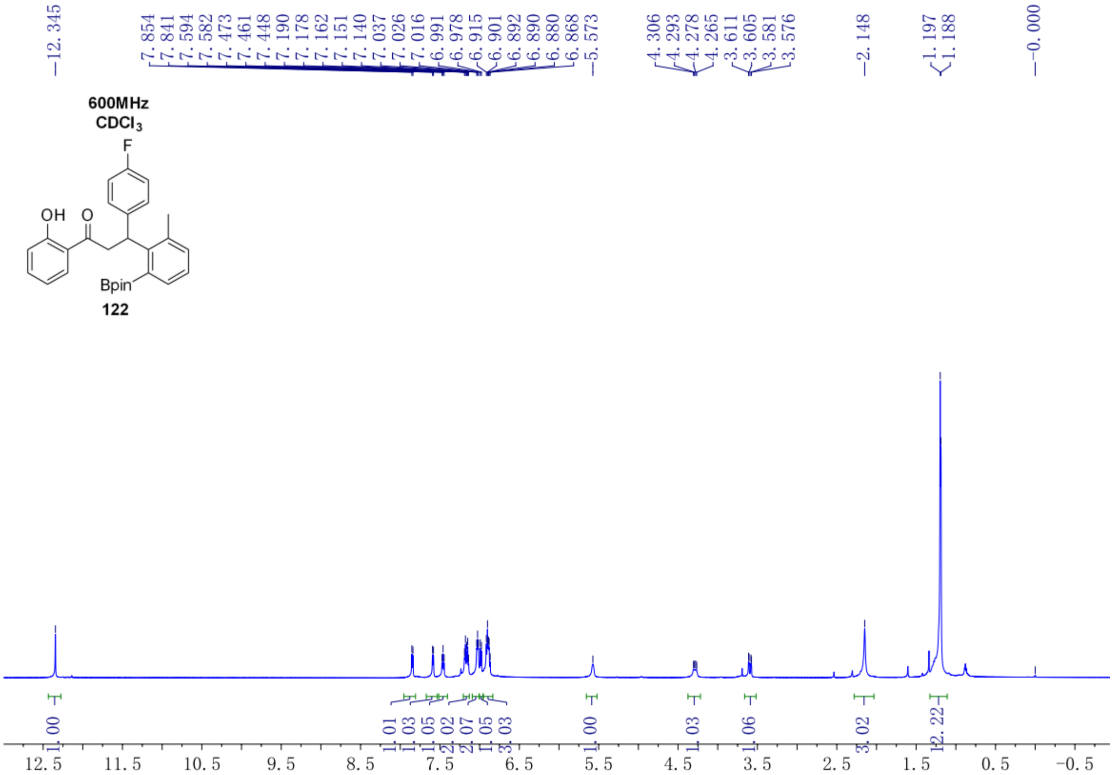
12.345
7.854
7.841
7.594
7.582
7.473
7.461
7.448
7.190
7.178
7.162
7.151
7.140
7.037
7.026
7.016
6.991
6.978
6.915
6.901
6.892
6.890
6.880
6.868
5.573
4.306
4.293
4.278
4.265
3.611
3.605
3.581
3.576
2.148
1.197
1.188
0.000
600MHz
CDCl3
F
OH O
Bpin
122
1.00
1.01
1.03
1.05
2.02
2.07
1.05
3.03
1.00
1.03
1.06
3.02
12.22
12.5 11.5 10.5 9.5 8.5 7.5 6.5 5.5 4.5 3.5 2.5 1.5 0.5 -0.5

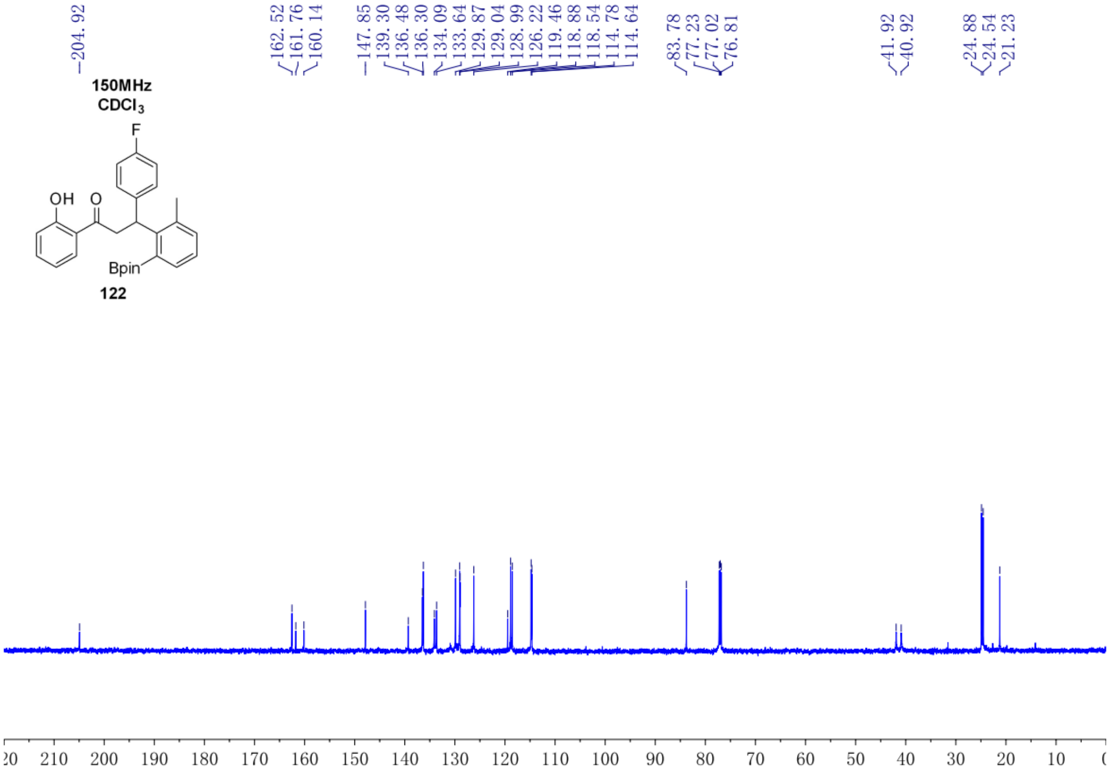
204.92
162.52
161.76
160.14
147.85
139.30
136.48
136.30
134.09
133.64
129.87
129.04
128.99
126.22
119.46
118.88
118.54
114.78
114.64
83.78
77.23
77.02
76.81
41.92
40.92
24.88
24.54
21.23
150MHz
CDCl3
F
OH O
Bpin
122
210 200 190 180 170 160 150 140 130 120 110 100 90 80 70 60 50 40 30 20 10

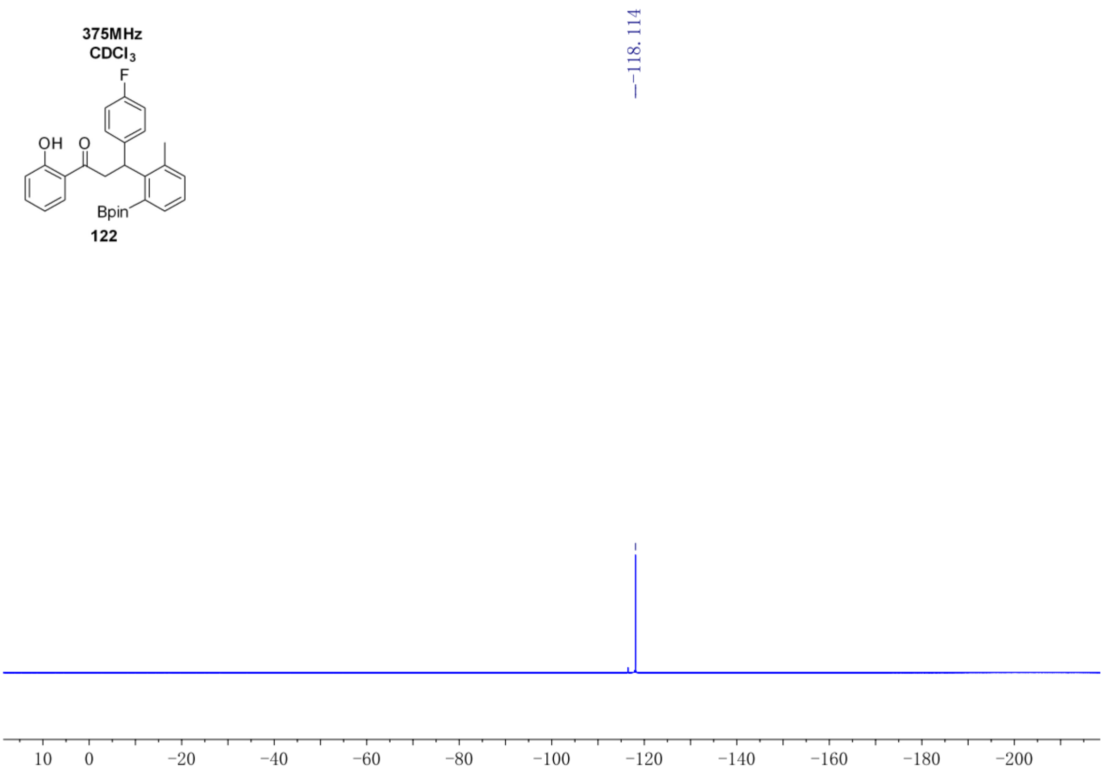
375MHz
$CDCl_3$
F
OH
O
Bpin
122
−118.114
10
0
−20
−40
−60
−80
−100
−120
−140
−160
−180
−200

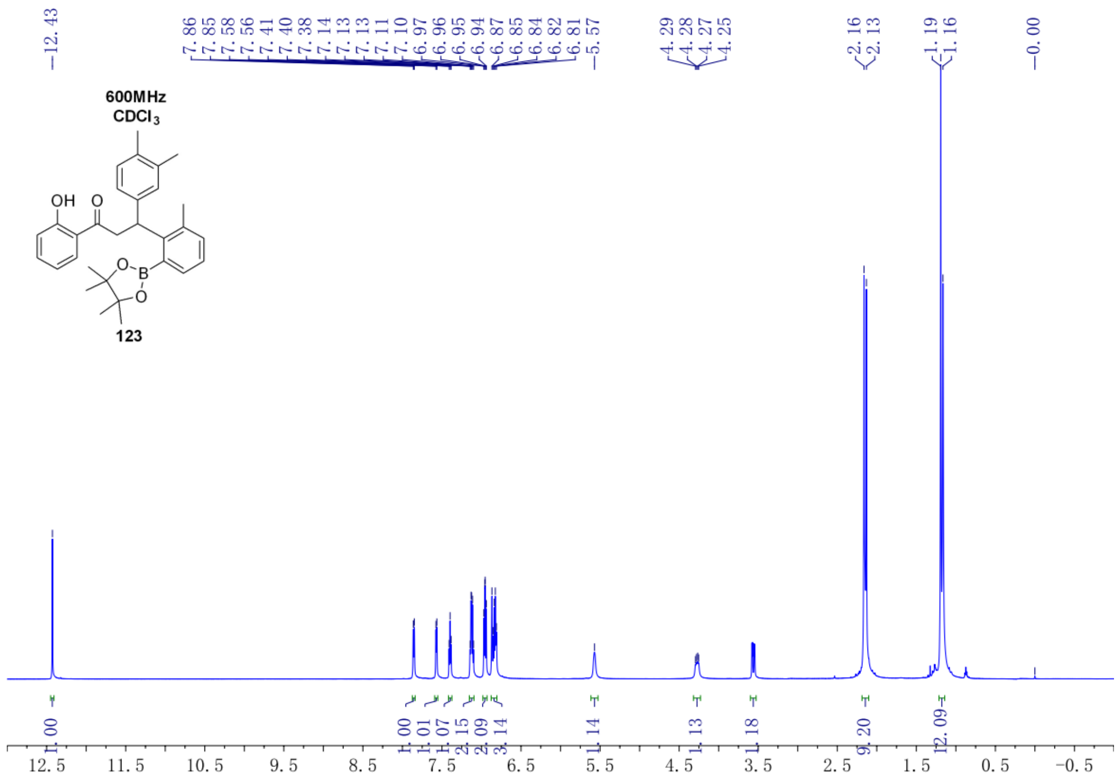
600MHz
$CDCl_3$
OH
O
O
B
O
123
12.43
7.86
7.85
7.58
7.56
7.41
7.40
7.38
7.14
7.13
7.13
7.11
7.10
6.97
6.96
6.95
6.94
6.87
6.85
6.84
6.82
6.81
5.57
4.29
4.28
4.27
4.25
2.16
2.13
1.19
1.16
0.00
1.00
1.01
1.07
2.15
2.09
3.14
1.14
1.13
1.18
9.20
12.09
12.5
11.5
10.5
9.5
8.5
7.5
6.5
5.5
4.5
3.5
2.5
1.5
0.5
−0.5

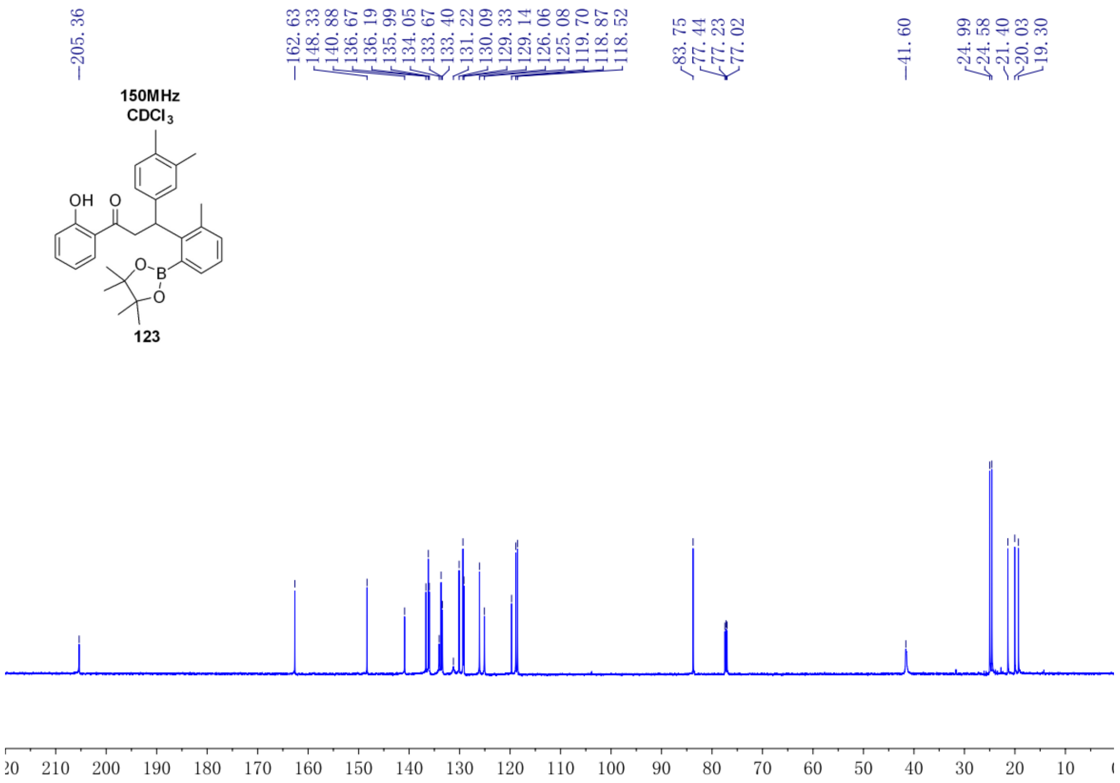
205.36
162.63 148.33 140.88 136.67 136.19 135.99 134.05 133.67 133.40 131.22 130.09 129.33 129.14 126.06 125.08 119.70 118.87 118.52
83.75 77.44 77.23 77.02
41.60
24.99 24.58 21.40 20.03 19.30
150MHz
$CDCl_3$
OH O
O B O
123

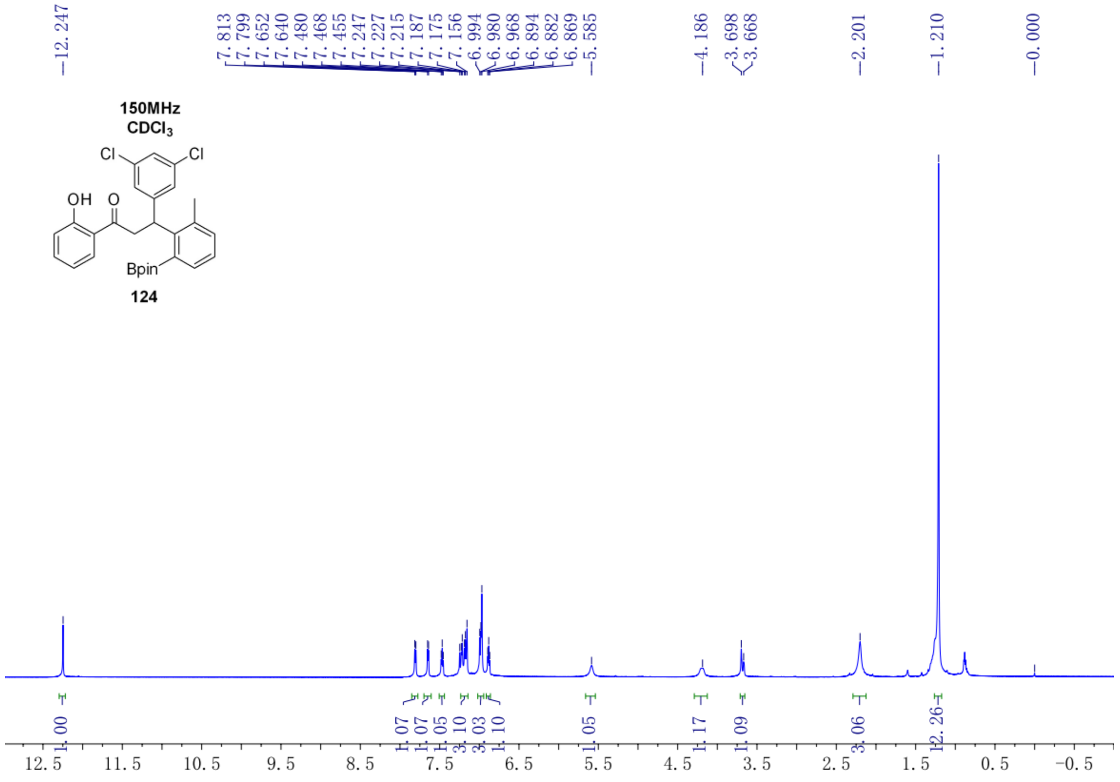
12.247
7.813 7.799 7.652 7.640 7.480 7.468 7.455 7.247 7.227 7.215 7.187 7.175 7.156 6.994 6.980 6.968 6.894 6.882 6.869 5.585
4.186 3.698 3.668
2.201
1.210
0.000
150MHz
$CDCl_3$
Cl Cl
OH O
Bpin
124
1.00 1.07 1.07 1.05 3.10 3.03 1.10 1.05 1.17 1.09 3.06 12.26

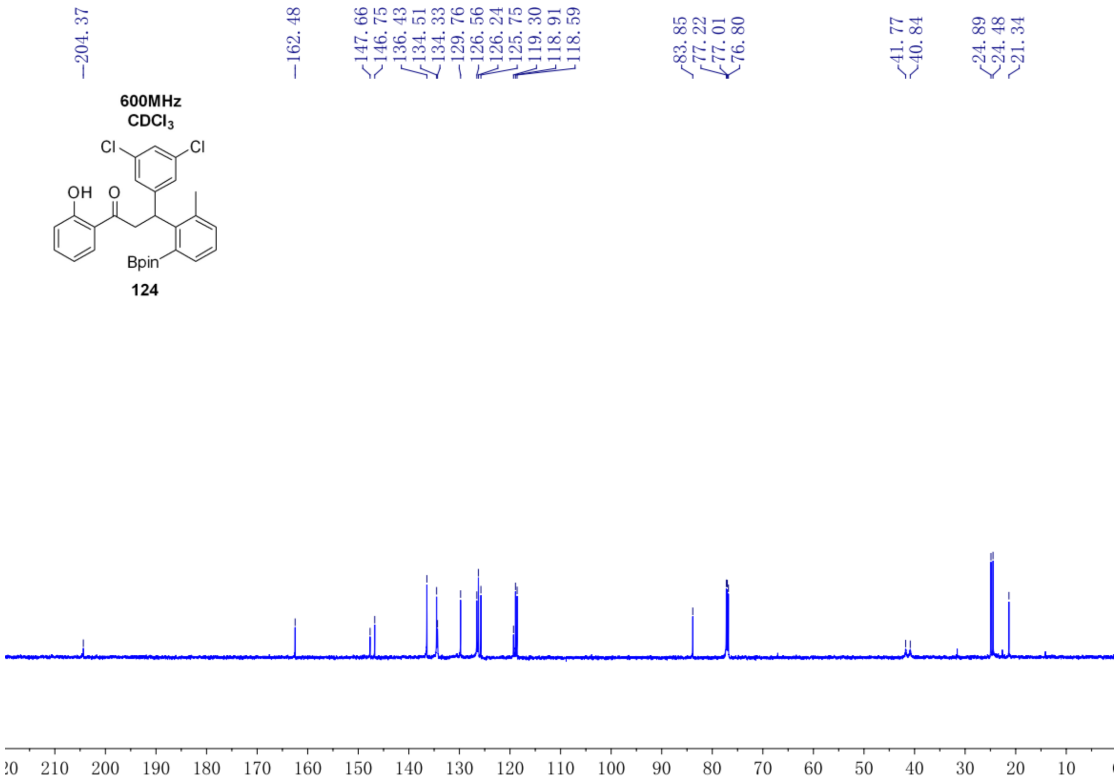
204.37
162.48
147.66
146.75
136.43
134.51
134.33
129.76
126.56
126.24
125.75
119.30
118.91
118.59
83.85
77.22
77.01
76.80
41.77
40.84
24.89
24.48
21.34
600MHz
CDCl3
Cl
Cl
OH
O
Bpin
124
210 200 190 180 170 160 150 140 130 120 110 100 90 80 70 60 50 40 30 20 10

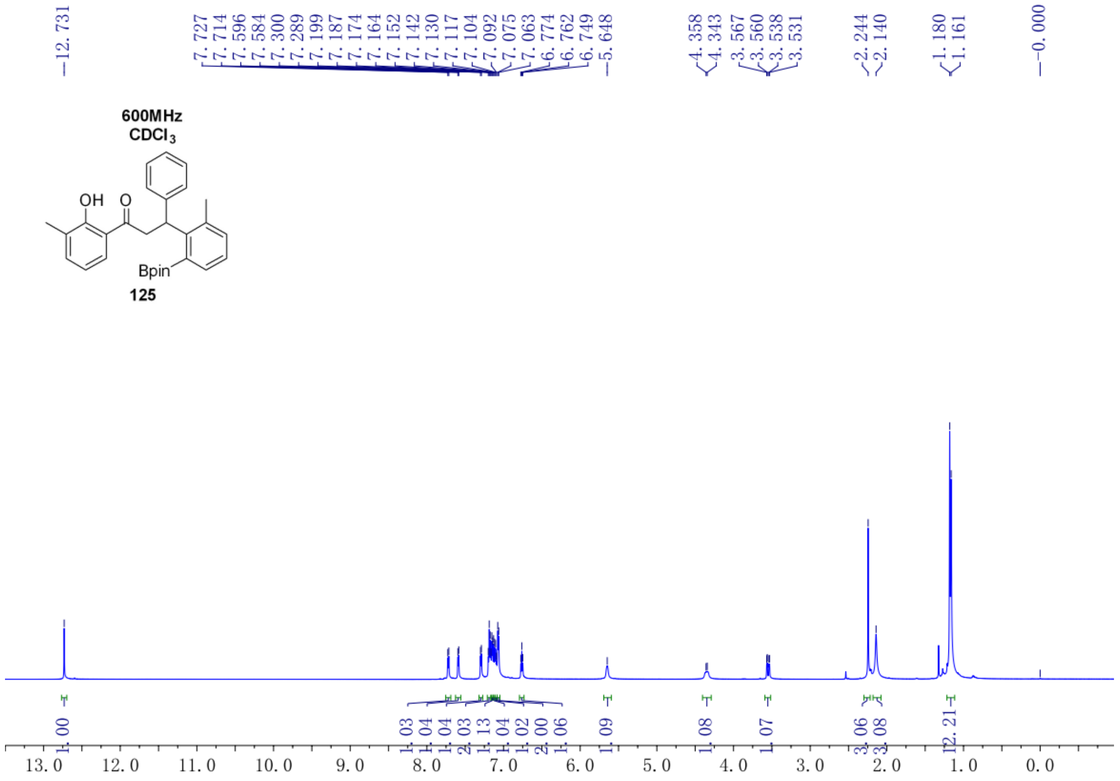
12.731
7.727
7.714
7.596
7.584
7.300
7.289
7.199
7.187
7.174
7.164
7.152
7.142
7.130
7.117
7.104
7.092
7.075
7.063
6.774
6.762
6.749
5.648
4.358
4.343
3.567
3.560
3.538
3.531
2.244
2.140
1.180
1.161
-0.000
600MHz
CDCl3
OH
O
Bpin
125
1.00
1.03
1.04
1.04
2.03
1.13
1.04
1.02
2.00
1.06
1.09
1.08
1.07
3.06
3.08
12.21
13.0 12.0 11.0 10.0 9.0 8.0 7.0 6.0 5.0 4.0 3.0 2.0 1.0 0.0

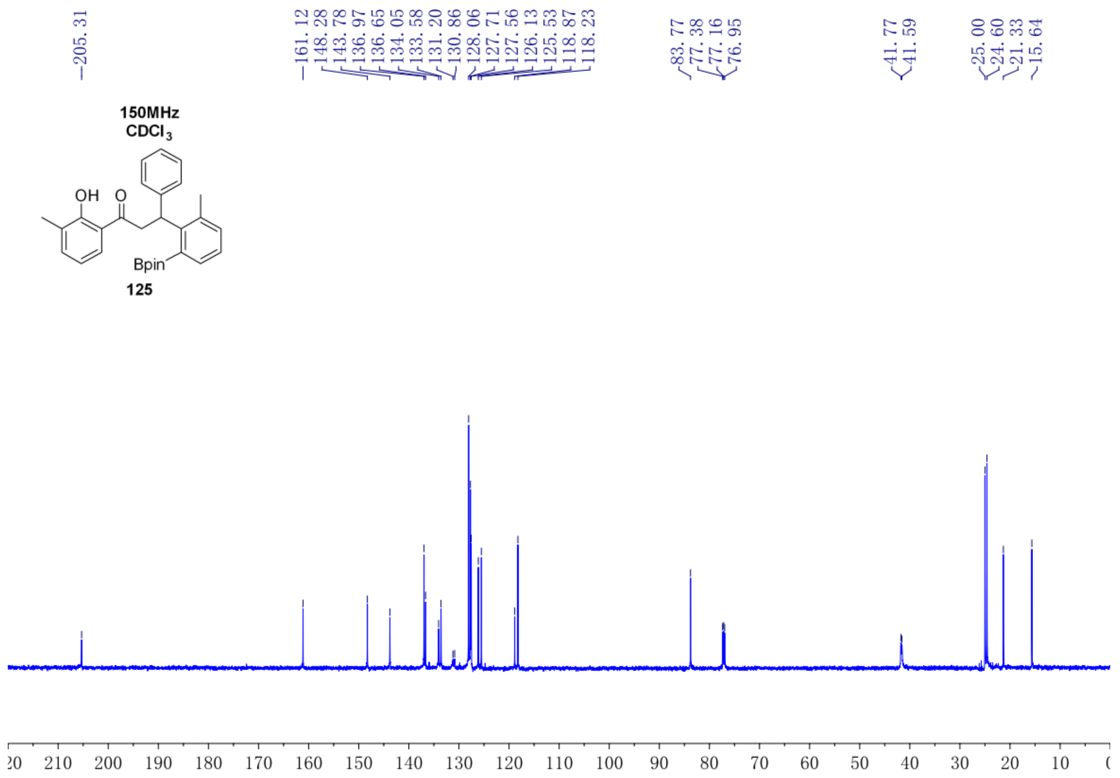
205.31
161.12
148.28
143.78
136.97
136.65
134.05
133.58
131.20
130.86
128.06
127.71
127.56
126.13
125.53
118.87
118.23
83.77
77.38
77.16
76.95
41.77
41.59
25.00
24.60
21.33
15.64
150MHz
CDCl3
OH
O
Bpin
125
210 200 190 180 170 160 150 140 130 120 110 100 90 80 70 60 50 40 30 20 10

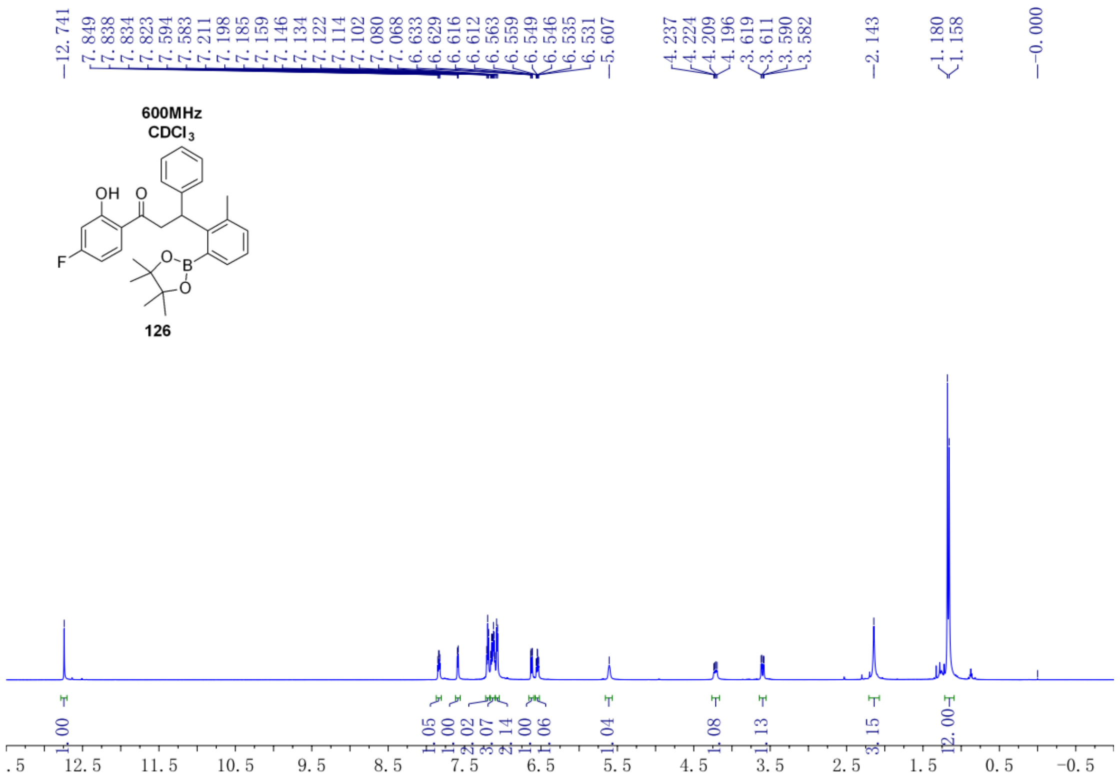
12.741
7.849
7.838
7.834
7.823
7.594
7.583
7.211
7.198
7.185
7.159
7.146
7.134
7.122
7.114
7.102
7.080
7.068
6.633
6.629
6.616
6.612
6.563
6.559
6.549
6.546
6.535
6.531
5.607
4.237
4.224
4.209
4.196
3.619
3.611
3.590
3.582
2.143
1.180
1.158
-0.000
600MHz
CDCl3
OH
O
F
O
B
O
126
1.00
1.05
1.00
2.02
3.07
2.14
1.00
1.06
1.04
1.08
1.13
3.15
12.00
12.5 11.5 10.5 9.5 8.5 7.5 6.5 5.5 4.5 3.5 2.5 1.5 0.5 -0.5

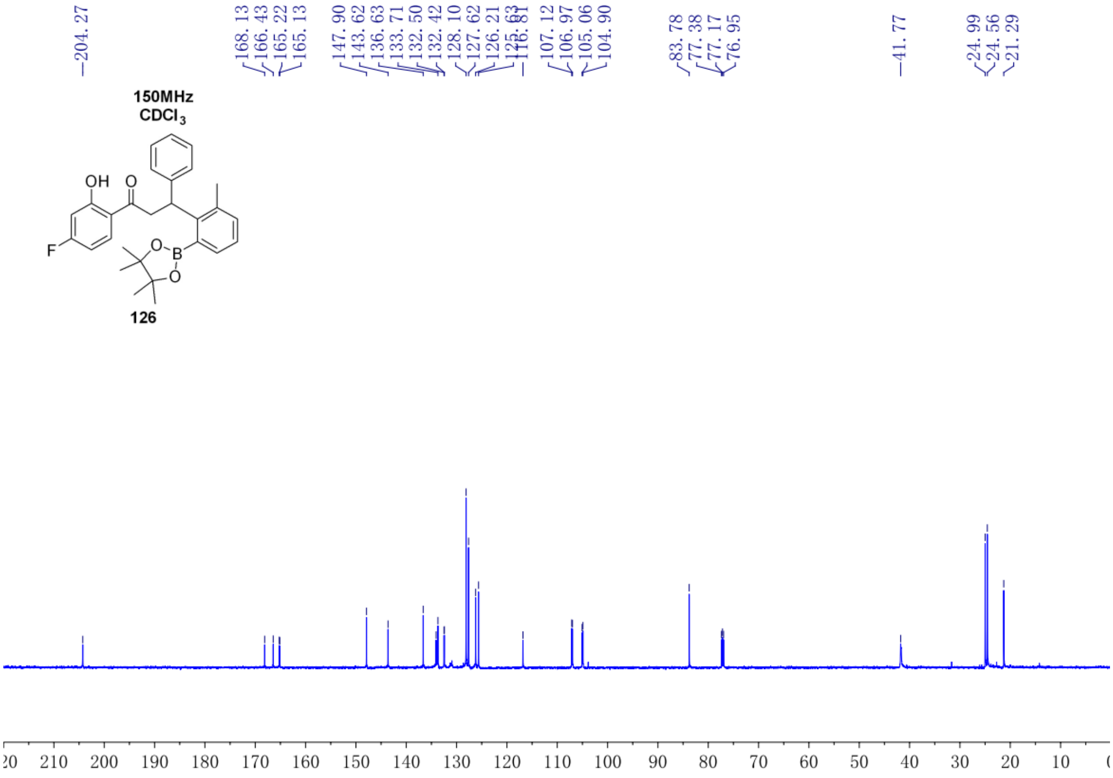
204.27
168.13
166.43
165.22
165.13
147.90
143.62
136.63
133.71
132.50
132.42
128.10
127.62
126.21
116.81
107.12
106.97
105.06
104.90
83.78
77.38
77.17
76.95
41.77
24.99
24.56
21.29
150MHz
CDCl3
OH
O
F
O
B
O
126
210 200 190 180 170 160 150 140 130 120 110 100 90 80 70 60 50 40 30 20 10

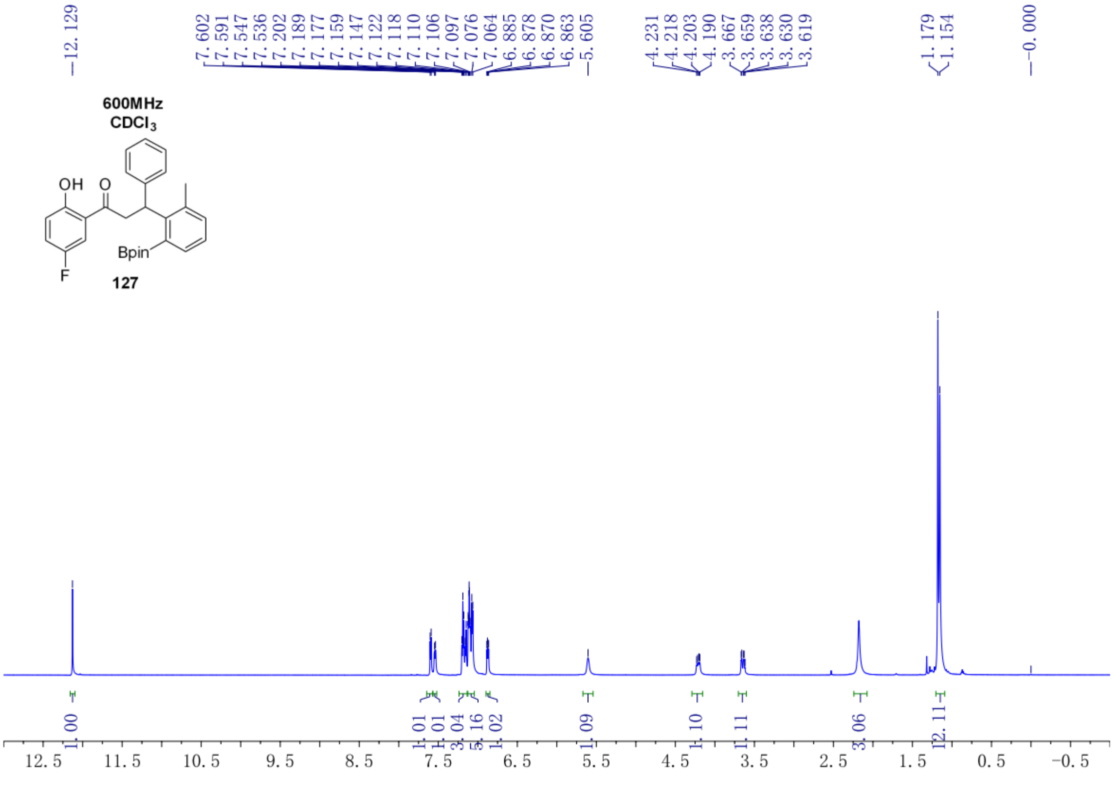
12.129
7.602
7.591
7.547
7.536
7.202
7.189
7.177
7.159
7.147
7.122
7.118
7.110
7.106
7.097
7.076
7.064
6.885
6.878
6.870
6.863
5.605
4.231
4.218
4.203
4.190
3.667
3.659
3.638
3.630
3.619
1.179
1.154
0.000
600MHz
CDCl3
OH
O
Bpin
F
127
1.00
1.01
1.01
3.04
5.16
1.02
1.09
1.10
1.11
3.06
12.11
12.5 11.5 10.5 9.5 8.5 7.5 6.5 5.5 4.5 3.5 2.5 1.5 0.5 -0.5

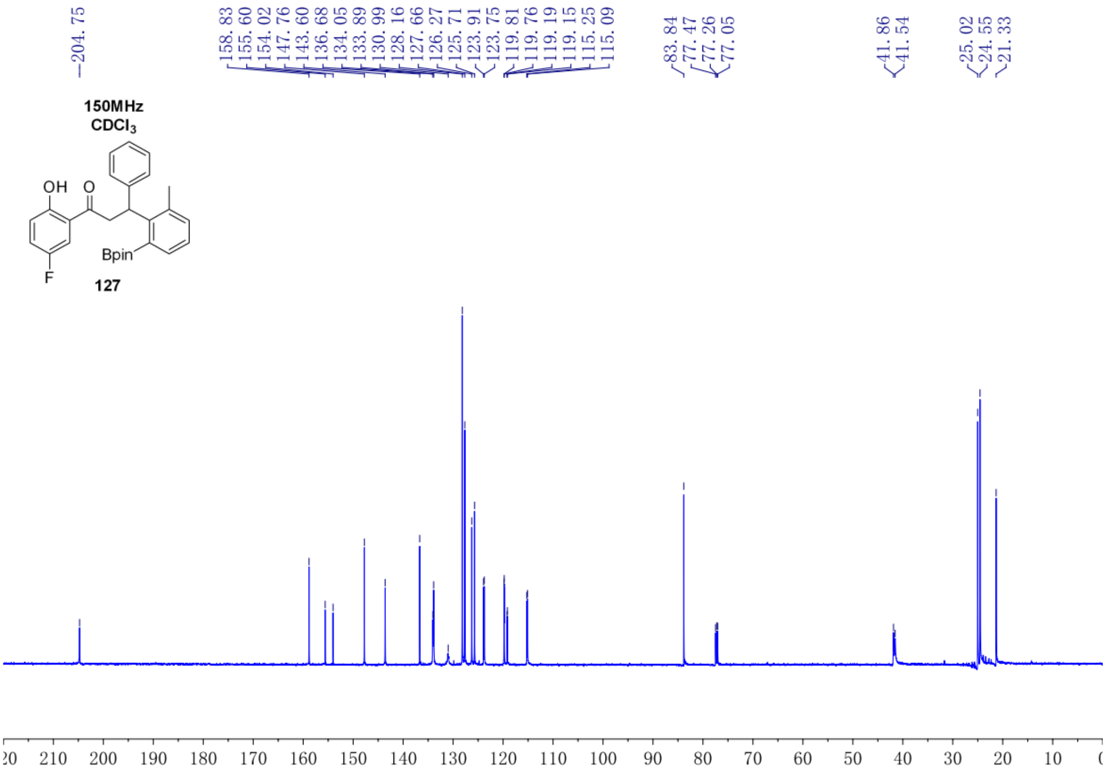
204.75
158.83
155.60
154.02
147.76
143.60
136.68
134.05
133.89
130.99
128.16
127.66
126.27
125.71
123.91
123.75
119.81
119.76
119.19
119.15
115.25
115.09
83.84
77.47
77.26
77.05
41.86
41.54
25.02
24.55
21.33
150MHz
$CDCl_3$
OH
O
F
Bpin
127
210
200
190
180
170
160
150
140
130
120
110
100
90
80
70
60
50
40
30
20
10

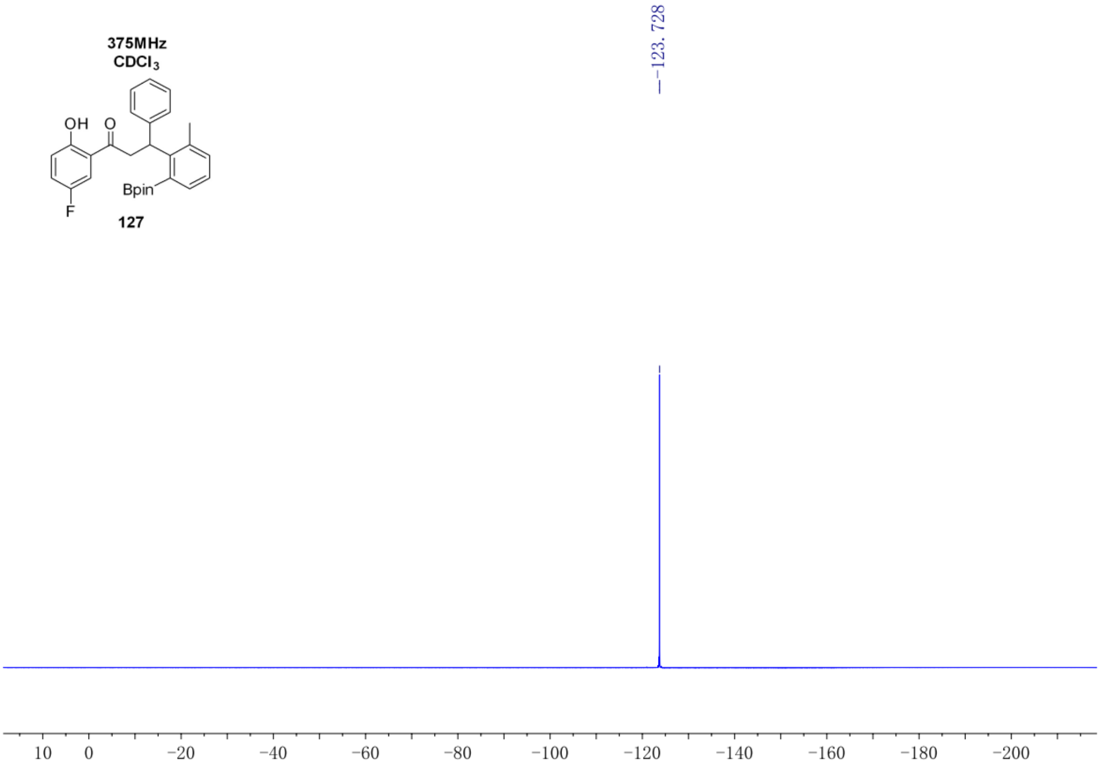
−123.728
375MHz
$CDCl_3$
OH
O
F
Bpin
127
10
0
−20
−40
−60
−80
−100
−120
−140
−160
−180
−200

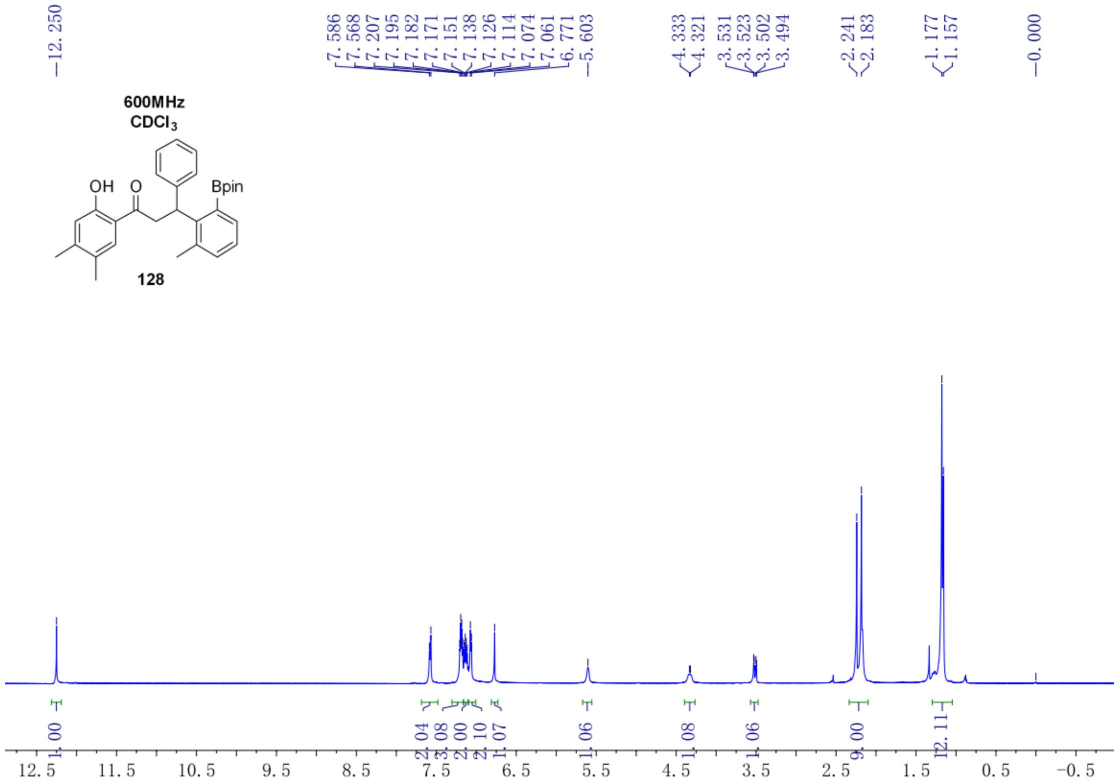
12.250
7.586
7.568
7.207
7.195
7.182
7.171
7.151
7.138
7.126
7.114
7.074
7.061
6.771
5.603
4.333
4.321
3.531
3.523
3.502
3.494
2.241
2.183
1.177
1.157
0.000
600MHz
CDCl3
OH
O
Bpin
128
1.00
2.04
3.08
2.00
2.10
1.07
1.06
1.08
1.06
9.00
12.11
12.5
11.5
10.5
9.5
8.5
7.5
6.5
5.5
4.5
3.5
2.5
1.5
0.5
-0.5

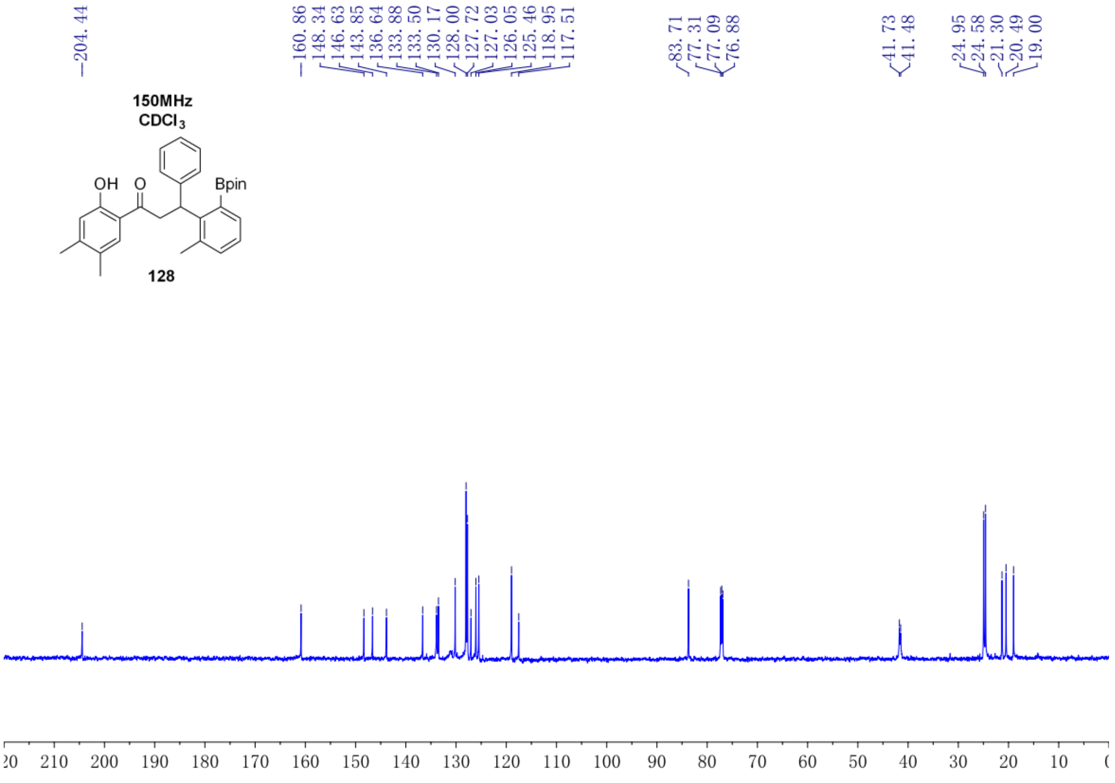
204.44
160.86
148.34
146.63
143.85
136.64
133.88
133.50
130.17
128.00
127.72
127.03
126.05
125.46
118.95
117.51
83.71
77.31
77.09
76.88
41.73
41.48
24.95
24.58
21.30
20.49
19.00
150MHz
CDCl3
OH
O
Bpin
128
210
200
190
180
170
160
150
140
130
120
110
100
90
80
70
60
50
40
30
20
10

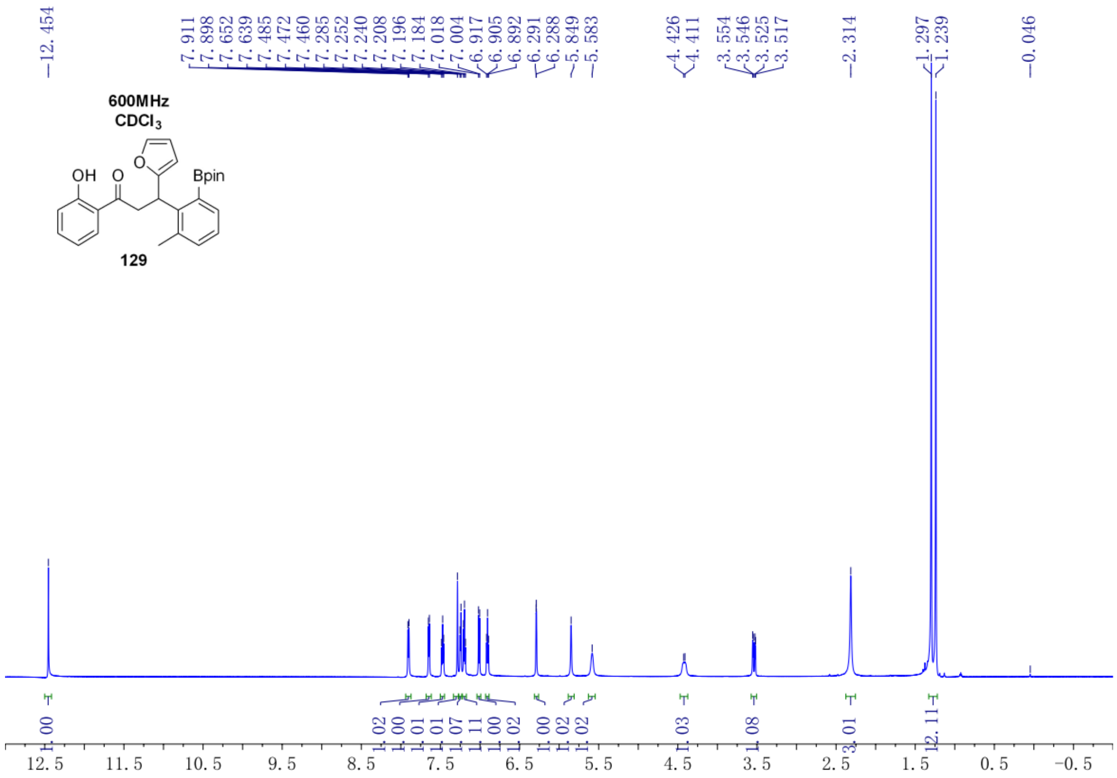
600MHz
CDCl3
OH O
O
Bpin
129
12.454
7.911 7.898 7.652 7.639 7.485 7.472 7.460 7.285 7.252 7.240 7.208 7.196 7.184 7.018 7.004 6.917 6.905 6.892 6.291 6.288 5.849 5.583
4.426 4.411
3.554 3.546 3.525 3.517
2.314
1.297 1.239
0.046
1.00 2.02 1.00 1.01 1.01 1.07 1.11 1.00 2.02 1.00 1.02 1.02 1.03 1.08 3.01 12.11
12.5 11.5 10.5 9.5 8.5 7.5 6.5 5.5 4.5 3.5 2.5 1.5 0.5 -0.5

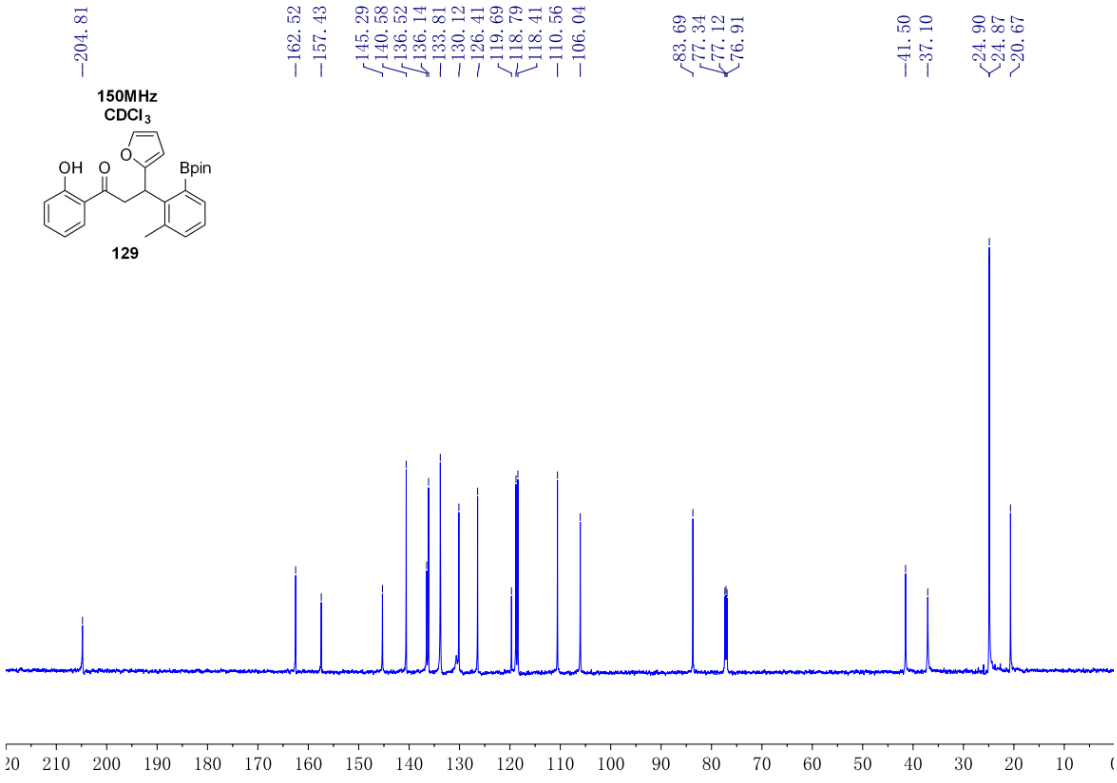
150MHz
CDCl3
OH O
O
Bpin
129
204.81
162.52 157.43
145.29 140.58 136.52 136.14 133.81 130.12 126.41 119.69 118.79 118.41 110.56 106.04
83.69 77.34 77.12 76.91
41.50 37.10
24.90 24.87 20.67
210 200 190 180 170 160 150 140 130 120 110 100 90 80 70 60 50 40 30 20 10

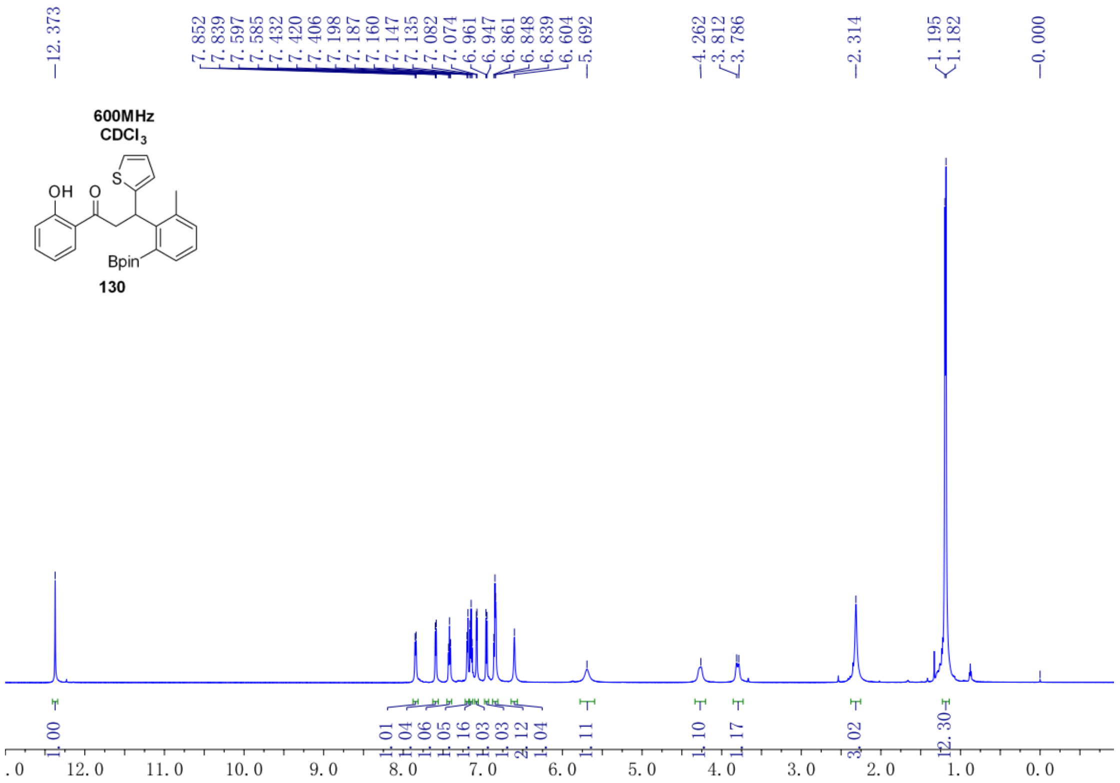
12.373
7.852
7.839
7.597
7.585
7.432
7.420
7.406
7.198
7.187
7.160
7.147
7.135
7.082
7.074
6.961
6.947
6.861
6.848
6.839
6.604
5.692
4.262
3.812
3.786
2.314
1.195
1.182
0.000
600MHz
$CDCl_3$
OH
O
S
Bpin
130
1.00
1.01
1.04
1.06
1.05
1.16
1.03
1.03
2.12
1.04
1.11
1.10
1.17
3.02
12.30
12.0
11.0
10.0
9.0
8.0
7.0
6.0
5.0
4.0
3.0
2.0
1.0
0.0

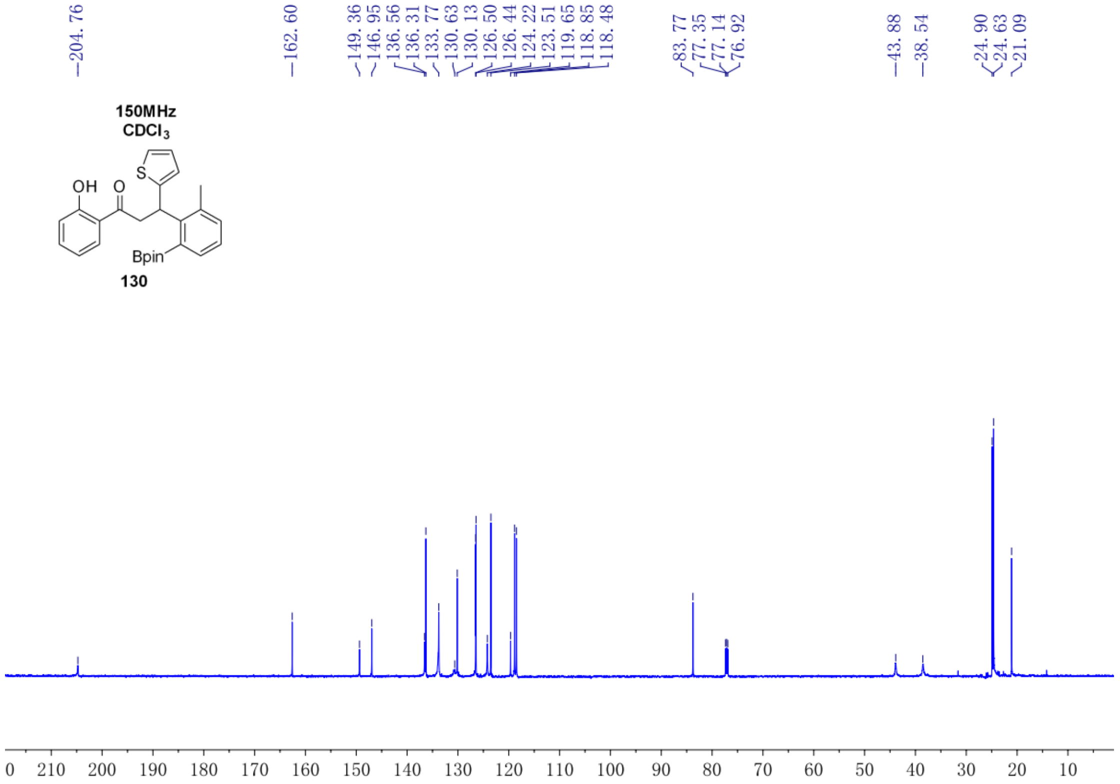
204.76
162.60
149.36
146.95
136.56
136.31
133.77
130.63
130.13
126.50
126.44
124.22
123.51
119.65
118.85
118.48
83.77
77.35
77.14
76.92
43.88
38.54
24.90
24.63
21.09
150MHz
$CDCl_3$
OH
O
S
Bpin
130
210
200
190
180
170
160
150
140
130
120
110
100
90
80
70
60
50
40
30
20
10

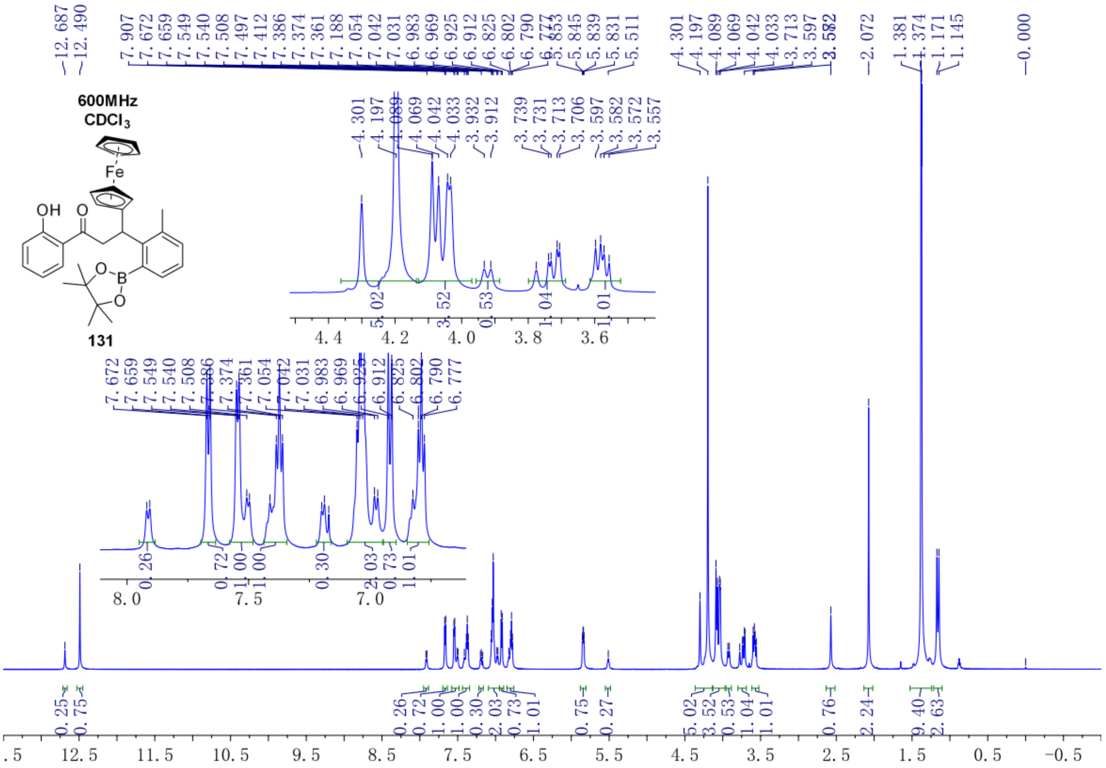
600MHz
$CDCl_3$
OH
O
Fe
131

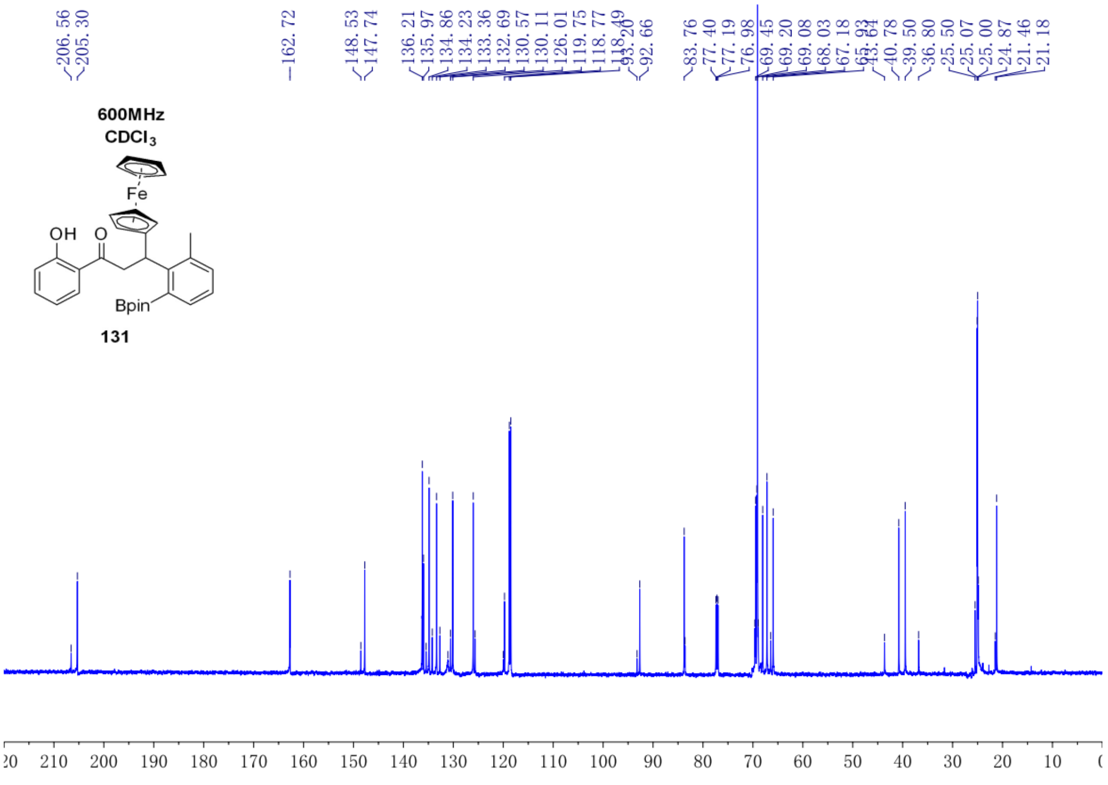
600MHz
$CDCl_3$
OH
O
Fe
Bpin
131

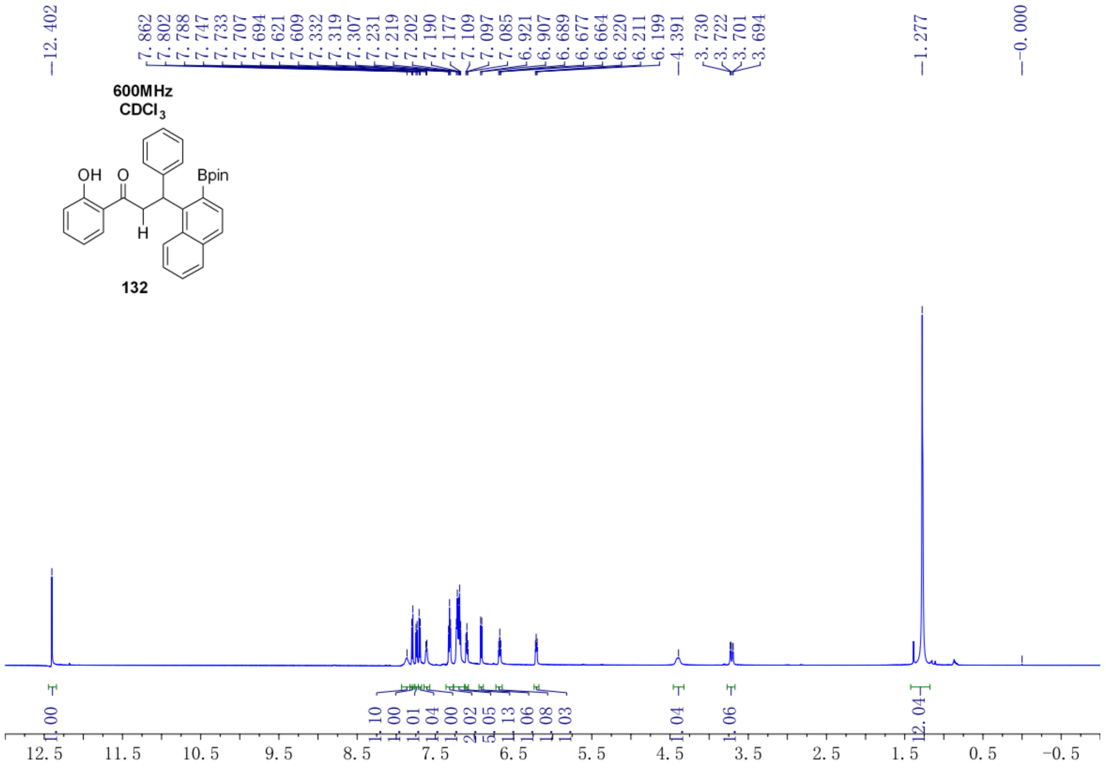
12.402
7.862 7.802 7.788 7.747 7.733 7.707 7.694 7.621 7.609 7.332 7.319 7.307 7.231 7.219 7.202 7.190 7.177 7.109 7.097 7.085 6.921 6.907 6.689 6.677 6.664 6.220 6.211 6.199
4.391
3.730 3.722 3.701 3.694
1.277
0.000
600MHz
CDCl3
OH
O
Bpin
H
132
1.00
1.10 1.00 1.01 1.04 1.00 2.02 5.05 1.13 1.06 1.08 1.03
1.04
1.06
12.04
12.5 11.5 10.5 9.5 8.5 7.5 6.5 5.5 4.5 3.5 2.5 1.5 0.5 -0.5

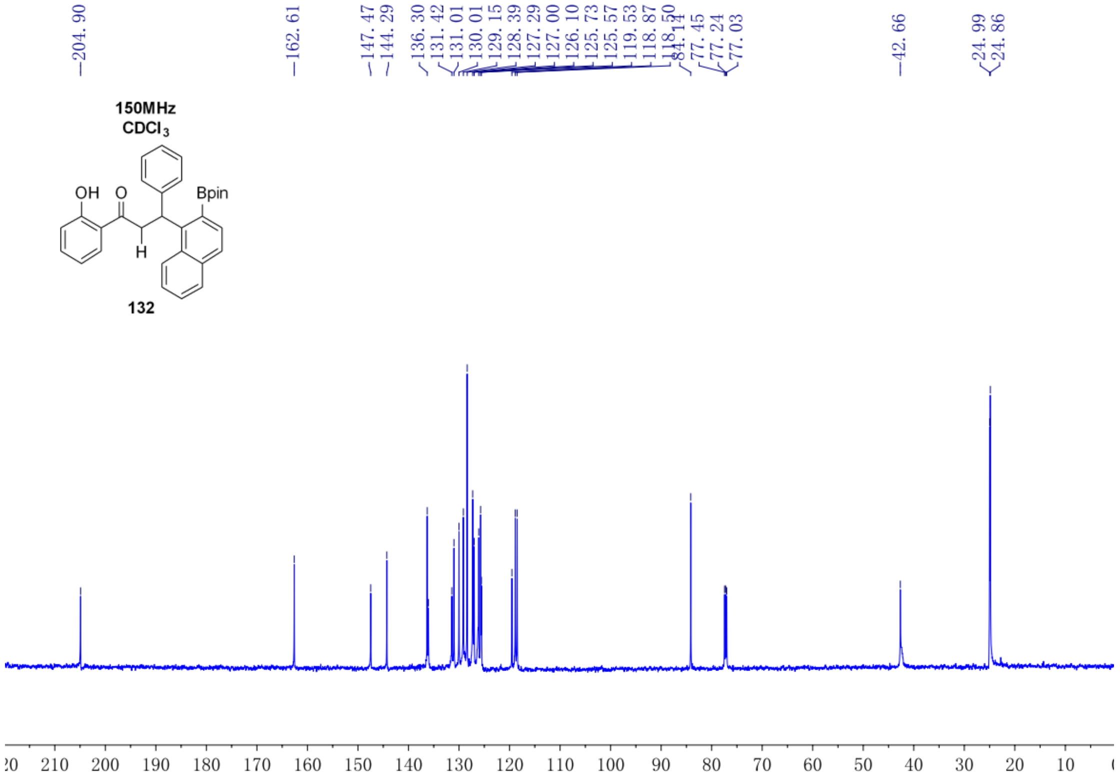
204.90
162.61
147.47 144.29
136.30 131.42 131.01 130.01 129.15 128.39 127.29 127.00 126.10 125.73 125.57 119.53 118.87 118.50
84.14
77.45 77.24 77.03
42.66
24.99 24.86
150MHz
CDCl3
OH
O
Bpin
H
132
210 200 190 180 170 160 150 140 130 120 110 100 90 80 70 60 50 40 30 20 10

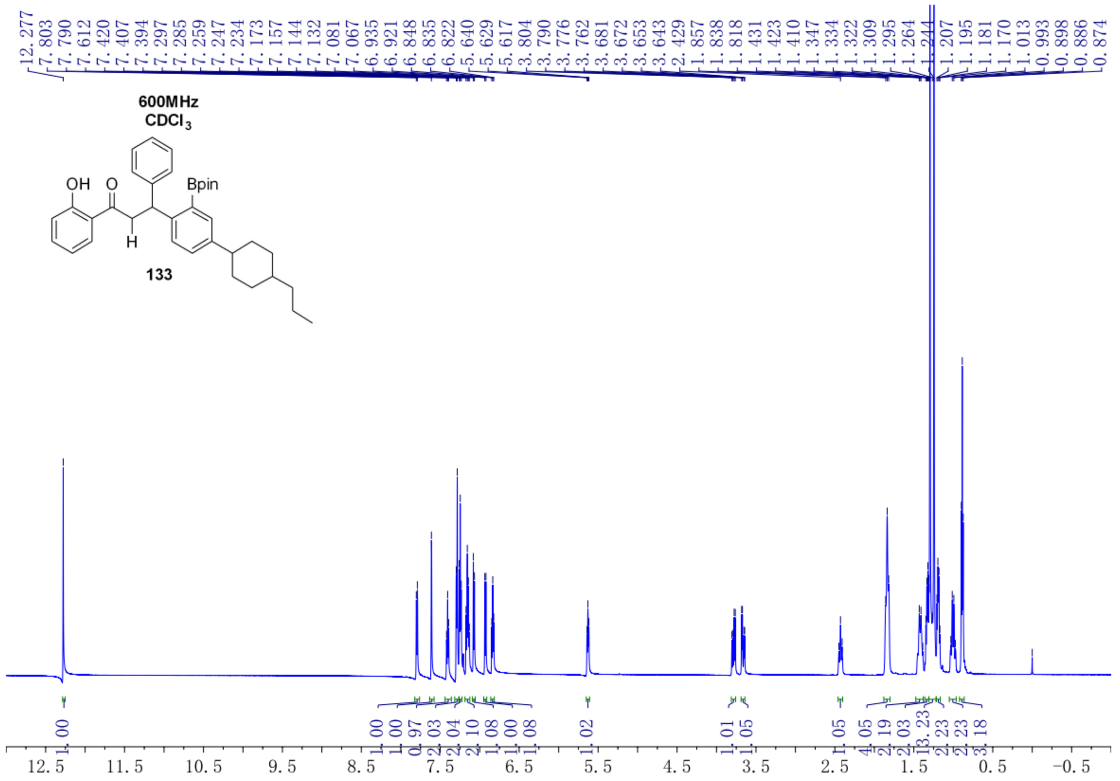
12.277
7.803
7.790
7.612
7.420
7.407
7.394
7.297
7.285
7.259
7.247
7.234
7.173
7.157
7.144
7.132
7.081
7.067
6.935
6.921
6.848
6.835
6.822
5.640
5.629
5.617
3.804
3.790
3.776
3.762
3.681
3.672
3.653
3.643
2.429
1.857
1.838
1.818
1.431
1.423
1.410
1.347
1.334
1.322
1.309
1.295
1.264
1.244
1.207
1.195
1.181
1.170
1.013
0.993
0.898
0.886
0.874
600MHz
CDCl3
OH
O
Bpin
H
133

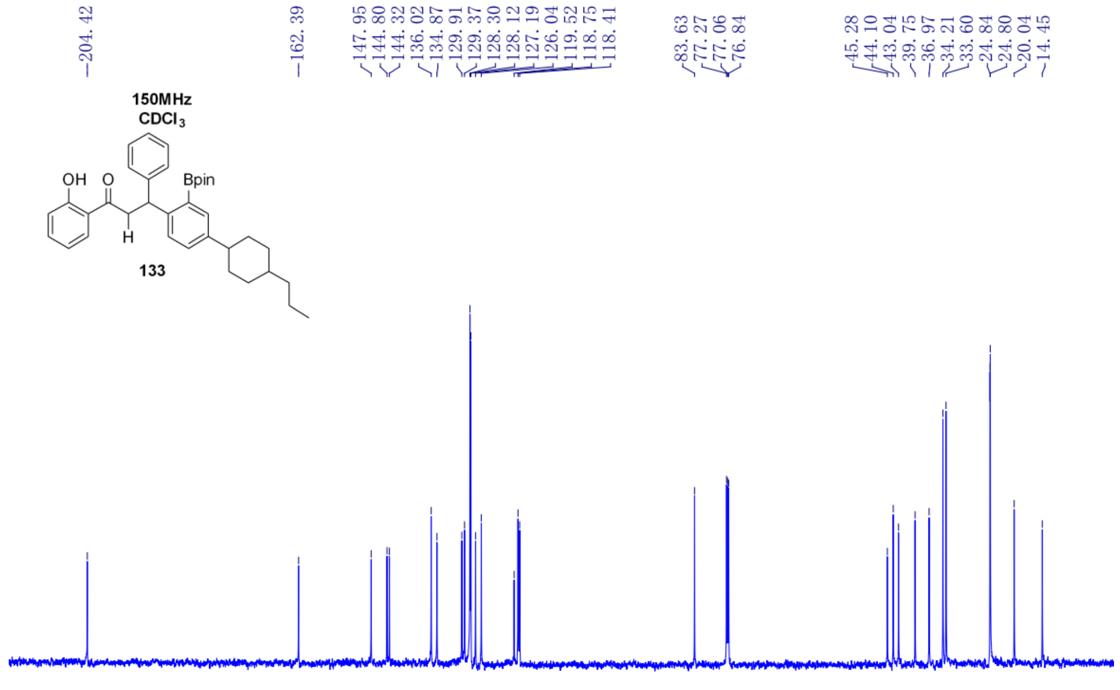
204.42
162.39
147.95
144.80
144.32
136.02
134.87
129.91
129.37
128.30
128.12
127.19
126.04
119.52
118.75
118.41
83.63
77.27
77.06
76.84
45.28
44.10
43.04
39.75
36.97
34.21
33.60
24.84
24.80
20.04
14.45
150MHz
CDCl3
OH
O
Bpin
H
133

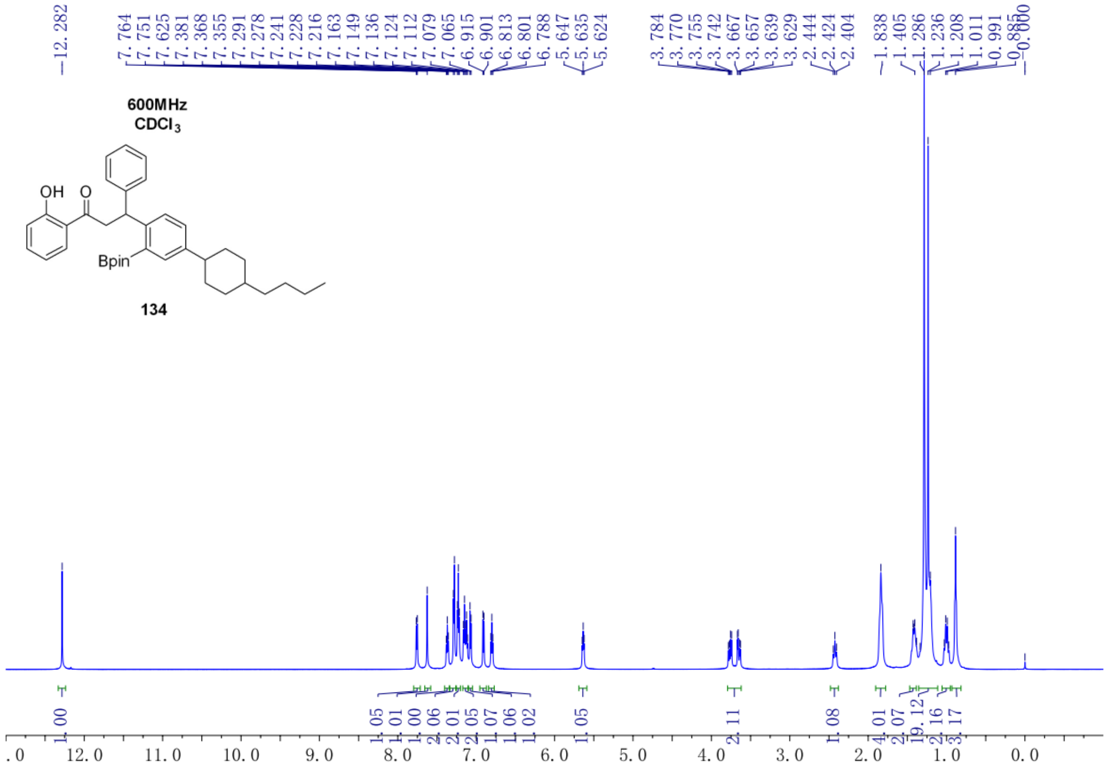
600MHz
CDCl3
OH
O
Bpin
134

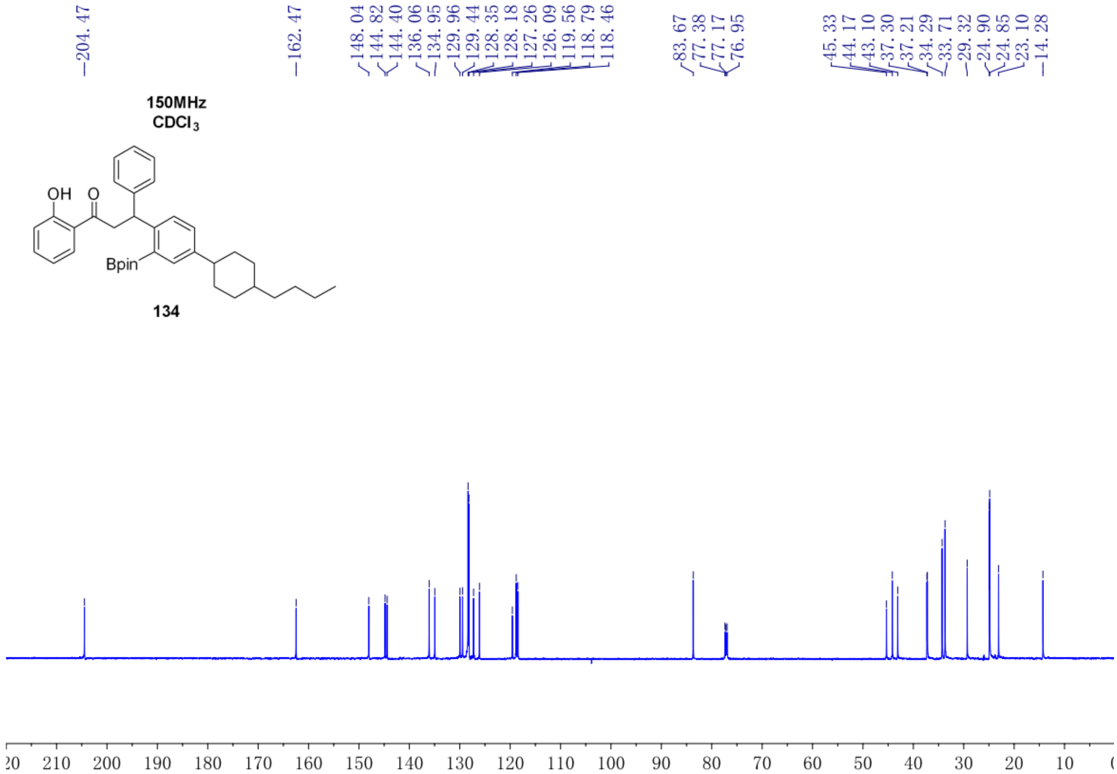
150MHz
CDCl3
OH
O
Bpin
134

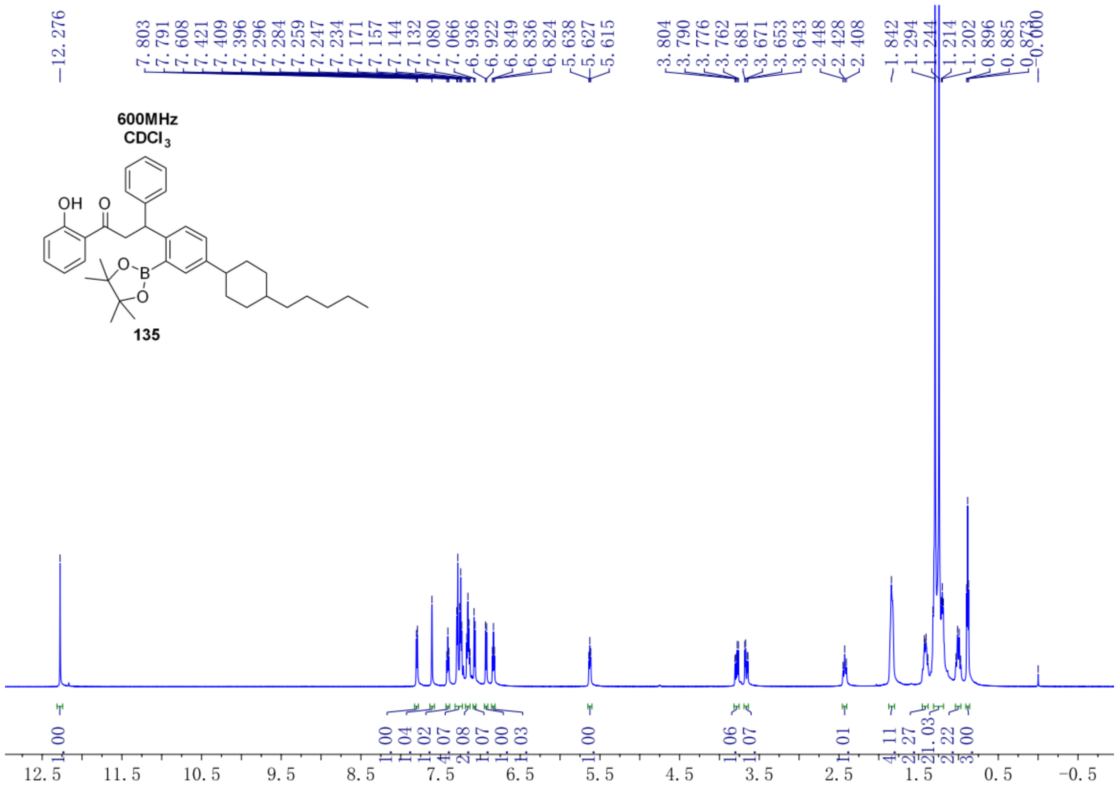
12.276
7.803 7.791 7.608 7.421 7.409 7.396 7.296 7.284 7.259 7.247 7.234 7.171 7.157 7.144 7.132 7.080 7.066 6.936 6.922 6.849 6.836 6.824 5.638 5.627 5.615
3.804 3.790 3.776 3.762 3.681 3.671 3.653 3.643 2.448 2.428 2.408
1.842 1.294 1.244 1.214 1.202 0.896 0.885 0.873 0.000
600MHz
CDCl3
OH
O
B
O
O
135

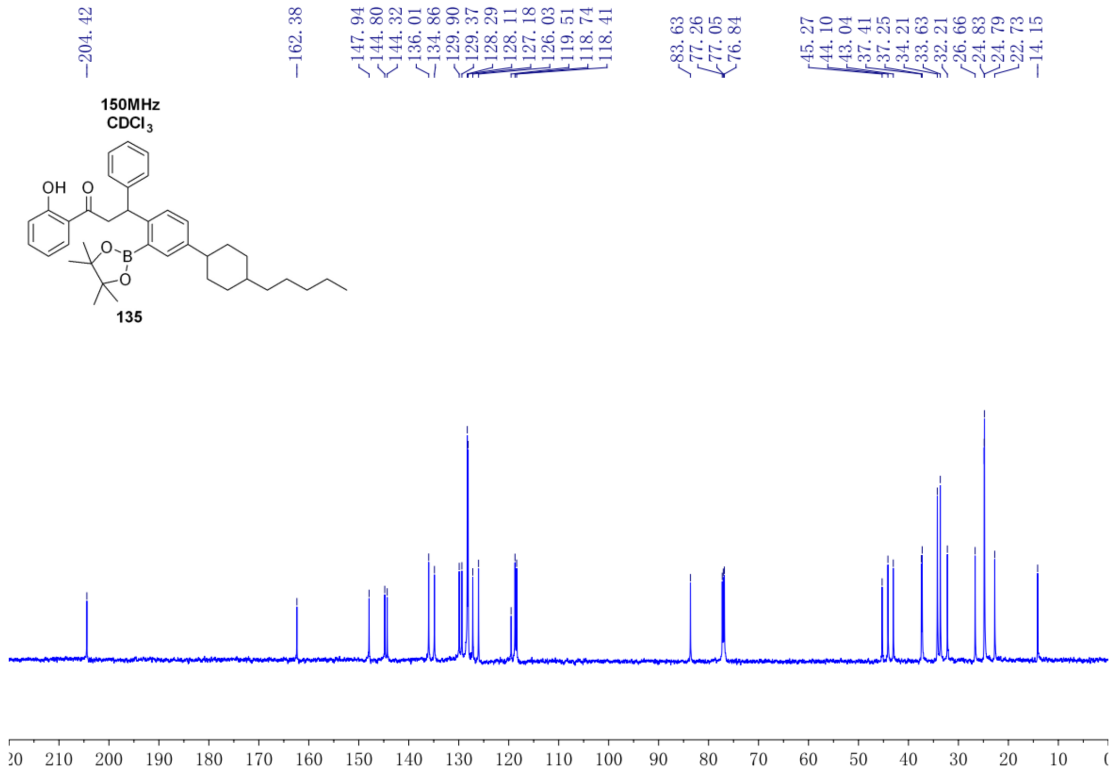
204.42
162.38
147.94 144.80 144.32 136.01 134.86 129.90 129.37 128.29 128.11 127.18 126.03 119.51 118.74 118.41
83.63 77.26 77.05 76.84
45.27 44.10 43.04 37.41 37.25 34.21 33.63 32.21 26.66 24.83 24.79 22.73 14.15
150MHz
CDCl3
OH
O
B
O
O
135

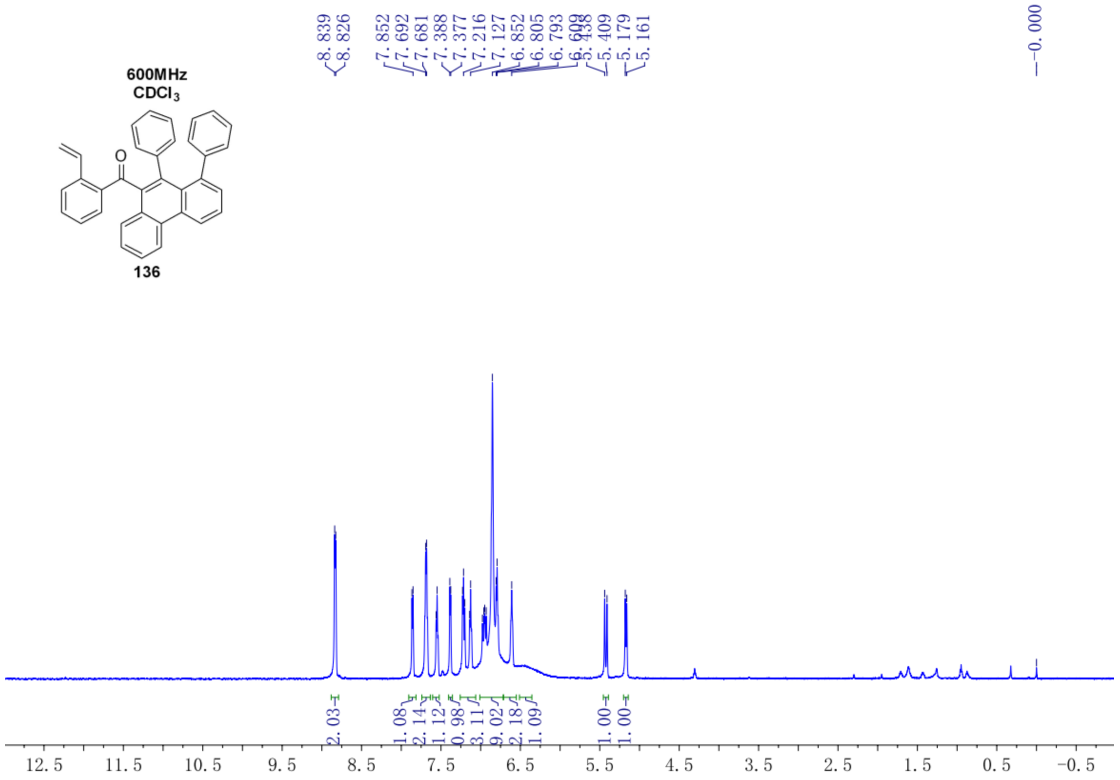
600MHz
$CDCl_3$
136
8.839
8.826
7.852
7.692
7.681
7.388
7.377
7.216
7.127
6.852
6.805
6.793
5.409
5.179
5.161
0.000
2.03
1.08
2.14
1.12
0.98
3.11
9.02
2.18
1.09
1.00
1.00

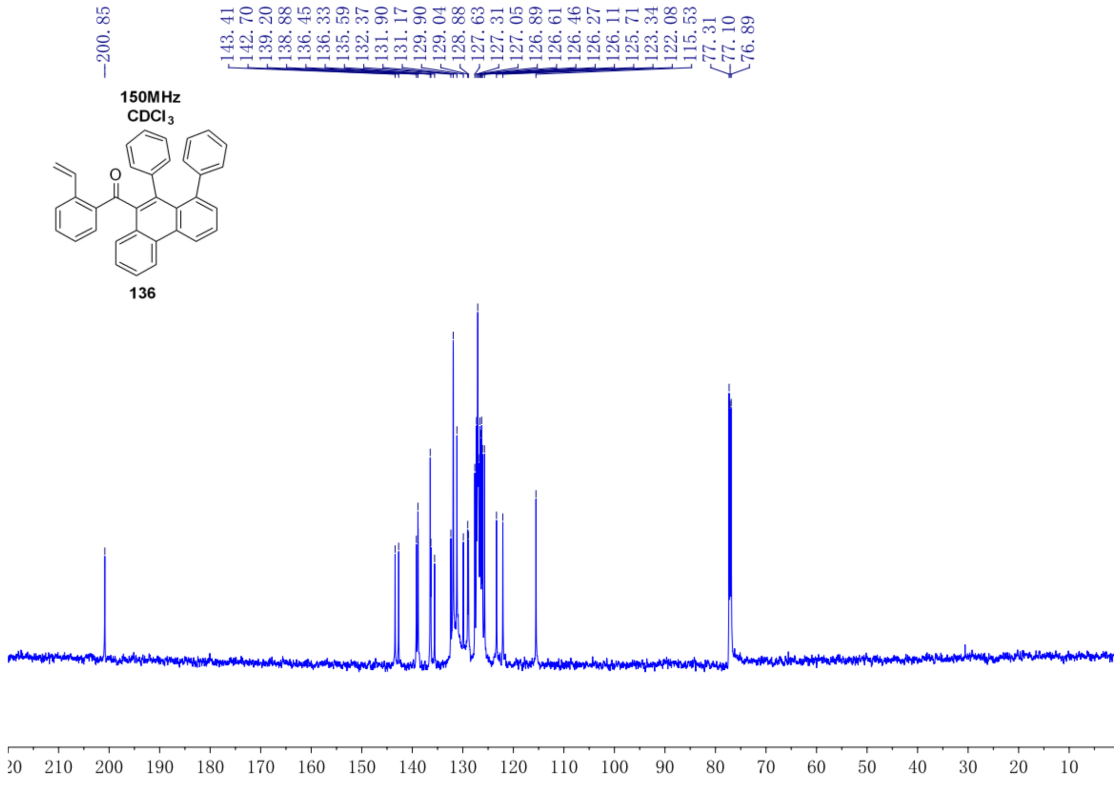
150MHz
$CDCl_3$
136
200.85
143.41
142.70
139.20
138.88
136.45
136.33
135.59
132.37
131.90
131.17
129.90
129.04
128.88
127.63
127.31
127.05
126.89
126.61
126.46
126.27
126.11
125.71
123.34
122.08
115.53
77.31
77.10
76.89

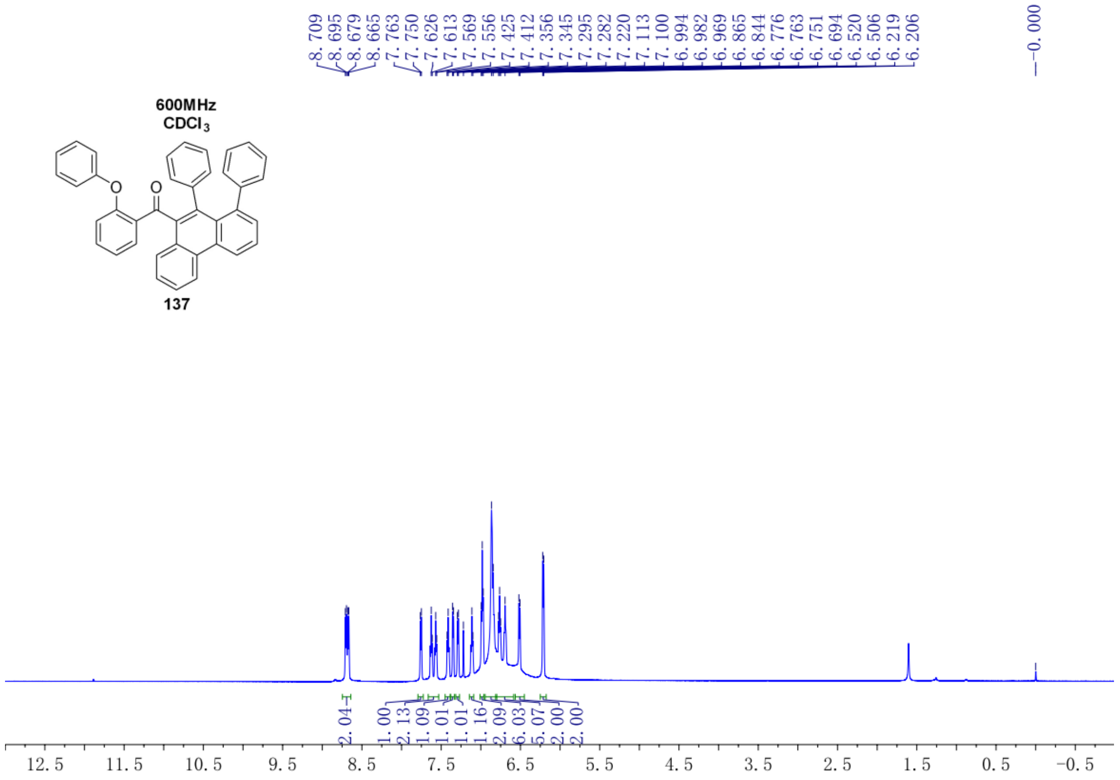

8.709
8.695
8.679
8.665
7.763
7.750
7.626
7.613
7.569
7.556
7.425
7.412
7.356
7.345
7.295
7.282
7.220
7.113
7.100
6.994
6.982
6.969
6.865
6.844
6.776
6.763
6.751
6.694
6.520
6.506
6.219
6.206
0.000
600MHz
CDCl3
O
O
137
2.04
1.00
2.13
1.09
1.01
1.01
1.16
2.09
6.03
5.07
2.00
2.00
12.5 11.5 10.5 9.5 8.5 7.5 6.5 5.5 4.5 3.5 2.5 1.5 0.5 -0.5

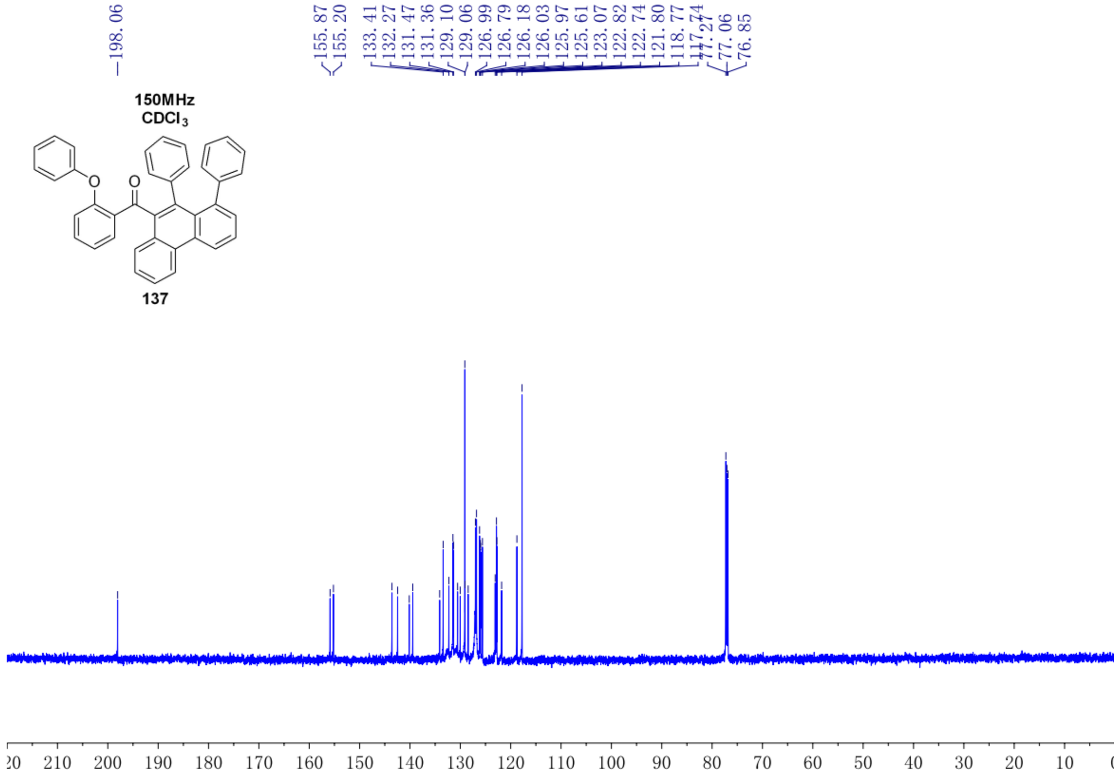

198.06
155.87
155.20
133.41
132.27
131.47
131.36
129.10
129.06
126.99
126.79
126.18
126.03
125.97
125.61
123.07
122.82
122.74
121.80
118.77
117.74
77.27
77.06
76.85
150MHz
CDCl3
O
O
137
210 200 190 180 170 160 150 140 130 120 110 100 90 80 70 60 50 40 30 20 10

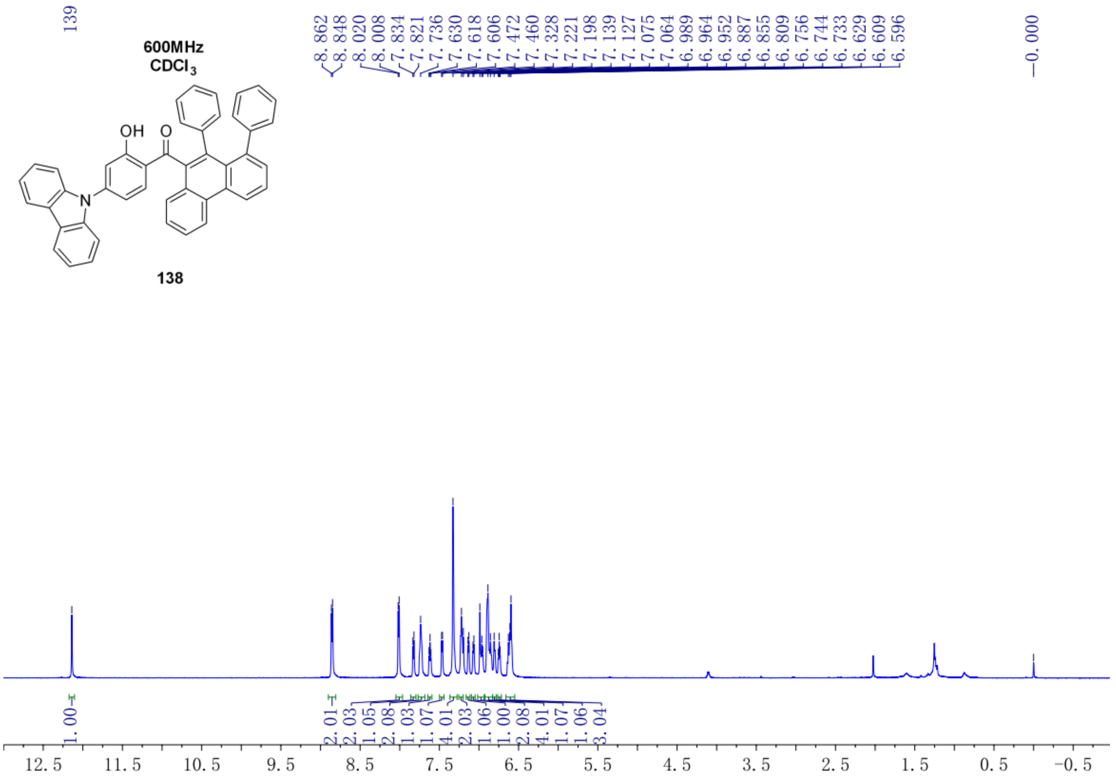
139
600MHz
CDCl3
OH
O
N
138
8.862
8.848
8.020
8.008
7.834
7.821
7.736
7.630
7.618
7.606
7.472
7.460
7.328
7.221
7.198
7.139
7.127
7.075
7.064
6.989
6.964
6.952
6.887
6.855
6.809
6.756
6.744
6.733
6.629
6.609
6.596
0.000
1.00
2.01
2.03
1.05
2.08
1.03
1.07
4.01
2.03
1.06
1.00
2.08
4.01
1.07
1.06
3.04
12.5
11.5
10.5
9.5
8.5
7.5
6.5
5.5
4.5
3.5
2.5
1.5
0.5
-0.5

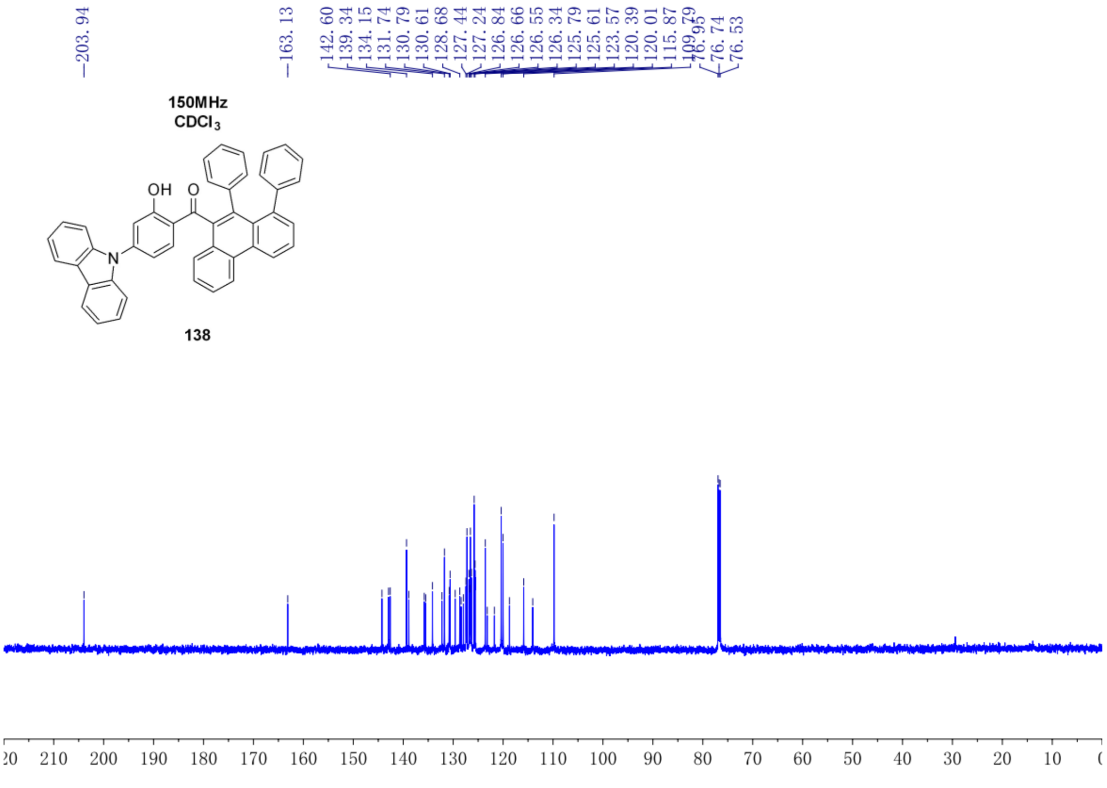
203.94
163.13
142.60
139.34
134.15
131.74
130.79
130.61
128.68
127.44
127.24
126.84
126.66
126.55
126.34
125.79
125.61
123.57
120.39
120.01
115.87
109.79
76.95
76.74
76.53
150MHz
CDCl3
OH
O
N
138
210
200
190
180
170
160
150
140
130
120
110
100
90
80
70
60
50
40
30
20
10

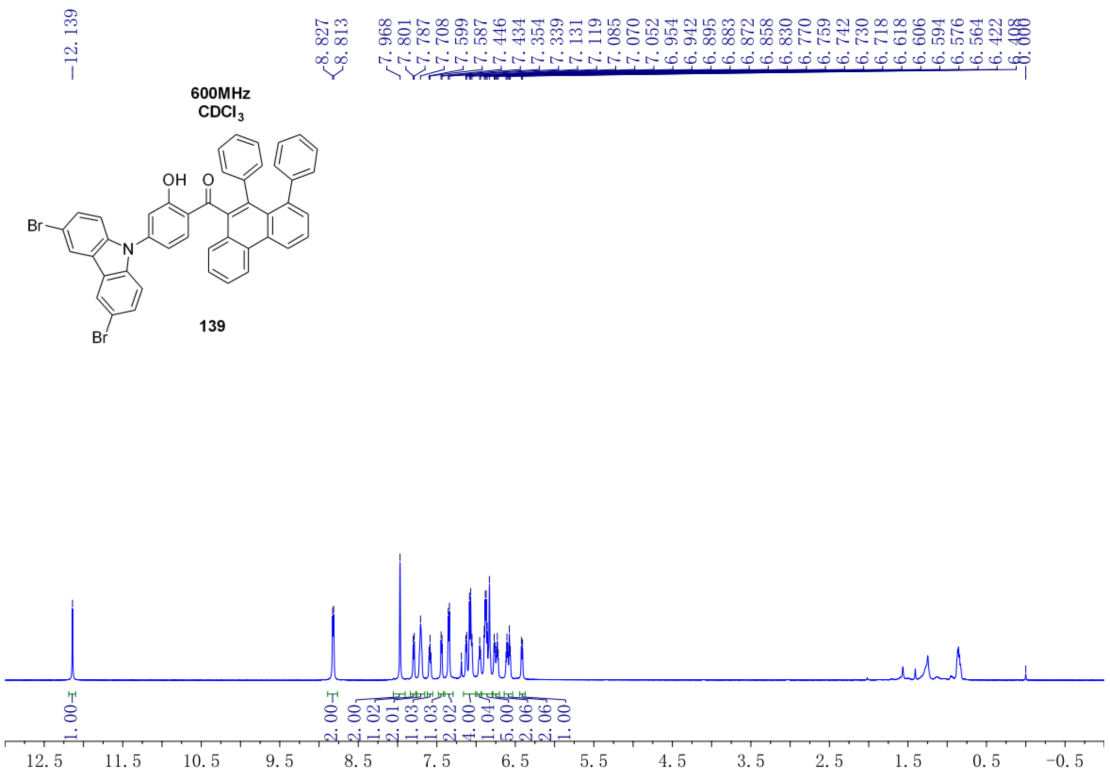
12.139
8.827
8.813
7.968
7.801
7.787
7.708
7.599
7.587
7.446
7.434
7.354
7.339
7.131
7.119
7.085
7.070
7.052
6.954
6.942
6.895
6.883
6.872
6.858
6.830
6.770
6.759
6.742
6.730
6.718
6.618
6.606
6.594
6.576
6.564
6.422
6.408
0.000
600MHz
CDCl3
OH
O
Br
N
Br
139
1.00
2.00
2.00
1.02
2.01
1.03
1.03
2.02
4.00
1.04
5.00
2.06
2.06
1.00
12.5
11.5
10.5
9.5
8.5
7.5
6.5
5.5
4.5
3.5
2.5
1.5
0.5
-0.5

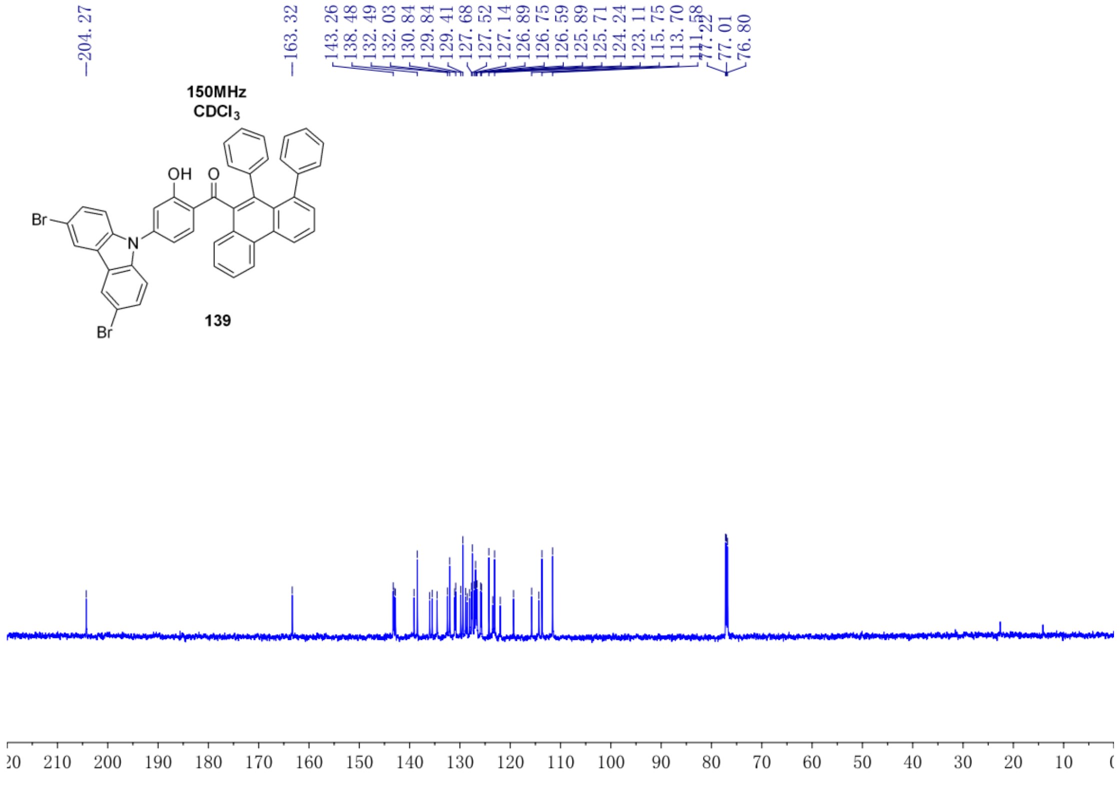
204.27
163.32
143.26
138.48
132.49
132.03
130.84
129.84
129.41
127.68
127.52
127.14
126.89
126.75
126.59
125.89
125.71
124.24
123.11
115.75
113.70
111.58
77.22
77.01
76.80
150MHz
CDCl3
OH
O
Br
N
Br
139
210
200
190
180
170
160
150
140
130
120
110
100
90
80
70
60
50
40
30
20
10

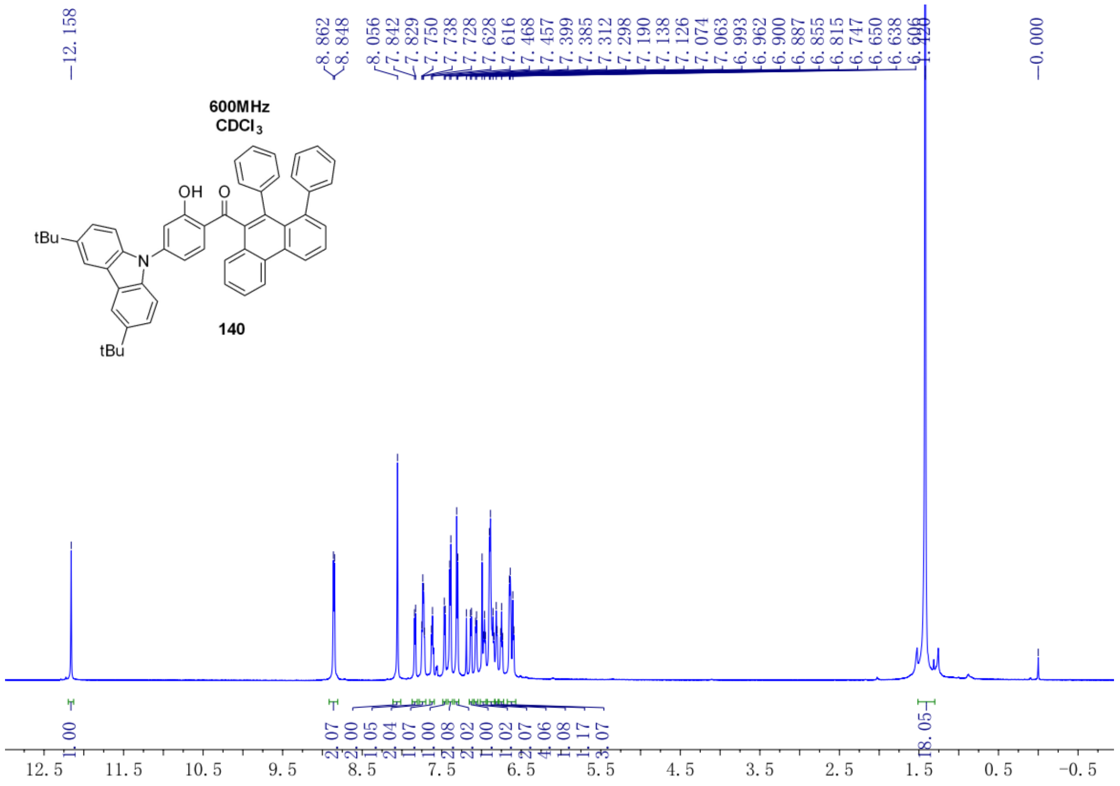
600MHz
CDCl3
OH
O
tBu
N
tBu
140
12.158
8.862
8.848
8.056
7.842
7.829
7.750
7.738
7.728
7.628
7.616
7.468
7.457
7.399
7.385
7.312
7.298
7.190
7.138
7.126
7.074
7.063
6.993
6.962
6.900
6.887
6.855
6.815
6.747
6.650
6.638
6.606
0.000
1.00
2.07
2.00
2.04
1.05
1.07
1.00
2.08
2.02
1.00
1.02
2.07
4.06
1.08
1.17
3.07
18.05
12.5
11.5
10.5
9.5
8.5
7.5
6.5
5.5
4.5
3.5
2.5
1.5
0.5
-0.5

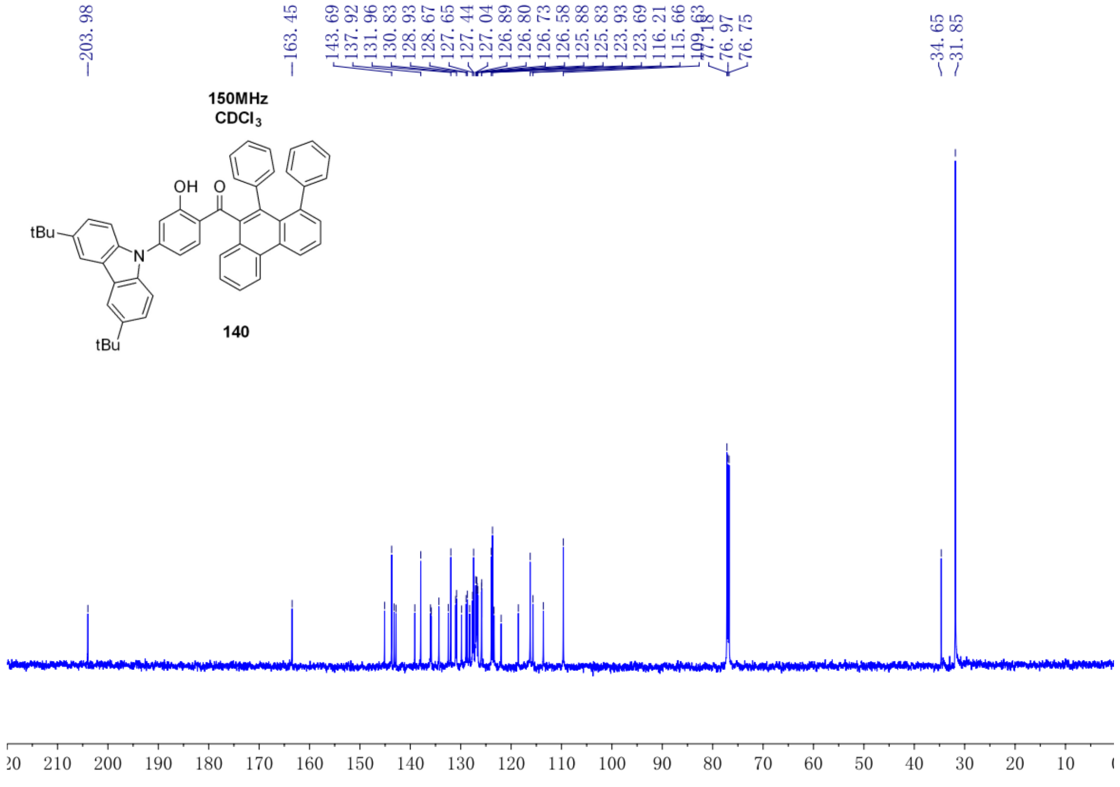
150MHz
CDCl3
OH
O
tBu
N
tBu
140
203.98
163.45
143.69
137.92
131.96
130.83
128.93
128.67
127.65
127.44
127.04
126.89
126.80
126.73
126.58
125.88
125.83
123.93
123.69
116.21
115.66
109.63
76.97
76.75
34.65
31.85
210
200
190
180
170
160
150
140
130
120
110
100
90
80
70
60
50
40
30
20
10

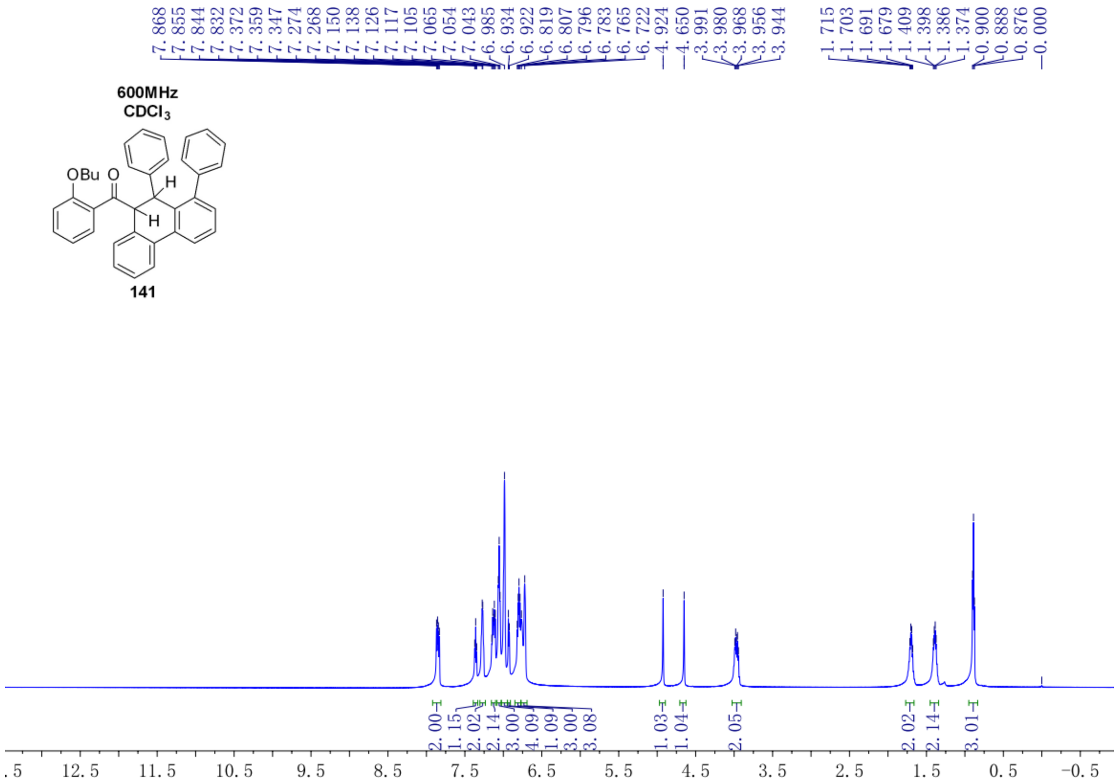
7.868 7.855 7.844 7.832 7.372 7.359 7.347 7.274 7.268 7.150 7.138 7.126 7.117 7.105 7.065 7.054 7.043 6.985 6.934 6.922 6.819 6.807 6.796 6.783 6.765 6.722 4.924 4.650 3.991 3.980 3.968 3.956 3.944 1.715 1.703 1.691 1.679 1.409 1.398 1.386 1.374 0.900 0.888 0.876 0.000
600MHz
CDCl3
OBu
O
H
H
141
2.00 1.15 2.02 2.14 3.00 4.09 1.09 3.00 3.08 1.03 1.04 2.05 2.02 2.14 3.01

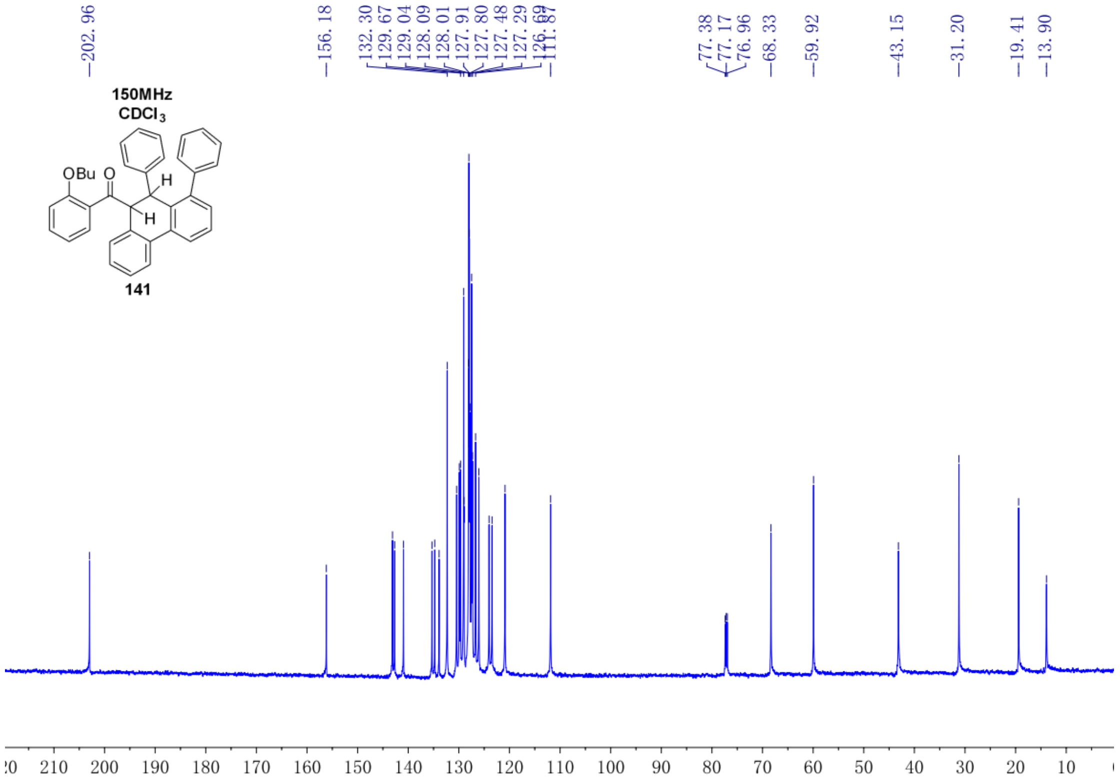
202.96 156.18 132.30 129.67 129.04 128.09 128.01 127.91 127.80 127.48 127.29 126.69 111.87 77.38 77.17 76.96 68.33 59.92 43.15 31.20 19.41 13.90
150MHz
CDCl3
OBu
O
H
H
141

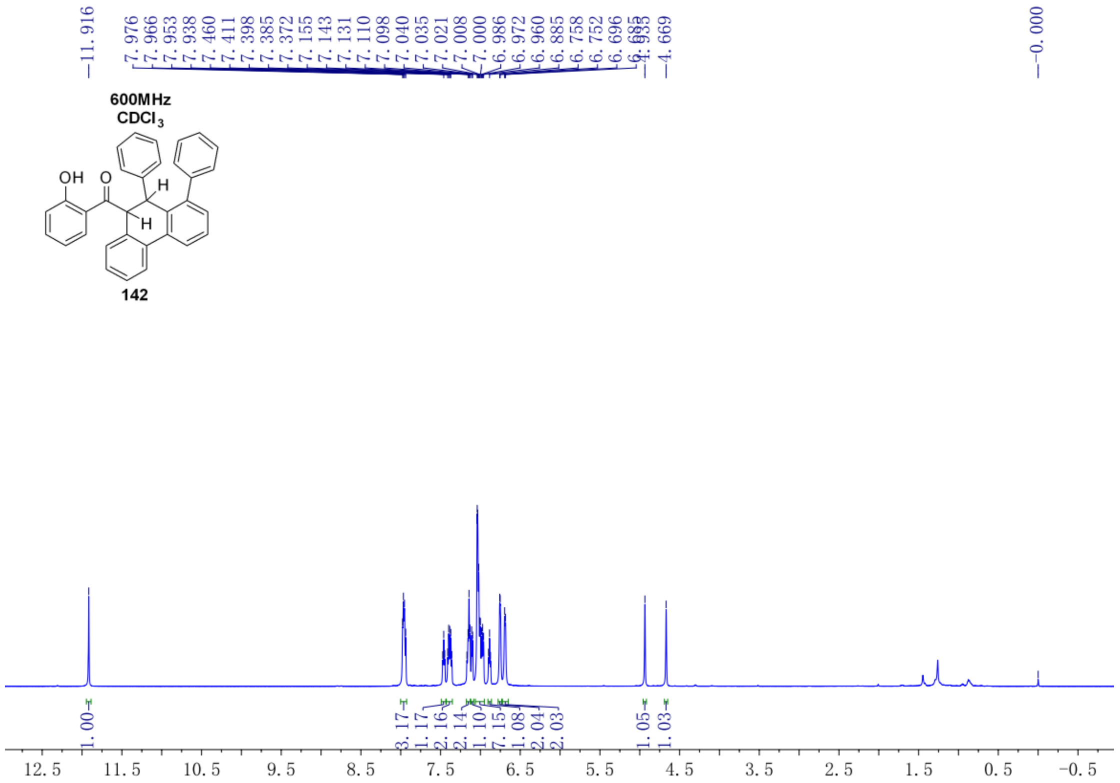

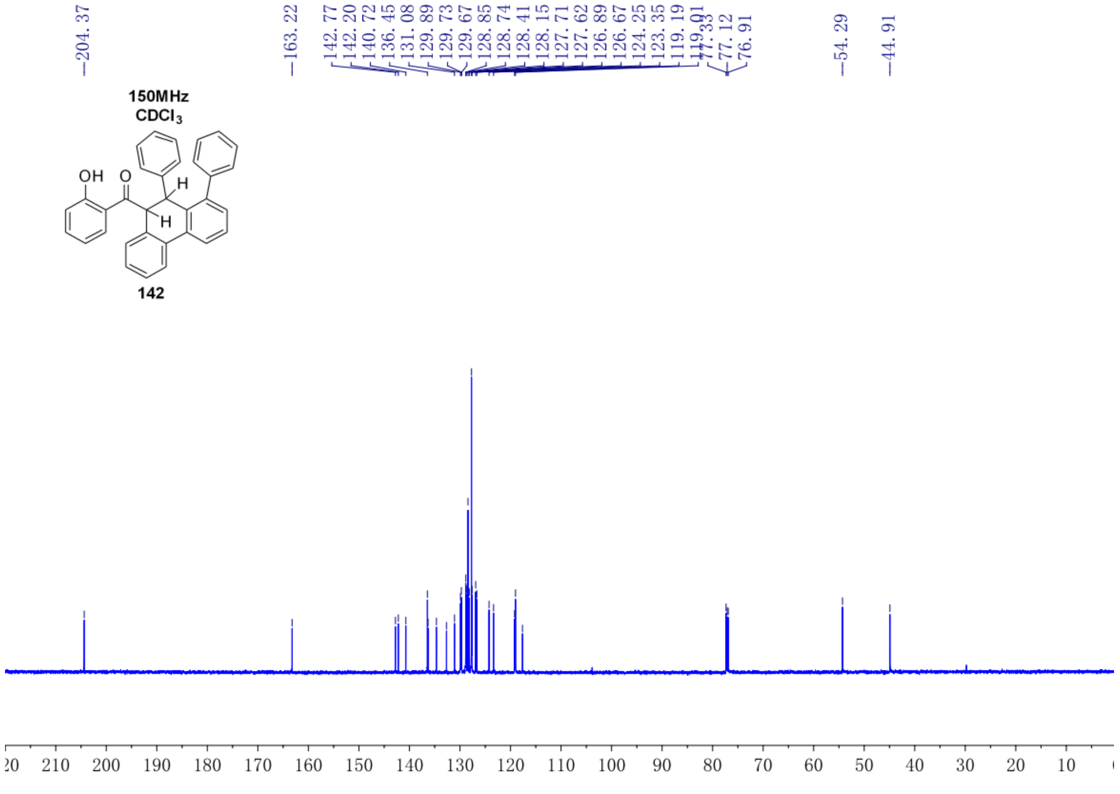

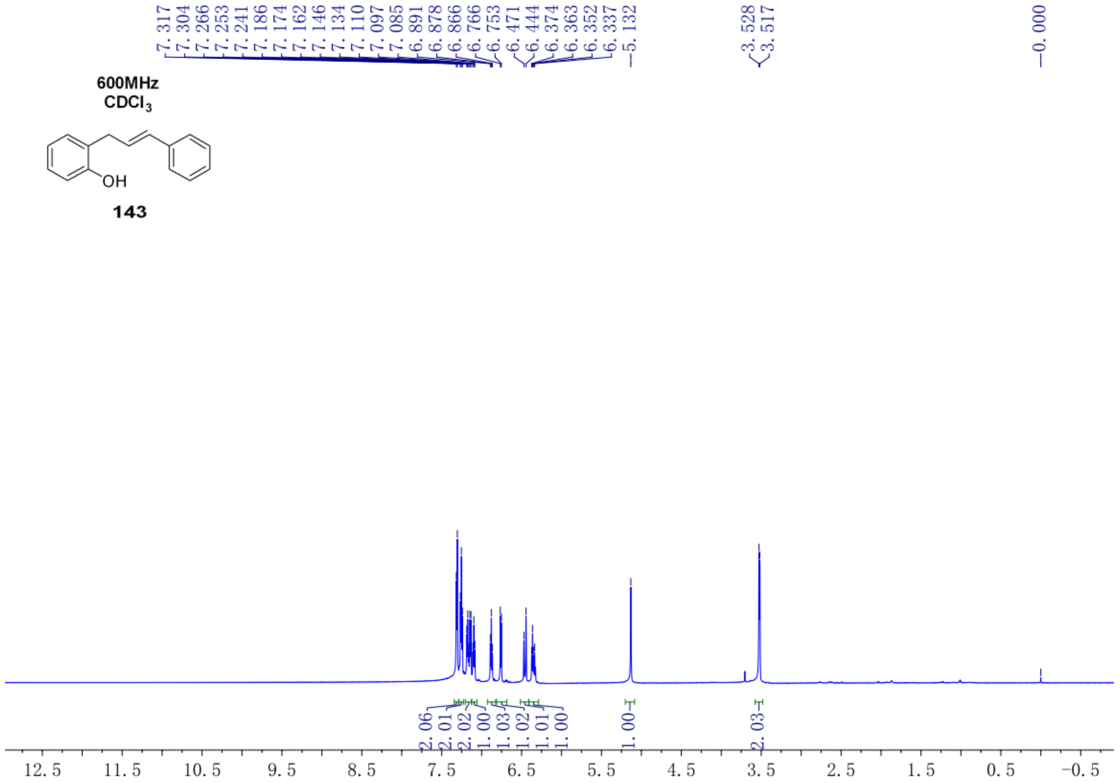
600MHz
$CDCl_3$
OH
143
7.317 7.304 7.266 7.253 7.241 7.186 7.174 7.162 7.146 7.134 7.110 7.097 7.085 6.891 6.878 6.866 6.766 6.753 6.471 6.444 6.374 6.363 6.352 6.337 5.132
3.528 3.517
0.000
2.06 2.01 2.02 1.00 1.03 1.02 1.01 1.00
1.00
2.03
12.5 11.5 10.5 9.5 8.5 7.5 6.5 5.5 4.5 3.5 2.5 1.5 0.5 -0.5

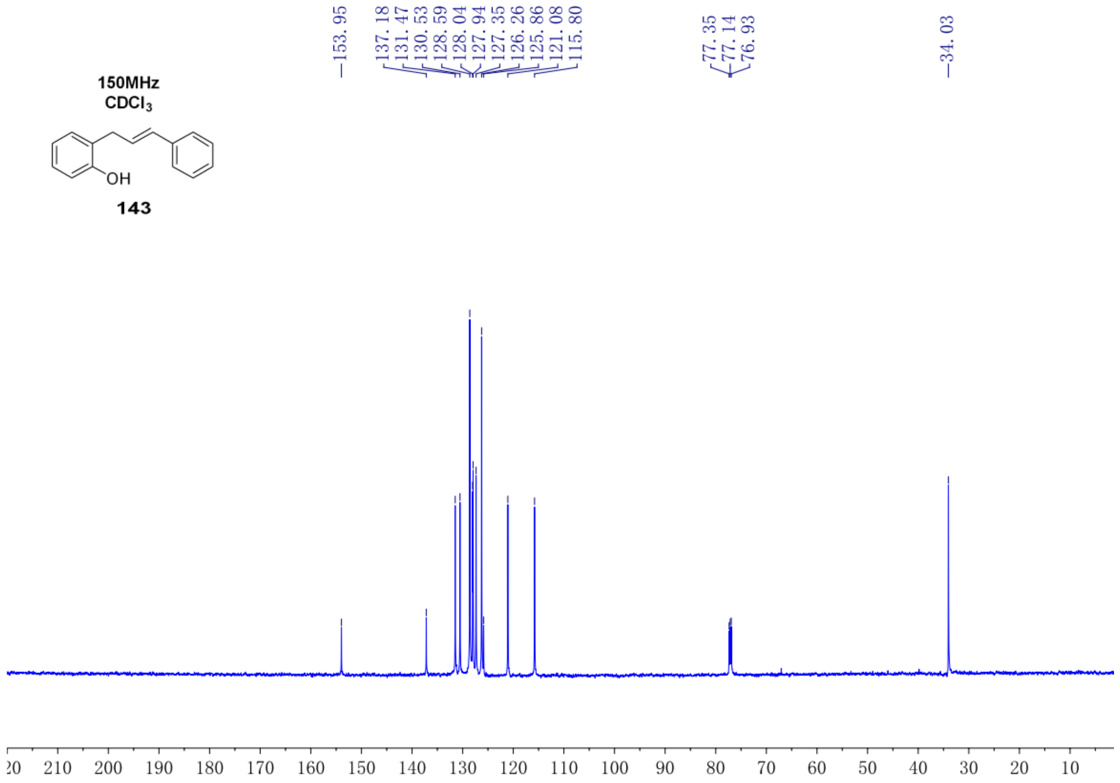
150MHz
$CDCl_3$
OH
143
153.95
137.18 131.47 130.53 128.59 128.04 127.94 127.35 126.26 125.86 121.08 115.80
77.35 77.14 76.93
34.03
210 200 190 180 170 160 150 140 130 120 110 100 90 80 70 60 50 40 30 20 10

5. Supplementary References

(1) Alcantara, A.-R., Marinas, J.-M. & Sinisterra, J.-V. Synthesis of 2'-hydroxychalcones and related compounds in interfacial solid-liquid conditions. *Tetrahedron Letters*, **28**, 1515-1518 (1987).

(2) Muller, B.-M., Litberg, T.-J. & Adler, M.-J. Extended Aromatic and Heteroaromatic Ring Systems in the Chalcone–Flavanone Molecular Switch Scaffold. *J. Org. Chem.* **81**, 5775-5781 (2016).

(3) Bhunia, A., Patra, A. & Biju, A.T. NHC-Catalyzed Reaction of Enals with Hydroxy Chalcones: Diastereoselective Synthesis of Functionalized Coumarins. *Org. Lett.* **15**, 1756-1759 (2013).
