# Supplementary material for: Quadruple C-H activation coupled to hydrofunctionalization and C-H silylation/borylation enabled by weakly coordinated palladium catalyst
Source: Nat Commun. 2020 Nov 9;11:5662. doi: 10.1038/s41467-020-19508-z (PMC7652853; doi:10.1038/s41467-020-19508-z)
Supplement: Supplementary file 3 — Description of Additional Supplementary Files [file 41467_2020_19508_MOESM3_ESM.pdf]

Description of Additional Supplementary Files

File Name: Supplementary Data 1
Description: Computational details
