# Supplementary material for: Quadruple C-H activation coupled to hydrofunctionalization and C-H silylation/borylation enabled by weakly coordinated palladium catalyst
Source: Nat Commun. 2020 Nov 9;11:5662. doi: 10.1038/s41467-020-19508-z (PMC7652853; doi:10.1038/s41467-020-19508-z)
Supplement: Supplementary file 4 — Supplementary Data 1 [file 41467_2020_19508_MOESM4_ESM.zip › 257266_3_data_set_4978237_qhzz8v.docx]

**Computational details**

**Complete reference for Gaussian 09**

Gaussian 09, Revision D.01, Frisch, M. J.; Trucks, G. W.; Schlegel, H. B.; Scuseria, G. E.; Robb, M. A.; Cheeseman, J. R.; Scalmani, G.; Barone, V.; Mennucci, B.; Petersson, G. A.; Nakatsuji, H.; Caricato, M.; Li, X.; Hratchian, H. P.; Izmaylov, A. F.; Bloino, J.; Zheng, G.; Sonnenberg, J. L.; Hada, M.; Ehara, M.; Toyota, K.; Fukuda, R.; Hasegawa, J.; Ishida, M.; Nakajima, T.; Honda, Y.; Kitao, O.; Nakai, H.; Vreven, T.; Montgomery, Jr., J. A.; Peralta, J. E.; Ogliaro, F.; Bearpark, M.; Heyd, J. J.; Brothers, E.; Kudin, K. N.; Staroverov, V. N.; Keith, T.; Kobayashi, R.; Normand, J.; Raghavachari, K.; Rendell, A.; Burant, J. C.; Iyengar, S. S.; Tomasi, J.; Cossi, M.; Rega, N.; Millam, J. M.; Klene, M.; Knox, J. E.; Cross, J. B.; Bakken, V.; Adamo, C.; Jaramillo, J.; Gomperts, R.; Stratmann, R. E.; Yazyev, O.; Austin, A. J.; Cammi, R.; Pomelli, C.; Ochterski, J. W.; Martin, R. L.; Morokuma, K.; Zakrzewski, V. G.; Voth, G. A.; Salvador, P.; Dannenberg, J. J.; Dapprich, S.; Daniels, A. D.; Farkas, O.; Foresman, J. B.; Ortiz, J. V.; Cioslowski, J.; and Fox, D. J. Gaussian 09, revision D.01; Gaussian, Inc.: Wallingford, CT, 2013.

**Computational Methods**

All the DFT calculations were carried out with the GAUSSIAN 09 series of programs. Density functional theory M06-L^1^ with a standard 6-31G(d) basis set (SDD basis set for Pd and I atoms) was used for geometry optimizations. Harmonic frequency calculations were performed for all stationary points to confirm them as a local minima or transition structures and to derive the thermochemical corrections for free energies. M06-L functional with basis set 6-311++G(d,p) (SDD basis set for Pd and I atoms) was employed to calculate the solvation single point energies to give more accurate energy information. The solvent effects were considered in single point calculations and geometry optmizations with a SMD continuum solvation model^2-3^.

**M06-L calculated absolute energies, enthalpies, and free energies of all structures**

| **Geometry** | **E(elec-M06-L)^1^** | **H(corr-M06-L)^2^** | **G(corr-M06-L)^3^** | **IF^5^** |
| --- | --- | --- | --- | --- |
| **I** | -625.092363 | 0.224405 | 0.16456 |  |
| **II** | -619.630259 | 0.209905 | 0.145859 |  |
| **TS_II-III_** | -619.630259 | 0.209905 | 0.146958 | -92.75 |
| **III** | -619.668519 | 0.210625 | 0.149772 |  |
| **IV** | -1793.760072 | 0.60574 | 0.495725 |  |
| **TS_IV-V_** | -1793.715786 | 0.599497 | 0.491779 | -1532.34 |
| **V** | -1793.770547 | 0.604951 | 0.494482 |  |
| **VI** | -1778.582646 | 0.551367 | 0.449295 |  |
| **TS_VI-VII_** | -2007.219969 | 0.601449 | 0.48744 | -1375.53 |
| **TS'_VI-VII_** | -2007.214015 | 0.601807 | 0.49131 | -1114.12 |
| **VII** | -1778.093026 | 0.536563 | 0.437931 |  |
| **VIII** | -1778.116963 | 0.53738 | 0.43663 |  |
| **TS_VIII-IX_** | -1778.084691 | 0.531235 | 0.429022 | -710.52 |
| **IX** | -1778.104425 | 0.533456 | 0.432064 |  |
| **TS_IX-II_** | -1778.057534 | 0.531968 | 0.430287 | -974.19 |
| **X** | -2007.269615 | 0.602085 | 0.489799 |  |
| **TS_X-II_** | -2007.242554 | 0.596898 | 0.486889 | -1328.73 |
| **IV'** | -1100.495997 | 0.346328 | 0.263859 |  |
| **TS_IV'-V'_** | -1100.468519 | 0.344847 | 0.266025 | -380.30 |
| **V'** | -1100.501534 | 0.34751 | 0.268863 |  |
| **VII'c** | -1317.585702 | 0.401312 | 0.316533 |  |
| **TS_VI'-VII'a_** | -1317.578112 | 0.397528 | 0.31637 | -416.84 |
| **TS_V'-VII'b_** | -1100.476729 | 0.341475 | 0.260899 | -745.68 |
| **VII'a** | -1317.613422 | 0.401487 | 0.318333 |  |
| **VII'b** | -637.168829 | 0.236544 | 0.169644 |  |
| **VIII'a** | -1317.138917 | 0.388354 | 0.305345 |  |
| **VIII'b** | -1317.113853 | 0.382353 | 0.302123 |  |
| **TS_VIII'a-IX'_** | -1317.113853 | 0.382353 | 0.302123 | -1425.68 |
| **TS_VIII'b-I_** | -854.23894 | 0.287168 | 0.215265 | -955.95 |
| **IX'** | -1087.974686 | 0.374927 | 0.290925 |  |
| **1** | -729.383084 | 0.245154 | 0.188642 |  |
| **X'** | -960.459782 | 0.330776 | 0.263984 |  |
| **142** | -1422.639275 | 0.415923 | 0.502952 |  |
| **4** | -1421.446163 | 0.394452 | 0.478781 |  |
| **VI'** | -1317.602281 | 0.400394 | 0.315942 |  |
| **TS_VI'-VII'c_** | -1317.574471 | 0.400777 | 0.318583 | -98.99 |

^1^The electronic energy calculated by M06-L in DMF solvent. ^2^The thermal correction to enthalpy calculated by M06-L in DMF solvent. ^3^The thermal correction to Gibbs free energy calculated by M06-L in DMF solvent. ^4^The M06-L calculated imaginary frequencies for the transition states.

**M06-L geometries for all the optimized compounds and transition states**

**I**

Pd 0.00000000 0.00000000 0.65609400

O 0.45135100 2.06561400 0.79744000

C 0.00000000 3.13245100 0.34558300

N -0.97262400 3.30614300 -0.55980300

H 0.42242400 4.08264200 0.70635000

C -1.36314500 4.64119900 -0.96320200

C -1.67058100 2.21657600 -1.20529400

H -1.19462300 4.77818200 -2.03851400

H -2.42766000 4.80338100 -0.75435100

H -0.77656300 5.38316000 -0.41402700

H -1.49026900 2.24373200 -2.28733900

H -1.31228200 1.25495200 -0.79861600

H -2.74926400 2.30567400 -1.02708500

O -0.45135100 -2.06561400 0.79744000

C 0.00000000 -3.13245100 0.34558300

N 0.97262400 -3.30614300 -0.55980300

H -0.42242400 -4.08264200 0.70635000

C 1.36314500 -4.64119900 -0.96320200

C 1.67058100 -2.21657600 -1.20529400

H 1.19462300 -4.77818200 -2.03851400

H 2.42766000 -4.80338100 -0.75435100

H 0.77656300 -5.38316000 -0.41402700

H 1.49026900 -2.24373200 -2.28733900

H 1.31228200 -1.25495200 -0.79861600

H 2.74926400 -2.30567400 -1.02708500

**II**

Pd 0.24208200 0.55237700 -0.12953700

C -1.72205000 0.40317600 0.09656900

C -1.87925500 1.12032200 -1.11754000

C -2.17033900 0.94346100 1.32452300

C -2.45671100 2.40282200 -1.06963900

H -1.68874700 0.64164700 -2.07556500

C -2.75177000 2.20509000 1.32707100

H -2.06418300 0.37347400 2.24503300

C -2.89837200 2.93222600 0.13643600

H -2.58279900 2.96190800 -1.99619900

H -3.09002500 2.63163500 2.27042000

H -3.36827300 3.91380200 0.15860200

I -1.60574200 -1.91706900 -0.02124400

O 2.43660600 0.80411900 -0.21274500

C 3.22592200 -0.14541700 -0.03785200

N 4.54620100 -0.00843500 0.10651800

H 2.88136500 -1.19134100 0.01106900

C 5.40703400 -1.15635400 0.29638200

C 5.18287500 1.29191900 0.07502000

H 6.14202400 -1.21725000 -0.51525200

H 5.94527400 -1.07082500 1.24794800

H 4.80902300 -2.07130900 0.30595400

H 5.89802600 1.33943100 -0.75479600

H 4.42204300 2.06250600 -0.05448500

H 5.72455900 1.46430300 1.01243500

**TS_II-III_**

Pd 0.24208200 0.55237700 -0.12953700

C -1.72205000 0.40317600 0.09656900

C -1.87925500 1.12032200 -1.11754000

C -2.17033900 0.94346100 1.32452300

C -2.45671100 2.40282200 -1.06963900

H -1.68874700 0.64164700 -2.07556500

C -2.75177000 2.20509000 1.32707100

H -2.06418300 0.37347400 2.24503300

C -2.89837200 2.93222600 0.13643600

H -2.58279900 2.96190800 -1.99619900

H -3.09002500 2.63163500 2.27042000

H -3.36827300 3.91380200 0.15860200

I -1.60574200 -1.91706900 -0.02124400

O 2.43660600 0.80411900 -0.21274500

C 3.22592200 -0.14541700 -0.03785200

N 4.54620100 -0.00843500 0.10651800

H 2.88136500 -1.19134100 0.01106900

C 5.40703400 -1.15635400 0.29638200

C 5.18287500 1.29191900 0.07502000

H 6.14202400 -1.21725000 -0.51525200

H 5.94527400 -1.07082500 1.24794800

H 4.80902300 -2.07130900 0.30595400

H 5.89802600 1.33943100 -0.75479600

H 4.42204300 2.06250600 -0.05448500

H 5.72455900 1.46430300 1.01243500

**III**

O 1.93302500 -1.04129100 -1.42459600

C 3.04481600 -0.90645000 -0.87935400

N 3.33547600 -1.06730200 0.41338700

H 3.92303100 -0.64048300 -1.48892000

C 4.67444900 -0.81448400 0.90786600

C 2.36680200 -1.50972100 1.39587500

H 4.65027600 -0.02672600 1.66995700

H 5.08803700 -1.72461000 1.35801900

H 5.31867100 -0.49434400 0.08525300

H 2.09305500 -0.68978200 2.07135700

H 1.46399300 -1.87764700 0.89698100

H 2.80597800 -2.32123700 1.98714800

Pd -0.11381400 -0.65369700 -0.61606700

C -1.96038500 -0.48596100 -0.03118300

I 0.34384400 1.86646700 0.15297300

C -2.30311800 -0.79550200 1.28811200

C -2.95068600 -0.26708600 -0.99280500

C -3.64826000 -0.96917000 1.62216000

H -1.53149200 -0.91579100 2.04923600

C -4.29225300 -0.44464400 -0.64511800

H -2.68462700 0.02251600 -2.00954500

C -4.64092900 -0.79638900 0.65808800

H -3.91682500 -1.22990100 2.64534800

H -5.06489400 -0.29598900 -1.39876000

H -5.68868300 -0.92079200 0.92666300

**IV**

C -1.89562500 4.71695800 -2.61898000

C -0.56164000 4.50452300 -2.96081400

C 0.13873700 3.46220000 -2.37112800

C -0.47301300 2.59085400 -1.45411500

C -1.82182100 2.83220200 -1.12940400

C -2.52551500 3.88729600 -1.69843900

H -2.45501200 5.53487400 -3.06746000

H -0.06888700 5.14933700 -3.68394100

H 1.17620000 3.29273500 -2.64929000

H -3.56295500 4.06093600 -1.41516300

C 0.28275700 1.44780200 -0.90239600

C 1.74021200 1.64989400 -0.52181500

H 2.24674100 2.21367000 -1.31508800

O -0.23982000 0.34552900 -0.68734600

O -2.42022000 2.04344700 -0.16999500

H -3.32953700 2.36350300 -0.00455600

C 2.46333600 0.31184100 -0.31120800

H 2.18059800 -0.31434200 -1.16380200

C 3.97142000 0.46858400 -0.37649200

C 4.62155300 0.30977400 -1.60558000

C 4.73976300 0.76353800 0.75472600

C 6.00366400 0.44532500 -1.70561200

H 4.03179200 0.06740600 -2.49100400

C 6.12265800 0.90343700 0.65681000

H 4.25482600 0.87337900 1.72537000

C 6.75917200 0.74382100 -0.57269300

H 6.49276600 0.30910300 -2.66902600

H 6.70563000 1.13303600 1.54744800

H 7.84059000 0.84532300 -0.64663500

C 2.00599800 -0.38465400 0.95284500

C 1.51684300 0.37158100 2.03824200

C 2.12946700 -1.78136700 1.07108300

C 1.05850800 -0.29606400 3.18424400

C 1.70616700 -2.41373800 2.24517800

C 1.14644000 -1.67768400 3.28512300

H 0.62899600 0.27262500 4.00686700

H 1.80032600 -3.49613600 2.32717600

H 0.78889700 -2.18209000 4.18105800

C 2.75484900 -2.59545800 -0.00349300

C 3.99152600 -3.21177100 0.22792700

C 2.14926800 -2.75835000 -1.25705600

C 4.61586400 -3.95372600 -0.77220100

H 4.47412500 -3.08691500 1.19771700

C 2.77016300 -3.50470300 -2.25510000

H 1.18173400 -2.29205800 -1.44705400

C 4.00874800 -4.09945700 -2.01791600

H 5.58206500 -4.41691000 -0.57780900

H 2.28387700 -3.62153700 -3.22242200

H 4.49776000 -4.67593100 -2.80131600

C 1.56130900 1.84517900 1.97586300

C 1.72421600 2.49296900 0.73492100

C 1.52637300 2.63882700 3.13358000

C 1.82439900 3.88229900 0.66986000

C 1.62325000 4.02349600 3.06146900

H 1.44517000 2.16548500 4.11013800

C 1.77485200 4.65286800 1.82674400

H 1.94646800 4.35895800 -0.30298800

H 1.59457600 4.61270100 3.97625200

H 1.86225400 5.73582900 1.76603600

Pd -2.35045900 -0.19948900 -0.26329100

C -5.20411700 -0.80523100 -0.72379700

C -6.56196600 -0.71390400 -0.40951200

C -6.96977500 -0.21583800 0.82726200

C -6.01577000 0.19604800 1.75536300

C -4.65417500 0.11236100 1.45136400

C -4.25040700 -0.37264100 0.20302700

H -4.89781900 -1.20630600 -1.68951300

H -7.30179900 -1.04543400 -1.13760300

H -8.02943100 -0.15636300 1.06975000

H -6.32519500 0.58029400 2.72688800

H -3.91657600 0.43121300 2.18913300

I -2.04741300 -2.83502300 -0.14898700

**TS_IV-V_**

C -2.23156600 4.25204300 -2.30516900

C -1.04287100 3.97134200 -2.97953700

C -0.20398700 2.96822700 -2.50934000

C -0.55558200 2.19870900 -1.39141400

C -1.75735400 2.50597000 -0.70514200

C -2.58325300 3.53302100 -1.16949300

H -2.88790300 5.04324400 -2.66254600

H -0.76994800 4.53236900 -3.86998200

H 0.72046700 2.74598300 -3.04049600

H -3.49345100 3.76358200 -0.61886800

C 0.33545000 1.09970700 -0.95329700

C 1.82059000 1.39852800 -0.80359500

H 2.18931700 1.76055400 -1.77466300

O -0.07878000 -0.03526600 -0.66177700

O -2.05493100 1.86952000 0.45849300

H -3.23892700 1.36254100 0.54326700

C 2.59894500 0.14095900 -0.38519300

H 2.26116400 -0.65393000 -1.05689300

C 4.09197700 0.28706200 -0.60991100

C 4.64891100 -0.17953700 -1.80595400

C 4.93761600 0.87285700 0.33820600

C 6.01280800 -0.05443800 -2.05666900

H 3.99988700 -0.65340000 -2.54435800

C 6.30200300 1.00329400 0.08820500

H 4.52824000 1.22241300 1.28595900

C 6.84417100 0.54131600 -1.10965600

H 6.42777800 -0.43038300 -2.99060400

H 6.94585900 1.46244200 0.83691700

H 7.91174200 0.63751100 -1.30080800

C 2.24494700 -0.25854400 1.03416400

C 1.87327400 0.73377800 1.96680100

C 2.32456600 -1.60333800 1.44107800

C 1.50597800 0.35302000 3.26615100

C 1.99646400 -1.94530200 2.75854400

C 1.56451300 -0.97652400 3.65917400

H 1.16827300 1.10604200 3.97576400

H 2.05906100 -2.98876500 3.06530900

H 1.28049200 -1.25842900 4.67140900

C 2.78298900 -2.67913100 0.52457900

C 3.96400100 -3.37724200 0.80608500

C 2.04480700 -3.04187400 -0.61034300

C 4.40408900 -4.39971300 -0.03145600

H 4.54638000 -3.10324900 1.68605100

C 2.48197000 -4.06570400 -1.44583800

H 1.11521300 -2.51822600 -0.83198600

C 3.66536500 -4.74564000 -1.16113500

H 5.32817600 -4.92731800 0.20029500

H 1.89207000 -4.33678000 -2.32008200

H 4.00843800 -5.54524100 -1.81562100

C 1.93900100 2.15516500 1.57867500

C 1.96373100 2.50779400 0.21512900

C 2.05635300 3.17992100 2.53077400

C 2.08517100 3.84309100 -0.16630100

C 2.16756000 4.50978400 2.14414100

H 2.08468700 2.93244700 3.59007000

C 2.18451600 4.84753100 0.79135500

H 2.10473300 4.09233500 -1.22736300

H 2.25792800 5.28528600 2.90258600

H 2.28459200 5.88662100 0.48348600

Pd -2.19357200 -0.19718100 -0.07533800

C -5.10782200 0.44181800 -0.42427300

C -6.45068700 0.35450900 -0.07758000

C -6.80871200 0.06782100 1.24130700

C -5.83162300 -0.11849100 2.22184400

C -4.48768800 -0.02855700 1.88223700

C -4.10650700 0.27518400 0.55713500

H -4.82311000 0.66107800 -1.45316800

H -7.22034100 0.49923600 -0.83326700

H -7.86127600 -0.01113500 1.50831200

H -6.12180200 -0.33994500 3.24717300

H -3.72180300 -0.17041600 2.64383700

I -2.73672800 -2.73718300 -0.66554100

**V**

C -1.45078600 5.04112800 -2.50502600

C -0.06942800 4.78897300 -2.56469300

C 0.43501900 3.66984500 -1.94453400

C -0.39332100 2.73868300 -1.26123800

C -1.81361100 2.99020100 -1.22769600

C -2.29557400 4.16810200 -1.85963600

H -1.86419700 5.92797700 -2.98352700

H 0.59359700 5.46234700 -3.10176800

H 1.50160800 3.47797400 -2.01917400

H -3.36613400 4.35357400 -1.80911300

C 0.19355200 1.59965500 -0.59978000

C 1.65766300 1.61301100 -0.16792300

H 2.21387300 2.29338800 -0.81755000

O -0.42085100 0.55370100 -0.25161600

O -2.70685400 2.27214600 -0.61171100

C 2.32254800 0.22666400 -0.26008700

H 1.96284100 -0.22219600 -1.19250400

C 3.82855800 0.36924700 -0.40453100

C 4.39704600 0.41567300 -1.68243300

C 4.67069500 0.46901300 0.70758100

C 5.77176000 0.56626700 -1.84677500

H 3.74864100 0.32299100 -2.55542900

C 6.04592000 0.62070400 0.54626500

H 4.24925200 0.41347000 1.71146200

C 6.60106200 0.67066200 -0.73112400

H 6.19722100 0.59358000 -2.84887000

H 6.68679500 0.69408000 1.42351100

H 7.67654800 0.78349400 -0.85683900

C 1.95387800 -0.69081500 0.88320600

C 1.57509900 -0.15551900 2.13217200

C 2.08934800 -2.08298900 0.72735200

C 1.24765200 -1.03363000 3.17680700

C 1.80729500 -2.92910800 1.80475100

C 1.36359000 -2.40753600 3.01623600

H 0.90198500 -0.64018500 4.13029500

H 1.91758600 -4.00558600 1.67711100

H 1.11383900 -3.07362200 3.84016100

C 2.56763800 -2.67422400 -0.54975800

C 3.82661500 -3.28468700 -0.61032800

C 1.78572000 -2.63265000 -1.71252000

C 4.30074200 -3.82145000 -1.80547700

H 4.44493300 -3.31758800 0.28714400

C 2.25739400 -3.17227800 -2.90627800

H 0.79651000 -2.17391400 -1.67438400

C 3.51906800 -3.76344100 -2.95762800

H 5.28656200 -4.28295600 -1.83640800

H 1.63569600 -3.13279800 -3.79928100

H 3.89082900 -4.17892100 -3.89265800

C 1.59620200 1.30597400 2.33782100

C 1.68080100 2.17869300 1.23444300

C 1.60776200 1.87309800 3.62323400

C 1.74428600 3.55914000 1.42715600

C 1.66831600 3.24895500 3.80754800

H 1.59223600 1.22763500 4.49862100

C 1.73679200 4.10178700 2.70708700

H 1.80184600 4.21161300 0.55533300

H 1.67523400 3.65554600 4.81731500

H 1.79328200 5.17971900 2.84508600

Pd -2.46311100 0.28930800 -0.22998200

C -5.23046300 -1.06776200 -1.34101500

C -5.70831400 -1.97091000 -0.41104000

C -5.69877100 -1.65542300 0.95996000

C -5.21184500 -0.43799000 1.39555100

C -4.73796900 0.50605000 0.46112900

C -4.74735700 0.18879400 -0.91951300

H -5.25350500 -1.30307000 -2.40296800

H -6.10001200 -2.93207000 -0.73901000

H -6.08403600 -2.37559000 1.67924900

H -5.22065800 -0.18498600 2.45354100

H -4.53075100 1.52426500 0.78387200

I -2.03307400 -2.33880100 0.31876600

H -4.55142200 0.96387400 -1.65764600

**VI**

C -3.39985100 3.88943000 -2.57503800

C -2.02065200 4.15675000 -2.49285700

C -1.20460900 3.26415900 -1.84309500

C -1.69484300 2.06520800 -1.25033600

C -3.10771400 1.78572500 -1.36605800

C -3.92402000 2.74226300 -2.03036300

H -4.06185900 4.59007100 -3.08203700

H -1.60288300 5.05455100 -2.94120400

H -0.14166400 3.48040500 -1.80284800

H -4.98701600 2.52193300 -2.09500700

C -0.79544900 1.18685200 -0.55385800

C 0.64328000 1.58104600 -0.22737100

H 0.99694200 2.29945700 -0.97153400

O -1.09853700 0.04385600 -0.10188900

O -3.73982200 0.74581400 -0.90673300

C 1.58780500 0.36100400 -0.27799700

H 1.27361900 -0.22504100 -1.14948900

C 3.01807700 0.78875900 -0.55088000

C 3.49847000 0.76968000 -1.86510100

C 3.87737900 1.20697900 0.47087300

C 4.80218600 1.16226200 -2.15557100

H 2.84013100 0.42710400 -2.66533000

C 5.18087200 1.60526200 0.18235600

H 3.52984200 1.20873600 1.50456200

C 5.64764000 1.58433000 -1.13089600

H 5.16028400 1.13166800 -3.18351300

H 5.83700200 1.92691500 0.98983400

H 6.66911000 1.88793300 -1.35391100

C 1.47793300 -0.52301600 0.94406000

C 1.06368000 0.01954400 2.17689700

C 1.84508100 -1.87790000 0.86276800

C 0.93984300 -0.82788100 3.28782500

C 1.75125400 -2.68906100 1.99870100

C 1.27634600 -2.17161700 3.20008600

H 0.56980200 -0.43379000 4.23196600

H 2.03854700 -3.73759800 1.92782600

H 1.17792600 -2.81571100 4.07198800

C 2.37957700 -2.45839600 -0.39803500

C 3.74135500 -2.76968800 -0.49575400

C 1.55438900 -2.70109600 -1.50433200

C 4.26836700 -3.29946200 -1.67156100

H 4.39244000 -2.57564800 0.35700700

C 2.07976200 -3.23328500 -2.67898400

H 0.48955800 -2.47261900 -1.43923300

C 3.43906700 -3.53040400 -2.76729500

H 5.33087200 -3.53020300 -1.73131600

H 1.42364300 -3.41990800 -3.52772200

H 3.84955800 -3.94278000 -3.68742300

C 0.81475200 1.46871600 2.28799300

C 0.64424100 2.25351600 1.12914700

C 0.81147900 2.11893300 3.53243300

C 0.45683100 3.63197000 1.23705900

C 0.61617000 3.49092100 3.63129000

H 0.99071400 1.54643000 4.44038000

C 0.43751700 4.25582100 2.48031200

H 0.33028600 4.22314200 0.33017900

H 0.61882700 3.96617300 4.61070500

H 0.29550300 5.33264200 2.54840800

Pd -2.88125700 -0.86664500 -0.10925200

O -4.48908000 -2.20247100 0.11255500

C -3.69577300 -3.07854500 0.60654700

O -2.46003000 -2.76545700 0.70677200

C -4.20245800 -4.39309500 1.06788600

H -4.71307400 -4.26649800 2.03036600

H -4.93431100 -4.79490600 0.36068900

H -3.38410700 -5.10481500 1.20153100

**TS_VI-VII_**

C -0.84140900 -5.28826100 -0.58312900

C 0.27542200 -4.90545500 0.17297100

C 0.50677300 -3.55571800 0.41638600

C -0.36605200 -2.55968200 -0.06566100

C -1.54958500 -2.95752500 -0.76401500

C -1.74050000 -4.33288800 -1.03673400

H -1.02383300 -6.33925000 -0.79527900

H 0.95277500 -5.65371300 0.57477100

H 1.35009300 -3.26878500 1.03379100

H -2.62616500 -4.61843700 -1.59794200

C -0.08413300 -1.12225400 0.14938800

C 1.26170200 -0.57902000 0.16488200

H 1.55052200 -0.87807800 1.53346100

O -1.05468000 -0.31365200 0.39676600

O -2.46369700 -2.10367900 -1.21307900

C 1.28693800 0.97473200 0.05204500

C 2.61661900 1.58817600 0.51654800

C 2.81642600 1.82039500 1.88823700

C 3.64221700 1.96026100 -0.36564300

C 4.00027400 2.38780600 2.36227700

H 2.03931900 1.53652100 2.59039800

C 4.83084000 2.52829300 0.10510800

H 3.51653100 1.81967200 -1.43410900

C 5.01719400 2.74388900 1.47141700

H 4.12542000 2.55827300 3.42906900

H 5.60827800 2.80623900 -0.60255300

H 5.93878300 3.18899500 1.83794300

C 0.89294800 1.35435700 -1.37875200

C 1.34646200 0.51766100 -2.43012000

C 0.04242000 2.44339700 -1.67726000

C 0.92407000 0.75718500 -3.74722000

C -0.36098000 2.65554700 -3.00833100

C 0.07140700 1.81990300 -4.03537300

H 1.24746000 0.09770300 -4.54702700

H -1.01704500 3.49216900 -3.23289400

H -0.25505300 1.99675300 -5.05681700

C -0.48714400 3.38380500 -0.64222200

C 0.36315200 4.12413100 0.19629600

C -1.87419600 3.59430800 -0.53356700

C -0.15539400 5.03169500 1.12285700

H 1.43783200 4.00171600 0.11109300

C -2.39351300 4.50034200 0.39355800

H -2.55013600 3.03511900 -1.17570700

C -1.53542600 5.22169900 1.22807100

H 0.52298800 5.59594300 1.75789100

H -3.46916100 4.64274700 0.46181700

H -1.93840100 5.92795700 1.94926300

C 2.28104500 -0.59204200 -2.11122000

C 2.26840500 -1.15256600 -0.80788600

C 3.21437500 -1.05596700 -3.05345800

C 3.22675400 -2.11947800 -0.47987300

C 4.13310500 -2.05191700 -2.72280900

H 3.24272700 -0.61615000 -4.04637900

C 4.14605200 -2.57586900 -1.42900200

H 3.26499400 -2.49986100 0.53665500

H 4.84917300 -2.39599300 -3.46488300

H 4.87972000 -3.32872700 -1.15063400

Pd -3.01051400 -0.70394400 0.09383700

O 1.61996800 -0.91584800 2.76890000

C 2.30910500 -1.87566300 3.32013000

O 2.87756200 -2.79625300 2.71993500

C 2.37338600 -1.77414600 4.83880700

H 2.89383900 -2.63621100 5.26304200

H 2.90166100 -0.85730900 5.12722700

H 1.36411400 -1.71073200 5.26085500

O -5.10962200 -0.71185600 0.00354900

C -5.16037200 0.22474700 0.87701400

O -4.04987000 0.65780900 1.33988600

C -6.46774000 0.81258700 1.30707700

H -7.26502900 0.06793600 1.23768800

H -6.39761900 1.19698400 2.32795500

H -6.71840500 1.64795400 0.64103300

H 0.51709200 1.36102800 0.72133700

**TS'_VI-VII_**

C 1.57766600 4.42493200 -2.45148900

C 0.23497900 4.21629100 -2.11709200

C -0.10479500 3.07628800 -1.40532200

C 0.84973000 2.12360400 -1.00284500

C 2.22405100 2.33561900 -1.35452100

C 2.54406500 3.50356700 -2.09134300

H 1.87122200 5.31523700 -3.00757800

H -0.53344900 4.92183800 -2.42561600

H -1.15266800 2.88434900 -1.18526500

H 3.58968200 3.66102300 -2.35172600

C 0.37679100 0.82777400 -0.46659900

C -0.67696900 0.67879500 0.48659900

H 0.35717300 0.13155700 1.50050400

O 0.94741000 -0.24962300 -0.91868100

O 3.22672000 1.56058000 -1.01250800

C -1.57252000 -0.55232300 0.32013900

C -2.30552800 -1.02728600 1.58011000

C -3.33160900 -1.97691300 1.45046500

C -2.03198200 -0.55556300 2.87003200

C -4.01544700 -2.47345900 2.55654400

H -3.62181000 -2.32177900 0.45865400

C -2.71838100 -1.04392700 3.98113600

H -1.27215100 0.20296100 3.02162200

C -3.70719000 -2.01263000 3.83442600

H -4.79974500 -3.21561800 2.41452600

H -2.47756600 -0.65360100 4.96937500

H -4.24213400 -2.39306900 4.70326100

C -2.60764300 -0.17596100 -0.72627200

C -3.29643100 1.03531000 -0.47086200

C -2.89085900 -0.91878100 -1.88944000

C -4.32606100 1.43888100 -1.32752200

C -3.93056800 -0.47559600 -2.72767500

C -4.65892000 0.67019200 -2.43705400

H -4.84699500 2.37823500 -1.14666100

H -4.17267400 -1.05668800 -3.61670900

H -5.46731400 0.98300300 -3.09585800

C -2.10195100 -2.09457400 -2.34131400

C -1.70194200 -2.14105600 -3.68824500

C -1.73517700 -3.17100400 -1.51807300

C -0.96141800 -3.20635800 -4.18936700

H -1.96250600 -1.31133000 -4.34476500

C -0.99503200 -4.23931500 -2.01862800

H -2.04593000 -3.19238100 -0.47597300

C -0.60174900 -4.26293200 -3.35479800

H -0.65914500 -3.20692800 -5.23531400

H -0.73227400 -5.06378500 -1.35751600

H -0.02121800 -5.09834800 -3.74242200

C -2.82301900 1.91523500 0.61401900

C -1.45937300 1.82804300 0.99445700

C -3.62196800 2.91995200 1.17520900

C -0.93236000 2.78078900 1.87178900

C -3.08755900 3.84527100 2.06558100

H -4.67793900 2.97881000 0.91336900

C -1.73390200 3.78740500 2.40105400

H 0.11992300 2.71357100 2.15395300

H -3.72708600 4.61332500 2.49746800

H -1.30978100 4.51362700 3.09272800

Pd 2.79423100 -0.13499000 -0.00665600

O 2.26651000 -1.79225400 1.20094700

C 1.46635600 -1.56199200 2.13978900

O 0.86515200 -0.43472500 2.36257600

C 1.16432900 -2.65700900 3.11137000

H 0.24665300 -3.16824000 2.78983600

H 1.96863500 -3.39523500 3.14778000

H 0.97388500 -2.25108500 4.10911700

O 4.57960000 0.00445600 1.00230900

C 5.46043300 -0.82760500 0.53293500

O 5.30101400 -1.56864600 -0.44252700

C 6.74808100 -0.85190700 1.32844000

H 6.68119000 -1.63420600 2.09539200

H 7.59545800 -1.09820400 0.68108300

H 6.94030700 0.09590200 1.84041000

H -0.94438700 -1.37222800 -0.04824400

**VII**

C -1.31862600 5.53078900 -0.09895900

C -0.41087200 5.29129200 -1.13047400

C 0.00603400 3.98170900 -1.37778500

C -0.45752400 2.91331500 -0.61166400

C -1.46077100 3.14470000 0.37544700

C -1.84526800 4.47552600 0.63795400

H -1.64110000 6.54909200 0.11891300

H -0.02992600 6.11108100 -1.73686200

H 0.70600600 3.78612900 -2.19041900

H -2.58144000 4.65302600 1.42143800

C -0.07576300 1.48903300 -0.80719400

C 1.10321400 0.92132000 -0.38016000

O -1.03248500 0.72746800 -1.32488500

O -2.00645300 2.14948100 1.05703500

C 1.24420000 -0.58373300 -0.38358000

C 2.34190400 -1.07698400 -1.32661900

C 2.51057300 -0.45918600 -2.57286600

C 3.16823600 -2.16118000 -1.01335600

C 3.46617400 -0.91272600 -3.47765700

H 1.88251500 0.39595000 -2.82852200

C 4.12723700 -2.61812700 -1.91681900

H 3.06812400 -2.65805400 -0.04879500

C 4.28103000 -1.99716900 -3.15321100

H 3.57787900 -0.41395400 -4.43970300

H 4.75824500 -3.46445600 -1.64807500

H 5.03077100 -2.35241500 -3.85843000

C 1.44753300 -1.05058600 1.04436100

C 2.27994900 -0.26652900 1.87493100

C 0.91988300 -2.26461200 1.52825800

C 2.53757100 -0.68743500 3.18705100

C 1.22923600 -2.67269600 2.83418500

C 2.02187500 -1.88559800 3.66178100

H 3.13802300 -0.06454700 3.84739700

H 0.82148500 -3.61388400 3.20199800

H 2.23040900 -2.20345900 4.68199300

C 0.09876200 -3.18234700 0.69819000

C 0.51802300 -4.51082100 0.53215700

C -1.11262500 -2.79143600 0.10929300

C -0.23631300 -5.41444700 -0.21199000

H 1.45647000 -4.83105300 0.98567100

C -1.86962100 -3.69558200 -0.63175600

H -1.47863800 -1.76917400 0.24170100

C -1.43262000 -5.00858200 -0.79954200

H 0.11449300 -6.43825100 -0.33393200

H -2.80667700 -3.36277400 -1.07435800

H -2.02400300 -5.71252700 -1.38302500

C 2.86133200 0.97620100 1.34169800

C 2.24464600 1.60514400 0.22370100

C 3.98839900 1.57002700 1.92454100

C 2.80684100 2.79423700 -0.26419300

C 4.51254700 2.76284300 1.43815300

H 4.47949100 1.08166500 2.76509000

C 3.91705800 3.37591200 0.33726700

H 2.38768300 3.24799300 -1.15708800

H 5.39457700 3.19912200 1.90417700

H 4.33424900 4.29457700 -0.07281200

Pd -2.65342000 0.59073300 -0.09584000

O -4.46558700 -0.04746600 0.89108000

C -4.64049400 -0.92592000 -0.01580000

O -3.79774300 -1.00155700 -0.97304400

C -5.81748700 -1.83877400 0.01684500

H -6.52338900 -1.55272400 -0.77234500

H -5.51112800 -2.87062000 -0.18502400

H -6.33305000 -1.78919700 0.97910300

H 0.29876500 -0.98414400 -0.76406900

**VIII**

C 5.74657800 -0.66270000 -1.62324500

C 4.98119500 -1.47765000 -2.47149300

C 3.59217600 -1.41041300 -2.39065400

C 2.94801400 -0.55791300 -1.47927300

C 3.72244100 0.30673500 -0.65359200

C 5.13440100 0.20823300 -0.72852700

H 6.83313300 -0.70202500 -1.66789300

H 5.46173700 -2.15268400 -3.17421400

H 2.97414300 -2.03989700 -3.02528200

H 5.72798300 0.85578400 -0.08750200

C 1.44980000 -0.62233100 -1.39313400

C 0.74871400 -0.40230900 -0.07999600

O 0.80384500 -0.84704700 -2.42699800

O 3.18180500 1.20198300 0.15319800

C -0.79069100 -0.58232200 -0.16161300

C -1.23808300 -2.03817800 -0.40886600

C -1.67261200 -2.42224400 -1.68784800

C -1.26868200 -3.00370000 0.61115100

C -2.10682600 -3.72415000 -1.94540600

H -1.66049200 -1.69145900 -2.48896500

C -1.69533400 -4.31024400 0.35531500

H -0.96867100 -2.74143600 1.62048400

C -2.11554900 -4.67833000 -0.92458400

H -2.44054000 -3.99148400 -2.94537600

H -1.70541100 -5.03808700 1.16326400

H -2.45157900 -5.69311600 -1.12257000

C -1.46406100 0.00334700 1.07771600

C -0.75252700 0.02665900 2.29991300

C -2.79195500 0.48540700 1.03540600

C -1.33275300 0.63361900 3.42835500

C -3.35759600 1.04714300 2.19047100

C -2.62344300 1.14908600 3.37222800

H -0.77216900 0.70352200 4.35543900

H -4.37570200 1.42522700 2.14994400

H -3.06284300 1.61637800 4.24963900

C -3.66210700 0.38631300 -0.18106100

C -4.79075300 -0.45033800 -0.15127300

C -3.41933400 1.14328200 -1.33998800

C -5.64820000 -0.53806200 -1.25065700

H -4.99218200 -1.04114000 0.73886100

C -4.27784500 1.05526400 -2.43864900

H -2.56308100 1.81064000 -1.36586800

C -5.39311500 0.21370000 -2.40018900

H -6.51338200 -1.19505200 -1.20770800

H -4.07741400 1.65217600 -3.32524900

H -6.05913700 0.14718600 -3.25671800

C 0.54652300 -0.68729300 2.38924400

C 1.28096900 -0.97095800 1.20427500

C 1.00439100 -1.18678400 3.61937400

C 2.40377500 -1.80949400 1.30556100

C 2.15765900 -1.96634100 3.70434200

H 0.43239200 -0.99727700 4.52219300

C 2.84797500 -2.29312100 2.53696000

H 2.94193700 -2.09632600 0.41101600

H 2.48953600 -2.34195800 4.66866200

H 3.72300100 -2.93706900 2.57678500

Pd 1.21879000 1.65652200 -0.08223900

O -0.60487800 2.66897900 -0.45727400

C -0.06323300 3.83247600 -0.39084700

O 1.17729400 3.92937300 -0.15503500

C -0.92203600 5.05889300 -0.55493400

H -1.78692400 4.85159500 -1.19065700

H -1.28984500 5.37118500 0.43091500

H -0.33816900 5.88254600 -0.97448800

H -1.12244800 -0.01524400 -1.02974500

**TS_VIII-IX_**

C 5.29675700 -1.95692300 1.25844600

C 4.80315200 -1.49501100 2.49713000

C 3.64286600 -0.74555000 2.50673500

C 2.91833000 -0.43414800 1.32699200

C 3.40750000 -0.92140900 0.05765100

C 4.62659700 -1.67433100 0.08528700

H 6.21221900 -2.54451900 1.22435900

H 5.32733500 -1.72036600 3.42160100

H 3.24853900 -0.35788100 3.44122800

H 5.00269000 -2.03159300 -0.87040500

C 1.75140700 0.45690200 1.51903300

C 0.85716100 0.94031900 0.36089800

O 1.49596200 0.94073400 2.62462000

O 2.85788200 -0.71330500 -1.10423100

C -0.56966400 0.61049700 0.35700600

C -1.03458200 -0.31305300 1.47344900

C -0.89474500 -1.70518000 1.42126900

C -1.54646200 0.27346000 2.64243900

C -1.26789300 -2.49538300 2.51412800

H -0.50358600 -2.17676400 0.52135700

C -1.91573800 -0.51569200 3.73082800

H -1.64842700 1.35247400 2.70418200

C -1.77928000 -1.90633100 3.67104100

H -1.16087200 -3.57568200 2.45306100

H -2.30703000 -0.04302900 4.62820600

H -2.06957500 -2.52203000 4.51850300

C -1.55657200 1.66834700 -0.07208400

C -1.09217700 2.97691600 -0.36824700

C -2.95556300 1.39823900 -0.14187400

C -2.03406600 4.01199000 -0.55388000

C -3.84911800 2.45450200 -0.33602000

C -3.39424700 3.76328200 -0.50887300

H -1.69844300 5.02754200 -0.72473600

H -4.91335400 2.24081400 -0.37382900

H -4.10305700 4.57612900 -0.64037700

C -3.56837600 0.02953300 -0.11111500

C -4.45363400 -0.34189900 0.91349700

C -3.37989500 -0.85443600 -1.18662300

C -5.10075100 -1.57885200 0.88483000

H -4.62727400 0.33885000 1.74202300

C -4.03021400 -2.09049900 -1.21788800

H -2.73781600 -0.56568300 -2.01419300

C -4.88701900 -2.46053100 -0.17838900

H -5.77517500 -1.85160500 1.69257200

H -3.87373500 -2.75805900 -2.06144400

H -5.39251400 -3.42234400 -0.20186200

C 0.34931400 3.24726300 -0.51282800

C 1.29710600 2.24989700 -0.17657900

C 0.82401900 4.48148200 -1.01033100

C 2.67386700 2.53481600 -0.31069800

C 2.18067300 4.73889800 -1.14728100

H 0.12552400 5.25320100 -1.31154600

C 3.11570400 3.76026600 -0.78663100

H 3.39999200 1.77527000 -0.04604300

H 2.51080100 5.69853900 -1.53558300

H 4.18069500 3.95231300 -0.88525000

Pd 0.76093300 -0.45676900 -1.16488900

O 0.57222500 -1.75693600 -2.83170000

C 0.37118900 -3.00714800 -2.57176000

O 0.22692400 -3.50682100 -1.44350200

C 0.29161100 -3.89215800 -3.81738400

H -0.60539200 -3.63877000 -4.39656300

H 1.15522900 -3.71982100 -4.46948900

H 0.24789900 -4.95012000 -3.54532100

H -0.80505600 -0.28333100 -0.92010600

**IX**

C -3.48318900 -3.81303000 -1.24920800

C -3.50536100 -3.11107600 -2.46938400

C -3.01607900 -1.82242300 -2.49620600

C -2.48613400 -1.18501200 -1.35068800

C -2.46571600 -1.89282300 -0.10055400

C -2.98352600 -3.22504100 -0.10862800

H -3.86029600 -4.83526000 -1.20474500

H -3.89350400 -3.57886500 -3.37207500

H -3.00918500 -1.25131300 -3.42357800

H -2.96286100 -3.76779900 0.83594100

C -1.98507700 0.16978500 -1.55295500

C -1.07495700 0.94106000 -0.60117400

O -2.21742300 0.79520900 -2.59598700

O -2.04180400 -1.42028300 1.02190400

C 0.30466100 0.56272300 -0.53916600

C 0.66914600 -0.71163600 -1.25351100

C 0.64663800 -1.97485900 -0.65757700

C 0.96676100 -0.61407200 -2.61943400

C 0.91177700 -3.11534100 -1.41183600

H 0.41854300 -2.05718700 0.40625700

C 1.22771500 -1.75478300 -3.37421700

H 0.97891600 0.36710600 -3.09515500

C 1.20183000 -3.01108800 -2.77102600

H 0.89735600 -4.09231000 -0.92987100

H 1.44960000 -1.66059200 -4.43625300

H 1.40636800 -3.90532900 -3.35750000

C 1.33846500 1.62194500 -0.45607200

C 0.93420400 2.98865800 -0.50060100

C 2.73101500 1.32210700 -0.42875800

C 1.90858500 3.98900500 -0.66166300

C 3.65608100 2.35321700 -0.59953500

C 3.25164200 3.67719800 -0.74297100

H 1.60581500 5.02915400 -0.74513400

H 4.71736700 2.10790500 -0.58122400

H 3.99038600 4.46415600 -0.88292200

C 3.30140500 -0.01325200 -0.13228000

C 4.31262200 -0.53860800 -0.94889600

C 2.92829700 -0.72903800 1.01315300

C 4.92396200 -1.74935000 -0.64003200

H 4.60746900 0.00657200 -1.84614800

C 3.55171200 -1.93286700 1.33112400

H 2.12811200 -0.35813600 1.65597600

C 4.54962500 -2.44959800 0.50635400

H 5.69705600 -2.14668000 -1.29620400

H 3.24228800 -2.46856700 2.22736100

H 5.03148300 -3.39469800 0.75248000

C -0.47454600 3.34826900 -0.36012500

C -1.46125900 2.33820400 -0.39937800

C -0.89825400 4.67795700 -0.15747800

C -2.82062400 2.67914700 -0.27389100

C -2.23814300 4.99619500 -0.01956600

H -0.16525800 5.47749500 -0.08355500

C -3.21092500 3.99137800 -0.08403600

H -3.56743700 1.88655700 -0.31806600

H -2.53165800 6.03158700 0.14413600

H -4.26560200 4.23913000 0.02199500

Pd -0.71066900 0.20608400 1.38501600

O -1.05086200 -0.17550000 3.43517500

C -0.34470500 -1.19362100 3.80663400

O 0.51005700 -1.76474600 3.11542900

C -0.66104800 -1.66793900 5.21024700

H -0.57502400 -0.84307500 5.92704000

H -1.69661000 -2.02484700 5.26507400

H 0.00627100 -2.47827500 5.51740700

H 0.20187900 1.38761700 1.85587600

**TS_IX-II_**

C -2.22681900 4.58646400 -0.91009200

C -1.62377500 5.10541600 0.24346500

C -0.79603800 4.28856300 0.99139500

C -0.54609500 2.95271600 0.62766100

C -1.14714300 2.44303500 -0.55196100

C -1.99491400 3.27815500 -1.30133700

H -2.88283800 5.21397500 -1.51181400

H -1.81172800 6.13251500 0.54873700

H -0.32074000 4.65152400 1.90128700

H -2.45877200 2.87390600 -2.20130900

C 0.29744300 2.14552700 1.53265000

C 0.32197100 0.63896000 1.41959600

O 0.92124100 2.66541700 2.45647300

O -0.83245000 1.20202100 -0.95922200

C 1.43805000 -0.02241000 0.95781700

C 2.61292100 0.79453300 0.56191400

C 2.49303400 1.82357800 -0.38090400

C 3.85855900 0.58907200 1.16879600

C 3.58356700 2.62320800 -0.70988200

H 1.53948600 1.96780600 -0.88821200

C 4.95130400 1.38492700 0.83817900

H 3.97067600 -0.20795800 1.90418400

C 4.81887800 2.40611500 -0.10195500

H 3.47038100 3.41010700 -1.45413600

H 5.91174000 1.20786900 1.32037100

H 5.67521700 3.02644200 -0.36107900

C 1.47389700 -1.47274400 0.96623700

C 0.46949800 -2.17765100 1.70617700

C 2.47941600 -2.24480000 0.29305400

C 0.61112500 -3.56507400 1.91416800

C 2.58261000 -3.60720900 0.55452400

C 1.67670300 -4.26350700 1.39005600

H -0.12952900 -4.10434800 2.49724600

H 3.35363800 -4.17911600 0.03952300

H 1.77716300 -5.33144800 1.57427200

C 3.34583000 -1.73049800 -0.79283500

C 4.71482400 -2.03091900 -0.82182800

C 2.79656700 -1.02332500 -1.87155500

C 5.51480200 -1.61925100 -1.88346600

H 5.15780700 -2.57323300 0.01389400

C 3.59472700 -0.61466800 -2.93539600

H 1.73026100 -0.79292600 -1.87043000

C 4.95814400 -0.90647700 -2.94445100

H 6.57871000 -1.85156200 -1.87898300

H 3.14727400 -0.06541000 -3.76272300

H 5.58213300 -0.58327800 -3.77606400

C -0.68482400 -1.46480400 2.21925600

C -0.78668800 -0.06170600 2.00470800

C -1.73364900 -2.11033200 2.91065000

C -1.93707700 0.62422300 2.46393900

C -2.84121100 -1.41747100 3.35748400

H -1.68660000 -3.17937400 3.10033400

C -2.95000100 -0.03858500 3.12510600

H -2.02108200 1.69821000 2.30606600

H -3.63273300 -1.94711600 3.88433000

H -3.82473800 0.50988500 3.46964000

Pd -2.54689100 -0.17510300 -1.34538300

O -4.01542700 -1.26845300 -2.45918000

C -4.62477100 -1.89083900 -1.51428500

O -4.32565100 -1.78083800 -0.30690100

C -5.75721100 -2.79097100 -1.95355300

H -5.39532200 -3.53605800 -2.67195000

H -6.21407700 -3.30830400 -1.10506500

H -6.52998000 -2.20837200 -2.46930100

H -1.47298900 0.89832300 -2.01055900

**X**

C 3.45010400 -0.51911500 3.52865800

C 2.47631200 -0.01770400 4.40925400

C 1.38241900 0.63671100 3.88170500

C 1.20076700 0.80346400 2.49120100

C 2.19261500 0.29777800 1.59385200

C 3.31646500 -0.36227600 2.16408900

H 4.31982400 -1.04472800 3.92404200

H 2.57975600 -0.14978000 5.48433700

H 0.60574000 1.03390300 4.53373800

H 4.05931300 -0.76325300 1.47486000

C -0.01934800 1.50132600 2.08760500

C -0.67266300 1.43970800 0.71101100

O -0.67896500 2.15375100 2.90395600

O 2.14305500 0.42838800 0.29797600

C -1.33655100 0.22821900 0.33662800

C -1.13050700 -0.95886900 1.23933500

C -0.07802000 -1.86866100 1.10679300

C -2.02499400 -1.10850300 2.30689700

C 0.07921600 -2.90235300 2.02723000

H 0.61567200 -1.76080100 0.27043700

C -1.86431900 -2.13846800 3.22966500

H -2.84873000 -0.40263400 2.41852000

C -0.81159600 -3.04094300 3.09068200

H 0.90114400 -3.60719800 1.90689900

H -2.56558200 -2.23621600 4.05698100

H -0.68481600 -3.84904800 3.80933400

C -2.62785000 0.30858900 -0.38542700

C -3.21270700 1.58713600 -0.61394700

C -3.35803600 -0.85501200 -0.76185700

C -4.54856200 1.66550400 -1.04527100

C -4.68406200 -0.72301200 -1.17764700

C -5.28996500 0.52558900 -1.28987600

H -5.01933800 2.63712300 -1.16939600

H -5.23613000 -1.62073300 -1.45335200

H -6.32893000 0.60370400 -1.60437500

C -2.77435100 -2.21395700 -0.84249800

C -3.45927900 -3.31625400 -0.31081300

C -1.57686900 -2.44158900 -1.53330400

C -2.95333200 -4.60522300 -0.45228300

H -4.38797200 -3.15203300 0.23681600

C -1.07726800 -3.73116000 -1.68562200

H -1.01266100 -1.60044800 -1.93908400

C -1.76079200 -4.81831600 -1.14372300

H -3.49124500 -5.44673600 -0.01788800

H -0.13754200 -3.87334700 -2.21681500

H -1.36572600 -5.82696800 -1.25543500

C -2.42618100 2.80094500 -0.41958100

C -1.16676800 2.72569700 0.21745900

C -2.86320600 4.06166900 -0.87384300

C -0.40539500 3.89275900 0.40733100

C -2.09694000 5.19953300 -0.69236200

H -3.81043300 4.15207900 -1.39973300

C -0.85858100 5.11872200 -0.04329800

H 0.56032200 3.81527700 0.90718700

H -2.45893100 6.15706300 -1.06205800

H -0.25399500 6.01211300 0.10159700

Pd 0.41077400 0.61612800 -0.96083100

O 1.79154200 0.51340300 -2.53931400

C 2.11006600 -0.72181100 -2.76732400

O 1.56809900 -1.70848700 -2.25104900

C 3.20798700 -0.88304900 -3.79746900

H 2.77247200 -0.82151400 -4.80317600

H 3.95387200 -0.08524300 -3.72095000

H 3.69840800 -1.85593100 -3.70025000

H -0.70379000 0.78564000 -2.04069000

O 4.49273800 0.90802300 -0.92684800

C 5.33287500 -0.12015300 -1.05501600

O 5.08804600 -1.25753100 -0.68668300

C 6.61166600 0.29313600 -1.72008700

H 6.42099000 0.53707500 -2.77224100

H 7.02271000 1.19617700 -1.25646000

H 7.34757800 -0.51234600 -1.67409400

H 3.62218400 0.59616900 -0.53373500

**TS_X-II_**

C 3.33611100 -0.87787700 3.53602900

C 2.34511100 -0.44299900 4.43047000

C 1.28009000 0.28327200 3.93728700

C 1.14429100 0.58515600 2.56525300

C 2.15346000 0.14577600 1.65599200

C 3.24716800 -0.58702200 2.18904900

H 4.18267000 -1.45868200 3.90270600

H 2.41111000 -0.68105700 5.49016800

H 0.49083100 0.63107500 4.60201700

H 4.00087900 -0.94015100 1.48606200

C -0.05811000 1.33875100 2.19803400

C -0.70441300 1.36644000 0.81850600

O -0.70733000 1.94672300 3.05477800

O 2.14242800 0.40213700 0.37285600

C -1.31228300 0.16099800 0.34324900

C -1.11723000 -1.07842700 1.17525300

C -0.06743800 -1.98674900 1.02079500

C -2.05220200 -1.29415600 2.19644100

C 0.03682500 -3.09283600 1.86126100

H 0.66441800 -1.82614100 0.22781100

C -1.94532000 -2.39535300 3.04052300

H -2.87139900 -0.58636400 2.32807500

C -0.90050000 -3.30170200 2.87246300

H 0.85747000 -3.79587200 1.72297900

H -2.68002300 -2.54366600 3.83027000

H -0.81503700 -4.16775500 3.52670000

C -2.59075400 0.24868800 -0.40163100

C -3.23111700 1.51198400 -0.53853200

C -3.25415700 -0.91326400 -0.88641500

C -4.55922600 1.56021800 -0.99808300

C -4.57604600 -0.81589000 -1.32070300

C -5.23890200 0.40907900 -1.34908900

H -5.07478300 2.51514400 -1.05905300

H -5.07623300 -1.71602100 -1.67681100

H -6.27379100 0.46414000 -1.68115400

C -2.58531200 -2.22429800 -1.05580800

C -3.15591700 -3.39812100 -0.54595900

C -1.40222000 -2.32021600 -1.79994400

C -2.54587200 -4.63210200 -0.75519100

H -4.07088700 -3.33423600 0.04381600

C -0.80221400 -3.55571000 -2.02373800

H -0.92885900 -1.41895500 -2.19123800

C -1.36838200 -4.71595400 -1.49821500

H -2.99160600 -5.53259200 -0.33471100

H 0.12132200 -3.59771900 -2.59870500

H -0.89402400 -5.68232200 -1.66240000

C -2.50530000 2.73911000 -0.22342300

C -1.25063800 2.66662800 0.42437000

C -2.99312100 4.01297200 -0.57868200

C -0.54097800 3.84566700 0.71311900

C -2.27791200 5.16394900 -0.29793700

H -3.93864300 4.10555100 -1.10715600

C -1.04190300 5.08343800 0.35518200

H 0.42317200 3.76984500 1.21532000

H -2.67679900 6.13184500 -0.59600100

H -0.47588600 5.98644900 0.57576300

Pd 0.42693300 0.71831400 -0.89611800

O 1.84735600 0.97410300 -2.43251800

C 2.13815500 -0.17580700 -2.94824000

O 1.56168300 -1.24146400 -2.68624700

C 3.23892900 -0.12735200 -3.98757400

H 2.78778400 -0.00334300 -4.98078200

H 3.92351100 0.71212400 -3.83085000

H 3.80366200 -1.06452300 -3.99946600

H -0.67201900 1.03427800 -1.95430200

O 4.41999200 1.02190600 -0.56874000

C 5.18384600 0.00921900 -0.95740300

O 4.87423400 -1.16979400 -0.86550400

C 6.50329800 0.47726700 -1.50329200

H 6.37685300 1.32519600 -2.18451100

H 7.14124900 0.82385400 -0.68071100

H 7.01559700 -0.33858300 -2.01862300

H 3.31475200 0.64049500 -0.15129900

**IV'**

C -4.37057700 -0.79699500 -0.89921700

C -3.04758100 -1.02935600 -0.53472900

C -2.72889300 -1.68958300 0.66618200

C -3.79116300 -2.10007600 1.48439900

C -5.11323800 -1.88886400 1.12478300

C -5.39988000 -1.23152500 -0.07181100

H -4.59106200 -0.27026400 -1.82686700

H -3.53911300 -2.59843200 2.41830900

H -5.91860200 -2.22775900 1.77211900

H -6.43120000 -1.04741800 -0.36580400

O -2.02213600 -0.58887800 -1.34784800

H -2.37883900 0.03611900 -2.00819400

C -1.34360500 -1.93495700 1.15302900

C -0.25712100 -2.00386800 0.14375200

H -0.52483300 -2.33303300 -0.86081800

C 1.06557200 -1.85931600 0.49894800

H 1.26809100 -1.65210900 1.55136700

O -1.12146600 -2.10436700 2.35239000

C 2.23176100 -2.07148200 -0.34002900

C 2.14434800 -2.33816800 -1.71835800

C 3.50211000 -1.98099100 0.25301700

C 3.29515500 -2.51993000 -2.47096400

H 1.16971500 -2.39239600 -2.20203100

C 4.65370700 -2.16546700 -0.50355800

H 3.57117000 -1.75685400 1.31802700

C 4.55264500 -2.43505700 -1.86698700

H 3.21686300 -2.72646300 -3.53668000

H 5.63092200 -2.09315000 -0.03009700

H 5.45253600 -2.57655900 -2.46276500

Pd -0.09883800 0.20875500 -0.19290600

C 1.39148900 0.92954700 0.91787600

C 2.59902600 1.26089600 0.30833400

C 1.21673400 1.05816900 2.29317500

C 3.66010100 1.70594400 1.10200500

H 2.72962900 1.16241600 -0.76956100

C 2.28769700 1.50226400 3.07438700

H 0.26749300 0.80311700 2.76459600

C 3.50769100 1.82380800 2.48208600

H 4.60933700 1.95569100 0.62869500

H 2.15816100 1.59353100 4.15218500

H 4.33860500 2.16687400 3.09593000

I -0.78621400 2.72622600 -0.85620700

**TS_IV'-V'_**

C -4.54748600 -0.52127600 -0.97774100

C -3.22508000 -0.69531800 -0.58238400

C -2.88974700 -1.55118700 0.48286200

C -3.94076500 -2.20446200 1.14346300

C -5.26170600 -2.04882700 0.75158200

C -5.56239500 -1.20216600 -0.31454600

H -4.78100900 0.15831000 -1.79633000

H -3.68121200 -2.85105200 1.97939200

H -6.05440400 -2.57862700 1.27407400

H -6.59330800 -1.05877400 -0.63172000

O -2.22650700 0.00858900 -1.22906700

H -2.61491800 0.71079000 -1.78483600

C -1.50881100 -1.79896400 0.98900000

C -0.38594000 -1.70771400 0.02603600

H -0.59095800 -1.99675800 -1.00950800

C 0.96609100 -1.77170800 0.49705500

H 1.07511200 -2.08766000 1.53434900

O -1.32178200 -2.08968500 2.17264800

C 2.05874100 -2.17640600 -0.39898500

C 2.07834200 -1.81124700 -1.75274100

C 3.11342000 -2.93968200 0.11671500

C 3.11536100 -2.22802800 -2.57825800

H 1.28398500 -1.17275400 -2.14561700

C 4.14839600 -3.36100100 -0.71239800

H 3.10952500 -3.21085600 1.17204500

C 4.15120900 -3.00721000 -2.06047900

H 3.12340300 -1.93530500 -3.62632900

H 4.95757900 -3.96338400 -0.30373500

H 4.96649400 -3.32762200 -2.70665400

Pd -0.21472400 0.32268800 -0.19256900

C 1.41342000 0.15861800 1.07127400

C 2.66249100 0.59214200 0.60719000

C 1.02669800 0.39647900 2.39971400

C 3.49681300 1.30910900 1.45811200

H 2.97420100 0.37885300 -0.41482400

C 1.87062500 1.11656700 3.24143500

H 0.07576500 0.01464200 2.77008200

C 3.10160800 1.57469700 2.77120600

H 4.46049100 1.66189000 1.09480700

H 1.56910600 1.31144500 4.26918300

H 3.76265900 2.13000000 3.43399200

I -0.07672900 3.07334900 -0.73334900

**V'**

C 1.41139900 5.29796500 -0.71297000

C 0.13938600 5.52308500 -0.18732300

C -0.57322800 4.46653100 0.36460700

C -0.04771100 3.16880500 0.38257800

C 1.24785600 2.98318900 -0.11347800

C 1.97722400 4.02810600 -0.66904700

H 1.97930500 6.11742800 -1.14814000

H -0.29595400 6.51933900 -0.21277700

H -1.57107100 4.62202500 0.77101600

H 2.98535500 3.85400300 -1.04274200

C -0.89655100 2.06284900 0.91189300

C -1.07387400 0.86294500 0.05204500

H -1.20351100 1.11706800 -1.00833400

O -1.49474300 2.19156400 1.97837900

O 1.82625900 1.73827300 0.06214800

H 2.70610600 1.69651900 -0.36276800

C -1.93463400 -0.25007200 0.59819400

H -1.97799000 -0.12382500 1.68685900

C -3.35085900 -0.38628300 0.08529700

C -4.33438000 -0.86999300 0.95564400

C -3.70842700 -0.10027500 -1.23682200

C -5.64155300 -1.06532000 0.52059400

H -4.06474300 -1.09576700 1.98808400

C -5.01774300 -0.29229300 -1.67396000

H -2.96385800 0.27813700 -1.93723200

C -5.98783300 -0.77560200 -0.79851100

H -6.39205000 -1.44199000 1.21373300

H -5.27945800 -0.06078800 -2.70523500

H -7.01029000 -0.92318600 -1.14228500

Pd 0.70406600 -0.16886000 -0.02899800

I 3.23857900 -1.48510400 -0.04065400

C -1.07185800 -1.48200300 0.32330400

C -0.75679900 -2.40989800 1.35057700

C -0.69635400 -1.79619700 -1.01528100

C -0.16017400 -3.61656800 1.04391400

H -1.03304900 -2.16700600 2.37602700

C -0.08529400 -3.03091100 -1.30435100

H -1.03402700 -1.16387700 -1.83600900

C 0.16543500 -3.93341500 -0.28759000

H 0.05127700 -4.33206200 1.83608700

H 0.17123500 -3.27091000 -2.33380100

H 0.62468700 -4.89361200 -0.51397500

**VII'c**

C 5.40599700 -0.27138600 0.56901200

C 4.96083500 0.96498100 1.03730100

C 3.59864500 1.21197300 1.13636600

C 2.65104800 0.25145200 0.75702600

C 3.13273600 -0.98835000 0.32915800

C 4.49172200 -1.25906600 0.22057200

H 6.47071300 -0.48005000 0.48828200

H 5.67630100 1.73436300 1.31842000

H 3.23124800 2.17394500 1.48859900

H 4.82960400 -2.24100100 -0.10840100

C 1.20746600 0.63785800 0.80336700

C 0.34446600 0.24342200 -0.34012900

H 0.91100800 0.33925800 -1.28131400

O 0.81244000 1.35568000 1.72291100

O 2.21340400 -2.00164900 0.10598600

H 2.64890900 -2.76445900 -0.32171000

C -1.00336700 0.95402900 -0.47841800

H -1.48620100 0.46749200 -1.34088800

C -1.98325600 0.81556200 0.67350100

C -3.14263600 1.60277300 0.67878000

C -1.83370800 -0.12590700 1.69742700

C -4.11282200 1.46012300 1.66822500

H -3.28725700 2.34290700 -0.10870100

C -2.80204600 -0.27660000 2.68728300

H -0.94570100 -0.75795800 1.72813100

C -3.94716000 0.51721900 2.68028300

H -5.00197600 2.08870900 1.64502400

H -2.65598400 -1.01742300 3.47243900

H -4.70243100 0.40254600 3.45616600

Pd 0.13334100 -1.80264300 -0.41984100

C -0.74274700 2.39175100 -0.91418000

C -0.57178400 2.66827700 -2.27559000

C -0.61312800 3.44381700 0.00016000

C -0.27755200 3.95642600 -2.71656000

H -0.67610200 1.85774400 -2.99875700

C -0.32226600 4.73341200 -0.43827600

H -0.74108200 3.24562500 1.06237100

C -0.15011300 4.99534600 -1.79691900

H -0.15358100 4.14867100 -3.78151100

H -0.22812700 5.53949300 0.28831700

H 0.07810200 6.00455600 -2.13620400

C -1.81452000 -3.25087800 -1.07577300

O -0.72385200 -3.83771500 -0.81725000

O -1.86865200 -1.96586600 -1.00364800

C -3.05069100 -4.00471800 -1.42520700

H -3.70150700 -3.41573000 -2.07742900

H -2.80685600 -4.95803500 -1.90206000

H -3.61019700 -4.22175200 -0.50655300

**TS_VI'-VII'a_**

C -3.54022000 3.85195000 0.62338900

C -2.43458700 4.64099600 0.29899700

C -1.29062800 4.03775000 -0.20797600

C -1.21852500 2.65000700 -0.37526700

C -2.36021500 1.87446000 -0.08922500

C -3.51264700 2.47672500 0.42573900

H -4.44225000 4.31279400 1.02267100

H -2.46548100 5.71814300 0.44797800

H -0.41455200 4.63385700 -0.45978400

H -4.38787900 1.86712100 0.64326600

C 0.05814100 2.04590900 -0.84734200

C 0.60253300 0.90488500 -0.06057500

H 0.62236500 1.13724100 1.01467600

O 0.67775100 2.53033500 -1.79408700

O -2.35707400 0.56271100 -0.42742400

H -3.16903600 -0.12456100 -0.03809100

C 1.83393600 0.20823900 -0.60050400

H 1.86908500 0.39264800 -1.68142600

C 3.18112200 0.57579400 -0.02082200

C 4.30359900 0.58202700 -0.85681500

C 3.35983600 0.85473300 1.33879100

C 5.57179200 0.85517500 -0.35149200

H 4.17462700 0.36709400 -1.91864800

C 4.62764700 1.12964400 1.84716000

H 2.50375200 0.86024100 2.01358200

C 5.73783300 1.12908700 1.00510800

H 6.43188600 0.85664700 -1.01924900

H 4.74707700 1.34623500 2.90774800

H 6.72776300 1.34417000 1.40398600

Pd -0.68966800 -0.67226800 -0.03254300

C 1.46744700 -1.26251800 -0.40385800

C 1.54437900 -2.19292300 -1.47005700

C 1.12030700 -1.74071400 0.89326800

C 1.32596300 -3.53733800 -1.24431400

H 1.81091600 -1.83046400 -2.46252000

C 0.90705700 -3.12213300 1.09995500

H 1.21674700 -1.08357100 1.75740400

C 1.00636100 -4.00544500 0.04495900

H 1.41141000 -4.24429400 -2.06760400

H 0.66958200 -3.47793800 2.10056200

H 0.84563500 -5.06958400 0.20656900

C -3.33608900 -2.20470700 0.28165500

O -3.94514400 -1.08143700 0.29325200

O -2.09371600 -2.36581800 0.11404900

C -4.19053200 -3.42696600 0.45327200

H -3.59587100 -4.30313100 0.72167100

H -4.96645300 -3.25538000 1.20568100

H -4.70618900 -3.63710500 -0.49209400

**TS_V'-VII'b_**

C -4.77148600 -2.87251900 -0.87123300

C -3.92417900 -3.91387000 -0.49452900

C -2.62108400 -3.62761800 -0.11837400

C -2.11515000 -2.31787900 -0.12432700

C -3.00441400 -1.28735500 -0.47785700

C -4.31670100 -1.55992800 -0.85409400

H -5.79828000 -3.07562500 -1.16785500

H -4.27787100 -4.94198400 -0.49932200

H -1.94148400 -4.42608100 0.17219200

H -4.98375700 -0.73876000 -1.11351200

C -0.67238600 -2.16526000 0.23860800

C 0.12965300 -1.19033700 -0.55521200

H -0.05274200 -1.26389700 -1.63414500

O -0.16513500 -2.91541800 1.06565100

O -2.59800600 0.02620400 -0.37080600

H -3.31030000 0.63672400 -0.64163800

C 1.39357700 -0.60415900 -0.16507800

H 1.08676000 0.89149300 -0.02100100

C 1.94442900 -0.83185600 1.21867200

C 1.29788300 -0.35024100 2.36099700

C 3.12248300 -1.57208600 1.36854600

C 1.80009000 -0.62219200 3.62848200

H 0.39224300 0.25056200 2.24389000

C 3.62048700 -1.85036800 2.63854400

H 3.64305700 -1.94773000 0.48862700

C 2.96323500 -1.37694800 3.77179100

H 1.28667900 -0.23355500 4.50620700

H 4.53299600 -2.43524200 2.73917500

H 3.36244100 -1.58413300 4.76305600

Pd -0.49488600 0.71954900 -0.16005700

I -1.15226800 3.34705100 0.27494000

C 2.42549600 -0.42005000 -1.24358600

C 3.33845100 0.63959400 -1.17395600

C 2.54632900 -1.35459500 -2.27677200

C 4.33740300 0.77590800 -2.12954200

H 3.25314400 1.36792900 -0.36629900

C 3.55407400 -1.22117700 -3.23039000

H 1.86627200 -2.20417600 -2.32334500

C 4.44757800 -0.15595600 -3.16232600

H 5.03269800 1.61102100 -2.06947800

H 3.64209400 -1.96022400 -4.02457400

H 5.23149100 -0.05220500 -3.91014500

**VII'a**

C -3.63109600 3.91657400 0.46689500

C -2.56810800 4.70387300 0.00677000

C -1.40575600 4.07926700 -0.41468300

C -1.25993000 2.68312200 -0.37441800

C -2.35975100 1.88502100 0.04163600

C -3.53059200 2.53561600 0.48914400

H -4.55296300 4.38884500 0.80495200

H -2.65016300 5.78857300 -0.00986500

H -0.55822700 4.66458600 -0.76937500

H -4.36990400 1.92205500 0.81508900

C 0.04562600 2.12096900 -0.78041600

C 0.58421400 0.95281900 -0.02246900

H 0.61145000 1.16442300 1.05741000

O 0.72764300 2.66085000 -1.65644600

O -2.37106300 0.56552200 -0.06870200

H -3.42389000 -0.56258600 0.41681400

C 1.82263300 0.28735500 -0.58814700

H 1.87882200 0.54852900 -1.65200800

C 3.15899600 0.60959700 0.04290500

C 4.30318700 0.62770900 -0.76327900

C 3.30734300 0.83234500 1.41622200

C 5.56216300 0.85867500 -0.21577300

H 4.19849800 0.45573100 -1.83550600

C 4.56608900 1.06514800 1.96714200

H 2.43412300 0.82585900 2.06833400

C 5.69755500 1.07798500 1.15419600

H 6.43925700 0.87020700 -0.86093100

H 4.66124500 1.23848500 3.03805800

H 6.68006800 1.26020600 1.58629900

Pd -0.67989300 -0.62675800 -0.03608000

C 1.46673000 -1.19870700 -0.50291500

C 1.49227000 -2.03233800 -1.64799300

C 1.19516500 -1.79126700 0.76081900

C 1.29990600 -3.39599500 -1.52998600

H 1.69695800 -1.58083100 -2.61860200

C 1.00520200 -3.18471600 0.86039600

H 1.30853800 -1.20170100 1.67079800

C 1.05947100 -3.97560100 -0.27161300

H 1.34287500 -4.02621500 -2.41624000

H 0.82873000 -3.62961600 1.83772500

H 0.92114900 -5.05214700 -0.19136300

C -3.23572400 -2.46346200 0.35432000

O -3.96263100 -1.39476400 0.61037700

O -2.04837400 -2.42685300 0.00619000

C -3.97580800 -3.74370700 0.52744100

H -3.32760900 -4.59778600 0.32680400

H -4.36750600 -3.81223900 1.54826200

H -4.83967200 -3.77324800 -0.14547800

**VII'b**

C -4.91940000 -2.26203100 -1.00314900

C -4.23996500 -3.41968400 -0.62206400

C -2.86959800 -3.36812700 -0.43148500

C -2.13143000 -2.18765300 -0.61953900

C -2.84579500 -1.03687900 -1.00073400

C -4.22552100 -1.07337500 -1.18927700

H -5.99671200 -2.27798900 -1.15526700

H -4.77940500 -4.35244700 -0.47502100

H -2.31384700 -4.25341900 -0.13139500

H -4.75423600 -0.16557500 -1.47719300

C -0.66288600 -2.27193000 -0.38097200

C 0.21731300 -1.17925600 -0.88181700

H -0.01765300 -0.85623000 -1.89983100

O -0.18643700 -3.27692000 0.14134800

O -2.17176500 0.14916800 -1.19320700

H -2.81345100 0.87511600 -1.31420900

C 1.47722800 -0.84283300 -0.38067100

H 0.88351100 1.24603800 0.87344600

C 1.97933300 -1.35717600 0.92172700

C 1.17919900 -1.39178700 2.07382800

C 3.28309900 -1.86598000 1.00071900

C 1.66331900 -1.91802100 3.26291500

H 0.16891200 -0.97935600 2.02770300

C 3.76488800 -2.40615900 2.19125500

H 3.91972700 -1.85575400 0.11771500

C 2.96036600 -2.43060200 3.32645100

H 1.02954800 -1.92242700 4.14773300

H 4.77833700 -2.80154400 2.22857400

H 3.34244100 -2.83794600 4.26068100

Pd -0.29878300 0.76270800 0.02512000

I -1.29929500 3.04888200 0.92833200

C 2.44352200 -0.13403800 -1.25670800

C 3.38716600 0.75075800 -0.70874700

C 2.45834300 -0.35577800 -2.64136400

C 4.30323000 1.40486400 -1.52193300

H 3.38373900 0.93541600 0.36554100

C 3.38164600 0.29512800 -3.45371700

H 1.76245400 -1.06663200 -3.08370900

C 4.30401100 1.17970000 -2.89902700

H 5.01881100 2.09612100 -1.08085000

H 3.38580100 0.10042500 -4.52464900

H 5.02585700 1.68807500 -3.53564800

**VIII'a**

C -5.33783700 -1.05646400 -0.88105700

C -5.14312100 -1.99444500 0.14260900

C -3.90404800 -2.05035100 0.76491500

C -2.85133500 -1.20188600 0.39512300

C -3.07097900 -0.18794200 -0.58704800

C -4.32693400 -0.18534400 -1.24982200

H -6.30047000 -1.00057700 -1.39036300

H -5.94188700 -2.67522100 0.43104200

H -3.70819800 -2.78237900 1.54906500

H -4.49283400 0.56715400 -2.02016500

C -1.52029200 -1.42874700 1.00547000

C -0.35542400 -1.22189000 0.11891300

H -0.52523700 -1.57214600 -0.90747800

O -1.40542900 -1.83272100 2.17280400

O -2.23179400 0.78257200 -0.84959800

C 1.00215600 -1.52728000 0.72033500

H 0.87570700 -2.18897400 1.58903800

C 2.01141400 -2.14046100 -0.21866600

C 2.98721900 -3.00525100 0.29059600

C 2.03654900 -1.84558800 -1.58791200

C 3.95749500 -3.56228400 -0.53846400

H 2.97847400 -3.24525800 1.35453300

C 3.00679300 -2.39973400 -2.42058300

H 1.29212900 -1.16807400 -2.00922200

C 3.97050600 -3.26065700 -1.89934400

H 4.70223200 -4.23867300 -0.12128700

H 3.00808500 -2.15658400 -3.48207500

H 4.72589400 -3.69664300 -2.55112900

Pd -0.31236900 0.82138900 -0.07176700

C 1.43958800 -0.15438300 1.24977000

C 1.27451400 0.17030100 2.61585000

C 2.01373600 0.81277200 0.39054300

C 1.70854800 1.38757600 3.10917400

H 0.81943700 -0.56785200 3.27488000

C 2.43615800 2.05424100 0.90928900

H 2.26572000 0.55650900 -0.63681800

C 2.29289600 2.33745600 2.25349200

H 1.60026400 1.60932900 4.16994900

H 2.87570400 2.78237100 0.22957500

H 2.63216000 3.29203400 2.65198000

C 0.46697800 3.36117300 -1.20394300

O -0.28433300 2.99052500 -0.22870900

O 1.04444900 2.60335900 -2.00450200

C 0.65136500 4.86499300 -1.31129500

H -0.31015200 5.38788300 -1.24862200

H 1.25773900 5.22190700 -0.46794200

H 1.15539000 5.14659500 -2.24133300

**VIII'b**

Pd -0.30701500 -0.54998800 0.54776800

H -0.28419900 -1.91527500 1.26381300

O -0.41916100 1.34772200 -0.65009700

C 0.39798200 2.25694100 -0.88011000

N 1.36402800 2.70816700 -0.07491900

H 0.35451700 2.79722900 -1.83944000

C 2.25511100 3.76471000 -0.51026800

C 1.56321800 2.22452100 1.27462700

H 2.17511800 4.62713800 0.16216900

H 3.29247600 3.40962100 -0.50145400

H 1.99499600 4.07636500 -1.52524100

H 1.44367600 3.05406700 1.98228900

H 0.83273600 1.44186800 1.50336900

H 2.57266600 1.81048000 1.38411200

O -2.20894100 -0.02482800 1.41998400

C -3.29786500 0.25056500 0.87408500

N -3.72490600 -0.05801900 -0.34981100

H -4.03888300 0.81522000 1.45821500

C -5.03152400 0.38834700 -0.79711900

C -2.98454600 -0.85980900 -1.30173500

H -4.92614400 1.00226300 -1.69880600

H -5.66241100 -0.47623200 -1.03424300

H -5.51229100 0.98117800 -0.01518700

H -2.77655800 -0.27358900 -2.20400400

H -2.03549000 -1.19686400 -0.86698600

H -3.58169300 -1.73656000 -1.57801300

I 2.08322400 -1.32836400 -0.31756200

**TS_VIII'a-IX'_**

C -5.55469800 -0.43518300 -0.81629300

C -5.41044900 -1.40489400 0.18479600

C -4.15264300 -1.60025200 0.73836500

C -3.03256200 -0.86408900 0.32766100

C -3.18685400 0.17486500 -0.64544000

C -4.47396400 0.32796700 -1.22647500

H -6.52957900 -0.26847900 -1.27591700

H -6.26105100 -2.00217000 0.50791400

H -3.99336200 -2.36046100 1.50381000

H -4.59699100 1.10149100 -1.98428100

C -1.71027000 -1.27548100 0.87130700

C -0.54919900 -1.08009800 -0.02129300

H -0.81009300 -1.30645200 -1.06570900

O -1.61588100 -1.82107600 1.98091300

O -2.24698000 1.01960400 -0.99063900

C 0.76647200 -1.70965200 0.42353000

H 0.55360100 -2.65991500 0.93891300

C 1.71680600 -2.00770400 -0.72172300

C 2.59143900 -3.09766300 -0.63483800

C 1.79038900 -1.19141100 -1.85769300

C 3.51289600 -3.36314400 -1.64509100

H 2.54496600 -3.74770000 0.24022000

C 2.71182000 -1.45295800 -2.86966800

H 1.11707500 -0.33721800 -1.94741700

C 3.57839600 -2.53905900 -2.76774100

H 4.17941400 -4.22014000 -1.55655500

H 2.74869000 -0.80522100 -3.74471200

H 4.29703200 -2.74523600 -3.55940000

Pd -0.37451700 0.95397900 -0.07094900

C 1.42588000 -0.78019700 1.41744000

C 2.09654500 -1.25005200 2.54544600

C 1.27650100 0.61550300 1.20262800

C 2.61982800 -0.36051600 3.48385000

H 2.18942500 -2.32526700 2.70642000

C 1.79108300 1.48900100 2.17983000

H 1.56346500 1.30670400 -0.00451600

C 2.45756300 1.01299900 3.30599600

H 3.14198400 -0.74086000 4.36099200

H 1.68972400 2.56630300 2.03734300

H 2.85457800 1.71170800 4.04142100

C 1.28099600 3.21532700 -0.81250400

O 0.07631500 3.10438300 -0.45834300

O 2.13917000 2.26507100 -0.74957200

C 1.77988200 4.53922700 -1.31436800

H 0.95387200 5.18889000 -1.61425000

H 2.33861000 5.03883900 -0.51343500

H 2.47028200 4.40306300 -2.15240400

**TS_VIII'b-I_**

Pd 0.91829600 -0.14672800 -0.39645300

H 2.14469900 1.03670900 -0.53892600

O -0.50381600 -2.27395700 -0.40988800

C -1.72602800 -2.12568700 -0.47333100

N -2.48961100 -1.51104200 0.45236900

H -2.32023100 -2.48925000 -1.33452300

C -3.93166000 -1.49852800 0.35350200

C -1.89040300 -0.99388600 1.66344000

H -4.38548200 -2.12846200 1.13032700

H -4.31560400 -0.47766700 0.47228900

H -4.23571300 -1.87726600 -0.62716900

H -1.89374700 -1.74695600 2.46369100

H -0.85179800 -0.70304700 1.45385500

H -2.45418200 -0.11898100 2.00288900

O -0.81696400 0.60787800 -1.45893200

C -1.88434600 1.10914300 -1.06068300

N -2.07936600 1.86932000 0.02392400

H -2.80512800 0.94984900 -1.64437700

C -3.39021700 2.41510700 0.31172900

C -1.00399100 2.28026800 0.90075000

H -3.72138100 2.09991300 1.30896000

H -3.35941000 3.51144400 0.28733400

H -4.11075900 2.06339200 -0.43203000

H -1.34449100 2.23030100 1.94088000

H -0.13633100 1.61384800 0.76413200

H -0.69864600 3.31067600 0.67801900

O 2.78079800 -0.79152900 0.47979300

C 3.64878200 0.10865000 0.33286700

O 3.39922600 1.22448500 -0.25774700

C 5.03750600 -0.09166900 0.84649600

H 5.12671200 -1.02798400 1.40123900

H 5.74474400 -0.10445600 0.00929300

H 5.32617200 0.74405200 1.49248500

**IX'**

C 5.35077400 1.04939900 -0.12912300

C 4.96345900 1.32640000 1.18959600

C 3.66354300 1.02785800 1.57132200

C 2.72800500 0.46816000 0.68764600

C 3.13770700 0.11424700 -0.64315100

C 4.46416500 0.46962400 -1.01942900

H 6.36404200 1.28337300 -0.45884600

H 5.66131400 1.77726800 1.89281100

H 3.31975000 1.24355000 2.58324000

H 4.77755100 0.22577600 -2.03485700

C 1.34002900 0.30995700 1.20140200

C 0.21888000 0.41933100 0.22640100

H 0.42268400 1.19607400 -0.52607200

O 1.13142600 0.18188000 2.41779900

O 2.39976300 -0.54476500 -1.49109400

C -1.19327300 0.51347200 0.82585400

H -1.08300500 0.79711000 1.88446500

C -2.02679300 1.58257200 0.15117700

C -2.60831900 2.61269100 0.89510600

C -2.25305400 1.55232700 -1.23196300

C -3.39182100 3.58934100 0.28013600

H -2.44092200 2.64792400 1.97261400

C -3.03006500 2.52680300 -1.85079100

H -1.80703100 0.75052600 -1.82496300

C -3.60435200 3.55044400 -1.09546300

H -3.83524900 4.38380000 0.87918900

H -3.19100800 2.48798200 -2.92760400

H -4.21332400 4.31268500 -1.57918200

Pd 0.50273700 -1.27208000 -0.84201900

C -1.86996900 -0.83578900 0.74327400

C -3.08063800 -1.11027700 1.38383600

C -1.23598600 -1.81971500 -0.05513300

C -3.68169000 -2.36242700 1.25315100

H -3.56045000 -0.33779900 1.98971500

C -1.85947800 -3.07319700 -0.17023100

C -3.07014300 -3.34396000 0.47259900

H -4.62359000 -2.57189800 1.75963900

H -1.39512100 -3.85739400 -0.77356300

H -3.53695900 -4.32378900 0.36535500

**1**

C 4.68192600 0.04505300 0.00002100

C 3.47200900 -0.66279900 0.00000700

C 2.23225500 0.04151800 -0.00001600

C 2.27859000 1.45086200 -0.00000400

C 3.47661400 2.14158300 0.00002600

C 4.68189000 1.42858900 0.00003400

H 5.61021700 -0.52234300 0.00002200

H 1.35251900 2.01910500 -0.00001900

H 3.48030100 3.22907100 0.00004100

H 5.63048800 1.96340600 0.00005500

O 3.53940500 -1.99925900 0.00000700

H 2.59006100 -2.31076800 -0.00000300

C 0.97207200 -0.71560100 -0.00003500

C -0.31287700 -0.01347600 -0.00006000

H -0.32633400 1.07318700 -0.00007700

C -1.46820100 -0.71523200 -0.00001200

H -1.37693800 -1.80404500 0.00002300

O 0.99200400 -1.97028500 -0.00004000

C -2.82431600 -0.20346500 0.00000600

C -3.13928200 1.16936800 -0.00002600

C -3.88190900 -1.13113600 0.00004800

C -4.46190800 1.58818800 -0.00002200

H -2.34128200 1.91055800 -0.00004200

C -5.20544000 -0.70985200 0.00004500

H -3.64609000 -2.19508700 0.00009000

C -5.49960200 0.65248100 0.00001000

H -4.69059500 2.65245100 -0.00004500

H -6.00946900 -1.44364800 0.00006900

H -6.53507300 0.98798500 0.00001800

**X'**

C 5.14235300 -1.44544300 0.79784100

C 3.95705900 -2.05099800 1.23459200

C 2.74245500 -1.55621600 0.79453100

C 2.66585900 -0.45395500 -0.07895100

C 3.88053200 0.16485700 -0.48667300

C 5.10827100 -0.35194300 -0.05078600

H 6.10416800 -1.82982100 1.13376100

H 3.99013400 -2.90062400 1.91260500

H 1.82073700 -2.01622200 1.14523600

H 6.02250700 0.13343500 -0.38632900

C 1.37937900 0.08192400 -0.54661400

C 0.15691200 -0.68362000 -0.27361200

H 0.27615200 -1.76533100 -0.23764600

O 1.33909100 1.13900400 -1.21562600

O 3.90318200 1.24041100 -1.28402500

H 2.94576800 1.47434900 -1.43993200

C -1.10066500 -0.18142600 -0.13162600

C -1.36501800 1.25944200 0.07075900

C -0.60214600 2.00286900 0.98290700

C -2.41001400 1.90040600 -0.61003400

C -0.86356700 3.35094600 1.19428700

H 0.19369200 1.50660900 1.53830400

C -2.66022000 3.25486800 -0.41144400

H -3.01926400 1.33148900 -1.31188900

C -1.88993600 3.98368000 0.49287400

H -0.26824400 3.91037500 1.91410300

H -3.46522600 3.74113500 -0.95971300

H -2.09472200 5.04006600 0.65858400

C -2.25414100 -1.10180500 -0.09489200

C -3.39237700 -0.80614200 0.67481100

C -2.23926900 -2.30450800 -0.82271700

C -4.46484400 -1.68944800 0.73092500

H -3.42533700 0.11524400 1.25352300

C -3.31549300 -3.18142300 -0.77326400

H -1.38312300 -2.53753000 -1.45426500

C -4.43190700 -2.87944700 0.00676900

H -5.33071000 -1.44633100 1.34412600

H -3.28876400 -4.10104700 -1.35517400

H -5.27528000 -3.56682900 0.04382200

**142**

C 4.01914900 -1.42158700 -1.61977400

C 3.15010700 -0.38629900 -1.97251800

C 1.78640500 -0.54270200 -1.76906200

C 1.24911900 -1.72959800 -1.23291500

C 2.14782100 -2.79813900 -0.94993100

C 3.52630700 -2.61503300 -1.11191600

H 5.09278700 -1.30428600 -1.76198400

H 3.53593700 0.53859900 -2.39560500

H 1.11215000 0.25780600 -2.06775300

H 4.19037500 -3.43910400 -0.85855500

C -0.19197400 -1.92062100 -0.96642300

C -1.06154700 -0.76848900 -0.94003900

H 0.71726800 -3.93865300 -0.57615700

O -0.65474500 -3.08713300 -0.82796100

O 1.71670400 -3.99060500 -0.51544600

C -0.68088300 0.45478600 -0.32663300

C -1.31822600 1.68963100 -0.71126900

C -1.75671300 1.84531800 -2.04566200

C -1.49569600 2.76687700 0.18578100

C -2.37887300 3.01432800 -2.45516300

H -1.57682100 1.03519400 -2.75189800

C -2.10469000 3.93832000 -0.23382200

H -1.16837500 2.66093500 1.21976600

C -2.55273600 4.06484900 -1.55222100

H -2.71340200 3.11935800 -3.48539100

H -2.24261800 4.75793600 0.46851300

H -3.02625900 4.98901000 -1.87846100

C -0.29012300 0.20383700 1.08139600

C -1.40905800 -0.45028800 1.70125000

C 0.85773800 0.53547000 1.81791900

C -1.35134000 -0.70313700 3.07205400

C 0.87732600 0.21504100 3.18703300

C -0.21350800 -0.37700400 3.80560700

H -2.18791600 -1.18879600 3.57108100

H 1.76278300 0.46024200 3.77104500

H -0.17693900 -0.59834300 4.87049600

C 2.06634300 1.14049000 1.21718900

C 3.33113300 0.65826300 1.58798900

C 2.00314800 2.20573600 0.30750200

C 4.49289900 1.22234200 1.06963500

H 3.40031100 -0.18465600 2.27519000

C 3.16508300 2.77532600 -0.20404300

H 1.03708200 2.61348700 0.01746600

C 4.41488100 2.28583200 0.17249600

H 5.46256600 0.82432600 1.36476200

H 3.09196200 3.61039600 -0.89908000

H 5.32280500 2.72976200 -0.23242500

C -2.60049000 -0.84305100 0.88825800

C -2.47048200 -1.00759500 -0.52371000

C -3.84299500 -1.10889700 1.47114200

C -3.54307700 -1.45299900 -1.28696200

C -4.92105400 -1.52479500 0.69158700

H -3.98345800 -0.97784600 2.54327200

C -4.77468700 -1.70366200 -0.68242800

H -3.41624600 -1.58379000 -2.36076600

H -5.88430200 -1.70725500 1.16467800

H -5.62119900 -2.02772600 -1.28502500

**4**

C -4.20979600 -2.56467400 2.09318400

C -3.26587900 -1.61559900 2.51167600

C -2.55319200 -0.90893100 1.56204800

C -2.75724000 -1.12337700 0.18341600

C -3.71451400 -2.09345900 -0.22134600

C -4.43443100 -2.80410500 0.74808300

H -4.77520600 -3.12707000 2.83451700

H -3.09544200 -1.43987800 3.57106200

H -1.81101500 -0.17413000 1.87378600

H -5.16236800 -3.54162800 0.41731300

C -2.00235400 -0.38077400 -0.81985200

C -1.02436100 0.68079700 -0.38795400

O -2.16149500 -0.56975300 -2.03864200

O -3.95865400 -2.36158200 -1.50952000

C 0.32648100 0.41864500 -0.38073700

C 0.81151000 -0.89294100 -0.88899100

C 0.43041600 -2.11319200 -0.31893000

C 1.64244500 -0.91146400 -2.01776900

C 0.87819200 -3.31810400 -0.85482100

H -0.18927000 -2.11734300 0.57727200

C 2.08559700 -2.11477600 -2.55697100

H 1.94613300 0.03287600 -2.47010100

C 1.70831800 -3.32371800 -1.97408700

H 0.58504700 -4.25669200 -0.38668800

H 2.73012000 -2.10776100 -3.43459800

H 2.06116200 -4.26621100 -2.38920700

C 1.26316300 1.48039100 -0.06468000

C 0.79568300 2.83023200 -0.05527000

C 2.64872600 1.23481000 0.20324400

C 1.73761000 3.87937800 -0.01211100

C 3.53581100 2.30421700 0.21827100

C 3.08942700 3.62022700 0.06731200

H 1.40358600 4.91257100 -0.04825800

H 4.58900600 2.10953600 0.41813300

H 3.80312200 4.44191600 0.07932700

C 3.17199700 -0.07769900 0.64824600

C 4.34497700 -0.62226200 0.10963900

C 2.54932500 -0.74979600 1.70968900

C 4.87034000 -1.81159900 0.60714600

H 4.83475900 -0.11568900 -0.72241800

C 3.08228700 -1.92977300 2.21768500

H 1.63805900 -0.33458400 2.14238400

C 4.24369400 -2.46798400 1.66557300

H 5.77497100 -2.22747800 0.16578200

H 2.58634100 -2.43244500 3.04672100

H 4.65869500 -3.39490200 2.05778100

C -0.63099400 3.09445500 -0.06730800

C -1.54083700 2.00421800 -0.16824300

C -1.16317200 4.39573900 0.05572400

C -2.93129800 2.25896900 -0.13752700

C -2.52559200 4.61867000 0.08988900

H -0.49612000 5.24921300 0.14394200

C -3.41896200 3.54221300 -0.00690000

H -3.63193800 1.42922900 -0.21843100

H -2.90388800 5.63371800 0.19553300

H -4.49294000 3.71650500 0.01938100

H -3.35189700 -1.77148000 -2.03189800

TS_VI'-VII'c_

C 5.48154000 0.61521400 -0.93679000

C 5.15835100 1.87048600 -0.42154200

C 3.92386900 2.07067700 0.18418400

C 2.98405700 1.03713700 0.26395700

C 3.35173700 -0.21724300 -0.22742900

C 4.58209000 -0.44106500 -0.83496700

H 6.44681600 0.44733700 -1.40973900

H 5.86846900 2.69101400 -0.49460600

H 3.65605400 3.04603000 0.58710800

H 4.84154800 -1.43352500 -1.20111900

C 1.65169800 1.31288600 0.88308200

C 0.43602200 1.03393900 0.07209300

H 0.58096600 1.35551800 -0.97056500

O 1.60813500 1.83690300 1.99682100

O 2.47083400 -1.25875700 0.00784400

C -0.86253100 1.51799400 0.71513800

H -0.60197800 2.31105000 1.43078500

C -1.86108500 2.08340600 -0.26660100

C -2.68900900 3.14188200 0.12414500

C -2.00534000 1.56743400 -1.55933200

C -3.63229800 3.67346700 -0.75168400

H -2.58496400 3.55583800 1.12787900

C -2.95038100 2.09464600 -2.43737700

H -1.38082100 0.73212800 -1.87803000

C -3.76604900 3.15107200 -2.03729200

H -4.26078900 4.50313800 -0.43110300

H -3.04794200 1.67787000 -3.43893600

H -4.50019400 3.56785300 -2.72484600

Pd 0.28211700 -1.00844500 -0.23099200

C -1.41214600 0.35094400 1.54118300

C -0.98700300 0.16785800 2.86500500

C -2.28291200 -0.59671300 0.97944800

C -1.41446900 -0.93144700 3.60249200

H -0.31393800 0.90022400 3.30742500

C -2.70060300 -1.70615600 1.71813700

H -2.65215300 -0.45651400 -0.03446800

C -2.26582300 -1.87839100 3.02859000

H -1.08394900 -1.05050900 4.63340400

H -3.37247700 -2.43203200 1.26095800

H -2.59483000 -2.73967800 3.60752400

C -1.08322100 -2.85782900 -1.48344400

O -0.08297800 -3.08970600 -0.71810000

O -1.44021900 -1.67223500 -1.73712300

C -1.83841400 -4.02146100 -2.04570700

H -2.38468200 -4.52640200 -1.23950700

H -2.55571600 -3.70522600 -2.80768100

H -1.15015100 -4.75750000 -2.47460600

H 2.75870900 -2.06019300 -0.47151500

VI’

C -3.53339800 3.89397100 0.64271500

C -2.43027500 4.66275700 0.26890400

C -1.30946800 4.04087300 -0.26771200

C -1.25804100 2.65042900 -0.41686600

C -2.39725700 1.90360100 -0.07671600

C -3.52777100 2.51511500 0.46324700

H -4.41637700 4.37119000 1.06382900

H -2.44431100 5.74233300 0.40111800

H -0.43640800 4.62516600 -0.55446600

H -4.40163400 1.91609000 0.71340300

C -0.00050800 2.01833600 -0.91705600

C 0.57616100 0.92790600 -0.08896600

H 0.56611300 1.17757400 0.98148100

O 0.55793000 2.44328500 -1.92755800

O -2.40930500 0.57263400 -0.39600800

H -3.10828100 -0.00409500 0.06430300

C 1.82529300 0.24438300 -0.60223300

H 1.88633800 0.43817100 -1.68039600

C 3.15433200 0.61453900 0.01597100

C 4.30045100 0.61166500 -0.78762800

C 3.29383000 0.90785300 1.37698700

C 5.55331600 0.89192400 -0.24951300

H 4.20224400 0.38481000 -1.85023800

C 4.54660400 1.18986300 1.91822300

H 2.41891000 0.91859500 2.02694300

C 5.68018100 1.18196200 1.10797500

H 6.43213000 0.88712800 -0.89235900

H 4.63535700 1.41859400 2.97923700

H 6.65802700 1.40352700 1.53238800

Pd -0.69427800 -0.67702400 -0.08483500

C 1.46474900 -1.23139900 -0.42858400

C 1.54834600 -2.14668100 -1.50840600

C 1.12592800 -1.73278300 0.86248600

C 1.35206900 -3.49717400 -1.29905700

H 1.80473200 -1.76690900 -2.49703100

C 0.93486900 -3.11829300 1.05255900

H 1.20270700 -1.08125500 1.73285400

C 1.04695300 -3.98616000 -0.01436300

H 1.44336600 -4.19192800 -2.13194800

H 0.70583400 -3.49091100 2.04888000

H 0.90574200 -5.05490800 0.13463700

C -3.23082300 -2.29629700 0.37871900

O -3.83782300 -1.21428900 0.61432800

O -2.01791800 -2.41182700 0.00192200

C -4.01097700 -3.57733300 0.53423000

H -3.36525800 -4.45907500 0.51599300

H -4.58570500 -3.56253500 1.46644900

H -4.73719000 -3.66120800 -0.28361600

**References**

1. Zhao, Y.; Truhlar, D. G. A New Local Density Functional for Main-Group Thermochemistry, Transition Metal Bonding, Thermochemical Kinetics, and Noncovalent Interactions. *J. Chem.* *Phys.* **2006**, *125* (19), 194101.
2. Cancès, E.; Mennucci, B.; Tomasi, J. A new integral equation formalism for the polarizable continuum model: Theoretical background and applications to isotropic and anisotropic dielectrics. *J. Chem. Phys*. **1997**, *107*, 3032−3041.
3. Cossi, M.; Barone, V.; Cammi, R.; Tomasi, J. Ab initio study of solvated molecules: a new implementation of the polarizable continuum model. *Chem. Phys. Lett.* **1996**, *255*, 327−335.
